# Identification of miRNA Regulatory Networks and Candidate Markers for Fracture Healing in Mice

**DOI:** 10.1155/2021/2866475

**Published:** 2021-11-16

**Authors:** Xianglu Li, Zhaohua Zhong, Enguang Ma, Xiaowei Wu

**Affiliations:** ^1^Department of Cadre Ward, The Second Affiliated Hospital, Harbin Medical University, Harbin 150001, China; ^2^Department of College of Basic Medicine, Harbin Medical University, Harbin 150001, China; ^3^Department of Urinary Surgery, Harbin First Hospital, Harbin 150010, China

## Abstract

**Background:**

It is important to improve the understanding of the fracture healing process at the molecular levels, then to discover potential miRNA regulatory mechanisms and candidate markers.

**Methods:**

Expression profiles of mRNA and miRNA were obtained from the Gene Expression Omnibus database. We performed differential analysis, enrichment analysis, protein-protein interaction (PPI) network analysis. The miRNA-mRNA network analysis was also performed.

**Results:**

We identified 499 differentially expressed mRNAs (DEmRs) that were upregulated and 534 downregulated DEmRs during fracture healing. They were mainly enriched in collagen fibril organization and immune response. Using the PPI network, we screened 10 hub genes that were upregulated and 10 hub genes downregulated with the largest connectivity. We further constructed the miRNA regulatory network for hub genes and identified 13 differentially expressed miRNAs (DEmiRs) regulators. Cd19 and Col6a1 were identified as key candidate mRNAs with the largest fold change, and their DEmiR regulators were key candidate regulators.

**Conclusion:**

Cd19 and Col6a1 might serve as candidate markers for fracture healing in subsequent studies. Their expression is regulated by miRNAs and is involved in collagen fibril organization and immune responses.

## 1. Introduction

Bone is a complex organ with multiple functions, including hematopoiesis, regulating and storing key minerals, protecting vital organs for life maintenance, and promoting movement. Bone fractures are the most common large organ trauma in humans and a major cause of disability in adults [1]. In the past few years, long bone fractures have become increasingly common, especially because of road traffic injuries. The Centers for Disease Control and prevention states that fractures occupy the top 20 in first-line diagnostics in emergency departments [2]. The management and treatment of fractures substantially increase the costs to the health care system and affect society due to increased morbidity and mortality [3].

Fracture healing begins with injury-induced hematoma and inflammation, which promote condensation of periosteal, endosteal, and bone marrow mesenchymal cells, as well as differentiation into chondrocyte and osteoblast lineages [4]. In the normal response of fracture healing, the granulation tissue formed gradually stiffens with healing until bone formation and is accompanied by the generation of massive callus [5]. The process of fracture healing can be influenced by many biological factors that may influence the development of a fracture. Among them, proper activation of the immune system is indispensable to maintain tissue integrity and promote restoration of homeostasis [6]. The majority of long bone diaphyseal fractures are treated surgically, and then, with the addition of scaffolds, growth factors, and cell therapy, more local bioenhancing can be achieved [7]. Recent data on open long bone fractures show that 17% develop nonunions and an additional 8% develop delayed union [8]. Delayed union and nonunion of fractures impose a significant burden on patients and healthcare systems. Given the limited therapeutic options, screening for effective preventive treatments and candidate markers for early treatment is necessary.

Collectively, these events involve a large number of secreted signaling messengers that activate signaling pathways leading to differential expression of a large number of genes, ultimately resulting in progenitor cell proliferation and differentiation leading to repair of fractured bone [9]. Genetic studies on bone healing demonstrate the molecular complexity of the repair process, with nearly 600 known genes and more than 100 novel genes [10]. A direct comparison of the fracture healing process with the gene expression changes without fracture may provide more insight.

MicroRNAs (miRNAs) are small, single stranded, noncoding RNAs that downregulate target gene expression mainly through mRNA degradation and transcriptional repression [11]. There is accumulating evidence that miRNAs play crucial roles in regulating various biological processes including bone homeostasis [12]. Multiple studies have shown that miRNAs are involved in fracture repair [13, 14]. The current understanding of how miRNAs regulate the expression of genes at the molecular level and thereby affect fracture healing is not well established. Therefore, there is an urgent need to understand the underlying causes of fracture healing and identify new targets for therapeutic intervention to promote optimal bone repair after fracture.

This study analyzed gene expression profiles during fracture healing in mice using microarray data from public databases to better understand the underlying mechanisms of fracture healing. Using a bioinformatics approach, we identified the regulatory roles of miRNAs during fracture healing. Potential targets of action and their biological functions were explored by enrichment analysis and protein-protein interaction (PPI) network analysis.

## 2. Materials and Methods

### 2.1. Data Collection

The data used for this study were obtained from the Gene Expression Omnibus (GEO) database. The GSE99388 dataset included mRNA expression profile of mouse tibial fractures at 0, 5, 10, and 20 days postfracture (DPF) on the platform of GPL6246. The whole diaphyseal bone was used for the time point 0 (prefracture), the callus after tibial fractures was collected for three time points (5 days postfracture, 10 days postfracture, and 20 days postfracture). The GSE76197 dataset included miRNA expression profile of mouse tibial fractures at 1, 3, 5, 7, 11, and 14 DPF, as well as intact (unfractured bone) on the platform of GPL21265.

### 2.2. Difference Analysis

Differential expression analysis was performed using the limma R package between 5, 10, 20 days, and 0 day after fracture, respectively. The mRNAs with a ∣log2(FoldChange) | >1 and false discovery rate (FDR) < 0.05 were assigned as differentially expressed mRNAs (DEmRs). The miRNAs with a ∣log2(FoldChange) | >1 and *P* value < 0.05 between 3, 5, 7, 11, 14 days, and 1 day after fracture were assigned as differentially expressed miRNAs (DEmiRs), respectively.

### 2.3. Enrichment Analysis

Gene Ontology (GO) and Kyoto Encyclopedia of Genes and Genomes (KEGG) enrichment analyses of DEmRs were performed using Search Tool for the Retrieval of Interacting Genes (STRING) (http://string-db.org/) database. The *P* value < 0.05 was considered significantly enriched.

### 2.4. Construction of Protein-Protein Interaction (PPI) Network

The PPI network of DEmRs was identified through the STRING database. The combined score > 0.5 was considered significant. Gephi software was used to visualize the PPI network. The hub genes were chosen based on their degree of connectivity with other genes.

### 2.5. Prediction of miRNA Regulators for mRNA

The miRNA regulators of DEmRs were predicted using miRDB online database (http://mirdb.org/). The expression changes of DEmRs in the regulatory network were utilized to identify key target mRNAs.

## 3. Results

### 3.1. mRNA Expression Changes in Fracture Healing in Mice

**Figure 1:**
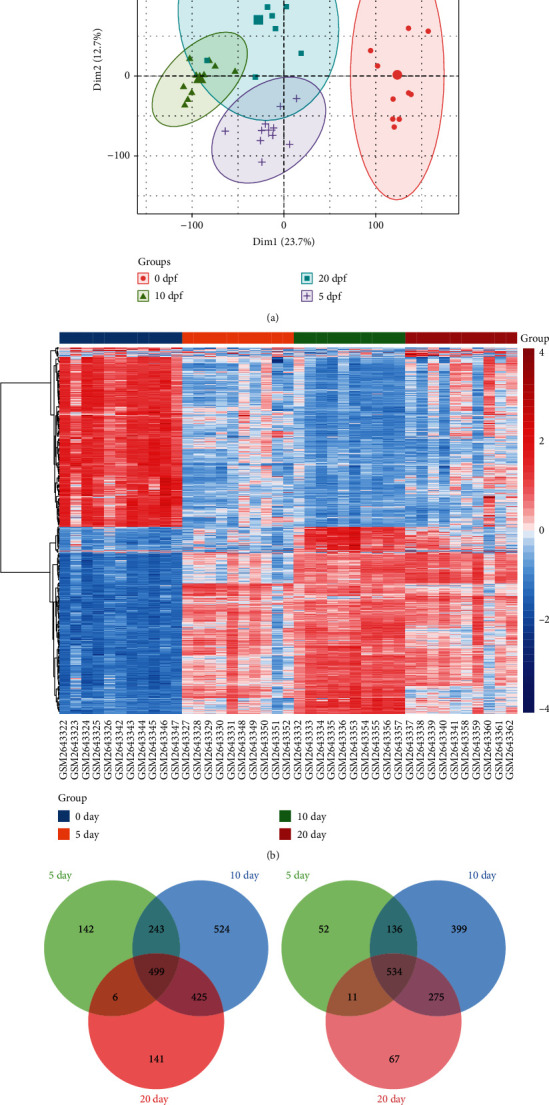
Differentially expressed genes in mice after fracture. (a) Principal component analysis of fracture samples from mice at different time points. (b) Heatmap of mRNA expression in samples at different time points. Red represents upregulation and blue represents downregulation. Differentially expressed mRNAs between different time points after fracture and 0 day. Intersection mRNAs that were upregulated (c) and downregulated (d) simultaneously in the three groups of differences.

First, we performed the principal component analysis (PCA) on samples from mice at different time points after fracture in the GSE99388 dataset. The distance between the time point 0 (prefracture) and the samples at other time points, especially day 10 (Figure 1(a)), was obtained. We then constructed a heatmap of gene expression at different time points, observing gene expression across all samples (Figure 1(b)). Suggesting differences in the expression of the genes we acquired at different time points. Using differential expression analysis, we obtained 1624, 3036, and 1959 differentially expressed mRNAs (DEmRs) between 5 (Table S1), 10 (Table S2), and 20 (Table S3) days and 0 days, respectively. Among them, 499 genes were upregulated and 534 genes were downregulated simultaneously in the three groups of DEmRs (Figures 1(c) and 1(d)). These common upregulated or common downregulated genes may be involved in fracture healing in mice.

### 3.2. Biological Functions of Fracture Healing

**Figure 2:**
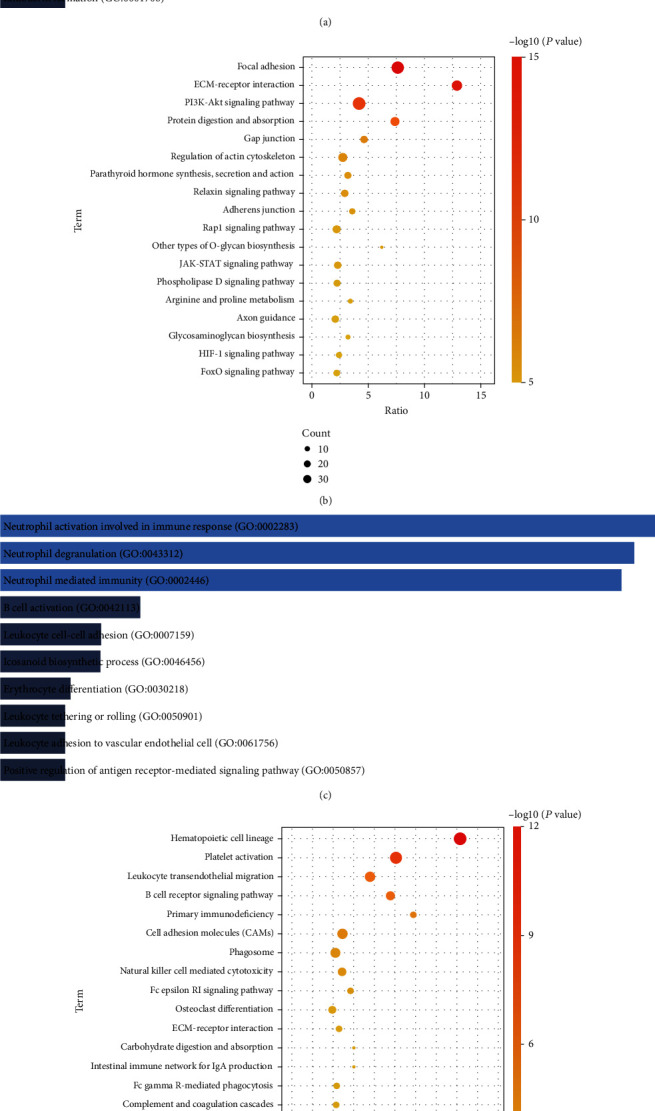
Enrichment results of mRNAs with altered expression during fracture repair. (a) The top ten biological processes of common upregulated mRNAs. (b) KEGG pathway of common upregulated mRNAs. (c) The top ten biological processes of common downregulated mRNAs. (d) KEGG pathway of common downregulated mRNAs.

We performed enrichment analysis for common upregulated mRNAs and common downregulated mRNAs, respectively. The terms of enrichment results were ranked by *P* value. GO enrichment results showed that the upregulated mRNAs were mainly involved in the biological processes of extracellular matrix organization and collagen fibril organization (Figure 2(a)). In KEGG enrichment results, focal adhesion, ECM receptor interaction, and PI3K-Akt signaling pathway were significantly enriched by the upregulated mRNAs (Figure 2(b)). The downregulated mRNAs were mainly enriched in biological processes of neutrophil activation induced in immune responses and neutrophil degradation (Figure 2(c)) and KEGG signaling pathways for hematopoietic cell lineage, platelet activation, and leukocyte transendothelial migration (Figure 2(d)).

### 3.3. PPI Network of Common DEmRs

**Figure 3:**
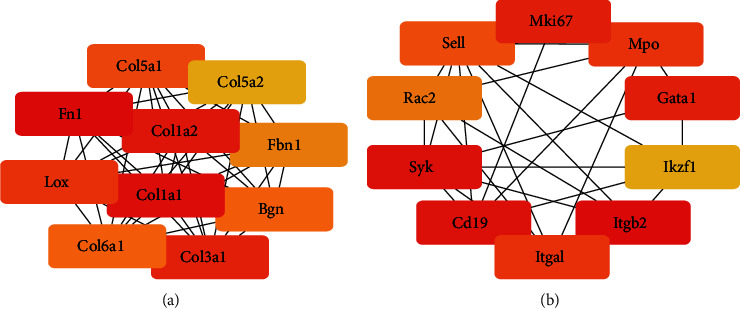
PPI network of common upregulated or downregulated mRNAs. (a) The top 10 degree in the PPI network of the common upregulated mRNAs. (b) The top 10 degree in the PPI network of the common downregulated mRNAs. The redder the color, the greater the degree of connectivity in the PPI network.

Utilizing the STRING database, we constructed PPI networks for common upregulated mRNAs and common downregulated mRNAs, respectively (Figure S1, S2). In the PPI network of common upregulated mRNAs, we identified the top 10 mRNAs with the largest degree of connectivity as hub genes (Figure 3(a)). Fn1, Col1a1, Col1a2, Col3a1, Lox, Col5a1, Bgn, Col6a1, Fbn1, and Col5a2 were included. In the PPI network of common downregulated mRNAs, we similarly identified 10 hub genes (Figure 3(b)), including Itgb2, Syk, Cd19, Mki67, Gata1, Itgal, Mpo, Sell, Rac2, and Ikzf1.

### 3.4. miRNA Regulators of Hub Genes

**Figure 4:**
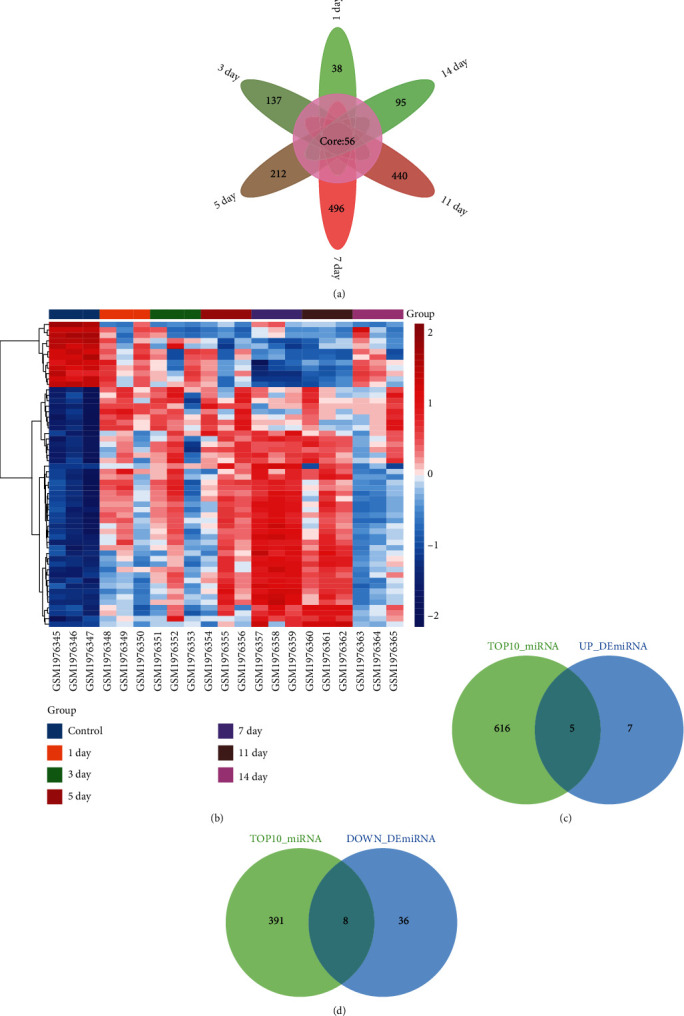
miRNA regulation of hub genes. (a) Differentially expressed miRNAs between mice at different time points after fracture and controls. Intersection genes were considered as core miRNAs. (b) Expression heatmap of core miRNAs in different groups. (c) Intersection between upregulated hub genes predicted miRNA regulators and downregulated DEmiRs. (d) Intersection between downregulated hub genes predicted miRNA regulators and upregulated DEmiRs.

By differential analysis, we obtained differentially expressed miRNAs (DEmiRs) between different time points after fracture in mice and controls in the GSE76197 dataset (Figure 4(a)). Fifty-six miRNAs were simultaneously present in the six groups of DEmiRs and were identified as core miRNAs. The expression of core miRNAs differed at different time points after fracture (Figure 4(b)). Among them, 44 DEmiRs were upregulated and 12 DEmiRs were downregulated after fracture compared with the control. Furthermore, to identify the regulators of hub genes, we predicted their targeted miRNA regulators using the miRDB database. The upregulated hub genes predicted 621 miRNA regulators, and the downregulated hub genes predicted 399 miRNA regulators. By comparing the predicted miRNA regulators of the upregulated hub genes with the downregulated DEmiRs, we identified 5 upregulated DEmiR regulators (Figure 4(c)). Similarly, we obtained upregulated DEmiR regulators regulating 8 downregulated hub genes (Figure 4(d)).

### 3.5. Regulatory Network of miRNAs Targeted mRNAs

**Figure 5:**
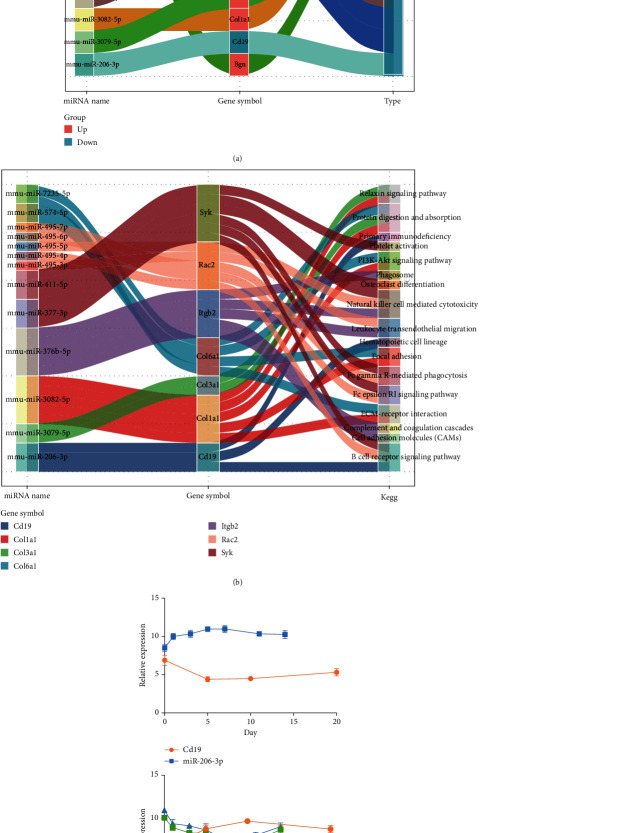
Identification of key regulatory pairs in the regulatory network of miRNA targeted mRNAs. (a) Targeted regulatory relationship between DEmiRs and hub genes. (b) DEmiRs participated in the KEGG signaling pathway by targeting hub genes. (c) Expression levels of key DEmiR regulators and mRNAs at different time points after fracture in mice.

Further, we showed 13 DEmiR regulators targeted regulation of 10 hub genes (Figure 5(a)). The upregulated targeted mRNAs (Col6a1, Col3a1, Col1a1, and Bgn) and downregulated targeted mRNAs (Cd19, Gata1, Ikzf1, Itgb2, Rac2, and Syk) were included. By constructing an integrated regulatory network, we identified the KEGG signaling pathways involved in these targeted mRNAs (Figure 5(b)). Among them, Cd19 and Col6a1 showed the biggest differential fold change and were considered the key target mRNAs, while miR-206-3p, miR-7235-5p, and miR-574-5p were the key regulators. Cd19 and Col6a1 showed the most obvious expression changes on day 10, and miR-206-3p, miR-7235-5p, and miR-574-5p showed the most obvious changes on day 7 (Figure 5(c)).

## 4. Discussion

Scientific advances in a deeper understanding of the molecular processes underlying fracture healing have led to the identification of key mediators that could be potential targets to promote bone regeneration. Using transcriptome data from public databases, we identified potential target genes during fracture healing in mice. Prior to analysis, we utilized PCA to examine the distance between samples with respect to control and postfracture callus. This makes the differential expression analysis of further genes valid. Between the different time points postfracture and the controls, we found 499 genes upregulated and 534 genes downregulated at the postfracture. The results of enrichment analysis showed that these genes were mainly associated with immune response, inflammatory response, and collagen fibril organization equal to tissue repair related biological roles. In addition, we also identified the regulatory network of key mRNAs by miRNAs.

Upregulated mRNAs were significantly enriched in the collagen fibril organization. There is a strong correlation between bone collagen production and connectivity with cortical bone fracture toughness, and collagen is a tool for fracture prevention and evaluation [15]. Focal adhesion is essential for bone formation and osteoblast migration during the physiological process of fracture healing [16]. ECM receptor interactions are enriched in fracture-related studies and participate in bone remodelling [17]. Activation of the PI3K-Akt signaling pathway has been shown to be associated with osteogenic differentiation [18]. From this, it was inferred that the upregulated genes might benefit postfracture repair by promoting bone formation.

The initial cellular components of the fracture milieu include immune cells such as platelets, erythrocytes, leukocytes, and neutrophils, which form the hematoma and contribute to the optimization of the healing process [6]. Neutrophils play a crucial regulatory role on the immune response at the fracture site, resolving inflammation, and inducing downstream responses that are essential for the success of bone repair [19]. These biological roles related to fracture healing were enriched by downregulated genes.

Using the PPI network, we identified the top 10 mRNAs with the largest connections for up- and downregulation, respectively, as hub genes, and they may have more extensive effects during fracture healing. To identify the regulatory mechanism of the expression of these hub genes during bone fracture, we predicted some miRNA regulators through online databases. In particular, the differentially expressed miRNAs were screened. This may further identify miRNA regulatory networks involved in fracture healing.

The development of effective targets for the treatment of bone fracture and even the acceleration of the normal physiological repair process are necessary. The results of our analysis show that Cd19 was downregulated the expression in fracture healing. The total number of immune cells increases with time, while B cells (CD19 +) become progressively fewer in fracture healing [20]. There are findings showing that a subpopulation of CD19 + B cells exacerbates local joint bone destruction [21]. The upregulated expression of miR-206-3p in fracture healing was predicted to target regulation of Cd19. miR-206 is dysregulated expressed in osteoporosis and osteonecrosis of the femoral head and may be involved in the differentiation of osteoblasts [22, 23]. Upon deletion of collagen 6A1 (Col6a1), it affects skeletal development and reduces bone density [24]. Collagen 6 is ubiquitous in articular chondrocyte PCM and mediates loading-induced chondrocyte proliferation and expression of chondrogenic genes [25]. miR-574-5p is involved in osteoblast differentiation and considered a new potential biomarker for osteoporosis [12, 26]. Therefore, the downregulated expression of Cd19 and upregulated Col6a1 in the postfracture callus may contribute to fracture healing. As predicted from the expression profiles of Cd19 and Col6a1 and their DEmiR regulators at different time points, the expression changes of genes had a tendency to gradually recover after day 10 of fracture in mice. The results showed that it may be possible to judge fracture healing based on gene expression changes at different times after fracture. This suggests that Cd19 and Col6a1 may be candidate markers and potential therapeutic for the fracture healing.

There are several limitations in this study. First, the data of our analysis were obtained from public databases, and the species was mouse. It remains to be shown whether the candidate genes we identified can be used in humans. Second, the differential expression of candidate genes as well as miRNA regulatory networks requires validation by molecular experiments, which is also the focus of our subsequent studies.

## 5. Conclusion

Our study showed that the mRNA expression changes during fracture healing were regulated by miRNAs. Changes in these genes affect the immune response and blood cell accumulation, and these biological effects explain the molecular regulatory mechanisms of the fracture healing process. We speculate that the expression changes of Cd19 and Col6a1 may serve as potential therapeutic targets for fracture healing. The potential of altered expression of miRNAs in diseases as biomarkers for disease initiation and progression is also suggested.

## Data Availability

The data used during the present study are available from the corresponding author upon reasonable request.

## Supplementary Materials

Supplementary 1 Figure S1: PPI network of the common upregulated mRNAs.

Supplementary 2 Figure S2: PPI network of the common downregulated mRNAs.

Supplementary 3 Table S1: the DEmiRs between 5 days after fracture and 0 day.

Supplementary 4 Table S2: the DEmiRs between 10 days after fracture and 0 day.

Supplementary 5 Table S3: the DEmiRs between 20 days after fracture and 0 day.
