# Supplementary material for: Identification of miRNA Regulatory Networks and Candidate Markers for Fracture Healing in Mice
Source: Comput Math Methods Med. 2021 Nov 16;2021:2866475. doi: 10.1155/2021/2866475 (PMC8611357; doi:10.1155/2021/2866475)
Supplement: Supplementary 3 — Table S1: the DEmiRs between 5 days after fracture and 0 day. [file 2866475.f3.docx]

**Table S1.** The DEmiRs between 5 days after fracture and 0 day.

|  | logFC | AveExpr | t | P.Value | adj.P.Val | Symbol |
| --- | --- | --- | --- | --- | --- | --- |
| 10547657 | 3.418967 | 7.786585 | -21.854 | 1.48E-16 | 2.35E-12 | C3ar1 |
| 10567355 | 2.249716 | 6.040499 | -21.7536 | 1.63E-16 | 2.35E-12 | Gprc5b |
| 10560709 | 2.204173 | 6.607261 | -21.3434 | 2.44E-16 | 2.35E-12 | Pvr |
| 10461022 | 1.39051 | 9.253552 | -20.9479 | 3.64E-16 | 2.62E-12 | Ppp1r14b |
| 10534667 | 4.045577 | 7.368074 | -20.4704 | 5.94E-16 | 2.82E-12 | Serpine1 |
| 10379518 | 4.24549 | 7.776467 | -20.3416 | 6.79E-16 | 2.82E-12 | Ccl7 |
| 10379530 | 3.583382 | 6.953528 | -20.3329 | 6.86E-16 | 2.82E-12 | Ccl12 |
| 10456400 | 2.773264 | 7.949869 | -19.8125 | 1.19E-15 | 3.76E-12 | Tubb6 |
| 10528143 | 1.442112 | 8.924291 | -19.7367 | 1.29E-15 | 3.76E-12 | Ppp1r14b |
| 10540085 | 2.930284 | 7.653375 | -19.7257 | 1.30E-15 | 3.76E-12 | Fbln2 |
| 10438064 | -3.86546 | 6.904023 | 19.60561 | 1.48E-15 | 3.88E-12 | Vpreb1 |
| 10422728 | 2.604538 | 9.077268 | -19.344 | 1.97E-15 | 4.73E-12 | Dab2 |
| 10521824 | 2.372934 | 6.54826 | -19.2546 | 2.17E-15 | 4.81E-12 | Sod3 |
| 10577757 | 1.602221 | 7.568715 | -19.0925 | 2.60E-15 | 5.34E-12 | Adam9 |
| 10398075 | 3.129541 | 8.408566 | -18.9959 | 2.89E-15 | 5.48E-12 | Serpina3n |
| 10492448 | 2.70997 | 6.674663 | -18.9492 | 3.04E-15 | 5.48E-12 | Ptx3 |
| 10403584 | 2.008547 | 7.351154 | -18.8281 | 3.48E-15 | 5.66E-12 | Nid1 |
| 10598976 | 4.164979 | 9.441404 | -18.7716 | 3.71E-15 | 5.66E-12 | Timp1 |
| 10472050 | 4.102218 | 6.455685 | -18.7665 | 3.73E-15 | 5.66E-12 | Tnfaip6 |
| 10569504 | 2.30032 | 6.484233 | -18.6582 | 4.21E-15 | 5.93E-12 | Tnfrsf23 |
| 10421309 | 2.644322 | 6.661306 | -18.6359 | 4.32E-15 | 5.93E-12 | Slc39a14 |
| 10350173 | 3.161893 | 7.30503 | -18.5096 | 4.98E-15 | 6.29E-12 | Tnnt2 |
| 10395103 | 2.956955 | 6.433909 | -18.5021 | 5.02E-15 | 6.29E-12 | Pxdn |
| 10537146 | 3.175357 | 6.295537 | -18.3528 | 5.96E-15 | 7.03E-12 | Akr1b8 |
| 10460541 | 2.471579 | 7.789785 | -18.3325 | 6.10E-15 | 7.03E-12 | Cd248 |
| 10458382 | 2.814197 | 6.824251 | -18.0794 | 8.16E-15 | 9.04E-12 | Cd14 |
| 10413047 | 1.94103 | 7.914019 | -17.8107 | 1.12E-14 | 1.19E-11 | Plau |
| 10471844 | 2.060163 | 6.802763 | -17.6969 | 1.28E-14 | 1.25E-11 | Nek6 |
| 10603099 | 2.67503 | 6.884468 | -17.6795 | 1.30E-14 | 1.25E-11 | Figf |
| 10410124 | 1.948217 | 9.082094 | -17.6775 | 1.31E-14 | 1.25E-11 | Ctsl |
| 10360406 | 2.774927 | 7.438329 | -17.6044 | 1.42E-14 | 1.32E-11 | Ifi205 |
| 10487040 | 2.94107 | 8.089315 | -17.2251 | 2.24E-14 | 2.02E-11 | Fbn1 |
| 10483706 | 3.246119 | 6.665028 | -17.1377 | 2.49E-14 | 2.18E-11 | Chrna1 |
| 10418506 | 2.122106 | 6.639721 | -17.1107 | 2.57E-14 | 2.18E-11 | Stab1 |
| 10462442 | 2.423336 | 5.656432 | -17.0353 | 2.82E-14 | 2.32E-11 | Il33 |
| 10502522 | 1.556721 | 6.85976 | -16.89 | 3.37E-14 | 2.63E-11 | Hs2st1 |
| 10435641 | 3.156633 | 9.432879 | -16.8862 | 3.39E-14 | 2.63E-11 | Fstl1 |
| 10481175 | 3.459823 | 7.15853 | -16.8362 | 3.60E-14 | 2.63E-11 | Tmem8c |
| 10492136 | 2.595103 | 6.043377 | -16.8179 | 3.68E-14 | 2.63E-11 | Dclk1 |
| 10512949 | 1.722337 | 7.486583 | -16.8078 | 3.73E-14 | 2.63E-11 | Abca1 |
| 10384398 | 2.685217 | 7.428865 | -16.8055 | 3.74E-14 | 2.63E-11 | Grb10 |
| 10558961 | 1.979562 | 7.486868 | -16.7822 | 3.85E-14 | 2.64E-11 | Tspan4 |
| 10513208 | 1.765832 | 6.886396 | -16.6882 | 4.33E-14 | 2.88E-11 | Svep1 |
| 10427471 | 2.09497 | 7.092091 | -16.6526 | 4.52E-14 | 2.88E-11 | Osmr |
| 10542355 | 2.000317 | 8.681987 | -16.6479 | 4.55E-14 | 2.88E-11 | Emp1 |
| 10420254 | -3.32027 | 6.538794 | 16.6404 | 4.59E-14 | 2.88E-11 | Mcpt8 |
| 10345762 | 2.63183 | 8.261191 | -16.6067 | 4.79E-14 | 2.94E-11 | Il1r1 |
| 10461587 | 2.97911 | 6.590773 | -16.5605 | 5.07E-14 | 3.04E-11 | Ms4a4a |
| 10364375 | 1.699287 | 9.351236 | -16.3509 | 6.60E-14 | 3.84E-11 | Cstb |
| 10527158 | 2.802731 | 6.945389 | -16.3441 | 6.66E-14 | 3.84E-11 | Fscn1 |
| 10500948 | 1.305219 | 6.714666 | -16.2801 | 7.22E-14 | 4.08E-11 | Cttnbp2nl |
| 10588037 | 1.782387 | 8.295206 | -16.1801 | 8.20E-14 | 4.54E-11 | Rbp1 |
| 10362538 | 2.109284 | 7.04813 | -16.1574 | 8.44E-14 | 4.59E-11 | Lama4 |
| 10447649 | 2.567838 | 7.624994 | -16.1295 | 8.74E-14 | 4.67E-11 | Fndc1 |
| 10447799 | 1.242084 | 7.11072 | -16.0491 | 9.69E-14 | 5.08E-11 | Igf2r |
| 10563597 | 3.757969 | 6.797411 | -15.9981 | 1.04E-13 | 5.28E-11 | Saa3 |
| 10377018 | 3.732047 | 6.495172 | -15.9905 | 1.05E-13 | 5.28E-11 | Myh3 |
| 10537062 | 3.67193 | 7.445776 | -15.9047 | 1.17E-13 | 5.80E-11 | Mest |
| 10566026 | 2.209223 | 5.846159 | -15.8589 | 1.24E-13 | 6.05E-11 | Folr2 |
| 10381798 | 2.649622 | 8.081576 | -15.7722 | 1.39E-13 | 6.56E-11 | Myl4 |
| 10569335 | 3.445559 | 8.736061 | -15.7716 | 1.39E-13 | 6.56E-11 | H19 |
| 10374083 | 2.805276 | 7.914325 | -15.6918 | 1.54E-13 | 7.16E-11 | Aebp1 |
| 10395163 | 2.075869 | 7.121542 | -15.6145 | 1.70E-13 | 7.80E-11 | Lamb1 |
| 10570957 | 3.824484 | 7.057336 | -15.5832 | 1.78E-13 | 8.00E-11 | Sfrp1 |
| 10496727 | 3.246872 | 6.256111 | -15.5203 | 1.93E-13 | 8.55E-11 | Ddah1 |
| 10414537 | 1.601923 | 7.697103 | -15.4793 | 2.04E-13 | 8.89E-11 | Ang |
| 10458894 | 3.471133 | 8.68713 | -15.4254 | 2.19E-13 | 9.22E-11 | Lox |
| 10515007 | 2.797803 | 7.660876 | -15.4235 | 2.19E-13 | 9.22E-11 | Gpx7 |
| 10537179 | -2.58285 | 8.833714 | 15.41014 | 2.23E-13 | 9.22E-11 | Bpgm |
| 10519998 | 3.632166 | 7.313995 | -15.4079 | 2.24E-13 | 9.22E-11 | Lrrc17 |
| 10487021 | 1.862089 | 6.870381 | -15.3913 | 2.29E-13 | 9.29E-11 | Slc30a4 |
| 10535956 | 1.419082 | 5.658894 | -15.3638 | 2.37E-13 | 9.50E-11 | Stard13 |
| 10407126 | 1.93717 | 6.543683 | -15.3353 | 2.47E-13 | 9.74E-11 | Plk2 |
| 10410931 | 3.860205 | 8.032675 | -15.3206 | 2.52E-13 | 9.80E-11 | Vcan |
| 10402783 | 1.629641 | 6.615295 | -15.2923 | 2.61E-13 | 1.00E-10 | Ahnak2 |
| 10367400 | 1.970015 | 6.508689 | -15.2223 | 2.87E-13 | 1.09E-10 | Mmp19 |
| 10467258 | 2.100506 | 7.255556 | -15.1343 | 3.23E-13 | 1.21E-10 | Myof |
| 10541496 | 3.028047 | 8.92046 | -15.1055 | 3.36E-13 | 1.23E-10 | Mfap5 |
| 10466886 | 2.090463 | 6.681962 | -15.1031 | 3.37E-13 | 1.23E-10 | Glis3 |
| 10412921 | 1.935639 | 6.521512 | -15.0341 | 3.70E-13 | 1.33E-10 | Nid2 |
| 10450325 | 2.09245 | 7.835722 | -15.022 | 3.76E-13 | 1.34E-10 | Cfb |
| 10531675 | 1.224857 | 9.000234 | -15.0056 | 3.84E-13 | 1.34E-10 | Sec31a |
| 10483353 | 2.644225 | 7.034022 | -15.0043 | 3.85E-13 | 1.34E-10 | Scn7a |
| 10439710 | 2.059068 | 7.206639 | -14.9877 | 3.94E-13 | 1.35E-10 | Phldb2 |
| 10557571 | -1.39953 | 6.248941 | 14.96879 | 4.04E-13 | 1.37E-10 | AI467606 |
| 10594066 | 1.893007 | 7.935679 | -14.8624 | 4.67E-13 | 1.56E-10 | Loxl1 |
| 10387855 | -1.33386 | 5.009792 | 14.85754 | 4.70E-13 | 1.56E-10 | Alox15 |
| 10422164 | 2.416777 | 6.202462 | -14.8187 | 4.96E-13 | 1.62E-10 | Ednrb |
| 10443463 | 2.122329 | 8.143063 | -14.8067 | 5.04E-13 | 1.63E-10 | Cdkn1a |
| 10466210 | 3.41093 | 7.328235 | -14.7856 | 5.19E-13 | 1.66E-10 | Ms4a6d |
| 10583071 | 2.505895 | 5.989327 | -14.7774 | 5.25E-13 | 1.66E-10 | Mmp3 |
| 10489759 | 2.266268 | 7.391473 | -14.7632 | 5.35E-13 | 1.68E-10 | Sulf2 |
| 10374366 | 1.845885 | 7.052629 | -14.7393 | 5.53E-13 | 1.71E-10 | Egfr |
| 10498273 | 1.861503 | 7.028717 | -14.6868 | 5.95E-13 | 1.82E-10 | Tm4sf1 |
| 10594679 | 1.085373 | 6.023591 | -14.6754 | 6.04E-13 | 1.83E-10 | Tln2 |
| 10505998 | 1.274641 | 5.65951 | -14.6529 | 6.24E-13 | 1.87E-10 | Fggy |
| 10499394 | 1.516595 | 8.114682 | -14.6404 | 6.35E-13 | 1.88E-10 | Lmna |
| 10507612 | 2.508817 | 7.350311 | -14.6271 | 6.46E-13 | 1.89E-10 | Lepre1 |
| 10598403 | 2.600263 | 8.419363 | -14.614 | 6.58E-13 | 1.90E-10 | Praf2 |
| 10606186 | 2.200459 | 7.001757 | -14.5966 | 6.74E-13 | 1.92E-10 | Slc16a2 |
| 10471535 | 1.713948 | 6.483175 | -14.586 | 6.84E-13 | 1.93E-10 | Fam129b |
| 10467124 | 2.746942 | 7.984772 | -14.567 | 7.03E-13 | 1.96E-10 | Acta2 |
| 10349993 | 3.180271 | 6.352376 | -14.5633 | 7.06E-13 | 1.96E-10 | Myog |
| 10574438 | 1.553615 | 7.76385 | -14.5205 | 7.50E-13 | 2.06E-10 | Cdh5 |
| 10574985 | 1.303782 | 6.954434 | -14.5094 | 7.61E-13 | 2.07E-10 | Slc7a6 |
| 10592342 | 1.238678 | 8.29189 | -14.4962 | 7.76E-13 | 2.09E-10 | Tbrg1 |
| 10366293 | 3.240399 | 7.868273 | -14.4823 | 7.91E-13 | 2.11E-10 | Csrp2 |
| 10578264 | 2.128229 | 7.436649 | -14.4627 | 8.13E-13 | 2.15E-10 | Msr1 |
| 10458303 | 1.69029 | 7.691555 | -14.376 | 9.18E-13 | 2.38E-10 | Ecscr |
| 10375614 | 1.793218 | 6.27562 | -14.3507 | 9.51E-13 | 2.45E-10 | Gfpt2 |
| 10467115 | -2.13245 | 6.263197 | 14.33033 | 9.79E-13 | 2.50E-10 | Ankrd22 |
| 10384223 | 2.10085 | 7.438776 | -14.3215 | 9.91E-13 | 2.51E-10 | Igfbp3 |
| 10536499 | 2.264309 | 8.155268 | -14.3099 | 1.01E-12 | 2.51E-10 | Cav1 |
| 10440522 | 1.884095 | 6.281794 | -14.3084 | 1.01E-12 | 2.51E-10 | Adamts1 |
| 10545958 | 1.490274 | 7.916179 | -14.2735 | 1.06E-12 | 2.60E-10 | Anxa4 |
| 10527649 | 2.572823 | 5.757519 | -14.2718 | 1.06E-12 | 2.60E-10 | Medag |
| 10432243 | 2.526456 | 7.036874 | -14.239 | 1.11E-12 | 2.70E-10 | Fkbp11 |
| 10400006 | 1.281235 | 6.028876 | -14.2211 | 1.14E-12 | 2.74E-10 | Ahr |
| 10364109 | -3.56867 | 6.227967 | 14.21425 | 1.15E-12 | 2.75E-10 | Vpreb3 |
| 10444890 | 1.700796 | 8.067492 | -14.1909 | 1.19E-12 | 2.82E-10 | Ier3 |
| 10606600 | 1.740637 | 5.334782 | -14.1538 | 1.26E-12 | 2.95E-10 | Pcdh19 |
| 10350848 | 1.137273 | 7.401228 | -14.0763 | 1.40E-12 | 3.26E-10 | 2810025M15Rik |
| 10601659 | 2.495661 | 6.988293 | -14.0595 | 1.44E-12 | 3.32E-10 | Srpx2 |
| 10379511 | 3.549859 | 7.579151 | -14.0458 | 1.47E-12 | 3.35E-10 | Ccl2 |
| 10388869 | 1.255271 | 7.025731 | -14.0276 | 1.51E-12 | 3.42E-10 | Tnfaip1 |
| 10492798 | 3.54498 | 8.828155 | -14.0012 | 1.56E-12 | 3.47E-10 | Sfrp2 |
| 10373223 | 1.597749 | 8.408778 | -13.9999 | 1.57E-12 | 3.47E-10 | Lrp1 |
| 10607283 | 1.924173 | 6.457537 | -13.9964 | 1.57E-12 | 3.47E-10 | Maged2 |
| 10564818 | 2.366556 | 7.663227 | -13.9954 | 1.58E-12 | 3.47E-10 | Anpep |
| 10407173 | 1.099348 | 9.329697 | -13.9798 | 1.61E-12 | 3.50E-10 | Il6st |
| 10402268 | 2.382905 | 9.30867 | -13.9791 | 1.61E-12 | 3.50E-10 | Lgmn |
| 10349661 | 1.390854 | 6.03234 | -13.9739 | 1.63E-12 | 3.50E-10 | 5430435G22Rik |
| 10368886 | -1.18724 | 7.871228 | 13.9652 | 1.65E-12 | 3.51E-10 | Foxo3 |
| 10585860 | -1.91087 | 7.85597 | 13.96061 | 1.66E-12 | 3.51E-10 | Adpgk |
| 10553897 | 1.076364 | 5.91212 | -13.9507 | 1.68E-12 | 3.54E-10 | Mtmr10 |
| 10513362 | -1.52489 | 6.406702 | 13.89328 | 1.83E-12 | 3.82E-10 | Susd1 |
| 10519555 | 1.595606 | 5.528315 | -13.8471 | 1.95E-12 | 3.99E-10 | Abcb1b |
| 10502748 | 1.258976 | 6.113256 | -13.8259 | 2.01E-12 | 4.04E-10 | Lphn2 |
| 10490903 | 2.144772 | 6.688916 | -13.822 | 2.03E-12 | 4.04E-10 | Car13 |
| 10417095 | 1.391126 | 5.945443 | -13.8212 | 2.03E-12 | 4.04E-10 | Farp1 |
| 10393823 | 1.293144 | 10.18077 | -13.8199 | 2.03E-12 | 4.04E-10 | P4hb |
| 10353524 | -1.01541 | 7.48736 | 13.80835 | 2.07E-12 | 4.08E-10 | Ogfrl1 |
| 10419850 | -2.14086 | 6.967013 | 13.78439 | 2.14E-12 | 4.19E-10 | Cebpe |
| 10368343 | 2.909473 | 6.15962 | -13.7695 | 2.19E-12 | 4.26E-10 | Arg1 |
| 10466888 | 1.614778 | 6.191021 | -13.7244 | 2.34E-12 | 4.49E-10 | Glis3 |
| 10493449 | 2.169092 | 6.179389 | -13.7228 | 2.34E-12 | 4.49E-10 | Thbs3 |
| 10576639 | 1.089486 | 7.588655 | -13.7159 | 2.36E-12 | 4.49E-10 | Nrp1 |
| 10432540 | 1.368917 | 6.878908 | -13.7149 | 2.37E-12 | 4.49E-10 | Lima1 |
| 10549222 | 2.355276 | 6.583216 | -13.699 | 2.42E-12 | 4.56E-10 | Bcat1 |
| 10489569 | 1.917442 | 8.088039 | -13.6915 | 2.45E-12 | 4.58E-10 | Pltp |
| 10411519 | 1.558169 | 8.169208 | -13.6784 | 2.50E-12 | 4.63E-10 | Map1b |
| 10583100 | -3.01103 | 9.348558 | 13.67632 | 2.51E-12 | 4.63E-10 | Mmp8 |
| 10548905 | 1.440909 | 6.763974 | -13.6341 | 2.67E-12 | 4.89E-10 | Eps8 |
| 10433114 | 2.102224 | 7.216755 | -13.6224 | 2.71E-12 | 4.95E-10 | Itga5 |
| 10537509 | -1.76551 | 5.949137 | 13.61806 | 2.73E-12 | 4.95E-10 | Mgam |
| 10538420 | 1.033136 | 8.872643 | -13.6113 | 2.76E-12 | 4.96E-10 | Gars |
| 10550994 | -2.97041 | 6.001757 | 13.5892 | 2.85E-12 | 5.10E-10 | Ceacam10 |
| 10495054 | 1.816165 | 7.435569 | -13.5765 | 2.90E-12 | 5.16E-10 | Rhoc |
| 10528015 | 2.086719 | 5.281215 | -13.5581 | 2.98E-12 | 5.26E-10 | Steap1 |
| 10460603 | 1.890689 | 8.340813 | -13.5557 | 2.99E-12 | 5.26E-10 | Efemp2 |
| 10355403 | 2.189097 | 10.23699 | -13.5479 | 3.03E-12 | 5.28E-10 | Fn1 |
| 10384370 | -1.15575 | 6.70093 | 13.51033 | 3.20E-12 | 5.55E-10 | Gm12000 |
| 10597413 | 1.994525 | 7.790423 | -13.4863 | 3.31E-12 | 5.72E-10 | Crtap |
| 10578829 | 1.457001 | 7.263005 | -13.4658 | 3.42E-12 | 5.86E-10 | Palld |
| 10436487 | 1.942804 | 5.871759 | -13.4506 | 3.49E-12 | 5.94E-10 | Vgll3 |
| 10492078 | 1.511227 | 7.878317 | -13.4492 | 3.50E-12 | 5.94E-10 | Alg5 |
| 10534168 | 1.284881 | 5.786265 | -13.4005 | 3.76E-12 | 6.31E-10 | Auts2 |
| 10581434 | 1.788001 | 5.422335 | -13.3425 | 4.10E-12 | 6.84E-10 | Dpep2 |
| 10346747 | 1.306351 | 6.931634 | -13.3354 | 4.15E-12 | 6.84E-10 | Cyp20a1 |
| 10514590 | 1.401071 | 7.375717 | -13.3349 | 4.15E-12 | 6.84E-10 | Dock7 |
| 10597531 | 1.423291 | 6.761078 | -13.3195 | 4.25E-12 | 6.92E-10 | Rbms3 |
| 10557177 | -1.34265 | 7.615034 | 13.31926 | 4.25E-12 | 6.92E-10 | Prkcb |
| 10569341 | 2.973242 | 7.641095 | -13.309 | 4.32E-12 | 6.99E-10 | H19 |
| 10474201 | -1.63533 | 8.673626 | 13.28858 | 4.45E-12 | 7.16E-10 | Lmo2 |
| 10409376 | -1.34044 | 6.182186 | 13.28218 | 4.49E-12 | 7.19E-10 | Hk3 |
| 10560282 | 1.100692 | 7.271693 | -13.263 | 4.62E-12 | 7.36E-10 | Arhgap35 |
| 10584435 | 1.241981 | 7.642908 | -13.2444 | 4.75E-12 | 7.50E-10 | Vwa5a |
| 10586357 | 2.482586 | 6.995048 | -13.2434 | 4.76E-12 | 7.50E-10 | Cilp |
| 10593198 | -3.4208 | 7.654929 | 13.23176 | 4.85E-12 | 7.55E-10 | Nxpe2 |
| 10439218 | 2.101549 | 6.47165 | -13.2254 | 4.89E-12 | 7.55E-10 | Pdia5 |
| 10556426 | 1.959317 | 7.653533 | -13.2244 | 4.90E-12 | 7.55E-10 | Parva |
| 10450038 | 2.246029 | 6.981554 | -13.2234 | 4.91E-12 | 7.55E-10 | Angptl4 |
| 10568328 | 1.682874 | 8.00795 | -13.2209 | 4.93E-12 | 7.55E-10 | Vkorc1 |
| 10443470 | -1.61017 | 6.997078 | 13.20589 | 5.04E-12 | 7.62E-10 | Rab44 |
| 10594103 | -1.07102 | 5.886577 | 13.20094 | 5.08E-12 | 7.62E-10 | Rec114 |
| 10565958 | 2.202795 | 7.353648 | -13.2004 | 5.08E-12 | 7.62E-10 | P2ry6 |
| 10419156 | -3.17817 | 8.06658 | 13.18037 | 5.24E-12 | 7.82E-10 | Ear2 |
| 10405587 | 1.783859 | 9.23123 | -13.1757 | 5.27E-12 | 7.83E-10 | Tgfbi |
| 10529656 | 1.954349 | 7.089171 | -13.1685 | 5.33E-12 | 7.84E-10 | Nsg1 |
| 10412078 | -1.45328 | 6.410613 | 13.1679 | 5.33E-12 | 7.84E-10 | Gapt |
| 10547282 | 1.756181 | 6.209403 | -13.152 | 5.46E-12 | 7.95E-10 | Zfp9 |
| 10472965 | 1.707086 | 5.897673 | -13.1443 | 5.53E-12 | 8.00E-10 | Hoxd8 |
| 10472440 | 1.53563 | 7.492049 | -13.137 | 5.59E-12 | 8.01E-10 | Tax1bp3 |
| 10471555 | 1.927322 | 7.709338 | -13.1227 | 5.71E-12 | 8.15E-10 | Angptl2 |
| 10358476 | 3.479101 | 8.66546 | -13.1145 | 5.78E-12 | 8.21E-10 | Prg4 |
| 10360806 | 1.179044 | 8.105557 | -13.1068 | 5.85E-12 | 8.23E-10 | Capn2 |
| 10378334 | 1.491802 | 7.485154 | -13.1066 | 5.85E-12 | 8.23E-10 | Tax1bp3 |
| 10517236 | 1.077446 | 6.203951 | -13.092 | 5.98E-12 | 8.37E-10 | Zfp593 |
| 10574023 | 2.367125 | 10.28921 | -13.0861 | 6.04E-12 | 8.40E-10 | Mt2 |
| 10549647 | -1.26382 | 5.112132 | 13.07947 | 6.10E-12 | 8.45E-10 | Ncr1 |
| 10396421 | 2.107964 | 8.71469 | -13.0673 | 6.21E-12 | 8.56E-10 | Hif1a |
| 10438060 | -2.08489 | 5.983021 | 13.05269 | 6.35E-12 | 8.68E-10 | Igll1 |
| 10560190 | 1.727513 | 6.907829 | -13.0523 | 6.36E-12 | 8.68E-10 | Ehd2 |
| 10423109 | 2.116965 | 5.979661 | -13.046 | 6.42E-12 | 8.72E-10 | Adamts12 |
| 10544186 | -2.1192 | 8.618151 | 13.02597 | 6.61E-12 | 8.95E-10 | Mkrn1 |
| 10507273 | 1.539201 | 6.120749 | -13.0171 | 6.70E-12 | 9.03E-10 | Pik3r3 |
| 10522503 | 1.979202 | 7.718275 | -13.0121 | 6.76E-12 | 9.05E-10 | Pdgfra |
| 10431637 | 1.499191 | 5.799314 | -13.0054 | 6.83E-12 | 9.08E-10 | Cpne8 |
| 10358816 | 1.546868 | 7.770828 | -13.0039 | 6.84E-12 | 9.08E-10 | Lamc1 |
| 10599174 | 2.153713 | 7.16472 | -12.9847 | 7.04E-12 | 9.27E-10 | Il13ra1 |
| 10428707 | 2.919882 | 6.275978 | -12.9748 | 7.15E-12 | 9.37E-10 | Has2 |
| 10435075 | -1.75421 | 9.501964 | 12.96503 | 7.26E-12 | 9.44E-10 | Tfrc |
| 10495830 | 2.059643 | 7.502359 | -12.9635 | 7.28E-12 | 9.44E-10 | Sec24d |
| 10371959 | 1.24873 | 7.895894 | -12.9529 | 7.39E-12 | 9.55E-10 | Elk3 |
| 10460544 | 1.23483 | 7.585884 | -12.9316 | 7.64E-12 | 9.83E-10 | Yif1a |
| 10546567 | 1.325481 | 6.655913 | -12.9244 | 7.72E-12 | 9.89E-10 | Eogt |
| 10461497 | 1.11814 | 9.046732 | -12.8985 | 8.04E-12 | 1.02E-09 | Ddb1 |
| 10452633 | 1.122719 | 7.496727 | -12.8865 | 8.19E-12 | 1.04E-09 | Tgif1 |
| 10458340 | 1.238086 | 6.009456 | -12.8763 | 8.31E-12 | 1.05E-09 | Hbegf |
| 10492864 | 1.241352 | 6.725339 | -12.8759 | 8.32E-12 | 1.05E-09 | Sh3d19 |
| 10502776 | 1.524587 | 7.022856 | -12.8642 | 8.47E-12 | 1.06E-09 | Lphn2 |
| 10605143 | -1.72294 | 6.960857 | 12.85887 | 8.54E-12 | 1.06E-09 | Arhgap4 |
| 10414262 | -3.31699 | 7.145656 | 12.85353 | 8.61E-12 | 1.06E-09 | Ear2 |
| 10499189 | 2.643541 | 5.685189 | -12.8394 | 8.80E-12 | 1.08E-09 | Fcrls |
| 10523120 | 3.678151 | 6.847301 | -12.8299 | 8.93E-12 | 1.09E-09 | Cxcl5 |
| 10381371 | 1.557301 | 5.710671 | -12.7975 | 9.39E-12 | 1.15E-09 | Aoc3 |
| 10597960 | -1.6603 | 6.33913 | 12.79117 | 9.48E-12 | 1.15E-09 | Slc6a20a |
| 10528008 | 1.671388 | 5.184284 | -12.7902 | 9.49E-12 | 1.15E-09 | Steap2 |
| 10444016 | -2.43846 | 7.752316 | 12.77734 | 9.68E-12 | 1.17E-09 | Pram1 |
| 10374248 | -2.22 | 6.534764 | 12.7702 | 9.79E-12 | 1.18E-09 | Abca13 |
| 10542791 | 1.667244 | 7.651066 | -12.7578 | 9.98E-12 | 1.19E-09 | Ppfibp1 |
| 10448307 | 1.470796 | 6.903515 | -12.7558 | 1.00E-11 | 1.19E-09 | Tnfrsf12a |
| 10598175 | -2.95349 | 7.835374 | 12.74551 | 1.02E-11 | 1.20E-09 | Ear10 |
| 10545255 | -1.66381 | 6.425609 | 12.74285 | 1.02E-11 | 1.20E-09 | Rpia |
| 10412562 | 1.464035 | 7.014797 | -12.739 | 1.03E-11 | 1.20E-09 | Flnb |
| 10478415 | 1.693884 | 7.26108 | -12.7316 | 1.04E-11 | 1.21E-09 | Wisp2 |
| 10514221 | 1.072326 | 7.880357 | -12.7164 | 1.06E-11 | 1.23E-09 | Plin2 |
| 10490159 | 2.020345 | 8.396797 | -12.6984 | 1.09E-11 | 1.26E-09 | Pmepa1 |
| 10445774 | -1.91612 | 8.047939 | 12.68415 | 1.12E-11 | 1.28E-09 | B430306N03Rik |
| 10381122 | 2.873869 | 7.477924 | -12.6775 | 1.13E-11 | 1.29E-09 | Fkbp10 |
| 10519497 | 1.420946 | 7.845842 | -12.6683 | 1.15E-11 | 1.30E-09 | Steap4 |
| 10477717 | 1.134003 | 5.843016 | -12.6561 | 1.17E-11 | 1.32E-09 | Procr |
| 10416689 | -2.88577 | 7.157151 | 12.64515 | 1.19E-11 | 1.33E-09 | Olfm4 |
| 10360227 | 1.167575 | 8.446207 | -12.6446 | 1.19E-11 | 1.33E-09 | Pea15a |
| 10605766 | 2.279643 | 8.49182 | -12.6243 | 1.23E-11 | 1.37E-09 | Maged1 |
| 10571142 | 1.518685 | 6.069828 | -12.616 | 1.24E-11 | 1.38E-09 | Gpr124 |
| 10494445 | 1.641552 | 6.937592 | -12.6093 | 1.26E-11 | 1.39E-09 | Lix1l |
| 10350516 | 2.944713 | 7.00111 | -12.6062 | 1.26E-11 | 1.39E-09 | Ptgs2 |
| 10490838 | 2.17704 | 8.244994 | -12.588 | 1.30E-11 | 1.43E-09 | Fabp5 |
| 10389339 | -1.43888 | 8.847435 | 12.57083 | 1.33E-11 | 1.46E-09 | Usp32 |
| 10360745 | -1.83271 | 8.349174 | 12.54137 | 1.40E-11 | 1.53E-09 | Lbr |
| 10534940 | -1.88717 | 6.711827 | 12.53703 | 1.41E-11 | 1.53E-09 | Pilrb2 |
| 10483381 | 1.44611 | 6.997088 | -12.5295 | 1.42E-11 | 1.54E-09 | Stk39 |
| 10471247 | 1.953396 | 6.075409 | -12.5274 | 1.43E-11 | 1.54E-09 | Aif1l |
| 10349166 | -2.90762 | 5.512 | 12.52608 | 1.43E-11 | 1.54E-09 | Serpinb10 |
| 10418171 | 1.370337 | 7.104416 | -12.5143 | 1.46E-11 | 1.56E-09 | Zcchc24 |
| 10425287 | 3.255789 | 6.811692 | -12.5089 | 1.47E-11 | 1.57E-09 | Kdelr3 |
| 10379535 | 4.574168 | 7.619346 | -12.5067 | 1.48E-11 | 1.57E-09 | Ccl8 |
| 10520452 | 3.365991 | 5.221189 | -12.5016 | 1.49E-11 | 1.58E-09 | Il6 |
| 10507137 | -2.58745 | 7.243837 | 12.49706 | 1.50E-11 | 1.58E-09 | Pdzk1ip1 |
| 10421456 | -1.91379 | 9.513663 | 12.49585 | 1.50E-11 | 1.58E-09 | Xpo7 |
| 10518147 | 2.869218 | 7.616161 | -12.4761 | 1.55E-11 | 1.62E-09 | Pdpn |
| 10500610 | -2.86856 | 8.827174 | 12.4704 | 1.56E-11 | 1.63E-09 | Fam46c |
| 10433096 | -2.70273 | 7.885752 | 12.4652 | 1.58E-11 | 1.63E-09 | Nfe2 |
| 10441178 | -2.47379 | 7.211385 | 12.46459 | 1.58E-11 | 1.63E-09 | Itgb2l |
| 10403727 | 1.532892 | 6.023056 | -12.4581 | 1.59E-11 | 1.65E-09 | Gli3 |
| 10393379 | 1.559863 | 6.610009 | -12.4192 | 1.69E-11 | 1.74E-09 | Mxra7 |
| 10349980 | 1.70195 | 6.96425 | -12.4085 | 1.72E-11 | 1.75E-09 | Mybph |
| 10501762 | 2.490973 | 7.199692 | -12.4079 | 1.72E-11 | 1.75E-09 | Snx7 |
| 10356520 | 2.678871 | 8.365032 | -12.4067 | 1.73E-11 | 1.75E-09 | Col6a3 |
| 10424140 | 2.420241 | 6.488063 | -12.403 | 1.74E-11 | 1.75E-09 | Col14a1 |
| 10356886 | -2.03273 | 5.637233 | 12.40161 | 1.74E-11 | 1.75E-09 | Slco4c1 |
| 10519140 | 2.327668 | 7.36595 | -12.4014 | 1.74E-11 | 1.75E-09 | Mmp23 |
| 10459421 | 1.56274 | 5.806122 | -12.3901 | 1.77E-11 | 1.77E-09 | Atp8b1 |
| 10428579 | 1.743542 | 7.865539 | -12.3893 | 1.78E-11 | 1.77E-09 | Ext1 |
| 10461878 | 1.689584 | 5.678311 | -12.3767 | 1.81E-11 | 1.80E-09 | Prune2 |
| 10375432 | -1.41357 | 4.970793 | 12.37497 | 1.82E-11 | 1.80E-09 | Fndc9 |
| 10585699 | 2.052638 | 8.27032 | -12.3708 | 1.83E-11 | 1.80E-09 | Fabp5 |
| 10349174 | 1.180905 | 5.419816 | -12.368 | 1.84E-11 | 1.81E-09 | Serpinb8 |
| 10484227 | 1.191609 | 6.15573 | -12.355 | 1.88E-11 | 1.84E-09 | Sestd1 |
| 10445753 | -2.51882 | 6.726652 | 12.35275 | 1.88E-11 | 1.84E-09 | Trem3 |
| 10474671 | 1.456754 | 7.595299 | -12.3473 | 1.90E-11 | 1.85E-09 | Spred1 |
| 10429564 | 1.635778 | 9.496314 | -12.3047 | 2.03E-11 | 1.96E-09 | Ly6a |
| 10393936 | 1.987119 | 6.906891 | -12.3032 | 2.04E-11 | 1.96E-09 | Cbr2 |
| 10481262 | -2.49024 | 7.536009 | 12.29886 | 2.05E-11 | 1.97E-09 | Fcnb |
| 10497548 | 1.366855 | 7.579898 | -12.2962 | 2.06E-11 | 1.97E-09 | Fndc3b |
| 10368199 | -2.73601 | 7.43842 | 12.29444 | 2.06E-11 | 1.97E-09 | Myb |
| 10385903 | 1.617658 | 7.001638 | -12.2833 | 2.10E-11 | 2.00E-09 | Pdlim4 |
| 10564343 | 1.233816 | 7.33996 | -12.2754 | 2.13E-11 | 2.02E-09 | Tjp1 |
| 10529875 | 1.235268 | 6.932098 | -12.2714 | 2.14E-11 | 2.02E-09 | Ldb2 |
| 10368647 | 1.805163 | 6.808927 | -12.2624 | 2.17E-11 | 2.05E-09 | Dse |
| 10407211 | 1.044164 | 7.872834 | -12.2436 | 2.24E-11 | 2.10E-09 | Ppap2a |
| 10542691 | -1.69367 | 7.073745 | 12.23344 | 2.28E-11 | 2.13E-09 | Lrmp |
| 10554752 | 2.13068 | 5.977164 | -12.2294 | 2.29E-11 | 2.14E-09 | Nox4 |
| 10552380 | -1.37995 | 5.771458 | 12.2216 | 2.32E-11 | 2.16E-09 | Siglecg |
| 10588049 | 1.046737 | 8.999781 | -12.1993 | 2.40E-11 | 2.22E-09 | Copb2 |
| 10361338 | -1.9408 | 5.962917 | 12.19885 | 2.40E-11 | 2.22E-09 | Ipcef1 |
| 10389654 | -2.34331 | 4.769168 | 12.19268 | 2.43E-11 | 2.24E-09 | Epx |
| 10358879 | 2.196024 | 6.721475 | -12.1832 | 2.47E-11 | 2.26E-09 | Npl |
| 10451670 | -2.81328 | 6.103361 | 12.16722 | 2.53E-11 | 2.30E-09 | Tspo2 |
| 10466200 | 2.573834 | 8.44906 | -12.1657 | 2.54E-11 | 2.30E-09 | Ms4a7 |
| 10547641 | -2.30647 | 7.387348 | 12.13071 | 2.68E-11 | 2.42E-09 | Slc2a3 |
| 10485645 | 2.017141 | 7.62093 | -12.1276 | 2.70E-11 | 2.43E-09 | Rcn1 |
| 10373340 | 1.034668 | 7.434751 | -12.1164 | 2.74E-11 | 2.46E-09 | Rbms2 |
| 10493709 | 1.231433 | 8.08098 | -12.1159 | 2.75E-11 | 2.46E-09 | Slc39a1 |
| 10442224 | 1.419419 | 6.766697 | -12.1067 | 2.79E-11 | 2.49E-09 | Zfp948 |
| 10425161 | 1.096204 | 11.06104 | -12.1005 | 2.82E-11 | 2.50E-09 | Lgals1 |
| 10534935 | -1.82905 | 7.722347 | 12.0974 | 2.83E-11 | 2.51E-09 | Pilrb1 |
| 10505172 | 1.25971 | 6.38292 | -12.0931 | 2.85E-11 | 2.52E-09 | Dnajc25 |
| 10419154 | -3.50232 | 7.964605 | 12.07157 | 2.95E-11 | 2.60E-09 | Ear1 |
| 10552760 | -1.14388 | 6.653792 | 12.05937 | 3.01E-11 | 2.63E-09 | Pnkp |
| 10491952 | -2.32701 | 7.87129 | 12.05104 | 3.05E-11 | 2.65E-09 | Mgst2 |
| 10478447 | -1.19116 | 8.099705 | 12.04288 | 3.09E-11 | 2.68E-09 | Stk4 |
| 10518408 | 1.679679 | 7.045672 | -12.0408 | 3.10E-11 | 2.68E-09 | Plod1 |
| 10539263 | 1.963952 | 6.851141 | -12.0301 | 3.15E-11 | 2.72E-09 | Loxl3 |
| 10532711 | 1.25435 | 6.307755 | -12.0264 | 3.17E-11 | 2.73E-09 | Cmklr1 |
| 10473444 | 1.995708 | 6.855772 | -12.0238 | 3.19E-11 | 2.73E-09 | Aplnr |
| 10477777 | 1.011601 | 9.174 | -12.0137 | 3.24E-11 | 2.77E-09 | Ergic3 |
| 10430145 | 1.385615 | 6.68803 | -12.002 | 3.30E-11 | 2.81E-09 | Rbfox2 |
| 10540241 | 1.146926 | 7.936007 | -11.9883 | 3.37E-11 | 2.85E-09 | Arl6ip5 |
| 10502071 | -1.31818 | 7.056321 | 11.98139 | 3.41E-11 | 2.88E-09 | 5730508B09Rik |
| 10435271 | 1.117171 | 8.096706 | -11.9775 | 3.43E-11 | 2.89E-09 | Heg1 |
| 10420035 | 1.167734 | 6.975104 | -11.9665 | 3.50E-11 | 2.93E-09 | Ipo4 |
| 10436372 | 1.539483 | 6.177051 | -11.9622 | 3.52E-11 | 2.94E-09 | Dcbld2 |
| 10403604 | -1.47066 | 8.181319 | 11.95569 | 3.56E-11 | 2.96E-09 | Lyst |
| 10391454 | 1.156468 | 7.581511 | -11.9509 | 3.59E-11 | 2.98E-09 | Vat1 |
| 10587023 | -1.96822 | 7.092657 | 11.94949 | 3.59E-11 | 2.98E-09 | Rab27a |
| 10387699 | -1.90252 | 6.540836 | 11.94392 | 3.63E-11 | 2.99E-09 | Acap1 |
| 10514177 | 1.939793 | 5.968035 | -11.938 | 3.66E-11 | 3.01E-09 | Bnc2 |
| 10563077 | 2.492031 | 8.387969 | -11.9343 | 3.68E-11 | 3.02E-09 | Rcn3 |
| 10416533 | 1.497187 | 6.249954 | -11.9293 | 3.71E-11 | 3.04E-09 | Ccdc122 |
| 10584208 | 1.265025 | 6.216682 | -11.9157 | 3.80E-11 | 3.08E-09 | Cdon |
| 10556381 | 1.153864 | 6.528643 | -11.9154 | 3.80E-11 | 3.08E-09 | Mical2 |
| 10371230 | 1.118306 | 7.619689 | -11.907 | 3.85E-11 | 3.12E-09 | Gna11 |
| 10563715 | -2.42188 | 7.401068 | 11.87426 | 4.06E-11 | 3.27E-09 | Mrgpra2a |
| 10377927 | -1.2994 | 8.024787 | 11.85909 | 4.16E-11 | 3.33E-09 | Rnf167 |
| 10399710 | -2.23244 | 8.038868 | 11.84607 | 4.25E-11 | 3.40E-09 | Rsad2 |
| 10583870 | 1.453134 | 6.375851 | -11.8362 | 4.32E-11 | 3.44E-09 | Bmper |
| 10351905 | -3.43467 | 7.359457 | 11.83126 | 4.36E-11 | 3.46E-09 | Spta1 |
| 10429580 | -3.35034 | 8.398545 | 11.81705 | 4.46E-11 | 3.51E-09 | I830127L07Rik |
| 10434441 | 1.066877 | 5.558937 | -11.8164 | 4.47E-11 | 3.51E-09 | Ece2 |
| 10566454 | 1.815019 | 7.950886 | -11.8128 | 4.49E-11 | 3.51E-09 | Prkcdbp |
| 10584841 | -1.15313 | 5.84173 | 11.81091 | 4.51E-11 | 3.51E-09 | Amica1 |
| 10593219 | 1.907219 | 7.04175 | -11.8106 | 4.51E-11 | 3.51E-09 | Nnmt |
| 10527936 | 1.297109 | 6.622618 | -11.8069 | 4.54E-11 | 3.51E-09 | Fzd1 |
| 10473356 | -2.31201 | 8.82607 | 11.79675 | 4.61E-11 | 3.56E-09 | Ube2l6 |
| 10493692 | 1.000272 | 6.534748 | -11.7865 | 4.69E-11 | 3.61E-09 | Rab13 |
| 10366951 | 2.179261 | 6.450994 | -11.7797 | 4.74E-11 | 3.64E-09 | Ndufa4l2 |
| 10436392 | -2.20466 | 8.33611 | 11.77506 | 4.78E-11 | 3.66E-09 | Cpox |
| 10362896 | -1.45243 | 11.2889 | 11.77452 | 4.78E-11 | 3.66E-09 | Cd24a |
| 10568202 | -1.74745 | 7.072045 | 11.76893 | 4.83E-11 | 3.68E-09 | 44440 |
| 10462922 | 1.340191 | 5.946847 | -11.7621 | 4.88E-11 | 3.71E-09 | Plce1 |
| 10602372 | -3.10477 | 9.052948 | 11.74671 | 5.01E-11 | 3.79E-09 | Alas2 |
| 10563712 | -2.51779 | 7.629655 | 11.73864 | 5.07E-11 | 3.83E-09 | Mrgpra2a |
| 10484402 | 1.026624 | 7.629863 | -11.7291 | 5.16E-11 | 3.87E-09 | Ctnnd1 |
| 10423971 | -2.50056 | 6.402626 | 11.72665 | 5.18E-11 | 3.87E-09 | Pkhd1l1 |
| 10590844 | 1.877622 | 5.87561 | -11.7254 | 5.19E-11 | 3.87E-09 | Arhgap42 |
| 10434782 | 1.32494 | 7.045225 | -11.7244 | 5.20E-11 | 3.87E-09 | Lpp |
| 10467191 | 2.930589 | 6.678645 | -11.7122 | 5.30E-11 | 3.94E-09 | Ankrd1 |
| 10488655 | -1.3671 | 8.224688 | 11.70166 | 5.39E-11 | 4.00E-09 | Bcl2l1 |
| 10561008 | -2.1308 | 7.764826 | 11.69926 | 5.41E-11 | 4.00E-09 | Ceacam1 |
| 10430968 | 1.432784 | 5.604462 | -11.6933 | 5.47E-11 | 4.03E-09 | A4galt |
| 10540298 | 2.411553 | 5.352017 | -11.6875 | 5.52E-11 | 4.05E-09 | Chl1 |
| 10545086 | -2.85391 | 8.587638 | 11.67716 | 5.62E-11 | 4.11E-09 | Snca |
| 10567564 | -1.60953 | 8.33366 | 11.66956 | 5.69E-11 | 4.15E-09 | Cdr2 |
| 10534862 | 1.921892 | 10.00441 | -11.6648 | 5.73E-11 | 4.17E-09 | Pcolce |
| 10502156 | 1.543665 | 5.99518 | -11.6383 | 5.99E-11 | 4.34E-09 | Ccdc109b |
| 10591517 | -1.37166 | 7.376248 | 11.63544 | 6.02E-11 | 4.34E-09 | Cdkn2d |
| 10392834 | 2.323101 | 8.17073 | -11.6346 | 6.03E-11 | 4.34E-09 | Gm11710 |
| 10468309 | 1.474787 | 8.205645 | -11.6268 | 6.10E-11 | 4.38E-09 | Sh3pxd2a |
| 10498998 | -1.56512 | 8.038038 | 11.62617 | 6.11E-11 | 4.38E-09 | D930015E06Rik |
| 10442762 | -2.58868 | 7.048379 | 11.57255 | 6.68E-11 | 4.75E-09 | Prss34 |
| 10419151 | -4.34138 | 7.319784 | 11.56164 | 6.80E-11 | 4.82E-09 | Ear1 |
| 10363512 | 1.026879 | 9.843524 | -11.5582 | 6.84E-11 | 4.83E-09 | Sar1a |
| 10394534 | 1.80579 | 6.277265 | -11.5507 | 6.93E-11 | 4.88E-09 | Osr1 |
| 10555323 | 2.221185 | 5.812209 | -11.5414 | 7.04E-11 | 4.94E-09 | P4ha3 |
| 10509002 | -3.75579 | 7.85825 | 11.53997 | 7.05E-11 | 4.94E-09 | Rhd |
| 10517609 | 1.602342 | 5.663281 | -11.5394 | 7.06E-11 | 4.94E-09 | Cda |
| 10505894 | 1.153419 | 5.682332 | -11.5327 | 7.14E-11 | 4.97E-09 | Mtap |
| 10381298 | 1.358122 | 6.361842 | -11.5293 | 7.18E-11 | 4.98E-09 | Ramp2 |
| 10586865 | 1.602567 | 6.178662 | -11.5288 | 7.18E-11 | 4.98E-09 | Aldh1a2 |
| 10605055 | 1.172475 | 5.17533 | -11.5205 | 7.29E-11 | 5.03E-09 | Haus7 |
| 10576090 | -1.60654 | 7.085171 | 11.5124 | 7.38E-11 | 5.08E-09 | Zfpm1 |
| 10445789 | -2.36029 | 7.440452 | 11.51169 | 7.39E-11 | 5.08E-09 | Treml1 |
| 10607752 | -1.32355 | 5.050745 | 11.50228 | 7.51E-11 | 5.15E-09 | Bmx |
| 10512757 | -3.32656 | 7.916454 | 11.50103 | 7.53E-11 | 5.15E-09 | Hemgn |
| 10413304 | 1.434661 | 9.330399 | -11.4949 | 7.60E-11 | 5.19E-09 | Arf4 |
| 10450675 | -2.05654 | 6.566444 | 11.49295 | 7.63E-11 | 5.19E-09 | H2-T24 |
| 10349968 | -2.33132 | 7.39981 | 11.49278 | 7.63E-11 | 5.19E-09 | Chil1 |
| 10426689 | 1.675737 | 6.159572 | -11.486 | 7.72E-11 | 5.22E-09 | Spats2 |
| 10392825 | 2.318338 | 8.330723 | -11.4856 | 7.72E-11 | 5.22E-09 | Gm11710 |
| 10424905 | 1.612096 | 6.744191 | -11.4695 | 7.93E-11 | 5.34E-09 | Scx |
| 10459496 | 1.340893 | 5.96854 | -11.4661 | 7.98E-11 | 5.35E-09 | Ccbe1 |
| 10560886 | -2.63123 | 8.123808 | 11.46582 | 7.98E-11 | 5.35E-09 | Cd177 |
| 10505276 | 1.248415 | 7.539875 | -11.4512 | 8.18E-11 | 5.46E-09 | Slc31a1 |
| 10588263 | 1.710624 | 6.741016 | -11.4453 | 8.26E-11 | 5.50E-09 | Slco2a1 |
| 10375880 | 1.036188 | 8.770569 | -11.4432 | 8.29E-11 | 5.50E-09 | Nhp2 |
| 10453057 | 1.516193 | 8.378526 | -11.4322 | 8.45E-11 | 5.59E-09 | Cyp1b1 |
| 10525989 | 1.705984 | 6.50301 | -11.4162 | 8.68E-11 | 5.73E-09 | Gpr133 |
| 10586744 | 1.108573 | 9.218776 | -11.4117 | 8.74E-11 | 5.76E-09 | Anxa2 |
| 10520553 | 1.042036 | 8.000208 | -11.4111 | 8.75E-11 | 5.76E-09 | Tmem214 |
| 10607143 | 2.838637 | 5.783545 | -11.4031 | 8.87E-11 | 5.82E-09 | Capn6 |
| 10544383 | -3.74111 | 7.409565 | 11.39595 | 8.98E-11 | 5.87E-09 | Kel |
| 10380699 | 1.908293 | 8.150179 | -11.3952 | 8.99E-11 | 5.87E-09 | Copz2 |
| 10551852 | 1.427985 | 6.688788 | -11.392 | 9.04E-11 | 5.89E-09 | Clip3 |
| 10541605 | 2.718604 | 6.269362 | -11.3803 | 9.22E-11 | 5.99E-09 | Clec4n |
| 10362201 | 1.547952 | 8.598332 | -11.3677 | 9.41E-11 | 6.10E-09 | Ctgf |
| 10370180 | 2.041084 | 7.37055 | -11.3676 | 9.41E-11 | 6.10E-09 | Col6a2 |
| 10379685 | -1.1664 | 6.02917 | 11.36536 | 9.45E-11 | 6.11E-09 | 1700020L24Rik |
| 10592816 | -2.45208 | 8.798419 | 11.35664 | 9.59E-11 | 6.18E-09 | Hmbs |
| 10530841 | 1.347761 | 9.71608 | -11.3551 | 9.62E-11 | 6.18E-09 | Igfbp7 |
| 10363498 | 1.040694 | 8.152686 | -11.3445 | 9.79E-11 | 6.27E-09 | Ppa1 |
| 10357590 | -1.89152 | 6.659596 | 11.32327 | 1.01E-10 | 6.45E-09 | Dyrk3 |
| 10344897 | 2.280608 | 7.078447 | -11.2951 | 1.06E-10 | 6.74E-09 | Sulf1 |
| 10600852 | -2.02023 | 9.265708 | 11.29227 | 1.07E-10 | 6.76E-09 | F630028O10Rik |
| 10496359 | 1.487962 | 7.200274 | -11.2875 | 1.08E-10 | 6.80E-09 | Emcn |
| 10389087 | -1.0904 | 6.938994 | 11.28179 | 1.09E-10 | 6.85E-09 | Rffl |
| 10559385 | 1.400794 | 5.464865 | -11.2804 | 1.09E-10 | 6.85E-09 | Mrgprf |
| 10368670 | -1.2038 | 8.508324 | 11.27861 | 1.09E-10 | 6.85E-09 | Amd2 |
| 10406905 | -1.59589 | 6.515848 | 11.2768 | 1.10E-10 | 6.85E-09 | Ccdc125 |
| 10568668 | 2.444675 | 6.576119 | -11.2763 | 1.10E-10 | 6.85E-09 | Adam12 |
| 10473125 | -1.63575 | 8.323081 | 11.27281 | 1.11E-10 | 6.88E-09 | Itga4 |
| 10459866 | -3.30618 | 7.840297 | 11.26785 | 1.11E-10 | 6.92E-09 | Slc14a1 |
| 10498647 | 1.621524 | 6.521268 | -11.2636 | 1.12E-10 | 6.96E-09 | B3galnt1 |
| 10539080 | -1.66429 | 8.691372 | 11.24744 | 1.15E-10 | 7.12E-09 | St3gal5 |
| 10603289 | 1.432745 | 6.287631 | -11.2465 | 1.16E-10 | 7.12E-09 | Clcn5 |
| 10462343 | -1.37403 | 6.165709 | 11.24473 | 1.16E-10 | 7.12E-09 | Gm9895 |
| 10349648 | -3.78069 | 9.231388 | 11.24468 | 1.16E-10 | 7.12E-09 | Ctse |
| 10389300 | -2.62473 | 7.540186 | 11.23639 | 1.18E-10 | 7.21E-09 | Dhrs11 |
| 10364251 | 1.474711 | 7.049063 | -11.2346 | 1.18E-10 | 7.21E-09 | Pofut2 |
| 10406817 | 1.003904 | 6.52173 | -11.2314 | 1.19E-10 | 7.24E-09 | Enc1 |
| 10445046 | -3.33095 | 7.308222 | 11.22306 | 1.20E-10 | 7.31E-09 | Trim10 |
| 10440534 | 1.275099 | 7.475336 | -11.2205 | 1.21E-10 | 7.32E-09 | Adamts5 |
| 10490923 | -3.72685 | 9.466801 | 11.2198 | 1.21E-10 | 7.32E-09 | Car2 |
| 10601328 | 1.001261 | 6.518166 | -11.2129 | 1.22E-10 | 7.39E-09 | Uprt |
| 10351197 | -2.05305 | 8.31164 | 11.20843 | 1.23E-10 | 7.43E-09 | Sell |
| 10424683 | -3.8344 | 8.628646 | 11.20612 | 1.24E-10 | 7.45E-09 | Ly6g |
| 10365716 | 1.80872 | 7.315301 | -11.2023 | 1.25E-10 | 7.48E-09 | Ikbip |
| 10477012 | 1.03475 | 8.625775 | -11.1952 | 1.26E-10 | 7.54E-09 | Fkbp1a |
| 10512935 | -1.19279 | 8.265437 | 11.19439 | 1.26E-10 | 7.54E-09 | Amd2 |
| 10376074 | 1.792192 | 6.240785 | -11.187 | 1.28E-10 | 7.61E-09 | P4ha2 |
| 10446334 | -1.1815 | 7.191101 | 11.17656 | 1.30E-10 | 7.70E-09 | Glcci1 |
| 10489246 | 1.289804 | 7.2406 | -11.1743 | 1.31E-10 | 7.71E-09 | Mafb |
| 10375055 | -2.57071 | 7.354979 | 11.17373 | 1.31E-10 | 7.71E-09 | Hbq1b |
| 10485982 | 3.036953 | 7.749284 | -11.1707 | 1.31E-10 | 7.73E-09 | Actc1 |
| 10408616 | -1.35633 | 6.375964 | 11.15665 | 1.35E-10 | 7.87E-09 | Slc22a23 |
| 10573457 | -2.38807 | 6.661325 | 11.14932 | 1.36E-10 | 7.95E-09 | Klf1 |
| 10345230 | 1.027187 | 6.542929 | -11.1478 | 1.37E-10 | 7.96E-09 | Rab23 |
| 10536494 | 1.65735 | 7.134591 | -11.1358 | 1.40E-10 | 8.11E-09 | Cav2 |
| 10552037 | 1.568453 | 6.664882 | -11.129 | 1.41E-10 | 8.18E-09 | Sbsn |
| 10423080 | 3.657687 | 6.66234 | -11.1126 | 1.45E-10 | 8.40E-09 | C1qtnf3 |
| 10516490 | 1.986731 | 5.296074 | -11.1062 | 1.47E-10 | 8.46E-09 | Gjb5 |
| 10406334 | -1.43057 | 6.882385 | 11.09608 | 1.49E-10 | 8.59E-09 | Mctp1 |
| 10578690 | -1.67005 | 7.147533 | 11.09247 | 1.50E-10 | 8.63E-09 | Neil3 |
| 10396476 | 1.136962 | 7.439133 | -11.085 | 1.52E-10 | 8.72E-09 | Rhoj |
| 10515848 | -3.02097 | 8.02346 | 11.08356 | 1.53E-10 | 8.73E-09 | Ermap |
| 10473399 | -3.47447 | 8.625726 | 11.07877 | 1.54E-10 | 8.78E-09 | Prg2 |
| 10401527 | 1.591917 | 6.754501 | -11.0739 | 1.55E-10 | 8.84E-09 | Ltbp2 |
| 10466530 | 1.444018 | 6.232165 | -11.065 | 1.58E-10 | 8.94E-09 | Pcsk5 |
| 10498284 | 1.314013 | 7.493333 | -11.0599 | 1.59E-10 | 9.00E-09 | Wwtr1 |
| 10510700 | 1.667118 | 5.55797 | -11.0388 | 1.65E-10 | 9.29E-09 | Gpr153 |
| 10591563 | 1.058393 | 6.393606 | -11.0171 | 1.71E-10 | 9.63E-09 | Kank2 |
| 10501007 | -1.50357 | 4.730444 | 10.99909 | 1.76E-10 | 9.88E-09 | Chil5 |
| 10369252 | 1.473623 | 7.088305 | -10.998 | 1.77E-10 | 9.88E-09 | 44449 |
| 10500335 | 2.025353 | 6.736729 | -10.9969 | 1.77E-10 | 9.88E-09 | Fcgr1 |
| 10493798 | 1.381378 | 7.760323 | -10.9945 | 1.78E-10 | 9.88E-09 | S100a16 |
| 10510391 | 1.290056 | 8.424428 | -10.9944 | 1.78E-10 | 9.88E-09 | Srm |
| 10467650 | -1.0115 | 6.081896 | 10.99366 | 1.78E-10 | 9.88E-09 | Frat2 |
| 10430929 | -1.16435 | 6.93262 | 10.99293 | 1.78E-10 | 9.88E-09 | Tbrg3 |
| 10415052 | 2.126309 | 8.511161 | -10.9906 | 1.79E-10 | 9.90E-09 | Mmp14 |
| 10517791 | -2.07027 | 5.707324 | 10.9877 | 1.80E-10 | 9.94E-09 | Padi4 |
| 10486664 | -3.41408 | 7.510192 | 10.98643 | 1.80E-10 | 9.94E-09 | Epb4.2 |
| 10397416 | 1.083017 | 7.107889 | -10.9787 | 1.83E-10 | 1.00E-08 | Ift43 |
| 10487208 | -2.01808 | 7.714504 | 10.97816 | 1.83E-10 | 1.00E-08 | Atp8b4 |
| 10429128 | -1.72099 | 7.911845 | 10.97138 | 1.85E-10 | 1.01E-08 | Sla |
| 10391119 | 2.012159 | 6.059444 | -10.9706 | 1.85E-10 | 1.01E-08 | Leprel4 |
| 10473281 | 1.348116 | 8.04133 | -10.9639 | 1.88E-10 | 1.02E-08 | Itgav |
| 10350149 | 2.481908 | 6.443254 | -10.9572 | 1.90E-10 | 1.03E-08 | Tnni1 |
| 10534389 | -3.78526 | 7.607359 | 10.94882 | 1.93E-10 | 1.04E-08 | Cldn13 |
| 10423599 | 2.221729 | 6.724521 | -10.9462 | 1.93E-10 | 1.05E-08 | Matn2 |
| 10436456 | 1.419995 | 8.014183 | -10.9445 | 1.94E-10 | 1.05E-08 | Pros1 |
| 10451142 | 1.460706 | 6.022084 | -10.9402 | 1.95E-10 | 1.05E-08 | Gm7325 |
| 10385391 | -2.22773 | 7.560772 | 10.93174 | 1.98E-10 | 1.07E-08 | Cyfip2 |
| 10488195 | 1.324814 | 8.996719 | -10.9301 | 1.99E-10 | 1.07E-08 | Rrbp1 |
| 10374333 | -1.76006 | 7.700287 | 10.923 | 2.01E-10 | 1.08E-08 | Ikzf1 |
| 10509441 | 1.184351 | 6.98103 | -10.9165 | 2.04E-10 | 1.09E-08 | Ece1 |
| 10360028 | 1.327657 | 8.099035 | -10.9163 | 2.04E-10 | 1.09E-08 | Fcgr2b |
| 10375065 | 1.767355 | 6.431509 | -10.9128 | 2.05E-10 | 1.09E-08 | Sh3pxd2b |
| 10561004 | 1.225011 | 7.22909 | -10.9116 | 2.05E-10 | 1.09E-08 | Erf |
| 10404783 | 1.033054 | 4.821787 | -10.9086 | 2.06E-10 | 1.09E-08 | Edn1 |
| 10459389 | -1.23289 | 8.306463 | 10.90799 | 2.07E-10 | 1.09E-08 | Amd2 |
| 10369844 | 1.379246 | 7.926579 | -10.8994 | 2.10E-10 | 1.11E-08 | Bicc1 |
| 10376396 | -1.38812 | 5.841104 | 10.88452 | 2.15E-10 | 1.13E-08 | Trim58 |
| 10354897 | -1.5822 | 8.678454 | 10.87709 | 2.18E-10 | 1.14E-08 | Trak2 |
| 10451641 | -1.94599 | 5.269112 | 10.87522 | 2.19E-10 | 1.15E-08 | 9830107B12Rik |
| 10368748 | -1.18054 | 8.465742 | 10.87389 | 2.19E-10 | 1.15E-08 | Amd2 |
| 10542872 | 1.655469 | 7.274787 | -10.8657 | 2.22E-10 | 1.16E-08 | Rps4l |
| 10562117 | -1.26194 | 5.922803 | 10.84256 | 2.32E-10 | 1.20E-08 | Ffar2 |
| 10379044 | 1.414022 | 6.285124 | -10.836 | 2.34E-10 | 1.21E-08 | Rab34 |
| 10583326 | 1.245044 | 5.919648 | -10.8273 | 2.38E-10 | 1.22E-08 | Slc36a4 |
| 10596166 | -1.44596 | 6.118464 | 10.82712 | 2.38E-10 | 1.22E-08 | 1300017J02Rik |
| 10387890 | 1.913897 | 7.04763 | -10.8257 | 2.38E-10 | 1.22E-08 | Cxcl16 |
| 10529824 | -1.29886 | 5.340854 | 10.81775 | 2.42E-10 | 1.24E-08 | Prom1 |
| 10376455 | -1.11252 | 8.41203 | 10.81457 | 2.43E-10 | 1.24E-08 | Hist3h2a |
| 10564539 | -1.66498 | 5.755955 | 10.81067 | 2.45E-10 | 1.25E-08 | Mctp2 |
| 10423293 | 1.089962 | 7.724325 | -10.8103 | 2.45E-10 | 1.25E-08 | Myo10 |
| 10476301 | -1.26096 | 7.183163 | 10.79733 | 2.51E-10 | 1.27E-08 | Smox |
| 10460371 | -1.313 | 6.90049 | 10.79666 | 2.51E-10 | 1.27E-08 | Ptprcap |
| 10426315 | -1.15526 | 7.1936 | 10.79464 | 2.52E-10 | 1.27E-08 | Lrrk2 |
| 10440019 | 2.77984 | 6.667263 | -10.7944 | 2.52E-10 | 1.27E-08 | Tmem45a |
| 10431802 | 1.184867 | 8.602742 | -10.7941 | 2.52E-10 | 1.27E-08 | Twf1 |
| 10413726 | 2.860378 | 7.459049 | -10.7902 | 2.54E-10 | 1.28E-08 | Tnnc1 |
| 10542172 | -3.05783 | 6.786194 | 10.7899 | 2.54E-10 | 1.28E-08 | Clec1b |
| 10458028 | -1.16472 | 7.659084 | 10.78762 | 2.55E-10 | 1.28E-08 | Gypc |
| 10445192 | -4.10206 | 7.546259 | 10.78267 | 2.57E-10 | 1.29E-08 | Rhag |
| 10399908 | -2.1057 | 7.99883 | 10.77916 | 2.59E-10 | 1.29E-08 | Prkar2b |
| 10361381 | -1.58275 | 7.736593 | 10.7763 | 2.60E-10 | 1.29E-08 | Syne1 |
| 10495659 | 1.880644 | 7.380785 | -10.7744 | 2.61E-10 | 1.30E-08 | Cnn3 |
| 10459481 | 1.418388 | 8.088168 | -10.7656 | 2.65E-10 | 1.31E-08 | Lman1 |
| 10538547 | 2.117359 | 7.687085 | -10.758 | 2.68E-10 | 1.32E-08 | Fkbp9 |
| 10359624 | 1.408673 | 8.517184 | -10.7483 | 2.73E-10 | 1.34E-08 | Prrx1 |
| 10568363 | 1.474752 | 7.261687 | -10.7293 | 2.82E-10 | 1.39E-08 | Armcx3 |
| 10351551 | 1.441336 | 5.492135 | -10.727 | 2.83E-10 | 1.39E-08 | Adamts4 |
| 10415392 | -1.6243 | 7.038712 | 10.72157 | 2.86E-10 | 1.40E-08 | Ltb4r1 |
| 10561854 | 1.03198 | 8.550436 | -10.7142 | 2.90E-10 | 1.41E-08 | Tbcb |
| 10606714 | 1.168795 | 6.963062 | -10.7141 | 2.90E-10 | 1.41E-08 | Gla |
| 10456018 | 2.245352 | 5.669086 | -10.7127 | 2.91E-10 | 1.41E-08 | Arsi |
| 10493812 | 1.512195 | 10.78715 | -10.7031 | 2.96E-10 | 1.43E-08 | S100a4 |
| 10365559 | 1.546085 | 8.524883 | -10.7003 | 2.97E-10 | 1.44E-08 | Igf1 |
| 10574027 | 1.418531 | 10.28736 | -10.6942 | 3.00E-10 | 1.45E-08 | Mt1 |
| 10423836 | 3.816545 | 8.144061 | -10.6929 | 3.01E-10 | 1.45E-08 | Cthrc1 |
| 10500666 | 1.770662 | 6.3596 | -10.692 | 3.01E-10 | 1.45E-08 | Ptgfrn |
| 10349383 | 1.053428 | 7.11306 | -10.691 | 3.02E-10 | 1.45E-08 | Slc35f5 |
| 10467136 | 1.070419 | 4.347196 | -10.6865 | 3.04E-10 | 1.46E-08 | Ch25h |
| 10568714 | -1.69185 | 9.784767 | 10.67584 | 3.10E-10 | 1.48E-08 | Mki67 |
| 10378754 | 1.489781 | 6.311007 | -10.6748 | 3.11E-10 | 1.48E-08 | Fam57a |
| 10606609 | 2.402035 | 6.561512 | -10.6704 | 3.13E-10 | 1.49E-08 | Tspan6 |
| 10401852 | -1.24922 | 7.445842 | 10.66907 | 3.14E-10 | 1.49E-08 | Cep128 |
| 10438907 | -3.18517 | 7.401374 | 10.66362 | 3.17E-10 | 1.50E-08 | Gp5 |
| 10360377 | 1.643098 | 8.475972 | -10.6627 | 3.17E-10 | 1.50E-08 | AI607873 |
| 10593449 | 1.356476 | 6.60456 | -10.6612 | 3.18E-10 | 1.51E-08 | Layn |
| 10603417 | -2.2221 | 6.44334 | 10.65617 | 3.21E-10 | 1.52E-08 | Gata1 |
| 10592535 | -1.46947 | 7.731443 | 10.64661 | 3.26E-10 | 1.54E-08 | Sorl1 |
| 10467739 | 1.433715 | 6.816898 | -10.6463 | 3.27E-10 | 1.54E-08 | Avpi1 |
| 10354374 | -1.78138 | 7.515758 | 10.63703 | 3.32E-10 | 1.55E-08 | Slc40a1 |
| 10527940 | 1.361278 | 6.291053 | -10.6367 | 3.32E-10 | 1.55E-08 | Cdk14 |
| 10471929 | -1.54714 | 8.466492 | 10.63347 | 3.34E-10 | 1.56E-08 | Arhgap15 |
| 10374777 | 1.987096 | 7.418356 | -10.6197 | 3.42E-10 | 1.59E-08 | Efemp1 |
| 10425410 | -2.061 | 6.585054 | 10.61888 | 3.43E-10 | 1.59E-08 | Grap2 |
| 10536794 | 1.191699 | 5.971026 | -10.6187 | 3.43E-10 | 1.59E-08 | Hilpda |
| 10582020 | 1.052089 | 7.230464 | -10.6177 | 3.44E-10 | 1.59E-08 | Gcsh |
| 10376434 | -2.8155 | 7.329545 | 10.61427 | 3.46E-10 | 1.60E-08 | Btnl10 |
| 10601778 | 1.286965 | 6.887012 | -10.6131 | 3.46E-10 | 1.60E-08 | Armcx3 |
| 10467637 | -1.49727 | 7.370856 | 10.60258 | 3.53E-10 | 1.63E-08 | Arhgap19 |
| 10557326 | 1.636759 | 7.973311 | -10.598 | 3.56E-10 | 1.64E-08 | Il4ra |
| 10470027 | 1.171203 | 6.609263 | -10.5949 | 3.58E-10 | 1.64E-08 | Npdc1 |
| 10606858 | 1.154824 | 9.400301 | -10.5934 | 3.59E-10 | 1.65E-08 | Tceal8 |
| 10430358 | 2.386583 | 6.826289 | -10.5874 | 3.62E-10 | 1.66E-08 | C1qtnf6 |
| 10595126 | -1.15795 | 7.650728 | 10.58634 | 3.63E-10 | 1.66E-08 | Fbxo9 |
| 10574276 | -1.68155 | 6.730965 | 10.57636 | 3.70E-10 | 1.69E-08 | Gpr97 |
| 10462507 | 1.450321 | 7.220872 | -10.5713 | 3.73E-10 | 1.70E-08 | Papss2 |
| 10464647 | -1.27212 | 6.03842 | 10.56598 | 3.77E-10 | 1.71E-08 | Tbc1d10c |
| 10381603 | 1.451875 | 6.284917 | -10.565 | 3.77E-10 | 1.71E-08 | Fzd2 |
| 10471067 | 1.704205 | 6.974341 | -10.5612 | 3.80E-10 | 1.72E-08 | Prrx2 |
| 10468980 | -1.45797 | 7.216177 | 10.55947 | 3.81E-10 | 1.72E-08 | Fam107b |
| 10603551 | -1.80421 | 9.514607 | 10.54744 | 3.89E-10 | 1.76E-08 | Cybb |
| 10381072 | -1.68453 | 6.16455 | 10.54147 | 3.93E-10 | 1.77E-08 | Cdc6 |
| 10361215 | -1.52173 | 6.000672 | 10.53715 | 3.96E-10 | 1.78E-08 | Traf3ip3 |
| 10431424 | 1.436298 | 7.26722 | -10.5275 | 4.03E-10 | 1.81E-08 | Plxnb2 |
| 10566350 | -1.5153 | 6.306067 | 10.52384 | 4.06E-10 | 1.82E-08 | Trim30b |
| 10509280 | 1.672465 | 7.651846 | -10.5231 | 4.06E-10 | 1.82E-08 | Hspg2 |
| 10553559 | -1.64543 | 5.898131 | 10.51911 | 4.09E-10 | 1.83E-08 | Siglech |
| 10562812 | -2.63471 | 6.23951 | 10.51817 | 4.10E-10 | 1.83E-08 | Spib |
| 10588007 | -1.65397 | 8.657809 | 10.50487 | 4.20E-10 | 1.86E-08 | Tfdp2 |
| 10579012 | 1.708557 | 6.003471 | -10.5047 | 4.20E-10 | 1.86E-08 | Csgalnact1 |
| 10569646 | 1.425571 | 7.965188 | -10.4995 | 4.24E-10 | 1.88E-08 | Ccnd1 |
| 10569719 | -1.2448 | 6.344142 | 10.49858 | 4.24E-10 | 1.88E-08 | A430078G23Rik |
| 10348739 | 1.105555 | 6.551425 | -10.4851 | 4.35E-10 | 1.91E-08 | Sned1 |
| 10354677 | -1.03092 | 7.042197 | 10.48263 | 4.37E-10 | 1.92E-08 | Ankrd44 |
| 10408613 | 2.015438 | 6.701387 | -10.4695 | 4.47E-10 | 1.96E-08 | Tubb2b |
| 10365420 | 1.279645 | 8.073288 | -10.4454 | 4.67E-10 | 2.03E-08 | Tmem263 |
| 10461423 | 1.511471 | 7.207154 | -10.4323 | 4.78E-10 | 2.07E-08 | Fads3 |
| 10575209 | -1.00644 | 5.83685 | 10.43097 | 4.79E-10 | 2.07E-08 | A430107J10Rik |
| 10350335 | -1.23692 | 8.162456 | 10.43063 | 4.79E-10 | 2.07E-08 | Hmbs |
| 10516823 | -2.31896 | 9.030744 | 10.43042 | 4.79E-10 | 2.07E-08 | Epb4.1 |
| 10569707 | 1.112477 | 8.995582 | -10.4243 | 4.85E-10 | 2.09E-08 | Myadm |
| 10455752 | 1.075763 | 6.298409 | -10.42 | 4.88E-10 | 2.10E-08 | Snx24 |
| 10487645 | 1.807023 | 6.516038 | -10.4187 | 4.90E-10 | 2.10E-08 | Cpxm1 |
| 10417759 | 1.321568 | 6.330649 | -10.4185 | 4.90E-10 | 2.10E-08 | Ube2e2 |
| 10573054 | -4.14661 | 8.907332 | 10.41062 | 4.97E-10 | 2.13E-08 | Gypa |
| 10347748 | 1.038279 | 6.008752 | -10.4064 | 5.00E-10 | 2.14E-08 | Utp14b |
| 10429341 | 1.102065 | 6.481511 | -10.4022 | 5.04E-10 | 2.15E-08 | Ptk2 |
| 10590635 | 1.967896 | 7.061573 | -10.4018 | 5.05E-10 | 2.15E-08 | Ccr5 |
| 10598013 | 1.967896 | 7.061573 | -10.4018 | 5.05E-10 | 2.15E-08 | Ccr5 |
| 10519060 | -1.72734 | 8.188379 | 10.39053 | 5.15E-10 | 2.18E-08 | Tnfrsf14 |
| 10400483 | -1.84292 | 5.408165 | 10.39007 | 5.15E-10 | 2.18E-08 | Slc25a21 |
| 10459391 | -1.58443 | 7.859904 | 10.38492 | 5.20E-10 | 2.20E-08 | Fech |
| 10494565 | -1.19256 | 6.890119 | 10.37991 | 5.25E-10 | 2.21E-08 | Fmo5 |
| 10498576 | 1.939139 | 7.048043 | -10.3723 | 5.32E-10 | 2.24E-08 | Lxn |
| 10377924 | -2.7698 | 6.956625 | 10.35647 | 5.47E-10 | 2.30E-08 | Gp1ba |
| 10476321 | 2.208675 | 5.807898 | -10.3486 | 5.55E-10 | 2.32E-08 | Prn |
| 10536818 | 1.658272 | 9.456395 | -10.3427 | 5.61E-10 | 2.34E-08 | Calu |
| 10568638 | -1.21728 | 6.754896 | 10.34155 | 5.62E-10 | 2.34E-08 | Uros |
| 10457872 | 1.097394 | 6.13614 | -10.3342 | 5.70E-10 | 2.37E-08 | Slc39a6 |
| 10389214 | 1.488037 | 8.260228 | -10.3163 | 5.88E-10 | 2.43E-08 | Ccl9 |
| 10478884 | 2.130585 | 7.244162 | -10.3157 | 5.89E-10 | 2.43E-08 | Snai1 |
| 10344952 | 1.323626 | 6.543373 | -10.3072 | 5.98E-10 | 2.47E-08 | Rdh10 |
| 10577641 | 1.690512 | 6.174177 | -10.3038 | 6.02E-10 | 2.48E-08 | 1810011O10Rik |
| 10376950 | 1.119376 | 9.309415 | -10.2988 | 6.07E-10 | 2.50E-08 | Pmp22 |
| 10568174 | -1.59105 | 7.19299 | 10.2905 | 6.16E-10 | 2.53E-08 | Spn |
| 10530201 | 1.305792 | 8.21093 | -10.2766 | 6.32E-10 | 2.59E-08 | Ugdh |
| 10507040 | 1.099345 | 6.20176 | -10.2712 | 6.38E-10 | 2.61E-08 | Spata6 |
| 10400515 | 1.239248 | 7.853449 | -10.2641 | 6.47E-10 | 2.64E-08 | Sec23a |
| 10378216 | -2.31007 | 7.041949 | 10.26236 | 6.49E-10 | 2.65E-08 | Atp2a3 |
| 10435948 | 2.279903 | 8.289003 | -10.2398 | 6.76E-10 | 2.74E-08 | Ccdc80 |
| 10570278 | -1.052 | 5.397093 | 10.23967 | 6.76E-10 | 2.74E-08 | Gm15352 |
| 10569020 | -2.30126 | 9.973679 | 10.23588 | 6.80E-10 | 2.75E-08 | Ifitm6 |
| 10573924 | 2.066769 | 8.969975 | -10.2316 | 6.86E-10 | 2.77E-08 | Mmp2 |
| 10569877 | -2.00471 | 8.88913 | 10.23159 | 6.86E-10 | 2.77E-08 | Mcemp1 |
| 10445767 | -2.03894 | 7.072869 | 10.23066 | 6.87E-10 | 2.77E-08 | Treml2 |
| 10545921 | -1.59197 | 7.86323 | 10.23001 | 6.88E-10 | 2.77E-08 | Mxd1 |
| 10351293 | 2.615554 | 8.547064 | -10.2271 | 6.91E-10 | 2.78E-08 | Dpt |
| 10490818 | 1.079667 | 5.302218 | -10.2262 | 6.93E-10 | 2.78E-08 | Stmn2 |
| 10525195 | -1.70522 | 8.60537 | 10.22427 | 6.95E-10 | 2.79E-08 | Gm15800 |
| 10434806 | 1.506405 | 8.09651 | -10.2114 | 7.11E-10 | 2.84E-08 | Lpp |
| 10461629 | 1.384655 | 6.888555 | -10.205 | 7.20E-10 | 2.86E-08 | Ms4a4d |
| 10404152 | -2.0921 | 7.420688 | 10.19912 | 7.27E-10 | 2.89E-08 | Fam65b |
| 10443598 | -1.07382 | 5.041572 | 10.19758 | 7.29E-10 | 2.89E-08 | Dnah8 |
| 10461869 | 1.328269 | 5.126932 | -10.1962 | 7.31E-10 | 2.89E-08 | Prune2 |
| 10421172 | -2.7236 | 9.193552 | 10.19517 | 7.33E-10 | 2.90E-08 | Slc25a37 |
| 10376787 | 1.358216 | 6.175809 | -10.1861 | 7.45E-10 | 2.94E-08 | B9d1 |
| 10367919 | -1.13344 | 6.39301 | 10.18589 | 7.45E-10 | 2.94E-08 | Stx11 |
| 10354247 | 1.019905 | 7.024373 | -10.1839 | 7.48E-10 | 2.94E-08 | Fhl2 |
| 10590860 | 1.726512 | 6.095505 | -10.1762 | 7.58E-10 | 2.98E-08 | Arhgap42 |
| 10586781 | 1.255922 | 6.761483 | -10.1739 | 7.62E-10 | 2.98E-08 | Myo1e |
| 10469786 | -2.14255 | 7.03795 | 10.1736 | 7.62E-10 | 2.98E-08 | Il1f9 |
| 10509246 | 1.091391 | 6.975685 | -10.171 | 7.66E-10 | 2.99E-08 | Luzp1 |
| 10409579 | 1.317205 | 8.92432 | -10.1697 | 7.67E-10 | 2.99E-08 | Cxcl14 |
| 10400844 | -1.58535 | 8.567039 | 10.16634 | 7.72E-10 | 3.00E-08 | Pygl |
| 10485117 | 2.165422 | 7.64273 | -10.1652 | 7.74E-10 | 3.00E-08 | Creb3l1 |
| 10395409 | 1.998383 | 6.588261 | -10.1582 | 7.84E-10 | 3.03E-08 | Meox2 |
| 10568024 | -1.40093 | 8.926599 | 10.14388 | 8.04E-10 | 3.11E-08 | Coro1a |
| 10583952 | -1.17461 | 7.525914 | 10.14067 | 8.09E-10 | 3.12E-08 | Ncapd3 |
| 10391762 | 1.314872 | 5.681461 | -10.1332 | 8.20E-10 | 3.16E-08 | Gjc1 |
| 10447602 | -1.06163 | 7.837469 | 10.12914 | 8.26E-10 | 3.18E-08 | Ezr |
| 10444752 | -1.64833 | 6.551155 | 10.12133 | 8.38E-10 | 3.22E-08 | Ltb |
| 10441497 | 1.579758 | 7.46305 | -10.1098 | 8.56E-10 | 3.27E-08 | Tulp4 |
| 10493108 | 1.999458 | 6.414382 | -10.1093 | 8.57E-10 | 3.27E-08 | Crabp2 |
| 10414590 | -3.70414 | 5.69939 | 10.10708 | 8.60E-10 | 3.28E-08 | Ear6 |
| 10462140 | -1.45914 | 8.368257 | 10.10539 | 8.63E-10 | 3.28E-08 | Dock8 |
| 10581151 | 1.500544 | 5.722896 | -10.0919 | 8.85E-10 | 3.35E-08 | Rrad |
| 10584674 | 1.110767 | 7.133624 | -10.0915 | 8.85E-10 | 3.35E-08 | Mcam |
| 10565456 | 1.406593 | 6.203102 | -10.0865 | 8.93E-10 | 3.38E-08 | Prss23 |
| 10606355 | 1.578145 | 6.729964 | -10.0791 | 9.05E-10 | 3.42E-08 | Cysltr1 |
| 10544885 | 2.413263 | 6.864011 | -10.0763 | 9.10E-10 | 3.43E-08 | Fkbp14 |
| 10473432 | 1.00882 | 5.950594 | -10.0715 | 9.18E-10 | 3.46E-08 | Tnks1bp1 |
| 10473847 | 1.165534 | 7.583051 | -10.0712 | 9.19E-10 | 3.46E-08 | Acp2 |
| 10586907 | -1.37555 | 6.842448 | 10.06611 | 9.27E-10 | 3.48E-08 | Mns1 |
| 10356172 | 1.133187 | 6.872082 | -10.0624 | 9.33E-10 | 3.50E-08 | Pid1 |
| 10464471 | 2.353382 | 5.502609 | -10.0604 | 9.37E-10 | 3.51E-08 | Gal |
| 10542665 | -1.00512 | 9.013126 | 10.05842 | 9.40E-10 | 3.51E-08 | Cmas |
| 10606948 | 1.174049 | 5.40052 | -10.0542 | 9.48E-10 | 3.54E-08 | Morc4 |
| 10589420 | -1.04131 | 7.007477 | 10.05013 | 9.55E-10 | 3.56E-08 | Cdc25a |
| 10583529 | -1.72798 | 6.189148 | 10.04763 | 9.59E-10 | 3.57E-08 | Icam4 |
| 10392142 | -2.46178 | 7.7616 | 10.04232 | 9.69E-10 | 3.60E-08 | Cd79b |
| 10421361 | 1.823267 | 7.53564 | -10.0323 | 9.87E-10 | 3.66E-08 | Bmp1 |
| 10401673 | 1.884763 | 7.170079 | -10.0287 | 9.93E-10 | 3.67E-08 | Tgfb3 |
| 10560624 | 1.627251 | 11.11103 | -10.0268 | 9.97E-10 | 3.68E-08 | Apoe |
| 10547404 | 1.212777 | 6.84858 | -10.0212 | 1.01E-09 | 3.71E-08 | Erc1 |
| 10438639 | -1.73409 | 6.060981 | 10.01746 | 1.01E-09 | 3.73E-08 | Dgkg |
| 10600169 | 1.568997 | 10.97459 | -10.0142 | 1.02E-09 | 3.75E-08 | Bgn |
| 10570894 | -2.58421 | 7.948309 | 10.01081 | 1.03E-09 | 3.76E-08 | Ank1 |
| 10375062 | -1.76379 | 5.26055 | 10.0005 | 1.05E-09 | 3.83E-08 | Hbq1a |
| 10538871 | -2.64798 | 6.24751 | 9.991652 | 1.06E-09 | 3.88E-08 | Igkv1-135 |
| 10436978 | 1.197338 | 6.227328 | -9.98428 | 1.08E-09 | 3.91E-08 | Cbr3 |
| 10500813 | -1.11345 | 9.464579 | 9.982985 | 1.08E-09 | 3.92E-08 | Hipk1 |
| 10445442 | -1.17646 | 7.002881 | 9.976797 | 1.09E-09 | 3.96E-08 | Gtpbp2 |
| 10586306 | 1.032086 | 5.559492 | -9.97518 | 1.10E-09 | 3.96E-08 | Igdcc4 |
| 10607738 | 1.136508 | 4.973772 | -9.97348 | 1.10E-09 | 3.96E-08 | Car5b |
| 10425866 | -1.85304 | 6.92978 | 9.963263 | 1.12E-09 | 4.03E-08 | Parvg |
| 10554240 | -2.07328 | 8.388212 | 9.944922 | 1.16E-09 | 4.15E-08 | Isg20 |
| 10492815 | -1.41403 | 6.805611 | 9.942135 | 1.16E-09 | 4.17E-08 | Tmem154 |
| 10421418 | -2.20682 | 7.434803 | 9.939427 | 1.17E-09 | 4.18E-08 | Dmtn |
| 10370210 | 2.283441 | 7.65218 | -9.93875 | 1.17E-09 | 4.18E-08 | Col6a1 |
| 10414548 | -1.64124 | 7.00981 | 9.938348 | 1.17E-09 | 4.18E-08 | Rnase6 |
| 10546137 | -1.13591 | 6.740082 | 9.933129 | 1.18E-09 | 4.21E-08 | Abtb1 |
| 10355050 | 1.088602 | 7.507603 | -9.92003 | 1.21E-09 | 4.29E-08 | Raph1 |
| 10502778 | 1.441761 | 4.915769 | -9.92 | 1.21E-09 | 4.29E-08 | Lphn2 |
| 10369290 | 1.421455 | 6.70884 | -9.91949 | 1.21E-09 | 4.29E-08 | Ddit4 |
| 10588243 | 1.330387 | 8.084654 | -9.91916 | 1.22E-09 | 4.29E-08 | Ryk |
| 10582626 | -1.90666 | 6.485 | 9.910673 | 1.23E-09 | 4.35E-08 | Abcb10 |
| 10552824 | 1.270176 | 7.866906 | -9.90917 | 1.24E-09 | 4.36E-08 | Rras |
| 10568553 | -1.04795 | 6.951187 | 9.902989 | 1.25E-09 | 4.40E-08 | Chst15 |
| 10536908 | -2.74984 | 7.239487 | 9.90121 | 1.26E-09 | 4.41E-08 | Tspan33 |
| 10419559 | -1.01679 | 3.887717 | 9.89827 | 1.26E-09 | 4.43E-08 | Rnase12 |
| 10352905 | 1.284585 | 8.638532 | -9.86936 | 1.33E-09 | 4.64E-08 | Cd34 |
| 10371321 | 1.196583 | 5.919408 | -9.86912 | 1.33E-09 | 4.64E-08 | Slc41a2 |
| 10429114 | -1.62786 | 6.174249 | 9.868724 | 1.33E-09 | 4.64E-08 | Tmem71 |
| 10346015 | 2.806657 | 9.196252 | -9.86693 | 1.34E-09 | 4.65E-08 | Col3a1 |
| 10544462 | 1.277405 | 5.593761 | -9.86599 | 1.34E-09 | 4.65E-08 | Fam115a |
| 10451851 | 1.325905 | 6.708276 | -9.85876 | 1.36E-09 | 4.70E-08 | Armcx3 |
| 10414065 | 1.999405 | 6.155665 | -9.85469 | 1.37E-09 | 4.72E-08 | Anxa8 |
| 10585282 | -1.00703 | 6.019356 | 9.846128 | 1.39E-09 | 4.79E-08 | LOC102635638 |
| 10458293 | 1.009602 | 6.411891 | -9.81911 | 1.46E-09 | 5.01E-08 | Dnajc18 |
| 10361186 | 2.038919 | 6.523953 | -9.8161 | 1.47E-09 | 5.03E-08 | Sertad4 |
| 10393449 | 1.185868 | 6.421644 | -9.81541 | 1.47E-09 | 5.03E-08 | Socs3 |
| 10438891 | -1.79643 | 5.109195 | 9.814717 | 1.48E-09 | 5.03E-08 | Gm1968 |
| 10447510 | -1.09142 | 8.22807 | 9.80506 | 1.50E-09 | 5.11E-08 | Amd2 |
| 10554599 | 1.330917 | 5.115298 | -9.8021 | 1.51E-09 | 5.12E-08 | Adamtsl3 |
| 10594825 | -1.44701 | 6.565446 | 9.792077 | 1.54E-09 | 5.20E-08 | Aqp9 |
| 10525210 | -1.60004 | 7.759279 | 9.790069 | 1.54E-09 | 5.21E-08 | Gm15800 |
| 10474545 | -1.04188 | 7.667412 | 9.784614 | 1.56E-09 | 5.25E-08 | Slc12a6 |
| 10399314 | -2.76776 | 7.216525 | 9.779315 | 1.58E-09 | 5.29E-08 | Mfsd2b |
| 10378240 | -1.80337 | 5.627695 | 9.778105 | 1.58E-09 | 5.30E-08 | P2rx1 |
| 10550509 | -2.57421 | 9.506697 | 9.767665 | 1.61E-09 | 5.40E-08 | Pglyrp1 |
| 10587266 | -1.26572 | 7.731552 | 9.766703 | 1.61E-09 | 5.40E-08 | Gclc |
| 10519578 | -1.73977 | 6.893201 | 9.765351 | 1.62E-09 | 5.41E-08 | Abcb4 |
| 10492689 | 1.649444 | 6.64948 | -9.75062 | 1.66E-09 | 5.54E-08 | Pdgfc |
| 10383289 | 1.05724 | 6.092432 | -9.74887 | 1.67E-09 | 5.55E-08 | Baiap2 |
| 10570855 | 1.540812 | 6.25561 | -9.74659 | 1.68E-09 | 5.55E-08 | Plat |
| 10469358 | 1.872373 | 8.709221 | -9.74655 | 1.68E-09 | 5.55E-08 | Mrc1 |
| 10603492 | 1.232786 | 6.544721 | -9.74631 | 1.68E-09 | 5.55E-08 | Porcn |
| 10351224 | -2.24529 | 6.568062 | 9.745409 | 1.68E-09 | 5.56E-08 | F5 |
| 10556297 | 1.258913 | 4.913535 | -9.7422 | 1.69E-09 | 5.58E-08 | Adm |
| 10372917 | 1.189667 | 6.545007 | -9.73972 | 1.70E-09 | 5.60E-08 | Tmem5 |
| 10484927 | 1.096738 | 7.286885 | -9.73761 | 1.70E-09 | 5.60E-08 | Slc39a13 |
| 10538880 | -3.61895 | 5.695656 | 9.731678 | 1.72E-09 | 5.65E-08 | Igkv1-117 |
| 10487447 | 1.07993 | 6.243315 | -9.72673 | 1.74E-09 | 5.69E-08 | Mall |
| 10409799 | -1.05317 | 8.301226 | 9.725579 | 1.74E-09 | 5.69E-08 | Isca1 |
| 10539669 | -1.55409 | 5.798549 | 9.719508 | 1.76E-09 | 5.75E-08 | Add2 |
| 10350297 | -1.08943 | 7.36456 | 9.714953 | 1.78E-09 | 5.78E-08 | Kif14 |
| 10460968 | -1.95833 | 7.623907 | 9.710851 | 1.79E-09 | 5.81E-08 | Rasgrp2 |
| 10466190 | 1.75425 | 5.582166 | -9.70998 | 1.79E-09 | 5.81E-08 | Ms4a14 |
| 10495147 | -1.03379 | 5.064959 | 9.709673 | 1.80E-09 | 5.81E-08 | Dennd2d |
| 10394674 | 1.46297 | 6.88018 | -9.70898 | 1.80E-09 | 5.81E-08 | Socs2 |
| 10518352 | -1.25149 | 10.34662 | 9.705052 | 1.81E-09 | 5.85E-08 | Gm13160 |
| 10493114 | 1.983148 | 5.190455 | -9.70308 | 1.82E-09 | 5.85E-08 | Nes |
| 10393320 | -1.84532 | 8.119652 | 9.696826 | 1.84E-09 | 5.89E-08 | Ube2o |
| 10441601 | -1.03539 | 5.651037 | 9.696621 | 1.84E-09 | 5.89E-08 | Tagap |
| 10513869 | -1.28537 | 7.267878 | 9.683585 | 1.89E-09 | 6.02E-08 | Megf9 |
| 10394735 | 1.107311 | 8.737177 | -9.67872 | 1.90E-09 | 6.07E-08 | Pdia6 |
| 10482920 | 1.063704 | 7.670256 | -9.67371 | 1.92E-09 | 6.11E-08 | Cd302 |
| 10440091 | 1.928488 | 7.896736 | -9.66578 | 1.95E-09 | 6.19E-08 | Col8a1 |
| 10406736 | -2.72054 | 7.246887 | 9.663788 | 1.96E-09 | 6.21E-08 | F2rl2 |
| 10571444 | 1.073539 | 6.120577 | -9.6622 | 1.96E-09 | 6.22E-08 | Slc7a2 |
| 10546294 | -1.95587 | 7.570088 | 9.661289 | 1.97E-09 | 6.22E-08 | Nup210 |
| 10422962 | -1.00604 | 7.408093 | 9.66118 | 1.97E-09 | 6.22E-08 | Nadk2 |
| 10472350 | -2.39678 | 6.875926 | 9.660333 | 1.97E-09 | 6.22E-08 | Gca |
| 10398069 | 1.62965 | 5.167676 | -9.65919 | 1.97E-09 | 6.23E-08 | Serpina3m |
| 10404024 | -1.21167 | 8.621113 | 9.650578 | 2.01E-09 | 6.32E-08 | Hist1h4h |
| 10358421 | -2.19788 | 7.842812 | 9.648372 | 2.01E-09 | 6.34E-08 | Rgs18 |
| 10518350 | -1.23192 | 10.65041 | 9.64764 | 2.02E-09 | 6.34E-08 | Hmgb2 |
| 10432640 | -1.93135 | 7.493834 | 9.641097 | 2.04E-09 | 6.40E-08 | Bin2 |
| 10571870 | -1.19562 | 10.73141 | 9.630812 | 2.08E-09 | 6.51E-08 | Hmgb2 |
| 10500808 | 1.53507 | 8.478675 | -9.62644 | 2.10E-09 | 6.55E-08 | Olfml3 |
| 10445574 | 1.315234 | 5.30694 | -9.62386 | 2.11E-09 | 6.57E-08 | Cul7 |
| 10544501 | -1.0431 | 8.247498 | 9.622737 | 2.11E-09 | 6.58E-08 | Ezh2 |
| 10497337 | -3.86627 | 9.639828 | 9.619741 | 2.13E-09 | 6.61E-08 | Car1 |
| 10537184 | 1.225991 | 8.289223 | -9.61954 | 2.13E-09 | 6.61E-08 | Cald1 |
| 10375083 | -1.31644 | 6.863339 | 9.617709 | 2.13E-09 | 6.61E-08 | Stk10 |
| 10457168 | -2.36982 | 6.364524 | 9.615183 | 2.14E-09 | 6.63E-08 | Cd226 |
| 10458906 | 1.86756 | 9.578935 | -9.61449 | 2.15E-09 | 6.63E-08 | Ppic |
| 10498160 | 1.241933 | 7.62441 | -9.60971 | 2.17E-09 | 6.69E-08 | Ufm1 |
| 10396952 | 1.116952 | 4.775914 | -9.60031 | 2.21E-09 | 6.78E-08 | Ttc9 |
| 10527229 | -1.72793 | 8.196841 | 9.59236 | 2.24E-09 | 6.88E-08 | Fam220a |
| 10375002 | -1.18947 | 8.606389 | 9.584335 | 2.27E-09 | 6.96E-08 | Cpeb4 |
| 10355329 | -1.10689 | 5.72795 | 9.579818 | 2.29E-09 | 7.01E-08 | Bard1 |
| 10447951 | 2.513928 | 8.148669 | -9.57305 | 2.32E-09 | 7.08E-08 | Thbs2 |
| 10456071 | 1.270266 | 8.337311 | -9.57248 | 2.33E-09 | 7.08E-08 | Csf1r |
| 10595466 | 1.368808 | 6.347643 | -9.57001 | 2.34E-09 | 7.10E-08 | Pgm3 |
| 10411082 | 1.905211 | 9.089714 | -9.56763 | 2.35E-09 | 7.12E-08 | Thbs4 |
| 10587871 | -1.7263 | 6.061319 | 9.561468 | 2.37E-09 | 7.20E-08 | Paqr9 |
| 10427744 | 1.111338 | 7.051627 | -9.56005 | 2.38E-09 | 7.21E-08 | Rai14 |
| 10374727 | -1.93111 | 6.997149 | 9.557481 | 2.39E-09 | 7.24E-08 | Bcl11a |
| 10393559 | 1.166883 | 9.280421 | -9.55604 | 2.40E-09 | 7.25E-08 | Timp2 |
| 10542164 | -1.41575 | 9.085503 | 9.542803 | 2.46E-09 | 7.41E-08 | Clec12a |
| 10520388 | -1.65823 | 7.331276 | 9.541864 | 2.46E-09 | 7.42E-08 | Rbm33 |
| 10533131 | 1.030761 | 6.842305 | -9.53869 | 2.48E-09 | 7.46E-08 | Plbd2 |
| 10598507 | -2.14486 | 6.285359 | 9.534913 | 2.50E-09 | 7.49E-08 | Slc38a5 |
| 10448559 | -1.15834 | 4.322567 | 9.533845 | 2.50E-09 | 7.49E-08 | D330041H03Rik |
| 10407081 | -1.39656 | 6.227158 | 9.529504 | 2.52E-09 | 7.54E-08 | Depdc1b |
| 10574572 | -2.23431 | 7.290408 | 9.527781 | 2.53E-09 | 7.55E-08 | Ces2g |
| 10363350 | 1.594007 | 6.833442 | -9.52659 | 2.54E-09 | 7.56E-08 | P4ha1 |
| 10375167 | -1.0712 | 5.695519 | 9.51911 | 2.57E-09 | 7.65E-08 | Fam196b |
| 10554814 | 1.443864 | 7.55741 | -9.51515 | 2.59E-09 | 7.70E-08 | 1700019G06Rik |
| 10507594 | 1.072624 | 7.418004 | -9.50832 | 2.63E-09 | 7.78E-08 | Slc2a1 |
| 10346168 | -1.63232 | 5.746475 | 9.50699 | 2.63E-09 | 7.80E-08 | Stat4 |
| 10364535 | -2.78039 | 8.56316 | 9.502027 | 2.66E-09 | 7.85E-08 | Elane |
| 10503259 | -1.23734 | 8.25957 | 9.500081 | 2.67E-09 | 7.88E-08 | Trp53inp1 |
| 10430195 | -1.36056 | 6.175947 | 9.49024 | 2.72E-09 | 8.01E-08 | Apol8 |
| 10466779 | -1.93171 | 6.980761 | 9.48737 | 2.73E-09 | 8.03E-08 | Pip5k1b |
| 10502780 | 1.067159 | 6.731053 | -9.48509 | 2.74E-09 | 8.06E-08 | Lphn2 |
| 10383025 | 1.327284 | 6.422451 | -9.47691 | 2.79E-09 | 8.14E-08 | C1qtnf1 |
| 10526783 | 1.37766 | 5.248591 | -9.47652 | 2.79E-09 | 8.14E-08 | Nxpe5 |
| 10502805 | 1.436626 | 6.349209 | -9.47516 | 2.80E-09 | 8.16E-08 | Ptgfr |
| 10589703 | -2.92255 | 10.06112 | 9.470459 | 2.82E-09 | 8.21E-08 | Ltf |
| 10385893 | -1.06758 | 6.391342 | 9.470056 | 2.82E-09 | 8.21E-08 | Slc22a4 |
| 10566934 | 1.193946 | 6.969481 | -9.46902 | 2.83E-09 | 8.22E-08 | Lyve1 |
| 10407985 | -1.49785 | 6.469532 | 9.468425 | 2.83E-09 | 8.22E-08 | Gpr141 |
| 10467319 | 1.614311 | 6.279051 | -9.46415 | 2.86E-09 | 8.27E-08 | Rbp4 |
| 10576973 | 1.190326 | 6.68247 | -9.45181 | 2.92E-09 | 8.45E-08 | Col4a1 |
| 10389719 | 1.314179 | 8.098797 | -9.44073 | 2.99E-09 | 8.61E-08 | Scpep1 |
| 10510574 | 1.468234 | 7.650361 | -9.44043 | 2.99E-09 | 8.61E-08 | Errfi1 |
| 10346348 | 1.21278 | 6.043185 | -9.43777 | 3.00E-09 | 8.65E-08 | Spats2l |
| 10357698 | -1.76894 | 6.613663 | 9.436538 | 3.01E-09 | 8.66E-08 | Tmcc2 |
| 10362245 | 1.129932 | 8.237909 | -9.43405 | 3.03E-09 | 8.69E-08 | Epb4.1l2 |
| 10547153 | -1.62613 | 7.439263 | 9.430872 | 3.04E-09 | 8.73E-08 | Alox5 |
| 10601581 | -1.01166 | 7.548665 | 9.42973 | 3.05E-09 | 8.74E-08 | Gm4992 |
| 10420631 | 1.141737 | 6.95889 | -9.42832 | 3.06E-09 | 8.75E-08 | Ebpl |
| 10585338 | 1.449014 | 5.6302 | -9.42184 | 3.10E-09 | 8.84E-08 | Kdelc2 |
| 10350864 | 1.436188 | 5.772316 | -9.42101 | 3.10E-09 | 8.85E-08 | Sec16b |
| 10580349 | -1.13429 | 5.929197 | 9.415904 | 3.13E-09 | 8.92E-08 | Mylk3 |
| 10586477 | 1.286933 | 9.752018 | -9.40876 | 3.17E-09 | 9.02E-08 | Ppib |
| 10485372 | -1.76965 | 5.093153 | 9.407657 | 3.18E-09 | 9.02E-08 | Rag1 |
| 10408531 | 1.186694 | 7.009276 | -9.40354 | 3.21E-09 | 9.09E-08 | Gmds |
| 10482929 | -1.24506 | 6.438102 | 9.396378 | 3.25E-09 | 9.19E-08 | Ly75 |
| 10545202 | -1.59244 | 6.028288 | 9.389036 | 3.30E-09 | 9.31E-08 | LOC102642862 |
| 10598771 | 1.209313 | 5.660751 | -9.38731 | 3.31E-09 | 9.33E-08 | Maoa |
| 10522060 | 1.709898 | 7.995962 | -9.37667 | 3.38E-09 | 9.50E-08 | Fam114a1 |
| 10595664 | 1.167447 | 9.101896 | -9.37593 | 3.38E-09 | 9.50E-08 | Tmed3 |
| 10458663 | 1.70802 | 7.215188 | -9.37059 | 3.42E-09 | 9.57E-08 | Dpysl3 |
| 10522788 | -1.92377 | 6.368879 | 9.369034 | 3.43E-09 | 9.59E-08 | Stap1 |
| 10515943 | 1.128322 | 6.913329 | -9.35072 | 3.55E-09 | 9.87E-08 | Ctps |
| 10559796 | 2.069582 | 6.724875 | -9.339 | 3.63E-09 | 1.01E-07 | Peg3 |
| 10476945 | -2.00246 | 7.035434 | 9.33654 | 3.65E-09 | 1.01E-07 | Cst7 |
| 10348817 | 1.196342 | 9.00268 | -9.33103 | 3.69E-09 | 1.01E-07 | 44441 |
| 10376778 | 2.184646 | 6.998857 | -9.32677 | 3.72E-09 | 1.02E-07 | Mfap4 |
| 10380514 | -1.50638 | 8.016453 | 9.323099 | 3.74E-09 | 1.03E-07 | Fam117a |
| 10578649 | 1.296891 | 6.055623 | -9.31692 | 3.79E-09 | 1.03E-07 | Tenm3 |
| 10548892 | -1.1234 | 10.44192 | 9.310779 | 3.83E-09 | 1.05E-07 | Arhgdib |
| 10495685 | 1.419344 | 6.951613 | -9.3093 | 3.84E-09 | 1.05E-07 | Arhgap29 |
| 10584628 | 1.841599 | 8.640494 | -9.30441 | 3.88E-09 | 1.05E-07 | Thy1 |
| 10603583 | 1.854325 | 6.458799 | -9.30016 | 3.91E-09 | 1.06E-07 | Srpx |
| 10548817 | -1.26839 | 9.427326 | 9.299592 | 3.92E-09 | 1.06E-07 | Plbd1 |
| 10575548 | -1.30477 | 7.313692 | 9.297474 | 3.93E-09 | 1.06E-07 | Gm26132 |
| 10553833 | 2.030676 | 5.532335 | -9.29572 | 3.94E-09 | 1.07E-07 | Ndn |
| 10523255 | 1.2584 | 5.247265 | -9.29522 | 3.95E-09 | 1.07E-07 | Stbd1 |
| 10481147 | 1.114137 | 9.2552 | -9.287 | 4.01E-09 | 1.08E-07 | Surf4 |
| 10421950 | -1.35908 | 6.092033 | 9.28317 | 4.04E-09 | 1.09E-07 | Dach1 |
| 10360018 | -1.16454 | 5.099853 | 9.280962 | 4.06E-09 | 1.09E-07 | Fcrla |
| 10435288 | -1.20045 | 4.927137 | 9.27801 | 4.08E-09 | 1.09E-07 | Muc13 |
| 10399419 | 1.305148 | 5.180661 | -9.27574 | 4.10E-09 | 1.10E-07 | Tubb2b |
| 10360090 | -1.67327 | 7.524402 | 9.275442 | 4.10E-09 | 1.10E-07 | Ppox |
| 10379736 | -2.00761 | 10.03605 | 9.272578 | 4.12E-09 | 1.10E-07 | Wfdc21 |
| 10469828 | -1.17388 | 6.81611 | 9.265985 | 4.18E-09 | 1.11E-07 | Psd4 |
| 10443009 | 1.250396 | 8.493963 | -9.25728 | 4.25E-09 | 1.13E-07 | Ergic1 |
| 10413710 | 2.149477 | 7.239396 | -9.25318 | 4.28E-09 | 1.13E-07 | Nt5dc2 |
| 10500685 | 1.048469 | 9.40034 | -9.24789 | 4.33E-09 | 1.14E-07 | Atp1a1 |
| 10591660 | -1.76537 | 6.510965 | 9.23616 | 4.43E-09 | 1.17E-07 | Epor |
| 10476252 | -1.42559 | 7.270389 | 9.234851 | 4.44E-09 | 1.17E-07 | Cdc25b |
| 10381809 | -2.13369 | 7.694722 | 9.234764 | 4.44E-09 | 1.17E-07 | Itgb3 |
| 10443408 | -1.13586 | 6.52788 | 9.230736 | 4.47E-09 | 1.17E-07 | Mapk13 |
| 10578572 | 1.650091 | 6.426634 | -9.22667 | 4.51E-09 | 1.18E-07 | Stox2 |
| 10344981 | 1.342354 | 4.899333 | -9.22517 | 4.52E-09 | 1.18E-07 | Pi15 |
| 10466224 | -2.7118 | 7.962314 | 9.219413 | 4.57E-09 | 1.20E-07 | Ms4a3 |
| 10601768 | 1.136821 | 5.540386 | -9.21392 | 4.62E-09 | 1.21E-07 | Armcx4 |
| 10515257 | -1.18257 | 6.747804 | 9.210555 | 4.65E-09 | 1.21E-07 | Rad54l |
| 10552125 | 1.049114 | 6.120868 | -9.20887 | 4.67E-09 | 1.22E-07 | Pepd |
| 10456140 | -1.00314 | 5.24858 | 9.197862 | 4.77E-09 | 1.24E-07 | Sh3tc2 |
| 10549733 | 1.206473 | 6.05445 | -9.18838 | 4.85E-09 | 1.26E-07 | Ssc5d |
| 10378816 | -1.74065 | 5.703392 | 9.184833 | 4.89E-09 | 1.26E-07 | Slc6a4 |
| 10450412 | -2.35057 | 6.582656 | 9.181797 | 4.92E-09 | 1.27E-07 | G6b |
| 10419416 | 1.733801 | 5.446341 | -9.17653 | 4.97E-09 | 1.28E-07 | 3632451O06Rik |
| 10408600 | 1.110341 | 9.694573 | -9.17178 | 5.01E-09 | 1.29E-07 | Serpinb6a |
| 10429926 | -1.00628 | 7.575549 | 9.168241 | 5.05E-09 | 1.30E-07 | Dgat1 |
| 10363575 | -1.02625 | 6.648815 | 9.162046 | 5.11E-09 | 1.31E-07 | Dna2 |
| 10600597 | 1.479519 | 6.902408 | -9.15584 | 5.17E-09 | 1.32E-07 | Tmem47 |
| 10436095 | -2.29446 | 4.948923 | 9.155573 | 5.17E-09 | 1.32E-07 | Retnla |
| 10566943 | -1.54546 | 6.260618 | 9.146984 | 5.26E-09 | 1.34E-07 | Mrvi1 |
| 10523128 | -2.71859 | 10.9806 | 9.146345 | 5.27E-09 | 1.34E-07 | Ppbp |
| 10494804 | 1.884256 | 5.89404 | -9.1446 | 5.29E-09 | 1.35E-07 | Casq2 |
| 10559248 | -1.68431 | 6.539273 | 9.135055 | 5.39E-09 | 1.37E-07 | Tspan32 |
| 10505717 | 1.369732 | 5.809574 | -9.1347 | 5.39E-09 | 1.37E-07 | Adamtsl1 |
| 10402142 | -1.19464 | 7.070913 | 9.134659 | 5.39E-09 | 1.37E-07 | Ccdc88c |
| 10460108 | 1.38008 | 7.07645 | -9.12948 | 5.44E-09 | 1.38E-07 | Gnpnat1 |
| 10598586 | -1.47748 | 6.348806 | 9.12493 | 5.49E-09 | 1.39E-07 | Xk |
| 10366746 | 1.422762 | 5.482075 | -9.12458 | 5.50E-09 | 1.39E-07 | Lrig3 |
| 10383564 | -1.4565 | 6.340879 | 9.117615 | 5.57E-09 | 1.40E-07 | Fn3k |
| 10567825 | -1.5771 | 6.923837 | 9.107842 | 5.68E-09 | 1.43E-07 | Lat |
| 10355327 | -1.20972 | 6.779284 | 9.105805 | 5.70E-09 | 1.43E-07 | Bard1 |
| 10436100 | -2.05355 | 11.11421 | 9.103465 | 5.73E-09 | 1.43E-07 | Retnlg |
| 10601854 | 1.463772 | 9.020187 | -9.10287 | 5.73E-09 | 1.43E-07 | Wbp5 |
| 10559547 | 1.805509 | 7.7517 | -9.09827 | 5.79E-09 | 1.44E-07 | Tnnt1 |
| 10548875 | -1.39745 | 6.615346 | 9.090112 | 5.88E-09 | 1.46E-07 | Art4 |
| 10499062 | -1.81613 | 6.759291 | 9.089568 | 5.88E-09 | 1.46E-07 | Fhdc1 |
| 10547740 | 1.34937 | 6.855176 | -9.08326 | 5.96E-09 | 1.47E-07 | C1s1 |
| 10567863 | -1.94636 | 5.953117 | 9.083157 | 5.96E-09 | 1.47E-07 | Cd19 |
| 10349559 | -1.05062 | 6.970383 | 9.080604 | 5.99E-09 | 1.48E-07 | Yod1 |
| 10469906 | 1.0052 | 6.47554 | -9.08015 | 5.99E-09 | 1.48E-07 | Nsmf |
| 10511617 | 1.699035 | 6.990118 | -9.06801 | 6.14E-09 | 1.51E-07 | Fam92a |
| 10365749 | -1.21163 | 8.034672 | 9.062856 | 6.20E-09 | 1.53E-07 | Lta4h |
| 10481304 | -2.07829 | 7.849369 | 9.058948 | 6.25E-09 | 1.54E-07 | Gfi1b |
| 10499168 | 1.231892 | 6.541651 | -9.05108 | 6.34E-09 | 1.56E-07 | Kirrel |
| 10498383 | 1.667442 | 7.295105 | -9.04868 | 6.37E-09 | 1.56E-07 | Igsf10 |
| 10359235 | 1.038086 | 6.094774 | -9.03298 | 6.57E-09 | 1.60E-07 | Rasal2 |
| 10433264 | 1.168615 | 7.582129 | -9.01555 | 6.80E-09 | 1.65E-07 | Glis2 |
| 10431697 | -1.25131 | 6.581718 | 9.01485 | 6.81E-09 | 1.65E-07 | Abcd2 |
| 10576332 | 1.444564 | 5.672562 | -9.01191 | 6.85E-09 | 1.66E-07 | Tubb3 |
| 10377560 | 1.039605 | 5.310636 | -8.98785 | 7.18E-09 | 1.72E-07 | Sat2 |
| 10446376 | 1.201614 | 8.466266 | -8.97868 | 7.31E-09 | 1.75E-07 | Man2a1 |
| 10413609 | 1.772503 | 9.860766 | -8.97124 | 7.42E-09 | 1.77E-07 | Mustn1 |
| 10468762 | 1.325583 | 6.452519 | -8.96829 | 7.46E-09 | 1.78E-07 | 4930506M07Rik |
| 10593123 | 1.166463 | 6.154808 | -8.96602 | 7.50E-09 | 1.79E-07 | Tagln |
| 10473349 | -2.70283 | 7.589551 | 8.964625 | 7.52E-09 | 1.79E-07 | Ypel4 |
| 10420261 | -2.63274 | 8.354571 | 8.961503 | 7.57E-09 | 1.80E-07 | Ctsg |
| 10547056 | -1.58356 | 6.691958 | 8.955682 | 7.65E-09 | 1.81E-07 | Tmem40 |
| 10379727 | 2.165118 | 10.12519 | -8.95443 | 7.67E-09 | 1.81E-07 | Wfdc17 |
| 10534927 | -1.42831 | 8.353466 | 8.951429 | 7.72E-09 | 1.82E-07 | Pilra |
| 10481592 | 1.320154 | 6.964823 | -8.95025 | 7.74E-09 | 1.82E-07 | Dnm1 |
| 10379652 | -1.12879 | 5.42331 | 8.947423 | 7.78E-09 | 1.83E-07 | Snord7 |
| 10509901 | 1.784959 | 7.356498 | -8.94726 | 7.78E-09 | 1.83E-07 | Mfap2 |
| 10474860 | 1.106917 | 7.035206 | -8.94501 | 7.82E-09 | 1.84E-07 | Chst14 |
| 10592355 | 2.632704 | 6.221471 | -8.9387 | 7.91E-09 | 1.86E-07 | Panx3 |
| 10510172 | -1.15732 | 10.52569 | 8.934318 | 7.98E-09 | 1.87E-07 | Hmgb2 |
| 10493193 | 1.022816 | 8.259824 | -8.93409 | 7.99E-09 | 1.87E-07 | Cct3 |
| 10462818 | -1.02935 | 6.726754 | 8.930369 | 8.04E-09 | 1.88E-07 | Hhex |
| 10542953 | 1.383789 | 6.28849 | -8.92697 | 8.10E-09 | 1.89E-07 | Tfpi2 |
| 10509163 | 1.263074 | 8.364683 | -8.92641 | 8.11E-09 | 1.89E-07 | Id3 |
| 10397633 | 1.430682 | 7.268284 | -8.9247 | 8.14E-09 | 1.89E-07 | Flrt2 |
| 10583669 | -1.03558 | 8.774887 | 8.916485 | 8.27E-09 | 1.92E-07 | AB124611 |
| 10415396 | 1.09521 | 5.406167 | -8.91293 | 8.33E-09 | 1.93E-07 | Nfatc4 |
| 10584561 | 1.336649 | 7.355387 | -8.91252 | 8.33E-09 | 1.93E-07 | Clmp |
| 10500204 | 1.913291 | 9.469788 | -8.91224 | 8.34E-09 | 1.93E-07 | Ecm1 |
| 10493995 | 1.14538 | 10.49948 | -8.91086 | 8.36E-09 | 1.93E-07 | S100a10 |
| 10419296 | -1.28231 | 7.319517 | 8.907715 | 8.41E-09 | 1.94E-07 | Wdhd1 |
| 10498620 | -1.65685 | 8.335441 | 8.89921 | 8.56E-09 | 1.97E-07 | Trim59 |
| 10578810 | -1.41421 | 7.13915 | 8.895361 | 8.62E-09 | 1.98E-07 | Clcn3 |
| 10393047 | 1.031768 | 6.763464 | -8.89432 | 8.64E-09 | 1.98E-07 | Galk1 |
| 10382956 | -1.63962 | 6.062539 | 8.887283 | 8.76E-09 | 2.00E-07 | Tmc8 |
| 10530633 | 1.254093 | 7.227792 | -8.88637 | 8.78E-09 | 2.00E-07 | Sgcb |
| 10391649 | -3.54064 | 9.263343 | 8.885471 | 8.79E-09 | 2.01E-07 | Slc4a1 |
| 10517488 | 1.181484 | 5.901292 | -8.88193 | 8.85E-09 | 2.01E-07 | Ephb2 |
| 10502655 | 1.819543 | 8.407636 | -8.88139 | 8.86E-09 | 2.01E-07 | Cyr61 |
| 10486119 | -1.63311 | 6.919084 | 8.873465 | 9.00E-09 | 2.04E-07 | Plcb2 |
| 10607475 | 1.47067 | 9.069066 | -8.86773 | 9.11E-09 | 2.06E-07 | Prdx4 |
| 10586246 | -1.27743 | 9.076417 | 8.842046 | 9.58E-09 | 2.15E-07 | Dennd4a |
| 10500982 | -1.68651 | 7.532384 | 8.836954 | 9.68E-09 | 2.17E-07 | I830077J02Rik |
| 10542140 | -1.55012 | 5.701168 | 8.834889 | 9.72E-09 | 2.18E-07 | Klrb1f |
| 10380174 | -2.66002 | 9.855933 | 8.832499 | 9.76E-09 | 2.18E-07 | Mpo |
| 10415844 | 1.041521 | 10.33329 | -8.83174 | 9.78E-09 | 2.19E-07 | Ctsb |
| 10536294 | 1.225674 | 6.319676 | -8.82672 | 9.88E-09 | 2.21E-07 | Peg10 |
| 10534202 | -1.46381 | 8.489782 | 8.82413 | 9.93E-09 | 2.22E-07 | Ncf1 |
| 10443421 | -1.06788 | 6.699211 | 8.823147 | 9.95E-09 | 2.22E-07 | Brpf3 |
| 10587554 | 1.385753 | 6.40522 | -8.81969 | 1.00E-08 | 2.23E-07 | Tpbg |
| 10377782 | 1.685785 | 5.645603 | -8.81634 | 1.01E-08 | 2.25E-07 | Clec10a |
| 10394471 | 1.332288 | 5.974188 | -8.81284 | 1.02E-08 | 2.25E-07 | Sdc1 |
| 10403031 | -2.92557 | 6.458702 | 8.809494 | 1.02E-08 | 2.27E-07 | Ighv1-55 |
| 10372028 | -1.20191 | 7.184086 | 8.800892 | 1.04E-08 | 2.30E-07 | Plxnc1 |
| 10509122 | -1.06309 | 6.306883 | 8.794552 | 1.05E-08 | 2.33E-07 | Cnr2 |
| 10553057 | 1.360681 | 6.076611 | -8.78489 | 1.07E-08 | 2.36E-07 | Mamstr |
| 10379215 | 1.185477 | 8.579759 | -8.78037 | 1.08E-08 | 2.37E-07 | Ift20 |
| 10606735 | 1.3069 | 5.53621 | -8.77752 | 1.09E-08 | 2.38E-07 | Armcx2 |
| 10421697 | 1.126474 | 6.452262 | -8.7774 | 1.09E-08 | 2.38E-07 | Lacc1 |
| 10579812 | 1.030303 | 6.26152 | -8.7735 | 1.10E-08 | 2.40E-07 | Ednra |
| 10449163 | -1.68671 | 8.678234 | 8.767686 | 1.11E-08 | 2.42E-07 | Pigq |
| 10534102 | 1.068403 | 7.087872 | -8.76765 | 1.11E-08 | 2.42E-07 | Gusb |
| 10586227 | -1.05226 | 9.146151 | 8.764056 | 1.12E-08 | 2.43E-07 | Dennd4a |
| 10522653 | -2.081 | 5.914443 | 8.763822 | 1.12E-08 | 2.43E-07 | A730089K16Rik |
| 10361055 | 1.301747 | 5.200783 | -8.75544 | 1.14E-08 | 2.47E-07 | Vash2 |
| 10408200 | -1.72491 | 9.27603 | 8.754343 | 1.14E-08 | 2.47E-07 | Hist1h4f |
| 10354389 | 1.00636 | 6.377114 | -8.7486 | 1.15E-08 | 2.49E-07 | Slc39a10 |
| 10605437 | 2.104662 | 7.42933 | -8.74848 | 1.15E-08 | 2.49E-07 | Pls3 |
| 10451646 | -1.42716 | 6.684033 | 8.748011 | 1.16E-08 | 2.49E-07 | A530064D06Rik |
| 10586242 | -1.27308 | 9.125241 | 8.722014 | 1.22E-08 | 2.62E-07 | Dennd4a |
| 10512747 | -1.08952 | 6.712935 | 8.721724 | 1.22E-08 | 2.62E-07 | 5830415F09Rik |
| 10362073 | 1.147913 | 7.322413 | -8.72042 | 1.22E-08 | 2.62E-07 | Sgk1 |
| 10400336 | 1.025746 | 8.030665 | -8.71765 | 1.23E-08 | 2.63E-07 | Snx6 |
| 10362314 | 1.179148 | 6.411346 | -8.71487 | 1.23E-08 | 2.64E-07 | Ptprk |
| 10604564 | 1.044476 | 6.901867 | -8.71186 | 1.24E-08 | 2.66E-07 | Gpc4 |
| 10370603 | -1.12235 | 4.812432 | 8.704307 | 1.26E-08 | 2.69E-07 | Odf3l2 |
| 10419082 | -2.22064 | 7.841388 | 8.703495 | 1.26E-08 | 2.69E-07 | Fam213a |
| 10514510 | 1.090453 | 5.24363 | -8.70132 | 1.27E-08 | 2.70E-07 | Cyp2j6 |
| 10421186 | -1.80515 | 6.199864 | 8.700584 | 1.27E-08 | 2.71E-07 | Gm10002 |
| 10484207 | 1.130273 | 5.109936 | -8.68837 | 1.30E-08 | 2.76E-07 | Ccdc141 |
| 10428534 | 1.39864 | 7.755956 | -8.68503 | 1.31E-08 | 2.78E-07 | Trps1 |
| 10361375 | -1.54605 | 8.42298 | 8.684883 | 1.31E-08 | 2.78E-07 | Fbxo5 |
| 10504670 | -1.14427 | 5.897886 | 8.677532 | 1.33E-08 | 2.81E-07 | E230008N13Rik |
| 10452613 | 1.207046 | 4.984818 | -8.67452 | 1.34E-08 | 2.82E-07 | Arhgap28 |
| 10540472 | 1.246907 | 7.454532 | -8.67412 | 1.34E-08 | 2.82E-07 | Bhlhe40 |
| 10438405 | -2.54168 | 7.563981 | 8.658095 | 1.38E-08 | 2.91E-07 | Iglv1 |
| 10570982 | 1.113938 | 7.5123 | -8.64247 | 1.43E-08 | 2.98E-07 | Fgfr1 |
| 10473406 | -1.79654 | 5.282502 | 8.637099 | 1.44E-08 | 3.01E-07 | Prg3 |
| 10354309 | 2.530373 | 7.764058 | -8.63578 | 1.45E-08 | 3.02E-07 | Col5a2 |
| 10354286 | 1.050884 | 6.346144 | -8.63111 | 1.46E-08 | 3.04E-07 | Kdelc1 |
| 10403957 | -1.07816 | 11.5208 | 8.628448 | 1.47E-08 | 3.05E-07 | Hist1h4m |
| 10408092 | -1.07816 | 11.5208 | 8.628448 | 1.47E-08 | 3.05E-07 | Hist1h4m |
| 10380403 | 1.108696 | 8.392765 | -8.62642 | 1.47E-08 | 3.06E-07 | Lrrc59 |
| 10403028 | -2.23416 | 5.782414 | 8.623407 | 1.48E-08 | 3.08E-07 | Ighv1-52 |
| 10459576 | -1.14494 | 7.218633 | 8.618441 | 1.50E-08 | 3.10E-07 | Cep76 |
| 10526520 | 1.088613 | 7.131369 | -8.60466 | 1.54E-08 | 3.19E-07 | Plod3 |
| 10402864 | -1.69218 | 6.061167 | 8.602032 | 1.55E-08 | 3.20E-07 | Igh-VX24 |
| 10419216 | 1.252983 | 7.235526 | -8.59983 | 1.56E-08 | 3.21E-07 | Gnpnat1 |
| 10579799 | 1.036218 | 6.280723 | -8.59404 | 1.57E-08 | 3.25E-07 | Tmem184c |
| 10495186 | -1.77186 | 6.955253 | 8.589613 | 1.59E-08 | 3.27E-07 | AI504432 |
| 10458583 | 1.240659 | 8.311556 | -8.58818 | 1.59E-08 | 3.27E-07 | Yipf5 |
| 10496023 | 1.054546 | 5.59764 | -8.58742 | 1.60E-08 | 3.27E-07 | Casp6 |
| 10525365 | -1.05496 | 6.13926 | 8.563923 | 1.67E-08 | 3.42E-07 | Hvcn1 |
| 10408094 | -1.03038 | 11.39674 | 8.56221 | 1.68E-08 | 3.43E-07 | Hist1h2ao |
| 10489127 | -1.00151 | 7.158116 | 8.560423 | 1.68E-08 | 3.43E-07 | Rbl1 |
| 10374106 | 1.085152 | 6.892859 | -8.55323 | 1.71E-08 | 3.48E-07 | Ykt6 |
| 10503709 | -1.26786 | 5.051123 | 8.551299 | 1.72E-08 | 3.48E-07 | D130062J21Rik |
| 10513608 | -1.68987 | 8.271135 | 8.550582 | 1.72E-08 | 3.49E-07 | Alad |
| 10470462 | 1.489599 | 6.732741 | -8.54734 | 1.73E-08 | 3.50E-07 | Col5a1 |
| 10548385 | -1.5151 | 5.970779 | 8.546061 | 1.73E-08 | 3.51E-07 | Olr1 |
| 10551666 | -1.42104 | 6.16433 | 8.543421 | 1.74E-08 | 3.52E-07 | Map4k1 |
| 10569569 | 1.035172 | 7.239494 | -8.54069 | 1.75E-08 | 3.54E-07 | Cttn |
| 10360764 | 1.397706 | 7.498526 | -8.53521 | 1.77E-08 | 3.57E-07 | Enah |
| 10481518 | 1.031675 | 6.350207 | -8.53369 | 1.78E-08 | 3.58E-07 | Ptges |
| 10430596 | -1.08666 | 7.003859 | 8.52671 | 1.80E-08 | 3.62E-07 | Sun2 |
| 10592618 | -1.1323 | 7.364631 | 8.525501 | 1.81E-08 | 3.62E-07 | Tbcel |
| 10448506 | -1.29719 | 6.777042 | 8.524371 | 1.81E-08 | 3.63E-07 | Ccnf |
| 10361807 | 1.006074 | 6.729016 | -8.51737 | 1.84E-08 | 3.67E-07 | Hivep2 |
| 10405179 | 1.251064 | 7.00476 | -8.50884 | 1.87E-08 | 3.72E-07 | S1pr3 |
| 10600500 | -1.19509 | 7.990309 | 8.507682 | 1.87E-08 | 3.73E-07 | Fam220a |
| 10375079 | 1.023184 | 7.191753 | -8.50751 | 1.88E-08 | 3.73E-07 | Ubtd2 |
| 10567049 | 1.126094 | 8.993709 | -8.50619 | 1.88E-08 | 3.73E-07 | Copb1 |
| 10416653 | -1.0697 | 6.594434 | 8.499631 | 1.91E-08 | 3.77E-07 | Kbtbd7 |
| 10391831 | 1.549756 | 7.585831 | -8.48922 | 1.95E-08 | 3.83E-07 | Dcakd |
| 10603896 | 1.345009 | 5.940441 | -8.48864 | 1.95E-08 | 3.84E-07 | Klhl13 |
| 10347931 | -1.033 | 10.8585 | 8.487002 | 1.96E-08 | 3.85E-07 | G530012D18Rik |
| 10399924 | -1.07089 | 7.488795 | 8.486505 | 1.96E-08 | 3.85E-07 | Pik3cg |
| 10403060 | -2.34581 | 8.306677 | 8.484759 | 1.96E-08 | 3.86E-07 | Igh-VJ558 |
| 10483865 | 2.291745 | 8.403056 | -8.4779 | 1.99E-08 | 3.91E-07 | Fkbp7 |
| 10368577 | 1.054036 | 6.657675 | -8.47736 | 1.99E-08 | 3.91E-07 | Rnf217 |
| 10448117 | 1.061647 | 5.446206 | -8.47417 | 2.01E-08 | 3.92E-07 | Has1 |
| 10525236 | -1.49317 | 7.767867 | 8.471249 | 2.02E-08 | 3.94E-07 | Gm15800 |
| 10351491 | 1.648206 | 7.599848 | -8.46813 | 2.03E-08 | 3.96E-07 | Olfml2b |
| 10357875 | -1.38174 | 8.257566 | 8.462188 | 2.06E-08 | 4.01E-07 | Btg2 |
| 10519324 | -1.06611 | 8.032058 | 8.459513 | 2.07E-08 | 4.02E-07 | Cdk6 |
| 10440393 | -1.02523 | 7.383021 | 8.455409 | 2.08E-08 | 4.04E-07 | Samsn1 |
| 10531146 | -1.0716 | 5.239475 | 8.453091 | 2.09E-08 | 4.06E-07 | Mkrn1-ps1 |
| 10587829 | 2.673928 | 7.504924 | -8.45187 | 2.10E-08 | 4.06E-07 | Plod2 |
| 10530592 | -1.12887 | 8.076642 | 8.445962 | 2.13E-08 | 4.10E-07 | Fryl |
| 10587880 | 1.396374 | 6.691587 | -8.44221 | 2.14E-08 | 4.13E-07 | Pcolce2 |
| 10523012 | -1.25768 | 7.600802 | 8.422107 | 2.23E-08 | 4.29E-07 | Dck |
| 10348166 | 1.522566 | 5.864325 | -8.41895 | 2.25E-08 | 4.31E-07 | Chrng |
| 10486396 | 1.095202 | 7.605223 | -8.41147 | 2.28E-08 | 4.37E-07 | Ehd4 |
| 10448124 | -1.87709 | 7.756972 | 8.410915 | 2.28E-08 | 4.37E-07 | Fpr1 |
| 10525381 | 1.022199 | 8.697209 | -8.40964 | 2.29E-08 | 4.38E-07 | Vps29 |
| 10505931 | 1.278084 | 5.541721 | -8.40918 | 2.29E-08 | 4.38E-07 | Ift74 |
| 10372021 | -1.17893 | 4.466934 | 8.406604 | 2.30E-08 | 4.39E-07 | Gm26122 |
| 10579609 | -1.35734 | 5.443939 | 8.406107 | 2.31E-08 | 4.40E-07 | Fcho1 |
| 10390299 | -1.20767 | 6.804695 | 8.391753 | 2.37E-08 | 4.51E-07 | Pnpo |
| 10546113 | 1.3325 | 8.947851 | -8.38918 | 2.39E-08 | 4.53E-07 | Sec61a1 |
| 10526502 | 1.088292 | 6.266424 | -8.38348 | 2.41E-08 | 4.56E-07 | Ift22 |
| 10396278 | -1.04691 | 7.48495 | 8.377395 | 2.44E-08 | 4.61E-07 | Daam1 |
| 10475378 | 1.199281 | 6.541941 | -8.37651 | 2.45E-08 | 4.62E-07 | Casc4 |
| 10388880 | 1.561409 | 7.397795 | -8.37622 | 2.45E-08 | 4.62E-07 | Tmem97 |
| 10409464 | 1.02389 | 6.342468 | -8.37194 | 2.47E-08 | 4.65E-07 | Dbn1 |
| 10513256 | 1.217326 | 7.457509 | -8.37189 | 2.47E-08 | 4.65E-07 | Lpar1 |
| 10382435 | 1.302955 | 6.838134 | -8.37027 | 2.48E-08 | 4.66E-07 | Gprc5c |
| 10505143 | 1.212123 | 5.406438 | -8.36816 | 2.49E-08 | 4.67E-07 | Akap2 |
| 10373768 | 1.749306 | 8.95776 | -8.36578 | 2.50E-08 | 4.69E-07 | Selm |
| 10566580 | -1.6281 | 5.103777 | 8.364013 | 2.51E-08 | 4.71E-07 | Gm4759 |
| 10513739 | 1.618864 | 9.693591 | -8.35635 | 2.55E-08 | 4.77E-07 | Tnc |
| 10408689 | 1.74174 | 5.181248 | -8.34961 | 2.59E-08 | 4.83E-07 | Nrn1 |
| 10586591 | 2.02932 | 5.736578 | -8.34862 | 2.59E-08 | 4.84E-07 | Car12 |
| 10526559 | -1.94184 | 6.58173 | 8.345572 | 2.61E-08 | 4.86E-07 | Ache |
| 10364529 | -2.46027 | 8.445355 | 8.344933 | 2.61E-08 | 4.87E-07 | Prtn3 |
| 10531126 | -3.46964 | 7.400711 | 8.343921 | 2.62E-08 | 4.87E-07 | Igj |
| 10383556 | -1.12756 | 7.018774 | 8.335799 | 2.66E-08 | 4.95E-07 | Fn3krp |
| 10532741 | 1.737863 | 7.70311 | -8.33206 | 2.68E-08 | 4.99E-07 | Tmem119 |
| 10405211 | 1.171737 | 5.99198 | -8.33195 | 2.68E-08 | 4.99E-07 | Gadd45g |
| 10438415 | -2.04676 | 5.730465 | 8.329428 | 2.70E-08 | 5.00E-07 | Iglv2 |
| 10482500 | 1.061107 | 7.996693 | -8.32761 | 2.71E-08 | 5.02E-07 | Rnd3 |
| 10408111 | -1.00786 | 11.49101 | 8.326668 | 2.71E-08 | 5.02E-07 | Hist1h2ao |
| 10554789 | 1.271845 | 9.232021 | -8.32459 | 2.72E-08 | 5.04E-07 | Ctsc |
| 10357115 | 1.699605 | 5.699262 | -8.32302 | 2.73E-08 | 5.05E-07 | Dsel |
| 10450424 | -1.12942 | 5.325901 | 8.322737 | 2.73E-08 | 5.05E-07 | Ly6g6f |
| 10404026 | -1.00351 | 11.44999 | 8.318197 | 2.76E-08 | 5.09E-07 | Hist1h2ao |
| 10364650 | -1.35612 | 7.819178 | 8.317283 | 2.77E-08 | 5.09E-07 | Hmha1 |
| 10450025 | -1.34015 | 8.54282 | 8.315196 | 2.78E-08 | 5.11E-07 | 44257 |
| 10408085 | -1.05225 | 11.33748 | 8.314785 | 2.78E-08 | 5.11E-07 | Hist1h2ao |
| 10547869 | 1.163399 | 6.586875 | -8.313 | 2.79E-08 | 5.12E-07 | Leprel2 |
| 10546454 | 1.308162 | 6.012159 | -8.30941 | 2.81E-08 | 5.15E-07 | Adamts9 |
| 10489891 | 1.209831 | 7.168885 | -8.30555 | 2.83E-08 | 5.18E-07 | B4galt5 |
| 10501020 | -1.88369 | 10.52049 | 8.302445 | 2.85E-08 | 5.21E-07 | Chil3 |
| 10403955 | -1.0091 | 11.49446 | 8.300371 | 2.86E-08 | 5.22E-07 | Hist1h2ao |
| 10501164 | 1.006937 | 7.245666 | -8.30005 | 2.87E-08 | 5.22E-07 | Csf1 |
| 10471953 | 1.00689 | 7.080103 | -8.29305 | 2.91E-08 | 5.28E-07 | Acvr2a |
| 10404389 | -1.22094 | 4.792301 | 8.285898 | 2.95E-08 | 5.34E-07 | Irf4 |
| 10585276 | -1.68935 | 6.583341 | 8.285887 | 2.95E-08 | 5.34E-07 | Pou2af1 |
| 10445688 | -1.13187 | 8.308509 | 8.281808 | 2.97E-08 | 5.37E-07 | Ccnd3 |
| 10463123 | -1.57543 | 4.938106 | 8.280176 | 2.98E-08 | 5.38E-07 | Dntt |
| 10554926 | 1.188515 | 7.475455 | -8.27699 | 3.00E-08 | 5.40E-07 | Ccdc90b |
| 10387821 | -2.75776 | 7.503827 | 8.276757 | 3.01E-08 | 5.40E-07 | Alox12 |
| 10552406 | -2.86025 | 7.57533 | 8.276407 | 3.01E-08 | 5.40E-07 | Nkg7 |
| 10381445 | 1.316894 | 7.364667 | -8.27129 | 3.04E-08 | 5.45E-07 | Tmem106a |
| 10360382 | 1.317266 | 7.923672 | -8.25642 | 3.13E-08 | 5.60E-07 | Ifi204 |
| 10601412 | 1.615185 | 5.84231 | -8.2507 | 3.17E-08 | 5.66E-07 | Lpar4 |
| 10530563 | -1.08754 | 7.966426 | 8.246693 | 3.20E-08 | 5.69E-07 | Fryl |
| 10466172 | -2.33759 | 6.593851 | 8.246103 | 3.20E-08 | 5.70E-07 | Ms4a1 |
| 10352178 | 1.352963 | 7.166722 | -8.24483 | 3.21E-08 | 5.70E-07 | Sccpdh |
| 10544638 | -1.1634 | 7.8785 | 8.241776 | 3.23E-08 | 5.73E-07 | Tra2a |
| 10569344 | 1.530661 | 7.057963 | -8.24159 | 3.23E-08 | 5.73E-07 | Igf2 |
| 10544982 | -1.55047 | 8.008475 | 8.238715 | 3.25E-08 | 5.76E-07 | Nt5c3 |
| 10571162 | 1.162647 | 8.226502 | -8.23557 | 3.27E-08 | 5.79E-07 | Eif4ebp1 |
| 10460891 | -1.65564 | 7.040375 | 8.231707 | 3.30E-08 | 5.83E-07 | Map4k2 |
| 10412207 | 1.916533 | 8.596682 | -8.23002 | 3.31E-08 | 5.84E-07 | Gpx8 |
| 10512226 | -1.07396 | 8.835077 | 8.227715 | 3.33E-08 | 5.86E-07 | Dcaf12 |
| 10422493 | -1.08113 | 6.663225 | 8.224857 | 3.35E-08 | 5.89E-07 | Gpr18 |
| 10530319 | -1.49265 | 8.291927 | 8.213958 | 3.42E-08 | 5.99E-07 | Atp8a1 |
| 10563099 | -1.75062 | 5.666843 | 8.204511 | 3.49E-08 | 6.11E-07 | Snord35b |
| 10371332 | 1.712457 | 5.82587 | -8.20318 | 3.50E-08 | 6.12E-07 | Aldh1l2 |
| 10493259 | 1.086328 | 9.496653 | -8.20186 | 3.51E-08 | 6.13E-07 | Ssr2 |
| 10596303 | -1.07865 | 5.475033 | 8.197889 | 3.54E-08 | 6.17E-07 | Acpp |
| 10587383 | 1.985715 | 6.831018 | -8.19763 | 3.54E-08 | 6.17E-07 | Cd109 |
| 10461622 | 1.793999 | 8.679651 | -8.19753 | 3.54E-08 | 6.17E-07 | Ms4a6b |
| 10362899 | -1.23621 | 7.199575 | 8.188042 | 3.61E-08 | 6.29E-07 | F830002L21Rik |
| 10379489 | 1.282477 | 5.431231 | -8.18744 | 3.61E-08 | 6.29E-07 | Tmem98 |
| 10463355 | 1.747372 | 7.163547 | -8.18471 | 3.64E-08 | 6.32E-07 | Scd2 |
| 10408118 | -1.01118 | 11.38676 | 8.180021 | 3.67E-08 | 6.37E-07 | Hist1h2ao |
| 10478692 | 1.124842 | 5.832028 | -8.17316 | 3.72E-08 | 6.45E-07 | Slc2a10 |
| 10390640 | -1.79262 | 5.279309 | 8.170816 | 3.74E-08 | 6.48E-07 | Ikzf3 |
| 10494395 | -1.00319 | 10.94212 | 8.166468 | 3.78E-08 | 6.53E-07 | Hist2h2aa1 |
| 10347291 | -1.61457 | 9.252669 | 8.160674 | 3.82E-08 | 6.60E-07 | Cxcr2 |
| 10362974 | -1.11643 | 6.815798 | 8.147879 | 3.92E-08 | 6.74E-07 | Hace1 |
| 10355567 | 1.049666 | 8.025874 | -8.13601 | 4.02E-08 | 6.88E-07 | Tmbim1 |
| 10498379 | 1.762538 | 7.3271 | -8.13486 | 4.03E-08 | 6.89E-07 | Igsf10 |
| 10517508 | 1.981756 | 8.570195 | -8.1278 | 4.09E-08 | 6.98E-07 | C1qb |
| 10571467 | 2.042048 | 6.379885 | -8.1256 | 4.11E-08 | 7.01E-07 | Pdgfrl |
| 10377148 | 1.631033 | 6.2285 | -8.12507 | 4.11E-08 | 7.01E-07 | Myh8 |
| 10538921 | -3.65876 | 7.714859 | 8.119334 | 4.16E-08 | 7.09E-07 | Igkv1-117 |
| 10365471 | -1.07245 | 6.767874 | 8.112573 | 4.22E-08 | 7.17E-07 | Fbxo7 |
| 10568536 | 1.69437 | 6.256192 | -8.10943 | 4.25E-08 | 7.22E-07 | Cpxm2 |
| 10381898 | 1.279942 | 6.825079 | -8.1091 | 4.25E-08 | 7.22E-07 | Mrc2 |
| 10599581 | -1.47805 | 7.038801 | 8.10694 | 4.27E-08 | 7.25E-07 | 2610018G03Rik |
| 10475643 | 1.235308 | 7.294375 | -8.10357 | 4.30E-08 | 7.29E-07 | Fgf7 |
| 10376444 | -1.3767 | 6.395605 | 8.09108 | 4.42E-08 | 7.45E-07 | Hist3h2ba |
| 10372342 | 1.020766 | 5.826312 | -8.08907 | 4.43E-08 | 7.47E-07 | Nav3 |
| 10473367 | -1.80472 | 8.598331 | 8.079016 | 4.53E-08 | 7.60E-07 | Slc43a1 |
| 10574471 | 1.30331 | 8.512561 | -8.07858 | 4.53E-08 | 7.60E-07 | Cmtm3 |
| 10478962 | -1.45091 | 7.326416 | 8.078213 | 4.54E-08 | 7.60E-07 | Fam210b |
| 10357239 | 1.363138 | 6.723847 | -8.07052 | 4.61E-08 | 7.71E-07 | Tmem37 |
| 10601846 | 1.251994 | 5.884463 | -8.06348 | 4.68E-08 | 7.81E-07 | Arxes2 |
| 10382328 | 1.226307 | 6.49465 | -8.06046 | 4.71E-08 | 7.85E-07 | Sox9 |
| 10354432 | 1.429564 | 7.25011 | -8.05853 | 4.73E-08 | 7.87E-07 | Myo1b |
| 10451993 | 1.34214 | 7.461055 | -8.0558 | 4.75E-08 | 7.90E-07 | D17Wsu104e |
| 10561920 | -1.32822 | 8.382145 | 8.054996 | 4.76E-08 | 7.90E-07 | Hcst |
| 10565315 | 1.010671 | 5.805827 | -8.05355 | 4.78E-08 | 7.92E-07 | Fah |
| 10492165 | 1.561121 | 4.927775 | -8.05307 | 4.78E-08 | 7.92E-07 | Gm25132 |
| 10412559 | -1.36292 | 7.327139 | 8.043478 | 4.88E-08 | 8.07E-07 | Slbp |
| 10476538 | 1.088331 | 6.501254 | -8.02642 | 5.05E-08 | 8.32E-07 | Btbd3 |
| 10533345 | -1.01333 | 8.151076 | 8.019801 | 5.13E-08 | 8.42E-07 | Aldh2 |
| 10539818 | -2.59317 | 7.577851 | 8.01653 | 5.16E-08 | 8.47E-07 | Gp9 |
| 10529299 | -1.0361 | 8.031783 | 8.003959 | 5.30E-08 | 8.65E-07 | Slbp |
| 10492330 | -1.31993 | 6.293312 | 8.001897 | 5.32E-08 | 8.68E-07 | P2ry1 |
| 10361509 | -1.13307 | 8.163263 | 7.997143 | 5.37E-08 | 8.75E-07 | Syne1 |
| 10402991 | -1.63033 | 6.825215 | 7.983528 | 5.53E-08 | 8.98E-07 | Ighv2-4 |
| 10370339 | -1.2958 | 5.240954 | 7.978563 | 5.59E-08 | 9.06E-07 | Trpm2 |
| 10516765 | 1.36945 | 7.271774 | -7.97712 | 5.61E-08 | 9.07E-07 | Serinc2 |
| 10403069 | -2.48933 | 4.469695 | 7.971487 | 5.67E-08 | 9.15E-07 | Igh-VJ558 |
| 10438445 | -1.26857 | 7.48969 | 7.97106 | 5.68E-08 | 9.15E-07 | Klhl6 |
| 10459335 | 1.23249 | 5.025838 | -7.96624 | 5.73E-08 | 9.23E-07 | Piezo2 |
| 10530783 | -1.44773 | 5.967607 | 7.965429 | 5.74E-08 | 9.24E-07 | A730089K16Rik |
| 10388160 | 2.340849 | 6.631542 | -7.96437 | 5.76E-08 | 9.25E-07 | Slc13a5 |
| 10472538 | -1.1926 | 6.221316 | 7.954735 | 5.88E-08 | 9.42E-07 | Dhrs9 |
| 10545014 | -1.23723 | 7.40226 | 7.952515 | 5.90E-08 | 9.44E-07 | Vopp1 |
| 10379633 | -1.26115 | 8.682431 | 7.951808 | 5.91E-08 | 9.45E-07 | Slfn1 |
| 10403021 | -2.03255 | 5.241555 | 7.950006 | 5.93E-08 | 9.47E-07 | Ighv1-42 |
| 10571530 | 1.222838 | 7.181285 | -7.94767 | 5.96E-08 | 9.50E-07 | Fat1 |
| 10559478 | -1.43096 | 6.900094 | 7.946659 | 5.98E-08 | 9.52E-07 | Lilra6 |
| 10407803 | 1.299002 | 7.062271 | -7.94393 | 6.01E-08 | 9.56E-07 | Gpr137b |
| 10382425 | 1.049632 | 6.635464 | -7.93953 | 6.07E-08 | 9.64E-07 | Gprc5c |
| 10479154 | -2.06553 | 6.797584 | 7.93537 | 6.12E-08 | 9.69E-07 | Tubb1 |
| 10558811 | -1.11725 | 7.498464 | 7.932676 | 6.15E-08 | 9.74E-07 | Ptdss2 |
| 10436519 | 1.319362 | 6.137833 | -7.93167 | 6.17E-08 | 9.76E-07 | Robo1 |
| 10429520 | -2.26357 | 6.988253 | 7.928502 | 6.21E-08 | 9.81E-07 | Ly6d |
| 10590628 | -1.55233 | 5.718565 | 7.922632 | 6.29E-08 | 9.91E-07 | Ccr3 |
| 10440993 | 1.048708 | 7.594504 | -7.9143 | 6.40E-08 | 1.01E-06 | Rcan1 |
| 10546184 | 1.060828 | 7.499363 | -7.91134 | 6.44E-08 | 1.01E-06 | Plxna1 |
| 10449893 | -1.16544 | 6.13498 | 7.909232 | 6.47E-08 | 1.01E-06 | Rasal3 |
| 10350247 | -1.30007 | 6.264856 | 7.899494 | 6.60E-08 | 1.03E-06 | Kif21b |
| 10424543 | 1.475696 | 6.916009 | -7.89725 | 6.63E-08 | 1.04E-06 | Wisp1 |
| 10485198 | 1.059667 | 5.61412 | -7.89525 | 6.66E-08 | 1.04E-06 | Tspan18 |
| 10545096 | -1.47539 | 5.73411 | 7.889621 | 6.74E-08 | 1.05E-06 | Mageb16 |
| 10531415 | 1.892644 | 6.566911 | -7.88338 | 6.83E-08 | 1.06E-06 | Cxcl10 |
| 10345824 | -1.25824 | 6.948981 | 7.882861 | 6.84E-08 | 1.06E-06 | Il18rap |
| 10532839 | 1.185291 | 5.760229 | -7.88256 | 6.84E-08 | 1.06E-06 | Trpv4 |
| 10592772 | -1.41243 | 6.854026 | 7.878973 | 6.89E-08 | 1.07E-06 | Abcg4 |
| 10605542 | -1.02292 | 4.99 | 7.875944 | 6.94E-08 | 1.07E-06 | Mageb16 |
| 10450484 | 1.428816 | 7.471541 | -7.87443 | 6.96E-08 | 1.08E-06 | Aif1 |
| 10492682 | 1.304461 | 6.43678 | -7.87212 | 6.99E-08 | 1.08E-06 | Fam198b |
| 10567580 | -1.0635 | 8.56477 | 7.87023 | 7.02E-08 | 1.08E-06 | Igsf6 |
| 10433274 | 1.029665 | 7.895973 | -7.86957 | 7.03E-08 | 1.08E-06 | Vasn |
| 10565794 | 1.809593 | 8.455731 | -7.8623 | 7.14E-08 | 1.10E-06 | Serpinh1 |
| 10392815 | 1.11269 | 8.178871 | -7.85974 | 7.18E-08 | 1.10E-06 | AF251705 |
| 10422052 | 1.094179 | 7.290771 | -7.85627 | 7.23E-08 | 1.11E-06 | Commd6 |
| 10517517 | 2.084966 | 8.727996 | -7.85014 | 7.33E-08 | 1.12E-06 | C1qa |
| 10570291 | -1.10362 | 6.790486 | 7.839287 | 7.50E-08 | 1.14E-06 | F10 |
| 10494386 | -1.0352 | 11.0296 | 7.835163 | 7.56E-08 | 1.15E-06 | Hist2h2ab |
| 10526181 | 1.253127 | 6.692698 | -7.83342 | 7.59E-08 | 1.15E-06 | Gatsl2 |
| 10453260 | -1.05339 | 5.566914 | 7.830685 | 7.63E-08 | 1.16E-06 | Haao |
| 10515755 | -1.83084 | 6.356315 | 7.8253 | 7.72E-08 | 1.17E-06 | Mpl |
| 10538811 | 1.264513 | 6.059821 | -7.82228 | 7.77E-08 | 1.17E-06 | Prdm5 |
| 10534679 | -1.14705 | 8.727452 | 7.819029 | 7.83E-08 | 1.18E-06 | Trim56 |
| 10474984 | -1.37469 | 8.484747 | 7.80732 | 8.02E-08 | 1.20E-06 | Nusap1 |
| 10534974 | -1.15404 | 7.888367 | 7.806423 | 8.04E-08 | 1.20E-06 | Mcm7 |
| 10376813 | -1.1104 | 7.850219 | 7.795693 | 8.22E-08 | 1.23E-06 | Specc1 |
| 10467470 | 1.088527 | 7.374591 | -7.78743 | 8.37E-08 | 1.25E-06 | Aldh18a1 |
| 10487945 | -1.02397 | 9.219283 | 7.783067 | 8.45E-08 | 1.26E-06 | Gpcpd1 |
| 10425037 | -2.13644 | 7.880742 | 7.774426 | 8.60E-08 | 1.28E-06 | Apol10a |
| 10459620 | -1.28913 | 6.226282 | 7.773793 | 8.61E-08 | 1.28E-06 | Rab27b |
| 10451551 | 1.063417 | 6.277557 | -7.77331 | 8.62E-08 | 1.28E-06 | Guca1a |
| 10512308 | 1.357708 | 6.757201 | -7.77085 | 8.67E-08 | 1.29E-06 | Sigmar1 |
| 10354267 | -1.97656 | 6.071121 | 7.765192 | 8.77E-08 | 1.30E-06 | Mettl21c |
| 10507131 | -1.10457 | 6.971934 | 7.763918 | 8.80E-08 | 1.30E-06 | Tal1 |
| 10548857 | -1.21634 | 8.548884 | 7.763708 | 8.80E-08 | 1.30E-06 | Hist4h4 |
| 10458999 | 1.508618 | 5.642253 | -7.7617 | 8.84E-08 | 1.30E-06 | Fbn2 |
| 10602020 | 1.351834 | 6.521148 | -7.76079 | 8.86E-08 | 1.30E-06 | Tbc1d8b |
| 10520965 | 1.223692 | 5.984842 | -7.7602 | 8.87E-08 | 1.31E-06 | Yes1 |
| 10404069 | -1.59678 | 9.171766 | 7.759209 | 8.89E-08 | 1.31E-06 | Hist1h1a |
| 10494407 | -1.48467 | 7.971251 | 7.745539 | 9.15E-08 | 1.34E-06 | Hist2h2bb |
| 10481627 | -1.9759 | 11.24117 | 7.738794 | 9.28E-08 | 1.36E-06 | Lcn2 |
| 10385826 | -1.59436 | 5.642416 | 7.73525 | 9.35E-08 | 1.36E-06 | Sowaha |
| 10384154 | -1.06483 | 7.326941 | 7.734899 | 9.36E-08 | 1.36E-06 | Myo1g |
| 10601844 | 1.144808 | 5.908723 | -7.733 | 9.40E-08 | 1.37E-06 | Bhlhb9 |
| 10444821 | -1.06856 | 5.918984 | 7.732904 | 9.40E-08 | 1.37E-06 | H2-Q5 |
| 10546725 | 1.068658 | 5.531837 | -7.72895 | 9.48E-08 | 1.38E-06 | Pdzrn3 |
| 10589535 | -1.69888 | 11.22845 | 7.728129 | 9.49E-08 | 1.38E-06 | Ngp |
| 10575799 | -1.12637 | 7.076421 | 7.722562 | 9.61E-08 | 1.39E-06 | Plcg2 |
| 10434668 | 1.741617 | 7.213113 | -7.71929 | 9.68E-08 | 1.40E-06 | Tmem97 |
| 10403048 | -2.58815 | 8.825734 | 7.715165 | 9.76E-08 | 1.41E-06 | Ighv1-62-3 |
| 10344966 | 1.105109 | 5.396971 | -7.71503 | 9.76E-08 | 1.41E-06 | Ly96 |
| 10407072 | -1.27008 | 5.370972 | 7.70611 | 9.95E-08 | 1.44E-06 | Elovl7 |
| 10559606 | -1.46535 | 5.8973 | 7.693239 | 1.02E-07 | 1.47E-06 | Tmem86b |
| 10456046 | 1.009038 | 6.913176 | -7.68372 | 1.04E-07 | 1.50E-06 | Pdgfrb |
| 10597098 | -1.78889 | 11.7024 | 7.679569 | 1.05E-07 | 1.51E-06 | Camp |
| 10498296 | 1.009981 | 7.661439 | -7.67046 | 1.07E-07 | 1.53E-06 | Commd2 |
| 10552697 | -1.35694 | 6.659225 | 7.665095 | 1.09E-07 | 1.55E-06 | Napsa |
| 10391697 | -1.95021 | 6.851863 | 7.650123 | 1.12E-07 | 1.59E-06 | Itga2b |
| 10517116 | -1.32344 | 7.69393 | 7.646799 | 1.13E-07 | 1.60E-06 | Rps6ka1 |
| 10595560 | 1.03235 | 5.261975 | -7.63535 | 1.16E-07 | 1.64E-06 | Tbx18 |
| 10538706 | -1.82712 | 6.219114 | 7.632673 | 1.16E-07 | 1.64E-06 | Mmrn1 |
| 10403015 | -2.52939 | 6.671091 | 7.622212 | 1.19E-07 | 1.67E-06 | Ighv1-18 |
| 10545707 | 1.264341 | 5.886246 | -7.6015 | 1.25E-07 | 1.74E-06 | Actg2 |
| 10475437 | -1.36901 | 8.361802 | 7.596685 | 1.26E-07 | 1.76E-06 | Sord |
| 10585390 | 1.269978 | 6.264068 | -7.59452 | 1.26E-07 | 1.76E-06 | Sln |
| 10501860 | 1.029197 | 7.517196 | -7.58625 | 1.29E-07 | 1.79E-06 | Fnbp1l |
| 10384458 | -1.27529 | 9.170963 | 7.585851 | 1.29E-07 | 1.79E-06 | Plek |
| 10420877 | -1.20561 | 7.067223 | 7.582113 | 1.30E-07 | 1.80E-06 | Esco2 |
| 10347948 | -1.07014 | 8.544745 | 7.581694 | 1.30E-07 | 1.80E-06 | Sp100 |
| 10478355 | -1.00169 | 7.28979 | 7.58089 | 1.30E-07 | 1.80E-06 | Mybl2 |
| 10569848 | -1.04233 | 8.055594 | 7.578096 | 1.31E-07 | 1.81E-06 | Stxbp2 |
| 10504755 | -2.19326 | 7.01362 | 7.577973 | 1.31E-07 | 1.81E-06 | Sympk |
| 10511258 | -1.04355 | 7.815828 | 7.56772 | 1.34E-07 | 1.85E-06 | Fam132a |
| 10364262 | -1.094 | 8.999291 | 7.565604 | 1.35E-07 | 1.85E-06 | Itgb2 |
| 10383010 | 1.022831 | 6.607514 | -7.56434 | 1.35E-07 | 1.86E-06 | Socs3 |
| 10429856 | -1.6599 | 7.704061 | 7.563704 | 1.35E-07 | 1.86E-06 | Gm10872 |
| 10406877 | 1.31304 | 6.529998 | -7.56099 | 1.36E-07 | 1.87E-06 | Serf1 |
| 10354741 | 1.09426 | 5.852651 | -7.55156 | 1.39E-07 | 1.90E-06 | Rftn2 |
| 10448278 | -1.92056 | 6.263712 | 7.550308 | 1.39E-07 | 1.91E-06 | Mmp25 |
| 10563338 | -1.88769 | 7.708696 | 7.549681 | 1.39E-07 | 1.91E-06 | Ppp1r15a |
| 10542181 | -1.47915 | 5.878846 | 7.545753 | 1.40E-07 | 1.92E-06 | Clec9a |
| 10401238 | 1.013693 | 8.534788 | -7.54565 | 1.40E-07 | 1.92E-06 | Zfp36l1 |
| 10384956 | -1.10596 | 7.472912 | 7.540815 | 1.42E-07 | 1.94E-06 | Chac2 |
| 10571274 | -1.00449 | 9.575814 | 7.540351 | 1.42E-07 | 1.94E-06 | Gsr |
| 10557591 | -1.53276 | 6.686531 | 7.533222 | 1.44E-07 | 1.97E-06 | Itgal |
| 10495651 | 1.00154 | 6.689281 | -7.52548 | 1.47E-07 | 1.99E-06 | Alg14 |
| 10475567 | -1.10106 | 6.414097 | 7.520643 | 1.48E-07 | 2.01E-06 | Slc24a5 |
| 10501222 | 1.164379 | 7.591145 | -7.51733 | 1.49E-07 | 2.02E-06 | Gstm2 |
| 10513666 | -1.41988 | 7.410226 | 7.515774 | 1.50E-07 | 2.03E-06 | Akna |
| 10439766 | 1.029448 | 6.180844 | -7.50701 | 1.53E-07 | 2.06E-06 | Pvrl3 |
| 10382376 | 1.026903 | 6.65414 | -7.49431 | 1.57E-07 | 2.11E-06 | Ttyh2 |
| 10498386 | 1.554534 | 7.216682 | -7.48185 | 1.61E-07 | 2.16E-06 | Igsf10 |
| 10539702 | 1.113422 | 6.419044 | -7.47474 | 1.64E-07 | 2.19E-06 | Fam136a |
| 10381708 | -1.07652 | 6.872918 | 7.472764 | 1.64E-07 | 2.20E-06 | Fmnl1 |
| 10526191 | 1.302417 | 6.583329 | -7.45927 | 1.69E-07 | 2.25E-06 | Gatsl2 |
| 10430818 | -1.11223 | 6.902811 | 7.457325 | 1.70E-07 | 2.26E-06 | Tnfrsf13c |
| 10563178 | -1.28702 | 7.758151 | 7.45484 | 1.71E-07 | 2.27E-06 | Cd37 |
| 10523151 | 1.395424 | 5.944344 | -7.43434 | 1.79E-07 | 2.35E-06 | Cxcl1 |
| 10359929 | 1.034159 | 7.076216 | -7.41865 | 1.85E-07 | 2.42E-06 | Ddr2 |
| 10596815 | -1.47301 | 7.969195 | 7.411401 | 1.88E-07 | 2.45E-06 | Rnf123 |
| 10419223 | 1.225512 | 7.569957 | -7.40636 | 1.90E-07 | 2.48E-06 | Fermt2 |
| 10408077 | -1.23093 | 8.413612 | 7.403819 | 1.91E-07 | 2.49E-06 | Hist1h2ak |
| 10368720 | -1.40432 | 7.597708 | 7.399045 | 1.93E-07 | 2.51E-06 | Slc16a10 |
| 10359307 | 2.227276 | 6.661133 | -7.38018 | 2.01E-07 | 2.61E-06 | Tnn |
| 10358894 | -1.4214 | 8.382745 | 7.378243 | 2.02E-07 | 2.62E-06 | Sord |
| 10523701 | 1.778714 | 10.72691 | -7.37637 | 2.03E-07 | 2.63E-06 | Ibsp |
| 10411226 | 1.05117 | 4.994475 | -7.37606 | 2.03E-07 | 2.63E-06 | F2rl1 |
| 10579347 | 1.229236 | 8.601852 | -7.37563 | 2.03E-07 | 2.63E-06 | Ifi30 |
| 10345791 | 1.569972 | 6.710874 | -7.37532 | 2.03E-07 | 2.63E-06 | Il1rl1 |
| 10420891 | 1.039947 | 6.91674 | -7.36414 | 2.08E-07 | 2.69E-06 | Scara3 |
| 10594582 | -1.25893 | 5.199312 | 7.35937 | 2.10E-07 | 2.71E-06 | Snx22 |
| 10517513 | 1.505405 | 9.965045 | -7.35594 | 2.12E-07 | 2.73E-06 | C1qc |
| 10539135 | 1.033115 | 8.144887 | -7.35248 | 2.14E-07 | 2.74E-06 | Capg |
| 10606694 | -1.1843 | 7.016581 | 7.351859 | 2.14E-07 | 2.75E-06 | Btk |
| 10566583 | -1.23529 | 8.518948 | 7.351272 | 2.14E-07 | 2.75E-06 | Gm8995 |
| 10545974 | 1.348308 | 7.99523 | -7.3467 | 2.16E-07 | 2.77E-06 | Antxr1 |
| 10495794 | -1.36572 | 6.692442 | 7.340819 | 2.19E-07 | 2.80E-06 | Pde5a |
| 10599200 | 1.025083 | 7.183009 | -7.33824 | 2.20E-07 | 2.82E-06 | Pgrmc1 |
| 10581340 | -1.49777 | 8.017422 | 7.332133 | 2.23E-07 | 2.85E-06 | Ranbp10 |
| 10387536 | 1.516382 | 9.414025 | -7.32474 | 2.27E-07 | 2.89E-06 | Cd68 |
| 10346878 | 1.179993 | 4.104878 | -7.32084 | 2.29E-07 | 2.92E-06 | Zdbf2 |
| 10431711 | 1.011899 | 6.004125 | -7.31888 | 2.30E-07 | 2.93E-06 | Slc2a13 |
| 10425053 | -1.254 | 7.459956 | 7.317359 | 2.31E-07 | 2.93E-06 | Ncf4 |
| 10507840 | 1.04653 | 5.851025 | -7.31068 | 2.34E-07 | 2.97E-06 | Heyl |
| 10430645 | -1.04417 | 4.857106 | 7.306389 | 2.36E-07 | 2.99E-06 | D730005E14Rik |
| 10436304 | 1.215793 | 8.462616 | -7.30494 | 2.37E-07 | 3.00E-06 | Abi3bp |
| 10497381 | 1.083562 | 5.000017 | -7.30456 | 2.37E-07 | 3.00E-06 | Cyp7b1 |
| 10360985 | -1.08089 | 7.642039 | 7.303446 | 2.38E-07 | 3.00E-06 | Cenpf |
| 10403018 | -2.79501 | 6.890019 | 7.302762 | 2.38E-07 | 3.00E-06 | Igh-VJ558 |
| 10502191 | 1.203996 | 9.7807 | -7.30202 | 2.38E-07 | 3.01E-06 | Ostc |
| 10404063 | -2.21913 | 7.751937 | 7.299528 | 2.40E-07 | 3.02E-06 | Hist1h2ab |
| 10433480 | 1.926077 | 6.198653 | -7.29874 | 2.40E-07 | 3.02E-06 | Rpl39l |
| 10592420 | 1.039909 | 4.547171 | -7.29684 | 2.41E-07 | 3.04E-06 | AW551984 |
| 10443749 | -1.08178 | 5.117635 | 7.292601 | 2.43E-07 | 3.06E-06 | Ubash3a |
| 10432675 | -1.49256 | 6.128598 | 7.282643 | 2.49E-07 | 3.12E-06 | I730030J21Rik |
| 10514466 | 1.114215 | 7.79142 | -7.26942 | 2.56E-07 | 3.20E-06 | Jun |
| 10554249 | 2.553426 | 6.266842 | -7.25385 | 2.65E-07 | 3.30E-06 | Acan |
| 10607395 | 1.18247 | 5.891794 | -7.25279 | 2.66E-07 | 3.30E-06 | Mageh1 |
| 10403043 | -2.29656 | 7.652096 | 7.243981 | 2.71E-07 | 3.36E-06 | Ighv1-62-3 |
| 10420957 | -1.01725 | 7.104328 | 7.241149 | 2.73E-07 | 3.37E-06 | Ptk2b |
| 10427590 | 1.001359 | 5.6666 | -7.23972 | 2.73E-07 | 3.38E-06 | Slc1a3 |
| 10583163 | -1.434 | 5.920764 | 7.237763 | 2.75E-07 | 3.39E-06 | Trpc6 |
| 10403038 | -2.19307 | 5.854299 | 7.231883 | 2.78E-07 | 3.43E-06 | Ighv1-61 |
| 10446282 | 1.413467 | 7.9372 | -7.22777 | 2.81E-07 | 3.45E-06 | Emr1 |
| 10403743 | 1.245738 | 7.353257 | -7.22278 | 2.84E-07 | 3.48E-06 | Inhba |
| 10406982 | 1.035347 | 5.074825 | -7.2173 | 2.87E-07 | 3.52E-06 | Adamts6 |
| 10584710 | -1.43449 | 9.187096 | 7.215284 | 2.89E-07 | 3.53E-06 | H2afx |
| 10445758 | -1.61613 | 5.878991 | 7.210547 | 2.92E-07 | 3.56E-06 | Treml4 |
| 10551025 | -2.29303 | 5.748343 | 7.194947 | 3.02E-07 | 3.67E-06 | Cd79a |
| 10403054 | -1.87733 | 7.36195 | 7.19359 | 3.03E-07 | 3.67E-06 | Igh-VJ558 |
| 10606369 | 2.004889 | 8.469978 | -7.18253 | 3.10E-07 | 3.75E-06 | Itm2a |
| 10345921 | 1.80992 | 8.90515 | -7.1823 | 3.10E-07 | 3.75E-06 | 1500015O10Rik |
| 10494271 | 1.072281 | 9.99884 | -7.179 | 3.13E-07 | 3.78E-06 | Ctss |
| 10529034 | 1.989212 | 7.211218 | -7.17679 | 3.14E-07 | 3.79E-06 | Cgref1 |
| 10458278 | -1.71682 | 5.487712 | 7.175891 | 3.15E-07 | 3.80E-06 | Mzb1 |
| 10476102 | -1.21701 | 6.620584 | 7.174152 | 3.16E-07 | 3.81E-06 | Gm24451 |
| 10606366 | 1.498118 | 5.277207 | -7.17295 | 3.17E-07 | 3.81E-06 | Zcchc5 |
| 10541885 | -1.0535 | 5.229971 | 7.171804 | 3.18E-07 | 3.82E-06 | Scnn1a |
| 10593293 | 1.02388 | 7.365622 | -7.16885 | 3.20E-07 | 3.84E-06 | Ncam1 |
| 10592330 | -1.3938 | 6.878213 | 7.167405 | 3.21E-07 | 3.85E-06 | Nrgn |
| 10522530 | -1.1453 | 6.51473 | 7.161878 | 3.25E-07 | 3.89E-06 | Kit |
| 10392822 | 1.003062 | 6.080877 | -7.15079 | 3.33E-07 | 3.97E-06 | Gm11709 |
| 10345807 | -1.2605 | 5.25856 | 7.147397 | 3.35E-07 | 4.00E-06 | Il18r1 |
| 10523156 | 2.667042 | 5.993827 | -7.13874 | 3.42E-07 | 4.06E-06 | Cxcl2 |
| 10408225 | -1.40549 | 8.898409 | 7.133496 | 3.46E-07 | 4.10E-06 | Hist1h4c |
| 10403079 | -2.05816 | 6.358159 | 7.12925 | 3.49E-07 | 4.14E-06 | LOC435333 |
| 10382106 | -1.06596 | 6.625809 | 7.12273 | 3.54E-07 | 4.19E-06 | Milr1 |
| 10346000 | 1.35104 | 5.92701 | -7.11386 | 3.61E-07 | 4.26E-06 | Gulp1 |
| 10496438 | 1.375083 | 6.848993 | -7.09872 | 3.73E-07 | 4.38E-06 | Adh1 |
| 10407281 | -1.32814 | 6.327274 | 7.097841 | 3.74E-07 | 4.39E-06 | Esm1 |
| 10373918 | 1.197651 | 5.216587 | -7.09599 | 3.76E-07 | 4.40E-06 | Lif |
| 10540275 | 1.506151 | 6.096806 | -7.09373 | 3.77E-07 | 4.42E-06 | Gxylt2 |
| 10405216 | -1.15121 | 7.659185 | 7.089307 | 3.81E-07 | 4.46E-06 | Syk |
| 10538903 | -1.78373 | 5.764636 | 7.089012 | 3.81E-07 | 4.46E-06 | Igk |
| 10509168 | -1.83521 | 7.220915 | 7.087881 | 3.82E-07 | 4.47E-06 | E2f2 |
| 10369481 | 1.06947 | 6.800723 | -7.053 | 4.13E-07 | 4.80E-06 | H2afy2 |
| 10398996 | 1.107052 | 7.380443 | -7.04564 | 4.20E-07 | 4.88E-06 | Crip2 |
| 10505623 | 1.465408 | 5.407089 | -7.04232 | 4.23E-07 | 4.91E-06 | Lurap1l |
| 10406530 | 1.017118 | 8.104936 | -7.03505 | 4.30E-07 | 4.97E-06 | Tmem167 |
| 10601044 | -1.76008 | 6.714125 | 7.030132 | 4.35E-07 | 5.02E-06 | Gdpd2 |
| 10457644 | 1.493007 | 6.565688 | -7.02887 | 4.36E-07 | 5.03E-06 | Cdh2 |
| 10546010 | -1.10415 | 6.983869 | 7.027275 | 4.37E-07 | 5.04E-06 | Arhgap25 |
| 10517967 | 1.304851 | 6.196417 | -7.01977 | 4.45E-07 | 5.12E-06 | Fblim1 |
| 10408741 | 1.17864 | 7.980727 | -7.01839 | 4.46E-07 | 5.13E-06 | Txndc5 |
| 10545198 | -3.06738 | 7.729403 | 7.010799 | 4.54E-07 | 5.21E-06 | Igkv4-59 |
| 10430372 | -1.72564 | 9.589616 | 7.008034 | 4.57E-07 | 5.24E-06 | Rac2 |
| 10445781 | 1.152116 | 7.082641 | -7.00325 | 4.62E-07 | 5.28E-06 | Trem2 |
| 10364601 | -1.19165 | 6.725074 | 6.996553 | 4.68E-07 | 5.35E-06 | Abca7 |
| 10475362 | -1.00074 | 6.769511 | 6.981924 | 4.84E-07 | 5.50E-06 | Wdr76 |
| 10446553 | 1.178474 | 6.121843 | -6.98186 | 4.84E-07 | 5.50E-06 | Epb4.1l3 |
| 10472916 | -1.05675 | 7.112464 | 6.979084 | 4.87E-07 | 5.54E-06 | Cdca7 |
| 10545196 | -2.4344 | 7.953185 | 6.963045 | 5.05E-07 | 5.71E-06 | Igkv4-61 |
| 10459288 | -1.42067 | 7.902535 | 6.961292 | 5.07E-07 | 5.72E-06 | Adrb2 |
| 10474875 | -1.34439 | 8.713803 | 6.953524 | 5.16E-07 | 5.81E-06 | Casc5 |
| 10542120 | -1.13596 | 6.312635 | 6.944067 | 5.27E-07 | 5.91E-06 | Clec2i |
| 10604528 | -1.34848 | 7.422052 | 6.939083 | 5.33E-07 | 5.97E-06 | Mbnl3 |
| 10375751 | 1.116088 | 7.41011 | -6.93861 | 5.33E-07 | 5.97E-06 | Adamts2 |
| 10380285 | 1.472353 | 6.226448 | -6.92866 | 5.45E-07 | 6.09E-06 | Tmem100 |
| 10348451 | 1.145845 | 6.55639 | -6.92675 | 5.48E-07 | 6.11E-06 | Ackr3 |
| 10541644 | 1.660077 | 6.364171 | -6.92427 | 5.51E-07 | 6.14E-06 | Cd163 |
| 10385635 | 1.231697 | 6.881351 | -6.91917 | 5.57E-07 | 6.20E-06 | Zfp354c |
| 10506301 | -1.36907 | 6.732201 | 6.918054 | 5.58E-07 | 6.21E-06 | Lepr |
| 10577164 | 1.020568 | 8.328524 | -6.9168 | 5.60E-07 | 6.22E-06 | Gas6 |
| 10545175 | -2.31939 | 7.037552 | 6.915675 | 5.61E-07 | 6.24E-06 | Igkv10-94 |
| 10545184 | -2.61384 | 7.957006 | 6.914072 | 5.63E-07 | 6.25E-06 | Igkv4-74 |
| 10554863 | 1.323236 | 5.936085 | -6.90675 | 5.73E-07 | 6.34E-06 | Sytl2 |
| 10379636 | -1.62602 | 8.557447 | 6.903093 | 5.77E-07 | 6.38E-06 | Slfn4 |
| 10430510 | 1.162999 | 7.088151 | -6.89985 | 5.82E-07 | 6.43E-06 | Tmem184b |
| 10562720 | -1.44395 | 7.414443 | 6.884458 | 6.02E-07 | 6.61E-06 | Siglece |
| 10471486 | 1.196731 | 7.222237 | -6.884 | 6.03E-07 | 6.61E-06 | Eng |
| 10478572 | -1.28002 | 9.182678 | 6.869558 | 6.22E-07 | 6.80E-06 | Ube2c |
| 10407940 | -1.14694 | 4.205661 | 6.868196 | 6.24E-07 | 6.81E-06 | Tcrg-V4 |
| 10559509 | 1.148484 | 6.582489 | -6.86639 | 6.27E-07 | 6.83E-06 | Cdc42ep5 |
| 10548552 | -1.10032 | 6.883143 | 6.85197 | 6.48E-07 | 7.01E-06 | Klra2 |
| 10487588 | -1.43984 | 4.872853 | 6.849983 | 6.50E-07 | 7.04E-06 | Il1a |
| 10553256 | 1.248014 | 6.619445 | -6.84353 | 6.60E-07 | 7.13E-06 | Myod1 |
| 10395320 | 1.378766 | 6.111656 | -6.82803 | 6.83E-07 | 7.36E-06 | Twist1 |
| 10365344 | -1.1688 | 7.947585 | 6.819808 | 6.96E-07 | 7.49E-06 | Tcp11l2 |
| 10595211 | 1.995716 | 8.711436 | -6.81473 | 7.04E-07 | 7.54E-06 | Col12a1 |
| 10559649 | -1.60298 | 7.035352 | 6.813853 | 7.06E-07 | 7.56E-06 | Cox6b2 |
| 10508454 | -1.22879 | 9.096589 | 6.781665 | 7.59E-07 | 8.02E-06 | Bsdc1 |
| 10502335 | -1.67026 | 6.693701 | 6.781006 | 7.60E-07 | 8.03E-06 | Bank1 |
| 10403959 | -1.01984 | 11.0919 | 6.780052 | 7.61E-07 | 8.04E-06 | Hist1h2bq |
| 10408087 | -1.01984 | 11.0919 | 6.780052 | 7.61E-07 | 8.04E-06 | Hist1h2bq |
| 10407792 | 1.088081 | 8.175645 | -6.7764 | 7.68E-07 | 8.09E-06 | Gpr137b-ps |
| 10545190 | -3.13519 | 5.527801 | 6.774749 | 7.71E-07 | 8.11E-06 | Igkv4-69 |
| 10595768 | -1.28021 | 5.613977 | 6.771443 | 7.76E-07 | 8.17E-06 | Pls1 |
| 10457587 | 1.094031 | 6.576051 | -6.7708 | 7.78E-07 | 8.17E-06 | Zfp521 |
| 10363962 | -1.47909 | 5.917663 | 6.766219 | 7.86E-07 | 8.25E-06 | Gnaz |
| 10583008 | 1.12421 | 5.954306 | -6.76259 | 7.92E-07 | 8.31E-06 | Casp12 |
| 10422348 | 1.098736 | 5.673316 | -6.74791 | 8.19E-07 | 8.56E-06 | Uggt2 |
| 10479041 | -1.97384 | 8.738341 | 6.744377 | 8.25E-07 | 8.61E-06 | Rbm38 |
| 10436024 | -1.65364 | 5.627814 | 6.74292 | 8.28E-07 | 8.63E-06 | Gcsam |
| 10396074 | 1.104434 | 7.413096 | -6.73827 | 8.37E-07 | 8.71E-06 | Mgat2 |
| 10494978 | -1.02379 | 7.050857 | 6.725784 | 8.61E-07 | 8.94E-06 | Ptpn22 |
| 10408081 | -1.46779 | 10.02134 | 6.721647 | 8.69E-07 | 9.01E-06 | Hist1h1b |
| 10379127 | -1.00729 | 7.24626 | 6.719162 | 8.74E-07 | 9.06E-06 | Spag5 |
| 10545187 | -2.8043 | 7.062549 | 6.714814 | 8.83E-07 | 9.13E-06 | Igkv4-70 |
| 10558150 | 1.291297 | 6.584552 | -6.71383 | 8.85E-07 | 9.15E-06 | Htra1 |
| 10512669 | -1.30774 | 5.698545 | 6.704801 | 9.03E-07 | 9.27E-06 | Pax5 |
| 10473312 | 1.524564 | 5.3251 | -6.69929 | 9.14E-07 | 9.37E-06 | Fam171b |
| 10545173 | -2.51326 | 6.62956 | 6.697865 | 9.17E-07 | 9.39E-06 | Igkv10-96 |
| 10541910 | -1.16643 | 7.300856 | 6.697581 | 9.18E-07 | 9.40E-06 | Vwf |
| 10555460 | -1.15985 | 7.213129 | 6.696175 | 9.21E-07 | 9.42E-06 | Stard10 |
| 10440258 | 1.551406 | 5.74997 | -6.69359 | 9.26E-07 | 9.47E-06 | Epha3 |
| 10440099 | -1.06163 | 7.687095 | 6.692647 | 9.28E-07 | 9.47E-06 | St3gal6 |
| 10594110 | 1.221165 | 7.891941 | -6.68754 | 9.39E-07 | 9.58E-06 | Neo1 |
| 10351667 | -1.42469 | 4.975166 | 6.683171 | 9.48E-07 | 9.66E-06 | Slamf1 |
| 10472757 | 1.14526 | 5.96943 | -6.67736 | 9.61E-07 | 9.76E-06 | Cybrd1 |
| 10395293 | -1.2449 | 7.759987 | 6.674742 | 9.67E-07 | 9.82E-06 | Atxn7l1 |
| 10386058 | 1.024115 | 12.01893 | -6.65605 | 1.01E-06 | 1.02E-05 | Sparc |
| 10492231 | -1.06256 | 5.595609 | 6.653618 | 1.01E-06 | 1.02E-05 | Med12l |
| 10346576 | -1.02141 | 7.111451 | 6.646593 | 1.03E-06 | 1.04E-05 | Stradb |
| 10465587 | -1.34366 | 7.713963 | 6.639615 | 1.05E-06 | 1.05E-05 | Fermt3 |
| 10390707 | -1.07084 | 9.490157 | 6.63435 | 1.06E-06 | 1.06E-05 | Top2a |
| 10408629 | 1.005852 | 5.749705 | -6.62938 | 1.07E-06 | 1.07E-05 | Pxdc1 |
| 10495763 | -1.11694 | 8.533442 | 6.618831 | 1.10E-06 | 1.10E-05 | Gclm |
| 10569011 | 1.743943 | 7.534523 | -6.59771 | 1.15E-06 | 1.14E-05 | Ifitm5 |
| 10536845 | 1.54137 | 7.227509 | -6.59205 | 1.17E-06 | 1.16E-05 | Flnc |
| 10584821 | -1.6822 | 4.741231 | 6.585196 | 1.19E-06 | 1.17E-05 | Cd3d |
| 10586244 | -1.1087 | 8.343531 | 6.571553 | 1.22E-06 | 1.20E-05 | Dennd4a |
| 10400748 | -1.00568 | 4.77696 | 6.57137 | 1.22E-06 | 1.20E-05 | Cdkl1 |
| 10415413 | 1.127837 | 5.885636 | -6.56037 | 1.25E-06 | 1.23E-05 | Nynrin |
| 10507500 | -1.29192 | 7.207496 | 6.554701 | 1.27E-06 | 1.24E-05 | Slc6a9 |
| 10541729 | -1.16604 | 9.306189 | 6.548135 | 1.29E-06 | 1.26E-05 | Cdca3 |
| 10404996 | 1.206348 | 7.583549 | -6.53979 | 1.31E-06 | 1.28E-05 | Ninj1 |
| 10598750 | 1.070556 | 5.123287 | -6.53211 | 1.34E-06 | 1.30E-05 | Gpr34 |
| 10463263 | 1.131964 | 7.19168 | -6.52969 | 1.35E-06 | 1.31E-05 | Lztfl1 |
| 10511180 | 1.270342 | 9.133629 | -6.52486 | 1.36E-06 | 1.32E-05 | Mxra8 |
| 10502359 | -1.08928 | 6.711801 | 6.517313 | 1.38E-06 | 1.34E-05 | Dapp1 |
| 10605874 | 1.676197 | 5.351497 | -6.51582 | 1.39E-06 | 1.34E-05 | Eda2r |
| 10508074 | -1.31389 | 8.16975 | 6.513371 | 1.40E-06 | 1.35E-05 | Csf3r |
| 10398326 | 1.205458 | 6.64194 | -6.51212 | 1.40E-06 | 1.35E-05 | Meg3 |
| 10526564 | -1.54992 | 7.222478 | 6.504219 | 1.43E-06 | 1.37E-05 | Ufsp1 |
| 10562709 | -1.17696 | 8.849211 | 6.49649 | 1.45E-06 | 1.39E-05 | Cd33 |
| 10523727 | 1.000004 | 7.347903 | -6.49283 | 1.46E-06 | 1.40E-05 | Pkd2 |
| 10461605 | -1.32766 | 5.657701 | 6.489985 | 1.47E-06 | 1.41E-05 | Ms4a4b |
| 10403941 | -1.37474 | 11.01898 | 6.482034 | 1.50E-06 | 1.43E-05 | Hist1h3f |
| 10551815 | 1.064157 | 7.655396 | -6.48129 | 1.50E-06 | 1.43E-05 | Zfp260 |
| 10368289 | 1.196519 | 8.019532 | -6.46577 | 1.56E-06 | 1.48E-05 | Enpp1 |
| 10492355 | 1.234517 | 6.92919 | -6.46375 | 1.57E-06 | 1.49E-05 | Mme |
| 10408246 | -1.35612 | 11.01036 | 6.452356 | 1.61E-06 | 1.52E-05 | Hist1h3f |
| 10362102 | -1.14897 | 7.311211 | 6.448252 | 1.62E-06 | 1.53E-05 | Gm10825 |
| 10404028 | -1.34634 | 11.02241 | 6.444031 | 1.64E-06 | 1.54E-05 | Hist1h3f |
| 10492021 | 2.044209 | 10.32674 | -6.43647 | 1.67E-06 | 1.57E-05 | Postn |
| 10576757 | -1.71825 | 5.026925 | 6.436336 | 1.67E-06 | 1.57E-05 | Fcer2a |
| 10361292 | -1.05017 | 4.800355 | 6.435091 | 1.67E-06 | 1.57E-05 | Cr2 |
| 10379176 | -1.18916 | 6.360915 | 6.41731 | 1.74E-06 | 1.63E-05 | Unc119 |
| 10388430 | 1.172686 | 10.05281 | -6.40364 | 1.80E-06 | 1.67E-05 | Serpinf1 |
| 10430956 | 1.170152 | 9.35886 | -6.39096 | 1.85E-06 | 1.72E-05 | Cyb5r3 |
| 10419288 | -1.49016 | 6.674464 | 6.389866 | 1.86E-06 | 1.72E-05 | Gch1 |
| 10367440 | 1.045627 | 5.72549 | -6.38897 | 1.86E-06 | 1.72E-05 | Itga7 |
| 10444284 | -1.35317 | 5.827646 | 6.387744 | 1.86E-06 | 1.73E-05 | H2-Ob |
| 10572398 | 1.026531 | 5.822223 | -6.37999 | 1.90E-06 | 1.75E-05 | Crlf1 |
| 10564978 | -1.0123 | 6.532995 | 6.377895 | 1.91E-06 | 1.76E-05 | Blm |
| 10408083 | -1.35835 | 11.02363 | 6.375455 | 1.92E-06 | 1.77E-05 | Hist1h3a |
| 10349593 | -1.19208 | 6.122221 | 6.371896 | 1.93E-06 | 1.78E-05 | Faim3 |
| 10572861 | -1.1667 | 5.320342 | 6.371347 | 1.94E-06 | 1.78E-05 | F2rl3 |
| 10462796 | -1.23988 | 8.571466 | 6.369114 | 1.95E-06 | 1.79E-05 | Kif11 |
| 10346150 | 1.024911 | 5.064845 | -6.36871 | 1.95E-06 | 1.79E-05 | Tmeff2 |
| 10408202 | -1.37125 | 11.00119 | 6.365223 | 1.96E-06 | 1.80E-05 | Hist1h3e |
| 10563780 | -1.6073 | 7.528505 | 6.338961 | 2.09E-06 | 1.90E-05 | E2f8 |
| 10443980 | -1.09083 | 8.62327 | 6.328889 | 2.14E-06 | 1.93E-05 | Myo1f |
| 10538135 | -1.55412 | 4.790744 | 6.327008 | 2.15E-06 | 1.94E-05 | Gimap7 |
| 10479221 | 1.084664 | 6.754869 | -6.32586 | 2.15E-06 | 1.95E-05 | Gm14403 |
| 10403011 | -1.0298 | 3.447029 | 6.32352 | 2.16E-06 | 1.96E-05 | Ighv13-2 |
| 10471721 | -1.48963 | 7.319227 | 6.320374 | 2.18E-06 | 1.97E-05 | Ptgs1 |
| 10489463 | -1.29343 | 9.012082 | 6.318391 | 2.19E-06 | 1.97E-05 | Slpi |
| 10586454 | -1.07758 | 5.610715 | 6.318097 | 2.19E-06 | 1.97E-05 | D030028M11Rik |
| 10541260 | -1.20077 | 5.855584 | 6.317699 | 2.19E-06 | 1.98E-05 | Cecr2 |
| 10404049 | -1.38712 | 11.11617 | 6.310876 | 2.23E-06 | 2.00E-05 | Hist1h3a |
| 10408239 | -1.38425 | 11.06745 | 6.306943 | 2.25E-06 | 2.02E-05 | Hist1h3a |
| 10382998 | -1.00907 | 8.814618 | 6.305441 | 2.26E-06 | 2.03E-05 | Birc5 |
| 10404065 | -1.38762 | 11.10032 | 6.303421 | 2.27E-06 | 2.03E-05 | Hist1h3f |
| 10521731 | -1.06356 | 7.802835 | 6.301135 | 2.28E-06 | 2.04E-05 | Ncapg |
| 10359762 | -1.0411 | 7.651812 | 6.288169 | 2.35E-06 | 2.09E-05 | Rcsd1 |
| 10531724 | -1.25578 | 9.368838 | 6.283772 | 2.37E-06 | 2.11E-05 | Plac8 |
| 10545194 | -1.93233 | 5.304756 | 6.282391 | 2.38E-06 | 2.11E-05 | Igkv4-62 |
| 10544588 | -1.79329 | 5.136676 | 6.280215 | 2.39E-06 | 2.12E-05 | Gimap3 |
| 10547943 | -1.09154 | 7.911796 | 6.278866 | 2.40E-06 | 2.13E-05 | Ncapd2 |
| 10404051 | -1.00686 | 11.87341 | 6.276503 | 2.41E-06 | 2.14E-05 | Hist1h4d |
| 10355325 | -1.22905 | 6.344524 | 6.271004 | 2.44E-06 | 2.16E-05 | Bard1 |
| 10423548 | 1.055302 | 7.621979 | -6.26919 | 2.45E-06 | 2.17E-05 | Sdc2 |
| 10437160 | 1.076207 | 7.314712 | -6.26494 | 2.48E-06 | 2.19E-05 | Ets2 |
| 10487238 | -1.39257 | 7.800682 | 6.255016 | 2.54E-06 | 2.23E-05 | Hdc |
| 10515385 | -1.00186 | 7.958007 | 6.253963 | 2.54E-06 | 2.23E-05 | Urod |
| 10408197 | -1.0035 | 9.191459 | 6.240794 | 2.62E-06 | 2.29E-05 | Hist1h2bh |
| 10592515 | -1.36016 | 6.854932 | 6.239929 | 2.63E-06 | 2.30E-05 | Ubash3b |
| 10525439 | 1.116461 | 7.871344 | -6.23905 | 2.63E-06 | 2.30E-05 | P2rx4 |
| 10408210 | -1.07577 | 11.17208 | 6.237128 | 2.64E-06 | 2.31E-05 | Hist1h2bf |
| 10361771 | 1.10024 | 6.778871 | -6.22405 | 2.72E-06 | 2.37E-05 | Plagl1 |
| 10501802 | -1.41006 | 6.302017 | 6.221816 | 2.74E-06 | 2.38E-05 | Tmem56 |
| 10370644 | -1.12861 | 6.052683 | 6.204991 | 2.85E-06 | 2.46E-05 | Prss57 |
| 10592266 | 1.139772 | 6.750504 | -6.18321 | 3.00E-06 | 2.57E-05 | Slc37a2 |
| 10568586 | -1.1078 | 7.326355 | 6.164791 | 3.13E-06 | 2.67E-05 | Fam53b |
| 10585438 | 1.697018 | 6.460423 | -6.1524 | 3.22E-06 | 2.73E-05 | Crabp1 |
| 10346843 | 1.129492 | 7.747766 | -6.14501 | 3.28E-06 | 2.77E-05 | Nrp2 |
| 10444824 | -1.74268 | 7.288092 | 6.129368 | 3.40E-06 | 2.86E-05 | H2-Q8 |
| 10573419 | -1.01243 | 7.55485 | 6.12119 | 3.46E-06 | 2.91E-05 | Lyl1 |
| 10571865 | -1.94628 | 6.379676 | 6.111164 | 3.55E-06 | 2.97E-05 | Scrg1 |
| 10473240 | 1.076605 | 9.699312 | -6.10809 | 3.57E-06 | 2.99E-05 | Eno1b |
| 10474381 | -1.27601 | 7.385063 | 6.107069 | 3.58E-06 | 2.99E-05 | Kif18a |
| 10369842 | 1.390457 | 7.776248 | -6.10107 | 3.63E-06 | 3.02E-05 | Bicc1 |
| 10572897 | 1.420729 | 10.42457 | -6.09854 | 3.65E-06 | 3.04E-05 | Hmox1 |
| 10586248 | -1.0184 | 7.99776 | 6.09377 | 3.69E-06 | 3.07E-05 | Dennd4a |
| 10590494 | -1.06777 | 7.63781 | 6.080337 | 3.81E-06 | 3.15E-05 | Kif15 |
| 10424119 | 1.217702 | 6.069293 | -6.07873 | 3.82E-06 | 3.16E-05 | Nov |
| 10545208 | -1.8329 | 6.235027 | 6.070468 | 3.90E-06 | 3.21E-05 | Igkv4-57 |
| 10500677 | -1.60351 | 5.895566 | 6.060379 | 3.99E-06 | 3.28E-05 | Cd2 |
| 10435185 | -1.14857 | 6.91319 | 6.045244 | 4.14E-06 | 3.38E-05 | n-R5s33 |
| 10548409 | -1.45261 | 4.878014 | 6.043816 | 4.15E-06 | 3.39E-05 | Klrc1 |
| 10404036 | -1.1334 | 6.408097 | 6.034766 | 4.24E-06 | 3.45E-05 | Hist1h2bg |
| 10494402 | -1.32074 | 10.9048 | 6.02371 | 4.35E-06 | 3.53E-05 | Hist2h3c2 |
| 10445977 | -1.18572 | 6.866104 | 6.017718 | 4.41E-06 | 3.58E-05 | Ebi3 |
| 10565811 | -1.1307 | 6.714052 | 6.008794 | 4.51E-06 | 3.64E-05 | Snord15b |
| 10602385 | -1.12131 | 6.004738 | 6.008098 | 4.51E-06 | 3.64E-05 | Pfkfb1 |
| 10438904 | 1.377361 | 6.045423 | -6.00723 | 4.52E-06 | 3.65E-05 | Lrrc15 |
| 10391811 | -1.3548 | 7.173063 | 5.998186 | 4.62E-06 | 3.72E-05 | Kif18b |
| 10500333 | -1.00969 | 10.56044 | 5.998011 | 4.62E-06 | 3.72E-05 | Hist2h4 |
| 10401068 | -1.65153 | 7.723535 | 5.990866 | 4.70E-06 | 3.77E-05 | Sptb |
| 10458314 | 1.041665 | 7.686679 | -5.99024 | 4.71E-06 | 3.78E-05 | Tmem173 |
| 10597182 | -1.08036 | 7.083769 | 5.987633 | 4.74E-06 | 3.80E-05 | Nbeal2 |
| 10593024 | -1.039 | 6.128839 | 5.977917 | 4.85E-06 | 3.87E-05 | Cd3e |
| 10403948 | -1.09454 | 11.31484 | 5.974321 | 4.89E-06 | 3.90E-05 | Hist1h2bn |
| 10578322 | 1.367339 | 7.334997 | -5.97237 | 4.91E-06 | 3.91E-05 | Gm9868 |
| 10606868 | 1.416739 | 6.660555 | -5.96794 | 4.96E-06 | 3.94E-05 | Bex1 |
| 10453747 | 1.06561 | 7.40958 | -5.9657 | 4.99E-06 | 3.96E-05 | Colec12 |
| 10458828 | 1.341543 | 7.094762 | -5.95402 | 5.13E-06 | 4.06E-05 | Cdo1 |
| 10586240 | -1.06364 | 9.48226 | 5.949765 | 5.18E-06 | 4.09E-05 | Dennd4a |
| 10492971 | -1.1451 | 5.098492 | 5.911127 | 5.67E-06 | 4.43E-05 | Fcrl1 |
| 10346321 | 1.372238 | 5.347767 | -5.90059 | 5.82E-06 | 4.52E-05 | Gm10561 |
| 10435019 | -1.229 | 5.907526 | 5.891726 | 5.94E-06 | 4.60E-05 | Smco1 |
| 10494405 | -1.36344 | 10.97909 | 5.89104 | 5.95E-06 | 4.61E-05 | Hist2h3c2 |
| 10572378 | 1.373089 | 7.013379 | -5.89008 | 5.96E-06 | 4.62E-05 | Comp |
| 10423498 | 1.025724 | 9.384191 | -5.84989 | 6.56E-06 | 5.02E-05 | Dap |
| 10462618 | 1.461216 | 7.540175 | -5.84968 | 6.56E-06 | 5.02E-05 | Ifit3 |
| 10406519 | 2.193516 | 5.108258 | -5.82806 | 6.90E-06 | 5.25E-05 | Hapln1 |
| 10574259 | -1.12181 | 7.099584 | 5.805789 | 7.28E-06 | 5.51E-05 | Gpr56 |
| 10467766 | 1.339799 | 5.491125 | -5.79048 | 7.55E-06 | 5.68E-05 | Loxl4 |
| 10566585 | -1.19915 | 7.752969 | 5.788488 | 7.58E-06 | 5.70E-05 | Gm1966 |
| 10537410 | -1.04299 | 6.643282 | 5.77648 | 7.80E-06 | 5.84E-05 | Tbxas1 |
| 10363445 | -1.01342 | 8.05469 | 5.774745 | 7.84E-06 | 5.86E-05 | 4632428N05Rik |
| 10461979 | -1.51684 | 8.316925 | 5.767223 | 7.98E-06 | 5.96E-05 | Aldh1a1 |
| 10380489 | -1.19331 | 6.508128 | 5.744027 | 8.43E-06 | 6.24E-05 | Samd14 |
| 10387625 | 1.224503 | 5.80203 | -5.74219 | 8.47E-06 | 6.27E-05 | Chrnb1 |
| 10403980 | -1.03628 | 11.54734 | 5.738244 | 8.55E-06 | 6.31E-05 | Hist1h2bq |
| 10545220 | -1.92642 | 4.709231 | 5.724239 | 8.84E-06 | 6.49E-05 | Igkv12-41 |
| 10403978 | -1.07615 | 11.36693 | 5.704739 | 9.26E-06 | 6.77E-05 | Hist1h2bq |
| 10410984 | -1.94078 | 7.807899 | 5.663555 | 1.02E-05 | 7.39E-05 | Ckmt2 |
| 10517587 | 1.503688 | 7.854767 | -5.66022 | 1.03E-05 | 7.44E-05 | Alpl |
| 10417212 | 1.228599 | 7.229827 | -5.65183 | 1.05E-05 | 7.56E-05 | Itgbl1 |
| 10546450 | 1.119508 | 5.886799 | -5.6515 | 1.05E-05 | 7.57E-05 | Adamts9 |
| 10366446 | -1.66992 | 8.489043 | 5.641927 | 1.08E-05 | 7.72E-05 | Tspan8 |
| 10430931 | -1.04464 | 7.055765 | 5.6339 | 1.10E-05 | 7.85E-05 | Nfam1 |
| 10504668 | -1.06655 | 6.086103 | 5.622518 | 1.13E-05 | 8.04E-05 | E230008N13Rik |
| 10408070 | -1.04001 | 11.43061 | 5.613076 | 1.15E-05 | 8.20E-05 | Hist1h2bq |
| 10350840 | 1.298616 | 7.14678 | -5.60992 | 1.16E-05 | 8.25E-05 | Angptl1 |
| 10586079 | 1.132834 | 6.390788 | -5.56842 | 1.28E-05 | 8.99E-05 | Itga11 |
| 10356262 | -1.07068 | 7.109662 | 5.564577 | 1.29E-05 | 9.07E-05 | Gm7609 |
| 10388488 | -1.20413 | 9.327008 | 5.557112 | 1.32E-05 | 9.21E-05 | Fam101b |
| 10425852 | -1.40089 | 8.662688 | 5.540714 | 1.37E-05 | 9.51E-05 | Parvb |
| 10594798 | -1.43344 | 6.261172 | 5.532813 | 1.40E-05 | 9.65E-05 | Gm23730 |
| 10394054 | -1.08824 | 5.712508 | 5.52146 | 1.43E-05 | 9.90E-05 | Cd7 |
| 10504753 | -1.07584 | 4.221954 | 5.505999 | 1.49E-05 | 0.000102 | Sympk |
| 10576581 | 1.11167 | 6.146605 | -5.50215 | 1.50E-05 | 0.000103 | Kcnk1 |
| 10394978 | -1.30608 | 8.862416 | 5.496962 | 1.52E-05 | 0.000104 | Rrm2 |
| 10406968 | -1.09985 | 7.448981 | 5.495277 | 1.53E-05 | 0.000104 | Cenpk |
| 10585194 | 1.039747 | 6.274906 | -5.49015 | 1.55E-05 | 0.000106 | Il18 |
| 10545215 | -2.75472 | 5.794537 | 5.483662 | 1.57E-05 | 0.000107 | Igkv12-46 |
| 10597239 | 1.085884 | 6.914616 | -5.44855 | 1.71E-05 | 0.000115 | Pth1r |
| 10530145 | 1.218042 | 6.773157 | -5.43294 | 1.77E-05 | 0.000119 | Tlr1 |
| 10398354 | 1.2001 | 4.693777 | -5.42377 | 1.81E-05 | 0.000121 | Gm24564 |
| 10538890 | -1.07307 | 4.728038 | 5.409128 | 1.88E-05 | 0.000125 | Sympk |
| 10562761 | 1.139287 | 6.948903 | -5.39954 | 1.92E-05 | 0.000128 | Clec11a |
| 10404132 | -1.07227 | 6.335441 | 5.39615 | 1.94E-05 | 0.000129 | Cmah |
| 10531191 | 1.135089 | 6.184434 | -5.39004 | 1.97E-05 | 0.00013 | Adamts3 |
| 10480891 | -1.03427 | 8.252365 | 5.358195 | 2.13E-05 | 0.000139 | Ubac1 |
| 10504761 | -1.10475 | 4.200854 | 5.356072 | 2.14E-05 | 0.00014 | Sympk |
| 10421517 | -1.15862 | 5.078425 | 5.346202 | 2.19E-05 | 0.000143 | Cysltr2 |
| 10445112 | -1.95539 | 5.537542 | 5.326199 | 2.30E-05 | 0.000149 | Ubd |
| 10510546 | 1.036714 | 9.552508 | -5.32484 | 2.30E-05 | 0.00015 | Eno1b |
| 10557855 | 1.113715 | 6.089959 | -5.30271 | 2.43E-05 | 0.000156 | Trim72 |
| 10489701 | 1.094092 | 6.040993 | -5.29451 | 2.48E-05 | 0.000159 | Ocstamp |
| 10498935 | -1.0513 | 5.429321 | 5.289984 | 2.51E-05 | 0.00016 | Gucy1b3 |
| 10538887 | -1.2126 | 4.223468 | 5.283185 | 2.55E-05 | 0.000163 | Igkv2-112 |
| 10427628 | -1.14588 | 6.255865 | 5.282468 | 2.55E-05 | 0.000163 | Il7r |
| 10587616 | 1.916087 | 6.057896 | -5.28041 | 2.57E-05 | 0.000163 | Prss35 |
| 10363173 | 1.186599 | 9.495978 | -5.27167 | 2.62E-05 | 0.000167 | Gja1 |
| 10567108 | -1.07393 | 7.546573 | 5.255317 | 2.73E-05 | 0.000172 | Sox6 |
| 10401931 | -1.00673 | 4.530156 | 5.241879 | 2.82E-05 | 0.000177 | Sympk |
| 10401937 | -1.00673 | 4.530156 | 5.241879 | 2.82E-05 | 0.000177 | Sympk |
| 10430344 | -1.28776 | 6.0017 | 5.21179 | 3.03E-05 | 0.000189 | Il2rb |
| 10532744 | -1.09886 | 8.904948 | 5.211091 | 3.03E-05 | 0.00019 | Selplg |
| 10505145 | 1.170271 | 5.775224 | -5.20564 | 3.07E-05 | 0.000192 | Musk |
| 10551696 | -1.10243 | 7.411005 | 5.180798 | 3.27E-05 | 0.000203 | Rasgrp4 |
| 10404061 | -1.55963 | 8.141631 | 5.17719 | 3.29E-05 | 0.000204 | Hist1h2bb |
| 10482030 | -1.00167 | 8.879665 | 5.166893 | 3.38E-05 | 0.000208 | Stom |
| 10593015 | -1.31205 | 5.54908 | 5.145978 | 3.55E-05 | 0.000218 | Cd3g |
| 10587231 | 1.067132 | 6.65899 | -5.14563 | 3.56E-05 | 0.000218 | Bmp5 |
| 10530615 | -1.21104 | 6.23016 | 5.134499 | 3.65E-05 | 0.000223 | Ociad2 |
| 10544273 | -1.12205 | 7.13244 | 5.114169 | 3.84E-05 | 0.000233 | Clec5a |
| 10582562 | -1.70362 | 9.860397 | 5.086486 | 4.11E-05 | 0.000247 | n-R5s151 |
| 10494262 | 1.382786 | 9.57847 | -5.0751 | 4.22E-05 | 0.000253 | Ctsk |
| 10601421 | 1.34066 | 5.841791 | -5.07079 | 4.27E-05 | 0.000255 | A630033H20Rik |
| 10484307 | 1.191531 | 4.783976 | -5.04989 | 4.49E-05 | 0.000266 | Frzb |
| 10502565 | -1.00168 | 5.638461 | 5.048754 | 4.50E-05 | 0.000267 | Clca2 |
| 10545210 | -1.87463 | 5.741305 | 5.004252 | 5.02E-05 | 0.000294 | Igkv4-55 |
| 10371506 | -1.10617 | 6.453913 | 4.975796 | 5.38E-05 | 0.000312 | Stab2 |
| 10475517 | -1.07102 | 7.826476 | 4.975316 | 5.39E-05 | 0.000312 | AA467197 |
| 10347928 | -1.04644 | 6.734294 | 4.957965 | 5.62E-05 | 0.000323 | Sp110 |
| 10582874 | -1.04644 | 6.734294 | 4.957965 | 5.62E-05 | 0.000323 | Sp110 |
| 10451763 | -1.10003 | 7.706934 | 4.957205 | 5.63E-05 | 0.000324 | Satb1 |
| 10550877 | -1.16179 | 7.359961 | 4.947955 | 5.76E-05 | 0.00033 | Kcnn4 |
| 10403063 | -1.40433 | 6.368563 | 4.938415 | 5.89E-05 | 0.000337 | Ighv8-12 |
| 10425799 | -1.01518 | 7.171477 | 4.936033 | 5.93E-05 | 0.000338 | Rnu12 |
| 10590417 | 1.019922 | 6.319354 | -4.93043 | 6.01E-05 | 0.000342 | Klhl40 |
| 10403943 | -1.23269 | 10.20159 | 4.926916 | 6.06E-05 | 0.000345 | Hist1h2bm |
| 10562132 | -1.06617 | 6.141278 | 4.894673 | 6.56E-05 | 0.000369 | Cd22 |
| 10362097 | -1.24934 | 6.732495 | 4.859564 | 7.15E-05 | 0.000396 | H60b |
| 10601648 | 1.320279 | 7.3522 | -4.85665 | 7.20E-05 | 0.000399 | Tnmd |
| 10538187 | 1.212027 | 7.586826 | -4.83085 | 7.67E-05 | 0.000421 | Gpnmb |
| 10495449 | 1.507963 | 7.358724 | -4.81221 | 8.02E-05 | 0.000437 | Col11a1 |
| 10377245 | -1.05384 | 6.053825 | 4.768864 | 8.92E-05 | 0.00048 | Dhrs7c |
| 10567171 | -1.07844 | 6.147432 | 4.755503 | 9.22E-05 | 0.000493 | Snord14a |
| 10517213 | 1.411243 | 5.596546 | -4.75367 | 9.26E-05 | 0.000495 | Cnksr1 |
| 10533050 | 1.204266 | 8.229059 | -4.74725 | 9.41E-05 | 0.000502 | Hspb8 |
| 10372230 | 1.455105 | 7.241803 | -4.74178 | 9.54E-05 | 0.000508 | Myf6 |
| 10574676 | 1.010563 | 7.19639 | -4.74168 | 9.54E-05 | 0.000508 | Nol3 |
| 10502552 | -1.2824 | 5.526438 | 4.733888 | 9.72E-05 | 0.000517 | Clca1 |
| 10398366 | 1.401021 | 5.420798 | -4.73353 | 9.73E-05 | 0.000517 | Gm25357 |
| 10423505 | -1.12732 | 6.692206 | 4.732944 | 9.75E-05 | 0.000518 | Cmbl |
| 10536220 | 1.400112 | 10.63524 | -4.7179 | 0.000101 | 0.000535 | Col1a2 |
| 10373367 | -1.0327 | 8.36133 | 4.717571 | 0.000101 | 0.000535 | Coq10a |
| 10444223 | -1.04704 | 6.108171 | 4.68418 | 0.00011 | 0.000574 | H2-Oa |
| 10533213 | -1.06919 | 6.116217 | 4.674536 | 0.000112 | 0.000587 | Oas3 |
| 10538126 | -1.02176 | 7.062289 | 4.670422 | 0.000114 | 0.000592 | Gimap4 |
| 10545239 | -2.08909 | 4.505288 | 4.658117 | 0.000117 | 0.000607 | LOC637260 |
| 10398362 | 1.206086 | 4.747685 | -4.6454 | 0.000121 | 0.000624 | AF357355 |
| 10438751 | -1.04476 | 2.936673 | 4.616143 | 0.00013 | 0.000664 | Gm24928 |
| 10513945 | -1.39441 | 6.939927 | 4.600337 | 0.000135 | 0.000688 | 2310002L09Rik |
| 10545237 | -1.93018 | 3.768255 | 4.539864 | 0.000157 | 0.000782 | Adck1 |
| 10545242 | -2.03289 | 4.780486 | 4.529549 | 0.000161 | 0.0008 | Igkv6-20 |
| 10551347 | -1.01549 | 9.29146 | 4.484264 | 0.000179 | 0.000883 | Blvrb |
| 10545180 | -1.18867 | 4.475097 | 4.471236 | 0.000185 | 0.000907 | Igkv4-91 |
| 10450482 | -1.69402 | 6.793895 | 4.461606 | 0.00019 | 0.000927 | Gm23442 |
| 10436363 | -1.00825 | 5.263958 | 4.45391 | 0.000193 | 0.00094 | Filip1l |
| 10534085 | -1.11327 | 5.778156 | 4.430973 | 0.000205 | 0.000988 | Phkg1 |
| 10542575 | -1.01066 | 6.031482 | 4.424646 | 0.000208 | 0.001002 | Pde3a |
| 10551836 | -1.13476 | 9.38653 | 4.391087 | 0.000226 | 0.001075 | Cox7a1 |
| 10387743 | -1.06688 | 7.356929 | 4.387756 | 0.000228 | 0.001082 | Slc2a4 |
| 10403034 | -1.67159 | 7.116187 | 4.353499 | 0.000248 | 0.001161 | Ighv8-8 |
| 10496262 | 1.034982 | 6.20876 | -4.31535 | 0.000272 | 0.001259 | Slc9b2 |
| 10492469 | -1.10478 | 6.553147 | 4.263807 | 0.000309 | 0.0014 | Mlf1 |
| 10380419 | 1.251692 | 10.49527 | -4.25229 | 0.000318 | 0.001433 | Col1a1 |
| 10509992 | 1.226589 | 8.042268 | -4.24481 | 0.000323 | 0.001456 | Hspb7 |
| 10402347 | 1.023862 | 10.1616 | -4.24439 | 0.000324 | 0.001457 | Ifi27l2a |
| 10543959 | 1.436689 | 7.825235 | -4.20484 | 0.000357 | 0.001586 | Ptn |
| 10380398 | -1.19261 | 7.552323 | 4.192941 | 0.000367 | 0.001623 | Chad |
| 10358652 | 1.330143 | 5.261714 | -4.16486 | 0.000394 | 0.001718 | Hmcn1 |
| 10508953 | 1.34425 | 7.333474 | -4.13315 | 0.000426 | 0.001839 | Trim63 |
| 10442584 | -1.13013 | 7.315469 | 4.107152 | 0.000454 | 0.001941 | Rpl3l |
| 10558090 | 1.013587 | 7.530353 | -4.10459 | 0.000456 | 0.001952 | Tacc2 |
| 10437180 | -1.17691 | 7.307866 | 4.050226 | 0.000522 | 0.00219 | Sh3bgr |
| 10538924 | -1.08288 | 6.763361 | 4.046122 | 0.000527 | 0.002208 | Igkv2-109 |
| 10365983 | 1.22063 | 10.19067 | -4.0425 | 0.000532 | 0.002225 | Lum |
| 10582580 | -1.3792 | 7.967261 | 4.036287 | 0.00054 | 0.002256 | n-R5s136 |
| 10346114 | -1.00848 | 6.751852 | 4.00058 | 0.000589 | 0.002432 | n-R5s211 |
| 10545247 | -2.14232 | 6.479969 | 3.989235 | 0.000606 | 0.002487 | Igkv6-14 |
| 10466127 | -1.01925 | 8.580694 | 3.987996 | 0.000608 | 0.002493 | AW112010 |
| 10389207 | -1.38651 | 7.981489 | 3.894087 | 0.000765 | 0.003036 | Ccl5 |
| 10385518 | -1.04322 | 7.687999 | 3.88303 | 0.000786 | 0.003105 | Tgtp2 |
| 10434778 | 1.071364 | 7.359313 | -3.88102 | 0.00079 | 0.003117 | Rtp4 |
| 10405047 | 1.017715 | 10.56565 | -3.84867 | 0.000855 | 0.003335 | Aspn |
| 10538882 | -1.76583 | 5.537377 | 3.834506 | 0.000885 | 0.003433 | Igkv9-124 |
| 10394488 | 1.018759 | 5.101744 | -3.82983 | 0.000895 | 0.003468 | Matn3 |
| 10582464 | -1.02647 | 6.225824 | 3.802306 | 0.000957 | 0.003675 | Gm24445 |
| 10371082 | -1.08125 | 7.366955 | 3.735437 | 0.001127 | 0.004234 | Nmrk2 |
| 10525343 | -1.33349 | 7.867317 | 3.722173 | 0.001163 | 0.004349 | Myl2 |
| 10494873 | -1.1163 | 9.245805 | 3.65309 | 0.001376 | 0.005027 | Ampd1 |
| 10478633 | -1.0716 | 9.368884 | 3.616405 | 0.001504 | 0.005422 | Mmp9 |
| 10545231 | -1.43858 | 6.735468 | 3.540529 | 0.001808 | 0.006314 | Igkv6-32 |
| 10403009 | -1.00091 | 3.757568 | 3.498423 | 0.002001 | 0.006896 | Ighv3-8 |
| 10417887 | -1.22831 | 7.72966 | 3.494088 | 0.002022 | 0.006953 | Mss51 |
| 10365987 | 1.078365 | 5.725259 | -3.48299 | 0.002077 | 0.007115 | Kera |
| 10378438 | -1.06148 | 4.938702 | 3.419871 | 0.002417 | 0.008072 | n-R5s71 |
| 10582582 | -1.23298 | 6.114436 | 3.312057 | 0.003129 | 0.010059 | Gm24089 |
| 10582584 | -1.23298 | 6.114436 | 3.312057 | 0.003129 | 0.010059 | Gm24089 |
| 10532582 | 1.111445 | 7.517465 | -3.2091 | 0.003996 | 0.012379 | Myo18b |
| 10532586 | 1.15654 | 6.659528 | -3.09301 | 0.005254 | 0.015548 | Myo18b |
| 10408543 | -1.16904 | 7.680267 | 2.814558 | 0.010013 | 0.026567 | Mylk4 |
| 10362436 | -1.03365 | 7.270954 | 2.682589 | 0.013502 | 0.034026 | Trdn |
| 10362440 | -1.07679 | 6.873465 | 2.664733 | 0.014054 | 0.035193 | Trdn |
| 10362456 | -1.10195 | 6.38608 | 2.487354 | 0.020817 | 0.048739 | Trdn |
| 10545177 | -1.21002 | 6.53994 | 2.406017 | 0.024841 | 0.056432 | Igkv19-93 |
