# Supplementary material for: Identification of miRNA Regulatory Networks and Candidate Markers for Fracture Healing in Mice
Source: Comput Math Methods Med. 2021 Nov 16;2021:2866475. doi: 10.1155/2021/2866475 (PMC8611357; doi:10.1155/2021/2866475)
Supplement: Supplementary 4 — Table S2: the DEmiRs between 10 days after fracture and 0 day. [file 2866475.f4.docx]

**Table S2.** The DEmiRs between 10 days after fracture and 0 day.

|  | logFC | AveExpr | t | P.Value | adj.P.Val | Symbol |
| --- | --- | --- | --- | --- | --- | --- |
| 10362495 | 5.055232 | 8.673661 | -45.3772 | 1.05E-23 | 3.02E-19 | Col10a1 |
| 10537062 | 5.195349 | 8.171214 | -41.6401 | 7.16E-23 | 1.03E-18 | Mest |
| 10384223 | 3.554365 | 8.130926 | -40.4178 | 1.39E-22 | 1.34E-18 | Igfbp3 |
| 10395103 | 3.381405 | 6.636028 | -35.4774 | 2.54E-21 | 1.83E-17 | Pxdn |
| 10406519 | 6.063794 | 6.951247 | -34.8183 | 3.85E-21 | 2.08E-17 | Hapln1 |
| 10384398 | 3.941456 | 8.027074 | -34.6338 | 4.33E-21 | 2.08E-17 | Grb10 |
| 10495830 | 2.632332 | 7.775068 | -33.7399 | 7.74E-21 | 3.19E-17 | Sec24d |
| 10547282 | 2.738257 | 6.677058 | -32.4684 | 1.81E-20 | 6.53E-17 | Zfp9 |
| 10592355 | 5.128455 | 7.409924 | -31.3712 | 3.88E-20 | 1.23E-16 | Panx3 |
| 10423080 | 6.073755 | 7.812848 | -31.2343 | 4.28E-20 | 1.23E-16 | C1qtnf3 |
| 10592342 | 1.798032 | 8.558249 | -29.3835 | 1.65E-19 | 3.96E-16 | Tbrg1 |
| 10601659 | 3.260112 | 7.352317 | -29.3809 | 1.65E-19 | 3.96E-16 | Srpx2 |
| 10362538 | 2.872922 | 7.411767 | -29.2337 | 1.84E-19 | 4.09E-16 | Lama4 |
| 10579812 | 2.187529 | 6.81258 | -29.0001 | 2.20E-19 | 4.31E-16 | Ednra |
| 10354741 | 2.157526 | 6.358968 | -28.9735 | 2.25E-19 | 4.31E-16 | Rftn2 |
| 10395163 | 2.503682 | 7.325262 | -28.3319 | 3.68E-19 | 6.42E-16 | Lamb1 |
| 10426689 | 2.213378 | 6.415591 | -28.2934 | 3.79E-19 | 6.42E-16 | Spats2 |
| 10607283 | 2.720355 | 6.836671 | -28.1595 | 4.21E-19 | 6.73E-16 | Maged2 |
| 10568328 | 2.062457 | 8.188703 | -27.9643 | 4.90E-19 | 7.43E-16 | Vkorc1 |
| 10403584 | 2.753346 | 7.70582 | -27.8373 | 5.42E-19 | 7.81E-16 | Nid1 |
| 10432404 | 1.491925 | 10.44383 | -27.7061 | 6.01E-19 | 8.25E-16 | Tuba1a |
| 10447649 | 3.941194 | 8.278973 | -27.308 | 8.26E-19 | 1.08E-15 | Fndc1 |
| 10423109 | 2.840482 | 6.324193 | -26.5503 | 1.53E-18 | 1.86E-15 | Adamts12 |
| 10470027 | 2.053999 | 7.029642 | -26.5356 | 1.55E-18 | 1.86E-15 | Npdc1 |
| 10391119 | 2.897201 | 6.480892 | -26.3738 | 1.77E-18 | 2.00E-15 | Leprel4 |
| 10539119 | 1.733934 | 6.644451 | -26.3116 | 1.87E-18 | 2.00E-15 | Ggcx |
| 10393823 | 1.524494 | 10.29094 | -26.3087 | 1.87E-18 | 2.00E-15 | P4hb |
| 10376778 | 3.712002 | 7.726169 | -25.9648 | 2.50E-18 | 2.57E-15 | Mfap4 |
| 10515007 | 2.922808 | 7.720402 | -25.8757 | 2.69E-18 | 2.68E-15 | Gpx7 |
| 10432243 | 2.531296 | 7.039179 | -25.708 | 3.11E-18 | 2.96E-15 | Fkbp11 |
| 10559790 | 3.936551 | 5.927593 | -25.678 | 3.19E-18 | 2.96E-15 | Zim1 |
| 10602068 | 2.616083 | 6.945654 | -25.5896 | 3.44E-18 | 3.09E-15 | Mid2 |
| 10578829 | 2.178944 | 7.606787 | -25.3644 | 4.17E-18 | 3.48E-15 | Palld |
| 10355403 | 2.958807 | 10.60352 | -25.3627 | 4.17E-18 | 3.48E-15 | Fn1 |
| 10485645 | 2.310485 | 7.760618 | -25.3117 | 4.36E-18 | 3.48E-15 | Rcn1 |
| 10509901 | 3.247812 | 8.053094 | -25.2889 | 4.45E-18 | 3.48E-15 | Mfap2 |
| 10412921 | 2.679731 | 6.875841 | -25.2825 | 4.47E-18 | 3.48E-15 | Nid2 |
| 10502522 | 1.663118 | 6.910426 | -25.2023 | 4.80E-18 | 3.64E-15 | Hs2st1 |
| 10473444 | 3.20017 | 7.429325 | -25.0706 | 5.38E-18 | 3.97E-15 | Aplnr |
| 10554249 | 5.826932 | 7.825654 | -25.0251 | 5.60E-18 | 4.03E-15 | Acan |
| 10529875 | 2.193752 | 7.388519 | -24.9078 | 6.20E-18 | 4.36E-15 | Ldb2 |
| 10395466 | 1.299693 | 6.112702 | -24.8554 | 6.49E-18 | 4.41E-15 | Dock4 |
| 10430358 | 3.112835 | 7.172123 | -24.834 | 6.62E-18 | 4.41E-15 | C1qtnf6 |
| 10459353 | 1.98642 | 5.64209 | -24.814 | 6.73E-18 | 4.41E-15 | Piezo2 |
| 10469322 | 1.028025 | 11.37379 | -24.7801 | 6.94E-18 | 4.44E-15 | Vim |
| 10519998 | 3.761326 | 7.3755 | -24.7018 | 7.43E-18 | 4.55E-15 | Lrrc17 |
| 10374366 | 2.605655 | 7.414424 | -24.6955 | 7.47E-18 | 4.55E-15 | Egfr |
| 10513208 | 2.618307 | 7.292337 | -24.6785 | 7.59E-18 | 4.55E-15 | Svep1 |
| 10594066 | 2.416144 | 8.184792 | -24.6539 | 7.75E-18 | 4.56E-15 | Loxl1 |
| 10484402 | 1.422333 | 7.818296 | -24.5995 | 8.14E-18 | 4.69E-15 | Ctnnd1 |
| 10496110 | 1.688796 | 6.142427 | -24.5434 | 8.55E-18 | 4.83E-15 | Papss1 |
| 10365420 | 1.938303 | 8.386934 | -24.5229 | 8.71E-18 | 4.83E-15 | Tmem263 |
| 10439218 | 2.097649 | 6.469793 | -24.4283 | 9.48E-18 | 5.15E-15 | Pdia5 |
| 10496727 | 4.31608 | 6.765258 | -24.3868 | 9.84E-18 | 5.17E-15 | Ddah1 |
| 10523511 | 2.806609 | 5.074553 | -24.3609 | 1.01E-17 | 5.17E-15 | Prkg2 |
| 10344837 | 1.48116 | 6.087324 | -24.3354 | 1.03E-17 | 5.17E-15 | Prex2 |
| 10568361 | 1.304317 | 7.551671 | -24.3138 | 1.05E-17 | 5.17E-15 | Yipf5 |
| 10493449 | 3.347487 | 6.74053 | -24.3048 | 1.06E-17 | 5.17E-15 | Thbs3 |
| 10527158 | 3.10479 | 7.089227 | -24.3047 | 1.06E-17 | 5.17E-15 | Fscn1 |
| 10428536 | 1.949088 | 7.828536 | -24.2823 | 1.08E-17 | 5.19E-15 | Trps1 |
| 10424140 | 3.730806 | 7.112141 | -24.2546 | 1.11E-17 | 5.23E-15 | Col14a1 |
| 10579860 | 1.563189 | 7.17218 | -24.2308 | 1.13E-17 | 5.23E-15 | Smad1 |
| 10492864 | 1.995488 | 7.084451 | -24.2199 | 1.14E-17 | 5.23E-15 | Sh3d19 |
| 10567355 | 1.963469 | 5.90419 | -24.1697 | 1.20E-17 | 5.38E-15 | Gprc5b |
| 10500948 | 1.4897 | 6.802514 | -24.1462 | 1.22E-17 | 5.41E-15 | Cttnbp2nl |
| 10547869 | 2.245217 | 7.102026 | -24.1148 | 1.26E-17 | 5.48E-15 | Leprel2 |
| 10351491 | 2.568675 | 8.038167 | -24.064 | 1.32E-17 | 5.66E-15 | Olfml2b |
| 10417095 | 2.102806 | 6.284338 | -24.0369 | 1.35E-17 | 5.66E-15 | Farp1 |
| 10439710 | 2.44375 | 7.38982 | -24.0301 | 1.36E-17 | 5.66E-15 | Phldb2 |
| 10485070 | 2.377291 | 7.231005 | -23.8938 | 1.54E-17 | 6.30E-15 | Mdk |
| 10576639 | 1.37243 | 7.72339 | -23.8811 | 1.55E-17 | 6.30E-15 | Nrp1 |
| 10519140 | 2.598357 | 7.49485 | -23.7849 | 1.70E-17 | 6.79E-15 | Mmp23 |
| 10412562 | 1.871263 | 7.208715 | -23.7311 | 1.78E-17 | 6.96E-15 | Flnb |
| 10421853 | 4.645502 | 6.746172 | -23.7268 | 1.79E-17 | 6.96E-15 | Lect1 |
| 10558345 | 1.370942 | 6.561955 | -23.6807 | 1.87E-17 | 7.17E-15 | Dock1 |
| 10440258 | 3.497627 | 6.676741 | -23.5716 | 2.06E-17 | 7.82E-15 | Epha3 |
| 10487645 | 2.78422 | 6.98137 | -23.4926 | 2.22E-17 | 8.23E-15 | Cpxm1 |
| 10460603 | 2.341436 | 8.555455 | -23.4888 | 2.23E-17 | 8.23E-15 | Efemp2 |
| 10449581 | 1.419974 | 8.918602 | -23.3634 | 2.50E-17 | 9.13E-15 | Mtch1 |
| 10544462 | 2.087572 | 5.979555 | -23.2789 | 2.71E-17 | 9.75E-15 | Fam115a |
| 10569335 | 4.385631 | 9.183715 | -23.2316 | 2.83E-17 | 1.01E-14 | H19 |
| 10588037 | 2.216989 | 8.502159 | -23.1735 | 2.99E-17 | 1.04E-14 | Rbp1 |
| 10566350 | -2.6871 | 5.74807 | 23.16716 | 3.01E-17 | 1.04E-14 | Trim30b |
| 10361771 | 3.321308 | 7.836522 | -23.1574 | 3.03E-17 | 1.04E-14 | Plagl1 |
| 10597413 | 2.588679 | 8.073353 | -23.1395 | 3.08E-17 | 1.05E-14 | Crtap |
| 10452508 | 1.584886 | 8.026685 | -23.1104 | 3.17E-17 | 1.06E-14 | Twsg1 |
| 10374083 | 3.636883 | 8.310328 | -23.0937 | 3.22E-17 | 1.06E-14 | Aebp1 |
| 10607143 | 4.6557 | 6.648813 | -23.091 | 3.23E-17 | 1.06E-14 | Capn6 |
| 10497548 | 2.030725 | 7.896027 | -22.9618 | 3.65E-17 | 1.17E-14 | Fndc3b |
| 10467258 | 2.362057 | 7.380104 | -22.9524 | 3.68E-17 | 1.17E-14 | Myof |
| 10553897 | 1.506575 | 6.116983 | -22.939 | 3.73E-17 | 1.17E-14 | Mtmr10 |
| 10425287 | 3.334168 | 6.849015 | -22.936 | 3.74E-17 | 1.17E-14 | Kdelr3 |
| 10542355 | 2.316639 | 8.832617 | -22.9051 | 3.85E-17 | 1.19E-14 | Emp1 |
| 10597531 | 1.986294 | 7.029174 | -22.6598 | 4.86E-17 | 1.49E-14 | Rbms3 |
| 10556426 | 2.604383 | 7.960708 | -22.5455 | 5.42E-17 | 1.65E-14 | Parva |
| 10417759 | 2.188544 | 6.743495 | -22.4972 | 5.68E-17 | 1.71E-14 | Ube2e2 |
| 10469906 | 1.628855 | 6.772518 | -22.4652 | 5.86E-17 | 1.74E-14 | Nsmf |
| 10529957 | 2.062782 | 6.604155 | -22.4329 | 6.05E-17 | 1.76E-14 | Gpr125 |
| 10554521 | 1.379988 | 7.083864 | -22.4204 | 6.12E-17 | 1.76E-14 | Pde8a |
| 10603896 | 2.923404 | 6.692058 | -22.4194 | 6.13E-17 | 1.76E-14 | Klhl13 |
| 10534168 | 2.294719 | 6.26714 | -22.4136 | 6.16E-17 | 1.76E-14 | Auts2 |
| 10476321 | 2.644718 | 6.015538 | -22.404 | 6.22E-17 | 1.76E-14 | Prn |
| 10585338 | 2.393251 | 6.079837 | -22.3533 | 6.53E-17 | 1.83E-14 | Kdelc2 |
| 10370180 | 3.153709 | 7.900371 | -22.333 | 6.66E-17 | 1.83E-14 | Col6a2 |
| 10435961 | 2.95798 | 6.141175 | -22.3295 | 6.68E-17 | 1.83E-14 | Gm10808 |
| 10447799 | 1.802763 | 7.37771 | -22.3237 | 6.72E-17 | 1.83E-14 | Igf2r |
| 10491970 | 1.645247 | 6.249565 | -22.3028 | 6.86E-17 | 1.85E-14 | Lhfp |
| 10531675 | 1.558834 | 9.159271 | -22.2053 | 7.54E-17 | 2.01E-14 | Sec31a |
| 10435641 | 3.44275 | 9.569125 | -22.1719 | 7.79E-17 | 2.06E-14 | Fstl1 |
| 10502748 | 1.605698 | 6.278362 | -22.1512 | 7.95E-17 | 2.06E-14 | Lphn2 |
| 10467124 | 3.325498 | 8.260275 | -22.1425 | 8.02E-17 | 2.06E-14 | Acta2 |
| 10549733 | 2.39502 | 6.620425 | -22.1418 | 8.02E-17 | 2.06E-14 | Ssc5d |
| 10594044 | 2.577029 | 8.654357 | -22.0054 | 9.17E-17 | 2.34E-14 | Islr |
| 10483381 | 2.161413 | 7.337708 | -21.9909 | 9.30E-17 | 2.35E-14 | Stk39 |
| 10455148 | 1.074482 | 5.646472 | -21.8299 | 1.09E-16 | 2.73E-14 | Pcdhgb1 |
| 10344981 | 2.440448 | 5.422235 | -21.8024 | 1.12E-16 | 2.78E-14 | Pi15 |
| 10530319 | -2.45221 | 7.834993 | 21.74538 | 1.19E-16 | 2.92E-14 | Atp8a1 |
| 10606609 | 3.559187 | 7.112537 | -21.7087 | 1.23E-16 | 3.00E-14 | Tspan6 |
| 10531588 | 2.450849 | 5.259922 | -21.689 | 1.25E-16 | 3.04E-14 | Prkg2 |
| 10569341 | 3.908011 | 8.086223 | -21.68 | 1.27E-16 | 3.04E-14 | H19 |
| 10551852 | 2.492455 | 7.195678 | -21.6493 | 1.31E-16 | 3.09E-14 | Clip3 |
| 10467319 | 3.295712 | 7.079719 | -21.6453 | 1.31E-16 | 3.09E-14 | Rbp4 |
| 10568668 | 2.989787 | 6.835696 | -21.6343 | 1.32E-16 | 3.10E-14 | Adam12 |
| 10545974 | 2.821185 | 8.6966 | -21.6186 | 1.35E-16 | 3.13E-14 | Antxr1 |
| 10590844 | 2.900795 | 6.362835 | -21.5884 | 1.39E-16 | 3.20E-14 | Arhgap42 |
| 10499394 | 1.475768 | 8.09524 | -21.5776 | 1.40E-16 | 3.21E-14 | Lmna |
| 10541094 | 1.192353 | 6.106793 | -21.5572 | 1.43E-16 | 3.25E-14 | Zfp637 |
| 10389816 | -1.63973 | 6.741842 | 21.45906 | 1.58E-16 | 3.56E-14 | Tom1l1 |
| 10507612 | 2.804088 | 7.490916 | -21.4444 | 1.60E-16 | 3.58E-14 | Lepre1 |
| 10456317 | 1.492573 | 6.320852 | -21.4218 | 1.64E-16 | 3.63E-14 | Zfp532 |
| 10414065 | 3.431849 | 6.837781 | -21.3858 | 1.70E-16 | 3.74E-14 | Anxa8 |
| 10350864 | 2.349824 | 6.207381 | -21.3648 | 1.74E-16 | 3.75E-14 | Sec16b |
| 10493245 | 1.971612 | 5.58679 | -21.3648 | 1.74E-16 | 3.75E-14 | Mex3a |
| 10474619 | 1.65111 | 6.662142 | -21.3611 | 1.74E-16 | 3.75E-14 | Fmn1 |
| 10494445 | 2.259369 | 7.231791 | -21.305 | 1.85E-16 | 3.94E-14 | Lix1l |
| 10353524 | -1.5882 | 7.214604 | 21.28251 | 1.89E-16 | 3.96E-14 | Ogfrl1 |
| 10496359 | 2.465205 | 7.665628 | -21.2807 | 1.89E-16 | 3.96E-14 | Emcn |
| 10497689 | 1.499393 | 6.386092 | -21.2766 | 1.90E-16 | 3.96E-14 | Gnb4 |
| 10459481 | 1.861592 | 8.299217 | -21.2641 | 1.92E-16 | 3.96E-14 | Lman1 |
| 10415052 | 3.211437 | 9.027889 | -21.2638 | 1.92E-16 | 3.96E-14 | Mmp14 |
| 10474419 | 2.701322 | 6.33789 | -21.1998 | 2.05E-16 | 4.17E-14 | Lgr4 |
| 10492355 | 2.547247 | 7.5543 | -21.175 | 2.11E-16 | 4.24E-14 | Mme |
| 10378334 | 1.73855 | 7.602653 | -21.1318 | 2.20E-16 | 4.40E-14 | Tax1bp3 |
| 10346348 | 1.703242 | 6.276739 | -21.0882 | 2.30E-16 | 4.55E-14 | Spats2l |
| 10408280 | 2.021784 | 6.387737 | -21.0856 | 2.31E-16 | 4.55E-14 | Lrrc16a |
| 10537146 | 2.719242 | 6.078339 | -20.9795 | 2.57E-16 | 5.04E-14 | Akr1b8 |
| 10409464 | 1.745118 | 6.68591 | -20.9394 | 2.68E-16 | 5.22E-14 | Dbn1 |
| 10421309 | 2.399198 | 6.54458 | -20.9239 | 2.72E-16 | 5.23E-14 | Slc39a14 |
| 10396170 | 1.895514 | 7.03601 | -20.9157 | 2.75E-16 | 5.23E-14 | Frmd6 |
| 10460541 | 2.954696 | 8.019841 | -20.9084 | 2.77E-16 | 5.23E-14 | Cd248 |
| 10355050 | 2.027289 | 7.954597 | -20.9046 | 2.78E-16 | 5.23E-14 | Raph1 |
| 10456400 | 2.656096 | 7.894075 | -20.8987 | 2.79E-16 | 5.23E-14 | Tubb6 |
| 10558961 | 1.829958 | 7.415628 | -20.8921 | 2.81E-16 | 5.23E-14 | Tspan4 |
| 10472440 | 1.768044 | 7.602722 | -20.8831 | 2.84E-16 | 5.25E-14 | Tax1bp3 |
| 10566502 | 1.278341 | 7.687849 | -20.871 | 2.88E-16 | 5.28E-14 | Arfip2 |
| 10501802 | -2.59425 | 5.738118 | 20.86392 | 2.90E-16 | 5.28E-14 | Tmem56 |
| 10459335 | 2.555191 | 5.655696 | -20.8265 | 3.01E-16 | 5.46E-14 | Piezo2 |
| 10548879 | 1.996942 | 11.26574 | -20.7804 | 3.16E-16 | 5.68E-14 | Mgp |
| 10473432 | 1.511104 | 6.189777 | -20.7726 | 3.18E-16 | 5.68E-14 | Tnks1bp1 |
| 10418171 | 1.674775 | 7.249387 | -20.7697 | 3.19E-16 | 5.68E-14 | Zcchc24 |
| 10453178 | 1.981492 | 7.30969 | -20.7598 | 3.23E-16 | 5.70E-14 | Map4k3 |
| 10474361 | 2.384042 | 6.20102 | -20.74 | 3.29E-16 | 5.79E-14 | Mpped2 |
| 10546567 | 1.750421 | 6.858266 | -20.7322 | 3.32E-16 | 5.80E-14 | Eogt |
| 10429114 | -2.29381 | 5.857132 | 20.66807 | 3.55E-16 | 6.15E-14 | Tmem71 |
| 10344990 | 2.418036 | 6.25178 | -20.6638 | 3.56E-16 | 6.15E-14 | Crispld1 |
| 10422728 | 2.474873 | 9.015523 | -20.6481 | 3.62E-16 | 6.21E-14 | Dab2 |
| 10590325 | 1.190515 | 9.607777 | -20.6374 | 3.66E-16 | 6.25E-14 | Ctnnb1 |
| 10586781 | 2.211664 | 7.216598 | -20.6075 | 3.78E-16 | 6.41E-14 | Myo1e |
| 10586933 | 1.395575 | 10.42185 | -20.5938 | 3.83E-16 | 6.46E-14 | Nedd4 |
| 10468309 | 2.414898 | 8.653317 | -20.557 | 3.98E-16 | 6.67E-14 | Sh3pxd2a |
| 10498284 | 2.299111 | 7.962428 | -20.5218 | 4.13E-16 | 6.89E-14 | Wwtr1 |
| 10574384 | 1.485049 | 6.13443 | -20.498 | 4.24E-16 | 7.02E-14 | Ndrg4 |
| 10484927 | 1.976996 | 7.706056 | -20.4341 | 4.53E-16 | 7.46E-14 | Slc39a13 |
| 10544875 | 1.757983 | 5.758335 | -20.3644 | 4.88E-16 | 7.99E-14 | Scrn1 |
| 10586907 | -2.33148 | 6.387246 | 20.32864 | 5.07E-16 | 8.25E-14 | Mns1 |
| 10605766 | 2.805588 | 8.74227 | -20.3042 | 5.20E-16 | 8.42E-14 | Maged1 |
| 10394488 | 5.149626 | 7.068824 | -20.2489 | 5.51E-16 | 8.87E-14 | Matn3 |
| 10379262 | 1.284164 | 7.927858 | -20.2224 | 5.67E-16 | 9.07E-14 | Nf1 |
| 10464754 | 1.829493 | 6.237568 | -20.1875 | 5.88E-16 | 9.37E-14 | Rhod |
| 10471929 | -2.43704 | 8.042733 | 20.17461 | 5.96E-16 | 9.44E-14 | Arhgap15 |
| 10531560 | 1.374643 | 7.711879 | -20.1247 | 6.29E-16 | 9.79E-14 | Antxr2 |
| 10570483 | 1.52796 | 5.798323 | -20.1211 | 6.31E-16 | 9.79E-14 | Arhgef10 |
| 10499189 | 2.406841 | 5.572475 | -20.1206 | 6.32E-16 | 9.79E-14 | Fcrls |
| 10564343 | 1.886106 | 7.650574 | -20.1179 | 6.34E-16 | 9.79E-14 | Tjp1 |
| 10495651 | 1.627807 | 6.987503 | -20.1149 | 6.36E-16 | 9.79E-14 | Alg14 |
| 10427744 | 1.882484 | 7.418839 | -20.0996 | 6.46E-16 | 9.90E-14 | Rai14 |
| 10605919 | 1.419494 | 7.62879 | -20.0622 | 6.72E-16 | 1.03E-13 | Pja1 |
| 10598976 | 3.591769 | 9.168447 | -20.0337 | 6.93E-16 | 1.05E-13 | Timp1 |
| 10466888 | 1.995687 | 6.372406 | -20.0137 | 7.08E-16 | 1.07E-13 | Glis3 |
| 10578222 | 1.615144 | 7.099285 | -20.0054 | 7.15E-16 | 1.07E-13 | Dlc1 |
| 10367282 | 1.520493 | 7.161331 | -19.9651 | 7.46E-16 | 1.11E-13 | Cnpy2 |
| 10423599 | 3.421569 | 7.295873 | -19.9615 | 7.49E-16 | 1.11E-13 | Matn2 |
| 10501762 | 2.958213 | 7.422188 | -19.9423 | 7.65E-16 | 1.13E-13 | Snx7 |
| 10487040 | 2.990989 | 8.113086 | -19.9292 | 7.75E-16 | 1.14E-13 | Fbn1 |
| 10527936 | 1.811741 | 6.867681 | -19.9242 | 7.80E-16 | 1.14E-13 | Fzd1 |
| 10490802 | 1.531046 | 5.859059 | -19.9132 | 7.89E-16 | 1.15E-13 | Zc2hc1a |
| 10595560 | 1.664884 | 5.563182 | -19.853 | 8.42E-16 | 1.22E-13 | Tbx18 |
| 10470834 | 1.05813 | 8.309016 | -19.8355 | 8.58E-16 | 1.23E-13 | Sptan1 |
| 10578623 | 1.280644 | 6.538467 | -19.8328 | 8.60E-16 | 1.23E-13 | Wwc2 |
| 10458999 | 3.292168 | 6.491563 | -19.7865 | 9.05E-16 | 1.29E-13 | Fbn2 |
| 10505954 | 1.441548 | 6.380672 | -19.7749 | 9.16E-16 | 1.30E-13 | Tek |
| 10458303 | 2.067517 | 7.871187 | -19.7322 | 9.59E-16 | 1.36E-13 | Ecscr |
| 10471535 | 1.998748 | 6.618794 | -19.7032 | 9.90E-16 | 1.39E-13 | Fam129b |
| 10535956 | 1.621963 | 5.755504 | -19.6304 | 1.07E-15 | 1.50E-13 | Stard13 |
| 10574438 | 2.4004 | 8.16708 | -19.6206 | 1.08E-15 | 1.51E-13 | Cdh5 |
| 10531177 | 2.797799 | 6.273001 | -19.6075 | 1.10E-15 | 1.52E-13 | Adamts3 |
| 10534935 | -2.38153 | 7.45926 | 19.59092 | 1.12E-15 | 1.54E-13 | Pilrb1 |
| 10436372 | 1.688469 | 6.247997 | -19.5875 | 1.12E-15 | 1.54E-13 | Dcbld2 |
| 10530287 | 1.711142 | 7.082297 | -19.5691 | 1.15E-15 | 1.56E-13 | Apbb2 |
| 10347980 | 1.380047 | 8.207499 | -19.5627 | 1.15E-15 | 1.57E-13 | Itm2c |
| 10568536 | 3.294651 | 7.01823 | -19.551 | 1.17E-15 | 1.58E-13 | Cpxm2 |
| 10356800 | 1.304645 | 10.15551 | -19.5364 | 1.19E-15 | 1.60E-13 | Hdlbp |
| 10559796 | 4.315385 | 7.794305 | -19.533 | 1.19E-15 | 1.60E-13 | Peg3 |
| 10548701 | 1.374851 | 7.743341 | -19.5295 | 1.20E-15 | 1.60E-13 | Lrp6 |
| 10403727 | 2.060052 | 6.274084 | -19.4996 | 1.24E-15 | 1.64E-13 | Gli3 |
| 10578810 | -2.36954 | 6.68423 | 19.49353 | 1.24E-15 | 1.64E-13 | Clcn3 |
| 10468527 | -1.79549 | 5.972142 | 19.49338 | 1.25E-15 | 1.64E-13 | 5830416P10Rik |
| 10571467 | 3.449048 | 7.049885 | -19.4579 | 1.29E-15 | 1.69E-13 | Pdgfrl |
| 10363512 | 1.333716 | 9.989637 | -19.443 | 1.32E-15 | 1.71E-13 | Sar1a |
| 10585860 | -2.03787 | 7.795492 | 19.43749 | 1.32E-15 | 1.71E-13 | Adpgk |
| 10407126 | 2.268268 | 6.701349 | -19.4198 | 1.35E-15 | 1.74E-13 | Plk2 |
| 10393887 | 2.121629 | 6.506478 | -19.405 | 1.37E-15 | 1.76E-13 | Pycr1 |
| 10560190 | 2.317971 | 7.189 | -19.3868 | 1.40E-15 | 1.79E-13 | Ehd2 |
| 10424119 | 3.237047 | 7.030886 | -19.3354 | 1.48E-15 | 1.88E-13 | Nov |
| 10538547 | 2.856816 | 8.039208 | -19.3277 | 1.49E-15 | 1.89E-13 | Fkbp9 |
| 10598403 | 2.707822 | 8.470582 | -19.3204 | 1.51E-15 | 1.89E-13 | Praf2 |
| 10371230 | 1.596717 | 7.847503 | -19.3142 | 1.52E-15 | 1.89E-13 | Gna11 |
| 10359754 | 1.706084 | 7.214464 | -19.3132 | 1.52E-15 | 1.89E-13 | Mpzl1 |
| 10366446 | -3.07585 | 7.819554 | 19.29987 | 1.54E-15 | 1.91E-13 | Tspan8 |
| 10391130 | 1.105072 | 7.326188 | -19.2884 | 1.56E-15 | 1.93E-13 | Nt5c3b |
| 10487208 | -2.98961 | 7.251871 | 19.28558 | 1.57E-15 | 1.93E-13 | Atp8b4 |
| 10550274 | 1.305022 | 6.156205 | -19.2549 | 1.62E-15 | 1.98E-13 | Meis3 |
| 10507273 | 2.145978 | 6.40969 | -19.2512 | 1.63E-15 | 1.98E-13 | Pik3r3 |
| 10498576 | 2.410022 | 7.272273 | -19.2503 | 1.63E-15 | 1.98E-13 | Lxn |
| 10432540 | 1.687261 | 7.0305 | -19.2407 | 1.65E-15 | 1.99E-13 | Lima1 |
| 10530841 | 2.182361 | 10.11351 | -19.2363 | 1.65E-15 | 1.99E-13 | Igfbp7 |
| 10473281 | 1.852244 | 8.281391 | -19.2305 | 1.66E-15 | 2.00E-13 | Itgav |
| 10363887 | 1.386614 | 4.730297 | -19.2162 | 1.69E-15 | 2.01E-13 | LOC101056131 |
| 10505008 | 1.426514 | 8.467076 | -19.2129 | 1.70E-15 | 2.01E-13 | Slc44a1 |
| 10563712 | -3.03705 | 7.38239 | 19.20918 | 1.70E-15 | 2.01E-13 | Mrgpra2a |
| 10445774 | -2.6931 | 7.677949 | 19.20634 | 1.71E-15 | 2.01E-13 | B430306N03Rik |
| 10444665 | 1.378157 | 7.599887 | -19.2052 | 1.71E-15 | 2.01E-13 | Ddah2 |
| 10470462 | 2.218719 | 7.079941 | -19.1998 | 1.72E-15 | 2.02E-13 | Col5a1 |
| 10500666 | 2.218995 | 6.573091 | -19.185 | 1.75E-15 | 2.04E-13 | Ptgfrn |
| 10430145 | 2.129764 | 7.042386 | -19.1768 | 1.77E-15 | 2.05E-13 | Rbfox2 |
| 10556509 | 1.989023 | 6.501089 | -19.1606 | 1.80E-15 | 2.08E-13 | Spon1 |
| 10356520 | 3.387561 | 8.702503 | -19.1589 | 1.80E-15 | 2.08E-13 | Col6a3 |
| 10584674 | 2.088797 | 7.599352 | -19.1374 | 1.85E-15 | 2.12E-13 | Mcam |
| 10356084 | 2.526348 | 8.155288 | -19.1332 | 1.85E-15 | 2.12E-13 | Irs1 |
| 10372965 | -1.47861 | 8.657169 | 19.12715 | 1.87E-15 | 2.13E-13 | Usp15 |
| 10422164 | 1.982658 | 5.995739 | -19.1213 | 1.88E-15 | 2.13E-13 | Ednrb |
| 10354432 | 2.580484 | 7.798167 | -19.1191 | 1.88E-15 | 2.13E-13 | Myo1b |
| 10410931 | 3.632566 | 7.924275 | -19.0914 | 1.94E-15 | 2.19E-13 | Vcan |
| 10379044 | 2.180768 | 6.650241 | -19.0781 | 1.97E-15 | 2.21E-13 | Rab34 |
| 10575702 | 5.49926 | 7.756997 | -19.0544 | 2.03E-15 | 2.26E-13 | Clec3a |
| 10585467 | 1.231325 | 5.358237 | -19.0231 | 2.10E-15 | 2.33E-13 | Hykk |
| 10418506 | 1.364756 | 6.279079 | -18.9962 | 2.16E-15 | 2.39E-13 | Stab1 |
| 10400515 | 1.774611 | 8.108384 | -18.9951 | 2.16E-15 | 2.39E-13 | Sec23a |
| 10509246 | 1.789512 | 7.308123 | -18.9884 | 2.18E-15 | 2.40E-13 | Luzp1 |
| 10535902 | -1.64244 | 3.97256 | 18.98431 | 2.19E-15 | 2.40E-13 | n-R5s180 |
| 10434105 | 1.848274 | 6.748926 | -18.9598 | 2.25E-15 | 2.46E-13 | Scarf2 |
| 10537184 | 1.518621 | 8.428571 | -18.9316 | 2.32E-15 | 2.53E-13 | Cald1 |
| 10365716 | 2.276953 | 7.53827 | -18.9196 | 2.36E-15 | 2.55E-13 | Ikbip |
| 10493692 | 1.271588 | 6.663946 | -18.9143 | 2.37E-15 | 2.56E-13 | Rab13 |
| 10586744 | 1.177703 | 9.251695 | -18.8914 | 2.43E-15 | 2.61E-13 | Anxa2 |
| 10505172 | 1.480652 | 6.48813 | -18.8888 | 2.44E-15 | 2.61E-13 | Dnajc25 |
| 10588007 | -2.06596 | 8.46162 | 18.86918 | 2.49E-15 | 2.66E-13 | Tfdp2 |
| 10461423 | 1.739745 | 7.315856 | -18.8633 | 2.51E-15 | 2.67E-13 | Fads3 |
| 10393379 | 2.030541 | 6.834141 | -18.8541 | 2.54E-15 | 2.69E-13 | Mxra7 |
| 10525989 | 1.945303 | 6.616972 | -18.8447 | 2.56E-15 | 2.71E-13 | Gpr133 |
| 10436978 | 1.865967 | 6.545723 | -18.8222 | 2.63E-15 | 2.77E-13 | Cbr3 |
| 10370210 | 3.161617 | 8.070359 | -18.7974 | 2.71E-15 | 2.83E-13 | Col6a1 |
| 10498519 | 1.096204 | 9.965818 | -18.7704 | 2.79E-15 | 2.91E-13 | Ssr3 |
| 10563715 | -2.87656 | 7.184553 | 18.76496 | 2.81E-15 | 2.92E-13 | Mrgpra2a |
| 10385635 | 2.38318 | 7.429677 | -18.745 | 2.87E-15 | 2.98E-13 | Zfp354c |
| 10368886 | -1.54005 | 7.703221 | 18.72325 | 2.94E-15 | 3.04E-13 | Foxo3 |
| 10416989 | 1.445175 | 6.872341 | -18.7055 | 3.00E-15 | 3.09E-13 | Gpr180 |
| 10369844 | 2.147827 | 8.29257 | -18.6936 | 3.04E-15 | 3.10E-13 | Bicc1 |
| 10346747 | 1.533453 | 7.039778 | -18.6924 | 3.05E-15 | 3.10E-13 | Cyp20a1 |
| 10458894 | 3.54484 | 8.722229 | -18.6924 | 3.05E-15 | 3.10E-13 | Lox |
| 10401852 | -2.05276 | 7.063204 | 18.69045 | 3.06E-15 | 3.10E-13 | Cep128 |
| 10371959 | 1.705156 | 8.11324 | -18.6867 | 3.07E-15 | 3.10E-13 | Elk3 |
| 10584628 | 2.309317 | 8.863217 | -18.6782 | 3.10E-15 | 3.12E-13 | Thy1 |
| 10435271 | 1.722748 | 8.385076 | -18.6571 | 3.17E-15 | 3.19E-13 | Heg1 |
| 10373340 | 1.244813 | 7.53482 | -18.6528 | 3.19E-15 | 3.19E-13 | Rbms2 |
| 10560282 | 1.393956 | 7.411342 | -18.6468 | 3.21E-15 | 3.20E-13 | Arhgap35 |
| 10597960 | -2.36521 | 6.003459 | 18.63783 | 3.24E-15 | 3.22E-13 | Slc6a20a |
| 10540085 | 2.480864 | 7.439366 | -18.6321 | 3.27E-15 | 3.23E-13 | Fbln2 |
| 10505623 | 2.184366 | 5.74945 | -18.6127 | 3.34E-15 | 3.28E-13 | Lurap1l |
| 10396476 | 1.750074 | 7.731091 | -18.6122 | 3.34E-15 | 3.28E-13 | Rhoj |
| 10350297 | -2.00875 | 6.92679 | 18.6112 | 3.34E-15 | 3.28E-13 | Kif14 |
| 10527940 | 1.954262 | 6.573426 | -18.6069 | 3.36E-15 | 3.28E-13 | Cdk14 |
| 10415396 | 1.624678 | 5.658294 | -18.5921 | 3.42E-15 | 3.33E-13 | Nfatc4 |
| 10591614 | 2.572912 | 7.476621 | -18.5892 | 3.43E-15 | 3.33E-13 | Dock6 |
| 10518408 | 2.286126 | 7.334457 | -18.5626 | 3.54E-15 | 3.42E-13 | Plod1 |
| 10375002 | -1.40198 | 8.505196 | 18.54169 | 3.62E-15 | 3.49E-13 | Cpeb4 |
| 10421361 | 2.418125 | 7.818905 | -18.5322 | 3.66E-15 | 3.52E-13 | Bmp1 |
| 10413304 | 1.635443 | 9.42601 | -18.5255 | 3.69E-15 | 3.53E-13 | Arf4 |
| 10381603 | 1.938692 | 6.516735 | -18.4957 | 3.82E-15 | 3.63E-13 | Fzd2 |
| 10498998 | -2.29535 | 7.690309 | 18.49505 | 3.82E-15 | 3.63E-13 | D930015E06Rik |
| 10428534 | 2.474802 | 8.268414 | -18.4911 | 3.84E-15 | 3.64E-13 | Trps1 |
| 10529485 | 2.17644 | 6.743926 | -18.4542 | 4.00E-15 | 3.78E-13 | Htra3 |
| 10581175 | 1.3413 | 6.750114 | -18.4377 | 4.08E-15 | 3.84E-13 | B3gnt9 |
| 10603492 | 1.800201 | 6.814919 | -18.4278 | 4.13E-15 | 3.87E-13 | Porcn |
| 10594679 | 1.458745 | 6.201388 | -18.4105 | 4.21E-15 | 3.94E-13 | Tln2 |
| 10459288 | -2.66416 | 7.310397 | 18.38112 | 4.36E-15 | 4.06E-13 | Adrb2 |
| 10566454 | 1.92135 | 8.00152 | -18.3585 | 4.47E-15 | 4.14E-13 | Prkcdbp |
| 10584803 | 1.031072 | 7.561942 | -18.3555 | 4.49E-15 | 4.14E-13 | Ift46 |
| 10587023 | -2.41577 | 6.879539 | 18.34469 | 4.54E-15 | 4.18E-13 | Rab27a |
| 10375065 | 2.225722 | 6.649779 | -18.3322 | 4.61E-15 | 4.23E-13 | Sh3pxd2b |
| 10381298 | 1.822837 | 6.583135 | -18.3053 | 4.76E-15 | 4.35E-13 | Ramp2 |
| 10366951 | 3.141837 | 6.909364 | -18.2984 | 4.79E-15 | 4.37E-13 | Ndufa4l2 |
| 10514590 | 1.709884 | 7.52277 | -18.291 | 4.84E-15 | 4.40E-13 | Dock7 |
| 10495054 | 1.998559 | 7.522423 | -18.2809 | 4.89E-15 | 4.43E-13 | Rhoc |
| 10409376 | -1.94147 | 5.895984 | 18.27268 | 4.94E-15 | 4.46E-13 | Hk3 |
| 10436519 | 2.499345 | 6.69973 | -18.2461 | 5.10E-15 | 4.59E-13 | Robo1 |
| 10569344 | 2.867309 | 7.694462 | -18.2303 | 5.19E-15 | 4.66E-13 | Igf2 |
| 10522060 | 2.242094 | 8.249389 | -18.2281 | 5.20E-15 | 4.66E-13 | Fam114a1 |
| 10362245 | 1.647488 | 8.484364 | -18.2246 | 5.22E-15 | 4.66E-13 | Epb4.1l2 |
| 10575019 | 2.419266 | 5.761811 | -18.2227 | 5.24E-15 | 4.66E-13 | Gm10629 |
| 10519196 | 1.535264 | 6.012736 | -18.2126 | 5.30E-15 | 4.70E-13 | Vwa1 |
| 10387699 | -2.36642 | 6.31993 | 18.20584 | 5.34E-15 | 4.72E-13 | Acap1 |
| 10492682 | 2.395722 | 6.956428 | -18.1911 | 5.43E-15 | 4.79E-13 | Fam198b |
| 10583669 | -1.95608 | 8.336555 | 18.17504 | 5.54E-15 | 4.86E-13 | AB124611 |
| 10360806 | 1.383335 | 8.202838 | -18.1468 | 5.72E-15 | 5.01E-13 | Capn2 |
| 10571774 | 1.159491 | 7.537586 | -18.1319 | 5.82E-15 | 5.08E-13 | Aga |
| 10588049 | 1.108443 | 9.029165 | -18.1213 | 5.89E-15 | 5.13E-13 | Copb2 |
| 10381122 | 3.402316 | 7.729565 | -18.1155 | 5.93E-15 | 5.15E-13 | Fkbp10 |
| 10512949 | 1.318595 | 7.294325 | -18.1093 | 5.98E-15 | 5.16E-13 | Abca1 |
| 10491952 | -2.77428 | 7.658302 | 18.10896 | 5.98E-15 | 5.16E-13 | Mgst2 |
| 10499168 | 1.856674 | 6.839167 | -18.1049 | 6.01E-15 | 5.17E-13 | Kirrel |
| 10606369 | 4.018021 | 9.428612 | -18.0914 | 6.10E-15 | 5.23E-13 | Itm2a |
| 10513362 | -1.88429 | 6.235556 | 18.08979 | 6.12E-15 | 5.23E-13 | Susd1 |
| 10456018 | 2.895565 | 5.978711 | -18.0706 | 6.25E-15 | 5.33E-13 | Arsi |
| 10534862 | 2.493592 | 10.27665 | -18.0655 | 6.29E-15 | 5.35E-13 | Pcolce |
| 10472350 | -3.11666 | 6.533129 | 18.04515 | 6.44E-15 | 5.45E-13 | Gca |
| 10605143 | -2.36462 | 6.655295 | 18.04379 | 6.45E-15 | 5.45E-13 | Arhgap4 |
| 10375079 | 1.810829 | 7.566822 | -18.0407 | 6.48E-15 | 5.46E-13 | Ubtd2 |
| 10383025 | 2.12082 | 6.800325 | -18.0336 | 6.53E-15 | 5.49E-13 | C1qtnf1 |
| 10359235 | 1.908271 | 6.509148 | -18.0313 | 6.55E-15 | 5.49E-13 | Rasal2 |
| 10345824 | -2.46314 | 6.375222 | 18.02723 | 6.58E-15 | 5.50E-13 | Il18rap |
| 10387659 | 1.429092 | 6.701985 | -18.0083 | 6.73E-15 | 5.60E-13 | Nlgn2 |
| 10345921 | 3.286476 | 9.608272 | -18.007 | 6.74E-15 | 5.60E-13 | 1500015O10Rik |
| 10370259 | 1.560773 | 6.030393 | -18.0018 | 6.78E-15 | 5.62E-13 | Col18a1 |
| 10385966 | 1.263947 | 8.959257 | -17.997 | 6.82E-15 | 5.62E-13 | Anxa6 |
| 10565255 | 2.061435 | 6.098853 | -17.9944 | 6.84E-15 | 5.62E-13 | Cemip |
| 10581271 | 1.223641 | 6.396428 | -17.9926 | 6.86E-15 | 5.62E-13 | Zdhhc1 |
| 10481592 | 2.433087 | 7.494791 | -17.992 | 6.86E-15 | 5.62E-13 | Dnm1 |
| 10591563 | 1.415612 | 6.56371 | -17.9705 | 7.04E-15 | 5.74E-13 | Kank2 |
| 10357472 | -1.81798 | 9.548541 | 17.96492 | 7.08E-15 | 5.77E-13 | Cxcr4 |
| 10582295 | -1.02253 | 9.880536 | 17.95051 | 7.20E-15 | 5.85E-13 | Odc1 |
| 10374248 | -2.82205 | 6.248076 | 17.94644 | 7.24E-15 | 5.86E-13 | Abca13 |
| 10578493 | 1.395324 | 5.675437 | -17.9408 | 7.29E-15 | 5.88E-13 | Tlr3 |
| 10542522 | 1.376258 | 6.406542 | -17.9387 | 7.31E-15 | 5.88E-13 | Plekha5 |
| 10365559 | 2.57854 | 9.016528 | -17.9278 | 7.40E-15 | 5.94E-13 | Igf1 |
| 10541496 | 3.544077 | 9.166189 | -17.9252 | 7.42E-15 | 5.94E-13 | Mfap5 |
| 10447602 | -1.50077 | 7.628352 | 17.91775 | 7.49E-15 | 5.98E-13 | Ezr |
| 10346321 | 3.180217 | 6.208709 | -17.9135 | 7.53E-15 | 5.99E-13 | Gm10561 |
| 10472212 | 1.000879 | 6.912767 | -17.901 | 7.64E-15 | 6.06E-13 | Pkp4 |
| 10393559 | 1.932297 | 9.644904 | -17.8798 | 7.83E-15 | 6.20E-13 | Timp2 |
| 10511368 | 1.367762 | 7.277097 | -17.8743 | 7.88E-15 | 6.22E-13 | Impad1 |
| 10549222 | 1.899853 | 6.366348 | -17.8656 | 7.97E-15 | 6.26E-13 | Bcat1 |
| 10396068 | -1.70391 | 5.840836 | 17.86518 | 7.97E-15 | 6.26E-13 | Lrr1 |
| 10495035 | -1.65932 | 7.335259 | 17.86308 | 7.99E-15 | 6.26E-13 | Slc16a1 |
| 10503188 | -1.53665 | 7.127534 | 17.84188 | 8.19E-15 | 6.40E-13 | Chd7 |
| 10543686 | -1.1865 | 7.85515 | 17.81751 | 8.43E-15 | 6.56E-13 | Ube2h |
| 10428579 | 2.053469 | 8.013123 | -17.8162 | 8.45E-15 | 6.56E-13 | Ext1 |
| 10573924 | 3.129451 | 9.476014 | -17.7926 | 8.69E-15 | 6.69E-13 | Mmp2 |
| 10440091 | 2.82876 | 8.325437 | -17.7888 | 8.73E-15 | 6.69E-13 | Col8a1 |
| 10402783 | 2.022859 | 6.802541 | -17.7887 | 8.73E-15 | 6.69E-13 | Ahnak2 |
| 10394674 | 2.269855 | 7.26441 | -17.7832 | 8.78E-15 | 6.71E-13 | Socs2 |
| 10511835 | 1.29332 | 4.624313 | -17.7729 | 8.89E-15 | 6.78E-13 | Fhl5 |
| 10529656 | 1.756826 | 6.995113 | -17.7602 | 9.03E-15 | 6.86E-13 | Nsg1 |
| 10603583 | 2.560367 | 6.79501 | -17.7509 | 9.13E-15 | 6.92E-13 | Srpx |
| 10605542 | -2.22696 | 4.416648 | 17.72614 | 9.40E-15 | 7.11E-13 | Mageb16 |
| 10509122 | -1.98268 | 5.868982 | 17.71566 | 9.52E-15 | 7.17E-13 | Cnr2 |
| 10452613 | 1.862815 | 5.297089 | -17.7149 | 9.53E-15 | 7.17E-13 | Arhgap28 |
| 10472965 | 1.864506 | 5.972635 | -17.6986 | 9.72E-15 | 7.29E-13 | Hoxd8 |
| 10537179 | -3.09003 | 8.5922 | 17.69538 | 9.75E-15 | 7.29E-13 | Bpgm |
| 10462822 | -1.58696 | 8.029729 | 17.69365 | 9.77E-15 | 7.29E-13 | Exoc6 |
| 10360745 | -2.272 | 8.139993 | 17.69267 | 9.79E-15 | 7.29E-13 | Lbr |
| 10440376 | 2.482341 | 4.640504 | -17.6778 | 9.96E-15 | 7.40E-13 | Lipi |
| 10361186 | 2.809927 | 6.8911 | -17.6685 | 1.01E-14 | 7.46E-13 | Sertad4 |
| 10403466 | 1.486976 | 6.179526 | -17.6619 | 1.02E-14 | 7.50E-13 | Dip2c |
| 10433088 | -1.1649 | 8.361459 | 17.63746 | 1.05E-14 | 7.70E-13 | Cbx5 |
| 10487021 | 2.105635 | 6.986355 | -17.6335 | 1.05E-14 | 7.72E-13 | Slc30a4 |
| 10594825 | -2.3932 | 6.114882 | 17.62086 | 1.07E-14 | 7.82E-13 | Aqp9 |
| 10582809 | -1.43283 | 7.665818 | 17.60094 | 1.09E-14 | 7.99E-13 | Tk1 |
| 10521824 | 1.67087 | 6.213944 | -17.5936 | 1.10E-14 | 8.04E-13 | Sod3 |
| 10570397 | 1.323342 | 7.540761 | -17.5578 | 1.15E-14 | 8.37E-13 | Cdc16 |
| 10383564 | -2.15673 | 6.007436 | 17.55455 | 1.15E-14 | 8.38E-13 | Fn3k |
| 10585545 | 1.317343 | 6.382513 | -17.542 | 1.17E-14 | 8.47E-13 | Rcn2 |
| 10396306 | 1.259845 | 7.091147 | -17.5391 | 1.18E-14 | 8.47E-13 | Jkamp |
| 10371888 | -1.40306 | 7.691634 | 17.53265 | 1.19E-14 | 8.52E-13 | Tmpo |
| 10380699 | 2.654405 | 8.50547 | -17.5289 | 1.19E-14 | 8.54E-13 | Copz2 |
| 10422962 | -1.58599 | 7.131925 | 17.50659 | 1.22E-14 | 8.74E-13 | Nadk2 |
| 10548163 | 1.302393 | 5.955717 | -17.5056 | 1.22E-14 | 8.74E-13 | Tulp3 |
| 10435948 | 3.291885 | 8.7709 | -17.5028 | 1.23E-14 | 8.74E-13 | Ccdc80 |
| 10490159 | 2.474621 | 8.613119 | -17.4878 | 1.25E-14 | 8.88E-13 | Pmepa1 |
| 10603551 | -2.38303 | 9.238978 | 17.48264 | 1.26E-14 | 8.92E-13 | Cybb |
| 10542691 | -2.3089 | 6.780778 | 17.47634 | 1.27E-14 | 8.96E-13 | Lrmp |
| 10586079 | 2.712451 | 7.142986 | -17.4647 | 1.29E-14 | 9.07E-13 | Itga11 |
| 10571530 | 2.490822 | 7.785087 | -17.4623 | 1.29E-14 | 9.07E-13 | Fat1 |
| 10447190 | 1.373154 | 6.270138 | -17.4556 | 1.30E-14 | 9.12E-13 | Plekhh2 |
| 10458906 | 2.716549 | 9.983216 | -17.4226 | 1.35E-14 | 9.45E-13 | Ppic |
| 10444016 | -2.93249 | 7.517062 | 17.41164 | 1.37E-14 | 9.55E-13 | Pram1 |
| 10495659 | 2.148758 | 7.508458 | -17.3859 | 1.42E-14 | 9.81E-13 | Cnn3 |
| 10403455 | 1.536805 | 7.038174 | -17.3807 | 1.42E-14 | 9.85E-13 | Dip2c |
| 10373768 | 2.222713 | 9.183192 | -17.3756 | 1.43E-14 | 9.88E-13 | Selm |
| 10406334 | -2.23272 | 6.500408 | 17.3615 | 1.46E-14 | 1.00E-12 | Mctp1 |
| 10361381 | -1.57738 | 7.739152 | 17.35392 | 1.47E-14 | 1.01E-12 | Syne1 |
| 10570855 | 1.756121 | 6.358138 | -17.333 | 1.51E-14 | 1.03E-12 | Plat |
| 10568714 | -2.18613 | 9.549395 | 17.33168 | 1.51E-14 | 1.03E-12 | Mki67 |
| 10474129 | 2.947163 | 6.193383 | -17.3303 | 1.51E-14 | 1.03E-12 | Pamr1 |
| 10534504 | 1.002802 | 6.648182 | -17.3288 | 1.52E-14 | 1.03E-12 | Tmem120a |
| 10368199 | -3.19494 | 7.21988 | 17.31696 | 1.54E-14 | 1.04E-12 | Myb |
| 10584883 | 2.18773 | 8.421437 | -17.3166 | 1.54E-14 | 1.04E-12 | Fxyd6 |
| 10581395 | 1.307993 | 6.632921 | -17.2975 | 1.58E-14 | 1.06E-12 | Slc12a4 |
| 10606366 | 3.29235 | 6.131603 | -17.2894 | 1.59E-14 | 1.07E-12 | Zcchc5 |
| 10375167 | -1.73894 | 5.377547 | 17.28398 | 1.60E-14 | 1.08E-12 | Fam196b |
| 10586505 | -1.31433 | 8.215203 | 17.28134 | 1.61E-14 | 1.08E-12 | Herc1 |
| 10600150 | 1.169663 | 7.485187 | -17.2653 | 1.64E-14 | 1.10E-12 | Zfp275 |
| 10509280 | 3.03416 | 8.300272 | -17.2548 | 1.66E-14 | 1.11E-12 | Hspg2 |
| 10502071 | -1.96849 | 6.746652 | 17.24453 | 1.68E-14 | 1.11E-12 | 5730508B09Rik |
| 10381371 | 1.772643 | 5.813215 | -17.2443 | 1.68E-14 | 1.11E-12 | Aoc3 |
| 10580349 | -2.06569 | 5.485671 | 17.24333 | 1.68E-14 | 1.11E-12 | Mylk3 |
| 10389087 | -1.39585 | 6.793538 | 17.24323 | 1.68E-14 | 1.11E-12 | Rffl |
| 10568202 | -1.95236 | 6.974468 | 17.24059 | 1.69E-14 | 1.11E-12 | 44440 |
| 10546137 | -1.58444 | 6.526497 | 17.23775 | 1.70E-14 | 1.12E-12 | Abtb1 |
| 10557862 | -1.98222 | 8.385389 | 17.2296 | 1.71E-14 | 1.12E-12 | Itgam |
| 10436487 | 2.051995 | 5.923754 | -17.2247 | 1.72E-14 | 1.13E-12 | Vgll3 |
| 10408450 | 1.80159 | 6.617043 | -17.2229 | 1.73E-14 | 1.13E-12 | Sox4 |
| 10587299 | 1.694815 | 6.880021 | -17.2221 | 1.73E-14 | 1.13E-12 | Ick |
| 10531173 | 1.773979 | 5.378631 | -17.2193 | 1.73E-14 | 1.13E-12 | Adamts3 |
| 10366746 | 2.627737 | 6.055872 | -17.2168 | 1.74E-14 | 1.13E-12 | Lrig3 |
| 10597518 | 1.451214 | 7.725546 | -17.2158 | 1.74E-14 | 1.13E-12 | Tgfbr2 |
| 10501860 | 1.605423 | 7.791589 | -17.2139 | 1.75E-14 | 1.13E-12 | Fnbp1l |
| 10435581 | -1.66914 | 5.956298 | 17.21088 | 1.75E-14 | 1.13E-12 | Polq |
| 10482929 | -1.85003 | 6.150019 | 17.20334 | 1.77E-14 | 1.14E-12 | Ly75 |
| 10519578 | -2.32294 | 6.6155 | 17.20032 | 1.78E-14 | 1.14E-12 | Abcb4 |
| 10576911 | 1.918371 | 6.482975 | -17.1914 | 1.79E-14 | 1.15E-12 | Efnb2 |
| 10564818 | 2.492216 | 7.723065 | -17.1856 | 1.81E-14 | 1.15E-12 | Anpep |
| 10505120 | 1.613431 | 5.522498 | -17.1506 | 1.89E-14 | 1.20E-12 | Palm2 |
| 10344897 | 2.871787 | 7.35996 | -17.1495 | 1.89E-14 | 1.20E-12 | Sulf1 |
| 10358816 | 2.013392 | 7.992982 | -17.1479 | 1.89E-14 | 1.20E-12 | Lamc1 |
| 10449225 | 1.297374 | 6.489343 | -17.1431 | 1.90E-14 | 1.21E-12 | Decr2 |
| 10373223 | 1.835483 | 8.521984 | -17.1401 | 1.91E-14 | 1.21E-12 | Lrp1 |
| 10381072 | -2.23359 | 5.903095 | 17.13957 | 1.91E-14 | 1.21E-12 | Cdc6 |
| 10439766 | 1.756168 | 6.526901 | -17.1378 | 1.92E-14 | 1.21E-12 | Pvrl3 |
| 10485828 | -1.25755 | 9.927147 | 17.12421 | 1.95E-14 | 1.22E-12 | Gm10792 |
| 10492798 | 3.652521 | 8.879365 | -17.1038 | 2.00E-14 | 1.25E-12 | Sfrp2 |
| 10351197 | -3.14132 | 7.793415 | 17.09223 | 2.03E-14 | 1.27E-12 | Sell |
| 10569719 | -1.69033 | 6.131985 | 17.08106 | 2.06E-14 | 1.28E-12 | A430078G23Rik |
| 10478447 | -1.5837 | 7.912778 | 17.07651 | 2.07E-14 | 1.29E-12 | Stk4 |
| 10457888 | 1.061483 | 6.511621 | -17.0696 | 2.08E-14 | 1.29E-12 | Tpgs2 |
| 10467768 | 2.886872 | 5.855371 | -17.0519 | 2.13E-14 | 1.32E-12 | Loxl4 |
| 10372807 | 2.102026 | 8.244944 | -17.0518 | 2.13E-14 | 1.32E-12 | Msrb3 |
| 10575120 | 1.113936 | 7.124923 | -17.0508 | 2.13E-14 | 1.32E-12 | Sntb2 |
| 10408629 | 2.176158 | 6.306994 | -17.0471 | 2.14E-14 | 1.32E-12 | Pxdc1 |
| 10542791 | 2.129316 | 7.8711 | -17.0425 | 2.16E-14 | 1.32E-12 | Ppfibp1 |
| 10359624 | 2.108248 | 8.850315 | -17.0343 | 2.18E-14 | 1.33E-12 | Prrx1 |
| 10410039 | 2.944959 | 7.599287 | -17.0254 | 2.20E-14 | 1.34E-12 | Ptch1 |
| 10592535 | -2.25187 | 7.358871 | 17.02465 | 2.20E-14 | 1.34E-12 | Sorl1 |
| 10381934 | 1.413014 | 6.963979 | -17.0165 | 2.23E-14 | 1.35E-12 | Tanc2 |
| 10452571 | 1.738801 | 7.157581 | -17.0158 | 2.23E-14 | 1.35E-12 | Ptprm |
| 10366886 | -1.42822 | 6.199218 | 17.0146 | 2.23E-14 | 1.35E-12 | Arhgap9 |
| 10494351 | 1.470746 | 5.432941 | -16.9995 | 2.27E-14 | 1.37E-12 | Mtmr11 |
| 10576973 | 2.107629 | 7.119281 | -16.9973 | 2.28E-14 | 1.37E-12 | Col4a1 |
| 10423971 | -3.59428 | 5.881804 | 16.99286 | 2.29E-14 | 1.38E-12 | Pkhd1l1 |
| 10398173 | -1.61791 | 7.772167 | 16.98071 | 2.33E-14 | 1.39E-12 | Vrk1 |
| 10460666 | 1.523845 | 7.287678 | -16.9797 | 2.33E-14 | 1.39E-12 | Ltbp3 |
| 10453759 | 2.095864 | 4.716245 | -16.9786 | 2.33E-14 | 1.39E-12 | Gm10554 |
| 10584208 | 1.883052 | 6.51098 | -16.9769 | 2.34E-14 | 1.39E-12 | Cdon |
| 10422028 | 1.184584 | 5.785162 | -16.976 | 2.34E-14 | 1.39E-12 | Tbc1d4 |
| 10483163 | 2.477828 | 7.182727 | -16.9705 | 2.36E-14 | 1.40E-12 | Grb14 |
| 10376455 | -1.80232 | 8.083553 | 16.96675 | 2.37E-14 | 1.40E-12 | Hist3h2a |
| 10523579 | 1.449987 | 6.720105 | -16.9598 | 2.39E-14 | 1.41E-12 | Arhgap24 |
| 10383289 | 1.341064 | 6.227587 | -16.9547 | 2.40E-14 | 1.42E-12 | Baiap2 |
| 10574985 | 1.461371 | 7.029476 | -16.9506 | 2.42E-14 | 1.42E-12 | Slc7a6 |
| 10476538 | 2.001793 | 6.936236 | -16.9454 | 2.43E-14 | 1.43E-12 | Btbd3 |
| 10422348 | 2.401473 | 6.293667 | -16.9422 | 2.44E-14 | 1.43E-12 | Uggt2 |
| 10413657 | 1.495468 | 6.900094 | -16.9288 | 2.48E-14 | 1.45E-12 | Glt8d1 |
| 10471844 | 1.590193 | 6.578968 | -16.9283 | 2.48E-14 | 1.45E-12 | Nek6 |
| 10555323 | 2.596477 | 5.99092 | -16.928 | 2.48E-14 | 1.45E-12 | P4ha3 |
| 10371987 | -1.14107 | 9.695159 | 16.92102 | 2.51E-14 | 1.46E-12 | Metap2 |
| 10461497 | 1.15869 | 9.066042 | -16.9145 | 2.53E-14 | 1.47E-12 | Ddb1 |
| 10484227 | 1.484093 | 6.295008 | -16.9138 | 2.53E-14 | 1.47E-12 | Sestd1 |
| 10607752 | -1.83615 | 4.806652 | 16.90737 | 2.55E-14 | 1.47E-12 | Bmx |
| 10388869 | 1.564037 | 7.172762 | -16.9033 | 2.56E-14 | 1.48E-12 | Tnfaip1 |
| 10579894 | 1.698359 | 4.81934 | -16.9007 | 2.57E-14 | 1.48E-12 | Hhip |
| 10590860 | 2.21693 | 6.329037 | -16.8873 | 2.61E-14 | 1.50E-12 | Arhgap42 |
| 10374406 | 1.443745 | 6.477695 | -16.8785 | 2.64E-14 | 1.52E-12 | Cnrip1 |
| 10493709 | 1.561203 | 8.238013 | -16.8754 | 2.65E-14 | 1.52E-12 | Slc39a1 |
| 10462507 | 2.534625 | 7.737207 | -16.8724 | 2.66E-14 | 1.52E-12 | Papss2 |
| 10419416 | 2.500269 | 5.811326 | -16.862 | 2.70E-14 | 1.54E-12 | 3632451O06Rik |
| 10606735 | 1.633811 | 5.691882 | -16.8555 | 2.72E-14 | 1.55E-12 | Armcx2 |
| 10557177 | -1.98771 | 7.30786 | 16.82771 | 2.81E-14 | 1.59E-12 | Prkcb |
| 10416974 | 1.579952 | 7.124722 | -16.8277 | 2.81E-14 | 1.59E-12 | Gpc6 |
| 10564539 | -2.39779 | 5.407 | 16.82672 | 2.82E-14 | 1.59E-12 | Mctp2 |
| 10425410 | -2.4541 | 6.397865 | 16.82632 | 2.82E-14 | 1.59E-12 | Grap2 |
| 10604564 | 1.585026 | 7.159271 | -16.8262 | 2.82E-14 | 1.59E-12 | Gpc4 |
| 10366293 | 2.914095 | 7.71289 | -16.8244 | 2.83E-14 | 1.59E-12 | Csrp2 |
| 10539080 | -2.02996 | 8.517244 | 16.82203 | 2.83E-14 | 1.59E-12 | St3gal5 |
| 10601412 | 3.089116 | 6.544182 | -16.8131 | 2.87E-14 | 1.60E-12 | Lpar4 |
| 10456492 | 1.08463 | 6.887679 | -16.8088 | 2.88E-14 | 1.61E-12 | Ldlrad4 |
| 10422013 | 1.695914 | 6.056917 | -16.8017 | 2.91E-14 | 1.62E-12 | Klf12 |
| 10541599 | -1.82808 | 5.256564 | 16.79585 | 2.93E-14 | 1.63E-12 | Clec4b2 |
| 10369615 | -1.72102 | 10.09922 | 16.78452 | 2.97E-14 | 1.65E-12 | Srgn |
| 10540275 | 2.78948 | 6.707915 | -16.7821 | 2.98E-14 | 1.65E-12 | Gxylt2 |
| 10505143 | 1.924945 | 5.745877 | -16.7753 | 3.00E-14 | 1.66E-12 | Akap2 |
| 10429128 | -2.53844 | 7.522583 | 16.77278 | 3.01E-14 | 1.66E-12 | Sla |
| 10563077 | 2.820036 | 8.544161 | -16.7607 | 3.06E-14 | 1.69E-12 | Rcn3 |
| 10558150 | 2.529415 | 7.174132 | -16.757 | 3.07E-14 | 1.69E-12 | Htra1 |
| 10497817 | 1.243071 | 10.07581 | -16.7569 | 3.07E-14 | 1.69E-12 | Anxa5 |
| 10457872 | 1.05972 | 6.1182 | -16.756 | 3.08E-14 | 1.69E-12 | Slc39a6 |
| 10406817 | 1.468299 | 6.74287 | -16.7532 | 3.09E-14 | 1.69E-12 | Enc1 |
| 10431711 | 1.718264 | 6.340489 | -16.7447 | 3.12E-14 | 1.70E-12 | Slc2a13 |
| 10416689 | -3.30804 | 6.95607 | 16.74135 | 3.14E-14 | 1.71E-12 | Olfm4 |
| 10364251 | 2.059988 | 7.327767 | -16.735 | 3.16E-14 | 1.72E-12 | Pofut2 |
| 10466886 | 2.160581 | 6.715352 | -16.7326 | 3.17E-14 | 1.72E-12 | Glis3 |
| 10495186 | -2.94121 | 6.398419 | 16.72492 | 3.20E-14 | 1.73E-12 | AI504432 |
| 10537509 | -2.12875 | 5.776165 | 16.72368 | 3.21E-14 | 1.73E-12 | Mgam |
| 10412298 | 1.591652 | 7.328918 | -16.7188 | 3.22E-14 | 1.74E-12 | Itga1 |
| 10360764 | 2.337895 | 7.946235 | -16.7144 | 3.24E-14 | 1.75E-12 | Enah |
| 10350024 | -1.55659 | 5.530541 | 16.7116 | 3.25E-14 | 1.75E-12 | Klhl12 |
| 10502776 | 1.818741 | 7.162929 | -16.7083 | 3.27E-14 | 1.75E-12 | Lphn2 |
| 10496872 | 1.444029 | 7.88433 | -16.6965 | 3.32E-14 | 1.78E-12 | Eltd1 |
| 10544150 | -1.22387 | 8.557652 | 16.68826 | 3.35E-14 | 1.79E-12 | Kdm7a |
| 10361215 | -2.0927 | 5.728783 | 16.67739 | 3.40E-14 | 1.81E-12 | Traf3ip3 |
| 10356886 | -2.77646 | 5.283075 | 16.66163 | 3.47E-14 | 1.85E-12 | Slco4c1 |
| 10441902 | 2.23618 | 8.037649 | -16.6555 | 3.49E-14 | 1.86E-12 | Smoc2 |
| 10372891 | 1.529749 | 6.251326 | -16.6518 | 3.51E-14 | 1.86E-12 | Srgap1 |
| 10399908 | -2.47801 | 7.82154 | 16.65018 | 3.52E-14 | 1.86E-12 | Prkar2b |
| 10600852 | -2.82822 | 8.880949 | 16.64754 | 3.53E-14 | 1.86E-12 | F630028O10Rik |
| 10350848 | 1.066186 | 7.367377 | -16.645 | 3.54E-14 | 1.87E-12 | 2810025M15Rik |
| 10436561 | -1.12613 | 8.810648 | 16.64078 | 3.56E-14 | 1.87E-12 | Usp25 |
| 10538802 | 2.191098 | 5.654201 | -16.6337 | 3.59E-14 | 1.89E-12 | Ndnf |
| 10445753 | -2.9751 | 6.509374 | 16.63048 | 3.60E-14 | 1.89E-12 | Trem3 |
| 10521440 | 1.024052 | 6.371924 | -16.6295 | 3.61E-14 | 1.89E-12 | Afap1 |
| 10389339 | -1.86558 | 8.644246 | 16.62597 | 3.62E-14 | 1.90E-12 | Usp32 |
| 10362499 | 1.517617 | 5.837954 | -16.6176 | 3.66E-14 | 1.91E-12 | Frk |
| 10518350 | -1.57971 | 10.48479 | 16.61195 | 3.69E-14 | 1.92E-12 | Hmgb2 |
| 10455813 | -1.79251 | 8.072072 | 16.6111 | 3.69E-14 | 1.92E-12 | Lmnb1 |
| 10381898 | 2.060833 | 7.196932 | -16.6037 | 3.73E-14 | 1.93E-12 | Mrc2 |
| 10555736 | 1.770447 | 5.327266 | -16.5916 | 3.79E-14 | 1.96E-12 | Olfr558 |
| 10423293 | 1.786142 | 8.055839 | -16.5739 | 3.87E-14 | 2.00E-12 | Myo10 |
| 10587639 | 2.201085 | 7.2321 | -16.5597 | 3.94E-14 | 2.03E-12 | Nt5e |
| 10496796 | -1.06162 | 7.198397 | 16.55941 | 3.94E-14 | 2.03E-12 | Ssx2ip |
| 10488060 | 1.388236 | 5.613915 | -16.5577 | 3.95E-14 | 2.03E-12 | Jag1 |
| 10490903 | 1.731987 | 6.492352 | -16.5521 | 3.98E-14 | 2.04E-12 | Car13 |
| 10373027 | 1.262182 | 7.915346 | -16.5504 | 3.99E-14 | 2.04E-12 | Tspan31 |
| 10438421 | -1.88089 | 4.194521 | 16.54867 | 4.00E-14 | 2.04E-12 | Olfr164 |
| 10349174 | 1.390777 | 5.519755 | -16.5362 | 4.06E-14 | 2.07E-12 | Serpinb8 |
| 10591517 | -1.83205 | 7.157017 | 16.5351 | 4.07E-14 | 2.07E-12 | Cdkn2d |
| 10590821 | 1.555544 | 4.520709 | -16.5337 | 4.07E-14 | 2.07E-12 | 9230110C19Rik |
| 10440491 | 1.56766 | 9.452191 | -16.526 | 4.11E-14 | 2.08E-12 | App |
| 10462922 | 1.777263 | 6.154977 | -16.5254 | 4.12E-14 | 2.08E-12 | Plce1 |
| 10498383 | 2.560986 | 7.720602 | -16.5173 | 4.16E-14 | 2.10E-12 | Igsf10 |
| 10379633 | -2.67162 | 8.010777 | 16.50735 | 4.21E-14 | 2.13E-12 | Slfn1 |
| 10464084 | 1.487937 | 8.15661 | -16.5037 | 4.23E-14 | 2.13E-12 | Tcf7l2 |
| 10504375 | 1.917525 | 6.719832 | -16.4963 | 4.27E-14 | 2.15E-12 | Npr2 |
| 10455948 | 1.778994 | 6.077187 | -16.4777 | 4.37E-14 | 2.19E-12 | Chsy3 |
| 10571870 | -1.55348 | 10.561 | 16.4765 | 4.38E-14 | 2.19E-12 | Hmgb2 |
| 10414102 | 1.466966 | 6.5954 | -16.4752 | 4.39E-14 | 2.19E-12 | Mmrn2 |
| 10538811 | 2.045386 | 6.431665 | -16.4636 | 4.45E-14 | 2.22E-12 | Prdm5 |
| 10371332 | 2.415036 | 6.160432 | -16.4562 | 4.49E-14 | 2.24E-12 | Aldh1l2 |
| 10567580 | -2.056 | 8.092149 | 16.45415 | 4.51E-14 | 2.24E-12 | Igsf6 |
| 10598240 | 1.115175 | 5.658766 | -16.4294 | 4.65E-14 | 2.31E-12 | Shroom4 |
| 10358224 | -1.83017 | 8.837717 | 16.42477 | 4.68E-14 | 2.31E-12 | Ptprc |
| 10545780 | 1.369427 | 7.043281 | -16.4248 | 4.68E-14 | 2.31E-12 | Exoc6b |
| 10572906 | -1.57252 | 7.28218 | 16.42466 | 4.68E-14 | 2.31E-12 | Mcm5 |
| 10575844 | 1.886606 | 6.885461 | -16.4215 | 4.70E-14 | 2.31E-12 | Cdh13 |
| 10350335 | -1.68518 | 7.949 | 16.41951 | 4.71E-14 | 2.32E-12 | Hmbs |
| 10570291 | -1.97523 | 6.375436 | 16.41606 | 4.73E-14 | 2.32E-12 | F10 |
| 10544186 | -2.81209 | 8.288204 | 16.41471 | 4.74E-14 | 2.32E-12 | Mkrn1 |
| 10447951 | 3.671535 | 8.69991 | -16.4116 | 4.76E-14 | 2.33E-12 | Thbs2 |
| 10354598 | 1.262656 | 5.71592 | -16.4026 | 4.81E-14 | 2.35E-12 | Hecw2 |
| 10443470 | -2.03715 | 6.793758 | 16.40093 | 4.82E-14 | 2.35E-12 | Rab44 |
| 10381096 | 1.878955 | 9.854286 | -16.3965 | 4.85E-14 | 2.36E-12 | Igfbp4 |
| 10607658 | 1.469633 | 5.927962 | -16.3798 | 4.95E-14 | 2.41E-12 | Reps2 |
| 10601648 | 3.637971 | 8.455862 | -16.3549 | 5.11E-14 | 2.48E-12 | Tnmd |
| 10468311 | 1.501153 | 5.98155 | -16.3494 | 5.15E-14 | 2.49E-12 | Sh3pxd2a |
| 10584561 | 1.984489 | 7.663882 | -16.3484 | 5.16E-14 | 2.49E-12 | Clmp |
| 10577757 | 1.228517 | 7.390761 | -16.3376 | 5.23E-14 | 2.52E-12 | Adam9 |
| 10478692 | 1.781278 | 6.144617 | -16.3214 | 5.34E-14 | 2.57E-12 | Slc2a10 |
| 10575993 | -1.18625 | 7.354435 | 16.3129 | 5.40E-14 | 2.60E-12 | 6430548M08Rik |
| 10467637 | -2.19441 | 7.03888 | 16.28859 | 5.57E-14 | 2.67E-12 | Arhgap19 |
| 10415408 | 1.238993 | 5.868359 | -16.2873 | 5.58E-14 | 2.67E-12 | Nynrin |
| 10554081 | 1.033616 | 5.087996 | -16.2841 | 5.60E-14 | 2.68E-12 | Ttc23 |
| 10406877 | 1.964577 | 6.840254 | -16.2822 | 5.61E-14 | 2.68E-12 | Serf1 |
| 10400844 | -1.99563 | 8.37167 | 16.27607 | 5.66E-14 | 2.69E-12 | Pygl |
| 10606948 | 1.968125 | 5.778651 | -16.2691 | 5.71E-14 | 2.71E-12 | Morc4 |
| 10571142 | 1.735136 | 6.1729 | -16.2673 | 5.72E-14 | 2.71E-12 | Gpr124 |
| 10440393 | -1.85106 | 6.989767 | 16.26644 | 5.73E-14 | 2.71E-12 | Samsn1 |
| 10460544 | 1.564635 | 7.742934 | -16.2553 | 5.81E-14 | 2.75E-12 | Yif1a |
| 10546454 | 2.260066 | 6.465447 | -16.2516 | 5.84E-14 | 2.76E-12 | Adamts9 |
| 10420988 | 1.444135 | 7.707064 | -16.2456 | 5.88E-14 | 2.77E-12 | Dpysl2 |
| 10494583 | 1.019581 | 8.7195 | -16.2235 | 6.05E-14 | 2.85E-12 | Sec22b |
| 10522503 | 1.906759 | 7.683779 | -16.2144 | 6.12E-14 | 2.88E-12 | Pdgfra |
| 10475378 | 1.944096 | 6.896615 | -16.212 | 6.14E-14 | 2.88E-12 | Casc4 |
| 10567219 | -1.31035 | 9.273206 | 16.20623 | 6.19E-14 | 2.90E-12 | Arl6ip1 |
| 10534667 | 2.922944 | 6.833487 | -16.2037 | 6.21E-14 | 2.90E-12 | Serpine1 |
| 10350173 | 2.439178 | 6.96088 | -16.2019 | 6.22E-14 | 2.91E-12 | Tnnt2 |
| 10481262 | -2.80162 | 7.387732 | 16.18864 | 6.33E-14 | 2.95E-12 | Fcnb |
| 10354286 | 1.587402 | 6.601629 | -16.1884 | 6.33E-14 | 2.95E-12 | Kdelc1 |
| 10579744 | 1.395085 | 6.053524 | -16.1866 | 6.35E-14 | 2.95E-12 | Large |
| 10572378 | 3.796865 | 8.167558 | -16.1811 | 6.39E-14 | 2.97E-12 | Comp |
| 10354389 | 1.435575 | 6.581502 | -16.18 | 6.40E-14 | 2.97E-12 | Slc39a10 |
| 10362201 | 2.247256 | 8.931334 | -16.1739 | 6.45E-14 | 2.98E-12 | Ctgf |
| 10578572 | 2.570461 | 6.864905 | -16.1672 | 6.51E-14 | 3.00E-12 | Stox2 |
| 10420035 | 1.362653 | 7.067922 | -16.1637 | 6.54E-14 | 3.01E-12 | Ipo4 |
| 10347564 | -1.07604 | 6.465757 | 16.15571 | 6.61E-14 | 3.03E-12 | Dnajb2 |
| 10543939 | 2.676737 | 6.495654 | -16.1493 | 6.66E-14 | 3.05E-12 | Fam180a |
| 10429341 | 1.642025 | 6.738635 | -16.1405 | 6.74E-14 | 3.08E-12 | Ptk2 |
| 10544501 | -1.50045 | 8.029712 | 16.12727 | 6.85E-14 | 3.12E-12 | Ezh2 |
| 10361098 | 1.669925 | 9.532578 | -16.1183 | 6.93E-14 | 3.16E-12 | Nenf |
| 10547641 | -2.49259 | 7.298719 | 16.11165 | 6.99E-14 | 3.18E-12 | Slc2a3 |
| 10383532 | -1.46317 | 7.016835 | 16.10582 | 7.05E-14 | 3.20E-12 | Narf |
| 10594645 | -1.22451 | 9.044819 | 16.10173 | 7.08E-14 | 3.21E-12 | Rab8b |
| 10544885 | 3.015384 | 7.150735 | -16.1 | 7.10E-14 | 3.21E-12 | Fkbp14 |
| 10445767 | -2.86503 | 6.679494 | 16.09438 | 7.15E-14 | 3.23E-12 | Treml2 |
| 10346576 | -1.59774 | 6.837007 | 16.09061 | 7.19E-14 | 3.24E-12 | Stradb |
| 10361338 | -2.51469 | 5.689636 | 16.08349 | 7.25E-14 | 3.27E-12 | Ipcef1 |
| 10455139 | 1.538072 | 5.282283 | -16.0718 | 7.36E-14 | 3.31E-12 | Pcdhb22 |
| 10445867 | -1.16858 | 7.075669 | 16.06915 | 7.39E-14 | 3.32E-12 | Plcl2 |
| 10514177 | 2.426898 | 6.19999 | -16.0648 | 7.43E-14 | 3.33E-12 | Bnc2 |
| 10500808 | 2.18842 | 8.789794 | -16.0635 | 7.44E-14 | 3.33E-12 | Olfml3 |
| 10471555 | 2.786658 | 8.118545 | -16.0608 | 7.47E-14 | 3.34E-12 | Angptl2 |
| 10408113 | -1.75367 | 8.05764 | 16.05716 | 7.51E-14 | 3.35E-12 | Hist1h4i |
| 10475487 | -1.51505 | 5.928961 | 16.05048 | 7.57E-14 | 3.37E-12 | Slc28a2 |
| 10421456 | -2.44265 | 9.261827 | 16.04774 | 7.60E-14 | 3.38E-12 | Xpo7 |
| 10353549 | 2.250684 | 6.445913 | -16.0406 | 7.67E-14 | 3.41E-12 | Fam135a |
| 10446656 | -1.59299 | 7.586852 | 16.03613 | 7.71E-14 | 3.42E-12 | Lpin2 |
| 10578649 | 2.152115 | 6.462873 | -16.0311 | 7.76E-14 | 3.44E-12 | Tenm3 |
| 10549647 | -1.65715 | 4.924832 | 16.02682 | 7.81E-14 | 3.45E-12 | Ncr1 |
| 10402787 | 1.604035 | 7.575339 | -16.0137 | 7.94E-14 | 3.51E-12 | Ahnak2 |
| 10578477 | 1.434168 | 5.99487 | -16.0065 | 8.02E-14 | 3.53E-12 | Fam149a |
| 10406905 | -2.1313 | 6.26089 | 16.00543 | 8.03E-14 | 3.53E-12 | Ccdc125 |
| 10360227 | 1.311257 | 8.514627 | -16.0024 | 8.06E-14 | 3.54E-12 | Pea15a |
| 10424746 | 1.058449 | 6.096629 | -15.9911 | 8.18E-14 | 3.59E-12 | Zfp623 |
| 10542665 | -1.43453 | 8.808649 | 15.98695 | 8.22E-14 | 3.60E-12 | Cmas |
| 10406982 | 1.829283 | 5.45289 | -15.9845 | 8.25E-14 | 3.60E-12 | Adamts6 |
| 10374333 | -2.61394 | 7.293677 | 15.98402 | 8.26E-14 | 3.60E-12 | Ikzf1 |
| 10433114 | 1.803737 | 7.074619 | -15.9808 | 8.29E-14 | 3.61E-12 | Itga5 |
| 10422244 | 2.584151 | 4.806747 | -15.9802 | 8.30E-14 | 3.61E-12 | Slitrk6 |
| 10593198 | -3.34905 | 7.689093 | 15.97179 | 8.39E-14 | 3.65E-12 | Nxpe2 |
| 10503107 | -1.86614 | 7.250592 | 15.96663 | 8.45E-14 | 3.66E-12 | 6330407A03Rik |
| 10400357 | -1.45922 | 7.81297 | 15.96524 | 8.46E-14 | 3.66E-12 | Baz1a |
| 10419892 | 1.632053 | 6.214104 | -15.9649 | 8.46E-14 | 3.66E-12 | Efs |
| 10349383 | 1.671326 | 7.407297 | -15.9558 | 8.57E-14 | 3.70E-12 | Slc35f5 |
| 10351551 | 1.976513 | 5.746981 | -15.9495 | 8.64E-14 | 3.73E-12 | Adamts4 |
| 10489759 | 2.312009 | 7.413255 | -15.9464 | 8.67E-14 | 3.74E-12 | Sulf2 |
| 10482802 | -2.29464 | 6.770518 | 15.93682 | 8.78E-14 | 3.78E-12 | Cytip |
| 10422321 | 1.404759 | 5.937471 | -15.9337 | 8.82E-14 | 3.79E-12 | Dzip1 |
| 10350516 | 3.602875 | 7.31452 | -15.9253 | 8.91E-14 | 3.82E-12 | Ptgs2 |
| 10467766 | 3.101966 | 6.330252 | -15.9232 | 8.94E-14 | 3.82E-12 | Loxl4 |
| 10346000 | 2.695974 | 6.567454 | -15.9195 | 8.98E-14 | 3.83E-12 | Gulp1 |
| 10445574 | 1.615947 | 5.450137 | -15.9194 | 8.98E-14 | 3.83E-12 | Cul7 |
| 10494467 | 2.198553 | 6.97897 | -15.9174 | 9.01E-14 | 3.83E-12 | Itga10 |
| 10507040 | 1.402836 | 6.346279 | -15.9165 | 9.02E-14 | 3.83E-12 | Spata6 |
| 10347291 | -3.17421 | 8.509983 | 15.90758 | 9.12E-14 | 3.87E-12 | Cxcr2 |
| 10426611 | 1.530716 | 6.03762 | -15.904 | 9.17E-14 | 3.88E-12 | Cacnb3 |
| 10596747 | 1.162929 | 6.365902 | -15.8999 | 9.21E-14 | 3.90E-12 | Sema3f |
| 10552276 | -1.28647 | 7.90423 | 15.89936 | 9.22E-14 | 3.90E-12 | Ube2h |
| 10519497 | 1.664102 | 7.96163 | -15.8924 | 9.31E-14 | 3.93E-12 | Steap4 |
| 10477777 | 1.229936 | 9.277969 | -15.8899 | 9.34E-14 | 3.93E-12 | Ergic3 |
| 10587383 | 2.789858 | 7.213943 | -15.86 | 9.71E-14 | 4.08E-12 | Cd109 |
| 10603109 | -1.34689 | 5.511835 | 15.84166 | 9.95E-14 | 4.18E-12 | Piga |
| 10600093 | 2.192455 | 5.039492 | -15.8404 | 9.96E-14 | 4.18E-12 | Zfp185 |
| 10346882 | 1.576497 | 5.351089 | -15.8326 | 1.01E-13 | 4.22E-12 | Adam23 |
| 10427095 | 1.730156 | 7.480773 | -15.8291 | 1.01E-13 | 4.23E-12 | Tenc1 |
| 10505073 | 1.505654 | 6.851174 | -15.8074 | 1.04E-13 | 4.34E-12 | Zfp462 |
| 10362314 | 1.913748 | 6.761156 | -15.8069 | 1.04E-13 | 4.34E-12 | Ptprk |
| 10529515 | 1.671114 | 6.619346 | -15.8025 | 1.05E-13 | 4.36E-12 | Sorcs2 |
| 10400510 | 1.617029 | 5.869587 | -15.7969 | 1.06E-13 | 4.39E-12 | Clec14a |
| 10571657 | -1.74711 | 8.305032 | 15.79545 | 1.06E-13 | 4.39E-12 | Acsl1 |
| 10482500 | 1.556265 | 8.232482 | -15.7934 | 1.06E-13 | 4.39E-12 | Rnd3 |
| 10510172 | -1.56259 | 10.3327 | 15.77682 | 1.08E-13 | 4.48E-12 | Hmgb2 |
| 10497831 | -1.70456 | 9.380106 | 15.76117 | 1.11E-13 | 4.57E-12 | Ccna2 |
| 10347948 | -2.01558 | 8.094533 | 15.75368 | 1.12E-13 | 4.61E-12 | Sp100 |
| 10490894 | 1.202352 | 5.393634 | -15.7527 | 1.12E-13 | 4.61E-12 | E2f5 |
| 10568638 | -1.80853 | 6.473349 | 15.75082 | 1.12E-13 | 4.61E-12 | Uros |
| 10361375 | -2.12384 | 8.147842 | 15.73916 | 1.14E-13 | 4.67E-12 | Fbxo5 |
| 10451646 | -2.2699 | 6.282725 | 15.73899 | 1.14E-13 | 4.67E-12 | A530064D06Rik |
| 10398240 | 1.875099 | 7.215446 | -15.7362 | 1.14E-13 | 4.68E-12 | Eml1 |
| 10557571 | -1.57143 | 6.167085 | 15.71804 | 1.17E-13 | 4.78E-12 | AI467606 |
| 10560886 | -2.84583 | 8.021621 | 15.71411 | 1.18E-13 | 4.79E-12 | Cd177 |
| 10409031 | -1.42668 | 9.364101 | 15.71351 | 1.18E-13 | 4.79E-12 | Dek |
| 10455108 | 1.833008 | 5.206719 | -15.7065 | 1.19E-13 | 4.83E-12 | Pcdhb16 |
| 10562761 | 2.385395 | 7.542288 | -15.702 | 1.20E-13 | 4.85E-12 | Clec11a |
| 10352194 | 1.451635 | 7.54888 | -15.6986 | 1.20E-13 | 4.87E-12 | Cdc42bpa |
| 10488195 | 1.554927 | 9.106296 | -15.6963 | 1.20E-13 | 4.88E-12 | Rrbp1 |
| 10404152 | -2.7328 | 7.115591 | 15.69325 | 1.21E-13 | 4.88E-12 | Fam65b |
| 10347931 | -2.09519 | 10.3527 | 15.693 | 1.21E-13 | 4.88E-12 | G530012D18Rik |
| 10580056 | 1.663216 | 7.351203 | -15.6814 | 1.23E-13 | 4.95E-12 | Gm10644 |
| 10601778 | 1.676465 | 7.072489 | -15.6782 | 1.23E-13 | 4.97E-12 | Armcx3 |
| 10595466 | 1.766447 | 6.536995 | -15.6753 | 1.24E-13 | 4.98E-12 | Pgm3 |
| 10491835 | -1.41583 | 6.650197 | 15.66907 | 1.25E-13 | 5.01E-12 | Larp1b |
| 10474860 | 1.615718 | 7.277492 | -15.6642 | 1.26E-13 | 5.04E-12 | Chst14 |
| 10497001 | 1.269628 | 6.261365 | -15.6636 | 1.26E-13 | 5.04E-12 | Cryz |
| 10414514 | -1.74996 | 8.833031 | 15.65338 | 1.28E-13 | 5.10E-12 | Pnp |
| 10484307 | 3.739246 | 5.997173 | -15.6456 | 1.29E-13 | 5.14E-12 | Frzb |
| 10569569 | 1.36586 | 7.396965 | -15.6451 | 1.29E-13 | 5.14E-12 | Cttn |
| 10436392 | -2.80432 | 8.050556 | 15.62288 | 1.33E-13 | 5.29E-12 | Cpox |
| 10348739 | 1.330348 | 6.658469 | -15.6186 | 1.34E-13 | 5.31E-12 | Sned1 |
| 10441497 | 1.846947 | 7.590283 | -15.6008 | 1.37E-13 | 5.43E-12 | Tulp4 |
| 10480891 | -1.88522 | 7.847147 | 15.59976 | 1.37E-13 | 5.43E-12 | Ubac1 |
| 10520965 | 2.262591 | 6.479556 | -15.5962 | 1.38E-13 | 5.45E-12 | Yes1 |
| 10516823 | -3.07454 | 8.670942 | 15.59511 | 1.38E-13 | 5.45E-12 | Epb4.1 |
| 10455112 | 1.815038 | 5.423886 | -15.5785 | 1.41E-13 | 5.56E-12 | Pcdhb17 |
| 10469786 | -3.06629 | 6.598074 | 15.5565 | 1.45E-13 | 5.72E-12 | Il1f9 |
| 10564417 | 1.717894 | 5.885032 | -15.5505 | 1.46E-13 | 5.76E-12 | Aldh1a3 |
| 10370037 | 1.222732 | 5.938608 | -15.5497 | 1.46E-13 | 5.76E-12 | Mmp11 |
| 10440522 | 1.947091 | 6.311792 | -15.5447 | 1.47E-13 | 5.79E-12 | Adamts1 |
| 10396421 | 2.255314 | 8.784857 | -15.541 | 1.48E-13 | 5.81E-12 | Hif1a |
| 10526566 | 1.554716 | 6.039336 | -15.5347 | 1.49E-13 | 5.85E-12 | Ephb4 |
| 10370587 | 1.241764 | 5.214746 | -15.5336 | 1.50E-13 | 5.85E-12 | Shc2 |
| 10494322 | -1.54572 | 8.248765 | 15.52558 | 1.51E-13 | 5.90E-12 | Anp32e |
| 10534940 | -2.32827 | 6.50178 | 15.5157 | 1.53E-13 | 5.97E-12 | Pilrb2 |
| 10376074 | 2.214492 | 6.44188 | -15.5082 | 1.55E-13 | 6.03E-12 | P4ha2 |
| 10591253 | 1.579562 | 7.553268 | -15.5057 | 1.55E-13 | 6.03E-12 | Zfp266 |
| 10351905 | -4.37156 | 6.91332 | 15.50545 | 1.55E-13 | 6.03E-12 | Spta1 |
| 10518352 | -1.50312 | 10.22679 | 15.50503 | 1.55E-13 | 6.03E-12 | Gm13160 |
| 10392449 | 1.36485 | 8.295553 | -15.5043 | 1.56E-13 | 6.03E-12 | Wipi1 |
| 10485982 | 3.156001 | 7.805974 | -15.5006 | 1.56E-13 | 6.05E-12 | Actc1 |
| 10434782 | 1.746553 | 7.245993 | -15.4987 | 1.57E-13 | 6.05E-12 | Lpp |
| 10366476 | 1.49543 | 7.04368 | -15.493 | 1.58E-13 | 6.08E-12 | Ptprb |
| 10441178 | -2.53587 | 7.181821 | 15.49243 | 1.58E-13 | 6.08E-12 | Itgb2l |
| 10563820 | -1.49601 | 7.841329 | 15.47109 | 1.63E-13 | 6.23E-12 | Svip |
| 10425161 | 1.411394 | 11.21113 | -15.457 | 1.66E-13 | 6.34E-12 | Lgals1 |
| 10349166 | -3.30814 | 5.321277 | 15.45332 | 1.67E-13 | 6.37E-12 | Serpinb10 |
| 10362102 | -1.7177 | 7.040383 | 15.45047 | 1.67E-13 | 6.38E-12 | Gm10825 |
| 10458663 | 2.03261 | 7.369755 | -15.4471 | 1.68E-13 | 6.40E-12 | Dpysl3 |
| 10495539 | 1.084182 | 6.152394 | -15.4456 | 1.68E-13 | 6.41E-12 | Extl2 |
| 10401673 | 2.746275 | 7.580323 | -15.4432 | 1.69E-13 | 6.42E-12 | Tgfb3 |
| 10600597 | 1.983469 | 7.142384 | -15.4411 | 1.69E-13 | 6.42E-12 | Tmem47 |
| 10398085 | -1.74235 | 8.678185 | 15.44056 | 1.69E-13 | 6.42E-12 | Glrx5 |
| 10358421 | -2.94854 | 7.485352 | 15.43807 | 1.70E-13 | 6.43E-12 | Rgs18 |
| 10466712 | 2.42684 | 6.145968 | -15.4336 | 1.71E-13 | 6.46E-12 | Mamdc2 |
| 10420891 | 1.955897 | 7.352907 | -15.4187 | 1.74E-13 | 6.58E-12 | Scara3 |
| 10427471 | 1.873061 | 6.98642 | -15.413 | 1.76E-13 | 6.62E-12 | Osmr |
| 10346015 | 3.428001 | 9.49213 | -15.4125 | 1.76E-13 | 6.62E-12 | Col3a1 |
| 10429580 | -3.30162 | 8.421743 | 15.41035 | 1.76E-13 | 6.63E-12 | I830127L07Rik |
| 10355329 | -1.73884 | 5.42702 | 15.40957 | 1.77E-13 | 6.63E-12 | Bard1 |
| 10456988 | 1.611776 | 5.299351 | -15.3932 | 1.81E-13 | 6.77E-12 | Pard6g |
| 10548817 | -2.09314 | 9.034588 | 15.39124 | 1.81E-13 | 6.77E-12 | Plbd1 |
| 10384370 | -1.40655 | 6.581498 | 15.39073 | 1.81E-13 | 6.77E-12 | Gm12000 |
| 10569877 | -2.83782 | 8.492411 | 15.37716 | 1.85E-13 | 6.89E-12 | Mcemp1 |
| 10378754 | 1.629735 | 6.377651 | -15.3652 | 1.88E-13 | 6.98E-12 | Fam57a |
| 10597575 | 1.905727 | 6.300964 | -15.3605 | 1.89E-13 | 7.02E-12 | Plcd1 |
| 10514902 | 1.247555 | 6.015006 | -15.3517 | 1.91E-13 | 7.08E-12 | Lrrc42 |
| 10449419 | 1.305277 | 5.913516 | -15.3498 | 1.91E-13 | 7.08E-12 | Tead3 |
| 10352767 | -1.48719 | 6.194366 | 15.34948 | 1.92E-13 | 7.08E-12 | Nek2 |
| 10578989 | 1.370617 | 7.178357 | -15.3432 | 1.93E-13 | 7.13E-12 | Psd3 |
| 10456046 | 1.890132 | 7.332745 | -15.3428 | 1.93E-13 | 7.13E-12 | Pdgfrb |
| 10458028 | -1.51467 | 7.49244 | 15.3366 | 1.95E-13 | 7.18E-12 | Gypc |
| 10528008 | 1.861884 | 5.274997 | -15.3281 | 1.97E-13 | 7.25E-12 | Steap2 |
| 10412260 | 1.478631 | 6.849279 | -15.3272 | 1.97E-13 | 7.25E-12 | Fst |
| 10573008 | 1.492853 | 6.656595 | -15.3262 | 1.98E-13 | 7.26E-12 | Zfp827 |
| 10435009 | -1.10117 | 4.313596 | 15.32163 | 1.99E-13 | 7.29E-12 | Bex6 |
| 10358581 | 2.185119 | 5.183547 | -15.3203 | 1.99E-13 | 7.30E-12 | Hmcn1 |
| 10532839 | 2.531543 | 6.401301 | -15.3171 | 2.00E-13 | 7.32E-12 | Trpv4 |
| 10570957 | 3.665676 | 6.981713 | -15.3124 | 2.01E-13 | 7.35E-12 | Sfrp1 |
| 10436598 | 2.410094 | 4.828179 | -15.3052 | 2.03E-13 | 7.42E-12 | 2810055G20Rik |
| 10478219 | 1.223844 | 7.041847 | -15.304 | 2.04E-13 | 7.42E-12 | Plcg1 |
| 10569504 | 1.845329 | 6.267571 | -15.3004 | 2.05E-13 | 7.44E-12 | Tnfrsf23 |
| 10429754 | 1.589574 | 6.044521 | -15.3001 | 2.05E-13 | 7.44E-12 | Nrbp2 |
| 10539517 | 1.231775 | 6.248804 | -15.2991 | 2.05E-13 | 7.44E-12 | Dysf |
| 10560709 | 1.726305 | 6.379705 | -15.2967 | 2.06E-13 | 7.46E-12 | Pvr |
| 10578241 | 1.528208 | 6.862193 | -15.2936 | 2.07E-13 | 7.46E-12 | Dlc1 |
| 10367919 | -1.86936 | 6.04257 | 15.2927 | 2.07E-13 | 7.46E-12 | Stx11 |
| 10594774 | -1.49906 | 9.137639 | 15.29266 | 2.07E-13 | 7.46E-12 | Ccnb2 |
| 10431637 | 1.997372 | 6.036543 | -15.2924 | 2.07E-13 | 7.46E-12 | Cpne8 |
| 10361055 | 1.332951 | 5.215642 | -15.2871 | 2.08E-13 | 7.51E-12 | Vash2 |
| 10453747 | 2.141027 | 7.921684 | -15.2842 | 2.09E-13 | 7.53E-12 | Colec12 |
| 10474201 | -2.00251 | 8.49878 | 15.28301 | 2.10E-13 | 7.53E-12 | Lmo2 |
| 10509441 | 1.576982 | 7.167997 | -15.2749 | 2.12E-13 | 7.60E-12 | Ece1 |
| 10437594 | -1.15979 | 9.087209 | 15.2673 | 2.14E-13 | 7.67E-12 | Usp7 |
| 10352905 | 1.740566 | 8.855666 | -15.2634 | 2.15E-13 | 7.70E-12 | Cd34 |
| 10354897 | -1.87411 | 8.53945 | 15.2579 | 2.17E-13 | 7.75E-12 | Trak2 |
| 10461452 | -1.52422 | 6.302507 | 15.25643 | 2.17E-13 | 7.75E-12 | Gm10143 |
| 10384652 | -1.50119 | 5.378597 | 15.25547 | 2.18E-13 | 7.75E-12 | Gm12057 |
| 10569707 | 1.388533 | 9.127037 | -15.2544 | 2.18E-13 | 7.75E-12 | Myadm |
| 10431424 | 1.783619 | 7.43261 | -15.2498 | 2.19E-13 | 7.79E-12 | Plxnb2 |
| 10410695 | 1.570412 | 7.376747 | -15.2431 | 2.21E-13 | 7.85E-12 | Rhobtb3 |
| 10490502 | 1.108235 | 4.884758 | -15.2422 | 2.21E-13 | 7.85E-12 | Tcfl5 |
| 10368495 | 1.463931 | 6.21671 | -15.239 | 2.22E-13 | 7.87E-12 | Rspo3 |
| 10595981 | 1.887585 | 7.013845 | -15.2304 | 2.25E-13 | 7.96E-12 | Mras |
| 10596637 | -1.40458 | 7.251853 | 15.22854 | 2.26E-13 | 7.97E-12 | Mapkapk3 |
| 10345101 | 3.384748 | 6.802138 | -15.2237 | 2.27E-13 | 8.01E-12 | Col9a1 |
| 10372342 | 1.645387 | 6.12375 | -15.2204 | 2.28E-13 | 8.04E-12 | Nav3 |
| 10385719 | 1.203462 | 7.987368 | -15.2161 | 2.29E-13 | 8.07E-12 | Sec24a |
| 10420254 | -3.05332 | 6.665913 | 15.21153 | 2.31E-13 | 8.11E-12 | Mcpt8 |
| 10437817 | 1.108831 | 7.780008 | -15.2038 | 2.33E-13 | 8.19E-12 | Pdxdc1 |
| 10418053 | 2.460123 | 7.497805 | -15.2024 | 2.34E-13 | 8.19E-12 | Kcnma1 |
| 10572605 | -1.43889 | 5.637295 | 15.19993 | 2.35E-13 | 8.21E-12 | Ankle1 |
| 10562117 | -1.55498 | 5.78326 | 15.19628 | 2.36E-13 | 8.24E-12 | Ffar2 |
| 10591369 | -1.28358 | 8.218754 | 15.19576 | 2.36E-13 | 8.24E-12 | Dnmt1 |
| 10498386 | 2.634612 | 7.731005 | -15.1944 | 2.36E-13 | 8.24E-12 | Igsf10 |
| 10554863 | 2.024092 | 6.269826 | -15.1924 | 2.37E-13 | 8.26E-12 | Sytl2 |
| 10412466 | 1.311312 | 7.47483 | -15.1836 | 2.40E-13 | 8.35E-12 | Hmgcs1 |
| 10351903 | -1.15122 | 4.292817 | 15.17822 | 2.42E-13 | 8.40E-12 | Olfr420 |
| 10441864 | 1.054645 | 7.107977 | -15.1737 | 2.43E-13 | 8.44E-12 | Mllt4 |
| 10435714 | 1.242606 | 8.581972 | -15.1719 | 2.44E-13 | 8.45E-12 | Tmem39a |
| 10593032 | -1.16779 | 5.674157 | 15.16164 | 2.47E-13 | 8.56E-12 | Gm10684 |
| 10545958 | 1.538627 | 7.939204 | -15.1592 | 2.48E-13 | 8.58E-12 | Anxa4 |
| 10565996 | 1.525735 | 7.185663 | -15.1549 | 2.49E-13 | 8.62E-12 | Inppl1 |
| 10561008 | -2.43621 | 7.619394 | 15.14973 | 2.51E-13 | 8.67E-12 | Ceacam1 |
| 10395409 | 2.794283 | 6.967261 | -15.142 | 2.54E-13 | 8.75E-12 | Meox2 |
| 10396270 | 1.770292 | 6.013394 | -15.1354 | 2.56E-13 | 8.82E-12 | Dact1 |
| 10490777 | 1.819602 | 7.507259 | -15.1314 | 2.57E-13 | 8.85E-12 | Zfhx4 |
| 10498273 | 1.924128 | 7.058539 | -15.1278 | 2.59E-13 | 8.89E-12 | Tm4sf1 |
| 10410460 | 1.457985 | 5.707096 | -15.1262 | 2.59E-13 | 8.90E-12 | Ube2ql1 |
| 10411519 | 1.5165 | 8.149366 | -15.1115 | 2.65E-13 | 9.05E-12 | Map1b |
| 10407081 | -1.96665 | 5.955684 | 15.11074 | 2.65E-13 | 9.05E-12 | Depdc1b |
| 10515257 | -1.79533 | 6.456014 | 15.10609 | 2.67E-13 | 9.10E-12 | Rad54l |
| 10467115 | -2.40482 | 6.133494 | 15.10393 | 2.67E-13 | 9.12E-12 | Ankrd22 |
| 10582868 | -1.6118 | 8.50814 | 15.10246 | 2.68E-13 | 9.12E-12 | LOC546061 |
| 10427898 | 1.395869 | 5.934186 | -15.0955 | 2.70E-13 | 9.20E-12 | Fbxl7 |
| 10594110 | 2.404494 | 8.455431 | -15.0914 | 2.72E-13 | 9.23E-12 | Neo1 |
| 10363575 | -1.634 | 6.359408 | 15.09106 | 2.72E-13 | 9.23E-12 | Dna2 |
| 10400805 | -1.16095 | 7.513077 | 15.09027 | 2.72E-13 | 9.23E-12 | Nin |
| 10472050 | 3.273548 | 6.06108 | -15.0763 | 2.78E-13 | 9.40E-12 | Tnfaip6 |
| 10436596 | 2.715391 | 6.335418 | -15.0759 | 2.78E-13 | 9.40E-12 | 2810055G20Rik |
| 10595664 | 1.411727 | 9.21822 | -15.0705 | 2.80E-13 | 9.45E-12 | Tmed3 |
| 10521090 | -1.37265 | 8.114301 | 15.06923 | 2.80E-13 | 9.46E-12 | Tacc3 |
| 10412207 | 2.837483 | 9.03523 | -15.0631 | 2.83E-13 | 9.52E-12 | Gpx8 |
| 10396840 | -1.72539 | 5.294997 | 15.06308 | 2.83E-13 | 9.52E-12 | Rdh12 |
| 10517488 | 1.924383 | 6.255054 | -15.0451 | 2.90E-13 | 9.74E-12 | Ephb2 |
| 10575160 | 1.025214 | 7.766081 | -15.0362 | 2.93E-13 | 9.85E-12 | Nfat5 |
| 10499666 | 1.557785 | 6.65983 | -15.0335 | 2.94E-13 | 9.88E-12 | Atp8b2 |
| 10391461 | -1.89106 | 6.201281 | 15.03058 | 2.96E-13 | 9.90E-12 | Brca1 |
| 10532741 | 2.711793 | 8.166886 | -15.0283 | 2.97E-13 | 9.92E-12 | Tmem119 |
| 10489127 | -1.70124 | 6.824913 | 15.0275 | 2.97E-13 | 9.92E-12 | Rbl1 |
| 10544148 | -1.1803 | 8.398473 | 15.01971 | 3.00E-13 | 1.00E-11 | Kdm7a |
| 10580473 | 1.047878 | 6.734333 | -15.0192 | 3.00E-13 | 1.00E-11 | Zfp423 |
| 10514347 | 1.657082 | 6.096176 | -15.019 | 3.00E-13 | 1.00E-11 | Cdkn2b |
| 10358529 | 2.244624 | 5.553618 | -15.0154 | 3.02E-13 | 1.00E-11 | Hmcn1 |
| 10471608 | -1.17808 | 6.784043 | 14.99835 | 3.09E-13 | 1.03E-11 | Cntrl |
| 10347639 | 1.348035 | 7.002551 | -14.9882 | 3.13E-13 | 1.04E-11 | Gmppa |
| 10487945 | -1.1821 | 9.143982 | 14.98304 | 3.16E-13 | 1.05E-11 | Gpcpd1 |
| 10515500 | 1.371106 | 5.6885 | -14.9826 | 3.16E-13 | 1.05E-11 | Ccdc24 |
| 10458960 | 1.001975 | 5.888496 | -14.982 | 3.16E-13 | 1.05E-11 | Aldh7a1 |
| 10461979 | -2.38812 | 7.902027 | 14.98139 | 3.16E-13 | 1.05E-11 | Aldh1a1 |
| 10498379 | 2.602273 | 7.726974 | -14.9807 | 3.17E-13 | 1.05E-11 | Igsf10 |
| 10376332 | -1.6782 | 5.168457 | 14.97921 | 3.17E-13 | 1.05E-11 | 4930438A08Rik |
| 10373179 | 1.254328 | 5.982353 | -14.9668 | 3.23E-13 | 1.06E-11 | Gli1 |
| 10407985 | -2.63387 | 5.92857 | 14.96357 | 3.24E-13 | 1.07E-11 | Gpr141 |
| 10455852 | 1.315067 | 7.58092 | -14.9624 | 3.25E-13 | 1.07E-11 | Prrc1 |
| 10416215 | 1.766639 | 8.628296 | -14.9581 | 3.27E-13 | 1.07E-11 | Loxl2 |
| 10412011 | -1.19076 | 8.179889 | 14.95301 | 3.29E-13 | 1.08E-11 | Kif2a |
| 10423548 | 2.160901 | 8.148454 | -14.9504 | 3.30E-13 | 1.08E-11 | Sdc2 |
| 10567010 | 1.999338 | 6.361785 | -14.9502 | 3.30E-13 | 1.08E-11 | Dkk3 |
| 10491732 | 2.213227 | 6.858844 | -14.9494 | 3.31E-13 | 1.08E-11 | Fat4 |
| 10485117 | 3.147166 | 8.110228 | -14.9482 | 3.31E-13 | 1.08E-11 | Creb3l1 |
| 10550994 | -2.70982 | 6.125849 | 14.94779 | 3.31E-13 | 1.08E-11 | Ceacam10 |
| 10399198 | -1.31117 | 9.435693 | 14.94406 | 3.33E-13 | 1.08E-11 | Ncoa4 |
| 10456522 | 1.296255 | 8.328975 | -14.9383 | 3.36E-13 | 1.09E-11 | Tcf4 |
| 10354309 | 3.175135 | 8.071087 | -14.9382 | 3.36E-13 | 1.09E-11 | Col5a2 |
| 10423836 | 4.183811 | 8.31895 | -14.9347 | 3.37E-13 | 1.09E-11 | Cthrc1 |
| 10554752 | 2.708615 | 6.252371 | -14.9335 | 3.38E-13 | 1.10E-11 | Nox4 |
| 10583100 | -3.00894 | 9.349552 | 14.9244 | 3.42E-13 | 1.11E-11 | Mmp8 |
| 10419296 | -2.03738 | 6.95996 | 14.92367 | 3.42E-13 | 1.11E-11 | Wdhd1 |
| 10506274 | -1.10499 | 5.31297 | 14.91938 | 3.45E-13 | 1.11E-11 | Dnajc6 |
| 10446253 | -1.79708 | 6.375263 | 14.9137 | 3.47E-13 | 1.12E-11 | Vav1 |
| 10527801 | -1.68738 | 5.674836 | 14.90969 | 3.49E-13 | 1.13E-11 | Brca2 |
| 10577641 | 2.393394 | 6.508882 | -14.9053 | 3.51E-13 | 1.13E-11 | 1810011O10Rik |
| 10356423 | 1.134726 | 6.612632 | -14.9036 | 3.52E-13 | 1.13E-11 | Usp40 |
| 10576090 | -1.8167 | 6.985094 | 14.8943 | 3.57E-13 | 1.15E-11 | Zfpm1 |
| 10504755 | -2.98731 | 6.6355 | 14.88773 | 3.60E-13 | 1.15E-11 | Sympk |
| 10361995 | -1.70007 | 5.50339 | 14.87089 | 3.68E-13 | 1.18E-11 | Mtfr2 |
| 10459183 | 1.774884 | 6.689104 | -14.8648 | 3.72E-13 | 1.18E-11 | Slc26a2 |
| 10441791 | 1.615499 | 6.961658 | -14.8647 | 3.72E-13 | 1.18E-11 | Airn |
| 10594301 | 1.099676 | 5.296061 | -14.8599 | 3.74E-13 | 1.19E-11 | Coro2b |
| 10587446 | 1.471741 | 6.514871 | -14.8597 | 3.74E-13 | 1.19E-11 | Myo6 |
| 10472240 | 1.280279 | 6.1865 | -14.8579 | 3.75E-13 | 1.19E-11 | Tanc1 |
| 10541182 | -1.02264 | 8.250343 | 14.85569 | 3.76E-13 | 1.19E-11 | Fbxl14 |
| 10600017 | -1.27765 | 8.118708 | 14.85334 | 3.77E-13 | 1.20E-11 | Hmgb3 |
| 10379215 | 1.2596 | 8.615056 | -14.8487 | 3.80E-13 | 1.20E-11 | Ift20 |
| 10530201 | 1.290487 | 8.203642 | -14.8458 | 3.81E-13 | 1.21E-11 | Ugdh |
| 10488655 | -1.8759 | 7.982401 | 14.84562 | 3.82E-13 | 1.21E-11 | Bcl2l1 |
| 10384458 | -2.43433 | 8.61904 | 14.83133 | 3.89E-13 | 1.23E-11 | Plek |
| 10578322 | 2.393312 | 7.823555 | -14.8288 | 3.91E-13 | 1.23E-11 | Gm9868 |
| 10601768 | 1.346667 | 5.640313 | -14.826 | 3.92E-13 | 1.23E-11 | Armcx4 |
| 10554574 | -1.36422 | 8.482235 | 14.82394 | 3.93E-13 | 1.24E-11 | Tm6sf1 |
| 10424543 | 1.995592 | 7.163578 | -14.8165 | 3.97E-13 | 1.25E-11 | Wisp1 |
| 10523281 | 1.057178 | 8.733299 | -14.8092 | 4.01E-13 | 1.26E-11 | 44450 |
| 10539773 | 1.167316 | 8.181377 | -14.808 | 4.02E-13 | 1.26E-11 | Gfpt1 |
| 10474671 | 1.602732 | 7.664812 | -14.8028 | 4.05E-13 | 1.27E-11 | Spred1 |
| 10381939 | 1.026991 | 6.350092 | -14.7991 | 4.07E-13 | 1.27E-11 | Tanc2 |
| 10381798 | 2.312658 | 7.921117 | -14.7935 | 4.10E-13 | 1.28E-11 | Myl4 |
| 10351140 | 1.57069 | 6.469846 | -14.7875 | 4.14E-13 | 1.29E-11 | Kifap3 |
| 10370510 | 1.609593 | 6.446864 | -14.7824 | 4.17E-13 | 1.30E-11 | Syde1 |
| 10596166 | -1.97039 | 5.868737 | 14.77991 | 4.18E-13 | 1.30E-11 | 1300017J02Rik |
| 10358668 | 2.506733 | 6.189367 | -14.776 | 4.20E-13 | 1.31E-11 | Hmcn1 |
| 10492102 | 1.291636 | 6.420754 | -14.7728 | 4.22E-13 | 1.31E-11 | Spg20 |
| 10355806 | -1.61415 | 10.11977 | 14.77154 | 4.23E-13 | 1.31E-11 | Tuba4a |
| 10475532 | -1.31479 | 7.500144 | 14.76965 | 4.24E-13 | 1.31E-11 | Sqrdl |
| 10413839 | -1.25827 | 9.206724 | 14.76916 | 4.24E-13 | 1.31E-11 | Ncoa4 |
| 10539822 | 1.110683 | 7.824626 | -14.7686 | 4.25E-13 | 1.31E-11 | Copg1 |
| 10545707 | 1.805774 | 6.144071 | -14.7548 | 4.33E-13 | 1.34E-11 | Actg2 |
| 10371217 | -1.39796 | 5.941616 | 14.75008 | 4.36E-13 | 1.34E-11 | S1pr4 |
| 10451993 | 1.570362 | 7.569732 | -14.7498 | 4.36E-13 | 1.34E-11 | D17Wsu104e |
| 10592719 | 1.487375 | 6.927284 | -14.7492 | 4.36E-13 | 1.34E-11 | Oaf |
| 10544982 | -2.36552 | 7.620357 | 14.74561 | 4.38E-13 | 1.35E-11 | Nt5c3 |
| 10600765 | -1.40418 | 5.379992 | 14.74213 | 4.41E-13 | 1.35E-11 | Pcyt1b |
| 10439612 | 1.647144 | 6.366311 | -14.7417 | 4.41E-13 | 1.35E-11 | Boc |
| 10381345 | -1.42316 | 9.083382 | 14.73753 | 4.43E-13 | 1.36E-11 | Psme3 |
| 10399710 | -3.16811 | 7.593309 | 14.7267 | 4.50E-13 | 1.38E-11 | Rsad2 |
| 10534927 | -2.10696 | 8.030299 | 14.72348 | 4.52E-13 | 1.38E-11 | Pilra |
| 10403604 | -1.52141 | 8.15715 | 14.71937 | 4.55E-13 | 1.39E-11 | Lyst |
| 10477187 | -1.34844 | 7.320401 | 14.71171 | 4.60E-13 | 1.40E-11 | Tpx2 |
| 10499536 | 1.602017 | 6.294571 | -14.6932 | 4.72E-13 | 1.44E-11 | Efna1 |
| 10547153 | -2.46363 | 7.040453 | 14.69027 | 4.74E-13 | 1.44E-11 | Alox5 |
| 10497203 | 2.189956 | 6.829936 | -14.6845 | 4.78E-13 | 1.45E-11 | Hey1 |
| 10403229 | 1.461074 | 5.624396 | -14.6798 | 4.81E-13 | 1.46E-11 | Itgb8 |
| 10511363 | 2.492042 | 8.10605 | -14.6797 | 4.81E-13 | 1.46E-11 | Penk |
| 10443598 | -1.40868 | 4.882112 | 14.66542 | 4.90E-13 | 1.49E-11 | Dnah8 |
| 10501007 | -1.73352 | 4.62094 | 14.66501 | 4.91E-13 | 1.49E-11 | Chil5 |
| 10501734 | 1.756394 | 7.331749 | -14.6601 | 4.94E-13 | 1.49E-11 | Palmd |
| 10542953 | 1.542308 | 6.363975 | -14.6592 | 4.95E-13 | 1.49E-11 | Tfpi2 |
| 10399087 | -1.82264 | 6.816607 | 14.65818 | 4.96E-13 | 1.49E-11 | Ncapg2 |
| 10481870 | -1.03373 | 8.066905 | 14.65758 | 4.96E-13 | 1.49E-11 | Gapvd1 |
| 10436945 | 2.190028 | 7.244431 | -14.6481 | 5.03E-13 | 1.51E-11 | Slc5a3 |
| 10484318 | 1.274114 | 8.795505 | -14.644 | 5.05E-13 | 1.52E-11 | Nckap1 |
| 10536499 | 2.292581 | 8.16873 | -14.6401 | 5.08E-13 | 1.53E-11 | Cav1 |
| 10434559 | 1.352648 | 5.592013 | -14.6347 | 5.12E-13 | 1.54E-11 | Ephb3 |
| 10473125 | -2.25112 | 8.030047 | 14.62936 | 5.16E-13 | 1.55E-11 | Itga4 |
| 10466659 | -2.14392 | 8.89744 | 14.62828 | 5.17E-13 | 1.55E-11 | Gda |
| 10458583 | 1.620028 | 8.492208 | -14.624 | 5.20E-13 | 1.56E-11 | Yipf5 |
| 10457587 | 2.279249 | 7.14044 | -14.6201 | 5.23E-13 | 1.56E-11 | Zfp521 |
| 10345715 | 1.089338 | 7.947524 | -14.6074 | 5.32E-13 | 1.59E-11 | Map4k4 |
| 10595211 | 3.570475 | 9.461321 | -14.6063 | 5.33E-13 | 1.59E-11 | Col12a1 |
| 10492689 | 1.674509 | 6.661415 | -14.6057 | 5.33E-13 | 1.59E-11 | Pdgfc |
| 10472128 | -1.2756 | 7.137016 | 14.60166 | 5.36E-13 | 1.60E-11 | Arl6ip6 |
| 10503174 | -1.42278 | 7.166521 | 14.58884 | 5.46E-13 | 1.62E-11 | Chd7 |
| 10497920 | 1.069033 | 6.346132 | -14.5881 | 5.47E-13 | 1.62E-11 | Ankrd50 |
| 10358555 | 1.919814 | 6.47761 | -14.5867 | 5.48E-13 | 1.62E-11 | Hmcn1 |
| 10471067 | 1.735897 | 6.989432 | -14.5862 | 5.48E-13 | 1.62E-11 | Prrx2 |
| 10442495 | 1.353947 | 6.998245 | -14.5843 | 5.50E-13 | 1.63E-11 | Pkd1 |
| 10406504 | 3.133144 | 6.646267 | -14.583 | 5.51E-13 | 1.63E-11 | Edil3 |
| 10482920 | 1.436108 | 7.847591 | -14.58 | 5.53E-13 | 1.63E-11 | Cd302 |
| 10402665 | 1.296665 | 6.849955 | -14.57 | 5.61E-13 | 1.65E-11 | Cdc42bpb |
| 10389300 | -3.14628 | 7.291829 | 14.56574 | 5.64E-13 | 1.66E-11 | Dhrs11 |
| 10559478 | -2.3236 | 6.475027 | 14.56183 | 5.67E-13 | 1.67E-11 | Lilra6 |
| 10377725 | 1.02754 | 6.037432 | -14.5618 | 5.67E-13 | 1.67E-11 | Dlg4 |
| 10351603 | -1.78928 | 7.316673 | 14.55198 | 5.75E-13 | 1.69E-11 | Arhgap30 |
| 10603708 | 1.290645 | 7.75005 | -14.5479 | 5.79E-13 | 1.69E-11 | Cask |
| 10552380 | -1.69066 | 5.623499 | 14.54783 | 5.79E-13 | 1.69E-11 | Siglecg |
| 10434806 | 2.071987 | 8.365834 | -14.5402 | 5.85E-13 | 1.71E-11 | Lpp |
| 10382328 | 2.076634 | 6.899568 | -14.5367 | 5.88E-13 | 1.72E-11 | Sox9 |
| 10462343 | -1.64211 | 6.038053 | 14.52994 | 5.93E-13 | 1.73E-11 | Gm9895 |
| 10428763 | -1.38733 | 7.199588 | 14.51837 | 6.03E-13 | 1.76E-11 | Atad2 |
| 10493114 | 1.702523 | 5.056824 | -14.5178 | 6.04E-13 | 1.76E-11 | Nes |
| 10548385 | -2.37705 | 5.560326 | 14.51554 | 6.06E-13 | 1.76E-11 | Olr1 |
| 10593842 | 1.748528 | 8.313589 | -14.515 | 6.06E-13 | 1.76E-11 | Tspan3 |
| 10452047 | 1.735043 | 7.142651 | -14.515 | 6.06E-13 | 1.76E-11 | Ptprs |
| 10397416 | 1.272885 | 7.198302 | -14.5145 | 6.07E-13 | 1.76E-11 | Ift43 |
| 10350377 | 1.218272 | 6.76446 | -14.5021 | 6.17E-13 | 1.79E-11 | Zbtb41 |
| 10372177 | 2.252436 | 5.798657 | -14.5014 | 6.18E-13 | 1.79E-11 | Tmtc2 |
| 10476759 | 1.250534 | 7.506469 | -14.4956 | 6.23E-13 | 1.80E-11 | Rin2 |
| 10603417 | -2.55149 | 6.286489 | 14.49357 | 6.25E-13 | 1.80E-11 | Gata1 |
| 10388160 | 3.491306 | 7.179379 | -14.4922 | 6.26E-13 | 1.81E-11 | Slc13a5 |
| 10546967 | 1.03608 | 8.353699 | -14.4874 | 6.30E-13 | 1.82E-11 | Sec13 |
| 10527229 | -2.12884 | 8.005934 | 14.48671 | 6.31E-13 | 1.82E-11 | Fam220a |
| 10587554 | 1.896966 | 6.648655 | -14.4836 | 6.34E-13 | 1.82E-11 | Tpbg |
| 10574471 | 1.690275 | 8.69683 | -14.4816 | 6.35E-13 | 1.82E-11 | Cmtm3 |
| 10598586 | -2.00197 | 6.099047 | 14.48149 | 6.35E-13 | 1.82E-11 | Xk |
| 10350337 | 1.537026 | 5.564915 | -14.481 | 6.36E-13 | 1.82E-11 | A130050O07Rik |
| 10480275 | 1.191583 | 5.365383 | -14.4781 | 6.39E-13 | 1.83E-11 | Nebl |
| 10588243 | 1.727022 | 8.273528 | -14.4722 | 6.44E-13 | 1.84E-11 | Ryk |
| 10395320 | 2.243242 | 6.523311 | -14.466 | 6.50E-13 | 1.86E-11 | Twist1 |
| 10583242 | 1.291665 | 7.415249 | -14.4651 | 6.50E-13 | 1.86E-11 | Sesn3 |
| 10411728 | -1.97853 | 6.338627 | 14.4585 | 6.57E-13 | 1.87E-11 | Cenph |
| 10442098 | -1.5539 | 5.635668 | 14.45638 | 6.58E-13 | 1.88E-11 | Fpr2 |
| 10359307 | 3.32201 | 7.182435 | -14.4487 | 6.66E-13 | 1.89E-11 | Tnn |
| 10430993 | -1.19022 | 5.401526 | 14.44824 | 6.66E-13 | 1.89E-11 | 1700001L05Rik |
| 10581772 | 1.030825 | 8.507718 | -14.4455 | 6.69E-13 | 1.90E-11 | Glg1 |
| 10394627 | 1.029586 | 6.788929 | -14.4415 | 6.73E-13 | 1.91E-11 | Nbas |
| 10456346 | -1.23308 | 8.976311 | 14.441 | 6.73E-13 | 1.91E-11 | Sec11c |
| 10594251 | -1.52852 | 7.687783 | 14.43677 | 6.77E-13 | 1.92E-11 | Kif23 |
| 10359034 | 1.481468 | 7.228317 | -14.4354 | 6.78E-13 | 1.92E-11 | Qsox1 |
| 10485963 | -1.64347 | 7.432949 | 14.43274 | 6.81E-13 | 1.92E-11 | Arhgap11a |
| 10469695 | -1.38926 | 7.966235 | 14.42345 | 6.90E-13 | 1.95E-11 | Apbb1ip |
| 10578690 | -2.09325 | 6.946009 | 14.4182 | 6.95E-13 | 1.96E-11 | Neil3 |
| 10586227 | -1.58615 | 8.891917 | 14.41255 | 7.01E-13 | 1.97E-11 | Dennd4a |
| 10358533 | 2.086155 | 5.148623 | -14.4087 | 7.05E-13 | 1.98E-11 | Hmcn1 |
| 10458731 | 1.426732 | 6.261741 | -14.4067 | 7.07E-13 | 1.99E-11 | Mcc |
| 10507131 | -1.56616 | 6.752131 | 14.40213 | 7.11E-13 | 2.00E-11 | Tal1 |
| 10589703 | -3.18031 | 9.938374 | 14.39359 | 7.20E-13 | 2.02E-11 | Ltf |
| 10586357 | 2.5001 | 7.003388 | -14.3922 | 7.21E-13 | 2.02E-11 | Cilp |
| 10433096 | -3.36013 | 7.572702 | 14.39036 | 7.23E-13 | 2.02E-11 | Nfe2 |
| 10568568 | 1.113599 | 8.809598 | -14.3845 | 7.29E-13 | 2.04E-11 | Oat |
| 10473356 | -3.0243 | 8.486884 | 14.38413 | 7.30E-13 | 2.04E-11 | Ube2l6 |
| 10568024 | -2.04868 | 8.618146 | 14.37958 | 7.34E-13 | 2.05E-11 | Coro1a |
| 10375055 | -3.09587 | 7.104902 | 14.37655 | 7.38E-13 | 2.05E-11 | Hbq1b |
| 10391762 | 1.651982 | 5.84199 | -14.3739 | 7.40E-13 | 2.06E-11 | Gjc1 |
| 10507484 | 1.084148 | 7.922441 | -14.3723 | 7.42E-13 | 2.06E-11 | Eri3 |
| 10400006 | 1.547026 | 6.155443 | -14.3703 | 7.44E-13 | 2.07E-11 | Ahr |
| 10547410 | 1.183179 | 6.893125 | -14.3697 | 7.45E-13 | 2.07E-11 | Erc1 |
| 10348829 | 1.681789 | 6.351253 | -14.3612 | 7.54E-13 | 2.09E-11 | Farp2 |
| 10523891 | -1.72745 | 4.88089 | 14.35943 | 7.56E-13 | 2.09E-11 | Ube2d2b |
| 10440019 | 3.039571 | 6.790945 | -14.355 | 7.61E-13 | 2.10E-11 | Tmem45a |
| 10495967 | -1.58016 | 5.947644 | 14.35006 | 7.66E-13 | 2.12E-11 | Tifa |
| 10443391 | -1.22556 | 7.805448 | 14.34812 | 7.68E-13 | 2.12E-11 | Mapk14 |
| 10462683 | -1.28524 | 7.061914 | 14.34633 | 7.70E-13 | 2.12E-11 | Pcgf5 |
| 10510482 | 1.155888 | 6.947223 | -14.3419 | 7.75E-13 | 2.13E-11 | Clstn1 |
| 10427904 | 1.759789 | 5.519455 | -14.341 | 7.76E-13 | 2.14E-11 | Fbxl7 |
| 10358583 | 2.782323 | 5.873094 | -14.3369 | 7.80E-13 | 2.15E-11 | Hmcn1 |
| 10376787 | 1.567068 | 6.275262 | -14.3349 | 7.83E-13 | 2.15E-11 | B9d1 |
| 10491300 | 1.076777 | 7.602049 | -14.3333 | 7.84E-13 | 2.15E-11 | Skil |
| 10425207 | 1.330814 | 8.913418 | -14.333 | 7.85E-13 | 2.15E-11 | H1f0 |
| 10455123 | 1.329976 | 4.057687 | -14.3324 | 7.85E-13 | 2.15E-11 | Pcdhb19 |
| 10575209 | -1.41568 | 5.641974 | 14.33132 | 7.87E-13 | 2.15E-11 | A430107J10Rik |
| 10534216 | 1.065805 | 8.463614 | -14.3296 | 7.89E-13 | 2.16E-11 | Gtf2i |
| 10595404 | 1.186077 | 7.378623 | -14.3264 | 7.92E-13 | 2.16E-11 | Fam46a |
| 10484283 | 1.236799 | 5.303436 | -14.3262 | 7.92E-13 | 2.16E-11 | Pde1a |
| 10465912 | -1.44541 | 5.612532 | 14.32614 | 7.93E-13 | 2.16E-11 | Fen1 |
| 10390707 | -1.61973 | 9.228782 | 14.32062 | 7.99E-13 | 2.17E-11 | Top2a |
| 10355327 | -2.05609 | 6.376249 | 14.31946 | 8.00E-13 | 2.18E-11 | Bard1 |
| 10413482 | 1.548823 | 5.572325 | -14.3151 | 8.05E-13 | 2.18E-11 | Wnt5a |
| 10404407 | 1.574028 | 6.812022 | -14.3141 | 8.06E-13 | 2.19E-11 | Foxc1 |
| 10414527 | -1.47526 | 8.964738 | 14.31191 | 8.09E-13 | 2.19E-11 | Pnp2 |
| 10409804 | -1.21414 | 8.641934 | 14.30764 | 8.14E-13 | 2.20E-11 | Zcchc6 |
| 10542738 | 1.201292 | 6.810189 | -14.3067 | 8.15E-13 | 2.20E-11 | Rassf8 |
| 10378216 | -2.68259 | 6.864558 | 14.30145 | 8.21E-13 | 2.22E-11 | Atp2a3 |
| 10579636 | -1.49656 | 6.007372 | 14.30016 | 8.23E-13 | 2.22E-11 | Cyp4f18 |
| 10561212 | 1.506289 | 6.441787 | -14.2943 | 8.29E-13 | 2.23E-11 | Ltbp4 |
| 10547740 | 2.108077 | 7.216465 | -14.294 | 8.30E-13 | 2.23E-11 | C1s1 |
| 10586306 | 1.649175 | 5.853344 | -14.2934 | 8.30E-13 | 2.23E-11 | Igdcc4 |
| 10556381 | 1.411667 | 6.651406 | -14.2927 | 8.31E-13 | 2.23E-11 | Mical2 |
| 10354168 | -1.57615 | 6.894308 | 14.29193 | 8.32E-13 | 2.23E-11 | Tbc1d8 |
| 10393320 | -2.42475 | 7.843736 | 14.29152 | 8.33E-13 | 2.23E-11 | Ube2o |
| 10394770 | -1.0711 | 9.269883 | 14.28036 | 8.46E-13 | 2.27E-11 | Odc1 |
| 10412078 | -1.58343 | 6.348637 | 14.27372 | 8.54E-13 | 2.29E-11 | Gapt |
| 10419082 | -3.35031 | 7.303449 | 14.27214 | 8.56E-13 | 2.29E-11 | Fam213a |
| 10451851 | 1.660546 | 6.867629 | -14.2706 | 8.58E-13 | 2.29E-11 | Armcx3 |
| 10485711 | 2.807261 | 6.117328 | -14.2576 | 8.74E-13 | 2.33E-11 | Fibin |
| 10588899 | -1.25405 | 11.45255 | 14.25305 | 8.80E-13 | 2.34E-11 | Gpx1 |
| 10546294 | -2.49284 | 7.314388 | 14.25304 | 8.80E-13 | 2.34E-11 | Nup210 |
| 10590489 | 1.383648 | 4.953721 | -14.25 | 8.84E-13 | 2.35E-11 | Zfp105 |
| 10523727 | 2.014709 | 7.831096 | -14.2437 | 8.92E-13 | 2.37E-11 | Pkd2 |
| 10460371 | -1.46723 | 6.827048 | 14.24109 | 8.95E-13 | 2.38E-11 | Ptprcap |
| 10536294 | 1.72812 | 6.558936 | -14.2372 | 9.00E-13 | 2.39E-11 | Peg10 |
| 10394534 | 2.014935 | 6.376858 | -14.2344 | 9.04E-13 | 2.39E-11 | Osr1 |
| 10594092 | 1.512688 | 7.054316 | -14.2329 | 9.06E-13 | 2.40E-11 | Cd276 |
| 10568150 | -1.8382 | 6.671238 | 14.23234 | 9.06E-13 | 2.40E-11 | Kif22 |
| 10414537 | 1.529333 | 7.662537 | -14.2309 | 9.08E-13 | 2.40E-11 | Ang |
| 10407145 | -1.23615 | 6.893107 | 14.22782 | 9.12E-13 | 2.41E-11 | Mier3 |
| 10505132 | 1.471174 | 5.632364 | -14.2278 | 9.12E-13 | 2.41E-11 | Akap2 |
| 10377804 | -1.91251 | 8.207908 | 14.22205 | 9.20E-13 | 2.42E-11 | Arrb2 |
| 10542172 | -3.5113 | 6.570256 | 14.21852 | 9.25E-13 | 2.43E-11 | Clec1b |
| 10536917 | 1.578512 | 6.847658 | -14.2165 | 9.27E-13 | 2.44E-11 | Smo |
| 10463486 | -1.21727 | 7.620344 | 14.21567 | 9.28E-13 | 2.44E-11 | Btrc |
| 10587266 | -2.03284 | 7.366257 | 14.21369 | 9.31E-13 | 2.44E-11 | Gclc |
| 10527638 | -1.74394 | 9.530551 | 14.2021 | 9.47E-13 | 2.48E-11 | Alox5ap |
| 10376950 | 1.595821 | 9.536294 | -14.1848 | 9.71E-13 | 2.54E-11 | Pmp22 |
| 10476401 | 1.299455 | 6.153597 | -14.1806 | 9.76E-13 | 2.55E-11 | Plcb1 |
| 10548552 | -2.24661 | 6.33729 | 14.17894 | 9.79E-13 | 2.56E-11 | Klra2 |
| 10568363 | 1.784369 | 7.409123 | -14.1706 | 9.91E-13 | 2.59E-11 | Armcx3 |
| 10495147 | -1.38614 | 4.89717 | 14.16677 | 9.96E-13 | 2.59E-11 | Dennd2d |
| 10494565 | -1.37812 | 6.801756 | 14.16656 | 9.96E-13 | 2.59E-11 | Fmo5 |
| 10564624 | 1.370163 | 5.412901 | -14.1664 | 9.97E-13 | 2.59E-11 | St8sia2 |
| 10360985 | -1.61352 | 7.388402 | 14.15916 | 1.01E-12 | 2.62E-11 | Cenpf |
| 10566877 | 1.237903 | 7.418871 | -14.1583 | 1.01E-12 | 2.62E-11 | Sbf2 |
| 10358521 | 2.039639 | 5.405563 | -14.157 | 1.01E-12 | 2.62E-11 | Hmcn1 |
| 10513818 | -1.16503 | 9.51492 | 14.14846 | 1.02E-12 | 2.65E-11 | Stmn1 |
| 10574456 | -1.46858 | 6.330699 | 14.14466 | 1.03E-12 | 2.66E-11 | Cklf |
| 10352954 | -1.36529 | 8.018548 | 14.14261 | 1.03E-12 | 2.67E-11 | Hmgb3 |
| 10466224 | -3.10851 | 7.773407 | 14.13848 | 1.04E-12 | 2.68E-11 | Ms4a3 |
| 10419323 | -1.75042 | 7.353958 | 14.12772 | 1.05E-12 | 2.72E-11 | Dlgap5 |
| 10546432 | 2.021502 | 6.041823 | -14.1276 | 1.05E-12 | 2.72E-11 | Adamts9 |
| 10346651 | 1.311657 | 8.19468 | -14.1253 | 1.06E-12 | 2.72E-11 | Bmpr2 |
| 10431802 | 1.1861 | 8.60333 | -14.1204 | 1.06E-12 | 2.74E-11 | Twf1 |
| 10503376 | 1.84403 | 5.81538 | -14.1173 | 1.07E-12 | 2.75E-11 | Triqk |
| 10496204 | -1.57492 | 7.331193 | 14.11303 | 1.08E-12 | 2.76E-11 | Cenpe |
| 10380514 | -1.98275 | 7.789613 | 14.11291 | 1.08E-12 | 2.76E-11 | Fam117a |
| 10466521 | -1.41864 | 7.556034 | 14.10778 | 1.08E-12 | 2.78E-11 | Gcnt1 |
| 10563085 | 1.100983 | 8.406317 | -14.1047 | 1.09E-12 | 2.79E-11 | Fcgrt |
| 10464647 | -1.37669 | 5.988625 | 14.10245 | 1.09E-12 | 2.80E-11 | Tbc1d10c |
| 10600502 | -1.36766 | 6.726894 | 14.10098 | 1.10E-12 | 2.80E-11 | Fam220-ps |
| 10371321 | 1.308703 | 5.972798 | -14.0972 | 1.10E-12 | 2.81E-11 | Slc41a2 |
| 10545096 | -2.7878 | 5.109153 | 14.09719 | 1.10E-12 | 2.81E-11 | Mageb16 |
| 10552245 | 1.070736 | 6.389383 | -14.096 | 1.10E-12 | 2.81E-11 | Tshz3 |
| 10544383 | -4.53467 | 7.031679 | 14.0949 | 1.10E-12 | 2.82E-11 | Kel |
| 10572357 | 1.180996 | 8.719203 | -14.0939 | 1.11E-12 | 2.82E-11 | Cope |
| 10571680 | -1.61155 | 5.379043 | 14.08949 | 1.11E-12 | 2.83E-11 | Cenpu |
| 10587880 | 2.791396 | 7.355883 | -14.083 | 1.12E-12 | 2.86E-11 | Pcolce2 |
| 10367400 | 1.516337 | 6.292651 | -14.0642 | 1.16E-12 | 2.93E-11 | Mmp19 |
| 10360412 | -1.03902 | 4.542606 | 14.06306 | 1.16E-12 | 2.93E-11 | Olfr419 |
| 10529299 | -1.52584 | 7.798577 | 14.06057 | 1.16E-12 | 2.94E-11 | Slbp |
| 10507137 | -3.01204 | 7.041647 | 14.05969 | 1.16E-12 | 2.94E-11 | Pdzk1ip1 |
| 10408531 | 1.44542 | 7.132479 | -14.058 | 1.17E-12 | 2.95E-11 | Gmds |
| 10455128 | 1.050167 | 4.136863 | -14.0548 | 1.17E-12 | 2.96E-11 | Pcdhb20 |
| 10523012 | -1.98261 | 7.255601 | 14.05357 | 1.17E-12 | 2.96E-11 | Dck |
| 10399924 | -1.7656 | 7.157984 | 14.04064 | 1.20E-12 | 3.01E-11 | Pik3cg |
| 10443408 | -1.48786 | 6.360262 | 14.03755 | 1.20E-12 | 3.02E-11 | Mapk13 |
| 10488482 | -1.42904 | 5.314082 | 14.03556 | 1.20E-12 | 3.03E-11 | Acss1 |
| 10406287 | 1.144977 | 7.130281 | -14.0304 | 1.21E-12 | 3.05E-11 | Ttc37 |
| 10458430 | -1.24157 | 7.41583 | 14.02885 | 1.22E-12 | 3.05E-11 | Diap1 |
| 10470564 | 1.198013 | 6.111093 | -14.0259 | 1.22E-12 | 3.06E-11 | Ralgds |
| 10529824 | -1.82834 | 5.088716 | 14.01993 | 1.23E-12 | 3.08E-11 | Prom1 |
| 10505734 | 1.294095 | 5.3691 | -14.0124 | 1.25E-12 | 3.11E-11 | Adamtsl1 |
| 10562576 | 1.633414 | 6.978386 | -14.0114 | 1.25E-12 | 3.11E-11 | Plekhf1 |
| 10435565 | -1.56411 | 7.989828 | 14.00966 | 1.25E-12 | 3.12E-11 | Hcls1 |
| 10602020 | 2.032312 | 6.845185 | -14.0087 | 1.25E-12 | 3.12E-11 | Tbc1d8b |
| 10566543 | 1.643322 | 5.971633 | -13.9991 | 1.27E-12 | 3.15E-11 | Dchs1 |
| 10424683 | -3.89609 | 8.599272 | 13.99324 | 1.28E-12 | 3.18E-11 | Ly6g |
| 10457644 | 2.316661 | 6.957904 | -13.9844 | 1.30E-12 | 3.21E-11 | Cdh2 |
| 10470283 | 1.134905 | 7.037976 | -13.9707 | 1.32E-12 | 3.27E-11 | Egfl7 |
| 10495935 | -1.42219 | 6.029945 | 13.96997 | 1.32E-12 | 3.27E-11 | Zgrf1 |
| 10421737 | 1.191604 | 6.782455 | -13.9674 | 1.33E-12 | 3.28E-11 | Tnfsf11 |
| 10355864 | 1.240157 | 6.758577 | -13.9668 | 1.33E-12 | 3.28E-11 | Chpf |
| 10393881 | -1.04782 | 7.674556 | 13.96614 | 1.33E-12 | 3.28E-11 | Mafg |
| 10369252 | 1.492408 | 7.09725 | -13.9635 | 1.34E-12 | 3.29E-11 | 44449 |
| 10403462 | 1.584087 | 7.412828 | -13.9614 | 1.34E-12 | 3.30E-11 | Dip2c |
| 10569429 | 1.644049 | 7.27649 | -13.9551 | 1.35E-12 | 3.33E-11 | Cdkn1c |
| 10444752 | -2.12367 | 6.324806 | 13.9542 | 1.36E-12 | 3.33E-11 | Ltb |
| 10534202 | -2.07159 | 8.200364 | 13.95327 | 1.36E-12 | 3.33E-11 | Ncf1 |
| 10578574 | 1.045353 | 4.785304 | -13.9492 | 1.37E-12 | 3.34E-11 | Stox2 |
| 10411611 | -1.99496 | 5.326132 | 13.94897 | 1.37E-12 | 3.34E-11 | Naip5 |
| 10414262 | -3.05596 | 7.269955 | 13.94544 | 1.37E-12 | 3.36E-11 | Ear2 |
| 10525195 | -1.971 | 8.478808 | 13.94544 | 1.37E-12 | 3.36E-11 | Gm15800 |
| 10531931 | 2.274014 | 9.23288 | -13.9419 | 1.38E-12 | 3.37E-11 | Sparcl1 |
| 10358670 | 2.384244 | 6.172872 | -13.9412 | 1.38E-12 | 3.37E-11 | Hmcn1 |
| 10606858 | 1.026164 | 9.339035 | -13.9318 | 1.40E-12 | 3.41E-11 | Tceal8 |
| 10569020 | -2.90555 | 9.685924 | 13.92419 | 1.42E-12 | 3.44E-11 | Ifitm6 |
| 10435497 | -2.47371 | 8.741151 | 13.92391 | 1.42E-12 | 3.44E-11 | Stfa2l1 |
| 10606186 | 2.286103 | 7.04254 | -13.9235 | 1.42E-12 | 3.44E-11 | Slc16a2 |
| 10516481 | 1.626427 | 6.257487 | -13.9229 | 1.42E-12 | 3.44E-11 | Gja4 |
| 10364375 | 1.481152 | 9.247362 | -13.9222 | 1.42E-12 | 3.44E-11 | Cstb |
| 10454786 | 1.149915 | 8.138027 | -13.9189 | 1.43E-12 | 3.46E-11 | Ctnna1 |
| 10422760 | -1.84473 | 8.545409 | 13.91886 | 1.43E-12 | 3.46E-11 | Fyb |
| 10600169 | 1.870147 | 11.118 | -13.915 | 1.44E-12 | 3.47E-11 | Bgn |
| 10352661 | 1.319064 | 7.09884 | -13.9045 | 1.46E-12 | 3.52E-11 | Ptpn14 |
| 10432045 | 3.430201 | 7.803203 | -13.8926 | 1.48E-12 | 3.58E-11 | Col2a1 |
| 10496125 | 1.652742 | 6.447314 | -13.8893 | 1.49E-12 | 3.60E-11 | Dkk2 |
| 10502774 | 1.283664 | 6.718651 | -13.8857 | 1.50E-12 | 3.61E-11 | Lphn2 |
| 10495449 | 3.34552 | 8.233751 | -13.8856 | 1.50E-12 | 3.61E-11 | Col11a1 |
| 10392845 | -2.24476 | 6.509696 | 13.88325 | 1.50E-12 | 3.62E-11 | Cd300lf |
| 10490731 | 1.782841 | 7.194034 | -13.8829 | 1.50E-12 | 3.62E-11 | Sox18 |
| 10426368 | -1.29557 | 4.334441 | 13.88156 | 1.51E-12 | 3.62E-11 | Lrrk2 |
| 10508151 | -1.60817 | 6.047939 | 13.88094 | 1.51E-12 | 3.62E-11 | Clspn |
| 10386070 | 1.123459 | 9.47101 | -13.8793 | 1.51E-12 | 3.63E-11 | Atox1 |
| 10559385 | 1.688498 | 5.601867 | -13.8536 | 1.57E-12 | 3.76E-11 | Mrgprf |
| 10561004 | 1.385908 | 7.305708 | -13.8508 | 1.58E-12 | 3.77E-11 | Erf |
| 10542397 | -1.03022 | 8.041438 | 13.84733 | 1.58E-12 | 3.79E-11 | H2afj |
| 10357115 | 2.521934 | 6.090847 | -13.8429 | 1.60E-12 | 3.81E-11 | Dsel |
| 10378870 | 1.227587 | 7.288898 | -13.8413 | 1.60E-12 | 3.81E-11 | Git1 |
| 10576854 | 1.171072 | 6.206432 | -13.8412 | 1.60E-12 | 3.81E-11 | Ctxn1 |
| 10551542 | 1.081235 | 5.99988 | -13.8393 | 1.60E-12 | 3.82E-11 | Fbxo17 |
| 10346168 | -2.26108 | 5.447068 | 13.83623 | 1.61E-12 | 3.84E-11 | Stat4 |
| 10415413 | 2.012198 | 6.30676 | -13.8322 | 1.62E-12 | 3.86E-11 | Nynrin |
| 10602896 | 2.488773 | 5.840009 | -13.8203 | 1.65E-12 | 3.92E-11 | Gpr64 |
| 10519060 | -2.33627 | 7.898415 | 13.81668 | 1.66E-12 | 3.93E-11 | Tnfrsf14 |
| 10358733 | 1.111596 | 7.860752 | -13.816 | 1.66E-12 | 3.94E-11 | Rgl1 |
| 10436590 | 2.401274 | 5.585538 | -13.8149 | 1.66E-12 | 3.94E-11 | 2810055G20Rik |
| 10517587 | 3.112896 | 8.621057 | -13.81 | 1.67E-12 | 3.96E-11 | Alpl |
| 10580191 | 1.65987 | 9.444075 | -13.8092 | 1.68E-12 | 3.97E-11 | Nfix |
| 10391454 | 1.198075 | 7.601324 | -13.8076 | 1.68E-12 | 3.97E-11 | Vat1 |
| 10417053 | 1.149026 | 9.575772 | -13.8016 | 1.70E-12 | 4.00E-11 | Mbnl2 |
| 10503401 | 1.03242 | 7.497889 | -13.7869 | 1.73E-12 | 4.08E-11 | Tmem55a |
| 10522788 | -2.46141 | 6.112859 | 13.78317 | 1.74E-12 | 4.10E-11 | Stap1 |
| 10401068 | -2.56987 | 7.286232 | 13.78221 | 1.74E-12 | 4.11E-11 | Sptb |
| 10472181 | 1.715607 | 5.318167 | -13.7802 | 1.75E-12 | 4.11E-11 | Galnt5 |
| 10562368 | 1.081876 | 7.695689 | -13.7719 | 1.77E-12 | 4.16E-11 | 4931406P16Rik |
| 10431697 | -1.41226 | 6.505075 | 13.77091 | 1.77E-12 | 4.16E-11 | Abcd2 |
| 10594855 | 1.324664 | 6.618988 | -13.7699 | 1.78E-12 | 4.17E-11 | Cgnl1 |
| 10459391 | -2.01556 | 7.6546 | 13.76574 | 1.79E-12 | 4.19E-11 | Fech |
| 10579925 | 1.062391 | 7.369975 | -13.7628 | 1.79E-12 | 4.20E-11 | Gab1 |
| 10460196 | 1.474171 | 7.334842 | -13.7628 | 1.79E-12 | 4.20E-11 | 1810055G02Rik |
| 10544452 | 1.06414 | 4.76492 | -13.7618 | 1.80E-12 | 4.20E-11 | Fam115c |
| 10586246 | -1.7554 | 8.848811 | 13.76138 | 1.80E-12 | 4.20E-11 | Dennd4a |
| 10600688 | -1.51168 | 6.903227 | 13.75943 | 1.80E-12 | 4.21E-11 | Tab3 |
| 10490104 | -1.48419 | 7.374474 | 13.7509 | 1.83E-12 | 4.26E-11 | Aurka |
| 10353438 | 1.321775 | 7.302286 | -13.7493 | 1.83E-12 | 4.26E-11 | Tram2 |
| 10485198 | 1.748684 | 5.942223 | -13.7362 | 1.87E-12 | 4.34E-11 | Tspan18 |
| 10541729 | -1.91813 | 8.948051 | 13.73145 | 1.88E-12 | 4.37E-11 | Cdca3 |
| 10604347 | 2.455796 | 6.216499 | -13.7305 | 1.88E-12 | 4.37E-11 | Smarca1 |
| 10528385 | -1.05602 | 5.485025 | 13.72302 | 1.90E-12 | 4.42E-11 | Reln |
| 10463557 | 1.101405 | 7.840904 | -13.7179 | 1.92E-12 | 4.45E-11 | Gbf1 |
| 10592816 | -3.18481 | 8.449497 | 13.71674 | 1.92E-12 | 4.45E-11 | Hmbs |
| 10381962 | 1.756646 | 6.722728 | -13.7163 | 1.92E-12 | 4.45E-11 | Ace |
| 10437080 | 1.422202 | 7.947894 | -13.7161 | 1.92E-12 | 4.45E-11 | Ttc3 |
| 10579548 | 1.030997 | 9.133005 | -13.7147 | 1.93E-12 | 4.45E-11 | Unc13a |
| 10534389 | -4.32914 | 7.348369 | 13.71237 | 1.93E-12 | 4.46E-11 | Cldn13 |
| 10568174 | -1.93279 | 7.030257 | 13.71127 | 1.94E-12 | 4.47E-11 | Spn |
| 10469581 | 1.732594 | 6.792031 | -13.7073 | 1.95E-12 | 4.49E-11 | Etl4 |
| 10591620 | 1.268506 | 6.084502 | -13.6975 | 1.98E-12 | 4.55E-11 | Dock6 |
| 10492448 | 2.406371 | 6.530092 | -13.6967 | 1.98E-12 | 4.55E-11 | Ptx3 |
| 10359929 | 1.696041 | 7.391398 | -13.696 | 1.98E-12 | 4.55E-11 | Ddr2 |
| 10511498 | -1.19831 | 7.840178 | 13.69578 | 1.98E-12 | 4.55E-11 | Plekhf2 |
| 10409799 | -1.46577 | 8.104749 | 13.69258 | 1.99E-12 | 4.57E-11 | Isca1 |
| 10539263 | 1.972406 | 6.855167 | -13.6877 | 2.01E-12 | 4.60E-11 | Loxl3 |
| 10515848 | -3.74988 | 7.676359 | 13.68719 | 2.01E-12 | 4.60E-11 | Ermap |
| 10601854 | 1.728816 | 9.146399 | -13.6864 | 2.01E-12 | 4.60E-11 | Wbp5 |
| 10508986 | -1.13347 | 9.583921 | 13.67838 | 2.03E-12 | 4.65E-11 | Stmn1 |
| 10463462 | 1.106523 | 7.08292 | -13.673 | 2.05E-12 | 4.68E-11 | Sfxn3 |
| 10529082 | 1.033683 | 7.223552 | -13.673 | 2.05E-12 | 4.68E-11 | Mpv17 |
| 10478928 | 1.261648 | 6.265754 | -13.6692 | 2.06E-12 | 4.70E-11 | Tshz2 |
| 10450675 | -2.18009 | 6.50761 | 13.66102 | 2.09E-12 | 4.75E-11 | H2-T24 |
| 10360914 | 1.492025 | 6.370608 | -13.6603 | 2.09E-12 | 4.75E-11 | Lyplal1 |
| 10368720 | -2.2104 | 7.21386 | 13.66002 | 2.09E-12 | 4.75E-11 | Slc16a10 |
| 10354247 | 1.478015 | 7.24252 | -13.656 | 2.10E-12 | 4.78E-11 | Fhl2 |
| 10400896 | 1.751232 | 5.54604 | -13.6455 | 2.13E-12 | 4.84E-11 | L3hypdh |
| 10588942 | 1.379604 | 7.169474 | -13.6449 | 2.14E-12 | 4.84E-11 | Lamb2 |
| 10399314 | -3.26747 | 6.97857 | 13.64197 | 2.15E-12 | 4.86E-11 | Mfsd2b |
| 10548892 | -1.48242 | 10.27096 | 13.63626 | 2.16E-12 | 4.89E-11 | Arhgdib |
| 10556820 | 1.310887 | 6.282168 | -13.619 | 2.22E-12 | 5.01E-11 | Tmem159 |
| 10432640 | -2.80053 | 7.07994 | 13.61804 | 2.22E-12 | 5.01E-11 | Bin2 |
| 10356968 | 1.543792 | 8.218151 | -13.613 | 2.24E-12 | 5.05E-11 | Pam |
| 10454015 | 1.230373 | 5.359998 | -13.6121 | 2.24E-12 | 5.05E-11 | Ttc39c |
| 10591139 | 1.701786 | 5.036216 | -13.6079 | 2.26E-12 | 5.07E-11 | Naalad2 |
| 10402142 | -1.56489 | 6.894602 | 13.60765 | 2.26E-12 | 5.07E-11 | Ccdc88c |
| 10420877 | -2.02818 | 6.675523 | 13.60746 | 2.26E-12 | 5.07E-11 | Esco2 |
| 10450025 | -1.7495 | 8.34789 | 13.60081 | 2.28E-12 | 5.12E-11 | 44257 |
| 10351347 | -1.71011 | 9.326893 | 13.59111 | 2.31E-12 | 5.19E-11 | Creg1 |
| 10498584 | 1.729742 | 5.126917 | -13.5911 | 2.31E-12 | 5.19E-11 | Rarres1 |
| 10434094 | 1.109469 | 6.905485 | -13.5899 | 2.32E-12 | 5.19E-11 | Klhl22 |
| 10490818 | 1.449927 | 5.478532 | -13.5892 | 2.32E-12 | 5.19E-11 | Stmn2 |
| 10439651 | 1.889561 | 8.397675 | -13.5881 | 2.33E-12 | 5.20E-11 | Cd200 |
| 10487238 | -2.38386 | 7.328641 | 13.58536 | 2.33E-12 | 5.21E-11 | Hdc |
| 10535497 | -1.4068 | 5.038975 | 13.5828 | 2.34E-12 | 5.23E-11 | Ankrd61 |
| 10400483 | -2.56774 | 5.06301 | 13.58134 | 2.35E-12 | 5.23E-11 | Slc25a21 |
| 10594800 | -1.04872 | 8.197314 | 13.57671 | 2.36E-12 | 5.27E-11 | Fam63b |
| 10593219 | 1.646293 | 6.917499 | -13.5699 | 2.39E-12 | 5.32E-11 | Nnmt |
| 10482824 | 1.488038 | 6.456952 | -13.5656 | 2.40E-12 | 5.35E-11 | Acvr1 |
| 10601980 | 1.157679 | 4.280902 | -13.564 | 2.41E-12 | 5.35E-11 | Mum1l1 |
| 10489569 | 1.944663 | 8.101001 | -13.5616 | 2.42E-12 | 5.37E-11 | Pltp |
| 10492136 | 2.079474 | 5.79784 | -13.5558 | 2.44E-12 | 5.41E-11 | Dclk1 |
| 10376534 | 1.01444 | 8.347343 | -13.5451 | 2.48E-12 | 5.49E-11 | Mprip |
| 10498620 | -1.85945 | 8.238963 | 13.54441 | 2.48E-12 | 5.49E-11 | Trim59 |
| 10493798 | 1.560374 | 7.845559 | -13.5413 | 2.49E-12 | 5.51E-11 | S100a16 |
| 10583529 | -2.26004 | 5.935787 | 13.53924 | 2.50E-12 | 5.53E-11 | Icam4 |
| 10394471 | 1.823174 | 6.207943 | -13.5363 | 2.51E-12 | 5.54E-11 | Sdc1 |
| 10408200 | -2.10712 | 9.094028 | 13.53377 | 2.52E-12 | 5.56E-11 | Hist1h4f |
| 10364650 | -2.1095 | 7.460429 | 13.53275 | 2.53E-12 | 5.56E-11 | Hmha1 |
| 10596533 | 1.009747 | 6.633158 | -13.5275 | 2.54E-12 | 5.60E-11 | Tex264 |
| 10602372 | -3.33117 | 8.94514 | 13.52741 | 2.55E-12 | 5.60E-11 | Alas2 |
| 10461391 | -1.2758 | 10.2524 | 13.52611 | 2.55E-12 | 5.61E-11 | Pcna |
| 10400072 | 2.160104 | 5.139296 | -13.5257 | 2.55E-12 | 5.61E-11 | Scin |
| 10352709 | -1.23784 | 4.636446 | 13.51991 | 2.57E-12 | 5.65E-11 | Nsl1 |
| 10552824 | 1.341169 | 7.900712 | -13.517 | 2.59E-12 | 5.67E-11 | Rras |
| 10412559 | -1.91384 | 7.064796 | 13.5144 | 2.60E-12 | 5.69E-11 | Slbp |
| 10483264 | 1.140108 | 5.474566 | -13.5089 | 2.62E-12 | 5.73E-11 | Ttc21b |
| 10360090 | -2.18781 | 7.27938 | 13.50641 | 2.63E-12 | 5.75E-11 | Ppox |
| 10433691 | 1.096304 | 7.356767 | -13.499 | 2.66E-12 | 5.81E-11 | Ntan1 |
| 10504424 | 1.24615 | 6.849296 | -13.4976 | 2.66E-12 | 5.81E-11 | Reck |
| 10421526 | -1.41586 | 8.53515 | 13.49653 | 2.67E-12 | 5.82E-11 | Rb1 |
| 10588263 | 2.528274 | 7.130373 | -13.4956 | 2.67E-12 | 5.82E-11 | Slco2a1 |
| 10483865 | 2.899952 | 8.692679 | -13.4907 | 2.69E-12 | 5.86E-11 | Fkbp7 |
| 10451670 | -3.23472 | 5.902675 | 13.4893 | 2.69E-12 | 5.87E-11 | Tspo2 |
| 10589438 | 1.235844 | 8.549796 | -13.4852 | 2.71E-12 | 5.90E-11 | Map4 |
| 10529375 | 1.174506 | 8.483442 | -13.4681 | 2.78E-12 | 6.04E-11 | Mxd4 |
| 10419156 | -2.88268 | 8.20729 | 13.46592 | 2.79E-12 | 6.06E-11 | Ear2 |
| 10595402 | 1.22167 | 7.018248 | -13.4642 | 2.80E-12 | 6.07E-11 | Fam46a |
| 10552037 | 1.664523 | 6.71063 | -13.4607 | 2.81E-12 | 6.09E-11 | Sbsn |
| 10582241 | 1.670854 | 6.793054 | -13.4531 | 2.85E-12 | 6.16E-11 | Zcchc14 |
| 10576610 | 1.238633 | 5.956063 | -13.4493 | 2.86E-12 | 6.19E-11 | Pard3 |
| 10367532 | -1.32146 | 4.044608 | 13.44007 | 2.90E-12 | 6.27E-11 | Tespa1 |
| 10500610 | -3.22142 | 8.659144 | 13.43962 | 2.90E-12 | 6.27E-11 | Fam46c |
| 10546450 | 2.265662 | 6.432587 | -13.4366 | 2.92E-12 | 6.29E-11 | Adamts9 |
| 10435075 | -1.63833 | 9.557143 | 13.43619 | 2.92E-12 | 6.29E-11 | Tfrc |
| 10566583 | -2.07656 | 8.118341 | 13.4301 | 2.95E-12 | 6.34E-11 | Gm8995 |
| 10584124 | 1.200107 | 5.997252 | -13.4275 | 2.96E-12 | 6.36E-11 | Arhgap32 |
| 10597239 | 2.739962 | 7.702272 | -13.4209 | 2.99E-12 | 6.41E-11 | Pth1r |
| 10506118 | -1.19166 | 7.15156 | 13.42084 | 2.99E-12 | 6.41E-11 | Usp1 |
| 10573234 | 1.20051 | 6.583398 | -13.4183 | 3.00E-12 | 6.42E-11 | Lphn1 |
| 10562563 | -1.75453 | 7.186519 | 13.4181 | 3.00E-12 | 6.42E-11 | Ccne1 |
| 10392142 | -3.00347 | 7.503653 | 13.41769 | 3.00E-12 | 6.42E-11 | Cd79b |
| 10448124 | -2.83439 | 7.301115 | 13.41641 | 3.01E-12 | 6.43E-11 | Fpr1 |
| 10349968 | -2.4802 | 7.328915 | 13.41055 | 3.03E-12 | 6.48E-11 | Chil1 |
| 10567564 | -1.66737 | 8.306117 | 13.40939 | 3.04E-12 | 6.49E-11 | Cdr2 |
| 10522430 | 1.059441 | 6.031529 | -13.4042 | 3.06E-12 | 6.54E-11 | Dcun1d4 |
| 10433274 | 1.426972 | 8.085167 | -13.4006 | 3.08E-12 | 6.57E-11 | Vasn |
| 10548086 | -1.30346 | 6.818125 | 13.39891 | 3.09E-12 | 6.58E-11 | Rad51ap1 |
| 10497149 | 1.413416 | 7.655122 | -13.3967 | 3.10E-12 | 6.59E-11 | Wls |
| 10409345 | 1.289637 | 7.594969 | -13.3964 | 3.10E-12 | 6.59E-11 | Cltb |
| 10549760 | 1.121602 | 7.023996 | -13.3903 | 3.13E-12 | 6.65E-11 | Zfp580 |
| 10351867 | -1.69433 | 6.564099 | 13.38118 | 3.17E-12 | 6.74E-11 | Aim2 |
| 10386636 | 1.260779 | 7.360224 | -13.3784 | 3.18E-12 | 6.76E-11 | Usp22 |
| 10441361 | 1.370954 | 6.322223 | -13.3759 | 3.20E-12 | 6.78E-11 | Tiam2 |
| 10408321 | -1.65763 | 5.556473 | 13.37305 | 3.21E-12 | 6.80E-11 | Gmnn |
| 10584841 | -1.59704 | 5.630347 | 13.35863 | 3.28E-12 | 6.95E-11 | Amica1 |
| 10536818 | 1.731658 | 9.491341 | -13.3577 | 3.28E-12 | 6.95E-11 | Calu |
| 10400748 | -1.79562 | 4.4008 | 13.35702 | 3.29E-12 | 6.96E-11 | Cdkl1 |
| 10509163 | 1.946632 | 8.690187 | -13.3488 | 3.33E-12 | 7.04E-11 | Id3 |
| 10505894 | 1.072571 | 5.643833 | -13.348 | 3.33E-12 | 7.04E-11 | Mtap |
| 10541034 | -1.46775 | 5.939482 | 13.34272 | 3.36E-12 | 7.09E-11 | Zfand4 |
| 10433264 | 1.73502 | 7.851845 | -13.3418 | 3.36E-12 | 7.09E-11 | Glis2 |
| 10536908 | -3.36102 | 6.948447 | 13.33953 | 3.38E-12 | 7.11E-11 | Tspan33 |
| 10379127 | -1.85204 | 6.843997 | 13.33453 | 3.40E-12 | 7.15E-11 | Spag5 |
| 10355967 | -1.28442 | 4.678152 | 13.33396 | 3.40E-12 | 7.15E-11 | Ap1s3 |
| 10536494 | 2.104961 | 7.34774 | -13.3339 | 3.41E-12 | 7.15E-11 | Cav2 |
| 10406736 | -3.50502 | 6.873327 | 13.33376 | 3.41E-12 | 7.15E-11 | F2rl2 |
| 10384985 | 1.205961 | 6.459346 | -13.3295 | 3.43E-12 | 7.18E-11 | Rhbdf1 |
| 10382998 | -1.79099 | 8.442272 | 13.32842 | 3.43E-12 | 7.19E-11 | Birc5 |
| 10483706 | 2.152822 | 6.14441 | -13.3272 | 3.44E-12 | 7.20E-11 | Chrna1 |
| 10419850 | -2.01973 | 7.024692 | 13.326 | 3.45E-12 | 7.20E-11 | Cebpe |
| 10475932 | 1.464469 | 6.278491 | -13.3258 | 3.45E-12 | 7.20E-11 | Fbln7 |
| 10467470 | 1.237388 | 7.445477 | -13.3228 | 3.46E-12 | 7.23E-11 | Aldh18a1 |
| 10526952 | 1.711494 | 6.3128 | -13.319 | 3.48E-12 | 7.27E-11 | Gper1 |
| 10356932 | -1.3859 | 7.653608 | 13.31798 | 3.49E-12 | 7.27E-11 | D1Ertd622e |
| 10591967 | 1.201397 | 5.536723 | -13.3174 | 3.49E-12 | 7.27E-11 | Jam3 |
| 10487930 | -1.27082 | 10.25339 | 13.3136 | 3.51E-12 | 7.31E-11 | Pcna |
| 10477012 | 1.257561 | 8.731876 | -13.3131 | 3.51E-12 | 7.31E-11 | Fkbp1a |
| 10358515 | 1.745289 | 4.886999 | -13.3128 | 3.52E-12 | 7.31E-11 | Hmcn1 |
| 10604633 | 1.510119 | 6.967061 | -13.3104 | 3.53E-12 | 7.33E-11 | Cxx1b |
| 10527252 | -1.47746 | 8.122355 | 13.30899 | 3.54E-12 | 7.34E-11 | Eif2ak1 |
| 10504775 | 1.686606 | 6.619317 | -13.2954 | 3.61E-12 | 7.48E-11 | Col15a1 |
| 10460968 | -2.19285 | 7.512234 | 13.29114 | 3.63E-12 | 7.52E-11 | Rasgrp2 |
| 10474875 | -1.93202 | 8.433978 | 13.28952 | 3.64E-12 | 7.54E-11 | Casc5 |
| 10416181 | 1.050814 | 4.912029 | -13.2858 | 3.66E-12 | 7.58E-11 | Stc1 |
| 10407511 | -1.12782 | 10.42665 | 13.27684 | 3.71E-12 | 7.67E-11 | LOC102640615 |
| 10566580 | -2.16971 | 4.845866 | 13.27544 | 3.72E-12 | 7.68E-11 | Gm4759 |
| 10522182 | -1.23276 | 5.551862 | 13.26669 | 3.77E-12 | 7.78E-11 | Rhoh |
| 10570982 | 1.810921 | 7.844197 | -13.2621 | 3.80E-12 | 7.83E-11 | Fgfr1 |
| 10459421 | 1.599136 | 5.823453 | -13.2599 | 3.81E-12 | 7.85E-11 | Atp8b1 |
| 10572679 | 1.148064 | 8.009128 | -13.2591 | 3.81E-12 | 7.85E-11 | Glt25d1 |
| 10513739 | 2.013086 | 9.881316 | -13.2581 | 3.82E-12 | 7.85E-11 | Tnc |
| 10524124 | 1.025445 | 7.223772 | -13.2579 | 3.82E-12 | 7.85E-11 | Golga3 |
| 10585588 | 2.574603 | 7.357001 | -13.2576 | 3.82E-12 | 7.85E-11 | Cspg4 |
| 10486664 | -4.31416 | 7.081583 | 13.25594 | 3.83E-12 | 7.86E-11 | Epb4.2 |
| 10502778 | 1.331399 | 4.863216 | -13.2544 | 3.84E-12 | 7.87E-11 | Lphn2 |
| 10345807 | -2.0047 | 4.90418 | 13.25113 | 3.86E-12 | 7.91E-11 | Il18r1 |
| 10503334 | 1.613145 | 5.705364 | -13.2411 | 3.92E-12 | 8.01E-11 | Gem |
| 10443463 | 2.063281 | 8.114945 | -13.2399 | 3.93E-12 | 8.02E-11 | Cdkn1a |
| 10499062 | -2.73773 | 6.320433 | 13.23794 | 3.94E-12 | 8.04E-11 | Fhdc1 |
| 10475610 | -1.38131 | 7.864945 | 13.23599 | 3.95E-12 | 8.05E-11 | Dut |
| 10450116 | 1.149677 | 8.284426 | -13.2349 | 3.96E-12 | 8.06E-11 | Slc39a7 |
| 10554814 | 1.940127 | 7.793725 | -13.2311 | 3.98E-12 | 8.10E-11 | 1700019G06Rik |
| 10474902 | -1.44132 | 6.025263 | 13.22928 | 3.99E-12 | 8.11E-11 | Rad51 |
| 10534974 | -1.60523 | 7.673517 | 13.22798 | 4.00E-12 | 8.12E-11 | Mcm7 |
| 10505717 | 2.062844 | 6.139628 | -13.2256 | 4.01E-12 | 8.15E-11 | Adamtsl1 |
| 10453678 | 1.194841 | 7.655545 | -13.2235 | 4.03E-12 | 8.17E-11 | Zeb1 |
| 10541683 | 1.325989 | 7.006595 | -13.2199 | 4.05E-12 | 8.21E-11 | C1rb |
| 10407209 | -1.15968 | 6.192506 | 13.21914 | 4.05E-12 | 8.21E-11 | Slc38a9 |
| 10379636 | -2.89996 | 7.95081 | 13.21424 | 4.08E-12 | 8.25E-11 | Slfn4 |
| 10520553 | 1.277488 | 8.112328 | -13.2126 | 4.09E-12 | 8.25E-11 | Tmem214 |
| 10438378 | -1.38284 | 7.317617 | 13.21075 | 4.10E-12 | 8.26E-11 | Cdc45 |
| 10506870 | 1.005227 | 6.29096 | -13.2065 | 4.13E-12 | 8.31E-11 | Txndc12 |
| 10362896 | -1.01288 | 11.4982 | 13.20605 | 4.13E-12 | 8.31E-11 | Cd24a |
| 10530592 | -1.5898 | 7.857153 | 13.20526 | 4.14E-12 | 8.32E-11 | Fryl |
| 10421269 | 1.203705 | 6.678952 | -13.2043 | 4.15E-12 | 8.32E-11 | Sorbs3 |
| 10489694 | 1.236318 | 4.896917 | -13.1998 | 4.17E-12 | 8.37E-11 | Zfp334 |
| 10605437 | 2.749484 | 7.736388 | -13.1982 | 4.18E-12 | 8.39E-11 | Pls3 |
| 10408557 | -1.7693 | 8.846502 | 13.19719 | 4.19E-12 | 8.40E-11 | Serpinb1a |
| 10421172 | -3.54659 | 8.801653 | 13.19592 | 4.20E-12 | 8.41E-11 | Slc25a37 |
| 10526559 | -2.16718 | 6.474427 | 13.19197 | 4.22E-12 | 8.45E-11 | Ache |
| 10565794 | 2.355565 | 8.715718 | -13.187 | 4.26E-12 | 8.51E-11 | Serpinh1 |
| 10586244 | -1.7155 | 8.054577 | 13.18357 | 4.28E-12 | 8.55E-11 | Dennd4a |
| 10515836 | -1.68012 | 7.387478 | 13.18259 | 4.28E-12 | 8.56E-11 | Ccnb1 |
| 10540738 | -1.53073 | 5.436212 | 13.18114 | 4.29E-12 | 8.56E-11 | Fancd2 |
| 10474998 | -1.7633 | 5.404154 | 13.17224 | 4.35E-12 | 8.67E-11 | n-R5s204 |
| 10383970 | 1.501719 | 6.64931 | -13.1712 | 4.36E-12 | 8.67E-11 | Kremen1 |
| 10528546 | -1.02893 | 8.71819 | 13.16958 | 4.37E-12 | 8.69E-11 | Gabarapl2 |
| 10548875 | -2.13838 | 6.262522 | 13.16917 | 4.37E-12 | 8.69E-11 | Art4 |
| 10595126 | -1.43909 | 7.516856 | 13.16765 | 4.38E-12 | 8.70E-11 | Fbxo9 |
| 10596303 | -1.62603 | 5.214376 | 13.15916 | 4.44E-12 | 8.81E-11 | Acpp |
| 10459755 | -1.44178 | 5.657284 | 13.15117 | 4.50E-12 | 8.91E-11 | Ska1 |
| 10412100 | -1.23996 | 6.721343 | 13.14737 | 4.52E-12 | 8.95E-11 | Map3k1 |
| 10510700 | 1.763733 | 5.603978 | -13.1467 | 4.53E-12 | 8.95E-11 | Gpr153 |
| 10525591 | -1.68634 | 6.278131 | 13.14622 | 4.53E-12 | 8.95E-11 | Kntc1 |
| 10418991 | 1.023013 | 8.282833 | -13.146 | 4.53E-12 | 8.95E-11 | Ccser2 |
| 10602090 | -1.15501 | 8.254146 | 13.14531 | 4.54E-12 | 8.95E-11 | Atg4a |
| 10438445 | -1.96512 | 7.158 | 13.1401 | 4.57E-12 | 9.01E-11 | Klhl6 |
| 10379184 | 1.397839 | 5.991151 | -13.1399 | 4.57E-12 | 9.01E-11 | Slc46a1 |
| 10461251 | 1.74697 | 5.838166 | -13.1365 | 4.60E-12 | 9.05E-11 | Lrrn4cl |
| 10499560 | 1.028438 | 7.354927 | -13.135 | 4.61E-12 | 9.07E-11 | Adam15 |
| 10517165 | -1.62082 | 10.57018 | 13.13394 | 4.61E-12 | 9.08E-11 | Cd52 |
| 10436941 | 1.19165 | 8.222599 | -13.1323 | 4.63E-12 | 9.09E-11 | Mrps6 |
| 10503617 | -1.65055 | 5.816576 | 13.13215 | 4.63E-12 | 9.09E-11 | Mms22l |
| 10569646 | 1.763851 | 8.126274 | -13.1269 | 4.66E-12 | 9.16E-11 | Ccnd1 |
| 10451372 | 1.237432 | 5.706861 | -13.125 | 4.68E-12 | 9.18E-11 | Ptk7 |
| 10457168 | -2.78871 | 6.165053 | 13.12375 | 4.69E-12 | 9.19E-11 | Cd226 |
| 10575497 | 1.971891 | 7.52093 | -13.1212 | 4.71E-12 | 9.22E-11 | Mtss1l |
| 10434291 | -1.8988 | 5.443994 | 13.11942 | 4.72E-12 | 9.24E-11 | B3gnt5 |
| 10565924 | -1.00937 | 5.486051 | 13.1185 | 4.73E-12 | 9.24E-11 | Relt |
| 10570894 | -2.86523 | 7.81449 | 13.11662 | 4.74E-12 | 9.26E-11 | Ank1 |
| 10495685 | 1.960586 | 7.209347 | -13.1161 | 4.74E-12 | 9.26E-11 | Arhgap29 |
| 10375751 | 1.780425 | 7.726461 | -13.1109 | 4.78E-12 | 9.33E-11 | Adamts2 |
| 10551815 | 1.927784 | 8.066647 | -13.1105 | 4.78E-12 | 9.33E-11 | Zfp260 |
| 10461057 | 1.755299 | 6.29769 | -13.1059 | 4.82E-12 | 9.39E-11 | Rcor2 |
| 10541587 | -1.29257 | 8.060328 | 13.10375 | 4.83E-12 | 9.42E-11 | Clec4a2 |
| 10348618 | -1.4019 | 6.117153 | 13.10097 | 4.85E-12 | 9.45E-11 | Asb1 |
| 10433776 | 2.18442 | 8.486621 | -13.1007 | 4.86E-12 | 9.45E-11 | Snai2 |
| 10435288 | -1.62152 | 4.726625 | 13.09978 | 4.86E-12 | 9.45E-11 | Muc13 |
| 10407192 | -1.15123 | 7.472699 | 13.09785 | 4.88E-12 | 9.46E-11 | Slc38a9 |
| 10602428 | 1.359405 | 4.698547 | -13.0964 | 4.89E-12 | 9.48E-11 | Wnk3 |
| 10578880 | 1.544397 | 4.596563 | -13.0911 | 4.93E-12 | 9.55E-11 | Tll1 |
| 10396278 | -1.321 | 7.354429 | 13.08926 | 4.94E-12 | 9.57E-11 | Daam1 |
| 10353420 | -1.60126 | 6.984975 | 13.08578 | 4.97E-12 | 9.61E-11 | Mcm3 |
| 10487109 | -1.0918 | 5.321981 | 13.08385 | 4.98E-12 | 9.64E-11 | Cep152 |
| 10513381 | -1.0133 | 10.13769 | 13.0825 | 4.99E-12 | 9.65E-11 | Ptbp3 |
| 10478884 | 2.401792 | 7.373309 | -13.081 | 5.00E-12 | 9.67E-11 | Snai1 |
| 10467650 | -1.12082 | 6.02984 | 13.07976 | 5.01E-12 | 9.68E-11 | Frat2 |
| 10513256 | 1.704077 | 7.689295 | -13.0789 | 5.02E-12 | 9.68E-11 | Lpar1 |
| 10456140 | -1.42264 | 5.048816 | 13.07446 | 5.06E-12 | 9.73E-11 | Sh3tc2 |
| 10446771 | 1.076376 | 7.138665 | -13.0744 | 5.06E-12 | 9.73E-11 | Lclat1 |
| 10357870 | 1.855284 | 9.072347 | -13.0695 | 5.09E-12 | 9.80E-11 | Prelp |
| 10554445 | -1.64483 | 7.800902 | 13.05353 | 5.22E-12 | 1.00E-10 | Prc1 |
| 10459905 | 1.244066 | 6.926771 | -13.0499 | 5.25E-12 | 1.01E-10 | Setbp1 |
| 10545255 | -1.77981 | 6.370371 | 13.04774 | 5.27E-12 | 1.01E-10 | Rpia |
| 10563178 | -2.03647 | 7.40127 | 13.04668 | 5.28E-12 | 1.01E-10 | Cd37 |
| 10368534 | -1.10488 | 6.864152 | 13.04587 | 5.28E-12 | 1.01E-10 | Ncoa7 |
| 10416700 | 1.58842 | 6.231812 | -13.042 | 5.31E-12 | 1.02E-10 | Pcdh17 |
| 10479490 | 1.0273 | 8.082163 | -13.0393 | 5.34E-12 | 1.02E-10 | Arfgap1 |
| 10594221 | 1.407924 | 6.372341 | -13.036 | 5.36E-12 | 1.02E-10 | Lrrc49 |
| 10517791 | -2.26503 | 5.614582 | 13.02568 | 5.45E-12 | 1.04E-10 | Padi4 |
| 10366983 | -1.44165 | 5.727045 | 13.0212 | 5.49E-12 | 1.05E-10 | Tmem194 |
| 10521626 | 1.343305 | 6.117194 | -13.0151 | 5.54E-12 | 1.06E-10 | Cc2d2a |
| 10577164 | 1.794591 | 8.697106 | -13.0147 | 5.54E-12 | 1.06E-10 | Gas6 |
| 10434441 | 1.231981 | 5.637558 | -13.0137 | 5.55E-12 | 1.06E-10 | Ece2 |
| 10421351 | 1.123822 | 6.332106 | -13.0129 | 5.56E-12 | 1.06E-10 | Polr3d |
| 10376446 | -1.19304 | 6.765059 | 13.01142 | 5.57E-12 | 1.06E-10 | Trim17 |
| 10567297 | 1.520664 | 8.006782 | -13.0109 | 5.57E-12 | 1.06E-10 | Itpripl2 |
| 10499080 | 1.024006 | 7.251464 | -13.0095 | 5.59E-12 | 1.06E-10 | Arfip1 |
| 10455752 | 1.195171 | 6.35527 | -13.0082 | 5.60E-12 | 1.06E-10 | Snx24 |
| 10542965 | 1.291641 | 6.477981 | -13.0074 | 5.61E-12 | 1.06E-10 | Sgce |
| 10428619 | 2.036978 | 7.038404 | -12.9988 | 5.68E-12 | 1.08E-10 | Enpp2 |
| 10406733 | 1.375298 | 7.451065 | -12.9976 | 5.69E-12 | 1.08E-10 | Zbed3 |
| 10376396 | -1.83325 | 5.629137 | 12.99059 | 5.75E-12 | 1.09E-10 | Trim58 |
| 10571444 | 2.036385 | 6.579075 | -12.987 | 5.78E-12 | 1.09E-10 | Slc7a2 |
| 10568436 | 1.608711 | 7.611789 | -12.9815 | 5.83E-12 | 1.10E-10 | Fgfr2 |
| 10604637 | 1.433546 | 6.993036 | -12.9805 | 5.84E-12 | 1.10E-10 | Cxx1b |
| 10512489 | -1.22296 | 6.059707 | 12.97397 | 5.90E-12 | 1.11E-10 | Arhgef39 |
| 10497520 | -1.63214 | 6.747664 | 12.97248 | 5.92E-12 | 1.11E-10 | Ect2 |
| 10583326 | 1.163964 | 5.881039 | -12.9716 | 5.92E-12 | 1.12E-10 | Slc36a4 |
| 10371379 | 2.003454 | 6.679597 | -12.9624 | 6.01E-12 | 1.13E-10 | Nuak1 |
| 10488382 | 1.100828 | 8.712312 | -12.9612 | 6.02E-12 | 1.13E-10 | Cd93 |
| 10437639 | 1.732511 | 7.668084 | -12.9608 | 6.02E-12 | 1.13E-10 | Emp2 |
| 10438415 | -2.79424 | 5.374522 | 12.95688 | 6.06E-12 | 1.14E-10 | Iglv2 |
| 10541260 | -2.07625 | 5.438689 | 12.95637 | 6.06E-12 | 1.14E-10 | Cecr2 |
| 10445046 | -4.12123 | 6.931901 | 12.95506 | 6.08E-12 | 1.14E-10 | Trim10 |
| 10448743 | -1.08854 | 7.127856 | 12.95243 | 6.10E-12 | 1.14E-10 | Fahd1 |
| 10376434 | -3.3708 | 7.065119 | 12.9465 | 6.16E-12 | 1.15E-10 | Btnl10 |
| 10500982 | -2.35314 | 7.214942 | 12.94649 | 6.16E-12 | 1.15E-10 | I830077J02Rik |
| 10377927 | -1.3015 | 8.023787 | 12.94601 | 6.16E-12 | 1.15E-10 | Rnf167 |
| 10425866 | -2.15483 | 6.786067 | 12.94509 | 6.17E-12 | 1.15E-10 | Parvg |
| 10421950 | -1.59382 | 5.98025 | 12.94365 | 6.18E-12 | 1.15E-10 | Dach1 |
| 10365471 | -1.43123 | 6.597027 | 12.93797 | 6.24E-12 | 1.16E-10 | Fbxo7 |
| 10473312 | 2.68481 | 5.877598 | -12.9346 | 6.27E-12 | 1.17E-10 | Fam171b |
| 10485213 | -1.40391 | 8.334087 | 12.91601 | 6.46E-12 | 1.20E-10 | Cd82 |
| 10518147 | 2.447659 | 7.415419 | -12.9073 | 6.54E-12 | 1.22E-10 | Pdpn |
| 10523468 | -1.1238 | 7.886069 | 12.90301 | 6.59E-12 | 1.22E-10 | Bmp2k |
| 10513544 | 1.363392 | 5.491669 | -12.9015 | 6.60E-12 | 1.23E-10 | Zfp37 |
| 10368647 | 1.574196 | 6.698942 | -12.8985 | 6.63E-12 | 1.23E-10 | Dse |
| 10582626 | -2.33258 | 6.28218 | 12.89187 | 6.70E-12 | 1.24E-10 | Abcb10 |
| 10411595 | -1.46277 | 6.659915 | 12.88631 | 6.76E-12 | 1.25E-10 | Naip2 |
| 10455653 | -1.10003 | 4.73819 | 12.87075 | 6.92E-12 | 1.28E-10 | Tnfaip8 |
| 10474984 | -1.83391 | 8.266071 | 12.86887 | 6.95E-12 | 1.28E-10 | Nusap1 |
| 10421877 | -1.65293 | 7.055555 | 12.86107 | 7.03E-12 | 1.30E-10 | Diap3 |
| 10379489 | 1.952466 | 5.750274 | -12.8587 | 7.06E-12 | 1.30E-10 | Tmem98 |
| 10416800 | 1.650864 | 7.415469 | -12.853 | 7.12E-12 | 1.31E-10 | Lmo7 |
| 10406364 | 1.243632 | 5.167845 | -12.8462 | 7.19E-12 | 1.33E-10 | 2210408I21Rik |
| 10420261 | -2.83831 | 8.256681 | 12.84164 | 7.25E-12 | 1.34E-10 | Ctsg |
| 10574276 | -1.97529 | 6.591091 | 12.83923 | 7.27E-12 | 1.34E-10 | Gpr97 |
| 10500802 | -1.13962 | 8.382105 | 12.83641 | 7.30E-12 | 1.34E-10 | Atg4a |
| 10546725 | 1.521382 | 5.74742 | -12.8343 | 7.33E-12 | 1.35E-10 | Pdzrn3 |
| 10592891 | 1.311995 | 6.437458 | -12.8301 | 7.38E-12 | 1.36E-10 | Phldb1 |
| 10560015 | -1.01786 | 7.32715 | 12.82854 | 7.39E-12 | 1.36E-10 | Rnf141 |
| 10382106 | -1.87211 | 6.24193 | 12.82378 | 7.45E-12 | 1.37E-10 | Milr1 |
| 10358490 | 1.447538 | 4.976716 | -12.8202 | 7.49E-12 | 1.38E-10 | Hmcn1 |
| 10402715 | 1.066154 | 7.302147 | -12.8111 | 7.60E-12 | 1.39E-10 | Bag5 |
| 10350689 | -1.26763 | 6.968193 | 12.80797 | 7.64E-12 | 1.40E-10 | Ncf2 |
| 10564849 | 1.141009 | 6.098615 | -12.8058 | 7.66E-12 | 1.40E-10 | 2610034B18Rik |
| 10461723 | -1.53966 | 8.590596 | 12.80385 | 7.69E-12 | 1.41E-10 | Fam111a |
| 10598175 | -2.72943 | 7.942068 | 12.79829 | 7.75E-12 | 1.42E-10 | Ear10 |
| 10587829 | 3.187689 | 7.749572 | -12.797 | 7.77E-12 | 1.42E-10 | Plod2 |
| 10494388 | -1.63075 | 6.811828 | 12.78812 | 7.88E-12 | 1.44E-10 | Hist2h2be |
| 10462442 | 2.013557 | 5.4613 | -12.7836 | 7.93E-12 | 1.45E-10 | Il33 |
| 10413932 | 1.32115 | 7.379461 | -12.7771 | 8.01E-12 | 1.46E-10 | Vstm4 |
| 10601942 | 2.337797 | 5.032641 | -12.7704 | 8.10E-12 | 1.47E-10 | Nrk |
| 10454632 | 1.714931 | 6.183093 | -12.7702 | 8.10E-12 | 1.47E-10 | Camk4 |
| 10384154 | -1.7246 | 7.012763 | 12.76531 | 8.16E-12 | 1.48E-10 | Myo1g |
| 10408225 | -1.786 | 8.717213 | 12.76506 | 8.17E-12 | 1.48E-10 | Hist1h4c |
| 10542981 | -1.87469 | 9.57602 | 12.76424 | 8.18E-12 | 1.48E-10 | Gmfg |
| 10562637 | -1.58196 | 8.420732 | 12.76408 | 8.18E-12 | 1.48E-10 | Ccnb1 |
| 10377859 | 1.141101 | 5.726933 | -12.7586 | 8.25E-12 | 1.49E-10 | Pld2 |
| 10453604 | 1.898137 | 7.529011 | -12.7574 | 8.26E-12 | 1.49E-10 | Bambi |
| 10375614 | 1.545443 | 6.157631 | -12.7568 | 8.27E-12 | 1.49E-10 | Gfpt2 |
| 10573893 | 1.056278 | 6.767909 | -12.7551 | 8.29E-12 | 1.50E-10 | Fto |
| 10436304 | 2.241362 | 8.950982 | -12.7533 | 8.32E-12 | 1.50E-10 | Abi3bp |
| 10355871 | 1.045392 | 6.20744 | -12.7415 | 8.47E-12 | 1.53E-10 | Obsl1 |
| 10391831 | 1.527147 | 7.575065 | -12.7406 | 8.48E-12 | 1.53E-10 | Dcakd |
| 10455873 | 1.778396 | 7.839909 | -12.7401 | 8.49E-12 | 1.53E-10 | Slc12a2 |
| 10529034 | 2.995076 | 7.6902 | -12.7347 | 8.56E-12 | 1.54E-10 | Cgref1 |
| 10459866 | -3.15845 | 7.910647 | 12.73091 | 8.61E-12 | 1.55E-10 | Slc14a1 |
| 10542872 | 1.173383 | 7.045222 | -12.7208 | 8.75E-12 | 1.57E-10 | Rps4l |
| 10448676 | 1.192558 | 6.77879 | -12.7189 | 8.78E-12 | 1.57E-10 | Slc9a3r2 |
| 10466172 | -3.44213 | 6.067883 | 12.70642 | 8.95E-12 | 1.60E-10 | Ms4a1 |
| 10530156 | -1.54866 | 4.902248 | 12.70493 | 8.97E-12 | 1.61E-10 | Tmem156 |
| 10448506 | -1.82348 | 6.526431 | 12.70263 | 9.00E-12 | 1.61E-10 | Ccnf |
| 10350742 | -1.64943 | 7.209664 | 12.70151 | 9.02E-12 | 1.61E-10 | Rnasel |
| 10564527 | 1.2607 | 6.598742 | -12.6983 | 9.07E-12 | 1.62E-10 | Nr2f2 |
| 10545086 | -3.75515 | 8.158478 | 12.69543 | 9.11E-12 | 1.63E-10 | Snca |
| 10504757 | -1.44199 | 4.721216 | 12.6933 | 9.14E-12 | 1.63E-10 | BC005685 |
| 10421029 | -1.39728 | 6.527537 | 12.69086 | 9.17E-12 | 1.64E-10 | Cdca2 |
| 10389719 | 1.607842 | 8.238636 | -12.6857 | 9.25E-12 | 1.65E-10 | Scpep1 |
| 10465185 | -1.06916 | 7.120113 | 12.68076 | 9.32E-12 | 1.66E-10 | Ehbp1l1 |
| 10402268 | 1.872149 | 9.065453 | -12.6772 | 9.37E-12 | 1.67E-10 | Lgmn |
| 10546152 | 1.294311 | 5.90998 | -12.6753 | 9.40E-12 | 1.67E-10 | Podxl2 |
| 10591660 | -2.31343 | 6.249987 | 12.67027 | 9.47E-12 | 1.68E-10 | Epor |
| 10502335 | -2.80272 | 6.154434 | 12.66576 | 9.54E-12 | 1.69E-10 | Bank1 |
| 10511180 | 2.211186 | 9.58165 | -12.6569 | 9.67E-12 | 1.72E-10 | Mxra8 |
| 10408094 | -1.13266 | 11.34804 | 12.65565 | 9.69E-12 | 1.72E-10 | Hist1h2ao |
| 10389022 | 2.073881 | 8.870212 | -12.6548 | 9.71E-12 | 1.72E-10 | Myo1d |
| 10404069 | -2.33812 | 8.818749 | 12.65164 | 9.76E-12 | 1.73E-10 | Hist1h1a |
| 10549377 | 1.00477 | 5.322765 | -12.6499 | 9.78E-12 | 1.73E-10 | 1700034J05Rik |
| 10381187 | -1.13493 | 8.14862 | 12.64537 | 9.85E-12 | 1.74E-10 | Atp6v0a1 |
| 10564938 | -1.07049 | 7.16596 | 12.64452 | 9.87E-12 | 1.74E-10 | Fes |
| 10474825 | -1.07919 | 6.85487 | 12.62767 | 1.01E-11 | 1.79E-10 | Knstrn |
| 10364535 | -3.00085 | 8.45818 | 12.62582 | 1.02E-11 | 1.79E-10 | Elane |
| 10575861 | 1.057595 | 10.1701 | -12.6191 | 1.03E-11 | 1.81E-10 | Hsbp1 |
| 10372028 | -1.43377 | 7.073674 | 12.61153 | 1.04E-11 | 1.83E-10 | Plxnc1 |
| 10358613 | 1.414322 | 4.511783 | -12.6101 | 1.04E-11 | 1.83E-10 | Hmcn1 |
| 10498647 | 1.529955 | 6.477664 | -12.5922 | 1.07E-11 | 1.88E-10 | B3galnt1 |
| 10468980 | -1.65206 | 7.123753 | 12.58831 | 1.08E-11 | 1.89E-10 | Fam107b |
| 10408118 | -1.15895 | 11.31639 | 12.58703 | 1.08E-11 | 1.89E-10 | Hist1h2ao |
| 10382449 | -1.07939 | 4.95022 | 12.57573 | 1.10E-11 | 1.92E-10 | Rab37 |
| 10364109 | -3.15794 | 6.423549 | 12.57499 | 1.10E-11 | 1.93E-10 | Vpreb3 |
| 10406270 | -1.55889 | 8.026514 | 12.57457 | 1.10E-11 | 1.93E-10 | Glrx |
| 10511429 | 1.80236 | 5.694578 | -12.5702 | 1.11E-11 | 1.94E-10 | Car8 |
| 10496656 | 1.643435 | 6.387166 | -12.5652 | 1.12E-11 | 1.95E-10 | Col24a1 |
| 10440738 | -1.16415 | 5.996335 | 12.56452 | 1.12E-11 | 1.95E-10 | Tiam1 |
| 10474769 | -1.40015 | 7.331667 | 12.56258 | 1.12E-11 | 1.96E-10 | Bub1b |
| 10415411 | 1.50147 | 6.210189 | -12.5589 | 1.13E-11 | 1.97E-10 | Nynrin |
| 10475362 | -1.60495 | 6.481793 | 12.55654 | 1.13E-11 | 1.97E-10 | Wdr76 |
| 10408741 | 1.513205 | 8.140044 | -12.5537 | 1.14E-11 | 1.98E-10 | Txndc5 |
| 10575548 | -1.76582 | 7.094143 | 12.55369 | 1.14E-11 | 1.98E-10 | Gm26132 |
| 10570068 | 1.394929 | 6.476046 | -12.5535 | 1.14E-11 | 1.98E-10 | Col4a2 |
| 10358666 | 2.224956 | 6.585757 | -12.5492 | 1.15E-11 | 1.99E-10 | Hmcn1 |
| 10535927 | 1.079438 | 11.4075 | -12.5485 | 1.15E-11 | 1.99E-10 | Gm5566 |
| 10380896 | 1.281725 | 5.77275 | -12.5402 | 1.16E-11 | 2.01E-10 | Erbb2 |
| 10554325 | -1.67506 | 6.094916 | 12.53918 | 1.17E-11 | 2.02E-10 | Ticrr |
| 10408085 | -1.17478 | 11.27913 | 12.53917 | 1.17E-11 | 2.02E-10 | Hist1h2ao |
| 10554005 | 1.035203 | 8.488162 | -12.5337 | 1.18E-11 | 2.03E-10 | Vimp |
| 10583207 | 1.004939 | 7.089662 | -12.531 | 1.18E-11 | 2.04E-10 | Maml2 |
| 10503212 | -1.36652 | 6.29518 | 12.52563 | 1.19E-11 | 2.05E-10 | Chd7 |
| 10596137 | 1.096509 | 7.314218 | -12.5173 | 1.21E-11 | 2.08E-10 | Srprb |
| 10586242 | -1.68823 | 8.927551 | 12.51513 | 1.21E-11 | 2.09E-10 | Dennd4a |
| 10600936 | 1.654263 | 6.526033 | -12.5094 | 1.22E-11 | 2.10E-10 | Efnb1 |
| 10499766 | 1.090907 | 4.726673 | -12.5091 | 1.22E-11 | 2.10E-10 | Slc27a3 |
| 10599686 | 1.460996 | 5.291002 | -12.5069 | 1.23E-11 | 2.11E-10 | Zfp449 |
| 10581013 | 1.809858 | 9.870299 | -12.4965 | 1.25E-11 | 2.14E-10 | Cdh11 |
| 10470775 | 1.501845 | 7.37189 | -12.4894 | 1.26E-11 | 2.16E-10 | Cercam |
| 10351455 | 2.200147 | 9.573402 | -12.4889 | 1.26E-11 | 2.16E-10 | Rgs5 |
| 10596815 | -1.8227 | 7.802676 | 12.48711 | 1.27E-11 | 2.17E-10 | Rnf123 |
| 10502191 | 1.465792 | 9.905364 | -12.4834 | 1.27E-11 | 2.18E-10 | Ostc |
| 10384622 | 1.345635 | 6.484242 | -12.4833 | 1.27E-11 | 2.18E-10 | Ehbp1 |
| 10501879 | 1.102525 | 6.626106 | -12.48 | 1.28E-11 | 2.19E-10 | Usp53 |
| 10593293 | 1.14298 | 7.422336 | -12.4798 | 1.28E-11 | 2.19E-10 | Ncam1 |
| 10442762 | -2.95567 | 6.873621 | 12.47744 | 1.29E-11 | 2.20E-10 | Prss34 |
| 10530733 | 1.16334 | 7.682785 | -12.4765 | 1.29E-11 | 2.20E-10 | Clock |
| 10413803 | 1.049387 | 5.899072 | -12.473 | 1.29E-11 | 2.21E-10 | Btd |
| 10438639 | -1.92469 | 5.970223 | 12.47198 | 1.30E-11 | 2.21E-10 | Dgkg |
| 10492540 | -1.24951 | 5.262985 | 12.46753 | 1.31E-11 | 2.22E-10 | Il12a |
| 10576088 | 1.478323 | 6.294616 | -12.4659 | 1.31E-11 | 2.23E-10 | Gm22 |
| 10373073 | 1.516555 | 6.030366 | -12.4644 | 1.31E-11 | 2.23E-10 | Arhgef25 |
| 10414333 | 1.417286 | 6.86599 | -12.463 | 1.32E-11 | 2.23E-10 | Samd4 |
| 10382376 | 1.779006 | 7.012284 | -12.4588 | 1.32E-11 | 2.25E-10 | Ttyh2 |
| 10408243 | -1.11386 | 11.45369 | 12.45388 | 1.33E-11 | 2.26E-10 | Hist1h4a |
| 10375432 | -1.48638 | 4.936121 | 12.45339 | 1.34E-11 | 2.27E-10 | Fndc9 |
| 10534253 | 1.003681 | 5.919957 | -12.4523 | 1.34E-11 | 2.27E-10 | Gtf2ird1 |
| 10512709 | -1.36961 | 8.792778 | 12.45202 | 1.34E-11 | 2.27E-10 | Slc25a51 |
| 10418927 | 1.76715 | 7.416407 | -12.4506 | 1.34E-11 | 2.27E-10 | Bmpr1a |
| 10550509 | -2.89648 | 9.353233 | 12.44713 | 1.35E-11 | 2.28E-10 | Pglyrp1 |
| 10379630 | -1.59913 | 8.218133 | 12.43777 | 1.37E-11 | 2.31E-10 | Slfn2 |
| 10550131 | -1.23876 | 4.484471 | 12.43765 | 1.37E-11 | 2.31E-10 | Pla2g4c |
| 10602198 | 1.913208 | 5.573585 | -12.4362 | 1.37E-11 | 2.31E-10 | Pak3 |
| 10542993 | 1.293647 | 7.751551 | -12.4355 | 1.37E-11 | 2.32E-10 | Pon3 |
| 10405216 | -1.8891 | 7.307808 | 12.43478 | 1.38E-11 | 2.32E-10 | Syk |
| 10567049 | 1.395388 | 9.121944 | -12.4331 | 1.38E-11 | 2.32E-10 | Copb1 |
| 10502780 | 1.36705 | 6.873858 | -12.4317 | 1.38E-11 | 2.33E-10 | Lphn2 |
| 10539135 | 1.406332 | 8.322609 | -12.4293 | 1.39E-11 | 2.33E-10 | Capg |
| 10403938 | -1.10001 | 11.32181 | 12.42433 | 1.40E-11 | 2.35E-10 | Hist1h2ao |
| 10364559 | -1.07984 | 6.276613 | 12.42244 | 1.40E-11 | 2.36E-10 | Arid3a |
| 10576034 | -1.61059 | 7.03492 | 12.41719 | 1.41E-11 | 2.37E-10 | Irf8 |
| 10462796 | -1.87639 | 8.268363 | 12.41661 | 1.42E-11 | 2.37E-10 | Kif11 |
| 10404045 | -1.09621 | 11.41064 | 12.41641 | 1.42E-11 | 2.37E-10 | Hist1h2ao |
| 10379026 | -1.35864 | 3.312545 | 12.41452 | 1.42E-11 | 2.38E-10 | Mir144 |
| 10595979 | 1.712088 | 7.133682 | -12.4132 | 1.42E-11 | 2.38E-10 | Mras |
| 10404026 | -1.10904 | 11.39974 | 12.41156 | 1.43E-11 | 2.38E-10 | Hist1h2ao |
| 10595159 | 1.064409 | 6.915668 | -12.4007 | 1.45E-11 | 2.42E-10 | Ooep |
| 10403063 | -2.3015 | 5.941336 | 12.3999 | 1.45E-11 | 2.42E-10 | Ighv8-12 |
| 10408111 | -1.11829 | 11.43843 | 12.39575 | 1.46E-11 | 2.44E-10 | Hist1h2ao |
| 10524266 | -1.36301 | 5.974819 | 12.3927 | 1.47E-11 | 2.45E-10 | Chek2 |
| 10418092 | 1.791196 | 5.403864 | -12.3925 | 1.47E-11 | 2.45E-10 | A830039N20Rik |
| 10375307 | 1.378817 | 5.839083 | -12.39 | 1.48E-11 | 2.46E-10 | C1qtnf2 |
| 10576581 | 2.115235 | 6.624493 | -12.3873 | 1.48E-11 | 2.46E-10 | Kcnk1 |
| 10562709 | -1.94314 | 8.484364 | 12.38399 | 1.49E-11 | 2.48E-10 | Cd33 |
| 10417212 | 2.38817 | 7.782004 | -12.3762 | 1.51E-11 | 2.51E-10 | Itgbl1 |
| 10403955 | -1.11778 | 11.4427 | 12.37029 | 1.53E-11 | 2.53E-10 | Hist1h2ao |
| 10593123 | 1.485739 | 6.306844 | -12.3695 | 1.53E-11 | 2.53E-10 | Tagln |
| 10345967 | 1.151991 | 5.06516 | -12.3661 | 1.54E-11 | 2.54E-10 | Bivm |
| 10345675 | 1.520642 | 6.083314 | -12.3561 | 1.56E-11 | 2.57E-10 | Npas2 |
| 10393106 | 1.122899 | 6.361617 | -12.3554 | 1.56E-11 | 2.58E-10 | Trim47 |
| 10564978 | -1.79077 | 6.162291 | 12.35408 | 1.57E-11 | 2.58E-10 | Blm |
| 10357590 | -2.1355 | 6.543417 | 12.35148 | 1.57E-11 | 2.59E-10 | Dyrk3 |
| 10579609 | -2.01466 | 5.130928 | 12.35067 | 1.57E-11 | 2.59E-10 | Fcho1 |
| 10418410 | -1.2987 | 8.363624 | 12.35027 | 1.57E-11 | 2.59E-10 | Prkcd |
| 10459363 | 1.125955 | 4.797261 | -12.346 | 1.59E-11 | 2.61E-10 | Piezo2 |
| 10530692 | 1.251071 | 6.361684 | -12.3403 | 1.60E-11 | 2.63E-10 | Kdr |
| 10547322 | 1.038164 | 5.286849 | -12.3361 | 1.61E-11 | 2.65E-10 | Cacna1c |
| 10416533 | 1.663429 | 6.329117 | -12.3313 | 1.62E-11 | 2.66E-10 | Ccdc122 |
| 10361234 | -1.59347 | 6.433979 | 12.32909 | 1.63E-11 | 2.67E-10 | Hsd11b1 |
| 10383731 | 1.142404 | 8.075874 | -12.329 | 1.63E-11 | 2.67E-10 | Smtn |
| 10559248 | -2.05295 | 6.363731 | 12.32881 | 1.63E-11 | 2.67E-10 | Tspan32 |
| 10476314 | 1.16906 | 7.776271 | -12.3239 | 1.64E-11 | 2.69E-10 | Prnp |
| 10535043 | 1.02378 | 7.027223 | -12.3238 | 1.64E-11 | 2.69E-10 | Pdgfa |
| 10497673 | 1.074025 | 5.855472 | -12.3231 | 1.65E-11 | 2.69E-10 | Zmat3 |
| 10391332 | 1.168938 | 7.269188 | -12.3181 | 1.66E-11 | 2.71E-10 | Ptrf |
| 10358575 | 1.732244 | 5.174934 | -12.3166 | 1.66E-11 | 2.72E-10 | Hmcn1 |
| 10590808 | 1.21581 | 6.981834 | -12.313 | 1.67E-11 | 2.73E-10 | Yap1 |
| 10438904 | 2.127157 | 6.402469 | -12.3098 | 1.68E-11 | 2.74E-10 | Lrrc15 |
| 10347921 | -2.02847 | 6.78209 | 12.30855 | 1.68E-11 | 2.74E-10 | A530040E14Rik |
| 10358635 | 1.187966 | 5.525099 | -12.3063 | 1.69E-11 | 2.75E-10 | Hmcn1 |
| 10356271 | -1.59822 | 7.596889 | 12.30315 | 1.70E-11 | 2.77E-10 | A530032D15Rik |
| 10420758 | -1.27405 | 5.268463 | 12.30131 | 1.70E-11 | 2.77E-10 | Blk |
| 10356999 | -1.0605 | 10.37166 | 12.28758 | 1.74E-11 | 2.83E-10 | Gm7110 |
| 10507840 | 1.788169 | 6.204186 | -12.2784 | 1.77E-11 | 2.87E-10 | Heyl |
| 10565862 | -1.11939 | 7.086553 | 12.27769 | 1.77E-11 | 2.87E-10 | Pold3 |
| 10445789 | -2.27485 | 7.481139 | 12.27524 | 1.78E-11 | 2.88E-10 | Treml1 |
| 10357875 | -1.91627 | 8.00303 | 12.2735 | 1.78E-11 | 2.89E-10 | Btg2 |
| 10451604 | 1.018683 | 6.202018 | -12.2732 | 1.78E-11 | 2.89E-10 | Mdfi |
| 10521757 | -1.23216 | 6.77341 | 12.27286 | 1.78E-11 | 2.89E-10 | Gm7931 |
| 10359762 | -1.42662 | 7.46823 | 12.2683 | 1.80E-11 | 2.91E-10 | Rcsd1 |
| 10436100 | -2.56234 | 10.87193 | 12.26024 | 1.82E-11 | 2.94E-10 | Retnlg |
| 10421970 | -1.89027 | 4.289868 | 12.25401 | 1.84E-11 | 2.97E-10 | Gm25831 |
| 10498345 | -1.57791 | 6.189945 | 12.2462 | 1.86E-11 | 3.00E-10 | Gpr171 |
| 10438907 | -3.14748 | 7.41932 | 12.24562 | 1.86E-11 | 3.00E-10 | Gp5 |
| 10354506 | -1.02639 | 6.136212 | 12.24539 | 1.86E-11 | 3.00E-10 | Mfsd6 |
| 10418868 | -1.40903 | 5.717422 | 12.24082 | 1.88E-11 | 3.02E-10 | Wdfy4 |
| 10389025 | 1.149053 | 8.311433 | -12.2381 | 1.89E-11 | 3.03E-10 | Myo1d |
| 10506767 | 1.270841 | 5.40153 | -12.238 | 1.89E-11 | 3.03E-10 | Echdc2 |
| 10548857 | -1.40864 | 8.457314 | 12.23535 | 1.89E-11 | 3.05E-10 | Hist4h4 |
| 10363350 | 1.763889 | 6.914338 | -12.233 | 1.90E-11 | 3.05E-10 | P4ha1 |
| 10348240 | 3.412943 | 7.223853 | -12.2325 | 1.90E-11 | 3.06E-10 | 3110079O15Rik |
| 10467013 | 1.280836 | 6.554522 | -12.231 | 1.91E-11 | 3.06E-10 | Prkg1 |
| 10408072 | -1.10874 | 11.43111 | 12.22865 | 1.92E-11 | 3.07E-10 | Hist1h2ao |
| 10605674 | -1.54795 | 7.088811 | 12.22852 | 1.92E-11 | 3.07E-10 | Pola1 |
| 10403957 | -1.19383 | 11.46572 | 12.22834 | 1.92E-11 | 3.07E-10 | Hist1h4m |
| 10408092 | -1.19383 | 11.46572 | 12.22834 | 1.92E-11 | 3.07E-10 | Hist1h4m |
| 10527649 | 2.582985 | 5.762358 | -12.227 | 1.92E-11 | 3.07E-10 | Medag |
| 10425037 | -3.09029 | 7.426526 | 12.2142 | 1.96E-11 | 3.13E-10 | Apol10a |
| 10581890 | 1.129041 | 6.390921 | -12.214 | 1.96E-11 | 3.13E-10 | Bcar1 |
| 10509002 | -4.01475 | 7.734936 | 12.21313 | 1.96E-11 | 3.14E-10 | Rhd |
| 10441601 | -1.17448 | 5.584803 | 12.20245 | 2.00E-11 | 3.19E-10 | Tagap |
| 10358519 | 1.509435 | 5.366379 | -12.1931 | 2.03E-11 | 3.23E-10 | Hmcn1 |
| 10358527 | 2.127619 | 5.572446 | -12.1908 | 2.04E-11 | 3.24E-10 | Hmcn1 |
| 10462818 | -1.48029 | 6.512021 | 12.18887 | 2.04E-11 | 3.25E-10 | Hhex |
| 10371356 | 1.015104 | 7.335761 | -12.1868 | 2.05E-11 | 3.26E-10 | Appl2 |
| 10374727 | -2.28721 | 6.827577 | 12.18625 | 2.05E-11 | 3.26E-10 | Bcl11a |
| 10576051 | 1.309399 | 6.022861 | -12.1827 | 2.06E-11 | 3.28E-10 | Foxc2 |
| 10363130 | 1.092613 | 7.535658 | -12.1822 | 2.06E-11 | 3.28E-10 | Gm26741 |
| 10393866 | -1.17008 | 6.272954 | 12.18051 | 2.07E-11 | 3.28E-10 | Sirt7 |
| 10349947 | 2.379205 | 9.84519 | -12.1779 | 2.08E-11 | 3.29E-10 | Fmod |
| 10593856 | 1.023189 | 8.002148 | -12.1769 | 2.08E-11 | 3.30E-10 | Peak1 |
| 10538338 | 1.140214 | 6.311206 | -12.1695 | 2.11E-11 | 3.33E-10 | Creb5 |
| 10459518 | -1.22519 | 7.386149 | 12.16934 | 2.11E-11 | 3.33E-10 | Mppe1 |
| 10448559 | -1.32798 | 4.241786 | 12.16896 | 2.11E-11 | 3.33E-10 | D330041H03Rik |
| 10547056 | -1.93893 | 6.522735 | 12.16816 | 2.11E-11 | 3.34E-10 | Tmem40 |
| 10422946 | 1.587781 | 4.77078 | -12.1634 | 2.13E-11 | 3.36E-10 | Ranbp3l |
| 10445442 | -1.48144 | 6.857654 | 12.16301 | 2.13E-11 | 3.36E-10 | Gtpbp2 |
| 10583952 | -1.46189 | 7.389116 | 12.162 | 2.13E-11 | 3.37E-10 | Ncapd3 |
| 10554926 | 1.699817 | 7.718932 | -12.1587 | 2.15E-11 | 3.38E-10 | Ccdc90b |
| 10503448 | 2.182435 | 6.403989 | -12.1546 | 2.16E-11 | 3.40E-10 | Mmp16 |
| 10479811 | -1.18152 | 6.092715 | 12.15398 | 2.16E-11 | 3.40E-10 | Mcm10 |
| 10355742 | -1.12763 | 5.841694 | 12.15266 | 2.17E-11 | 3.41E-10 | Abcb6 |
| 10539669 | -1.96615 | 5.60233 | 12.14936 | 2.18E-11 | 3.42E-10 | Add2 |
| 10466216 | -1.23745 | 4.249662 | 12.1458 | 2.19E-11 | 3.44E-10 | Ms4a2 |
| 10501971 | 1.239712 | 5.972196 | -12.1443 | 2.20E-11 | 3.45E-10 | Ank2 |
| 10358615 | 1.68537 | 6.076506 | -12.1443 | 2.20E-11 | 3.45E-10 | Hmcn1 |
| 10359635 | 1.092558 | 5.92724 | -12.1416 | 2.21E-11 | 3.46E-10 | Gorab |
| 10607619 | 1.163354 | 4.294843 | -12.1413 | 2.21E-11 | 3.46E-10 | Cdkl5 |
| 10569848 | -1.58289 | 7.798183 | 12.14064 | 2.21E-11 | 3.46E-10 | Stxbp2 |
| 10555297 | 1.671433 | 5.881324 | -12.1405 | 2.21E-11 | 3.46E-10 | Kcne3 |
| 10517967 | 2.007128 | 6.530834 | -12.1402 | 2.21E-11 | 3.46E-10 | Fblim1 |
| 10469828 | -1.62245 | 6.602503 | 12.13847 | 2.22E-11 | 3.46E-10 | Psd4 |
| 10368289 | 1.818853 | 8.315881 | -12.1347 | 2.23E-11 | 3.48E-10 | Enpp1 |
| 10440344 | 1.393326 | 5.858718 | -12.127 | 2.26E-11 | 3.52E-10 | Robo2 |
| 10409502 | -1.42081 | 6.859735 | 12.12661 | 2.26E-11 | 3.52E-10 | Dok3 |
| 10489471 | 2.060144 | 5.487079 | -12.1258 | 2.26E-11 | 3.52E-10 | Matn4 |
| 10479154 | -2.68538 | 6.502419 | 12.12472 | 2.27E-11 | 3.53E-10 | Tubb1 |
| 10373542 | -1.19126 | 7.074596 | 12.1237 | 2.27E-11 | 3.53E-10 | Dgka |
| 10360920 | 1.85984 | 6.404537 | -12.1202 | 2.28E-11 | 3.55E-10 | Tgfb2 |
| 10397645 | -1.43215 | 7.187287 | 12.11756 | 2.29E-11 | 3.56E-10 | Gpr65 |
| 10520304 | 1.147501 | 5.639407 | -12.1103 | 2.32E-11 | 3.60E-10 | Actr3b |
| 10450682 | -1.15337 | 6.782277 | 12.10928 | 2.32E-11 | 3.61E-10 | C920025E04Rik |
| 10407211 | 1.177334 | 7.936248 | -12.105 | 2.34E-11 | 3.63E-10 | Ppap2a |
| 10384956 | -1.8394 | 7.123655 | 12.10222 | 2.35E-11 | 3.65E-10 | Chac2 |
| 10372917 | 1.5146 | 6.699737 | -12.0869 | 2.41E-11 | 3.73E-10 | Tmem5 |
| 10543306 | 1.244253 | 6.809039 | -12.0837 | 2.42E-11 | 3.75E-10 | Tspan12 |
| 10531203 | 1.454237 | 5.942556 | -12.0824 | 2.43E-11 | 3.75E-10 | Adamts3 |
| 10410124 | 1.240027 | 8.744861 | -12.0774 | 2.45E-11 | 3.78E-10 | Ctsl |
| 10562812 | -3.083 | 6.026039 | 12.07693 | 2.45E-11 | 3.78E-10 | Spib |
| 10565315 | 1.287417 | 5.93761 | -12.0745 | 2.46E-11 | 3.80E-10 | Fah |
| 10588091 | -1.35998 | 5.960833 | 12.07295 | 2.47E-11 | 3.80E-10 | Cep70 |
| 10360018 | -1.57665 | 4.90361 | 12.07239 | 2.47E-11 | 3.81E-10 | Fcrla |
| 10397633 | 1.698492 | 7.395813 | -12.0647 | 2.50E-11 | 3.85E-10 | Flrt2 |
| 10567863 | -2.39997 | 5.737113 | 12.05607 | 2.54E-11 | 3.90E-10 | Cd19 |
| 10512757 | -3.95256 | 7.61836 | 12.05585 | 2.54E-11 | 3.90E-10 | Hemgn |
| 10423894 | 1.705871 | 5.470775 | -12.0553 | 2.54E-11 | 3.90E-10 | Dcstamp |
| 10438405 | -2.88837 | 7.398889 | 12.05402 | 2.54E-11 | 3.91E-10 | Iglv1 |
| 10438891 | -2.02582 | 4.999962 | 12.04653 | 2.57E-11 | 3.95E-10 | Gm1968 |
| 10476252 | -1.88577 | 7.051254 | 12.04404 | 2.59E-11 | 3.97E-10 | Cdc25b |
| 10584941 | 1.150956 | 7.84707 | -12.035 | 2.62E-11 | 4.02E-10 | Bace1 |
| 10579799 | 1.161581 | 6.34042 | -12.0338 | 2.63E-11 | 4.03E-10 | Tmem184c |
| 10437210 | 1.014719 | 5.527458 | -12.0319 | 2.64E-11 | 4.04E-10 | Bace2 |
| 10516529 | 1.032321 | 5.384953 | -12.023 | 2.68E-11 | 4.10E-10 | Azin2 |
| 10555695 | -1.36762 | 8.695941 | 12.01715 | 2.70E-11 | 4.13E-10 | Rrm1 |
| 10430929 | -1.35048 | 6.843989 | 12.01587 | 2.71E-11 | 4.14E-10 | Tbrg3 |
| 10571274 | -1.44196 | 9.367496 | 12.01391 | 2.72E-11 | 4.15E-10 | Gsr |
| 10356278 | -1.84443 | 8.694264 | 12.01248 | 2.72E-11 | 4.16E-10 | Sp110 |
| 10422518 | 1.547858 | 5.717248 | -12.0063 | 2.75E-11 | 4.19E-10 | Tmtc4 |
| 10411739 | -1.50353 | 8.158597 | 12.00458 | 2.76E-11 | 4.20E-10 | Ccnb1 |
| 10525210 | -1.80442 | 7.661953 | 12.00455 | 2.76E-11 | 4.20E-10 | Gm15800 |
| 10413710 | 1.989442 | 7.163189 | -11.9946 | 2.80E-11 | 4.27E-10 | Nt5dc2 |
| 10526120 | 1.237595 | 7.766869 | -11.9904 | 2.82E-11 | 4.29E-10 | Tpst1 |
| 10361748 | -1.28905 | 8.344858 | 11.98282 | 2.86E-11 | 4.33E-10 | Fbxo30 |
| 10418848 | -1.6898 | 6.938474 | 11.98279 | 2.86E-11 | 4.33E-10 | Wdfy4 |
| 10390640 | -2.48608 | 4.949088 | 11.98104 | 2.87E-11 | 4.34E-10 | Ikzf3 |
| 10564713 | 2.11958 | 7.545111 | -11.9795 | 2.87E-11 | 4.35E-10 | Mfge8 |
| 10600301 | 1.215353 | 9.353095 | -11.9776 | 2.88E-11 | 4.36E-10 | Ssr4 |
| 10370544 | 1.339298 | 7.090309 | -11.9658 | 2.94E-11 | 4.44E-10 | 2610008E11Rik |
| 10523595 | 1.319303 | 6.578678 | -11.9637 | 2.95E-11 | 4.46E-10 | Ptpn13 |
| 10455098 | 1.228151 | 4.572371 | -11.9598 | 2.97E-11 | 4.48E-10 | Pcdhb14 |
| 10472538 | -1.80287 | 5.930709 | 11.95203 | 3.01E-11 | 4.53E-10 | Dhrs9 |
| 10429327 | -1.203 | 7.833503 | 11.95078 | 3.01E-11 | 4.54E-10 | Ago2 |
| 10492330 | -1.60766 | 6.156298 | 11.9494 | 3.02E-11 | 4.55E-10 | P2ry1 |
| 10358658 | 2.168392 | 5.99445 | -11.9462 | 3.04E-11 | 4.56E-10 | Hmcn1 |
| 10358664 | 2.344396 | 5.684835 | -11.9426 | 3.05E-11 | 4.59E-10 | Hmcn1 |
| 10601581 | -1.29198 | 7.41518 | 11.93973 | 3.07E-11 | 4.60E-10 | Gm4992 |
| 10470948 | 1.293148 | 6.166132 | -11.9355 | 3.09E-11 | 4.63E-10 | Slc39a1 |
| 10360120 | 1.208394 | 8.670421 | -11.9328 | 3.10E-11 | 4.64E-10 | Ufc1 |
| 10408616 | -1.53001 | 6.29326 | 11.92663 | 3.13E-11 | 4.69E-10 | Slc22a23 |
| 10434191 | -1.49497 | 6.09672 | 11.92238 | 3.16E-11 | 4.72E-10 | Txnrd2 |
| 10393408 | -1.0028 | 6.085547 | 11.92065 | 3.17E-11 | 4.73E-10 | Tmc6 |
| 10587495 | 1.342383 | 6.122167 | -11.9148 | 3.20E-11 | 4.77E-10 | Irak1bp1 |
| 10547657 | 1.695371 | 6.965825 | -11.9132 | 3.20E-11 | 4.78E-10 | C3ar1 |
| 10368670 | -1.13752 | 8.539884 | 11.91192 | 3.21E-11 | 4.78E-10 | Amd2 |
| 10361509 | -1.09052 | 8.183523 | 11.9091 | 3.23E-11 | 4.80E-10 | Syne1 |
| 10403028 | -2.842 | 5.492968 | 11.90033 | 3.27E-11 | 4.86E-10 | Ighv1-52 |
| 10585282 | -1.12606 | 5.962679 | 11.89889 | 3.28E-11 | 4.87E-10 | LOC102635638 |
| 10498018 | 2.01764 | 7.393013 | -11.8971 | 3.29E-11 | 4.89E-10 | Pcdh18 |
| 10492558 | -1.25896 | 9.26684 | 11.89533 | 3.30E-11 | 4.89E-10 | Smc4 |
| 10393364 | 1.277053 | 7.867363 | -11.8944 | 3.31E-11 | 4.90E-10 | Cygb |
| 10438293 | 1.750239 | 6.4724 | -11.8812 | 3.38E-11 | 5.00E-10 | Zdhhc8 |
| 10551587 | -1.30469 | 5.883016 | 11.87377 | 3.42E-11 | 5.05E-10 | Rinl |
| 10392464 | 1.408114 | 6.139283 | -11.8736 | 3.42E-11 | 5.05E-10 | Fam20a |
| 10428103 | 1.003807 | 7.451663 | -11.8736 | 3.42E-11 | 5.05E-10 | Stk3 |
| 10508074 | -2.32912 | 7.686303 | 11.87312 | 3.42E-11 | 5.05E-10 | Csf3r |
| 10392739 | 1.992696 | 5.975997 | -11.8728 | 3.43E-11 | 5.05E-10 | Sdk2 |
| 10506822 | -1.76878 | 5.467692 | 11.86696 | 3.46E-11 | 5.10E-10 | Orc1 |
| 10582501 | -1.07133 | 6.149196 | 11.86471 | 3.47E-11 | 5.11E-10 | Fanca |
| 10569011 | 2.686888 | 7.983544 | -11.8588 | 3.51E-11 | 5.16E-10 | Ifitm5 |
| 10358633 | 2.418266 | 6.0019 | -11.8534 | 3.54E-11 | 5.21E-10 | Hmcn1 |
| 10405343 | 1.064354 | 6.430836 | -11.8529 | 3.54E-11 | 5.21E-10 | Tspan17 |
| 10588509 | 1.247366 | 6.049887 | -11.85 | 3.56E-11 | 5.23E-10 | Pcbp4 |
| 10559454 | -1.48923 | 9.041668 | 11.84965 | 3.56E-11 | 5.23E-10 | Gm10693 |
| 10356248 | -1.95084 | 6.312973 | 11.84808 | 3.57E-11 | 5.24E-10 | C130026I21Rik |
| 10538640 | -1.17951 | 7.52712 | 11.84079 | 3.61E-11 | 5.30E-10 | Abcg2 |
| 10566333 | -1.35903 | 7.689266 | 11.84031 | 3.61E-11 | 5.30E-10 | Trim5 |
| 10534456 | 1.073598 | 6.670192 | -11.8399 | 3.62E-11 | 5.30E-10 | Hip1 |
| 10370766 | 1.10317 | 6.851576 | -11.8382 | 3.63E-11 | 5.31E-10 | Gamt |
| 10538880 | -3.91665 | 5.553895 | 11.83674 | 3.64E-11 | 5.32E-10 | Igkv1-117 |
| 10440953 | -1.3157 | 6.15156 | 11.83623 | 3.64E-11 | 5.32E-10 | Donson |
| 10539766 | 1.094957 | 7.119235 | -11.8357 | 3.64E-11 | 5.32E-10 | Aak1 |
| 10377560 | 1.669777 | 5.610718 | -11.8338 | 3.65E-11 | 5.34E-10 | Sat2 |
| 10358517 | 1.756069 | 5.290825 | -11.8333 | 3.66E-11 | 5.34E-10 | Hmcn1 |
| 10496519 | 1.118865 | 5.051453 | -11.831 | 3.67E-11 | 5.36E-10 | Unc5c |
| 10358525 | 1.848897 | 4.126039 | -11.8296 | 3.68E-11 | 5.37E-10 | Hmcn1 |
| 10404024 | -1.69571 | 8.390618 | 11.82645 | 3.70E-11 | 5.39E-10 | Hist1h4h |
| 10401527 | 1.263504 | 6.598114 | -11.8249 | 3.71E-11 | 5.40E-10 | Ltbp2 |
| 10526943 | -1.16025 | 6.615317 | 11.81794 | 3.75E-11 | 5.45E-10 | Gpr146 |
| 10389858 | 1.042347 | 8.850879 | -11.8177 | 3.75E-11 | 5.45E-10 | Nme2 |
| 10514054 | 1.40719 | 8.710151 | -11.8175 | 3.75E-11 | 5.45E-10 | Nfib |
| 10544002 | 1.596327 | 6.62843 | -11.8146 | 3.77E-11 | 5.47E-10 | Creb3l2 |
| 10606600 | 1.421369 | 5.18275 | -11.814 | 3.78E-11 | 5.47E-10 | Pcdh19 |
| 10521927 | 1.425133 | 6.487954 | -11.8133 | 3.78E-11 | 5.48E-10 | Tbc1d19 |
| 10356262 | -1.99499 | 6.669516 | 11.81049 | 3.80E-11 | 5.50E-10 | Gm7609 |
| 10429856 | -2.77611 | 7.172531 | 11.81011 | 3.80E-11 | 5.50E-10 | Gm10872 |
| 10520234 | 1.267536 | 6.444377 | -11.8082 | 3.81E-11 | 5.51E-10 | Chpf2 |
| 10535807 | 1.459074 | 6.644431 | -11.8069 | 3.82E-11 | 5.52E-10 | Flt1 |
| 10604576 | 1.894032 | 6.597699 | -11.8042 | 3.84E-11 | 5.54E-10 | Gpc3 |
| 10479041 | -2.82787 | 8.331659 | 11.79466 | 3.90E-11 | 5.62E-10 | Rbm38 |
| 10443009 | 1.693718 | 8.705069 | -11.7845 | 3.96E-11 | 5.71E-10 | Ergic1 |
| 10511617 | 2.020124 | 7.143018 | -11.7809 | 3.99E-11 | 5.74E-10 | Fam92a |
| 10460738 | -1.37551 | 6.841836 | 11.77994 | 3.99E-11 | 5.75E-10 | Cdca5 |
| 10490826 | 1.235108 | 6.27538 | -11.7777 | 4.01E-11 | 5.77E-10 | Zbtb10 |
| 10350149 | 2.385086 | 6.397148 | -11.7727 | 4.04E-11 | 5.81E-10 | Tnni1 |
| 10552760 | -1.0845 | 6.682071 | 11.77171 | 4.05E-11 | 5.82E-10 | Pnkp |
| 10545458 | 1.162817 | 6.259592 | -11.7711 | 4.05E-11 | 5.82E-10 | Tcf7l1 |
| 10485674 | 1.022489 | 7.328203 | -11.7686 | 4.07E-11 | 5.84E-10 | Arl14ep |
| 10406968 | -1.92158 | 7.057683 | 11.76603 | 4.09E-11 | 5.86E-10 | Cenpk |
| 10565727 | 1.178323 | 6.028286 | -11.7649 | 4.10E-11 | 5.87E-10 | Tsku |
| 10425053 | -1.94482 | 7.130993 | 11.76302 | 4.11E-11 | 5.89E-10 | Ncf4 |
| 10524052 | 1.021032 | 6.815854 | -11.7624 | 4.11E-11 | 5.89E-10 | Fgfrl1 |
| 10584435 | 1.144675 | 7.596571 | -11.7609 | 4.12E-11 | 5.90E-10 | Vwa5a |
| 10429573 | -1.21652 | 9.926497 | 11.75894 | 4.14E-11 | 5.91E-10 | Ly6c2 |
| 10368918 | 1.231084 | 6.079618 | -11.7587 | 4.14E-11 | 5.91E-10 | Sobp |
| 10410408 | 1.19784 | 6.548792 | -11.7575 | 4.15E-11 | 5.92E-10 | Adcy2 |
| 10388996 | -1.05951 | 7.576661 | 11.75125 | 4.19E-11 | 5.97E-10 | Crlf3 |
| 10458555 | 1.073784 | 5.777607 | -11.7498 | 4.20E-11 | 5.99E-10 | Spry4 |
| 10440002 | -1.36439 | 4.522801 | 11.74795 | 4.21E-11 | 6.00E-10 | Gpr128 |
| 10542181 | -2.19282 | 5.539003 | 11.7473 | 4.22E-11 | 6.00E-10 | Clec9a |
| 10494407 | -1.92379 | 7.762148 | 11.74104 | 4.26E-11 | 6.06E-10 | Hist2h2bb |
| 10430968 | 1.264433 | 5.524295 | -11.7401 | 4.27E-11 | 6.07E-10 | A4galt |
| 10545921 | -1.69373 | 7.814772 | 11.73714 | 4.29E-11 | 6.09E-10 | Mxd1 |
| 10446027 | -1.2203 | 6.737698 | 11.7345 | 4.31E-11 | 6.12E-10 | Chaf1a |
| 10377924 | -2.95464 | 6.868604 | 11.73411 | 4.31E-11 | 6.12E-10 | Gp1ba |
| 10362186 | 2.821105 | 5.713846 | -11.7307 | 4.34E-11 | 6.15E-10 | Moxd1 |
| 10476104 | -1.46511 | 4.978851 | 11.72967 | 4.34E-11 | 6.15E-10 | Gm23650 |
| 10603431 | -1.20752 | 6.573334 | 11.72072 | 4.41E-11 | 6.24E-10 | Suv39h1 |
| 10382425 | 1.689638 | 6.940229 | -11.7091 | 4.49E-11 | 6.36E-10 | Gprc5c |
| 10586477 | 1.412977 | 9.812039 | -11.7054 | 4.52E-11 | 6.39E-10 | Ppib |
| 10383556 | -1.68481 | 6.753418 | 11.70386 | 4.53E-11 | 6.41E-10 | Fn3krp |
| 10521731 | -1.70171 | 7.498954 | 11.7034 | 4.54E-11 | 6.41E-10 | Ncapg |
| 10403060 | -2.90281 | 8.04144 | 11.69883 | 4.57E-11 | 6.45E-10 | Igh-VJ558 |
| 10538871 | -2.97786 | 6.090426 | 11.69705 | 4.59E-11 | 6.47E-10 | Igkv1-135 |
| 10408074 | -1.07996 | 11.80254 | 11.69469 | 4.60E-11 | 6.49E-10 | Hist1h4m |
| 10385118 | -1.83249 | 7.497827 | 11.69271 | 4.62E-11 | 6.51E-10 | Dock2 |
| 10415392 | -1.71591 | 6.995085 | 11.68967 | 4.64E-11 | 6.54E-10 | Ltb4r1 |
| 10489246 | 1.487989 | 7.334974 | -11.6884 | 4.65E-11 | 6.55E-10 | Mafb |
| 10603289 | 1.222241 | 6.187391 | -11.6874 | 4.66E-11 | 6.55E-10 | Clcn5 |
| 10415885 | 1.133367 | 5.207199 | -11.6748 | 4.76E-11 | 6.68E-10 | Sox7 |
| 10546113 | 1.437283 | 8.997748 | -11.6731 | 4.77E-11 | 6.69E-10 | Sec61a1 |
| 10361882 | 1.475568 | 6.445708 | -11.669 | 4.81E-11 | 6.74E-10 | Nhsl1 |
| 10348817 | 1.197354 | 9.003162 | -11.6643 | 4.84E-11 | 6.78E-10 | 44441 |
| 10603151 | 1.423236 | 6.873035 | -11.6614 | 4.87E-11 | 6.81E-10 | Gpm6b |
| 10445688 | -1.43265 | 8.165279 | 11.65667 | 4.91E-11 | 6.85E-10 | Ccnd3 |
| 10362097 | -1.74644 | 6.49578 | 11.64842 | 4.97E-11 | 6.94E-10 | H60b |
| 10373902 | 1.252685 | 5.56612 | -11.6474 | 4.98E-11 | 6.95E-10 | Gatsl3 |
| 10544106 | -1.1335 | 4.777462 | 11.64673 | 4.99E-11 | 6.95E-10 | Gm15697 |
| 10399178 | -1.20119 | 6.14743 | 11.64462 | 5.01E-11 | 6.98E-10 | Cdca7l |
| 10450412 | -2.65729 | 6.436596 | 11.64232 | 5.03E-11 | 7.00E-10 | G6b |
| 10382435 | 1.949644 | 7.146081 | -11.638 | 5.06E-11 | 7.05E-10 | Gprc5c |
| 10497994 | 1.118374 | 4.280934 | -11.6377 | 5.06E-11 | 7.05E-10 | Pabpc4l |
| 10574572 | -3.1964 | 6.832271 | 11.63538 | 5.08E-11 | 7.06E-10 | Ces2g |
| 10477854 | 1.089294 | 6.659329 | -11.6341 | 5.10E-11 | 7.08E-10 | Epb4.1l1 |
| 10478415 | 1.104088 | 6.980225 | -11.633 | 5.10E-11 | 7.09E-10 | Wisp2 |
| 10385893 | -1.44794 | 6.210218 | 11.6307 | 5.12E-11 | 7.11E-10 | Slc22a4 |
| 10602401 | 1.22147 | 6.248751 | -11.6302 | 5.13E-11 | 7.11E-10 | Fgd1 |
| 10424779 | -1.28427 | 8.424464 | 11.62374 | 5.18E-11 | 7.19E-10 | Cks2 |
| 10603796 | 1.450856 | 4.847589 | -11.6182 | 5.23E-11 | 7.25E-10 | 4930578C19Rik |
| 10589420 | -1.06227 | 6.997496 | 11.61735 | 5.24E-11 | 7.26E-10 | Cdc25a |
| 10392221 | 1.145437 | 8.207944 | -11.6136 | 5.27E-11 | 7.30E-10 | Pecam1 |
| 10375175 | 1.765438 | 7.687121 | -11.6131 | 5.28E-11 | 7.30E-10 | Slit3 |
| 10523701 | 2.072134 | 10.86664 | -11.6122 | 5.29E-11 | 7.31E-10 | Ibsp |
| 10570556 | -1.05935 | 6.236695 | 11.60497 | 5.35E-11 | 7.39E-10 | Mcph1 |
| 10539739 | -1.55866 | 6.406081 | 11.59935 | 5.40E-11 | 7.46E-10 | Asprv1 |
| 10570278 | -1.09448 | 5.376864 | 11.59843 | 5.41E-11 | 7.47E-10 | Gm15352 |
| 10382956 | -1.97738 | 5.901704 | 11.59171 | 5.47E-11 | 7.55E-10 | Tmc8 |
| 10463224 | 1.191262 | 8.723384 | -11.5847 | 5.54E-11 | 7.63E-10 | Marveld1 |
| 10546434 | 1.539356 | 6.314636 | -11.5803 | 5.58E-11 | 7.68E-10 | Adamts9 |
| 10508533 | 1.102899 | 6.492112 | -11.5781 | 5.60E-11 | 7.70E-10 | Col16a1 |
| 10365749 | -1.37142 | 7.958583 | 11.57706 | 5.61E-11 | 7.71E-10 | Lta4h |
| 10474545 | -1.11563 | 7.632294 | 11.5761 | 5.62E-11 | 7.72E-10 | Slc12a6 |
| 10361169 | 1.067455 | 4.787407 | -11.5758 | 5.62E-11 | 7.72E-10 | Hhat |
| 10347792 | 2.532543 | 7.070377 | -11.5734 | 5.64E-11 | 7.75E-10 | Gm9747 |
| 10494390 | -1.0104 | 11.00363 | 11.55686 | 5.80E-11 | 7.95E-10 | Hist2h2aa1 |
| 10427166 | -1.39691 | 5.863609 | 11.55304 | 5.84E-11 | 8.00E-10 | Espl1 |
| 10531126 | -3.93097 | 7.181032 | 11.55102 | 5.86E-11 | 8.01E-10 | Igj |
| 10358654 | 2.04114 | 6.814091 | -11.551 | 5.86E-11 | 8.01E-10 | Hmcn1 |
| 10390299 | -1.81792 | 6.514101 | 11.55087 | 5.86E-11 | 8.01E-10 | Pnpo |
| 10550102 | -1.39925 | 7.13635 | 11.5478 | 5.89E-11 | 8.05E-10 | Lig1 |
| 10442396 | -1.06947 | 6.55527 | 11.54319 | 5.94E-11 | 8.11E-10 | Abca3 |
| 10404067 | -1.02902 | 11.84814 | 11.54308 | 5.94E-11 | 8.11E-10 | Hist1h4h |
| 10380174 | -2.86219 | 9.759662 | 11.54116 | 5.96E-11 | 8.13E-10 | Mpo |
| 10449266 | 1.298864 | 6.507691 | -11.5297 | 6.07E-11 | 8.26E-10 | Itfg3 |
| 10483401 | -1.49413 | 6.75002 | 11.52875 | 6.08E-11 | 8.27E-10 | Spc25 |
| 10368748 | -1.1242 | 8.492571 | 11.52518 | 6.12E-11 | 8.32E-10 | Amd2 |
| 10403193 | -1.09083 | 6.285606 | 11.5136 | 6.24E-11 | 8.48E-10 | Sp4 |
| 10368092 | 1.026554 | 5.537036 | -11.5125 | 6.25E-11 | 8.49E-10 | Hebp2 |
| 10462140 | -1.74233 | 8.233406 | 11.51185 | 6.26E-11 | 8.50E-10 | Dock8 |
| 10430195 | -1.75608 | 5.987602 | 11.51052 | 6.27E-11 | 8.51E-10 | Apol8 |
| 10368577 | 1.528518 | 6.883619 | -11.5102 | 6.28E-11 | 8.51E-10 | Rnf217 |
| 10541301 | -1.4168 | 7.844032 | 11.50948 | 6.28E-11 | 8.52E-10 | Tuba8 |
| 10583044 | 2.111494 | 10.19431 | -11.5071 | 6.31E-11 | 8.54E-10 | Mmp13 |
| 10423498 | 1.737919 | 9.723331 | -11.5056 | 6.33E-11 | 8.56E-10 | Dap |
| 10369541 | -1.02664 | 7.275838 | 11.49838 | 6.40E-11 | 8.65E-10 | Hk1 |
| 10426315 | -1.15634 | 7.193086 | 11.49231 | 6.47E-11 | 8.73E-10 | Lrrk2 |
| 10358531 | 2.01204 | 5.311103 | -11.4917 | 6.48E-11 | 8.73E-10 | Hmcn1 |
| 10436666 | 1.645096 | 7.376923 | -11.4906 | 6.49E-11 | 8.74E-10 | Jam2 |
| 10429520 | -2.53882 | 6.857183 | 11.48943 | 6.50E-11 | 8.76E-10 | Ly6d |
| 10598771 | 1.383881 | 5.743879 | -11.4873 | 6.52E-11 | 8.78E-10 | Maoa |
| 10505931 | 1.813263 | 5.796569 | -11.4872 | 6.53E-11 | 8.78E-10 | Ift74 |
| 10396952 | 1.384938 | 4.903526 | -11.4799 | 6.61E-11 | 8.87E-10 | Ttc9 |
| 10480329 | 1.053327 | 6.867859 | -11.4798 | 6.61E-11 | 8.87E-10 | Dnajc1 |
| 10553967 | 1.842 | 6.937458 | -11.4795 | 6.61E-11 | 8.87E-10 | Pcsk6 |
| 10466794 | -1.03288 | 6.164616 | 11.47816 | 6.63E-11 | 8.89E-10 | Fam122a |
| 10553559 | -1.96165 | 5.747555 | 11.47 | 6.72E-11 | 9.00E-10 | Siglech |
| 10447341 | 1.224316 | 7.220376 | -11.4698 | 6.72E-11 | 9.00E-10 | Rhoq |
| 10432294 | -1.02107 | 7.814091 | 11.46246 | 6.80E-11 | 9.11E-10 | Kmt2d |
| 10351047 | -1.5308 | 5.942898 | 11.45675 | 6.87E-11 | 9.19E-10 | Cenpl |
| 10531197 | 1.536539 | 5.815083 | -11.4513 | 6.93E-11 | 9.27E-10 | Adamts3 |
| 10402705 | 1.40168 | 5.939531 | -11.4452 | 7.01E-11 | 9.36E-10 | Gm266 |
| 10482030 | -1.90557 | 8.449237 | 11.44396 | 7.02E-11 | 9.38E-10 | Stom |
| 10403048 | -3.16357 | 8.551726 | 11.43881 | 7.08E-11 | 9.45E-10 | Ighv1-62-3 |
| 10430319 | -1.45692 | 6.923742 | 11.431 | 7.18E-11 | 9.56E-10 | Tst |
| 10545202 | -1.89274 | 5.88529 | 11.431 | 7.18E-11 | 9.56E-10 | LOC102642862 |
| 10411235 | -1.58962 | 7.218197 | 11.42703 | 7.23E-11 | 9.61E-10 | Iqgap2 |
| 10573483 | -1.19463 | 9.336584 | 11.42489 | 7.25E-11 | 9.64E-10 | Prdx2 |
| 10411306 | 1.32534 | 6.953859 | -11.4236 | 7.27E-11 | 9.66E-10 | Polk |
| 10472757 | 1.806626 | 6.284366 | -11.4235 | 7.27E-11 | 9.66E-10 | Cybrd1 |
| 10494386 | -1.17018 | 10.96532 | 11.41695 | 7.35E-11 | 9.76E-10 | Hist2h2ab |
| 10490775 | 1.203271 | 5.151718 | -11.416 | 7.36E-11 | 9.77E-10 | Gm10748 |
| 10348653 | 1.990222 | 8.000746 | -11.4113 | 7.42E-11 | 9.84E-10 | Gpc1 |
| 10414548 | -2.21379 | 6.737169 | 11.40954 | 7.44E-11 | 9.87E-10 | Rnase6 |
| 10573457 | -2.47827 | 6.618372 | 11.4081 | 7.46E-11 | 9.89E-10 | Klf1 |
| 10394783 | 1.24535 | 5.896941 | -11.405 | 7.50E-11 | 9.93E-10 | Gm9222 |
| 10394786 | 1.24535 | 5.896941 | -11.405 | 7.50E-11 | 9.93E-10 | Gm9222 |
| 10394498 | 1.463507 | 5.213693 | -11.4044 | 7.51E-11 | 9.93E-10 | Wdr35 |
| 10395252 | -1.19491 | 6.73348 | 11.40416 | 7.51E-11 | 9.93E-10 | Ccdc71l |
| 10405811 | 1.053554 | 6.331397 | -11.4038 | 7.52E-11 | 9.94E-10 | Habp4 |
| 10590494 | -1.58456 | 7.391723 | 11.39546 | 7.62E-11 | 1.01E-09 | Kif15 |
| 10478355 | -1.58885 | 7.010194 | 11.39127 | 7.68E-11 | 1.01E-09 | Mybl2 |
| 10474045 | 1.210891 | 7.08392 | -11.3892 | 7.71E-11 | 1.02E-09 | Chst1 |
| 10423654 | 1.763165 | 5.669429 | -11.3841 | 7.77E-11 | 1.02E-09 | Osr2 |
| 10443764 | -1.15508 | 5.937141 | 11.38309 | 7.79E-11 | 1.03E-09 | Slc37a1 |
| 10440993 | 1.621309 | 7.867171 | -11.3723 | 7.93E-11 | 1.04E-09 | Rcan1 |
| 10565775 | -1.00724 | 6.45414 | 11.37089 | 7.95E-11 | 1.05E-09 | Dgat2 |
| 10445796 | -1.03047 | 6.835705 | 11.37066 | 7.95E-11 | 1.05E-09 | Oard1 |
| 10566943 | -1.54085 | 6.26281 | 11.37065 | 7.95E-11 | 1.05E-09 | Mrvi1 |
| 10465282 | -1.26731 | 6.467062 | 11.36613 | 8.02E-11 | 1.05E-09 | Pola2 |
| 10447130 | 1.300696 | 5.746198 | -11.3588 | 8.12E-11 | 1.06E-09 | Pkdcc |
| 10382985 | -1.43237 | 6.260836 | 11.35846 | 8.12E-11 | 1.07E-09 | Afmid |
| 10599200 | 1.386468 | 7.355097 | -11.3565 | 8.15E-11 | 1.07E-09 | Pgrmc1 |
| 10480956 | -1.14014 | 6.182558 | 11.35624 | 8.15E-11 | 1.07E-09 | Card9 |
| 10481175 | 1.815248 | 6.375399 | -11.3454 | 8.30E-11 | 1.09E-09 | Tmem8c |
| 10458983 | -1.32271 | 6.731815 | 11.34468 | 8.31E-11 | 1.09E-09 | 44258 |
| 10587616 | 3.107548 | 6.625259 | -11.3329 | 8.48E-11 | 1.11E-09 | Prss35 |
| 10466530 | 1.18681 | 6.109685 | -11.3327 | 8.49E-11 | 1.11E-09 | Pcsk5 |
| 10446553 | 1.606776 | 6.325796 | -11.3326 | 8.49E-11 | 1.11E-09 | Epb4.1l3 |
| 10485388 | 1.517168 | 7.58319 | -11.3318 | 8.50E-11 | 1.11E-09 | Ldlrad3 |
| 10538901 | -1.26923 | 4.554058 | 11.32976 | 8.53E-11 | 1.11E-09 | BC005685 |
| 10504127 | 1.21313 | 9.076016 | -11.3261 | 8.58E-11 | 1.12E-09 | Gm13304 |
| 10504154 | 1.21313 | 9.076016 | -11.3261 | 8.58E-11 | 1.12E-09 | Gm13304 |
| 10504183 | 1.21313 | 9.076016 | -11.3261 | 8.58E-11 | 1.12E-09 | Gm13304 |
| 10512377 | 1.21313 | 9.076016 | -11.3261 | 8.58E-11 | 1.12E-09 | Gm13304 |
| 10358717 | 1.253801 | 8.683897 | -11.3256 | 8.59E-11 | 1.12E-09 | 1700025G04Rik |
| 10375083 | -1.41511 | 6.816351 | 11.3233 | 8.62E-11 | 1.12E-09 | Stk10 |
| 10571567 | 1.481542 | 7.225764 | -11.3224 | 8.64E-11 | 1.12E-09 | Sorbs2 |
| 10546031 | -1.05332 | 6.018507 | 11.31878 | 8.69E-11 | 1.13E-09 | Aplf |
| 10557591 | -2.10723 | 6.412972 | 11.31628 | 8.73E-11 | 1.13E-09 | Itgal |
| 10581479 | 2.757918 | 7.62339 | -11.3162 | 8.73E-11 | 1.13E-09 | Smpd3 |
| 10398885 | 1.082098 | 5.863575 | -11.316 | 8.73E-11 | 1.13E-09 | Cep170b |
| 10503178 | -1.10697 | 6.095739 | 11.31415 | 8.76E-11 | 1.13E-09 | Chd7 |
| 10595371 | 1.229159 | 7.037115 | -11.313 | 8.78E-11 | 1.14E-09 | Hmgn3 |
| 10495763 | -1.94895 | 8.137244 | 11.31206 | 8.79E-11 | 1.14E-09 | Gclm |
| 10493995 | 1.36546 | 10.60428 | -11.3071 | 8.86E-11 | 1.15E-09 | S100a10 |
| 10412123 | -1.50837 | 7.50463 | 11.30293 | 8.93E-11 | 1.15E-09 | Ncf2 |
| 10554281 | -1.09413 | 5.510789 | 11.29726 | 9.02E-11 | 1.16E-09 | Fanci |
| 10526564 | -1.96138 | 7.026545 | 11.29203 | 9.10E-11 | 1.17E-09 | Ufsp1 |
| 10347910 | 1.000466 | 6.863998 | -11.2878 | 9.16E-11 | 1.18E-09 | Fbxo36 |
| 10419154 | -3.10619 | 8.153235 | 11.28495 | 9.21E-11 | 1.19E-09 | Ear1 |
| 10350840 | 2.049731 | 7.504453 | -11.2811 | 9.27E-11 | 1.19E-09 | Angptl1 |
| 10351293 | 3.002456 | 8.731303 | -11.2764 | 9.34E-11 | 1.20E-09 | Dpt |
| 10586184 | -1.00391 | 7.863294 | 11.27225 | 9.41E-11 | 1.21E-09 | Tipin |
| 10569618 | 1.598542 | 6.064873 | -11.271 | 9.43E-11 | 1.21E-09 | Ano1 |
| 10487447 | 1.412376 | 6.401623 | -11.2641 | 9.54E-11 | 1.23E-09 | Mall |
| 10581800 | -1.12924 | 7.408591 | 11.26358 | 9.55E-11 | 1.23E-09 | Rfwd3 |
| 10353004 | -1.41978 | 9.03011 | 11.26139 | 9.59E-11 | 1.23E-09 | Cks2 |
| 10495574 | -1.309 | 6.981537 | 11.25523 | 9.69E-11 | 1.24E-09 | Sass6 |
| 10600825 | 1.1479 | 4.990032 | -11.2541 | 9.71E-11 | 1.24E-09 | Zc3h12b |
| 10583573 | -1.14986 | 5.793454 | 11.2536 | 9.72E-11 | 1.24E-09 | Atg4d |
| 10372069 | 1.198943 | 6.281524 | -11.2528 | 9.73E-11 | 1.25E-09 | Socs2 |
| 10470014 | 1.165615 | 6.289491 | -11.2513 | 9.75E-11 | 1.25E-09 | Entpd2 |
| 10485225 | 1.337861 | 7.636983 | -11.2489 | 9.80E-11 | 1.25E-09 | Ext2 |
| 10534909 | -1.86678 | 7.769979 | 11.24886 | 9.80E-11 | 1.25E-09 | Gm15753 |
| 10554808 | 1.323892 | 6.187332 | -11.2462 | 9.84E-11 | 1.26E-09 | Fzd4 |
| 10379363 | -1.55729 | 6.378466 | 11.23975 | 9.95E-11 | 1.27E-09 | Atad5 |
| 10565456 | 1.505036 | 6.24998 | -11.234 | 1.00E-10 | 1.28E-09 | Prss23 |
| 10546010 | -1.70857 | 6.696051 | 11.23169 | 1.01E-10 | 1.29E-09 | Arhgap25 |
| 10546430 | 1.54684 | 6.068145 | -11.2286 | 1.01E-10 | 1.29E-09 | Adamts9 |
| 10463355 | 2.252308 | 7.403992 | -11.2225 | 1.03E-10 | 1.31E-09 | Scd2 |
| 10358559 | 1.700599 | 6.217113 | -11.2165 | 1.04E-10 | 1.32E-09 | Hmcn1 |
| 10544273 | -2.11481 | 6.659698 | 11.21647 | 1.04E-10 | 1.32E-09 | Clec5a |
| 10469457 | 1.713509 | 8.397769 | -11.2144 | 1.04E-10 | 1.32E-09 | Plxdc2 |
| 10605338 | -1.35059 | 8.412867 | 11.21405 | 1.04E-10 | 1.32E-09 | G6pdx |
| 10383867 | -1.06109 | 8.457256 | 11.21291 | 1.04E-10 | 1.32E-09 | Mtmr3 |
| 10601846 | 1.826598 | 6.158084 | -11.2117 | 1.04E-10 | 1.33E-09 | Arxes2 |
| 10464370 | -1.21351 | 4.927421 | 11.20981 | 1.05E-10 | 1.33E-09 | Slc18a2 |
| 10459389 | -1.10378 | 8.367946 | 11.20812 | 1.05E-10 | 1.33E-09 | Amd2 |
| 10487577 | -1.30948 | 6.148069 | 11.20287 | 1.06E-10 | 1.34E-09 | Ckap2l |
| 10599654 | 1.226982 | 7.047946 | -11.2018 | 1.06E-10 | 1.34E-09 | Cxx1c |
| 10350668 | 1.117863 | 4.50602 | -11.1921 | 1.08E-10 | 1.37E-09 | Colgalt2 |
| 10534041 | -1.00508 | 7.57651 | 11.19021 | 1.08E-10 | 1.37E-09 | Stx2 |
| 10459576 | -1.39265 | 7.100678 | 11.18986 | 1.08E-10 | 1.37E-09 | Cep76 |
| 10367746 | 1.153206 | 7.733407 | -11.1866 | 1.09E-10 | 1.38E-09 | Sash1 |
| 10464704 | -1.36283 | 8.093918 | 11.18042 | 1.10E-10 | 1.39E-09 | Adrbk1 |
| 10487588 | -2.26364 | 4.480567 | 11.17852 | 1.11E-10 | 1.39E-09 | Il1a |
| 10432439 | 1.093257 | 6.809557 | -11.1782 | 1.11E-10 | 1.39E-09 | Fmnl3 |
| 10403558 | -1.09595 | 5.640491 | 11.17807 | 1.11E-10 | 1.39E-09 | Ero1lb |
| 10523134 | -1.69835 | 10.50683 | 11.17562 | 1.11E-10 | 1.40E-09 | Pf4 |
| 10451547 | -1.05461 | 7.762515 | 11.17541 | 1.11E-10 | 1.40E-09 | Gm16494 |
| 10542120 | -1.9865 | 5.907617 | 11.17262 | 1.12E-10 | 1.40E-09 | Clec2i |
| 10501020 | -3.05219 | 9.964056 | 11.16918 | 1.12E-10 | 1.41E-09 | Chil3 |
| 10415045 | 1.085011 | 8.973698 | -11.1687 | 1.12E-10 | 1.41E-09 | Mrpl52 |
| 10459084 | 1.266556 | 6.133741 | -11.1676 | 1.13E-10 | 1.41E-09 | Synpo |
| 10471882 | 1.86581 | 6.179144 | -11.1592 | 1.14E-10 | 1.43E-09 | Olfml2a |
| 10458293 | 1.227121 | 6.515471 | -11.1565 | 1.15E-10 | 1.44E-09 | Dnajc18 |
| 10544588 | -2.53478 | 4.783584 | 11.15481 | 1.15E-10 | 1.44E-09 | Gimap3 |
| 10492078 | 1.346688 | 7.799965 | -11.1536 | 1.15E-10 | 1.44E-09 | Alg5 |
| 10530563 | -1.27866 | 7.875418 | 11.15298 | 1.16E-10 | 1.45E-09 | Fryl |
| 10347968 | -1.36329 | 4.817386 | 11.15083 | 1.16E-10 | 1.45E-09 | n-R5s215 |
| 10571371 | 1.079817 | 7.18374 | -11.1508 | 1.16E-10 | 1.45E-09 | Tusc3 |
| 10542140 | -1.99898 | 5.487427 | 11.14989 | 1.16E-10 | 1.45E-09 | Klrb1f |
| 10545173 | -3.22706 | 6.289656 | 11.14592 | 1.17E-10 | 1.46E-09 | Igkv10-96 |
| 10364262 | -1.63862 | 8.73995 | 11.14519 | 1.17E-10 | 1.46E-09 | Itgb2 |
| 10416126 | 1.407492 | 5.594555 | -11.144 | 1.17E-10 | 1.46E-09 | Ebf2 |
| 10466779 | -2.20603 | 6.850134 | 11.13684 | 1.19E-10 | 1.48E-09 | Pip5k1b |
| 10524284 | 1.170867 | 5.649402 | -11.1306 | 1.20E-10 | 1.49E-09 | Ttc28 |
| 10505276 | 1.136332 | 7.486502 | -11.1299 | 1.20E-10 | 1.49E-09 | Slc31a1 |
| 10387797 | 1.318446 | 5.576531 | -11.1164 | 1.23E-10 | 1.53E-09 | Bcl6b |
| 10512236 | -1.29997 | 5.765806 | 11.11562 | 1.23E-10 | 1.53E-09 | Kif24 |
| 10481627 | -2.49944 | 10.99187 | 11.11469 | 1.23E-10 | 1.53E-09 | Lcn2 |
| 10405464 | -1.36735 | 6.550312 | 11.11411 | 1.24E-10 | 1.53E-09 | Grk6 |
| 10571621 | 1.128195 | 8.537752 | -11.1125 | 1.24E-10 | 1.53E-09 | Ufsp2 |
| 10433003 | 2.495037 | 7.654477 | -11.106 | 1.25E-10 | 1.55E-09 | Sp7 |
| 10516956 | 1.29703 | 5.93808 | -11.1025 | 1.26E-10 | 1.56E-09 | Smpdl3b |
| 10594289 | 1.076816 | 6.12892 | -11.1024 | 1.26E-10 | 1.56E-09 | Glce |
| 10512935 | -1.05792 | 8.329663 | 11.1022 | 1.26E-10 | 1.56E-09 | Amd2 |
| 10450038 | 1.480689 | 6.617106 | -11.1006 | 1.27E-10 | 1.56E-09 | Angptl4 |
| 10542066 | 1.135116 | 6.072073 | -11.0997 | 1.27E-10 | 1.56E-09 | Tspan11 |
| 10425321 | -1.75555 | 8.192065 | 11.09674 | 1.27E-10 | 1.57E-09 | Apobec3 |
| 10357594 | -1.33678 | 6.588479 | 11.09218 | 1.28E-10 | 1.58E-09 | Rassf5 |
| 10523802 | -1.37414 | 5.58663 | 11.08668 | 1.30E-10 | 1.59E-09 | Cdc7 |
| 10547769 | -1.40436 | 7.853725 | 11.0853 | 1.30E-10 | 1.60E-09 | Ptpn6 |
| 10357137 | 1.347672 | 5.691725 | -11.0748 | 1.32E-10 | 1.63E-09 | Gli2 |
| 10391697 | -2.45039 | 6.61368 | 11.0719 | 1.33E-10 | 1.63E-09 | Itga2b |
| 10485372 | -2.07585 | 4.947344 | 11.06283 | 1.35E-10 | 1.66E-09 | Rag1 |
| 10443980 | -1.79021 | 8.290231 | 11.0611 | 1.35E-10 | 1.66E-09 | Myo1f |
| 10517274 | 1.031592 | 6.663784 | -11.0592 | 1.36E-10 | 1.67E-09 | Sepn1 |
| 10561920 | -1.77506 | 8.169364 | 11.03856 | 1.41E-10 | 1.72E-09 | Hcst |
| 10418193 | 1.47304 | 9.881488 | -11.0368 | 1.41E-10 | 1.73E-09 | Plac9b |
| 10405185 | -1.45358 | 8.983311 | 11.02635 | 1.44E-10 | 1.76E-09 | Cks2 |
| 10445268 | 1.143498 | 7.51144 | -11.0251 | 1.44E-10 | 1.76E-09 | Gpr116 |
| 10411622 | -1.4224 | 5.024593 | 11.01856 | 1.46E-10 | 1.78E-09 | Naip6 |
| 10599001 | 2.960192 | 6.330352 | -11.0184 | 1.46E-10 | 1.78E-09 | Agtr2 |
| 10500324 | -1.04892 | 11.17548 | 11.01625 | 1.46E-10 | 1.79E-09 | Hist2h2aa1 |
| 10500329 | -1.02347 | 11.05586 | 11.0148 | 1.47E-10 | 1.79E-09 | Hist2h2aa1 |
| 10441787 | 1.576462 | 7.898915 | -11.0137 | 1.47E-10 | 1.79E-09 | Airn |
| 10585942 | 1.062712 | 4.973659 | -11.0135 | 1.47E-10 | 1.79E-09 | Gramd2 |
| 10557434 | -1.20263 | 6.967083 | 11.01221 | 1.48E-10 | 1.80E-09 | Apobr |
| 10445192 | -4.78128 | 7.222822 | 11.00694 | 1.49E-10 | 1.81E-09 | Rhag |
| 10496262 | 2.26372 | 6.793873 | -11.0049 | 1.49E-10 | 1.82E-09 | Slc9b2 |
| 10452269 | -1.58974 | 6.47401 | 11.00299 | 1.50E-10 | 1.82E-09 | Dennd1c |
| 10605820 | 1.020585 | 6.057996 | -11.0026 | 1.50E-10 | 1.82E-09 | Zc4h2 |
| 10551666 | -1.77136 | 5.99751 | 10.99878 | 1.51E-10 | 1.83E-09 | Map4k1 |
| 10483353 | 2.416246 | 6.92546 | -10.9979 | 1.51E-10 | 1.83E-09 | Scn7a |
| 10504054 | -1.15015 | 5.14148 | 10.99759 | 1.51E-10 | 1.83E-09 | n-R5s183 |
| 10358631 | 2.379726 | 5.963164 | -10.9922 | 1.53E-10 | 1.85E-09 | Hmcn1 |
| 10554240 | -2.50085 | 8.184607 | 10.9919 | 1.53E-10 | 1.85E-09 | Isg20 |
| 10384373 | -1.82721 | 7.418976 | 10.98568 | 1.55E-10 | 1.87E-09 | Fignl1 |
| 10547985 | -1.64371 | 5.186483 | 10.98362 | 1.55E-10 | 1.87E-09 | Cd27 |
| 10438060 | -2.07218 | 5.989074 | 10.98197 | 1.56E-10 | 1.88E-09 | Igll1 |
| 10358577 | 2.279464 | 5.555834 | -10.9796 | 1.56E-10 | 1.89E-09 | Hmcn1 |
| 10515613 | 1.405981 | 6.546158 | -10.9785 | 1.56E-10 | 1.89E-09 | Ptprf |
| 10351679 | -1.37674 | 8.564137 | 10.97552 | 1.57E-10 | 1.90E-09 | Cd84 |
| 10529457 | 2.178193 | 6.582856 | -10.975 | 1.57E-10 | 1.90E-09 | Cpz |
| 10545168 | -1.17337 | 4.708544 | 10.97473 | 1.57E-10 | 1.90E-09 | Tacstd2 |
| 10442224 | 1.433557 | 6.773429 | -10.9737 | 1.58E-10 | 1.90E-09 | Zfp948 |
| 10364601 | -1.79431 | 6.438095 | 10.96889 | 1.59E-10 | 1.91E-09 | Abca7 |
| 10588876 | 1.147038 | 6.314966 | -10.9647 | 1.60E-10 | 1.93E-09 | Nicn1 |
| 10483719 | 1.747852 | 5.727368 | -10.9646 | 1.60E-10 | 1.93E-09 | Chn1 |
| 10346722 | 1.40657 | 7.481545 | -10.9634 | 1.61E-10 | 1.93E-09 | Nbeal1 |
| 10358547 | 2.348744 | 5.30624 | -10.9631 | 1.61E-10 | 1.93E-09 | Hmcn1 |
| 10606694 | -1.87281 | 6.688719 | 10.95929 | 1.62E-10 | 1.94E-09 | Btk |
| 10367734 | 1.371413 | 6.257338 | -10.9554 | 1.63E-10 | 1.95E-09 | Ust |
| 10607395 | 1.317248 | 5.955974 | -10.9554 | 1.63E-10 | 1.95E-09 | Mageh1 |
| 10424905 | 1.5102 | 6.695669 | -10.9437 | 1.66E-10 | 1.99E-09 | Scx |
| 10356461 | -1.27295 | 7.622696 | 10.94304 | 1.66E-10 | 1.99E-09 | Hjurp |
| 10351463 | 1.918005 | 10.26062 | -10.9428 | 1.67E-10 | 1.99E-09 | Rgs5 |
| 10418096 | 1.106399 | 6.367159 | -10.9395 | 1.68E-10 | 2.00E-09 | Dlg5 |
| 10357698 | -2.099 | 6.456493 | 10.93862 | 1.68E-10 | 2.00E-09 | Tmcc2 |
| 10385248 | -1.66958 | 6.943227 | 10.93149 | 1.70E-10 | 2.03E-09 | Hmmr |
| 10471486 | 1.938275 | 7.575353 | -10.9312 | 1.70E-10 | 2.03E-09 | Eng |
| 10534152 | 1.215513 | 5.21446 | -10.9265 | 1.71E-10 | 2.04E-09 | Wbscr17 |
| 10355500 | 1.3438 | 9.5342 | -10.9245 | 1.72E-10 | 2.05E-09 | Igfbp5 |
| 10429926 | -1.20847 | 7.47927 | 10.92124 | 1.73E-10 | 2.06E-09 | Dgat1 |
| 10486119 | -1.92263 | 6.781218 | 10.9199 | 1.73E-10 | 2.06E-09 | Plcb2 |
| 10362899 | -1.63898 | 7.007782 | 10.91397 | 1.75E-10 | 2.08E-09 | F830002L21Rik |
| 10358662 | 1.950673 | 6.169636 | -10.9094 | 1.77E-10 | 2.10E-09 | Hmcn1 |
| 10522653 | -2.6335 | 5.651347 | 10.9073 | 1.77E-10 | 2.10E-09 | A730089K16Rik |
| 10539111 | 1.387773 | 7.14766 | -10.9054 | 1.78E-10 | 2.11E-09 | Tmem150a |
| 10421697 | 1.180657 | 6.478064 | -10.8978 | 1.80E-10 | 2.13E-09 | Lacc1 |
| 10401935 | -1.20395 | 4.112898 | 10.89259 | 1.82E-10 | 2.15E-09 | BC005685 |
| 10403934 | -1.40224 | 7.92171 | 10.89157 | 1.82E-10 | 2.16E-09 | Isca1 |
| 10527920 | 1.018095 | 6.649682 | -10.891 | 1.82E-10 | 2.16E-09 | Cyp51 |
| 10431874 | -1.39829 | 6.911155 | 10.88966 | 1.83E-10 | 2.16E-09 | Slc38a1 |
| 10353733 | -1.02135 | 6.104173 | 10.88942 | 1.83E-10 | 2.16E-09 | Prim2 |
| 10508772 | -1.82211 | 6.981554 | 10.88278 | 1.85E-10 | 2.19E-09 | Fgr |
| 10361110 | -1.50972 | 6.354335 | 10.88124 | 1.86E-10 | 2.19E-09 | Dtl |
| 10607225 | 1.584354 | 4.697689 | -10.8755 | 1.87E-10 | 2.21E-09 | Lrch2 |
| 10556302 | -1.32101 | 7.276919 | 10.87527 | 1.87E-10 | 2.21E-09 | Ampd3 |
| 10409220 | -1.01259 | 11.16891 | 10.87154 | 1.89E-10 | 2.22E-09 | Hist2h2aa1 |
| 10495896 | 1.06355 | 7.836668 | -10.8597 | 1.93E-10 | 2.27E-09 | Camk2d |
| 10544638 | -1.07664 | 7.919818 | 10.85709 | 1.94E-10 | 2.28E-09 | Tra2a |
| 10416655 | -1.37192 | 4.948928 | 10.85562 | 1.94E-10 | 2.28E-09 | Zbtbd6 |
| 10489701 | 2.424828 | 6.674677 | -10.8524 | 1.95E-10 | 2.29E-09 | Ocstamp |
| 10493108 | 1.841394 | 6.339113 | -10.8523 | 1.95E-10 | 2.29E-09 | Crabp2 |
| 10382502 | 1.195136 | 6.005597 | -10.8519 | 1.95E-10 | 2.29E-09 | Cdr2l |
| 10503176 | -1.45358 | 6.755322 | 10.85056 | 1.96E-10 | 2.30E-09 | Chd7 |
| 10403043 | -2.87694 | 7.375726 | 10.84701 | 1.97E-10 | 2.31E-09 | Ighv1-62-3 |
| 10524310 | 1.331488 | 8.043338 | -10.8455 | 1.98E-10 | 2.31E-09 | Ttc28 |
| 10378816 | -2.04599 | 5.557988 | 10.83421 | 2.02E-10 | 2.36E-09 | Slc6a4 |
| 10389581 | 1.197191 | 6.896497 | -10.8337 | 2.02E-10 | 2.36E-09 | Ypel2 |
| 10512747 | -1.33528 | 6.595904 | 10.8333 | 2.02E-10 | 2.36E-09 | 5830415F09Rik |
| 10513666 | -1.8251 | 7.217263 | 10.81863 | 2.07E-10 | 2.42E-09 | Akna |
| 10543959 | 2.934541 | 8.538498 | -10.809 | 2.11E-10 | 2.46E-09 | Ptn |
| 10442087 | 1.142953 | 5.216591 | -10.8067 | 2.12E-10 | 2.46E-09 | Spaca6 |
| 10491272 | -1.2204 | 5.339078 | 10.80349 | 2.13E-10 | 2.48E-09 | Gpr160 |
| 10445627 | 1.0917 | 8.440349 | -10.8028 | 2.13E-10 | 2.48E-09 | 2310039H08Rik |
| 10451061 | 1.09604 | 8.404637 | -10.8 | 2.14E-10 | 2.49E-09 | Runx2 |
| 10528077 | -1.0728 | 6.839627 | 10.79645 | 2.15E-10 | 2.51E-09 | Dbf4 |
| 10493259 | 1.205064 | 9.553194 | -10.7949 | 2.16E-10 | 2.51E-09 | Ssr2 |
| 10379840 | 1.223896 | 6.586548 | -10.7907 | 2.18E-10 | 2.53E-09 | Myo19 |
| 10368675 | 1.092262 | 10.42014 | -10.7906 | 2.18E-10 | 2.53E-09 | Marcks |
| 10419288 | -2.50122 | 6.193008 | 10.78514 | 2.20E-10 | 2.55E-09 | Gch1 |
| 10577449 | -1.0517 | 4.898969 | 10.78132 | 2.21E-10 | 2.57E-09 | Atp7b |
| 10345241 | 1.56899 | 7.912694 | -10.7785 | 2.22E-10 | 2.58E-09 | Dst |
| 10567825 | -1.65575 | 6.886381 | 10.77784 | 2.23E-10 | 2.58E-09 | Lat |
| 10568461 | -1.10759 | 7.327698 | 10.7706 | 2.26E-10 | 2.61E-09 | Rfwd3 |
| 10541049 | -1.19928 | 7.504176 | 10.76922 | 2.26E-10 | 2.61E-09 | 44263 |
| 10398362 | 3.169803 | 5.682788 | -10.7678 | 2.27E-10 | 2.62E-09 | AF357355 |
| 10585699 | 1.771767 | 8.136571 | -10.7652 | 2.28E-10 | 2.63E-09 | Fabp5 |
| 10606071 | -1.50993 | 6.310894 | 10.765 | 2.28E-10 | 2.63E-09 | Ercc6l |
| 10397683 | 1.202182 | 5.62961 | -10.7581 | 2.31E-10 | 2.66E-09 | Ttc8 |
| 10385903 | 1.271209 | 6.836662 | -10.7568 | 2.31E-10 | 2.66E-09 | Pdlim4 |
| 10418180 | 1.449846 | 9.903117 | -10.7537 | 2.32E-10 | 2.68E-09 | Plac9b |
| 10418205 | 1.449846 | 9.903117 | -10.7537 | 2.32E-10 | 2.68E-09 | Plac9b |
| 10490923 | -3.89379 | 9.387307 | 10.75202 | 2.33E-10 | 2.68E-09 | Car2 |
| 10442616 | -1.28199 | 8.209939 | 10.75164 | 2.33E-10 | 2.68E-09 | Hagh |
| 10458046 | 2.313321 | 9.18906 | -10.7513 | 2.33E-10 | 2.68E-09 | Nrep |
| 10533095 | 1.013721 | 6.63118 | -10.749 | 2.34E-10 | 2.69E-09 | Fbxw8 |
| 10362974 | -1.32122 | 6.718281 | 10.73793 | 2.39E-10 | 2.74E-09 | Hace1 |
| 10497590 | 1.135008 | 5.123427 | -10.7311 | 2.42E-10 | 2.77E-09 | Mecom |
| 10349157 | -2.07139 | 5.280024 | 10.7276 | 2.43E-10 | 2.79E-09 | Serpinb2 |
| 10531146 | -1.27383 | 5.143172 | 10.72384 | 2.45E-10 | 2.80E-09 | Mkrn1-ps1 |
| 10545130 | -1.49698 | 9.053873 | 10.72025 | 2.47E-10 | 2.82E-09 | Gadd45a |
| 10575799 | -1.423 | 6.935167 | 10.71933 | 2.47E-10 | 2.82E-09 | Plcg2 |
| 10566926 | -1.06649 | 6.919737 | 10.71607 | 2.48E-10 | 2.84E-09 | Rnf141 |
| 10373530 | -1.22099 | 7.186419 | 10.71437 | 2.49E-10 | 2.84E-09 | Cdk2 |
| 10488291 | 1.010367 | 6.20652 | -10.7099 | 2.51E-10 | 2.86E-09 | Rbbp9 |
| 10358579 | 1.663926 | 5.546056 | -10.7084 | 2.52E-10 | 2.87E-09 | Hmcn1 |
| 10552697 | -1.79578 | 6.450253 | 10.70827 | 2.52E-10 | 2.87E-09 | Napsa |
| 10597098 | -2.03589 | 11.58478 | 10.70687 | 2.53E-10 | 2.87E-09 | Camp |
| 10465861 | -1.48521 | 7.721029 | 10.70638 | 2.53E-10 | 2.88E-09 | Incenp |
| 10598198 | 1.104073 | 7.448536 | -10.6967 | 2.57E-10 | 2.92E-09 | Mia3 |
| 10556076 | 1.58851 | 6.041804 | -10.6942 | 2.58E-10 | 2.93E-09 | Olfml1 |
| 10384539 | 1.121684 | 4.903417 | -10.6916 | 2.59E-10 | 2.94E-09 | Slc1a4 |
| 10365991 | 1.780322 | 4.432586 | -10.6912 | 2.60E-10 | 2.94E-09 | Epyc |
| 10535780 | -1.22498 | 5.389891 | 10.67914 | 2.65E-10 | 3.01E-09 | Flt3 |
| 10358982 | 1.111515 | 6.312447 | -10.6678 | 2.71E-10 | 3.06E-09 | Mr1 |
| 10472364 | 1.381568 | 5.951567 | -10.6626 | 2.73E-10 | 3.09E-09 | Scn2a1 |
| 10405432 | -1.17986 | 6.249212 | 10.66108 | 2.74E-10 | 3.10E-09 | Rgs14 |
| 10606876 | 1.102902 | 8.881403 | -10.6588 | 2.75E-10 | 3.11E-09 | Morf4l2 |
| 10520604 | 1.164612 | 7.015692 | -10.6572 | 2.76E-10 | 3.11E-09 | Emilin1 |
| 10517116 | -1.77749 | 7.477717 | 10.65567 | 2.77E-10 | 3.12E-09 | Rps6ka1 |
| 10391985 | -1.22213 | 7.918591 | 10.65537 | 2.77E-10 | 3.12E-09 | Gm24095 |
| 10405739 | -1.22213 | 7.918591 | 10.65537 | 2.77E-10 | 3.12E-09 | Gm24095 |
| 10409988 | -1.22213 | 7.918591 | 10.65537 | 2.77E-10 | 3.12E-09 | Gm24095 |
| 10583318 | -1.22213 | 7.918591 | 10.65537 | 2.77E-10 | 3.12E-09 | Gm24095 |
| 10516246 | -1.83705 | 7.434612 | 10.6532 | 2.78E-10 | 3.12E-09 | Cdca8 |
| 10349648 | -4.36752 | 8.951949 | 10.65093 | 2.79E-10 | 3.14E-09 | Ctse |
| 10419790 | 1.011803 | 5.054639 | -10.6502 | 2.79E-10 | 3.14E-09 | Ajuba |
| 10428070 | 1.509584 | 5.44561 | -10.6475 | 2.81E-10 | 3.15E-09 | 9430069I07Rik |
| 10511258 | -1.35067 | 7.669584 | 10.64628 | 2.81E-10 | 3.16E-09 | Fam132a |
| 10540472 | 1.554351 | 7.600934 | -10.6439 | 2.82E-10 | 3.17E-09 | Bhlhe40 |
| 10408077 | -1.73563 | 8.173279 | 10.64177 | 2.84E-10 | 3.18E-09 | Hist1h2ak |
| 10572669 | -1.00486 | 5.986088 | 10.63282 | 2.88E-10 | 3.23E-09 | Fam129c |
| 10358565 | 2.459656 | 4.871642 | -10.6323 | 2.88E-10 | 3.23E-09 | Hmcn1 |
| 10358585 | 1.672293 | 5.716377 | -10.6287 | 2.90E-10 | 3.25E-09 | Hmcn1 |
| 10495891 | 1.259614 | 4.766377 | -10.6284 | 2.90E-10 | 3.25E-09 | Arsj |
| 10485402 | 1.24066 | 5.110456 | -10.6268 | 2.91E-10 | 3.25E-09 | Fjx1 |
| 10452815 | -1.1605 | 7.545012 | 10.6196 | 2.95E-10 | 3.29E-09 | Xdh |
| 10481304 | -2.40501 | 7.693789 | 10.61691 | 2.96E-10 | 3.30E-09 | Gfi1b |
| 10369911 | -1.05297 | 6.431233 | 10.6124 | 2.99E-10 | 3.33E-09 | Gucd1 |
| 10486255 | -1.67886 | 5.281537 | 10.61008 | 3.00E-10 | 3.34E-09 | Oip5 |
| 10421418 | -2.47667 | 7.306302 | 10.60973 | 3.00E-10 | 3.34E-09 | Dmtn |
| 10468898 | -1.28489 | 5.305548 | 10.60835 | 3.01E-10 | 3.35E-09 | Lax1 |
| 10449893 | -1.4579 | 5.99571 | 10.60739 | 3.02E-10 | 3.35E-09 | Rasal3 |
| 10502224 | 1.846168 | 8.6167 | -10.6036 | 3.04E-10 | 3.37E-09 | Sgms2 |
| 10449163 | -1.97579 | 8.540576 | 10.60314 | 3.04E-10 | 3.38E-09 | Pigq |
| 10379736 | -2.48122 | 9.81052 | 10.59794 | 3.07E-10 | 3.40E-09 | Wfdc21 |
| 10547100 | 1.708763 | 7.712436 | -10.5942 | 3.09E-10 | 3.42E-09 | Plxnd1 |
| 10563780 | -2.53013 | 7.089064 | 10.59418 | 3.09E-10 | 3.42E-09 | E2f8 |
| 10364529 | -2.7583 | 8.303436 | 10.58792 | 3.12E-10 | 3.45E-09 | Prtn3 |
| 10475990 | -1.18992 | 8.074242 | 10.57869 | 3.17E-10 | 3.50E-09 | Slc20a1 |
| 10546056 | -1.08306 | 6.597579 | 10.5768 | 3.19E-10 | 3.51E-09 | Rab43 |
| 10373810 | 1.247621 | 5.9784 | -10.5757 | 3.19E-10 | 3.52E-09 | Dusp18 |
| 10349593 | -1.98434 | 5.744956 | 10.57501 | 3.20E-10 | 3.52E-09 | Faim3 |
| 10402211 | 1.73118 | 7.969549 | -10.574 | 3.20E-10 | 3.52E-09 | Fbln5 |
| 10592772 | -1.81148 | 6.663999 | 10.56678 | 3.24E-10 | 3.56E-09 | Abcg4 |
| 10590620 | -1.2564 | 4.672922 | 10.56666 | 3.24E-10 | 3.56E-09 | Ccr9 |
| 10404132 | -2.08285 | 5.854209 | 10.56539 | 3.25E-10 | 3.57E-09 | Cmah |
| 10355567 | 1.111747 | 8.055436 | -10.5622 | 3.27E-10 | 3.59E-09 | Tmbim1 |
| 10481147 | 1.275479 | 9.33203 | -10.5562 | 3.30E-10 | 3.62E-09 | Surf4 |
| 10449245 | -1.15448 | 4.380427 | 10.55374 | 3.32E-10 | 3.64E-09 | Pdia2 |
| 10424695 | 1.36532 | 8.291495 | -10.5534 | 3.32E-10 | 3.64E-09 | Gpihbp1 |
| 10503182 | -1.05077 | 5.823781 | 10.55235 | 3.33E-10 | 3.64E-09 | Chd7 |
| 10419223 | 1.721315 | 7.806054 | -10.5429 | 3.38E-10 | 3.70E-09 | Fermt2 |
| 10492169 | 1.266304 | 4.722944 | -10.5414 | 3.39E-10 | 3.70E-09 | Mab21l1 |
| 10598474 | 1.181548 | 6.723837 | -10.5369 | 3.42E-10 | 3.73E-09 | Slc35a2 |
| 10451641 | -1.89826 | 5.291837 | 10.53583 | 3.43E-10 | 3.74E-09 | 9830107B12Rik |
| 10597279 | -1.26674 | 5.91232 | 10.52996 | 3.46E-10 | 3.78E-09 | Ccrl2 |
| 10363082 | -1.39555 | 8.640229 | 10.52486 | 3.50E-10 | 3.80E-09 | Lilrb4 |
| 10571815 | -1.0536 | 5.617083 | 10.52329 | 3.51E-10 | 3.81E-09 | Gpm6a |
| 10349559 | -1.26748 | 6.867118 | 10.52091 | 3.52E-10 | 3.83E-09 | Yod1 |
| 10560919 | -1.70592 | 6.924157 | 10.51608 | 3.55E-10 | 3.86E-09 | Atp1a3 |
| 10358656 | 1.887558 | 6.005323 | -10.5128 | 3.57E-10 | 3.88E-09 | Hmcn1 |
| 10369989 | 1.354234 | 7.794886 | -10.5027 | 3.64E-10 | 3.95E-09 | Ddt |
| 10598093 | -1.48049 | 7.110613 | 10.50152 | 3.65E-10 | 3.95E-09 | Tarm1 |
| 10526520 | 1.108058 | 7.140629 | -10.4977 | 3.67E-10 | 3.98E-09 | Plod3 |
| 10428672 | -1.61428 | 5.510562 | 10.49767 | 3.67E-10 | 3.98E-09 | Dscc1 |
| 10358652 | 3.076539 | 6.093331 | -10.4916 | 3.71E-10 | 4.02E-09 | Hmcn1 |
| 10572398 | 1.311378 | 5.957865 | -10.4902 | 3.72E-10 | 4.03E-09 | Crlf1 |
| 10358650 | 2.227399 | 6.232712 | -10.4862 | 3.75E-10 | 4.05E-09 | Hmcn1 |
| 10400057 | -1.06374 | 7.083122 | 10.47695 | 3.81E-10 | 4.12E-09 | Arl4a |
| 10574676 | 1.076891 | 7.227975 | -10.4669 | 3.88E-10 | 4.19E-09 | Nol3 |
| 10509168 | -2.5563 | 6.877538 | 10.46355 | 3.90E-10 | 4.21E-09 | E2f2 |
| 10547943 | -1.68929 | 7.627155 | 10.46259 | 3.91E-10 | 4.22E-09 | Ncapd2 |
| 10501222 | 1.68664 | 7.839841 | -10.4618 | 3.92E-10 | 4.22E-09 | Gstm2 |
| 10510391 | 1.270474 | 8.415103 | -10.46 | 3.93E-10 | 4.23E-09 | Srm |
| 10502655 | 2.213845 | 8.595399 | -10.46 | 3.93E-10 | 4.23E-09 | Cyr61 |
| 10601011 | -1.48834 | 6.93937 | 10.45944 | 3.93E-10 | 4.23E-09 | Kif4 |
| 10361807 | 1.49907 | 6.963776 | -10.4566 | 3.95E-10 | 4.25E-09 | Hivep2 |
| 10451805 | -1.53264 | 6.813892 | 10.45635 | 3.96E-10 | 4.26E-09 | Sgol1 |
| 10552406 | -2.90564 | 7.553717 | 10.45342 | 3.98E-10 | 4.27E-09 | Nkg7 |
| 10556442 | 1.513079 | 7.403819 | -10.4465 | 4.03E-10 | 4.32E-09 | Tead1 |
| 10450325 | 1.643458 | 7.621916 | -10.4437 | 4.05E-10 | 4.34E-09 | Cfb |
| 10533131 | 1.248204 | 6.945849 | -10.4437 | 4.05E-10 | 4.34E-09 | Plbd2 |
| 10515385 | -1.67805 | 7.636013 | 10.43788 | 4.09E-10 | 4.38E-09 | Urod |
| 10351689 | -1.229 | 5.465928 | 10.43732 | 4.09E-10 | 4.38E-09 | Gm10521 |
| 10376929 | 1.256845 | 6.90205 | -10.4365 | 4.10E-10 | 4.39E-09 | Tvp23b |
| 10516966 | -1.24983 | 7.382811 | 10.42887 | 4.16E-10 | 4.45E-09 | Themis2 |
| 10432675 | -2.12929 | 5.825393 | 10.41995 | 4.23E-10 | 4.52E-09 | I730030J21Rik |
| 10587871 | -1.98536 | 5.937956 | 10.41835 | 4.24E-10 | 4.53E-09 | Paqr9 |
| 10361869 | 1.072647 | 6.442957 | -10.4148 | 4.26E-10 | 4.55E-09 | Nhsl1 |
| 10567173 | 1.186515 | 7.57692 | -10.4147 | 4.27E-10 | 4.55E-09 | Pik3c2a |
| 10531189 | 1.8484 | 6.475264 | -10.4093 | 4.31E-10 | 4.59E-09 | Adamts3 |
| 10545175 | -3.13532 | 6.649016 | 10.40441 | 4.35E-10 | 4.63E-09 | Igkv10-94 |
| 10547009 | 1.148443 | 7.701486 | -10.3981 | 4.40E-10 | 4.68E-09 | Vgll4 |
| 10445702 | -1.0981 | 5.844181 | 10.39633 | 4.41E-10 | 4.69E-09 | Usp49 |
| 10598178 | 1.045439 | 7.594243 | -10.3947 | 4.42E-10 | 4.70E-09 | Disp1 |
| 10357436 | -1.27886 | 8.230806 | 10.39437 | 4.43E-10 | 4.70E-09 | Mcm6 |
| 10393431 | -1.46212 | 7.436039 | 10.39317 | 4.44E-10 | 4.71E-09 | Tk1 |
| 10399391 | -1.57955 | 6.473593 | 10.39227 | 4.44E-10 | 4.72E-09 | Gen1 |
| 10462702 | 1.530944 | 5.74947 | -10.3842 | 4.51E-10 | 4.78E-09 | Hectd2 |
| 10531187 | 1.428539 | 6.380927 | -10.3822 | 4.52E-10 | 4.80E-09 | Adamts3 |
| 10425116 | 1.84031 | 5.674774 | -10.3795 | 4.55E-10 | 4.82E-09 | Cdc42ep1 |
| 10445746 | -1.78603 | 5.543644 | 10.37742 | 4.56E-10 | 4.83E-09 | Trem1 |
| 10388430 | 1.720176 | 10.31352 | -10.3683 | 4.64E-10 | 4.91E-09 | Serpinf1 |
| 10492815 | -1.41698 | 6.80421 | 10.36535 | 4.67E-10 | 4.93E-09 | Tmem154 |
| 10378549 | 1.394041 | 7.049408 | -10.3652 | 4.67E-10 | 4.93E-09 | Rtn4rl1 |
| 10416423 | -1.03288 | 5.459474 | 10.36009 | 4.71E-10 | 4.97E-09 | 5031414D18Rik |
| 10490838 | 1.779779 | 8.055822 | -10.358 | 4.73E-10 | 4.99E-09 | Fabp5 |
| 10396831 | -1.16665 | 5.475227 | 10.35657 | 4.74E-10 | 5.00E-09 | Arg2 |
| 10473349 | -2.78314 | 7.551306 | 10.35386 | 4.76E-10 | 5.02E-09 | Ypel4 |
| 10581340 | -1.83169 | 7.858411 | 10.35115 | 4.79E-10 | 5.05E-09 | Ranbp10 |
| 10412667 | 1.388506 | 6.33151 | -10.3475 | 4.82E-10 | 5.08E-09 | Ptprg |
| 10457323 | 1.546058 | 5.528316 | -10.3442 | 4.85E-10 | 5.11E-09 | Mkx |
| 10352178 | 1.689944 | 7.327189 | -10.3415 | 4.87E-10 | 5.13E-09 | Sccpdh |
| 10448557 | -1.20195 | 6.964148 | 10.34147 | 4.87E-10 | 5.13E-09 | D330041H03Rik |
| 10500804 | 1.179889 | 5.231389 | -10.3414 | 4.87E-10 | 5.13E-09 | LOC269472 |
| 10598507 | -2.50694 | 6.11294 | 10.33758 | 4.91E-10 | 5.16E-09 | Slc38a5 |
| 10586250 | -1.40197 | 7.783112 | 10.33432 | 4.94E-10 | 5.19E-09 | Dennd4a |
| 10432957 | -1.15163 | 5.463267 | 10.32846 | 4.99E-10 | 5.24E-09 | Itgb7 |
| 10472724 | 1.04653 | 8.966842 | -10.3277 | 5.00E-10 | 5.24E-09 | Gorasp2 |
| 10530633 | 1.552972 | 7.370116 | -10.3275 | 5.00E-10 | 5.24E-09 | Sgcb |
| 10497345 | -2.00548 | 5.660081 | 10.32564 | 5.01E-10 | 5.26E-09 | Gm9733 |
| 10497337 | -4.89879 | 9.148151 | 10.32207 | 5.05E-10 | 5.29E-09 | Car1 |
| 10604528 | -1.75051 | 7.230611 | 10.31713 | 5.09E-10 | 5.33E-09 | Mbnl3 |
| 10408613 | 1.661466 | 6.532829 | -10.3056 | 5.20E-10 | 5.42E-09 | Tubb2b |
| 10443527 | -1.26357 | 6.715863 | 10.30487 | 5.21E-10 | 5.43E-09 | Pim1 |
| 10368638 | 1.525229 | 5.954663 | -10.3022 | 5.23E-10 | 5.45E-09 | Fam26e |
| 10548761 | -1.25274 | 7.08847 | 10.30084 | 5.25E-10 | 5.46E-09 | Hebp1 |
| 10572212 | -1.33761 | 6.839561 | 10.29897 | 5.26E-10 | 5.48E-09 | Gmip |
| 10463123 | -2.0191 | 4.726839 | 10.28683 | 5.38E-10 | 5.59E-09 | Dntt |
| 10440513 | 1.422977 | 7.038404 | -10.2866 | 5.38E-10 | 5.59E-09 | Cyyr1 |
| 10439471 | 1.09101 | 6.808847 | -10.2852 | 5.40E-10 | 5.60E-09 | Poglut1 |
| 10599174 | 1.568544 | 6.886069 | -10.2841 | 5.41E-10 | 5.61E-09 | Il13ra1 |
| 10358091 | 1.157575 | 7.231682 | -10.2827 | 5.42E-10 | 5.62E-09 | Nav1 |
| 10403031 | -3.22622 | 6.315531 | 10.27917 | 5.46E-10 | 5.65E-09 | Ighv1-55 |
| 10476326 | -1.02417 | 7.467019 | 10.27786 | 5.47E-10 | 5.67E-09 | Cds2 |
| 10434366 | 1.11945 | 7.683323 | -10.2769 | 5.48E-10 | 5.67E-09 | Dvl3 |
| 10347928 | -1.95172 | 6.303208 | 10.2769 | 5.48E-10 | 5.67E-09 | Sp110 |
| 10582874 | -1.95172 | 6.303208 | 10.2769 | 5.48E-10 | 5.67E-09 | Sp110 |
| 10358603 | 1.141666 | 5.108084 | -10.2746 | 5.50E-10 | 5.69E-09 | Hmcn1 |
| 10503172 | -1.24913 | 4.218476 | 10.27431 | 5.51E-10 | 5.69E-09 | Chd7 |
| 10588707 | -1.42597 | 7.682508 | 10.27296 | 5.52E-10 | 5.70E-09 | Ifrd2 |
| 10586248 | -1.58219 | 7.729285 | 10.26199 | 5.63E-10 | 5.80E-09 | Dennd4a |
| 10430818 | -1.59604 | 6.672425 | 10.25752 | 5.68E-10 | 5.85E-09 | Tnfrsf13c |
| 10359970 | -1.01522 | 6.240008 | 10.25326 | 5.72E-10 | 5.89E-09 | Nos1ap |
| 10519324 | -1.09443 | 8.018573 | 10.2489 | 5.77E-10 | 5.93E-09 | Cdk6 |
| 10350392 | -1.52492 | 6.861242 | 10.24198 | 5.84E-10 | 6.00E-09 | Aspm |
| 10395414 | 1.466126 | 6.23886 | -10.24 | 5.86E-10 | 6.02E-09 | Agmo |
| 10531181 | 1.178326 | 6.019969 | -10.2378 | 5.89E-10 | 6.04E-09 | Adamts3 |
| 10394978 | -2.17417 | 8.44904 | 10.23706 | 5.90E-10 | 6.05E-09 | Rrm2 |
| 10422493 | -1.20815 | 6.602737 | 10.23112 | 5.96E-10 | 6.11E-09 | Gpr18 |
| 10559467 | -1.47167 | 8.301396 | 10.23027 | 5.97E-10 | 6.12E-09 | Gm15448 |
| 10422892 | 1.116699 | 6.079775 | -10.222 | 6.06E-10 | 6.21E-09 | 2410089E03Rik |
| 10458843 | 1.136626 | 6.227744 | -10.2207 | 6.08E-10 | 6.22E-09 | Sema6a |
| 10487480 | -1.3898 | 7.284794 | 10.22004 | 6.08E-10 | 6.23E-09 | Bub1 |
| 10349993 | 1.853847 | 5.720745 | -10.2155 | 6.13E-10 | 6.27E-09 | Myog |
| 10531193 | 1.536768 | 6.900099 | -10.2128 | 6.16E-10 | 6.30E-09 | Adamts3 |
| 10364149 | 1.731459 | 7.644654 | -10.2073 | 6.23E-10 | 6.36E-09 | S100b |
| 10403021 | -2.72262 | 4.912952 | 10.20582 | 6.24E-10 | 6.37E-09 | Ighv1-42 |
| 10590918 | 1.467571 | 7.410873 | -10.2038 | 6.27E-10 | 6.40E-09 | Amotl1 |
| 10605792 | 1.263881 | 5.681498 | -10.1943 | 6.38E-10 | 6.50E-09 | Spin4 |
| 10472916 | -1.62479 | 6.841971 | 10.19414 | 6.38E-10 | 6.50E-09 | Cdca7 |
| 10559606 | -1.92371 | 5.679035 | 10.19149 | 6.41E-10 | 6.53E-09 | Tmem86b |
| 10376444 | -1.75463 | 6.215635 | 10.18622 | 6.47E-10 | 6.58E-09 | Hist3h2ba |
| 10494395 | -1.1134 | 10.88964 | 10.18148 | 6.53E-10 | 6.63E-09 | Hist2h2aa1 |
| 10469312 | 1.243612 | 6.101724 | -10.1777 | 6.57E-10 | 6.67E-09 | Pter |
| 10448278 | -2.25199 | 6.105886 | 10.17694 | 6.58E-10 | 6.68E-09 | Mmp25 |
| 10358557 | 1.261511 | 4.311424 | -10.1761 | 6.59E-10 | 6.68E-09 | Hmcn1 |
| 10488673 | 1.127562 | 6.098977 | -10.1754 | 6.60E-10 | 6.69E-09 | Foxs1 |
| 10378240 | -1.80033 | 5.629144 | 10.17147 | 6.65E-10 | 6.73E-09 | P2rx1 |
| 10404063 | -2.24063 | 7.741699 | 10.16835 | 6.69E-10 | 6.77E-09 | Hist1h2ab |
| 10458340 | 1.286562 | 6.03254 | -10.1618 | 6.77E-10 | 6.84E-09 | Hbegf |
| 10350733 | 1.165709 | 6.519977 | -10.1584 | 6.81E-10 | 6.88E-09 | Rgs16 |
| 10351691 | -1.593 | 4.858716 | 10.15835 | 6.81E-10 | 6.88E-09 | Slamf6 |
| 10403941 | -1.53129 | 10.94443 | 10.15565 | 6.85E-10 | 6.91E-09 | Hist1h3f |
| 10448307 | 1.187132 | 6.768437 | -10.1543 | 6.86E-10 | 6.92E-09 | Tnfrsf12a |
| 10583870 | 1.20087 | 6.255726 | -10.1458 | 6.97E-10 | 7.02E-09 | Bmper |
| 10503709 | -1.47237 | 4.953735 | 10.14394 | 7.00E-10 | 7.04E-09 | D130062J21Rik |
| 10562720 | -2.05563 | 7.123167 | 10.14387 | 7.00E-10 | 7.04E-09 | Siglece |
| 10491486 | -1.0184 | 8.577748 | 10.13995 | 7.05E-10 | 7.09E-09 | Atp11b |
| 10423825 | 1.235075 | 6.449981 | -10.1387 | 7.06E-10 | 7.11E-09 | Fzd6 |
| 10500656 | -1.26436 | 4.886887 | 10.13557 | 7.10E-10 | 7.14E-09 | Cd101 |
| 10473367 | -2.39617 | 8.316689 | 10.13379 | 7.13E-10 | 7.16E-09 | Slc43a1 |
| 10406031 | -1.22789 | 8.163697 | 10.13245 | 7.15E-10 | 7.17E-09 | Lpcat1 |
| 10408083 | -1.53365 | 10.94015 | 10.13048 | 7.17E-10 | 7.19E-09 | Hist1h3a |
| 10541246 | -1.54344 | 7.734313 | 10.12451 | 7.25E-10 | 7.26E-09 | Il17ra |
| 10363224 | 1.101292 | 5.53155 | -10.1209 | 7.30E-10 | 7.31E-09 | Fabp7 |
| 10573419 | -1.50763 | 7.319038 | 10.12022 | 7.31E-10 | 7.31E-09 | Lyl1 |
| 10370339 | -1.70413 | 5.046514 | 10.1172 | 7.35E-10 | 7.35E-09 | Trpm2 |
| 10418185 | 1.150478 | 7.753662 | -10.1124 | 7.42E-10 | 7.41E-09 | Tmem254b |
| 10538921 | -3.75725 | 7.66796 | 10.10783 | 7.48E-10 | 7.47E-09 | Igkv1-117 |
| 10395039 | -1.35635 | 4.907925 | 10.1074 | 7.48E-10 | 7.48E-09 | Cmpk2 |
| 10505438 | -1.56782 | 6.297088 | 10.10195 | 7.56E-10 | 7.55E-09 | Orm1 |
| 10404028 | -1.47639 | 10.96049 | 10.09996 | 7.59E-10 | 7.58E-09 | Hist1h3f |
| 10453260 | -1.36146 | 5.420215 | 10.09924 | 7.60E-10 | 7.58E-09 | Haao |
| 10558134 | 1.03788 | 7.178932 | -10.0968 | 7.63E-10 | 7.61E-09 | Plekha1 |
| 10403034 | -2.6499 | 6.650323 | 10.0866 | 7.78E-10 | 7.75E-09 | Ighv8-8 |
| 10408202 | -1.50417 | 10.93789 | 10.07849 | 7.89E-10 | 7.85E-09 | Hist1h3e |
| 10592618 | -1.36212 | 7.255194 | 10.07533 | 7.94E-10 | 7.90E-09 | Tbcel |
| 10488507 | 1.110149 | 7.431062 | -10.0726 | 7.98E-10 | 7.93E-09 | Abhd12 |
| 10454828 | -1.01992 | 7.666419 | 10.07168 | 8.00E-10 | 7.94E-09 | Pnet-ps |
| 10375677 | -1.32345 | 3.513798 | 10.06362 | 8.12E-10 | 8.05E-09 | Mir340 |
| 10566585 | -2.02223 | 7.361023 | 10.06034 | 8.16E-10 | 8.09E-09 | Gm1966 |
| 10600500 | -1.45557 | 7.866271 | 10.06025 | 8.17E-10 | 8.09E-09 | Fam220a |
| 10481574 | -1.32405 | 6.01586 | 10.05776 | 8.20E-10 | 8.11E-09 | Fam78a |
| 10525236 | -1.6604 | 7.688236 | 10.05703 | 8.22E-10 | 8.12E-09 | Gm15800 |
| 10534679 | -1.54965 | 8.535742 | 10.05472 | 8.25E-10 | 8.15E-09 | Trim56 |
| 10385826 | -2.2491 | 5.330633 | 10.04687 | 8.37E-10 | 8.25E-09 | Sowaha |
| 10519105 | 1.004658 | 7.741301 | -10.0439 | 8.42E-10 | 8.29E-09 | Ski |
| 10471457 | 1.281754 | 6.536025 | -10.0413 | 8.46E-10 | 8.33E-09 | St6galnac4 |
| 10591653 | 1.024031 | 7.362762 | -10.0394 | 8.49E-10 | 8.36E-09 | Tmem205 |
| 10404049 | -1.51562 | 11.05497 | 10.03703 | 8.53E-10 | 8.39E-09 | Hist1h3a |
| 10514510 | 1.309386 | 5.347883 | -10.0307 | 8.63E-10 | 8.48E-09 | Cyp2j6 |
| 10497358 | -1.49389 | 7.450835 | 10.01748 | 8.84E-10 | 8.68E-09 | Sirpb1a |
| 10540207 | 1.13384 | 4.956203 | -10.0163 | 8.86E-10 | 8.69E-09 | A730049H05Rik |
| 10571840 | 1.396772 | 8.741903 | -10.0161 | 8.86E-10 | 8.69E-09 | Hpgd |
| 10577954 | -1.1739 | 5.81762 | 10.00762 | 9.00E-10 | 8.82E-09 | Rab11fip1 |
| 10569714 | 1.41411 | 6.037148 | -10.0055 | 9.04E-10 | 8.85E-09 | Cacng7 |
| 10593878 | 1.038833 | 5.634617 | -10.0049 | 9.05E-10 | 8.85E-09 | Snx33 |
| 10415857 | 1.24373 | 5.512028 | -10.0043 | 9.06E-10 | 8.86E-09 | Fam167a |
| 10408081 | -1.69734 | 9.912022 | 9.999428 | 9.14E-10 | 8.94E-09 | Hist1h1b |
| 10586252 | -1.46579 | 7.333126 | 9.99831 | 9.16E-10 | 8.95E-09 | Dennd4a |
| 10458828 | 2.069705 | 7.441506 | -9.98837 | 9.33E-10 | 9.11E-09 | Cdo1 |
| 10369388 | 1.901095 | 6.780884 | -9.98152 | 9.45E-10 | 9.22E-09 | Unc5b |
| 10438017 | -1.0015 | 6.898843 | 9.967949 | 9.69E-10 | 9.44E-09 | Fgd4 |
| 10358561 | 1.784558 | 5.084664 | -9.96583 | 9.73E-10 | 9.47E-09 | Hmcn1 |
| 10418198 | 1.172714 | 7.730989 | -9.96098 | 9.82E-10 | 9.55E-09 | Tmem254b |
| 10536429 | 1.137032 | 8.65725 | -9.95845 | 9.87E-10 | 9.59E-09 | Tmem106b |
| 10404065 | -1.52562 | 11.0346 | 9.95518 | 9.93E-10 | 9.64E-09 | Hist1h3f |
| 10504957 | -1.45683 | 8.037049 | 9.955007 | 9.93E-10 | 9.64E-09 | Smc2 |
| 10585276 | -2.0965 | 6.389459 | 9.952547 | 9.98E-10 | 9.68E-09 | Pou2af1 |
| 10497372 | -1.70612 | 7.295887 | 9.94513 | 1.01E-09 | 9.81E-09 | Gm5150 |
| 10381708 | -1.44555 | 6.697188 | 9.943201 | 1.02E-09 | 9.84E-09 | Fmnl1 |
| 10528476 | -1.11066 | 3.97974 | 9.941479 | 1.02E-09 | 9.87E-09 | Gm22887 |
| 10389606 | -1.53273 | 7.778542 | 9.938312 | 1.02E-09 | 9.91E-09 | Prr11 |
| 10581961 | 1.45094 | 5.223134 | -9.93763 | 1.03E-09 | 9.92E-09 | Adamts18 |
| 10394735 | 1.003528 | 8.687757 | -9.93617 | 1.03E-09 | 9.94E-09 | Pdia6 |
| 10558811 | -1.36803 | 7.379045 | 9.935677 | 1.03E-09 | 9.95E-09 | Ptdss2 |
| 10443421 | -1.34548 | 6.567019 | 9.935653 | 1.03E-09 | 9.95E-09 | Brpf3 |
| 10513608 | -2.20464 | 8.026009 | 9.929266 | 1.04E-09 | 1.00E-08 | Alad |
| 10408246 | -1.48818 | 10.94747 | 9.927287 | 1.05E-09 | 1.01E-08 | Hist1h3f |
| 10350102 | -1.44409 | 6.507363 | 9.925897 | 1.05E-09 | 1.01E-08 | Ptpn7 |
| 10405179 | 1.50765 | 7.126944 | -9.92444 | 1.05E-09 | 1.01E-08 | S1pr3 |
| 10400170 | 1.137606 | 4.720612 | -9.92282 | 1.05E-09 | 1.01E-08 | Prkd1 |
| 10586240 | -1.51162 | 9.26894 | 9.922009 | 1.06E-09 | 1.02E-08 | Dennd4a |
| 10583071 | 1.482089 | 5.5018 | -9.92185 | 1.06E-09 | 1.02E-08 | Mmp3 |
| 10439296 | -1.98033 | 6.389137 | 9.920811 | 1.06E-09 | 1.02E-08 | Stfa2 |
| 10408239 | -1.5247 | 11.00057 | 9.920034 | 1.06E-09 | 1.02E-08 | Hist1h3a |
| 10446074 | -1.60628 | 7.658884 | 9.913519 | 1.07E-09 | 1.03E-08 | Uhrf1 |
| 10478572 | -1.66269 | 9.000453 | 9.912571 | 1.08E-09 | 1.03E-08 | Ube2c |
| 10495781 | 1.701825 | 6.582058 | -9.90957 | 1.08E-09 | 1.04E-08 | Bcar3 |
| 10469058 | 3.221579 | 6.1497 | -9.90836 | 1.08E-09 | 1.04E-08 | Ucma |
| 10353844 | -1.2259 | 6.343037 | 9.904585 | 1.09E-09 | 1.04E-08 | Neurl3 |
| 10542164 | -1.37863 | 9.10318 | 9.903872 | 1.09E-09 | 1.05E-08 | Clec12a |
| 10515994 | -1.02215 | 8.259942 | 9.903246 | 1.09E-09 | 1.05E-08 | Smap2 |
| 10476945 | -2.1146 | 6.982035 | 9.901109 | 1.10E-09 | 1.05E-08 | Cst7 |
| 10478962 | -1.66355 | 7.225161 | 9.899202 | 1.10E-09 | 1.05E-08 | Fam210b |
| 10416653 | -1.18243 | 6.540753 | 9.894202 | 1.11E-09 | 1.06E-08 | Kbtbd7 |
| 10346960 | 1.013567 | 6.891389 | -9.89234 | 1.12E-09 | 1.07E-08 | Ccnyl1 |
| 10437590 | -1.21632 | 8.376503 | 9.891434 | 1.12E-09 | 1.07E-08 | Carhsp1 |
| 10404389 | -1.49601 | 4.661318 | 9.891163 | 1.12E-09 | 1.07E-08 | Irf4 |
| 10588731 | 1.048853 | 5.647975 | -9.88931 | 1.12E-09 | 1.07E-08 | Mst1r |
| 10451679 | 1.120499 | 6.401733 | -9.88824 | 1.13E-09 | 1.07E-08 | Daam2 |
| 10359908 | 1.611287 | 5.709557 | -9.88595 | 1.13E-09 | 1.08E-08 | Rgs4 |
| 10358567 | 2.157067 | 5.14833 | -9.87525 | 1.15E-09 | 1.10E-08 | Hmcn1 |
| 10401933 | -1.27228 | 5.397547 | 9.869257 | 1.17E-09 | 1.11E-08 | Sympk |
| 10504759 | -1.27228 | 5.397547 | 9.869257 | 1.17E-09 | 1.11E-08 | Sympk |
| 10351111 | 1.38578 | 6.364233 | -9.86542 | 1.17E-09 | 1.11E-08 | Dnm3os |
| 10353934 | 1.144802 | 7.264896 | -9.86317 | 1.18E-09 | 1.12E-08 | Actr1b |
| 10418210 | 1.133365 | 7.70131 | -9.86297 | 1.18E-09 | 1.12E-08 | Tmem254b |
| 10440534 | 1.249963 | 7.463367 | -9.85618 | 1.19E-09 | 1.13E-08 | Adamts5 |
| 10492021 | 2.546952 | 10.56614 | -9.85426 | 1.20E-09 | 1.14E-08 | Postn |
| 10460891 | -1.64213 | 7.046808 | 9.852016 | 1.20E-09 | 1.14E-08 | Map4k2 |
| 10356293 | -1.20378 | 6.620909 | 9.849436 | 1.21E-09 | 1.15E-08 | A630001G21Rik |
| 10360070 | -1.00468 | 10.90756 | 9.849225 | 1.21E-09 | 1.15E-08 | Fcer1g |
| 10435149 | 1.021857 | 8.174378 | -9.84919 | 1.21E-09 | 1.15E-08 | Fyttd1 |
| 10567043 | 1.145148 | 7.195341 | -9.84893 | 1.21E-09 | 1.15E-08 | Rras2 |
| 10444306 | -1.12647 | 4.655014 | 9.846863 | 1.22E-09 | 1.15E-08 | H2-Eb2 |
| 10491136 | -1.18419 | 6.219594 | 9.842228 | 1.23E-09 | 1.16E-08 | Tnik |
| 10438064 | -2.66002 | 7.478041 | 9.840866 | 1.23E-09 | 1.16E-08 | Vpreb1 |
| 10524684 | 1.593412 | 5.348225 | -9.83952 | 1.23E-09 | 1.16E-08 | Msi1 |
| 10586254 | -1.32402 | 8.515335 | 9.83756 | 1.24E-09 | 1.17E-08 | Dennd4a |
| 10522655 | -1.53779 | 4.341608 | 9.834687 | 1.24E-09 | 1.17E-08 | C530008M17Rik |
| 10477637 | 1.303755 | 8.15461 | -9.83352 | 1.25E-09 | 1.17E-08 | Map1lc3a |
| 10548899 | 1.640873 | 5.730654 | -9.81905 | 1.28E-09 | 1.21E-08 | Rerg |
| 10493086 | -1.19418 | 8.470828 | 9.81616 | 1.29E-09 | 1.21E-08 | Hdgf |
| 10409567 | -1.48438 | 5.864596 | 9.812886 | 1.30E-09 | 1.22E-08 | Tifab |
| 10555460 | -1.3735 | 7.111389 | 9.809263 | 1.31E-09 | 1.23E-08 | Stard10 |
| 10492231 | -1.50427 | 5.385269 | 9.797742 | 1.33E-09 | 1.25E-08 | Med12l |
| 10523717 | 1.456031 | 10.89957 | -9.79738 | 1.33E-09 | 1.25E-08 | Spp1 |
| 10498357 | -1.27399 | 5.276819 | 9.793364 | 1.34E-09 | 1.26E-08 | F630111L10Rik |
| 10495945 | -1.18612 | 5.539771 | 9.793248 | 1.35E-09 | 1.26E-08 | Zgrf1 |
| 10587231 | 1.691128 | 6.956131 | -9.78332 | 1.37E-09 | 1.28E-08 | Bmp5 |
| 10484237 | 1.030415 | 5.636279 | -9.78273 | 1.37E-09 | 1.28E-08 | Zfp385b |
| 10590245 | -1.30288 | 6.680899 | 9.78071 | 1.38E-09 | 1.29E-08 | Slc25a38 |
| 10573054 | -4.90121 | 8.548001 | 9.775905 | 1.39E-09 | 1.30E-08 | Gypa |
| 10597182 | -1.54482 | 6.862594 | 9.769493 | 1.41E-09 | 1.31E-08 | Nbeal2 |
| 10346843 | 1.686508 | 8.013012 | -9.76889 | 1.41E-09 | 1.31E-08 | Nrp2 |
| 10414315 | -1.35867 | 7.677441 | 9.764345 | 1.42E-09 | 1.32E-08 | Cdkn3 |
| 10467739 | 1.226185 | 6.718074 | -9.75238 | 1.45E-09 | 1.35E-08 | Avpi1 |
| 10348321 | -1.0193 | 7.542886 | 9.749134 | 1.46E-09 | 1.36E-08 | Dgkd |
| 10377148 | 1.866157 | 6.340463 | -9.74271 | 1.48E-09 | 1.38E-08 | Myh8 |
| 10461930 | -1.30339 | 7.292527 | 9.738604 | 1.49E-09 | 1.39E-08 | D030056L22Rik |
| 10500204 | 1.904111 | 9.465417 | -9.73645 | 1.50E-09 | 1.39E-08 | Ecm1 |
| 10546184 | 1.303945 | 7.615133 | -9.73612 | 1.50E-09 | 1.39E-08 | Plxna1 |
| 10444284 | -2.08727 | 5.478074 | 9.735371 | 1.50E-09 | 1.39E-08 | H2-Ob |
| 10430956 | 1.505939 | 9.518759 | -9.72938 | 1.52E-09 | 1.41E-08 | Cyb5r3 |
| 10460253 | -1.25583 | 4.987102 | 9.727619 | 1.52E-09 | 1.41E-08 | Aldh3b2 |
| 10494262 | 2.16898 | 9.952848 | -9.7209 | 1.54E-09 | 1.43E-08 | Ctsk |
| 10531724 | -2.04699 | 8.992069 | 9.719357 | 1.55E-09 | 1.43E-08 | Plac8 |
| 10594762 | 1.007308 | 4.417393 | -9.70565 | 1.59E-09 | 1.47E-08 | Fam81a |
| 10526181 | 1.108815 | 6.623978 | -9.70187 | 1.60E-09 | 1.48E-08 | Gatsl2 |
| 10358623 | 1.85849 | 4.758753 | -9.69782 | 1.61E-09 | 1.49E-08 | Hmcn1 |
| 10400141 | -1.03782 | 5.806862 | 9.696264 | 1.62E-09 | 1.49E-08 | Zbed4 |
| 10596893 | 1.187925 | 7.353915 | -9.69491 | 1.62E-09 | 1.50E-08 | Dag1 |
| 10481518 | 1.112654 | 6.388768 | -9.68406 | 1.65E-09 | 1.52E-08 | Ptges |
| 10586591 | 2.056311 | 5.749431 | -9.68318 | 1.66E-09 | 1.53E-08 | Car12 |
| 10578904 | 2.214512 | 8.979205 | -9.67868 | 1.67E-09 | 1.54E-08 | Cpe |
| 10421186 | -1.7695 | 6.21684 | 9.67545 | 1.68E-09 | 1.55E-08 | Gm10002 |
| 10456184 | 1.158241 | 6.215645 | -9.67474 | 1.68E-09 | 1.55E-08 | Apcdd1 |
| 10471129 | 1.106824 | 4.151383 | -9.66633 | 1.71E-09 | 1.57E-08 | Ncs1 |
| 10387821 | -2.84443 | 7.462553 | 9.663353 | 1.72E-09 | 1.58E-08 | Alox12 |
| 10392642 | 1.225876 | 5.940214 | -9.66129 | 1.73E-09 | 1.58E-08 | Abca5 |
| 10475264 | -1.19604 | 7.605027 | 9.655672 | 1.75E-09 | 1.60E-08 | Ccndbp1 |
| 10526654 | -1.20924 | 6.637369 | 9.651494 | 1.76E-09 | 1.61E-08 | Gm7285 |
| 10524312 | 1.208345 | 7.648024 | -9.64806 | 1.77E-09 | 1.62E-08 | Ttc28 |
| 10371296 | 1.195863 | 5.276464 | -9.64626 | 1.78E-09 | 1.63E-08 | Glt8d2 |
| 10597973 | 1.182883 | 8.63836 | -9.64594 | 1.78E-09 | 1.63E-08 | Lztfl1 |
| 10374236 | 1.106199 | 5.453185 | -9.64126 | 1.79E-09 | 1.64E-08 | Upp1 |
| 10507500 | -1.72931 | 6.999214 | 9.640315 | 1.80E-09 | 1.64E-08 | Slc6a9 |
| 10345762 | 1.519527 | 7.731523 | -9.63815 | 1.80E-09 | 1.65E-08 | Il1r1 |
| 10366707 | 1.479711 | 5.313533 | -9.63629 | 1.81E-09 | 1.65E-08 | Avpr1a |
| 10503210 | -1.01479 | 7.422342 | 9.625845 | 1.85E-09 | 1.68E-08 | Chd7 |
| 10402991 | -1.94859 | 6.673664 | 9.624666 | 1.85E-09 | 1.68E-08 | Ighv2-4 |
| 10475437 | -1.3587 | 8.36671 | 9.623374 | 1.86E-09 | 1.69E-08 | Sord |
| 10363962 | -1.81558 | 5.757433 | 9.618336 | 1.87E-09 | 1.70E-08 | Gnaz |
| 10563338 | -2.23094 | 7.545241 | 9.615861 | 1.88E-09 | 1.71E-08 | Ppp1r15a |
| 10521111 | 1.653837 | 6.569656 | -9.61553 | 1.88E-09 | 1.71E-08 | Fgfr3 |
| 10431659 | 1.331871 | 5.60948 | -9.61463 | 1.89E-09 | 1.71E-08 | Kif21a |
| 10494804 | 1.632472 | 5.774143 | -9.6069 | 1.92E-09 | 1.74E-08 | Casq2 |
| 10456556 | 1.060178 | 5.022637 | -9.60173 | 1.93E-09 | 1.75E-08 | Stard6 |
| 10530783 | -1.88475 | 5.759502 | 9.59918 | 1.94E-09 | 1.76E-08 | A730089K16Rik |
| 10487011 | -1.34498 | 6.856569 | 9.588951 | 1.98E-09 | 1.79E-08 | Gatm |
| 10409557 | -1.06618 | 7.546104 | 9.587566 | 1.99E-09 | 1.80E-08 | H2afy |
| 10395293 | -1.57567 | 7.602477 | 9.574981 | 2.04E-09 | 1.83E-08 | Atxn7l1 |
| 10420268 | 2.183965 | 5.716099 | -9.57301 | 2.04E-09 | 1.84E-08 | Gzme |
| 10596857 | -1.02213 | 7.42033 | 9.566384 | 2.07E-09 | 1.86E-08 | Apeh |
| 10358591 | 1.02261 | 5.782869 | -9.56553 | 2.07E-09 | 1.86E-08 | Hmcn1 |
| 10561187 | 2.739525 | 5.284933 | -9.56483 | 2.08E-09 | 1.87E-08 | Mia |
| 10503170 | -1.18022 | 7.890888 | 9.562238 | 2.09E-09 | 1.87E-08 | Chd7 |
| 10553833 | 1.952611 | 5.495161 | -9.56189 | 2.09E-09 | 1.87E-08 | Ndn |
| 10413047 | 1.098018 | 7.512585 | -9.55784 | 2.10E-09 | 1.89E-08 | Plau |
| 10531195 | 1.375255 | 4.181049 | -9.55676 | 2.11E-09 | 1.89E-08 | Adamts3 |
| 10359582 | -1.66149 | 5.763158 | 9.556525 | 2.11E-09 | 1.89E-08 | Fmo2 |
| 10488697 | -1.25231 | 7.150589 | 9.553694 | 2.12E-09 | 1.90E-08 | Plagl2 |
| 10548905 | 1.075087 | 6.589773 | -9.55058 | 2.13E-09 | 1.91E-08 | Eps8 |
| 10450496 | -1.30174 | 8.931731 | 9.549845 | 2.14E-09 | 1.91E-08 | Lst1 |
| 10601421 | 2.232825 | 6.266632 | -9.5485 | 2.14E-09 | 1.92E-08 | A630033H20Rik |
| 10368243 | 1.457775 | 6.757449 | -9.53812 | 2.19E-09 | 1.95E-08 | Eya4 |
| 10507112 | -1.66979 | 6.054164 | 9.531058 | 2.21E-09 | 1.98E-08 | Stil |
| 10385391 | -1.98956 | 7.674189 | 9.529145 | 2.22E-09 | 1.98E-08 | Cyfip2 |
| 10356267 | -1.01054 | 7.173342 | 9.528003 | 2.23E-09 | 1.99E-08 | LOC100041877 |
| 10391649 | -4.38448 | 8.861517 | 9.524512 | 2.24E-09 | 2.00E-08 | Slc4a1 |
| 10375062 | -1.70458 | 5.288744 | 9.523954 | 2.25E-09 | 2.00E-08 | Hbq1a |
| 10598152 | -1.45435 | 5.67046 | 9.521333 | 2.26E-09 | 2.01E-08 | BC147527 |
| 10437160 | 1.30628 | 7.424271 | -9.52013 | 2.26E-09 | 2.01E-08 | Ets2 |
| 10509178 | 1.111582 | 5.872123 | -9.51792 | 2.27E-09 | 2.02E-08 | Asap3 |
| 10443749 | -1.35315 | 4.988412 | 9.51499 | 2.28E-09 | 2.03E-08 | Ubash3a |
| 10358535 | 1.785599 | 4.114929 | -9.51413 | 2.29E-09 | 2.03E-08 | Hmcn1 |
| 10393879 | -1.08833 | 8.07528 | 9.513478 | 2.29E-09 | 2.04E-08 | Mafg |
| 10553131 | 1.041197 | 8.837268 | -9.51279 | 2.29E-09 | 2.04E-08 | Kdelr1 |
| 10565935 | 1.064916 | 6.503895 | -9.51039 | 2.30E-09 | 2.05E-08 | Arhgef17 |
| 10521972 | 1.158946 | 6.851073 | -9.50776 | 2.32E-09 | 2.06E-08 | Pcdh7 |
| 10437205 | 1.342375 | 6.299411 | -9.50417 | 2.33E-09 | 2.07E-08 | Pcp4 |
| 10366645 | -1.34376 | 5.531961 | 9.503413 | 2.34E-09 | 2.07E-08 | 1700006J14Rik |
| 10450374 | -1.29144 | 9.073953 | 9.496112 | 2.37E-09 | 2.10E-08 | D17H6S56E-5 |
| 10460108 | 1.376692 | 7.074837 | -9.49586 | 2.37E-09 | 2.10E-08 | Gnpnat1 |
| 10368683 | 1.412783 | 5.634111 | -9.49324 | 2.38E-09 | 2.11E-08 | Fam229b |
| 10354777 | 2.074199 | 7.776676 | -9.48486 | 2.42E-09 | 2.14E-08 | Satb2 |
| 10384233 | 1.162657 | 8.381027 | -9.48393 | 2.42E-09 | 2.14E-08 | Tns3 |
| 10607475 | 1.25696 | 8.9673 | -9.48352 | 2.43E-09 | 2.14E-08 | Prdx4 |
| 10465314 | -1.00499 | 7.545152 | 9.472757 | 2.48E-09 | 2.18E-08 | Capn1 |
| 10480347 | -1.01355 | 8.072617 | 9.469765 | 2.49E-09 | 2.19E-08 | Pip4k2a |
| 10363445 | -1.48616 | 7.829577 | 9.468877 | 2.50E-09 | 2.20E-08 | 4632428N05Rik |
| 10401296 | 1.567923 | 6.470291 | -9.46616 | 2.51E-09 | 2.21E-08 | Slc8a3 |
| 10542857 | -1.19997 | 5.582176 | 9.454021 | 2.57E-09 | 2.25E-08 | Far2 |
| 10370446 | -1.05711 | 7.386437 | 9.453263 | 2.57E-09 | 2.25E-08 | Trappc10 |
| 10419151 | -3.66604 | 7.641376 | 9.450664 | 2.59E-09 | 2.26E-08 | Ear1 |
| 10390458 | 1.188899 | 5.835026 | -9.44712 | 2.60E-09 | 2.28E-08 | Pcgf2 |
| 10501586 | 1.059163 | 8.37479 | -9.44 | 2.64E-09 | 2.30E-08 | S1pr1 |
| 10352000 | -1.61342 | 4.628249 | 9.437397 | 2.65E-09 | 2.32E-08 | Kmo |
| 10589535 | -2.01158 | 11.07954 | 9.435121 | 2.66E-09 | 2.32E-08 | Ngp |
| 10520544 | 1.332734 | 7.31301 | -9.4331 | 2.67E-09 | 2.33E-08 | Mapre3 |
| 10436456 | 1.165853 | 7.893163 | -9.43019 | 2.69E-09 | 2.34E-08 | Pros1 |
| 10492824 | -1.22633 | 4.439561 | 9.427131 | 2.71E-09 | 2.36E-08 | Tmem154 |
| 10517677 | 1.332843 | 6.684037 | -9.42659 | 2.71E-09 | 2.36E-08 | Nbl1 |
| 10440417 | 1.492765 | 6.043702 | -9.42549 | 2.71E-09 | 2.36E-08 | LOC102633149 |
| 10393064 | -1.34455 | 5.403687 | 9.425322 | 2.71E-09 | 2.36E-08 | Unc13d |
| 10512226 | -1.14399 | 8.80173 | 9.422014 | 2.73E-09 | 2.38E-08 | Dcaf12 |
| 10445953 | -1.49697 | 5.464598 | 9.421182 | 2.74E-09 | 2.38E-08 | Emr4 |
| 10500345 | -1.57124 | 6.348622 | 9.419529 | 2.75E-09 | 2.39E-08 | Terc |
| 10474381 | -1.75872 | 7.1552 | 9.415372 | 2.77E-09 | 2.40E-08 | Kif18a |
| 10401607 | 1.099301 | 5.096357 | -9.40754 | 2.81E-09 | 2.44E-08 | Pgf |
| 10508454 | -1.48363 | 8.975237 | 9.405714 | 2.82E-09 | 2.45E-08 | Bsdc1 |
| 10603099 | 1.510826 | 6.330085 | -9.40228 | 2.84E-09 | 2.46E-08 | Figf |
| 10427468 | -1.42641 | 7.884859 | 9.401106 | 2.84E-09 | 2.47E-08 | LOC102633612 |
| 10473399 | -3.12593 | 8.7917 | 9.394413 | 2.88E-09 | 2.49E-08 | Prg2 |
| 10377018 | 2.065787 | 5.701715 | -9.3913 | 2.90E-09 | 2.51E-08 | Myh3 |
| 10402864 | -1.846 | 5.98792 | 9.383085 | 2.95E-09 | 2.54E-08 | Igh-VX24 |
| 10479159 | -1.22643 | 5.326648 | 9.382624 | 2.95E-09 | 2.55E-08 | Zfp831 |
| 10505489 | 1.163524 | 6.853982 | -9.37948 | 2.97E-09 | 2.56E-08 | Pappa |
| 10466441 | -1.2397 | 7.057794 | 9.375332 | 2.99E-09 | 2.58E-08 | Vps13a |
| 10563099 | -2.04788 | 5.52529 | 9.372026 | 3.01E-09 | 2.59E-08 | Snord35b |
| 10531175 | 1.005245 | 4.529837 | -9.36971 | 3.02E-09 | 2.60E-08 | Adamts3 |
| 10397966 | -1.16404 | 6.012664 | 9.366989 | 3.04E-09 | 2.61E-08 | Otub2 |
| 10400971 | 1.053545 | 5.661653 | -9.36548 | 3.05E-09 | 2.62E-08 | Six4 |
| 10494548 | 1.139476 | 5.284378 | -9.36425 | 3.06E-09 | 2.63E-08 | Gja5 |
| 10503214 | -1.16701 | 5.13441 | 9.36156 | 3.07E-09 | 2.64E-08 | Chd7 |
| 10440206 | 1.341839 | 6.290376 | -9.35949 | 3.08E-09 | 2.65E-08 | Arl6 |
| 10507101 | 1.201525 | 6.619476 | -9.35858 | 3.09E-09 | 2.65E-08 | Trabd2b |
| 10408600 | 1.322601 | 9.795649 | -9.35703 | 3.10E-09 | 2.66E-08 | Serpinb6a |
| 10599487 | -1.405 | 6.209933 | 9.355595 | 3.11E-09 | 2.66E-08 | Sash3 |
| 10458195 | -1.19916 | 5.883536 | 9.354428 | 3.11E-09 | 2.67E-08 | Cdc25c |
| 10367775 | -1.06148 | 8.353645 | 9.350046 | 3.14E-09 | 2.69E-08 | Stxbp5 |
| 10573261 | -1.55301 | 7.086152 | 9.347867 | 3.15E-09 | 2.70E-08 | Asf1b |
| 10454369 | 1.110105 | 5.875835 | -9.33574 | 3.23E-09 | 2.76E-08 | Fhod3 |
| 10571705 | -1.00846 | 7.887876 | 9.328084 | 3.28E-09 | 2.80E-08 | Irf2 |
| 10368268 | 1.11259 | 4.219757 | -9.3278 | 3.28E-09 | 2.80E-08 | Gm24655 |
| 10567995 | 1.587608 | 8.926003 | -9.32765 | 3.28E-09 | 2.80E-08 | Nupr1 |
| 10529264 | 1.265945 | 5.989311 | -9.32682 | 3.29E-09 | 2.80E-08 | Spon2 |
| 10433480 | 1.912969 | 6.192411 | -9.32264 | 3.31E-09 | 2.82E-08 | Rpl39l |
| 10581434 | 1.007203 | 5.050527 | -9.32235 | 3.31E-09 | 2.82E-08 | Dpep2 |
| 10515028 | 1.268789 | 6.214619 | -9.31805 | 3.34E-09 | 2.84E-08 | Zfyve9 |
| 10528015 | 1.127114 | 4.82426 | -9.31768 | 3.34E-09 | 2.85E-08 | Steap1 |
| 10499378 | -1.98598 | 7.158179 | 9.315276 | 3.36E-09 | 2.86E-08 | Sema4a |
| 10376201 | 1.436432 | 10.55353 | -9.31499 | 3.36E-09 | 2.86E-08 | Gpx3 |
| 10475517 | -1.92639 | 7.419157 | 9.314817 | 3.36E-09 | 2.86E-08 | AA467197 |
| 10435982 | -1.34712 | 5.184398 | 9.313725 | 3.37E-09 | 2.86E-08 | Btla |
| 10607738 | 1.231013 | 5.018775 | -9.31034 | 3.39E-09 | 2.88E-08 | Car5b |
| 10358894 | -1.38634 | 8.399437 | 9.309824 | 3.40E-09 | 2.88E-08 | Sord |
| 10484503 | 1.31362 | 5.71586 | -9.3083 | 3.41E-09 | 2.89E-08 | Lrrc55 |
| 10386495 | 1.051339 | 7.215053 | -9.30547 | 3.42E-09 | 2.90E-08 | Tom1l2 |
| 10520521 | -1.27695 | 8.174673 | 9.303033 | 3.44E-09 | 2.92E-08 | Cenpa |
| 10567108 | -1.08005 | 7.543657 | 9.29395 | 3.50E-09 | 2.97E-08 | Sox6 |
| 10347767 | 1.205544 | 5.158688 | -9.29276 | 3.51E-09 | 2.97E-08 | Kcne4 |
| 10601844 | 1.288609 | 5.9772 | -9.2855 | 3.56E-09 | 3.01E-08 | Bhlhb9 |
| 10392440 | -1.50756 | 6.679065 | 9.285466 | 3.56E-09 | 3.01E-08 | Slc16a6 |
| 10569181 | -1.14152 | 5.805698 | 9.2849 | 3.56E-09 | 3.01E-08 | Pidd1 |
| 10389395 | -1.04869 | 5.718309 | 9.284155 | 3.57E-09 | 3.02E-08 | Brip1 |
| 10455259 | -1.04843 | 7.217315 | 9.280926 | 3.59E-09 | 3.03E-08 | Arhgap26 |
| 10590031 | 1.006628 | 5.980321 | -9.2801 | 3.60E-09 | 3.04E-08 | Itga9 |
| 10465831 | -1.73449 | 7.905137 | 9.27954 | 3.60E-09 | 3.04E-08 | 5730408K05Rik |
| 10589929 | -1.01052 | 7.940939 | 9.264456 | 3.71E-09 | 3.12E-08 | Cmtm6 |
| 10450872 | -1.32709 | 5.018903 | 9.256182 | 3.77E-09 | 3.16E-08 | Olfr99 |
| 10371271 | 1.439515 | 6.977816 | -9.25244 | 3.80E-09 | 3.18E-08 | Zfp781 |
| 10405063 | 1.72707 | 10.36459 | -9.25241 | 3.80E-09 | 3.18E-08 | Ogn |
| 10556297 | 1.335567 | 4.950037 | -9.2519 | 3.80E-09 | 3.18E-08 | Adm |
| 10475314 | 1.211882 | 6.108067 | -9.24533 | 3.85E-09 | 3.22E-08 | Map1a |
| 10497237 | -1.09113 | 5.594 | 9.24337 | 3.87E-09 | 3.23E-08 | Pag1 |
| 10461439 | 1.117309 | 7.641721 | -9.24084 | 3.89E-09 | 3.24E-08 | Fads1 |
| 10369815 | -1.16035 | 8.058038 | 9.239837 | 3.89E-09 | 3.25E-08 | Cdk1 |
| 10534889 | -1.06021 | 6.94025 | 9.237781 | 3.91E-09 | 3.26E-08 | Agfg2 |
| 10497490 | 1.183887 | 5.333153 | -9.23765 | 3.91E-09 | 3.26E-08 | Naaladl2 |
| 10571252 | -1.16183 | 3.837691 | 9.235271 | 3.93E-09 | 3.27E-08 | Tex15 |
| 10546452 | 1.032161 | 6.568972 | -9.23527 | 3.93E-09 | 3.27E-08 | Adamts9 |
| 10398354 | 2.196612 | 5.168307 | -9.22655 | 4.00E-09 | 3.33E-08 | Gm24564 |
| 10358513 | 1.102744 | 4.357421 | -9.22586 | 4.00E-09 | 3.33E-08 | Hmcn1 |
| 10382462 | -1.07506 | 7.347343 | 9.225802 | 4.00E-09 | 3.33E-08 | Slc9a3r1 |
| 10381211 | 1.231699 | 6.34225 | -9.22196 | 4.03E-09 | 3.35E-08 | Naglu |
| 10372021 | -1.18481 | 4.464134 | 9.220584 | 4.04E-09 | 3.36E-08 | Gm26122 |
| 10584595 | 1.531135 | 7.033604 | -9.21337 | 4.10E-09 | 3.40E-08 | 2610203C20Rik |
| 10531191 | 1.871041 | 6.534887 | -9.21055 | 4.12E-09 | 3.42E-08 | Adamts3 |
| 10537909 | 1.577539 | 11.26353 | -9.19961 | 4.21E-09 | 3.49E-08 | Rny3 |
| 10504670 | -1.19273 | 5.87481 | 9.198224 | 4.22E-09 | 3.50E-08 | E230008N13Rik |
| 10375360 | 1.146336 | 7.993223 | -9.197 | 4.23E-09 | 3.51E-08 | Ebf1 |
| 10371506 | -1.53441 | 6.249991 | 9.195327 | 4.25E-09 | 3.52E-08 | Stab2 |
| 10607848 | 1.166612 | 4.550827 | -9.19416 | 4.26E-09 | 3.52E-08 | Egfl6 |
| 10538706 | -1.90825 | 6.18048 | 9.175022 | 4.42E-09 | 3.64E-08 | Mmrn1 |
| 10411082 | 1.854182 | 9.065415 | -9.17188 | 4.45E-09 | 3.65E-08 | Thbs4 |
| 10494405 | -1.53073 | 10.89943 | 9.170714 | 4.46E-09 | 3.66E-08 | Hist2h3c2 |
| 10369481 | 1.052398 | 6.792594 | -9.16438 | 4.51E-09 | 3.70E-08 | H2afy2 |
| 10363173 | 1.847569 | 9.810726 | -9.16287 | 4.53E-09 | 3.71E-08 | Gja1 |
| 10514466 | 1.385781 | 7.920737 | -9.16284 | 4.53E-09 | 3.71E-08 | Jun |
| 10543791 | 1.215989 | 6.2907 | -9.15882 | 4.56E-09 | 3.74E-08 | Podxl |
| 10554013 | 1.196263 | 8.550012 | -9.15847 | 4.57E-09 | 3.74E-08 | Chsy1 |
| 10604380 | 1.000102 | 7.784674 | -9.14934 | 4.65E-09 | 3.80E-08 | Zdhhc9 |
| 10440388 | 1.006759 | 7.112302 | -9.14071 | 4.73E-09 | 3.86E-08 | Hspa13 |
| 10424607 | -1.10719 | 9.147024 | 9.137642 | 4.76E-09 | 3.88E-08 | Ptp4a3 |
| 10590631 | -1.43065 | 8.071083 | 9.133948 | 4.79E-09 | 3.91E-08 | Ccr2 |
| 10378855 | -1.28859 | 5.778771 | 9.127635 | 4.85E-09 | 3.95E-08 | Ssh2 |
| 10566326 | -1.3214 | 6.930753 | 9.125127 | 4.88E-09 | 3.97E-08 | Trim12a |
| 10461605 | -1.80258 | 5.431549 | 9.122001 | 4.91E-09 | 3.99E-08 | Ms4a4b |
| 10504692 | -1.36782 | 8.192803 | 9.118603 | 4.94E-09 | 4.02E-08 | Tmod1 |
| 10494402 | -1.47961 | 10.82915 | 9.117399 | 4.95E-09 | 4.03E-08 | Hist2h3c2 |
| 10595768 | -1.70805 | 5.410245 | 9.115663 | 4.97E-09 | 4.04E-08 | Pls1 |
| 10524790 | -1.3981 | 6.578957 | 9.114321 | 4.98E-09 | 4.05E-08 | Cit |
| 10487340 | -1.41119 | 7.002152 | 9.112615 | 5.00E-09 | 4.06E-08 | Ncaph |
| 10503186 | -1.14151 | 6.610185 | 9.11123 | 5.01E-09 | 4.07E-08 | Chd7 |
| 10453738 | 1.0252 | 5.325028 | -9.10973 | 5.03E-09 | 4.08E-08 | Fzd8 |
| 10591123 | 2.639981 | 6.651523 | -9.10973 | 5.03E-09 | 4.08E-08 | Fat3 |
| 10379652 | -1.28397 | 5.349415 | 9.093783 | 5.19E-09 | 4.20E-08 | Snord7 |
| 10503180 | -1.03383 | 4.420601 | 9.093205 | 5.19E-09 | 4.20E-08 | Chd7 |
| 10527963 | 1.642712 | 4.058989 | -9.08938 | 5.23E-09 | 4.23E-08 | Gm10484 |
| 10367982 | 1.299743 | 6.17604 | -9.08674 | 5.26E-09 | 4.25E-08 | Gpr126 |
| 10502359 | -1.48892 | 6.521496 | 9.076383 | 5.37E-09 | 4.33E-08 | Dapp1 |
| 10557992 | 1.338307 | 8.27829 | -9.07541 | 5.38E-09 | 4.33E-08 | Bag3 |
| 10445758 | -1.99622 | 5.697997 | 9.072979 | 5.40E-09 | 4.35E-08 | Treml4 |
| 10406598 | 1.262705 | 7.233713 | -9.06793 | 5.46E-09 | 4.39E-08 | Serinc5 |
| 10408928 | 1.228353 | 8.114104 | -9.06236 | 5.52E-09 | 4.44E-08 | Hspb1 |
| 10545184 | -3.70351 | 7.438116 | 9.058634 | 5.56E-09 | 4.47E-08 | Igkv4-74 |
| 10398326 | 1.843985 | 6.946 | -9.05699 | 5.58E-09 | 4.48E-08 | Meg3 |
| 10399588 | 1.240385 | 4.830798 | -9.04787 | 5.68E-09 | 4.55E-08 | Zfp125 |
| 10586454 | -1.36712 | 5.472838 | 9.046365 | 5.70E-09 | 4.56E-08 | D030028M11Rik |
| 10591118 | 2.378335 | 7.322579 | -9.04328 | 5.73E-09 | 4.59E-08 | Fat3 |
| 10351667 | -1.84964 | 4.77281 | 9.04278 | 5.74E-09 | 4.59E-08 | Slamf1 |
| 10419736 | 1.040722 | 9.77587 | -9.02922 | 5.89E-09 | 4.70E-08 | Dad1 |
| 10393728 | 1.04904 | 7.511118 | -9.02753 | 5.91E-09 | 4.72E-08 | Slc38a10 |
| 10519488 | -1.05876 | 9.606294 | 9.02558 | 5.94E-09 | 4.73E-08 | Tubb4b |
| 10369842 | 2.030916 | 8.081228 | -9.01566 | 6.05E-09 | 4.82E-08 | Bicc1 |
| 10379685 | -1.11154 | 6.055296 | 9.012765 | 6.09E-09 | 4.85E-08 | 1700020L24Rik |
| 10519886 | 2.187769 | 7.016752 | -9.01002 | 6.12E-09 | 4.87E-08 | Sema3c |
| 10401238 | 1.159958 | 8.604438 | -9.00836 | 6.14E-09 | 4.89E-08 | Zfp36l1 |
| 10419216 | 1.328307 | 7.271394 | -9.00725 | 6.15E-09 | 4.90E-08 | Gnpnat1 |
| 10369301 | -1.0413 | 4.386554 | 8.990196 | 6.37E-09 | 5.05E-08 | Chst3 |
| 10541910 | -1.48305 | 7.150083 | 8.987521 | 6.40E-09 | 5.08E-08 | Vwf |
| 10420385 | 1.138834 | 6.993585 | -8.98749 | 6.40E-09 | 5.08E-08 | N6amt2 |
| 10465833 | 1.315112 | 6.243174 | -8.98175 | 6.47E-09 | 5.13E-08 | Rom1 |
| 10403464 | 1.174984 | 4.450916 | -8.9683 | 6.65E-09 | 5.26E-08 | Dip2c |
| 10571958 | 1.002069 | 5.90664 | -8.96724 | 6.66E-09 | 5.27E-08 | Sh3rf1 |
| 10382200 | 1.030877 | 5.109298 | -8.96711 | 6.67E-09 | 5.27E-08 | Cep112 |
| 10469941 | -1.23306 | 6.168103 | 8.963455 | 6.71E-09 | 5.31E-08 | Gm22572 |
| 10427336 | -1.00943 | 8.954452 | 8.963365 | 6.72E-09 | 5.31E-08 | Nckap1l |
| 10546163 | -1.39163 | 7.303831 | 8.959878 | 6.76E-09 | 5.34E-08 | Mcm2 |
| 10432032 | 1.602218 | 7.328486 | -8.9578 | 6.79E-09 | 5.36E-08 | Vdr |
| 10591120 | 1.577845 | 6.092514 | -8.95751 | 6.79E-09 | 5.36E-08 | Fat3 |
| 10365590 | 1.057377 | 7.644886 | -8.95392 | 6.84E-09 | 5.40E-08 | Ccdc53 |
| 10459552 | -1.01122 | 6.732926 | 8.948642 | 6.92E-09 | 5.44E-08 | Spire1 |
| 10538903 | -2.2684 | 5.533844 | 8.948583 | 6.92E-09 | 5.44E-08 | Igk |
| 10548375 | -1.28616 | 6.526785 | 8.945362 | 6.96E-09 | 5.48E-08 | Clec7a |
| 10481621 | 1.011538 | 6.657798 | -8.94348 | 6.99E-09 | 5.49E-08 | 1110008P14Rik |
| 10542740 | 1.288306 | 6.947165 | -8.94079 | 7.02E-09 | 5.52E-08 | Sspn |
| 10403038 | -2.64938 | 5.637012 | 8.940746 | 7.03E-09 | 5.52E-08 | Ighv1-61 |
| 10427280 | 1.172901 | 5.744544 | -8.93736 | 7.07E-09 | 5.55E-08 | Hoxc10 |
| 10355984 | 1.077969 | 10.05398 | -8.93699 | 7.08E-09 | 5.56E-08 | Serpine2 |
| 10572772 | -1.09809 | 5.654434 | 8.929732 | 7.18E-09 | 5.63E-08 | Hsh2d |
| 10523128 | -3.45913 | 10.62797 | 8.920109 | 7.32E-09 | 5.73E-08 | Ppbp |
| 10480628 | -1.0449 | 9.812341 | 8.919917 | 7.32E-09 | 5.73E-08 | Tubb4b |
| 10413726 | 2.433225 | 7.255642 | -8.90787 | 7.50E-09 | 5.86E-08 | Tnnc1 |
| 10492971 | -1.69949 | 4.834499 | 8.904073 | 7.56E-09 | 5.90E-08 | Fcrl1 |
| 10465587 | -1.8391 | 7.478037 | 8.897477 | 7.66E-09 | 5.98E-08 | Fermt3 |
| 10476989 | -1.20281 | 5.841626 | 8.896125 | 7.68E-09 | 5.99E-08 | Gins1 |
| 10486061 | -1.15233 | 5.65808 | 8.895272 | 7.69E-09 | 6.00E-08 | Rasgrp1 |
| 10479950 | -1.09884 | 7.958929 | 8.883574 | 7.87E-09 | 6.13E-08 | Celf2 |
| 10504891 | 1.072923 | 5.289473 | -8.87655 | 7.99E-09 | 6.20E-08 | Tmeff1 |
| 10407803 | 1.496107 | 7.156131 | -8.87638 | 7.99E-09 | 6.20E-08 | Gpr137b |
| 10547404 | 1.12356 | 6.806096 | -8.87126 | 8.07E-09 | 6.26E-08 | Erc1 |
| 10482772 | 1.351446 | 6.672819 | -8.8689 | 8.11E-09 | 6.29E-08 | Nr4a2 |
| 10503196 | -1.4305 | 5.868376 | 8.857295 | 8.30E-09 | 6.43E-08 | Chd7 |
| 10387743 | -1.2554 | 7.26716 | 8.83617 | 8.66E-09 | 6.69E-08 | Slc2a4 |
| 10405587 | 1.243356 | 8.973848 | -8.83607 | 8.66E-09 | 6.69E-08 | Tgfbi |
| 10383088 | 1.327469 | 7.613424 | -8.82882 | 8.79E-09 | 6.78E-08 | Gaa |
| 10394258 | 1.060698 | 6.34007 | -8.82824 | 8.80E-09 | 6.78E-08 | Adcy3 |
| 10587508 | -1.06165 | 6.688932 | 8.816218 | 9.01E-09 | 6.94E-08 | Ttk |
| 10574682 | -1.53849 | 8.33038 | 8.794084 | 9.42E-09 | 7.23E-08 | E2f4 |
| 10427026 | 1.076258 | 5.318656 | -8.79343 | 9.44E-09 | 7.24E-08 | Grasp |
| 10526191 | 1.173964 | 6.52216 | -8.78977 | 9.51E-09 | 7.28E-08 | Gatsl2 |
| 10407072 | -1.44205 | 5.289079 | 8.789708 | 9.51E-09 | 7.28E-08 | Elovl7 |
| 10537410 | -1.45849 | 6.445423 | 8.788598 | 9.53E-09 | 7.30E-08 | Tbxas1 |
| 10512669 | -1.70695 | 5.508446 | 8.779831 | 9.70E-09 | 7.42E-08 | Pax5 |
| 10528207 | -1.21881 | 9.247694 | 8.770915 | 9.87E-09 | 7.54E-08 | Cd36 |
| 10553057 | 1.086556 | 5.946076 | -8.76944 | 9.90E-09 | 7.56E-08 | Mamstr |
| 10350247 | -1.34614 | 6.242919 | 8.768521 | 9.92E-09 | 7.57E-08 | Kif21b |
| 10400023 | -1.22345 | 7.690025 | 8.758753 | 1.01E-08 | 7.71E-08 | Tspan13 |
| 10413086 | 1.090126 | 8.32136 | -8.75818 | 1.01E-08 | 7.72E-08 | Adk |
| 10529584 | 1.005047 | 7.599117 | -8.75384 | 1.02E-08 | 7.78E-08 | Man2b2 |
| 10371176 | 1.368651 | 8.19797 | -8.75021 | 1.03E-08 | 7.83E-08 | Nfic |
| 10476939 | 1.696093 | 7.963248 | -8.74996 | 1.03E-08 | 7.83E-08 | Zfp937 |
| 10424582 | 1.302162 | 4.182165 | -8.74624 | 1.04E-08 | 7.89E-08 | Gm24787 |
| 10591110 | 1.630443 | 6.264681 | -8.74468 | 1.04E-08 | 7.91E-08 | Fat3 |
| 10366153 | 1.151002 | 4.453652 | -8.74419 | 1.04E-08 | 7.91E-08 | Rassf9 |
| 10556598 | 1.393889 | 7.371877 | -8.74191 | 1.05E-08 | 7.95E-08 | Xylt1 |
| 10559446 | -1.51454 | 8.477995 | 8.73966 | 1.05E-08 | 7.98E-08 | Pirb |
| 10359890 | -1.29878 | 7.472552 | 8.737608 | 1.06E-08 | 8.01E-08 | Nuf2 |
| 10498568 | 1.21438 | 5.733681 | -8.73135 | 1.07E-08 | 8.11E-08 | Shox2 |
| 10587799 | 1.253263 | 5.095175 | -8.73106 | 1.07E-08 | 8.11E-08 | Plscr2 |
| 10420274 | 2.606333 | 4.569536 | -8.72692 | 1.08E-08 | 8.17E-08 | Gzmd |
| 10565958 | 1.172949 | 6.863245 | -8.7098 | 1.12E-08 | 8.45E-08 | P2ry6 |
| 10515431 | -1.07376 | 6.837182 | 8.709259 | 1.12E-08 | 8.46E-08 | Kif2c |
| 10431872 | -1.18008 | 4.352376 | 8.696947 | 1.15E-08 | 8.66E-08 | Slc38a1 |
| 10350146 | 1.257658 | 8.187535 | -8.68482 | 1.18E-08 | 8.86E-08 | Phlda3 |
| 10576757 | -2.30213 | 4.748886 | 8.684387 | 1.18E-08 | 8.87E-08 | Fcer2a |
| 10466624 | -1.42568 | 4.906436 | 8.684006 | 1.18E-08 | 8.87E-08 | Aldh1a7 |
| 10526410 | 1.196275 | 7.952262 | -8.67562 | 1.20E-08 | 9.02E-08 | Hspb1 |
| 10452709 | -1.07122 | 7.755196 | 8.670276 | 1.21E-08 | 9.11E-08 | Ndc80 |
| 10391103 | 1.371861 | 7.712907 | -8.66997 | 1.21E-08 | 9.11E-08 | Jup |
| 10538590 | -1.23068 | 6.281843 | 8.668074 | 1.22E-08 | 9.14E-08 | Herc6 |
| 10450145 | -1.33414 | 8.392998 | 8.66647 | 1.22E-08 | 9.17E-08 | Psmb9 |
| 10437945 | -1.10656 | 7.244821 | 8.661797 | 1.23E-08 | 9.25E-08 | Mcm4 |
| 10437151 | 1.127613 | 5.115696 | -8.65368 | 1.25E-08 | 9.39E-08 | Kcnj15 |
| 10420426 | -1.17333 | 5.848881 | 8.645313 | 1.27E-08 | 9.54E-08 | Ska3 |
| 10398366 | 2.486137 | 5.93752 | -8.64293 | 1.28E-08 | 9.58E-08 | Gm25357 |
| 10603440 | -1.14301 | 6.670683 | 8.641658 | 1.28E-08 | 9.60E-08 | Was |
| 10358569 | 1.266746 | 5.532613 | -8.64136 | 1.28E-08 | 9.60E-08 | Hmcn1 |
| 10485607 | 1.020629 | 8.067399 | -8.63719 | 1.29E-08 | 9.68E-08 | Qser1 |
| 10548409 | -1.81684 | 4.704573 | 8.624767 | 1.33E-08 | 9.90E-08 | Klrc1 |
| 10518686 | -1.30036 | 6.948164 | 8.620857 | 1.34E-08 | 9.97E-08 | Pik3cd |
| 10515755 | -1.88951 | 6.328377 | 8.61737 | 1.35E-08 | 1.00E-07 | Mpl |
| 10452419 | 1.207083 | 6.318625 | -8.61465 | 1.36E-08 | 1.01E-07 | Efna5 |
| 10545231 | -3.23858 | 5.878326 | 8.613248 | 1.36E-08 | 1.01E-07 | Igkv6-32 |
| 10521863 | 1.040184 | 8.103924 | -8.61085 | 1.37E-08 | 1.02E-07 | Anapc4 |
| 10348299 | -1.40244 | 4.99398 | 8.607944 | 1.37E-08 | 1.02E-07 | 5830472F04Rik |
| 10540298 | 1.599788 | 4.965462 | -8.60633 | 1.38E-08 | 1.02E-07 | Chl1 |
| 10522749 | 1.287204 | 4.478794 | -8.59792 | 1.40E-08 | 1.04E-07 | Lphn3 |
| 10583021 | 1.514161 | 6.907534 | -8.59463 | 1.41E-08 | 1.05E-07 | Pdgfd |
| 10545196 | -3.10716 | 7.632824 | 8.59302 | 1.42E-08 | 1.05E-07 | Igkv4-61 |
| 10551025 | -2.62921 | 5.588256 | 8.589382 | 1.43E-08 | 1.06E-07 | Cd79a |
| 10497349 | -1.30523 | 7.696146 | 8.587096 | 1.43E-08 | 1.06E-07 | Sirpb1a |
| 10542340 | -1.00295 | 6.690754 | 8.586374 | 1.44E-08 | 1.06E-07 | 8430419L09Rik |
| 10512308 | 1.231861 | 6.697274 | -8.58555 | 1.44E-08 | 1.07E-07 | Sigmar1 |
| 10396402 | 1.106699 | 6.652319 | -8.58217 | 1.45E-08 | 1.07E-07 | Prkch |
| 10519693 | 1.700537 | 5.739811 | -8.58001 | 1.45E-08 | 1.08E-07 | Sema3d |
| 10420957 | -1.25577 | 6.990748 | 8.579041 | 1.46E-08 | 1.08E-07 | Ptk2b |
| 10595791 | -1.06524 | 9.03533 | 8.577151 | 1.46E-08 | 1.08E-07 | Nyx |
| 10573319 | 1.335283 | 5.866789 | -8.57431 | 1.47E-08 | 1.09E-07 | Podnl1 |
| 10408689 | 2.173224 | 5.386717 | -8.5675 | 1.49E-08 | 1.10E-07 | Nrn1 |
| 10501164 | 1.036739 | 7.259857 | -8.5668 | 1.49E-08 | 1.10E-07 | Csf1 |
| 10595480 | 1.515135 | 7.648448 | -8.56599 | 1.50E-08 | 1.10E-07 | Me1 |
| 10497364 | -1.36607 | 7.67475 | 8.562754 | 1.51E-08 | 1.11E-07 | Sirpb1a |
| 10379957 | 1.134399 | 7.215537 | -8.56135 | 1.51E-08 | 1.11E-07 | Rnft1 |
| 10391811 | -1.77394 | 6.973476 | 8.550573 | 1.54E-08 | 1.14E-07 | Kif18b |
| 10591643 | -1.10662 | 6.166179 | 8.550299 | 1.55E-08 | 1.14E-07 | Rab3d |
| 10427628 | -1.97733 | 5.859937 | 8.548087 | 1.55E-08 | 1.14E-07 | Il7r |
| 10478949 | 1.030316 | 6.177026 | -8.54782 | 1.55E-08 | 1.14E-07 | Dok5 |
| 10539818 | -2.72429 | 7.515412 | 8.543973 | 1.57E-08 | 1.15E-07 | Gp9 |
| 10545187 | -3.6957 | 6.63807 | 8.535521 | 1.59E-08 | 1.17E-07 | Igkv4-70 |
| 10377405 | -1.53163 | 7.659941 | 8.527291 | 1.62E-08 | 1.19E-07 | Aurkb |
| 10416887 | -1.04054 | 4.961695 | 8.526265 | 1.62E-08 | 1.19E-07 | Slain1 |
| 10598456 | -1.23726 | 8.10662 | 8.525457 | 1.63E-08 | 1.19E-07 | Otud5 |
| 10411059 | 1.042088 | 6.220803 | -8.52296 | 1.63E-08 | 1.20E-07 | Zfyve16 |
| 10379518 | 2.331923 | 6.865245 | -8.52271 | 1.63E-08 | 1.20E-07 | Ccl7 |
| 10469255 | -1.09827 | 6.921402 | 8.515135 | 1.66E-08 | 1.21E-07 | Prkcq |
| 10405047 | 1.832 | 10.9534 | -8.51353 | 1.67E-08 | 1.22E-07 | Aspn |
| 10519607 | -1.23312 | 7.90365 | 8.504572 | 1.70E-08 | 1.24E-07 | Tmem243 |
| 10397364 | 1.103558 | 5.649869 | -8.49373 | 1.73E-08 | 1.26E-07 | Mfsd7c |
| 10475643 | 1.649901 | 7.4918 | -8.4899 | 1.75E-08 | 1.27E-07 | Fgf7 |
| 10471424 | 1.474665 | 7.40999 | -8.48591 | 1.76E-08 | 1.28E-07 | Fam102a |
| 10485622 | 1.244265 | 8.488194 | -8.4758 | 1.80E-08 | 1.31E-07 | Qser1 |
| 10533345 | -1.18416 | 8.069725 | 8.472034 | 1.81E-08 | 1.32E-07 | Aldh2 |
| 10571384 | 1.045064 | 5.503339 | -8.46865 | 1.83E-08 | 1.32E-07 | Micu3 |
| 10346695 | 1.117189 | 7.241506 | -8.46645 | 1.83E-08 | 1.33E-07 | Nbeal1 |
| 10400941 | -1.06725 | 7.207056 | 8.465628 | 1.84E-08 | 1.33E-07 | Dhrs7 |
| 10536635 | 1.164179 | 7.838687 | -8.46228 | 1.85E-08 | 1.34E-07 | Cped1 |
| 10365344 | -1.03711 | 8.010295 | 8.460788 | 1.86E-08 | 1.34E-07 | Tcp11l2 |
| 10535006 | -1.13537 | 7.05015 | 8.459537 | 1.86E-08 | 1.35E-07 | BC037034 |
| 10361292 | -1.35889 | 4.653345 | 8.456207 | 1.87E-08 | 1.35E-07 | Cr2 |
| 10471953 | 1.135824 | 7.1415 | -8.45391 | 1.88E-08 | 1.36E-07 | Acvr2a |
| 10376459 | -1.00572 | 6.315268 | 8.439994 | 1.94E-08 | 1.40E-07 | Gm12260 |
| 10588786 | -1.32259 | 6.19432 | 8.439138 | 1.94E-08 | 1.40E-07 | Uba7 |
| 10513805 | 1.006348 | 4.87749 | -8.43805 | 1.95E-08 | 1.40E-07 | Brinp1 |
| 10395692 | 1.470773 | 6.562068 | -8.43586 | 1.95E-08 | 1.41E-07 | Arhgap5 |
| 10358599 | 1.225611 | 5.991082 | -8.43475 | 1.96E-08 | 1.41E-07 | Hmcn1 |
| 10358553 | 1.069874 | 4.223739 | -8.43308 | 1.97E-08 | 1.41E-07 | Hmcn1 |
| 10551696 | -1.81658 | 7.070935 | 8.431757 | 1.97E-08 | 1.42E-07 | Rasgrp4 |
| 10558880 | 1.43256 | 5.787688 | -8.4232 | 2.01E-08 | 1.44E-07 | Eps8l2 |
| 10522530 | -1.16275 | 6.506419 | 8.418342 | 2.03E-08 | 1.45E-07 | Kit |
| 10363070 | -1.316 | 8.844554 | 8.416233 | 2.03E-08 | 1.46E-07 | Gp49a |
| 10351873 | -1.96471 | 6.198537 | 8.410215 | 2.06E-08 | 1.47E-07 | Pyhin1 |
| 10447946 | 1.027599 | 5.724759 | -8.40926 | 2.06E-08 | 1.48E-07 | 4930474M22Rik |
| 10500333 | -1.18142 | 10.47867 | 8.405258 | 2.08E-08 | 1.49E-07 | Hist2h4 |
| 10538892 | -1.29984 | 4.52837 | 8.402157 | 2.09E-08 | 1.49E-07 | Env |
| 10565570 | -1.17564 | 5.455744 | 8.396816 | 2.12E-08 | 1.51E-07 | Ddias |
| 10589889 | 1.334717 | 7.453117 | -8.3927 | 2.14E-08 | 1.52E-07 | Glb1 |
| 10416736 | -1.1871 | 6.718758 | 8.389213 | 2.15E-08 | 1.53E-07 | Bora |
| 10429564 | 1.370616 | 9.370046 | -8.3887 | 2.15E-08 | 1.53E-07 | Ly6a |
| 10556553 | 1.263555 | 5.817816 | -8.38676 | 2.16E-08 | 1.54E-07 | Insc |
| 10503695 | -1.05917 | 4.974061 | 8.38532 | 2.17E-08 | 1.54E-07 | Bach2 |
| 10545198 | -3.68147 | 7.436981 | 8.38464 | 2.17E-08 | 1.54E-07 | Igkv4-59 |
| 10594798 | -1.97817 | 6.001776 | 8.369814 | 2.24E-08 | 1.58E-07 | Gm23730 |
| 10473880 | 1.210901 | 7.124646 | -8.36373 | 2.27E-08 | 1.60E-07 | Lrp4 |
| 10389222 | -1.46151 | 8.679671 | 8.358385 | 2.29E-08 | 1.61E-07 | Ccl6 |
| 10500685 | 1.026751 | 9.389998 | -8.35746 | 2.30E-08 | 1.62E-07 | Atp1a1 |
| 10558903 | -1.19665 | 8.275793 | 8.355014 | 2.31E-08 | 1.62E-07 | Taldo1 |
| 10531972 | -1.01508 | 4.677662 | 8.349478 | 2.34E-08 | 1.64E-07 | Gbp8 |
| 10358563 | 1.746749 | 5.733966 | -8.34756 | 2.35E-08 | 1.65E-07 | Hmcn1 |
| 10468893 | -1.14688 | 7.978232 | 8.347012 | 2.35E-08 | 1.65E-07 | Csf2ra |
| 10358571 | 1.159296 | 4.464922 | -8.34592 | 2.35E-08 | 1.65E-07 | Hmcn1 |
| 10458382 | 1.185287 | 6.04858 | -8.34336 | 2.37E-08 | 1.66E-07 | Cd14 |
| 10594582 | -1.33865 | 5.161353 | 8.341352 | 2.38E-08 | 1.67E-07 | Snx22 |
| 10348451 | 1.241805 | 6.602085 | -8.33948 | 2.38E-08 | 1.67E-07 | Ackr3 |
| 10430645 | -1.16032 | 4.801796 | 8.335831 | 2.40E-08 | 1.68E-07 | D730005E14Rik |
| 10380403 | 1.092628 | 8.385114 | -8.32646 | 2.45E-08 | 1.71E-07 | Lrrc59 |
| 10562132 | -1.71178 | 5.833844 | 8.319677 | 2.48E-08 | 1.74E-07 | Cd22 |
| 10591131 | 1.855691 | 5.733636 | -8.31641 | 2.50E-08 | 1.75E-07 | Fat3 |
| 10583809 | 1.400078 | 5.427214 | -8.31374 | 2.52E-08 | 1.75E-07 | Cnn1 |
| 10403871 | -1.03741 | 6.849886 | 8.306448 | 2.55E-08 | 1.78E-07 | Aoah |
| 10502805 | 1.482022 | 6.370826 | -8.30386 | 2.57E-08 | 1.79E-07 | Ptgfr |
| 10365983 | 2.199775 | 10.65693 | -8.30153 | 2.58E-08 | 1.80E-07 | Lum |
| 10557156 | -1.30169 | 7.155909 | 8.295084 | 2.61E-08 | 1.82E-07 | Plk1 |
| 10403756 | 1.067799 | 7.568797 | -8.28521 | 2.67E-08 | 1.85E-07 | Yae1d1 |
| 10407916 | 1.067799 | 7.568797 | -8.28521 | 2.67E-08 | 1.85E-07 | Yae1d1 |
| 10489878 | 1.524158 | 7.731432 | -8.26885 | 2.76E-08 | 1.91E-07 | Ptgis |
| 10545190 | -3.65276 | 5.281339 | 8.266162 | 2.78E-08 | 1.92E-07 | Igkv4-69 |
| 10583008 | 1.675882 | 6.217007 | -8.26426 | 2.79E-08 | 1.92E-07 | Casp12 |
| 10444821 | -1.07368 | 5.916546 | 8.263821 | 2.79E-08 | 1.92E-07 | H2-Q5 |
| 10461214 | 1.055877 | 8.931588 | -8.26124 | 2.81E-08 | 1.93E-07 | Tmem223 |
| 10588495 | 1.0902 | 7.765874 | -8.26034 | 2.81E-08 | 1.94E-07 | Dusp7 |
| 10467110 | 1.103661 | 5.984675 | -8.25344 | 2.85E-08 | 1.96E-07 | Lipo1 |
| 10385526 | -1.05948 | 7.219614 | 8.251125 | 2.87E-08 | 1.97E-07 | 9930111J21Rik1 |
| 10403015 | -2.83553 | 6.52531 | 8.250347 | 2.87E-08 | 1.97E-07 | Ighv1-18 |
| 10404061 | -2.10506 | 7.881904 | 8.24455 | 2.90E-08 | 1.99E-07 | Hist1h2bb |
| 10444236 | -1.32742 | 6.836665 | 8.238356 | 2.94E-08 | 2.02E-07 | H2-DMb2 |
| 10471912 | -1.10585 | 4.124415 | 8.230053 | 2.99E-08 | 2.05E-07 | Kynu |
| 10493812 | 1.136745 | 10.60836 | -8.22188 | 3.05E-08 | 2.08E-07 | S100a4 |
| 10461160 | -1.21167 | 5.503051 | 8.221358 | 3.05E-08 | 2.08E-07 | Gm23246 |
| 10430510 | 1.528562 | 7.262229 | -8.21828 | 3.07E-08 | 2.10E-07 | Tmem184b |
| 10584317 | 1.066647 | 5.905391 | -8.21794 | 3.07E-08 | 2.10E-07 | Esam |
| 10484463 | 1.01909 | 10.51538 | -8.21269 | 3.10E-08 | 2.12E-07 | Serping1 |
| 10585390 | 1.169697 | 6.216315 | -8.20526 | 3.15E-08 | 2.15E-07 | Sln |
| 10384811 | 1.007555 | 7.929609 | -8.20202 | 3.17E-08 | 2.16E-07 | Ccdc104 |
| 10395702 | 1.206088 | 5.672935 | -8.20197 | 3.17E-08 | 2.16E-07 | Akap6 |
| 10495206 | 1.091077 | 6.719791 | -8.20043 | 3.19E-08 | 2.17E-07 | Slc16a4 |
| 10585974 | 1.553871 | 6.672932 | -8.19748 | 3.20E-08 | 2.18E-07 | Myo9a |
| 10503198 | -1.01936 | 5.06904 | 8.187566 | 3.27E-08 | 2.22E-07 | Chd7 |
| 10428707 | 1.938511 | 5.808659 | -8.18276 | 3.31E-08 | 2.24E-07 | Has2 |
| 10425333 | -1.19389 | 6.829965 | 8.174139 | 3.37E-08 | 2.27E-07 | Apobec3 |
| 10427590 | 1.444541 | 5.877639 | -8.17288 | 3.37E-08 | 2.28E-07 | Slc1a3 |
| 10430679 | -1.64033 | 4.77123 | 8.166786 | 3.42E-08 | 2.31E-07 | Gm24204 |
| 10358660 | 1.718563 | 4.748662 | -8.16662 | 3.42E-08 | 2.31E-07 | Hmcn1 |
| 10395273 | -1.06817 | 6.167667 | 8.164067 | 3.44E-08 | 2.32E-07 | Gdap10 |
| 10594053 | -1.00026 | 7.146202 | 8.160963 | 3.46E-08 | 2.33E-07 | Pml |
| 10385513 | -1.04647 | 7.2347 | 8.16062 | 3.46E-08 | 2.33E-07 | 9930111J21Rik2 |
| 10455135 | 1.083718 | 3.924292 | -8.15992 | 3.47E-08 | 2.33E-07 | Pcdhb21 |
| 10579049 | 1.602323 | 6.188629 | -8.15619 | 3.49E-08 | 2.35E-07 | Gm10033 |
| 10496971 | -1.17188 | 3.627772 | 8.15224 | 3.52E-08 | 2.37E-07 | Asb17 |
| 10347919 | -1.3155 | 5.45983 | 8.147858 | 3.56E-08 | 2.39E-07 | LOC102634459 |
| 10356291 | -1.3155 | 5.45983 | 8.147858 | 3.56E-08 | 2.39E-07 | LOC102634459 |
| 10428081 | -1.3114 | 6.074807 | 8.143645 | 3.59E-08 | 2.41E-07 | Hrsp12 |
| 10364916 | -1.05937 | 7.344489 | 8.143479 | 3.59E-08 | 2.41E-07 | Sppl2b |
| 10380566 | 1.575302 | 7.534683 | -8.13882 | 3.62E-08 | 2.43E-07 | Phospho1 |
| 10352048 | -1.00913 | 5.839987 | 8.135108 | 3.65E-08 | 2.44E-07 | Exo1 |
| 10346365 | -1.05861 | 7.587466 | 8.134123 | 3.66E-08 | 2.45E-07 | Sgol2 |
| 10533213 | -1.81177 | 5.762607 | 8.131568 | 3.68E-08 | 2.46E-07 | Oas3 |
| 10582860 | -1.26036 | 5.39982 | 8.129336 | 3.70E-08 | 2.47E-07 | LOC102634459 |
| 10582877 | -1.26036 | 5.39982 | 8.129336 | 3.70E-08 | 2.47E-07 | LOC102634459 |
| 10380419 | 1.992595 | 10.84808 | -8.12385 | 3.74E-08 | 2.49E-07 | Col1a1 |
| 10396936 | 1.300859 | 5.813364 | -8.12302 | 3.75E-08 | 2.50E-07 | Smoc1 |
| 10398360 | 2.376149 | 4.956997 | -8.11994 | 3.77E-08 | 2.51E-07 | Gm23508 |
| 10462632 | -1.2062 | 6.80446 | 8.116002 | 3.80E-08 | 2.53E-07 | Kif20b |
| 10386236 | -1.04448 | 5.377589 | 8.114819 | 3.81E-08 | 2.54E-07 | Hist3h2bb-ps |
| 10403948 | -1.01136 | 11.35445 | 8.113148 | 3.82E-08 | 2.55E-07 | Hist1h2bn |
| 10463263 | 1.33774 | 7.289669 | -8.10362 | 3.90E-08 | 2.59E-07 | Lztfl1 |
| 10518927 | -1.01506 | 6.771298 | 8.092736 | 3.99E-08 | 2.65E-07 | Kcnab2 |
| 10369647 | 1.018799 | 7.600012 | -8.08865 | 4.03E-08 | 2.67E-07 | Ddx50 |
| 10371159 | -1.21821 | 8.017397 | 8.085901 | 4.05E-08 | 2.68E-07 | Fzr1 |
| 10358619 | 1.50252 | 6.14563 | -8.08551 | 4.05E-08 | 2.69E-07 | Hmcn1 |
| 10497356 | -1.32276 | 7.548949 | 8.083329 | 4.07E-08 | 2.70E-07 | Sirpb1a |
| 10538882 | -2.5919 | 5.14401 | 8.083318 | 4.07E-08 | 2.70E-07 | Igkv9-124 |
| 10536220 | 1.939815 | 10.89225 | -8.0832 | 4.07E-08 | 2.70E-07 | Col1a2 |
| 10471247 | 1.168415 | 5.701608 | -8.08292 | 4.08E-08 | 2.70E-07 | Aif1l |
| 10512827 | -1.07544 | 4.628992 | 8.081585 | 4.09E-08 | 2.70E-07 | Gm568 |
| 10356269 | -1.16234 | 7.801339 | 8.08085 | 4.09E-08 | 2.71E-07 | LOC100041708 |
| 10416566 | -1.02727 | 6.764889 | 8.078749 | 4.11E-08 | 2.72E-07 | Epsti1 |
| 10604832 | -1.1543 | 6.251782 | 8.078658 | 4.11E-08 | 2.72E-07 | Mir505 |
| 10534960 | 1.42308 | 4.282037 | -8.07716 | 4.13E-08 | 2.72E-07 | Gjc3 |
| 10586448 | -1.13707 | 7.026079 | 8.075156 | 4.14E-08 | 2.74E-07 | 2810417H13Rik |
| 10472549 | 1.189347 | 5.416337 | -8.06651 | 4.22E-08 | 2.78E-07 | Bbs5 |
| 10520388 | -1.29803 | 7.502801 | 8.061607 | 4.26E-08 | 2.81E-07 | Rbm33 |
| 10422052 | 1.008718 | 7.250076 | -8.02974 | 4.56E-08 | 2.99E-07 | Commd6 |
| 10581605 | -1.30366 | 10.12749 | 8.029473 | 4.56E-08 | 2.99E-07 | Hp |
| 10591090 | 1.60204 | 6.655959 | -8.02683 | 4.59E-08 | 3.00E-07 | Fat3 |
| 10358595 | 1.113629 | 4.700787 | -8.02643 | 4.59E-08 | 3.00E-07 | Hmcn1 |
| 10607952 | 1.292684 | 7.891858 | -8.02525 | 4.60E-08 | 3.01E-07 | Vamp7 |
| 10559399 | 1.709004 | 6.113074 | -8.02247 | 4.63E-08 | 3.03E-07 | Oscar |
| 10584710 | -1.65829 | 9.080526 | 8.014515 | 4.71E-08 | 3.07E-07 | H2afx |
| 10388880 | 1.119636 | 7.187427 | -8.01442 | 4.71E-08 | 3.07E-07 | Tmem97 |
| 10373918 | 1.445235 | 5.334484 | -8.01426 | 4.71E-08 | 3.07E-07 | Lif |
| 10479221 | 1.510184 | 6.957497 | -7.99597 | 4.90E-08 | 3.18E-07 | Gm14403 |
| 10365987 | 2.162553 | 6.241539 | -7.99223 | 4.94E-08 | 3.21E-07 | Kera |
| 10586017 | 1.282531 | 8.198814 | -7.98149 | 5.05E-08 | 3.27E-07 | Uaca |
| 10575616 | -1.11792 | 6.274031 | 7.980563 | 5.06E-08 | 3.28E-07 | Gabarapl2 |
| 10592266 | 1.559145 | 6.950205 | -7.97903 | 5.08E-08 | 3.29E-07 | Slc37a2 |
| 10405753 | 1.587381 | 7.553286 | -7.97487 | 5.12E-08 | 3.31E-07 | Me1 |
| 10591094 | 1.317738 | 5.990636 | -7.97422 | 5.13E-08 | 3.32E-07 | Fat3 |
| 10506488 | 1.212143 | 8.326459 | -7.96331 | 5.25E-08 | 3.39E-07 | Ppap2b |
| 10493382 | -1.3722 | 5.03598 | 7.95879 | 5.30E-08 | 3.42E-07 | Pklr |
| 10498367 | -1.16763 | 6.773406 | 7.958705 | 5.30E-08 | 3.42E-07 | P2ry13 |
| 10410477 | 1.2594 | 4.68175 | -7.95229 | 5.37E-08 | 3.46E-07 | Adamts16 |
| 10604897 | -1.22858 | 4.775017 | 7.951979 | 5.38E-08 | 3.46E-07 | Gm22435 |
| 10579052 | 1.436692 | 5.946261 | -7.95046 | 5.39E-08 | 3.47E-07 | Gm10033 |
| 10579525 | 1.165791 | 7.213043 | -7.94238 | 5.49E-08 | 3.53E-07 | Plvap |
| 10592420 | 1.282849 | 4.662857 | -7.93539 | 5.57E-08 | 3.58E-07 | AW551984 |
| 10352867 | 1.054588 | 6.707875 | -7.9352 | 5.57E-08 | 3.58E-07 | Plxna2 |
| 10355325 | -1.48148 | 6.224322 | 7.93265 | 5.60E-08 | 3.59E-07 | Bard1 |
| 10554599 | 1.293916 | 5.097679 | -7.92892 | 5.65E-08 | 3.62E-07 | Adamtsl3 |
| 10580210 | -1.24077 | 8.381423 | 7.9289 | 5.65E-08 | 3.62E-07 | Rad23a |
| 10463476 | 1.998989 | 6.413759 | -7.92309 | 5.72E-08 | 3.66E-07 | Kazald1 |
| 10386058 | 1.082385 | 12.04668 | -7.92242 | 5.73E-08 | 3.66E-07 | Sparc |
| 10439058 | -1.16169 | 6.904394 | 7.919351 | 5.76E-08 | 3.69E-07 | Nrros |
| 10379535 | 3.184707 | 6.957698 | -7.9095 | 5.88E-08 | 3.75E-07 | Ccl8 |
| 10541605 | 1.934609 | 5.896031 | -7.90305 | 5.97E-08 | 3.80E-07 | Clec4n |
| 10438753 | 1.242913 | 6.965578 | -7.90072 | 6.00E-08 | 3.82E-07 | Leprel1 |
| 10359201 | -1.18584 | 6.538451 | 7.899909 | 6.01E-08 | 3.82E-07 | Ralgps2 |
| 10559649 | -1.65883 | 7.008756 | 7.885161 | 6.20E-08 | 3.94E-07 | Cox6b2 |
| 10379176 | -1.3951 | 6.262845 | 7.870232 | 6.40E-08 | 4.06E-07 | Unc119 |
| 10374400 | -1.05822 | 4.545454 | 7.865448 | 6.46E-08 | 4.10E-07 | Fbxo48 |
| 10435185 | -1.4826 | 6.754125 | 7.858576 | 6.56E-08 | 4.15E-07 | n-R5s33 |
| 10523923 | -1.02101 | 5.128628 | 7.857191 | 6.58E-08 | 4.16E-07 | Ccdc18 |
| 10444152 | 1.436148 | 7.191546 | -7.85092 | 6.67E-08 | 4.22E-07 | Col11a2 |
| 10413609 | 1.414823 | 9.690442 | -7.84716 | 6.72E-08 | 4.25E-07 | Mustn1 |
| 10576556 | -1.09166 | 8.262703 | 7.840465 | 6.82E-08 | 4.30E-07 | 4930566D17Rik |
| 10479423 | 1.702712 | 7.004745 | -7.8381 | 6.85E-08 | 4.32E-07 | Col9a3 |
| 10592515 | -1.72145 | 6.68289 | 7.836723 | 6.87E-08 | 4.33E-07 | Ubash3b |
| 10533504 | 1.034943 | 5.3124 | -7.83663 | 6.88E-08 | 4.33E-07 | Ift81 |
| 10541491 | -1.14281 | 4.25408 | 7.832858 | 6.93E-08 | 4.37E-07 | 1700063H04Rik |
| 10385466 | 1.293107 | 7.395402 | -7.82592 | 7.03E-08 | 4.42E-07 | Sgcd |
| 10604694 | 1.16561 | 5.489819 | -7.82391 | 7.06E-08 | 4.44E-07 | Mtap7d3 |
| 10548345 | -1.33507 | 4.748471 | 7.821099 | 7.11E-08 | 4.46E-07 | Klrk1 |
| 10445977 | -1.44005 | 6.744996 | 7.820116 | 7.12E-08 | 4.47E-07 | Ebi3 |
| 10494672 | 1.446522 | 7.150001 | -7.81915 | 7.14E-08 | 4.48E-07 | Tbx15 |
| 10568586 | -1.21556 | 7.275039 | 7.810262 | 7.27E-08 | 4.55E-07 | Fam53b |
| 10544801 | 1.161789 | 6.121118 | -7.80897 | 7.29E-08 | 4.56E-07 | Hoxa11 |
| 10350506 | 2.175374 | 5.45644 | -7.78468 | 7.68E-08 | 4.79E-07 | Brinp3 |
| 10406530 | 1.050799 | 8.120974 | -7.78438 | 7.69E-08 | 4.79E-07 | Tmem167 |
| 10467191 | 1.369306 | 5.935177 | -7.77928 | 7.77E-08 | 4.84E-07 | Ankrd1 |
| 10532669 | 1.043908 | 6.089228 | -7.77257 | 7.89E-08 | 4.91E-07 | 2900026A02Rik |
| 10557960 | 1.437054 | 8.299541 | -7.76509 | 8.01E-08 | 4.98E-07 | Tgfb1i1 |
| 10427125 | 1.257459 | 8.266758 | -7.75879 | 8.12E-08 | 5.04E-07 | Igfbp6 |
| 10594426 | -1.22156 | 5.684681 | 7.754764 | 8.19E-08 | 5.08E-07 | Zwilch |
| 10485370 | -1.40984 | 3.957403 | 7.743731 | 8.39E-08 | 5.19E-07 | B230118H07Rik |
| 10545194 | -2.31951 | 5.120389 | 7.740081 | 8.46E-08 | 5.23E-07 | Igkv4-62 |
| 10382973 | -1.37789 | 5.836411 | 7.72634 | 8.71E-08 | 5.37E-07 | 6030468B19Rik |
| 10379530 | 1.532924 | 5.977119 | -7.71724 | 8.88E-08 | 5.47E-07 | Ccl12 |
| 10507099 | 1.019171 | 5.610326 | -7.71702 | 8.89E-08 | 5.47E-07 | Trabd2b |
| 10559547 | 1.32501 | 7.522891 | -7.71452 | 8.94E-08 | 5.50E-07 | Tnnt1 |
| 10543058 | 1.496411 | 7.83459 | -7.7107 | 9.01E-08 | 5.54E-07 | Dlx5 |
| 10500677 | -1.90283 | 5.753029 | 7.699587 | 9.23E-08 | 5.66E-07 | Cd2 |
| 10345752 | -1.50693 | 5.752969 | 7.694951 | 9.32E-08 | 5.72E-07 | Il1r2 |
| 10430931 | -1.49165 | 6.842905 | 7.692213 | 9.38E-08 | 5.75E-07 | Nfam1 |
| 10545247 | -3.38312 | 5.889114 | 7.690858 | 9.40E-08 | 5.76E-07 | Igkv6-14 |
| 10439483 | 1.119961 | 7.704529 | -7.68167 | 9.59E-08 | 5.86E-07 | Arhgap31 |
| 10591116 | 1.885132 | 7.07516 | -7.67369 | 9.76E-08 | 5.95E-07 | Fat3 |
| 10407792 | 1.17702 | 8.217997 | -7.66839 | 9.87E-08 | 6.02E-07 | Gpr137b-ps |
| 10536845 | 1.44225 | 7.180309 | -7.66359 | 9.98E-08 | 6.08E-07 | Flnc |
| 10501063 | -1.35282 | 9.80218 | 7.644849 | 1.04E-07 | 6.31E-07 | Cd53 |
| 10468762 | 1.223994 | 6.404143 | -7.64462 | 1.04E-07 | 6.31E-07 | 4930506M07Rik |
| 10591092 | 1.592567 | 5.504394 | -7.64462 | 1.04E-07 | 6.31E-07 | Fat3 |
| 10364361 | -1.08781 | 5.859852 | 7.643347 | 1.04E-07 | 6.32E-07 | Icosl |
| 10438708 | 1.023045 | 4.799403 | -7.63955 | 1.05E-07 | 6.37E-07 | Masp1 |
| 10511779 | 1.933795 | 8.609073 | -7.63891 | 1.05E-07 | 6.38E-07 | Atp6v0d2 |
| 10601424 | -1.35578 | 4.033306 | 7.638532 | 1.05E-07 | 6.38E-07 | Gpr174 |
| 10591129 | 1.938923 | 7.186175 | -7.63651 | 1.06E-07 | 6.41E-07 | Fat3 |
| 10459854 | -1.00253 | 4.695022 | 7.635954 | 1.06E-07 | 6.41E-07 | F830208F22Rik |
| 10497381 | 1.061599 | 4.989559 | -7.6356 | 1.06E-07 | 6.41E-07 | Cyp7b1 |
| 10444824 | -1.90264 | 7.211918 | 7.627927 | 1.08E-07 | 6.51E-07 | H2-Q8 |
| 10575255 | -1.08381 | 5.335049 | 7.627022 | 1.08E-07 | 6.52E-07 | Pkd1l3 |
| 10403069 | -2.40185 | 4.511353 | 7.62071 | 1.09E-07 | 6.60E-07 | Igh-VJ558 |
| 10472400 | 1.200269 | 4.67374 | -7.6126 | 1.11E-07 | 6.71E-07 | Scn2a1 |
| 10351224 | -1.5007 | 6.92263 | 7.597527 | 1.15E-07 | 6.91E-07 | F5 |
| 10366653 | 1.696396 | 6.840668 | -7.59565 | 1.16E-07 | 6.94E-07 | Wif1 |
| 10396877 | 1.124856 | 5.315525 | -7.59487 | 1.16E-07 | 6.95E-07 | Galnt16 |
| 10490053 | -1.08041 | 6.445412 | 7.593715 | 1.16E-07 | 6.96E-07 | Zfp217 |
| 10427402 | 1.107921 | 7.776242 | -7.58901 | 1.17E-07 | 7.03E-07 | Ghr |
| 10476301 | -1.1223 | 7.249195 | 7.573636 | 1.21E-07 | 7.25E-07 | Smox |
| 10538924 | -1.80653 | 6.418767 | 7.573613 | 1.21E-07 | 7.25E-07 | Igkv2-109 |
| 10408197 | -1.16183 | 9.11606 | 7.563845 | 1.24E-07 | 7.39E-07 | Hist1h2bh |
| 10483081 | 1.629248 | 8.716187 | -7.5584 | 1.25E-07 | 7.47E-07 | Fap |
| 10531179 | 1.344995 | 4.861485 | -7.55707 | 1.26E-07 | 7.49E-07 | Adamts3 |
| 10349711 | 1.203671 | 6.980838 | -7.54492 | 1.29E-07 | 7.66E-07 | Slc41a1 |
| 10358637 | 1.05734 | 4.670319 | -7.53366 | 1.32E-07 | 7.84E-07 | Hmcn1 |
| 10368409 | 1.192285 | 7.025712 | -7.52117 | 1.36E-07 | 8.03E-07 | Lama2 |
| 10533050 | 1.396374 | 8.320539 | -7.51186 | 1.39E-07 | 8.18E-07 | Hspb8 |
| 10574415 | -1.11466 | 5.38503 | 7.502819 | 1.41E-07 | 8.33E-07 | 1700047G07Rik |
| 10605874 | 1.250671 | 5.148866 | -7.49972 | 1.42E-07 | 8.38E-07 | Eda2r |
| 10591112 | 1.414445 | 4.248657 | -7.49384 | 1.44E-07 | 8.48E-07 | Fat3 |
| 10376813 | -1.02603 | 7.890393 | 7.492041 | 1.45E-07 | 8.51E-07 | Specc1 |
| 10508651 | 1.538962 | 7.774569 | -7.48468 | 1.47E-07 | 8.64E-07 | Sdc3 |
| 10538890 | -1.40915 | 4.568 | 7.478242 | 1.49E-07 | 8.75E-07 | Sympk |
| 10463643 | -1.05472 | 4.121489 | 7.462261 | 1.55E-07 | 9.04E-07 | Gm24610 |
| 10464471 | 1.294753 | 4.9985 | -7.45637 | 1.57E-07 | 9.15E-07 | Gal |
| 10541885 | -1.06115 | 5.226325 | 7.440065 | 1.62E-07 | 9.44E-07 | Scnn1a |
| 10405033 | 1.428999 | 7.598467 | -7.42374 | 1.68E-07 | 9.77E-07 | Ecm2 |
| 10591114 | 1.271231 | 5.860757 | -7.4157 | 1.71E-07 | 9.92E-07 | Fat3 |
| 10461558 | -1.12333 | 7.138899 | 7.415194 | 1.71E-07 | 9.93E-07 | Slc15a3 |
| 10358057 | 1.128533 | 7.853924 | -7.41501 | 1.71E-07 | 9.93E-07 | Shisa4 |
| 10494978 | -1.03313 | 7.04641 | 7.41239 | 1.72E-07 | 9.98E-07 | Ptpn22 |
| 10545014 | -1.0888 | 7.472939 | 7.40511 | 1.75E-07 | 1.01E-06 | Vopp1 |
| 10579012 | 1.260353 | 5.790041 | -7.39608 | 1.79E-07 | 1.03E-06 | Csgalnact1 |
| 10400984 | 1.090679 | 5.669135 | -7.38804 | 1.82E-07 | 1.05E-06 | Tmem30b |
| 10375515 | -1.17051 | 5.832351 | 7.378685 | 1.86E-07 | 1.07E-06 | Ifi47 |
| 10561461 | 1.213958 | 7.25891 | -7.3609 | 1.93E-07 | 1.11E-06 | Samd4b |
| 10560624 | 1.210071 | 10.91237 | -7.35252 | 1.97E-07 | 1.13E-06 | Apoe |
| 10444890 | 1.057323 | 7.761076 | -7.34813 | 1.99E-07 | 1.14E-06 | Ier3 |
| 10525365 | -1.02734 | 6.152413 | 7.347512 | 1.99E-07 | 1.14E-06 | Hvcn1 |
| 10400589 | -1.37922 | 6.959432 | 7.322791 | 2.10E-07 | 1.20E-06 | Mis18bp1 |
| 10402708 | 1.592089 | 9.346167 | -7.31929 | 2.12E-07 | 1.20E-06 | Ckb |
| 10400157 | 1.059959 | 5.404829 | -7.31505 | 2.14E-07 | 1.21E-06 | Nova1 |
| 10398996 | 1.104517 | 7.379236 | -7.31122 | 2.15E-07 | 1.22E-06 | Crip2 |
| 10405189 | -1.14415 | 4.344232 | 7.306193 | 2.18E-07 | 1.24E-06 | Gm22806 |
| 10528227 | 1.550612 | 6.92314 | -7.30337 | 2.19E-07 | 1.24E-06 | Gnai1 |
| 10360344 | -1.04366 | 6.421381 | 7.298397 | 2.22E-07 | 1.25E-06 | Ackr1 |
| 10462281 | 1.431392 | 7.84297 | -7.29721 | 2.22E-07 | 1.26E-06 | Vldlr |
| 10502552 | -1.79328 | 5.283162 | 7.297103 | 2.22E-07 | 1.26E-06 | Clca1 |
| 10400948 | 1.19447 | 3.995574 | -7.29635 | 2.23E-07 | 1.26E-06 | 4930447C04Rik |
| 10514049 | 1.398738 | 8.676201 | -7.292 | 2.25E-07 | 1.27E-06 | Nfib |
| 10556456 | 1.273754 | 8.22736 | -7.28771 | 2.27E-07 | 1.28E-06 | Tead1 |
| 10576010 | -1.03451 | 7.238455 | 7.28115 | 2.30E-07 | 1.30E-06 | Gse1 |
| 10591135 | 1.850636 | 7.701201 | -7.27869 | 2.32E-07 | 1.30E-06 | Fat3 |
| 10347925 | -1.52098 | 5.428548 | 7.275896 | 2.33E-07 | 1.31E-06 | Gm7609 |
| 10590558 | 1.183486 | 5.872765 | -7.27437 | 2.34E-07 | 1.32E-06 | Clec3b |
| 10387890 | 1.20532 | 6.710213 | -7.26886 | 2.37E-07 | 1.33E-06 | Cxcl16 |
| 10590628 | -1.47978 | 5.753108 | 7.261065 | 2.41E-07 | 1.35E-06 | Ccr3 |
| 10385776 | 1.03603 | 5.879506 | -7.25454 | 2.44E-07 | 1.37E-06 | Tcf7 |
| 10358549 | 1.274336 | 5.20246 | -7.25067 | 2.46E-07 | 1.38E-06 | Hmcn1 |
| 10358601 | 1.173764 | 5.430834 | -7.24556 | 2.49E-07 | 1.39E-06 | Hmcn1 |
| 10545210 | -2.55865 | 5.415578 | 7.240809 | 2.52E-07 | 1.41E-06 | Igkv4-55 |
| 10483249 | 1.043705 | 6.440849 | -7.24068 | 2.52E-07 | 1.41E-06 | Galnt3 |
| 10582879 | -1.72008 | 6.035153 | 7.238577 | 2.53E-07 | 1.41E-06 | Csprs |
| 10540523 | 1.188146 | 8.41338 | -7.23666 | 2.54E-07 | 1.42E-06 | Lmcd1 |
| 10420302 | 1.372382 | 4.393115 | -7.22877 | 2.59E-07 | 1.44E-06 | Gzmc |
| 10465132 | -1.0698 | 7.138429 | 7.221229 | 2.63E-07 | 1.46E-06 | Sipa1 |
| 10414590 | -2.55037 | 6.248807 | 7.214333 | 2.67E-07 | 1.49E-06 | Ear6 |
| 10356274 | -1.5758 | 5.587093 | 7.214059 | 2.67E-07 | 1.49E-06 | Csprs |
| 10430344 | -1.60829 | 5.849069 | 7.210137 | 2.70E-07 | 1.50E-06 | Il2rb |
| 10464594 | -1.43778 | 5.320992 | 7.203963 | 2.73E-07 | 1.52E-06 | BC021614 |
| 10445826 | -1.01646 | 6.604169 | 7.202086 | 2.74E-07 | 1.52E-06 | Mocs1 |
| 10551347 | -1.53219 | 9.045409 | 7.201839 | 2.75E-07 | 1.52E-06 | Blvrb |
| 10403018 | -2.92271 | 6.829211 | 7.200853 | 2.75E-07 | 1.53E-06 | Igh-VJ558 |
| 10504761 | -1.42309 | 4.049264 | 7.18473 | 2.85E-07 | 1.58E-06 | Sympk |
| 10463112 | -1.09491 | 5.704642 | 7.184213 | 2.86E-07 | 1.58E-06 | Ccnj |
| 10531183 | 1.266224 | 4.680938 | -7.1842 | 2.86E-07 | 1.58E-06 | Adamts3 |
| 10360406 | 1.613622 | 6.885326 | -7.18301 | 2.86E-07 | 1.58E-06 | Ifi205 |
| 10501048 | -1.12396 | 5.012374 | 7.179857 | 2.88E-07 | 1.59E-06 | 2010016I18Rik |
| 10555235 | -1.04923 | 7.585697 | 7.175402 | 2.91E-07 | 1.60E-06 | Arrb1 |
| 10437399 | -1.05012 | 7.26656 | 7.157522 | 3.03E-07 | 1.66E-06 | Coro7 |
| 10571865 | 1.728416 | 8.129531 | -7.15718 | 3.03E-07 | 1.66E-06 | Scrg1 |
| 10347915 | -1.5579 | 5.397823 | 7.149325 | 3.09E-07 | 1.69E-06 | Gm7609 |
| 10461594 | -1.57361 | 7.026762 | 7.134802 | 3.19E-07 | 1.74E-06 | Ms4a4c |
| 10392796 | -1.06678 | 5.895468 | 7.130575 | 3.22E-07 | 1.76E-06 | Cd300lb |
| 10512470 | -1.16351 | 6.129557 | 7.126138 | 3.25E-07 | 1.77E-06 | Cd72 |
| 10532744 | -1.51307 | 8.707706 | 7.125399 | 3.26E-07 | 1.77E-06 | Selplg |
| 10501608 | -1.01245 | 8.62071 | 7.124522 | 3.26E-07 | 1.78E-06 | Vcam1 |
| 10571715 | 1.084614 | 6.433322 | -7.12343 | 3.27E-07 | 1.78E-06 | Enpp6 |
| 10548333 | -1.00022 | 4.037372 | 7.119728 | 3.30E-07 | 1.79E-06 | Cd69 |
| 10518570 | -1.01717 | 8.751263 | 7.097965 | 3.46E-07 | 1.88E-06 | Pgd |
| 10502565 | -1.27497 | 5.508322 | 7.094894 | 3.49E-07 | 1.89E-06 | Clca2 |
| 10567171 | -1.40459 | 5.992126 | 7.090018 | 3.52E-07 | 1.91E-06 | Snord14a |
| 10404429 | 1.173226 | 5.423501 | -7.08509 | 3.56E-07 | 1.93E-06 | Serpinb9 |
| 10444223 | -1.54132 | 5.872799 | 7.080911 | 3.60E-07 | 1.94E-06 | H2-Oa |
| 10358625 | 1.055256 | 5.02106 | -7.08082 | 3.60E-07 | 1.94E-06 | Hmcn1 |
| 10579958 | -1.12405 | 6.104987 | 7.073209 | 3.66E-07 | 1.97E-06 | Il15 |
| 10607124 | 1.118635 | 7.740456 | -7.06724 | 3.71E-07 | 2.00E-06 | Chrdl1 |
| 10547664 | -1.65695 | 5.909291 | 7.064909 | 3.73E-07 | 2.01E-06 | Clec4e |
| 10504753 | -1.35619 | 4.088454 | 7.060698 | 3.76E-07 | 2.03E-06 | Sympk |
| 10545208 | -2.42898 | 5.951176 | 7.053643 | 3.82E-07 | 2.06E-06 | Igkv4-57 |
| 10598032 | -1.55009 | 8.568995 | 7.03719 | 3.97E-07 | 2.13E-06 | mt-Tm |
| 10406672 | 1.11711 | 8.950332 | -7.03437 | 3.99E-07 | 2.14E-06 | Arsb |
| 10353545 | 1.038462 | 7.235923 | -7.01832 | 4.14E-07 | 2.21E-06 | 1110058L19Rik |
| 10355706 | 1.173737 | 6.107181 | -7.01666 | 4.15E-07 | 2.22E-06 | Ihh |
| 10438690 | -1.18098 | 6.89731 | 7.013381 | 4.19E-07 | 2.23E-06 | Rfc4 |
| 10581266 | 1.150975 | 6.836975 | -7.00085 | 4.31E-07 | 2.29E-06 | Tppp3 |
| 10585778 | 1.008997 | 7.31158 | -6.9949 | 4.36E-07 | 2.32E-06 | Sema7a |
| 10471880 | 1.418408 | 5.97543 | -6.9942 | 4.37E-07 | 2.32E-06 | Mir181b-2 |
| 10565990 | -1.02208 | 5.367405 | 6.981406 | 4.50E-07 | 2.38E-06 | Art2a-ps |
| 10398364 | 1.737412 | 6.017773 | -6.97848 | 4.53E-07 | 2.39E-06 | DQ267102 |
| 10503508 | 1.032528 | 6.88075 | -6.97712 | 4.54E-07 | 2.40E-06 | Ggh |
| 10503523 | 1.032528 | 6.88075 | -6.97712 | 4.54E-07 | 2.40E-06 | Ggh |
| 10483819 | 1.257942 | 5.749013 | -6.97408 | 4.57E-07 | 2.41E-06 | Ttc30b |
| 10417526 | -1.30064 | 4.59376 | 6.964948 | 4.67E-07 | 2.46E-06 | Dnase1l3 |
| 10358648 | 1.102832 | 4.548819 | -6.95795 | 4.74E-07 | 2.50E-06 | Hmcn1 |
| 10386020 | 1.26887 | 6.80883 | -6.95506 | 4.77E-07 | 2.51E-06 | Slc36a2 |
| 10536667 | 1.877763 | 6.377648 | -6.9247 | 5.11E-07 | 2.67E-06 | Ptprz1 |
| 10479192 | 1.101135 | 8.860383 | -6.91902 | 5.18E-07 | 2.70E-06 | Gm14305 |
| 10479195 | 1.101135 | 8.860383 | -6.91902 | 5.18E-07 | 2.70E-06 | Gm14305 |
| 10479198 | 1.101135 | 8.860383 | -6.91902 | 5.18E-07 | 2.70E-06 | Gm14305 |
| 10490273 | 1.101135 | 8.860383 | -6.91902 | 5.18E-07 | 2.70E-06 | Gm14305 |
| 10488575 | -1.14966 | 7.352911 | 6.914728 | 5.23E-07 | 2.73E-06 | Psmf1 |
| 10434698 | 1.099105 | 4.875874 | -6.91351 | 5.24E-07 | 2.73E-06 | Fetub |
| 10538135 | -1.49724 | 4.81783 | 6.90076 | 5.40E-07 | 2.80E-06 | Gimap7 |
| 10382341 | 1.163106 | 4.785055 | -6.90076 | 5.40E-07 | 2.80E-06 | Sstr2 |
| 10582337 | 1.048452 | 7.022718 | -6.88785 | 5.56E-07 | 2.88E-06 | Piezo1 |
| 10492964 | -1.3614 | 4.547207 | 6.885701 | 5.58E-07 | 2.90E-06 | Cd5l |
| 10513957 | 1.111297 | 8.364101 | -6.8664 | 5.83E-07 | 3.01E-06 | Ptprd |
| 10431915 | 1.333449 | 6.685352 | -6.86607 | 5.84E-07 | 3.01E-06 | Slc38a4 |
| 10358597 | 1.001571 | 3.701452 | -6.84201 | 6.16E-07 | 3.16E-06 | Hmcn1 |
| 10436106 | -1.05088 | 7.198576 | 6.837868 | 6.22E-07 | 3.19E-06 | C330027C09Rik |
| 10409990 | 1.611682 | 6.888316 | -6.83561 | 6.25E-07 | 3.20E-06 | 6720489N17Rik |
| 10550877 | -1.54814 | 7.175983 | 6.834819 | 6.27E-07 | 3.21E-06 | Kcnn4 |
| 10600698 | -1.09217 | 5.595853 | 6.796938 | 6.83E-07 | 3.48E-06 | 5430427O19Rik |
| 10461158 | -1.22509 | 7.85331 | 6.784922 | 7.02E-07 | 3.57E-06 | Gm22680 |
| 10494114 | -1.02961 | 6.93555 | 6.778463 | 7.12E-07 | 3.62E-06 | Selenbp1 |
| 10475567 | -1.00102 | 6.461738 | 6.774426 | 7.19E-07 | 3.65E-06 | Slc24a5 |
| 10413542 | -1.19826 | 8.699759 | 6.772366 | 7.22E-07 | 3.66E-06 | Tkt |
| 10436024 | -1.62702 | 5.640492 | 6.767961 | 7.30E-07 | 3.69E-06 | Gcsam |
| 10439299 | -1.4781 | 6.470041 | 6.754473 | 7.52E-07 | 3.80E-06 | Stfa3 |
| 10436600 | 1.912268 | 6.558082 | -6.75294 | 7.55E-07 | 3.81E-06 | Mir99a |
| 10424781 | -1.50036 | 9.145847 | 6.752655 | 7.55E-07 | 3.81E-06 | Grina |
| 10607499 | 1.712204 | 7.863248 | -6.74907 | 7.62E-07 | 3.84E-06 | Phex |
| 10404036 | -1.16918 | 6.391056 | 6.735323 | 7.86E-07 | 3.95E-06 | Hist1h2bg |
| 10416037 | -1.0317 | 6.361708 | 6.724411 | 8.06E-07 | 4.04E-06 | Pbk |
| 10592330 | -1.27126 | 6.936565 | 6.723883 | 8.07E-07 | 4.05E-06 | Nrgn |
| 10562657 | 1.236767 | 5.270258 | -6.70894 | 8.35E-07 | 4.17E-06 | Gm5595 |
| 10434668 | 1.139344 | 6.926317 | -6.70776 | 8.37E-07 | 4.18E-06 | Tmem97 |
| 10507742 | 1.181331 | 6.666254 | -6.70527 | 8.42E-07 | 4.20E-06 | Col9a2 |
| 10465895 | 1.031697 | 8.26686 | -6.69974 | 8.53E-07 | 4.25E-06 | Fads2 |
| 10590306 | 1.181096 | 6.397206 | -6.69771 | 8.57E-07 | 4.27E-06 | Entpd3 |
| 10480849 | -1.10911 | 6.328338 | 6.691322 | 8.69E-07 | 4.32E-06 | Fcna |
| 10542470 | -1.12946 | 9.304116 | 6.648703 | 9.58E-07 | 4.73E-06 | Mgst1 |
| 10427436 | -1.21708 | 5.494909 | 6.64026 | 9.77E-07 | 4.81E-06 | C7 |
| 10545215 | -3.23643 | 5.56515 | 6.634532 | 9.90E-07 | 4.87E-06 | Igkv12-46 |
| 10444244 | -1.33151 | 6.76726 | 6.632285 | 9.95E-07 | 4.90E-06 | Tap1 |
| 10480139 | 1.01954 | 5.398729 | -6.63014 | 1.00E-06 | 4.92E-06 | C1ql3 |
| 10364950 | 1.061609 | 7.272043 | -6.62791 | 1.01E-06 | 4.94E-06 | Gadd45b |
| 10434745 | -1.31649 | 7.808047 | 6.623106 | 1.02E-06 | 4.99E-06 | Gm24616 |
| 10509992 | 1.177342 | 8.018817 | -6.61281 | 1.04E-06 | 5.10E-06 | Hspb7 |
| 10442932 | -1.2805 | 6.582061 | 6.599283 | 1.07E-06 | 5.25E-06 | Tmem8 |
| 10430245 | -1.07271 | 7.066857 | 6.597871 | 1.08E-06 | 5.26E-06 | Gm22107 |
| 10544932 | -1.18924 | 4.954113 | 6.589862 | 1.10E-06 | 5.35E-06 | Inmt |
| 10433101 | -1.00483 | 5.370024 | 6.584661 | 1.11E-06 | 5.41E-06 | Gpr84 |
| 10488459 | 1.087143 | 3.844613 | -6.56609 | 1.16E-06 | 5.63E-06 | Zfp442 |
| 10504668 | -1.23308 | 6.0068 | 6.553015 | 1.19E-06 | 5.78E-06 | E230008N13Rik |
| 10487597 | -1.5261 | 7.291185 | 6.528674 | 1.26E-06 | 6.09E-06 | Il1b |
| 10450501 | -1.08026 | 5.962488 | 6.525417 | 1.27E-06 | 6.13E-06 | Tnf |
| 10526656 | -1.07988 | 7.865724 | 6.519825 | 1.29E-06 | 6.20E-06 | Lrch4 |
| 10593015 | -1.41004 | 5.502421 | 6.51959 | 1.29E-06 | 6.20E-06 | Cd3g |
| 10481164 | -1.01933 | 6.193465 | 6.516364 | 1.30E-06 | 6.24E-06 | Slc2a6 |
| 10591125 | 1.546145 | 6.492726 | -6.49982 | 1.35E-06 | 6.47E-06 | Fat3 |
| 10430372 | -1.53396 | 9.680892 | 6.499347 | 1.35E-06 | 6.47E-06 | Rac2 |
| 10421517 | -1.38987 | 4.968308 | 6.485123 | 1.40E-06 | 6.67E-06 | Cysltr2 |
| 10403054 | -2.19469 | 7.210829 | 6.475539 | 1.43E-06 | 6.80E-06 | Igh-VJ558 |
| 10392834 | 1.215087 | 7.643104 | -6.47184 | 1.44E-06 | 6.85E-06 | Gm11710 |
| 10525158 | -1.10321 | 4.688331 | 6.470219 | 1.45E-06 | 6.88E-06 | Oas1b |
| 10383192 | -1.10312 | 6.826534 | 6.455745 | 1.50E-06 | 7.09E-06 | Rnf213 |
| 10591127 | 1.601571 | 6.21816 | -6.45366 | 1.50E-06 | 7.12E-06 | Fat3 |
| 10442643 | -1.05878 | 6.099312 | 6.4478 | 1.52E-06 | 7.21E-06 | Nme3 |
| 10596072 | 1.2029 | 6.833626 | -6.44653 | 1.53E-06 | 7.23E-06 | Ppp2r3a |
| 10394054 | -1.22084 | 5.649368 | 6.434429 | 1.57E-06 | 7.42E-06 | Cd7 |
| 10582376 | 1.025531 | 7.771833 | -6.43221 | 1.58E-06 | 7.46E-06 | Piezo1 |
| 10574023 | 1.041996 | 9.658193 | -6.41114 | 1.66E-06 | 7.80E-06 | Mt2 |
| 10545220 | -2.09699 | 4.628007 | 6.407433 | 1.67E-06 | 7.86E-06 | Igkv12-41 |
| 10451287 | -1.38923 | 6.826215 | 6.398155 | 1.71E-06 | 8.01E-06 | Gm9706 |
| 10370644 | -1.11101 | 6.061064 | 6.389126 | 1.75E-06 | 8.17E-06 | Prss57 |
| 10461152 | -1.03118 | 6.631573 | 6.387311 | 1.75E-06 | 8.20E-06 | Gm24452 |
| 10466200 | 1.427663 | 7.903265 | -6.37805 | 1.79E-06 | 8.36E-06 | Ms4a7 |
| 10463632 | -1.13088 | 5.893632 | 6.374356 | 1.81E-06 | 8.43E-06 | Tmem180 |
| 10555389 | -1.36189 | 9.92538 | 6.372528 | 1.81E-06 | 8.46E-06 | Ucp2 |
| 10535282 | -1.04869 | 5.465158 | 6.371704 | 1.82E-06 | 8.47E-06 | Card11 |
| 10389654 | -1.1861 | 5.320222 | 6.336231 | 1.97E-06 | 9.14E-06 | Epx |
| 10538993 | -1.01408 | 5.142288 | 6.335547 | 1.98E-06 | 9.16E-06 | Cd8a |
| 10398075 | 1.202785 | 7.491063 | -6.33482 | 1.98E-06 | 9.17E-06 | Serpina3n |
| 10574259 | -1.25821 | 7.034629 | 6.329408 | 2.01E-06 | 9.27E-06 | Gpr56 |
| 10471721 | -1.37853 | 7.372131 | 6.324871 | 2.03E-06 | 9.36E-06 | Ptgs1 |
| 10473406 | -1.30655 | 5.51583 | 6.317764 | 2.06E-06 | 9.50E-06 | Prg3 |
| 10574163 | -1.09346 | 6.111104 | 6.31384 | 2.08E-06 | 9.58E-06 | Nlrc5 |
| 10401931 | -1.17794 | 4.448624 | 6.312788 | 2.09E-06 | 9.60E-06 | Sympk |
| 10401937 | -1.17794 | 4.448624 | 6.312788 | 2.09E-06 | 9.60E-06 | Sympk |
| 10466210 | 1.611278 | 6.471258 | -6.30251 | 2.14E-06 | 9.81E-06 | Ms4a6d |
| 10585976 | 1.347674 | 7.485196 | -6.29778 | 2.16E-06 | 9.91E-06 | Myo9a |
| 10358617 | 1.11495 | 5.171028 | -6.29764 | 2.16E-06 | 9.91E-06 | Hmcn1 |
| 10374777 | 1.554206 | 7.212218 | -6.28646 | 2.22E-06 | 1.01E-05 | Efemp1 |
| 10439009 | 1.00257 | 9.653856 | -6.27927 | 2.26E-06 | 1.03E-05 | Apod |
| 10458278 | -1.47505 | 5.602839 | 6.261611 | 2.35E-06 | 1.07E-05 | Mzb1 |
| 10447317 | 1.065403 | 8.444241 | -6.25964 | 2.36E-06 | 1.08E-05 | Epas1 |
| 10379511 | 1.417176 | 6.563588 | -6.25807 | 2.37E-06 | 1.08E-05 | Ccl2 |
| 10517508 | 1.404956 | 8.295529 | -6.25533 | 2.39E-06 | 1.09E-05 | C1qb |
| 10503264 | -1.07494 | 7.362182 | 6.254204 | 2.39E-06 | 1.09E-05 | Ccne2 |
| 10478508 | 1.057983 | 8.581965 | -6.24327 | 2.45E-06 | 1.12E-05 | Pigt |
| 10521537 | 1.27223 | 6.53828 | -6.23759 | 2.49E-06 | 1.13E-05 | Cytl1 |
| 10431051 | 1.390567 | 5.99246 | -6.23228 | 2.52E-06 | 1.14E-05 | Scube1 |
| 10403079 | -1.99589 | 6.387808 | 6.212985 | 2.64E-06 | 1.19E-05 | LOC435333 |
| 10403943 | -1.17749 | 10.22787 | 6.207594 | 2.67E-06 | 1.21E-05 | Hist1h2bm |
| 10387536 | 1.401222 | 9.359187 | -6.19521 | 2.75E-06 | 1.24E-05 | Cd68 |
| 10392825 | 1.200713 | 7.798521 | -6.16216 | 2.97E-06 | 1.33E-05 | Gm11710 |
| 10566346 | -1.20416 | 5.478329 | 6.137568 | 3.15E-06 | 1.40E-05 | Trim12c |
| 10461614 | -1.06826 | 8.421511 | 6.102708 | 3.42E-06 | 1.52E-05 | Ms4a6c |
| 10545177 | -2.15861 | 6.088232 | 6.07757 | 3.63E-06 | 1.60E-05 | Igkv19-93 |
| 10351131 | -1.17604 | 6.447352 | 6.068474 | 3.70E-06 | 1.63E-05 | Myoc |
| 10379190 | 1.030053 | 5.691273 | -6.06801 | 3.71E-06 | 1.63E-05 | Vtn |
| 10467508 | -1.1615 | 6.125029 | 6.056857 | 3.81E-06 | 1.67E-05 | Blnk |
| 10474700 | 1.040725 | 10.0688 | -6.02795 | 4.08E-06 | 1.78E-05 | Thbs1 |
| 10576140 | -1.06164 | 6.1813 | 6.01554 | 4.20E-06 | 1.83E-05 | Cdt1 |
| 10571601 | 1.114218 | 10.28916 | -5.97311 | 4.64E-06 | 2.01E-05 | Pdlim3 |
| 10586172 | -1.01103 | 4.544225 | 5.957191 | 4.82E-06 | 2.08E-05 | Gm23136 |
| 10519855 | 1.156574 | 7.102944 | -5.95002 | 4.90E-06 | 2.11E-05 | Cacna2d1 |
| 10347583 | 1.199831 | 9.272804 | -5.94915 | 4.91E-06 | 2.11E-05 | Des |
| 10377982 | 1.112378 | 9.103009 | -5.91351 | 5.35E-06 | 2.28E-05 | Kif1c |
| 10549445 | -1.0716 | 6.399404 | 5.904098 | 5.47E-06 | 2.33E-05 | Gm23456 |
| 10582275 | -1.24214 | 8.264234 | 5.873189 | 5.89E-06 | 2.50E-05 | Slc7a5 |
| 10550320 | -1.18033 | 5.912176 | 5.873128 | 5.89E-06 | 2.50E-05 | n-R5s151 |
| 10582562 | -2.291 | 9.580691 | 5.851727 | 6.20E-06 | 2.62E-05 | n-R5s151 |
| 10472923 | 1.078348 | 5.446321 | -5.83817 | 6.40E-06 | 2.70E-05 | Ak4 |
| 10555862 | -1.00583 | 6.078764 | 5.799219 | 7.03E-06 | 2.93E-05 | Trim34a |
| 10591739 | 1.38079 | 10.46267 | -5.79278 | 7.14E-06 | 2.98E-05 | Acp5 |
| 10398039 | -1.13695 | 5.028326 | 5.783342 | 7.30E-06 | 3.04E-05 | Serpina3f |
| 10358611 | 1.156191 | 5.160383 | -5.77959 | 7.36E-06 | 3.06E-05 | Hmcn1 |
| 10493203 | 1.025457 | 7.9965 | -5.76743 | 7.58E-06 | 3.14E-05 | 0610031J06Rik |
| 10601416 | -1.07958 | 3.782168 | 5.763122 | 7.66E-06 | 3.17E-05 | P2ry10 |
| 10381809 | -1.10354 | 8.185271 | 5.761148 | 7.70E-06 | 3.19E-05 | Itgb3 |
| 10574098 | -1.09502 | 5.825597 | 5.751976 | 7.87E-06 | 3.25E-05 | Nlrc5 |
| 10563770 | 1.440149 | 7.545067 | -5.73427 | 8.21E-06 | 3.38E-05 | Csrp3 |
| 10585978 | 1.045634 | 6.743256 | -5.72527 | 8.39E-06 | 3.45E-05 | Myo9a |
| 10598073 | -1.05868 | 10.77015 | 5.695948 | 9.00E-06 | 3.68E-05 | mt-Tq |
| 10403073 | -1.31539 | 3.862815 | 5.673107 | 9.51E-06 | 3.87E-05 | Ighv1-84 |
| 10410984 | -1.5252 | 8.005792 | 5.630203 | 1.05E-05 | 4.26E-05 | Ckmt2 |
| 10420247 | 1.249101 | 6.058443 | -5.60939 | 1.11E-05 | 4.46E-05 | Mcpt4 |
| 10583163 | -1.06225 | 6.097788 | 5.60893 | 1.11E-05 | 4.46E-05 | Trpc6 |
| 10592888 | -1.05385 | 4.410029 | 5.57817 | 1.20E-05 | 4.78E-05 | Cxcr5 |
| 10406663 | 1.151444 | 8.74574 | -5.56972 | 1.22E-05 | 4.87E-05 | Arsb |
| 10424555 | -1.11823 | 6.878708 | 5.561936 | 1.24E-05 | 4.95E-05 | Gm25987 |
| 10566358 | -1.04525 | 7.813865 | 5.529768 | 1.34E-05 | 5.31E-05 | Trim30a |
| 10601385 | -1.06307 | 6.907081 | 5.484245 | 1.50E-05 | 5.87E-05 | Tlr13 |
| 10380398 | 1.816981 | 8.985461 | -5.45813 | 1.60E-05 | 6.21E-05 | Chad |
| 10405058 | 1.564427 | 8.0081 | -5.45726 | 1.60E-05 | 6.22E-05 | Omd |
| 10519815 | 1.226808 | 7.504979 | -5.41855 | 1.76E-05 | 6.78E-05 | Cacna2d1 |
| 10346150 | 1.022079 | 5.063496 | -5.39589 | 1.86E-05 | 7.14E-05 | Tmeff2 |
| 10598041 | -1.28939 | 8.008769 | 5.395118 | 1.86E-05 | 7.15E-05 | mt-Tk |
| 10454731 | -1.13935 | 4.511311 | 5.379755 | 1.93E-05 | 7.40E-05 | n-R5s25 |
| 10598077 | -1.56324 | 8.826488 | 5.368953 | 1.98E-05 | 7.58E-05 | mt-Tn |
| 10545235 | -1.10966 | 3.936915 | 5.363019 | 2.01E-05 | 7.68E-05 | Igkv8-28 |
| 10453231 | 1.070882 | 4.269901 | -5.35742 | 2.04E-05 | 7.78E-05 | Slc8a1 |
| 10425852 | -1.49086 | 8.619842 | 5.321118 | 2.23E-05 | 8.44E-05 | Parvb |
| 10501555 | -1.21316 | 6.773145 | 5.310603 | 2.29E-05 | 8.64E-05 | Amy1 |
| 10545217 | -1.18033 | 3.479818 | 5.308665 | 2.30E-05 | 8.67E-05 | Igkv5-45 |
| 10542214 | -1.22153 | 6.256102 | 5.298267 | 2.36E-05 | 8.87E-05 | Klrd1 |
| 10367076 | -1.08877 | 7.302747 | 5.288873 | 2.41E-05 | 9.06E-05 | Prim1 |
| 10392364 | 1.019119 | 8.937281 | -5.27768 | 2.48E-05 | 9.28E-05 | Cacng1 |
| 10445112 | -1.9715 | 5.52987 | 5.25966 | 2.59E-05 | 9.66E-05 | Ubd |
| 10606058 | -1.02789 | 5.567254 | 5.235852 | 2.74E-05 | 0.000102 | Cxcr3 |
| 10407390 | 1.070426 | 7.814586 | -5.23443 | 2.75E-05 | 0.000102 | Ptbp1 |
| 10371662 | -1.55739 | 5.895987 | 5.210227 | 2.92E-05 | 0.000108 | Spic |
| 10582916 | -1.16412 | 3.973824 | 5.153401 | 3.36E-05 | 0.000122 | Gm17535 |
| 10598081 | -1.65389 | 8.492142 | 5.146569 | 3.41E-05 | 0.000124 | mt-Ty |
| 10346114 | -1.31915 | 6.603915 | 5.099649 | 3.83E-05 | 0.000138 | n-R5s211 |
| 10598062 | -1.24551 | 7.872204 | 5.097217 | 3.85E-05 | 0.000139 | mt-Th |
| 10500837 | -1.01412 | 6.502351 | 5.075696 | 4.06E-05 | 0.000146 | Dclre1b |
| 10439292 | -1.31071 | 7.945805 | 5.053918 | 4.28E-05 | 0.000153 | Stfa1 |
| 10545212 | -1.61997 | 4.779509 | 5.01987 | 4.66E-05 | 0.000166 | Igkv5-48 |
| 10435504 | -1.07522 | 4.277779 | 4.977623 | 5.17E-05 | 0.000182 | Gm5416 |
| 10574149 | -1.16752 | 6.872624 | 4.970485 | 5.26E-05 | 0.000185 | Nlrc5 |
| 10563883 | -1.05326 | 7.147488 | 4.917212 | 5.99E-05 | 0.000209 | Depdc1a |
| 10598064 | -1.27192 | 7.424458 | 4.876201 | 6.63E-05 | 0.000229 | mt-Ts2 |
| 10435043 | 1.125916 | 6.53193 | -4.85569 | 6.97E-05 | 0.00024 | Tm4sf19 |
| 10351509 | -1.05362 | 6.1847 | 4.852834 | 7.02E-05 | 0.000241 | Fcgr4 |
| 10481845 | -1.04786 | 6.937386 | 4.850938 | 7.06E-05 | 0.000242 | Mvb12b |
| 10351206 | -1.02325 | 6.567293 | 4.812482 | 7.76E-05 | 0.000264 | Selp |
| 10598057 | -1.15221 | 7.098651 | 4.803332 | 7.93E-05 | 0.000269 | mt-Tr |
| 10584821 | -1.14506 | 4.997009 | 4.795429 | 8.09E-05 | 0.000274 | Cd3d |
| 10497122 | -1.05912 | 7.17003 | 4.780484 | 8.39E-05 | 0.000284 | Depdc1a |
| 10585438 | 1.052594 | 6.153554 | -4.76306 | 8.76E-05 | 0.000295 | Crabp1 |
| 10544523 | 1.15263 | 7.241619 | -4.74667 | 9.12E-05 | 0.000306 | Rny1 |
| 10379727 | 1.214248 | 9.672395 | -4.68754 | 0.000106 | 0.00035 | Wfdc17 |
| 10608136 | -1.11082 | 8.018304 | 4.686573 | 0.000106 | 0.00035 | n-R5s1 |
| 10598079 | -1.71409 | 5.797643 | 4.658123 | 0.000114 | 0.000373 | mt-Tc |
| 10523693 | 1.442798 | 8.3566 | -4.6371 | 0.00012 | 0.000391 | Dmp1 |
| 10466127 | -1.06077 | 8.560923 | 4.632539 | 0.000121 | 0.000395 | AW112010 |
| 10354267 | -1.11185 | 6.48289 | 4.596888 | 0.000132 | 0.000429 | Mettl21c |
| 10417887 | -1.10721 | 7.787327 | 4.581681 | 0.000137 | 0.000444 | Mss51 |
| 10603003 | 1.17267 | 4.121263 | -4.57617 | 0.000139 | 0.00045 | Gm23794 |
| 10545180 | -1.29905 | 4.422536 | 4.559695 | 0.000145 | 0.000467 | Igkv4-91 |
| 10595094 | 1.014976 | 7.721311 | -4.53119 | 0.000155 | 0.000498 | Mlip |
| 10408543 | -1.33266 | 7.602349 | 4.514746 | 0.000162 | 0.000517 | Mylk4 |
| 10598075 | -1.38217 | 8.887612 | 4.499048 | 0.000168 | 0.000537 | mt-Ta |
| 10555233 | -1.15341 | 7.078141 | 4.361119 | 0.000237 | 0.000733 | n-R5s156 |
| 10389207 | -1.35943 | 7.994382 | 4.35823 | 0.000238 | 0.000737 | Ccl5 |
| 10525343 | -1.20511 | 7.928451 | 4.281333 | 0.000288 | 0.000876 | Myl2 |
| 10483046 | -1.04502 | 6.058599 | 4.242419 | 0.000318 | 0.000956 | Dpp4 |
| 10377429 | 1.316419 | 8.732075 | -4.22536 | 0.000331 | 0.000994 | Snord118 |
| 10399428 | 1.316419 | 8.732075 | -4.22536 | 0.000331 | 0.000994 | Snord118 |
| 10434747 | -1.05321 | 7.03047 | 4.177749 | 0.000373 | 0.001105 | Adipoq |
| 10513512 | 1.493721 | 4.068952 | -4.09865 | 0.000453 | 0.001314 | Mup2 |
| 10513497 | 1.56268 | 4.596578 | -4.07219 | 0.000484 | 0.001395 | Mup2 |
| 10513455 | 1.807058 | 5.008599 | -3.94998 | 0.000654 | 0.001837 | Mup2 |
| 10513467 | 1.531862 | 4.692203 | -3.94758 | 0.000658 | 0.001847 | Mup2 |
| 10513428 | 1.714252 | 4.690502 | -3.9344 | 0.000679 | 0.001902 | Mup2 |
| 10513420 | 1.841272 | 5.146372 | -3.89216 | 0.000754 | 0.002092 | Mup2 |
| 10513437 | 1.757367 | 4.973956 | -3.87135 | 0.000793 | 0.002192 | Mup10 |
| 10513472 | 1.702818 | 4.97174 | -3.80074 | 0.000943 | 0.00257 | Mup10 |
| 10582580 | -1.33339 | 7.989077 | 3.778986 | 0.000995 | 0.002699 | n-R5s136 |
| 10513504 | 1.71616 | 4.984692 | -3.77734 | 0.000999 | 0.002708 | Mup2 |
| 10520452 | 1.09492 | 4.139727 | -3.7527 | 0.001061 | 0.002863 | Il6 |
| 10451953 | -1.06567 | 9.37909 | 3.707753 | 0.001185 | 0.003164 | Lrg1 |
| 10545239 | -1.56 | 4.757236 | 3.547689 | 0.00175 | 0.004506 | LOC637260 |
| 10545237 | -1.49898 | 3.97359 | 3.54729 | 0.001752 | 0.00451 | Adck1 |
| 10378438 | -1.01102 | 4.962732 | 3.440515 | 0.002269 | 0.005693 | n-R5s71 |
| 10545242 | -1.50636 | 5.031215 | 3.433002 | 0.00231 | 0.005788 | Igkv6-20 |
| 10472378 | 1.092003 | 5.313771 | -3.38423 | 0.002598 | 0.006416 | Scn2a1 |
| 10345527 | -1.29783 | 3.926213 | 3.082404 | 0.005332 | 0.012199 | n-R5s210 |
| 10582582 | -1.09112 | 6.181988 | 2.831393 | 0.009558 | 0.020473 | Gm24089 |
| 10582584 | -1.09112 | 6.181988 | 2.831393 | 0.009558 | 0.020473 | Gm24089 |
| 10485357 | -1.05682 | 8.539814 | 2.39452 | 0.025341 | 0.048472 | Gm10800 |
