# Supplementary material for: Identification of miRNA Regulatory Networks and Candidate Markers for Fracture Healing in Mice
Source: Comput Math Methods Med. 2021 Nov 16;2021:2866475. doi: 10.1155/2021/2866475 (PMC8611357; doi:10.1155/2021/2866475)
Supplement: Supplementary 5 — Table S3: the DEmiRs between 20 days after fracture and 0 day. [file 2866475.f5.docx]

Table S3. The DEmiRs between 20 days after fracture and 0 day.

|  | logFC | AveExpr | t | P.Value | adj.P.Val | Symbol |
| --- | --- | --- | --- | --- | --- | --- |
| 10565067 | 2.473404 | 7.887278 | -24.7843 | 6.60E-18 | 1.90E-13 | Nmb |
| 10423894 | 2.920725 | 6.049277 | -23.0915 | 3.09E-17 | 4.45E-13 | Dcstamp |
| 10473281 | 2.460069 | 8.570831 | -22.3856 | 6.06E-17 | 5.82E-13 | Itgav |
| 10406672 | 2.164915 | 9.449287 | -21.5133 | 1.43E-16 | 1.03E-12 | Arsb |
| 10384398 | 2.579085 | 7.378326 | -21.0034 | 2.41E-16 | 1.23E-12 | Grb10 |
| 10422244 | 3.37432 | 5.183018 | -20.7693 | 3.07E-16 | 1.23E-12 | Slitrk6 |
| 10411373 | 1.603811 | 9.750179 | -20.7341 | 3.18E-16 | 1.23E-12 | Hexb |
| 10505143 | 2.396502 | 5.970428 | -20.5375 | 3.91E-16 | 1.23E-12 | Akap2 |
| 10353524 | -1.46458 | 7.27347 | 20.4906 | 4.10E-16 | 1.23E-12 | Ogfrl1 |
| 10497203 | 2.731213 | 7.087678 | -20.4526 | 4.27E-16 | 1.23E-12 | Hey1 |
| 10527158 | 2.723082 | 6.907461 | -20.3037 | 5.00E-16 | 1.30E-12 | Fscn1 |
| 10458999 | 1.625819 | 5.698063 | -20.227 | 5.42E-16 | 1.30E-12 | Fbn2 |
| 10408280 | 2.256834 | 6.499666 | -19.9697 | 7.14E-16 | 1.58E-12 | Lrrc16a |
| 10607143 | 2.281725 | 5.518349 | -19.8336 | 8.27E-16 | 1.64E-12 | Capn6 |
| 10365420 | 1.773829 | 8.308613 | -19.8029 | 8.55E-16 | 1.64E-12 | Tmem263 |
| 10568328 | 1.94319 | 8.13191 | -19.6791 | 9.78E-16 | 1.71E-12 | Vkorc1 |
| 10607283 | 2.050494 | 6.51769 | -19.6155 | 1.05E-15 | 1.71E-12 | Maged2 |
| 10492682 | 2.649265 | 7.077163 | -19.5133 | 1.17E-15 | 1.71E-12 | Fam198b |
| 10606609 | 2.421636 | 6.570846 | -19.5079 | 1.18E-15 | 1.71E-12 | Tspan6 |
| 10496262 | 3.500341 | 7.38274 | -19.5005 | 1.19E-15 | 1.71E-12 | Slc9b2 |
| 10473444 | 3.34699 | 7.49924 | -19.4029 | 1.32E-15 | 1.82E-12 | Aplnr |
| 10574438 | 2.58024 | 8.252719 | -19.1983 | 1.66E-15 | 2.18E-12 | Cdh5 |
| 10363224 | 2.330286 | 6.116785 | -19.0663 | 1.92E-15 | 2.41E-12 | Fabp7 |
| 10597413 | 2.58221 | 8.070273 | -19.0229 | 2.02E-15 | 2.43E-12 | Crtap |
| 10496359 | 2.569741 | 7.715407 | -18.6713 | 3.01E-15 | 3.34E-12 | Emcn |
| 10592355 | 4.079444 | 6.910395 | -18.6701 | 3.01E-15 | 3.34E-12 | Panx3 |
| 10485117 | 3.227443 | 8.148455 | -18.5819 | 3.33E-15 | 3.56E-12 | Creb3l1 |
| 10595404 | 1.81953 | 7.680268 | -18.4277 | 3.98E-15 | 4.03E-12 | Fam46a |
| 10587383 | 3.558872 | 7.58014 | -18.4035 | 4.09E-15 | 4.03E-12 | Cd109 |
| 10585860 | -2.11267 | 7.759872 | 18.38196 | 4.20E-15 | 4.03E-12 | Adpgk |
| 10568668 | 3.165203 | 6.919228 | -18.2692 | 4.78E-15 | 4.45E-12 | Adam12 |
| 10531675 | 1.678328 | 9.216173 | -18.2339 | 4.98E-15 | 4.49E-12 | Sec31a |
| 10362314 | 2.358824 | 6.973097 | -17.9772 | 6.74E-15 | 5.86E-12 | Ptprk |
| 10416689 | -3.04197 | 7.082771 | 17.95508 | 6.92E-15 | 5.86E-12 | Olfm4 |
| 10605919 | 1.561995 | 7.696648 | -17.875 | 7.60E-15 | 6.26E-12 | Pja1 |
| 10453178 | 2.155273 | 7.392442 | -17.8058 | 8.25E-15 | 6.61E-12 | Map4k3 |
| 10507612 | 2.588456 | 7.388234 | -17.7478 | 8.85E-15 | 6.86E-12 | Lepre1 |
| 10603896 | 1.965789 | 6.236051 | -17.7289 | 9.05E-15 | 6.86E-12 | Klhl13 |
| 10590844 | 2.472004 | 6.158649 | -17.7067 | 9.29E-15 | 6.86E-12 | Arhgap42 |
| 10362538 | 2.177108 | 7.080427 | -17.6594 | 9.83E-15 | 7.08E-12 | Lama4 |
| 10349174 | 1.651278 | 5.643803 | -17.6043 | 1.05E-14 | 7.38E-12 | Serpinb8 |
| 10422822 | 2.201843 | 9.265547 | -17.5832 | 1.08E-14 | 7.39E-12 | Lifr |
| 10519998 | 2.870461 | 6.951278 | -17.5306 | 1.15E-14 | 7.68E-12 | Lrrc17 |
| 10427744 | 2.058864 | 7.50283 | -17.5132 | 1.17E-14 | 7.68E-12 | Rai14 |
| 10457644 | 2.95623 | 7.26246 | -17.4857 | 1.21E-14 | 7.71E-12 | Cdh2 |
| 10492448 | 1.821951 | 6.251797 | -17.4726 | 1.23E-14 | 7.71E-12 | Ptx3 |
| 10499666 | 1.368784 | 6.569829 | -17.4057 | 1.34E-14 | 8.05E-12 | Atp8b2 |
| 10395103 | 2.119895 | 6.035309 | -17.3955 | 1.35E-14 | 8.05E-12 | Pxdn |
| 10355050 | 2.153327 | 8.014615 | -17.3834 | 1.37E-14 | 8.05E-12 | Raph1 |
| 10537062 | 2.681496 | 6.974141 | -17.3682 | 1.40E-14 | 8.05E-12 | Mest |
| 10602068 | 2.312129 | 6.800914 | -17.263 | 1.59E-14 | 8.95E-12 | Mid2 |
| 10417759 | 2.234556 | 6.765405 | -17.2498 | 1.61E-14 | 8.95E-12 | Ube2e2 |
| 10414102 | 1.575826 | 6.647239 | -17.2213 | 1.67E-14 | 9.09E-12 | Mmrn2 |
| 10344637 | 1.307034 | 7.751792 | -17.1549 | 1.81E-14 | 9.68E-12 | Atp6v1h |
| 10407803 | 2.58372 | 7.674042 | -17.1035 | 1.93E-14 | 9.95E-12 | Gpr137b |
| 10571530 | 2.661315 | 7.866274 | -17.1033 | 1.93E-14 | 9.95E-12 | Fat1 |
| 10564343 | 2.217921 | 7.808581 | -17.08 | 1.99E-14 | 1.01E-11 | Tjp1 |
| 10354777 | 2.776159 | 8.110942 | -17.0663 | 2.02E-14 | 1.01E-11 | Satb2 |
| 10366653 | 2.623778 | 7.282278 | -16.9799 | 2.25E-14 | 1.10E-11 | Wif1 |
| 10351491 | 2.483096 | 7.997415 | -16.959 | 2.31E-14 | 1.11E-11 | Olfml2b |
| 10601659 | 2.44462 | 6.963988 | -16.921 | 2.42E-14 | 1.14E-11 | Srpx2 |
| 10472440 | 1.583429 | 7.51481 | -16.8942 | 2.51E-14 | 1.16E-11 | Tax1bp3 |
| 10605766 | 2.487564 | 8.59083 | -16.8803 | 2.55E-14 | 1.17E-11 | Maged1 |
| 10378334 | 1.541589 | 7.508862 | -16.8115 | 2.78E-14 | 1.24E-11 | Tax1bp3 |
| 10355984 | 1.688319 | 10.34462 | -16.805 | 2.80E-14 | 1.24E-11 | Serpine2 |
| 10529957 | 1.671497 | 6.417829 | -16.7571 | 2.97E-14 | 1.30E-11 | Gpr125 |
| 10505120 | 1.921273 | 5.669089 | -16.7214 | 3.11E-14 | 1.34E-11 | Palm2 |
| 10361381 | -1.9599 | 7.557 | 16.6996 | 3.20E-14 | 1.35E-11 | Syne1 |
| 10536294 | 1.904788 | 6.643064 | -16.6878 | 3.24E-14 | 1.35E-11 | Peg10 |
| 10542355 | 1.850845 | 8.61081 | -16.6681 | 3.33E-14 | 1.36E-11 | Emp1 |
| 10495830 | 2.070072 | 7.507325 | -16.663 | 3.35E-14 | 1.36E-11 | Sec24d |
| 10590860 | 2.266058 | 6.352431 | -16.6539 | 3.39E-14 | 1.36E-11 | Arhgap42 |
| 10546454 | 2.806985 | 6.725884 | -16.4931 | 4.15E-14 | 1.64E-11 | Adamts9 |
| 10395163 | 1.74017 | 6.961685 | -16.4571 | 4.34E-14 | 1.69E-11 | Lamb1 |
| 10435641 | 2.550042 | 9.144026 | -16.4263 | 4.52E-14 | 1.74E-11 | Fstl1 |
| 10492355 | 2.713141 | 7.633297 | -16.4033 | 4.65E-14 | 1.74E-11 | Mme |
| 10592342 | 1.460142 | 8.397349 | -16.3908 | 4.73E-14 | 1.74E-11 | Tbrg1 |
| 10421361 | 2.489565 | 7.852924 | -16.386 | 4.76E-14 | 1.74E-11 | Bmp1 |
| 10529457 | 3.070315 | 7.007676 | -16.3844 | 4.77E-14 | 1.74E-11 | Cpz |
| 10381122 | 3.104666 | 7.587827 | -16.3222 | 5.16E-14 | 1.86E-11 | Fkbp10 |
| 10425287 | 3.216631 | 6.793045 | -16.2991 | 5.32E-14 | 1.89E-11 | Kdelr3 |
| 10460603 | 1.965311 | 8.376348 | -16.2886 | 5.39E-14 | 1.89E-11 | Efemp2 |
| 10531560 | 1.774814 | 7.902437 | -16.2658 | 5.55E-14 | 1.93E-11 | Antxr2 |
| 10370180 | 2.400039 | 7.54148 | -16.1976 | 6.06E-14 | 2.08E-11 | Col6a2 |
| 10376074 | 2.159286 | 6.415591 | -16.1497 | 6.45E-14 | 2.18E-11 | P4ha2 |
| 10502522 | 1.633864 | 6.896495 | -16.1441 | 6.50E-14 | 2.18E-11 | Hs2st1 |
| 10485645 | 2.236339 | 7.72531 | -16.0913 | 6.96E-14 | 2.30E-11 | Rcn1 |
| 10494467 | 1.80122 | 6.789764 | -16.0763 | 7.09E-14 | 2.32E-11 | Itga10 |
| 10356886 | -2.58253 | 5.37542 | 16.03873 | 7.45E-14 | 2.39E-11 | Slco4c1 |
| 10354432 | 2.594468 | 7.804826 | -16.0361 | 7.47E-14 | 2.39E-11 | Myo1b |
| 10432032 | 2.002846 | 7.519261 | -16.007 | 7.76E-14 | 2.46E-11 | Vdr |
| 10380699 | 2.606687 | 8.482748 | -15.9912 | 7.92E-14 | 2.46E-11 | Copz2 |
| 10371332 | 2.680358 | 6.286776 | -15.9889 | 7.95E-14 | 2.46E-11 | Aldh1l2 |
| 10426689 | 2.375896 | 6.492981 | -15.9764 | 8.08E-14 | 2.48E-11 | Spats2 |
| 10447799 | 1.322261 | 7.1489 | -15.9591 | 8.26E-14 | 2.51E-11 | Igf2r |
| 10547282 | 2.048628 | 6.348663 | -15.9508 | 8.35E-14 | 2.51E-11 | Zfp9 |
| 10567355 | 1.638794 | 5.749583 | -15.9215 | 8.68E-14 | 2.58E-11 | Gprc5b |
| 10467258 | 1.884779 | 7.152829 | -15.9045 | 8.88E-14 | 2.58E-11 | Myof |
| 10546432 | 2.818639 | 6.421412 | -15.8142 | 1.00E-13 | 2.86E-11 | Adamts9 |
| 10420891 | 2.29679 | 7.515237 | -15.798 | 1.02E-13 | 2.86E-11 | Scara3 |
| 10412921 | 1.579686 | 6.35201 | -15.7961 | 1.02E-13 | 2.86E-11 | Nid2 |
| 10427471 | 1.709653 | 6.908607 | -15.7951 | 1.03E-13 | 2.86E-11 | Osmr |
| 10468309 | 2.284167 | 8.591064 | -15.7891 | 1.03E-13 | 2.86E-11 | Sh3pxd2a |
| 10379184 | 1.769407 | 6.168089 | -15.7672 | 1.06E-13 | 2.90E-11 | Slc46a1 |
| 10458828 | 2.857465 | 7.81663 | -15.7647 | 1.07E-13 | 2.90E-11 | Cdo1 |
| 10484402 | 1.419608 | 7.816999 | -15.7474 | 1.09E-13 | 2.94E-11 | Ctnnd1 |
| 10496110 | 1.548073 | 6.075416 | -15.7161 | 1.14E-13 | 3.04E-11 | Papss1 |
| 10357280 | 1.520363 | 7.814767 | -15.704 | 1.16E-13 | 3.05E-11 | Insig2 |
| 10529875 | 1.738449 | 7.171708 | -15.6933 | 1.17E-13 | 3.05E-11 | Ldb2 |
| 10513739 | 2.373636 | 10.05301 | -15.6914 | 1.18E-13 | 3.05E-11 | Tnc |
| 10477777 | 1.29426 | 9.3086 | -15.6778 | 1.20E-13 | 3.08E-11 | Ergic3 |
| 10471424 | 1.770429 | 7.55083 | -15.6408 | 1.26E-13 | 3.17E-11 | Fam102a |
| 10351400 | 1.689023 | 6.066412 | -15.6316 | 1.27E-13 | 3.17E-11 | Fam78b |
| 10472757 | 2.224832 | 6.483512 | -15.6244 | 1.29E-13 | 3.17E-11 | Cybrd1 |
| 10519497 | 1.93314 | 8.089744 | -15.6237 | 1.29E-13 | 3.17E-11 | Steap4 |
| 10404407 | 1.974315 | 7.002635 | -15.6226 | 1.29E-13 | 3.17E-11 | Foxc1 |
| 10487040 | 2.119731 | 7.698201 | -15.6145 | 1.30E-13 | 3.18E-11 | Fbn1 |
| 10438421 | -1.62496 | 4.316393 | 15.57476 | 1.37E-13 | 3.32E-11 | Olfr164 |
| 10511779 | 3.108632 | 9.168519 | -15.5615 | 1.40E-13 | 3.36E-11 | Atp6v0d2 |
| 10495781 | 2.205576 | 6.82194 | -15.5467 | 1.43E-13 | 3.37E-11 | Bcar3 |
| 10464471 | 2.82058 | 5.725084 | -15.5463 | 1.43E-13 | 3.37E-11 | Gal |
| 10439710 | 1.969457 | 7.163967 | -15.5236 | 1.47E-13 | 3.44E-11 | Phldb2 |
| 10361509 | -1.66858 | 7.908255 | 15.51941 | 1.48E-13 | 3.44E-11 | Syne1 |
| 10423805 | 1.420568 | 8.530108 | -15.4886 | 1.54E-13 | 3.53E-11 | Atp6v1c1 |
| 10453759 | 2.27534 | 4.80171 | -15.486 | 1.55E-13 | 3.53E-11 | Gm10554 |
| 10456400 | 2.300193 | 7.724597 | -15.4766 | 1.57E-13 | 3.53E-11 | Tubb6 |
| 10559399 | 2.618657 | 6.546242 | -15.4708 | 1.58E-13 | 3.53E-11 | Oscar |
| 10372988 | 1.536516 | 6.173647 | -15.468 | 1.58E-13 | 3.53E-11 | Slc16a7 |
| 10400510 | 2.017714 | 6.060389 | -15.4641 | 1.59E-13 | 3.53E-11 | Clec14a |
| 10423293 | 1.851452 | 8.086939 | -15.3932 | 1.75E-13 | 3.85E-11 | Myo10 |
| 10458894 | 3.167819 | 8.542695 | -15.3632 | 1.82E-13 | 3.98E-11 | Lox |
| 10503448 | 3.246191 | 6.910539 | -15.3524 | 1.85E-13 | 4.00E-11 | Mmp16 |
| 10458906 | 2.718739 | 9.984259 | -15.3379 | 1.89E-13 | 4.00E-11 | Ppic |
| 10415052 | 2.49809 | 8.6882 | -15.3377 | 1.89E-13 | 4.00E-11 | Mmp14 |
| 10497548 | 1.646863 | 7.713235 | -15.3375 | 1.89E-13 | 4.00E-11 | Fndc3b |
| 10582241 | 1.633956 | 6.775483 | -15.3158 | 1.94E-13 | 4.09E-11 | Zcchc14 |
| 10354389 | 1.519563 | 6.621496 | -15.2971 | 1.99E-13 | 4.16E-11 | Slc39a10 |
| 10388160 | 3.437228 | 7.153627 | -15.2759 | 2.05E-13 | 4.25E-11 | Slc13a5 |
| 10581479 | 2.603023 | 7.549631 | -15.2712 | 2.07E-13 | 4.25E-11 | Smpd3 |
| 10409376 | -1.70187 | 6.010078 | 15.25215 | 2.12E-13 | 4.33E-11 | Hk3 |
| 10422946 | 2.133084 | 5.030448 | -15.2317 | 2.18E-13 | 4.42E-11 | Ranbp3l |
| 10467319 | 2.504193 | 6.702805 | -15.2194 | 2.22E-13 | 4.47E-11 | Rbp4 |
| 10389025 | 1.518826 | 8.487515 | -15.2022 | 2.27E-13 | 4.54E-11 | Myo1d |
| 10597531 | 1.501991 | 6.798554 | -15.1885 | 2.31E-13 | 4.59E-11 | Rbms3 |
| 10529034 | 3.320367 | 7.845101 | -15.1516 | 2.43E-13 | 4.80E-11 | Cgref1 |
| 10538547 | 2.685708 | 7.957728 | -15.1373 | 2.48E-13 | 4.86E-11 | Fkbp9 |
| 10435654 | 1.083749 | 8.815584 | -15.116 | 2.55E-13 | 4.97E-11 | Lrrc58 |
| 10348829 | 2.186369 | 6.591529 | -15.1111 | 2.57E-13 | 4.97E-11 | Farp2 |
| 10374248 | -2.56055 | 6.372597 | 15.10078 | 2.61E-13 | 5.01E-11 | Abca13 |
| 10483719 | 2.669309 | 6.166157 | -15.0951 | 2.63E-13 | 5.01E-11 | Chn1 |
| 10394674 | 2.031776 | 7.15104 | -15.0565 | 2.77E-13 | 5.22E-11 | Socs2 |
| 10534862 | 2.420102 | 10.24166 | -15.054 | 2.78E-13 | 5.22E-11 | Pcolce |
| 10407792 | 2.157528 | 8.684905 | -15.0509 | 2.79E-13 | 5.22E-11 | Gpr137b-ps |
| 10354741 | 1.884662 | 6.229033 | -15.0461 | 2.81E-13 | 5.22E-11 | Rftn2 |
| 10597239 | 2.28732 | 7.486728 | -15.0159 | 2.93E-13 | 5.39E-11 | Pth1r |
| 10505954 | 1.526219 | 6.420992 | -15.0133 | 2.94E-13 | 5.39E-11 | Tek |
| 10554800 | 1.406023 | 7.25575 | -14.9997 | 2.99E-13 | 5.46E-11 | Rab38 |
| 10356800 | 1.320645 | 10.16313 | -14.9258 | 3.32E-13 | 6.01E-11 | Hdlbp |
| 10433003 | 2.624685 | 7.716214 | -14.8675 | 3.59E-13 | 6.47E-11 | Sp7 |
| 10493709 | 1.433952 | 8.177417 | -14.8555 | 3.66E-13 | 6.54E-11 | Slc39a1 |
| 10505623 | 2.49587 | 5.897785 | -14.8284 | 3.80E-13 | 6.72E-11 | Lurap1l |
| 10421309 | 1.847691 | 6.281958 | -14.8227 | 3.83E-13 | 6.72E-11 | Slc39a14 |
| 10359754 | 1.876005 | 7.295379 | -14.8209 | 3.84E-13 | 6.72E-11 | Mpzl1 |
| 10596137 | 1.261054 | 7.392573 | -14.8189 | 3.85E-13 | 6.72E-11 | Srprb |
| 10500948 | 1.306178 | 6.715122 | -14.8057 | 3.92E-13 | 6.80E-11 | Cttnbp2nl |
| 10380566 | 1.930359 | 7.703758 | -14.7524 | 4.22E-13 | 7.26E-11 | Phospho1 |
| 10569011 | 2.902152 | 8.086051 | -14.7504 | 4.23E-13 | 7.26E-11 | Ifitm5 |
| 10432404 | 1.109175 | 10.26156 | -14.7433 | 4.27E-13 | 7.29E-11 | Tuba1a |
| 10501762 | 2.314777 | 7.11579 | -14.7205 | 4.41E-13 | 7.41E-11 | Snx7 |
| 10578572 | 2.698039 | 6.925657 | -14.7202 | 4.41E-13 | 7.41E-11 | Stox2 |
| 10382435 | 2.357237 | 7.340173 | -14.7159 | 4.44E-13 | 7.41E-11 | Gprc5c |
| 10382425 | 1.977455 | 7.077284 | -14.7149 | 4.45E-13 | 7.41E-11 | Gprc5c |
| 10534216 | 1.182573 | 8.519218 | -14.6953 | 4.57E-13 | 7.57E-11 | Gtf2i |
| 10468762 | 2.234202 | 6.885195 | -14.6911 | 4.60E-13 | 7.57E-11 | 4930506M07Rik |
| 10393823 | 1.260934 | 10.16543 | -14.6557 | 4.83E-13 | 7.91E-11 | P4hb |
| 10439651 | 2.018419 | 8.459036 | -14.6435 | 4.91E-13 | 8.00E-11 | Cd200 |
| 10480238 | 1.577428 | 5.753614 | -14.6157 | 5.11E-13 | 8.26E-11 | St8sia6 |
| 10474671 | 1.857692 | 7.786221 | -14.6128 | 5.13E-13 | 8.26E-11 | Spred1 |
| 10498584 | 1.760888 | 5.141748 | -14.6058 | 5.18E-13 | 8.30E-11 | Rarres1 |
| 10487021 | 2.204512 | 7.03344 | -14.5954 | 5.26E-13 | 8.37E-11 | Slc30a4 |
| 10504817 | 1.638745 | 8.786093 | -14.5823 | 5.36E-13 | 8.48E-11 | Tgfbr1 |
| 10379489 | 1.854482 | 5.703615 | -14.575 | 5.41E-13 | 8.52E-11 | Tmem98 |
| 10538290 | 1.540932 | 8.508925 | -14.57 | 5.45E-13 | 8.54E-11 | Snx10 |
| 10571715 | 1.934933 | 6.838236 | -14.5507 | 5.60E-13 | 8.72E-11 | Enpp6 |
| 10563077 | 2.606364 | 8.442413 | -14.5412 | 5.68E-13 | 8.77E-11 | Rcn3 |
| 10394471 | 1.846631 | 6.219113 | -14.5391 | 5.69E-13 | 8.77E-11 | Sdc1 |
| 10576973 | 1.812969 | 6.978967 | -14.5246 | 5.81E-13 | 8.91E-11 | Col4a1 |
| 10403584 | 1.701637 | 7.205006 | -14.4619 | 6.35E-13 | 9.68E-11 | Nid1 |
| 10588037 | 1.587005 | 8.202167 | -14.4535 | 6.43E-13 | 9.75E-11 | Rbp1 |
| 10474419 | 2.242547 | 6.119426 | -14.4475 | 6.48E-13 | 9.78E-11 | Lgr4 |
| 10350864 | 1.890645 | 5.988724 | -14.4426 | 6.53E-13 | 9.80E-11 | Sec16b |
| 10400515 | 1.697341 | 8.071589 | -14.399 | 6.95E-13 | 1.03E-10 | Sec23a |
| 10553967 | 1.904326 | 6.967137 | -14.3985 | 6.95E-13 | 1.03E-10 | Pcsk6 |
| 10583044 | 2.610904 | 10.43212 | -14.3832 | 7.10E-13 | 1.05E-10 | Mmp13 |
| 10589889 | 2.122951 | 7.828466 | -14.3827 | 7.11E-13 | 1.05E-10 | Glb1 |
| 10350848 | 1.281601 | 7.469955 | -14.3748 | 7.19E-13 | 1.05E-10 | 2810025M15Rik |
| 10470283 | 1.310442 | 7.121565 | -14.37 | 7.24E-13 | 1.05E-10 | Egfl7 |
| 10444016 | -2.78139 | 7.589017 | 14.36307 | 7.31E-13 | 1.06E-10 | Pram1 |
| 10507273 | 1.781947 | 6.236342 | -14.3522 | 7.42E-13 | 1.07E-10 | Pik3r3 |
| 10484503 | 1.827876 | 5.960744 | -14.3517 | 7.43E-13 | 1.07E-10 | Lrrc55 |
| 10386070 | 1.27541 | 9.543367 | -14.3198 | 7.78E-13 | 1.11E-10 | Atox1 |
| 10381898 | 1.976203 | 7.156632 | -14.3185 | 7.79E-13 | 1.11E-10 | Mrc2 |
| 10569618 | 1.924745 | 6.220208 | -14.3097 | 7.89E-13 | 1.11E-10 | Ano1 |
| 10426611 | 1.545403 | 6.044613 | -14.3018 | 7.98E-13 | 1.12E-10 | Cacnb3 |
| 10573319 | 1.523244 | 5.956294 | -14.2984 | 8.02E-13 | 1.12E-10 | Podnl1 |
| 10575993 | -1.23355 | 7.331911 | 14.29043 | 8.11E-13 | 1.13E-10 | 6430548M08Rik |
| 10576581 | 2.860911 | 6.979577 | -14.2786 | 8.25E-13 | 1.14E-10 | Kcnk1 |
| 10595402 | 1.81946 | 7.30291 | -14.2771 | 8.27E-13 | 1.14E-10 | Fam46a |
| 10391119 | 2.219996 | 6.158414 | -14.2714 | 8.33E-13 | 1.14E-10 | Leprel4 |
| 10467768 | 2.037427 | 5.450873 | -14.2652 | 8.41E-13 | 1.15E-10 | Loxl4 |
| 10586781 | 1.907304 | 7.071665 | -14.259 | 8.48E-13 | 1.15E-10 | Myo1e |
| 10402708 | 2.432021 | 9.746134 | -14.2483 | 8.61E-13 | 1.17E-10 | Ckb |
| 10375065 | 1.963509 | 6.524915 | -14.23 | 8.84E-13 | 1.19E-10 | Sh3pxd2b |
| 10351551 | 1.567281 | 5.552109 | -14.2188 | 8.99E-13 | 1.20E-10 | Adamts4 |
| 10556426 | 2.10674 | 7.723735 | -14.1857 | 9.43E-13 | 1.26E-10 | Parva |
| 10578829 | 1.713385 | 7.385092 | -14.1664 | 9.69E-13 | 1.29E-10 | Palld |
| 10571840 | 1.888428 | 8.976025 | -14.1576 | 9.82E-13 | 1.29E-10 | Hpgd |
| 10536494 | 1.931813 | 7.265288 | -14.1558 | 9.84E-13 | 1.29E-10 | Cav2 |
| 10572146 | 1.414092 | 9.395329 | -14.1515 | 9.90E-13 | 1.30E-10 | Atp6v1b2 |
| 10443470 | -1.87937 | 6.868889 | 14.14781 | 9.96E-13 | 1.30E-10 | Rab44 |
| 10537509 | -1.91515 | 5.877879 | 14.1342 | 1.02E-12 | 1.32E-10 | Mgam |
| 10546567 | 1.432204 | 6.706734 | -14.125 | 1.03E-12 | 1.32E-10 | Eogt |
| 10591112 | 1.990854 | 4.523138 | -14.1247 | 1.03E-12 | 1.32E-10 | Fat3 |
| 10490802 | 1.188976 | 5.696168 | -14.1029 | 1.06E-12 | 1.36E-10 | Zc2hc1a |
| 10579636 | -1.36544 | 6.06981 | 14.08785 | 1.09E-12 | 1.38E-10 | Cyp4f18 |
| 10567995 | 2.432252 | 9.328214 | -14.0828 | 1.09E-12 | 1.39E-10 | Nupr1 |
| 10375751 | 2.070569 | 7.864625 | -14.0586 | 1.13E-12 | 1.43E-10 | Adamts2 |
| 10484283 | 1.6835 | 5.51615 | -14.0582 | 1.13E-12 | 1.43E-10 | Pde1a |
| 10584561 | 1.922345 | 7.63429 | -14.0499 | 1.15E-12 | 1.44E-10 | Clmp |
| 10517587 | 2.737361 | 8.44223 | -14.036 | 1.17E-12 | 1.46E-10 | Alpl |
| 10578222 | 1.311692 | 6.954784 | -14.0184 | 1.20E-12 | 1.49E-10 | Dlc1 |
| 10546430 | 2.05715 | 6.31115 | -13.9861 | 1.26E-12 | 1.55E-10 | Adamts9 |
| 10462507 | 1.414469 | 7.2038 | -13.9816 | 1.27E-12 | 1.55E-10 | Papss2 |
| 10361338 | -2.38919 | 5.749397 | 13.96617 | 1.30E-12 | 1.58E-10 | Ipcef1 |
| 10356520 | 2.629616 | 8.341577 | -13.9461 | 1.33E-12 | 1.62E-10 | Col6a3 |
| 10538753 | 1.292626 | 5.850103 | -13.9369 | 1.35E-12 | 1.64E-10 | Gm15534 |
| 10373027 | 1.259231 | 7.913941 | -13.9282 | 1.37E-12 | 1.65E-10 | Tspan31 |
| 10396170 | 1.840244 | 7.009691 | -13.9264 | 1.37E-12 | 1.65E-10 | Frmd6 |
| 10432243 | 2.180669 | 6.872214 | -13.9125 | 1.40E-12 | 1.67E-10 | Fkbp11 |
| 10365716 | 1.6687 | 7.248625 | -13.9121 | 1.40E-12 | 1.67E-10 | Ikbip |
| 10375079 | 1.315874 | 7.33113 | -13.9097 | 1.41E-12 | 1.67E-10 | Ubtd2 |
| 10425207 | 1.31599 | 8.906359 | -13.9065 | 1.41E-12 | 1.67E-10 | H1f0 |
| 10530692 | 1.701042 | 6.575956 | -13.8931 | 1.44E-12 | 1.70E-10 | Kdr |
| 10396840 | -1.54886 | 5.379057 | 13.88689 | 1.46E-12 | 1.70E-10 | Rdh12 |
| 10496789 | 1.301383 | 5.51666 | -13.8858 | 1.46E-12 | 1.70E-10 | Lpar3 |
| 10553897 | 1.125587 | 5.93556 | -13.8828 | 1.46E-12 | 1.70E-10 | Mtmr10 |
| 10456988 | 1.847779 | 5.411734 | -13.8825 | 1.47E-12 | 1.70E-10 | Pard6g |
| 10346843 | 2.246822 | 8.279828 | -13.8677 | 1.50E-12 | 1.73E-10 | Nrp2 |
| 10412078 | -1.48972 | 6.393259 | 13.86223 | 1.51E-12 | 1.73E-10 | Gapt |
| 10600150 | 1.183495 | 7.491774 | -13.8463 | 1.54E-12 | 1.77E-10 | Zfp275 |
| 10368495 | 2.013044 | 6.478192 | -13.8438 | 1.55E-12 | 1.77E-10 | Rspo3 |
| 10495054 | 1.730478 | 7.394765 | -13.8338 | 1.57E-12 | 1.79E-10 | Rhoc |
| 10408450 | 1.6403 | 6.540238 | -13.8274 | 1.59E-12 | 1.80E-10 | Sox4 |
| 10366476 | 1.409777 | 7.002892 | -13.8192 | 1.61E-12 | 1.81E-10 | Ptprb |
| 10424543 | 2.079565 | 7.203565 | -13.8052 | 1.64E-12 | 1.83E-10 | Wisp1 |
| 10494445 | 1.788749 | 7.007686 | -13.7999 | 1.65E-12 | 1.84E-10 | Lix1l |
| 10412466 | 1.047647 | 7.349276 | -13.7968 | 1.66E-12 | 1.84E-10 | Hmgcs1 |
| 10515007 | 2.31037 | 7.428765 | -13.7957 | 1.66E-12 | 1.84E-10 | Gpx7 |
| 10546434 | 2.346067 | 6.698784 | -13.7885 | 1.68E-12 | 1.85E-10 | Adamts9 |
| 10571467 | 3.017312 | 6.844296 | -13.7832 | 1.70E-12 | 1.85E-10 | Pdgfrl |
| 10492864 | 1.346355 | 6.77534 | -13.7828 | 1.70E-12 | 1.85E-10 | Sh3d19 |
| 10604564 | 1.733533 | 7.229989 | -13.7765 | 1.71E-12 | 1.86E-10 | Gpc4 |
| 10546450 | 2.694451 | 6.636772 | -13.7616 | 1.75E-12 | 1.89E-10 | Adamts9 |
| 10346747 | 1.427783 | 6.989459 | -13.7607 | 1.75E-12 | 1.89E-10 | Cyp20a1 |
| 10449225 | 1.271116 | 6.47684 | -13.7596 | 1.76E-12 | 1.89E-10 | Decr2 |
| 10483381 | 1.583486 | 7.062505 | -13.7564 | 1.76E-12 | 1.89E-10 | Stk39 |
| 10451004 | 1.343119 | 8.92034 | -13.7529 | 1.77E-12 | 1.89E-10 | Cd2ap |
| 10592266 | 2.381611 | 7.341856 | -13.7417 | 1.80E-12 | 1.92E-10 | Slc37a2 |
| 10401519 | 1.090955 | 10.17958 | -13.7244 | 1.85E-12 | 1.95E-10 | Npc2 |
| 10600936 | 1.820121 | 6.605013 | -13.6945 | 1.93E-12 | 2.03E-10 | Efnb1 |
| 10494262 | 2.9362 | 10.31819 | -13.6699 | 2.00E-12 | 2.09E-10 | Ctsk |
| 10372342 | 1.798836 | 6.196821 | -13.663 | 2.03E-12 | 2.10E-10 | Nav3 |
| 10420596 | 1.384958 | 7.050263 | -13.6627 | 2.03E-12 | 2.10E-10 | Tnfrsf19 |
| 10395320 | 2.52161 | 6.655868 | -13.6517 | 2.06E-12 | 2.13E-10 | Twist1 |
| 10396402 | 1.51445 | 6.846486 | -13.6477 | 2.07E-12 | 2.13E-10 | Prkch |
| 10488195 | 1.52435 | 9.091736 | -13.6475 | 2.07E-12 | 2.13E-10 | Rrbp1 |
| 10591090 | 2.129983 | 6.90736 | -13.6405 | 2.09E-12 | 2.14E-10 | Fat3 |
| 10590325 | 1.106184 | 9.567619 | -13.6375 | 2.10E-12 | 2.14E-10 | Ctnnb1 |
| 10355403 | 1.877347 | 10.08854 | -13.6349 | 2.11E-12 | 2.14E-10 | Fn1 |
| 10544462 | 1.608584 | 5.751465 | -13.6097 | 2.19E-12 | 2.22E-10 | Fam115a |
| 10406877 | 1.904453 | 6.811623 | -13.5926 | 2.25E-12 | 2.27E-10 | Serf1 |
| 10407126 | 2.028135 | 6.587 | -13.5904 | 2.26E-12 | 2.27E-10 | Plk2 |
| 10423109 | 1.86532 | 5.85983 | -13.5835 | 2.28E-12 | 2.28E-10 | Adamts12 |
| 10413932 | 1.320908 | 7.379346 | -13.5797 | 2.29E-12 | 2.29E-10 | Vstm4 |
| 10473880 | 1.447199 | 7.237169 | -13.5538 | 2.38E-12 | 2.37E-10 | Lrp4 |
| 10445774 | -2.38106 | 7.82654 | 13.5472 | 2.41E-12 | 2.38E-10 | B430306N03Rik |
| 10510700 | 1.687371 | 5.567615 | -13.542 | 2.43E-12 | 2.39E-10 | Gpr153 |
| 10505276 | 1.469196 | 7.645009 | -13.5417 | 2.43E-12 | 2.39E-10 | Slc31a1 |
| 10439566 | 1.387622 | 8.584929 | -13.5073 | 2.56E-12 | 2.50E-10 | Atp6v1a |
| 10598976 | 3.043565 | 8.907398 | -13.5035 | 2.57E-12 | 2.51E-10 | Timp1 |
| 10453604 | 2.370995 | 7.754181 | -13.4918 | 2.62E-12 | 2.55E-10 | Bambi |
| 10369388 | 2.190037 | 6.918476 | -13.4769 | 2.67E-12 | 2.59E-10 | Unc5b |
| 10586591 | 2.051743 | 5.747256 | -13.4699 | 2.70E-12 | 2.61E-10 | Car12 |
| 10441864 | 1.306078 | 7.227707 | -13.4525 | 2.77E-12 | 2.67E-10 | Mllt4 |
| 10564539 | -2.1639 | 5.518375 | 13.43984 | 2.83E-12 | 2.72E-10 | Mctp2 |
| 10591139 | 1.254788 | 4.82336 | -13.4197 | 2.91E-12 | 2.79E-10 | Naalad2 |
| 10570957 | 1.330538 | 5.869742 | -13.4052 | 2.98E-12 | 2.84E-10 | Sfrp1 |
| 10455092 | 1.053094 | 4.058693 | -13.4031 | 2.99E-12 | 2.84E-10 | Pcdhb12 |
| 10505132 | 1.590781 | 5.68932 | -13.3947 | 3.03E-12 | 2.87E-10 | Akap2 |
| 10344981 | 1.953803 | 5.1905 | -13.3874 | 3.06E-12 | 2.89E-10 | Pi15 |
| 10461057 | 1.539408 | 6.194884 | -13.3694 | 3.14E-12 | 2.96E-10 | Rcor2 |
| 10440091 | 2.333362 | 8.089533 | -13.3674 | 3.15E-12 | 2.96E-10 | Col8a1 |
| 10415857 | 1.241574 | 5.511001 | -13.365 | 3.17E-12 | 2.96E-10 | Fam167a |
| 10586933 | 1.178075 | 10.31828 | -13.3401 | 3.29E-12 | 3.06E-10 | Nedd4 |
| 10528385 | -1.14374 | 5.443254 | 13.33361 | 3.32E-12 | 3.08E-10 | Reln |
| 10416974 | 1.542684 | 7.106975 | -13.309 | 3.45E-12 | 3.18E-10 | Gpc6 |
| 10463476 | 3.122197 | 6.94862 | -13.3044 | 3.47E-12 | 3.19E-10 | Kazald1 |
| 10478692 | 1.62854 | 6.071884 | -13.3 | 3.49E-12 | 3.20E-10 | Slc2a10 |
| 10542691 | -1.67614 | 7.082093 | 13.29953 | 3.50E-12 | 3.20E-10 | Lrmp |
| 10590031 | 1.563531 | 6.245513 | -13.2889 | 3.55E-12 | 3.24E-10 | Itga9 |
| 10369252 | 1.53034 | 7.115313 | -13.2872 | 3.56E-12 | 3.24E-10 | 44449 |
| 10497920 | 1.106289 | 6.363873 | -13.2706 | 3.65E-12 | 3.31E-10 | Ankrd50 |
| 10476321 | 2.308761 | 5.855558 | -13.2664 | 3.68E-12 | 3.32E-10 | Prn |
| 10459481 | 1.528656 | 8.140676 | -13.215 | 3.97E-12 | 3.58E-10 | Lman1 |
| 10349968 | -2.59121 | 7.276054 | 13.17995 | 4.19E-12 | 3.76E-10 | Chil1 |
| 10410931 | 2.683375 | 7.47228 | -13.1775 | 4.21E-12 | 3.77E-10 | Vcan |
| 10544885 | 2.700318 | 7.000704 | -13.1707 | 4.25E-12 | 3.79E-10 | Fkbp14 |
| 10522503 | 1.884412 | 7.673137 | -13.1688 | 4.27E-12 | 3.79E-10 | Pdgfra |
| 10576639 | 1.176142 | 7.62992 | -13.1574 | 4.34E-12 | 3.85E-10 | Nrp1 |
| 10517141 | -1.05494 | 10.67596 | 13.1481 | 4.40E-12 | 3.88E-10 | Hmgn2 |
| 10529824 | -1.76383 | 5.11944 | 13.13885 | 4.47E-12 | 3.92E-10 | Prom1 |
| 10588243 | 1.82387 | 8.319646 | -13.1324 | 4.51E-12 | 3.95E-10 | Ryk |
| 10554521 | 1.069982 | 6.936242 | -13.1277 | 4.54E-12 | 3.97E-10 | Pde8a |
| 10558150 | 2.074493 | 6.957503 | -13.1187 | 4.61E-12 | 3.98E-10 | Htra1 |
| 10523717 | 1.936831 | 11.12852 | -13.1186 | 4.61E-12 | 3.98E-10 | Spp1 |
| 10352867 | 1.734302 | 7.031548 | -13.1181 | 4.61E-12 | 3.98E-10 | Plxna2 |
| 10583100 | -2.71751 | 9.48833 | 13.1128 | 4.65E-12 | 3.99E-10 | Mmp8 |
| 10468527 | -1.48004 | 6.122353 | 13.11248 | 4.65E-12 | 3.99E-10 | 5830416P10Rik |
| 10594066 | 1.450627 | 7.725022 | -13.1014 | 4.73E-12 | 4.04E-10 | Loxl1 |
| 10367919 | -1.59762 | 6.171974 | 13.08629 | 4.84E-12 | 4.11E-10 | Stx11 |
| 10602020 | 1.773255 | 6.721824 | -13.0719 | 4.95E-12 | 4.19E-10 | Tbc1d8b |
| 10467766 | 2.689865 | 6.134013 | -13.0702 | 4.96E-12 | 4.19E-10 | Loxl4 |
| 10388430 | 2.214126 | 10.54873 | -13.0552 | 5.08E-12 | 4.27E-10 | Serpinf1 |
| 10386020 | 1.905598 | 7.112034 | -13.0504 | 5.12E-12 | 4.28E-10 | Slc36a2 |
| 10549222 | 1.388519 | 6.122856 | -13.0205 | 5.36E-12 | 4.46E-10 | Bcat1 |
| 10523845 | 1.195899 | 4.024001 | -13.0198 | 5.36E-12 | 4.46E-10 | Ephx4 |
| 10389087 | -1.22101 | 6.876799 | 13.0165 | 5.39E-12 | 4.46E-10 | Rffl |
| 10550274 | 1.194388 | 6.103522 | -13.0147 | 5.40E-12 | 4.46E-10 | Meis3 |
| 10579812 | 1.690773 | 6.576029 | -13.0144 | 5.41E-12 | 4.46E-10 | Ednra |
| 10518408 | 1.734178 | 7.071624 | -13.0075 | 5.47E-12 | 4.49E-10 | Plod1 |
| 10460541 | 1.420336 | 7.289193 | -13.0054 | 5.48E-12 | 4.49E-10 | Cd248 |
| 10470462 | 1.851948 | 6.905288 | -13.0051 | 5.49E-12 | 4.49E-10 | Col5a1 |
| 10438017 | -1.31518 | 6.749472 | 13.0019 | 5.51E-12 | 4.50E-10 | Fgd4 |
| 10493798 | 1.785055 | 7.95255 | -12.9935 | 5.58E-12 | 4.54E-10 | S100a16 |
| 10344837 | 1.306382 | 6.004097 | -12.9919 | 5.60E-12 | 4.54E-10 | Prex2 |
| 10406254 | 1.138412 | 8.633226 | -12.9619 | 5.86E-12 | 4.72E-10 | Ell2 |
| 10585778 | 1.508509 | 7.549443 | -12.9602 | 5.88E-12 | 4.72E-10 | Sema7a |
| 10493245 | 1.633822 | 5.425938 | -12.9493 | 5.98E-12 | 4.79E-10 | Mex3a |
| 10540275 | 2.51578 | 6.577581 | -12.9461 | 6.01E-12 | 4.80E-10 | Gxylt2 |
| 10573924 | 2.561389 | 9.205508 | -12.9445 | 6.02E-12 | 4.80E-10 | Mmp2 |
| 10472965 | 1.974367 | 6.02495 | -12.9415 | 6.05E-12 | 4.80E-10 | Hoxd8 |
| 10517791 | -2.21916 | 5.636422 | 12.93579 | 6.11E-12 | 4.83E-10 | Padi4 |
| 10491970 | 1.28301 | 6.077071 | -12.9335 | 6.13E-12 | 4.83E-10 | Lhfp |
| 10560282 | 1.249101 | 7.342364 | -12.9323 | 6.14E-12 | 4.83E-10 | Arhgap35 |
| 10592535 | -1.66793 | 7.636938 | 12.92934 | 6.17E-12 | 4.84E-10 | Sorl1 |
| 10509163 | 2.006546 | 8.718717 | -12.9279 | 6.18E-12 | 4.84E-10 | Id3 |
| 10368289 | 1.687297 | 8.253236 | -12.9208 | 6.25E-12 | 4.88E-10 | Enpp1 |
| 10422348 | 1.711197 | 5.964964 | -12.9179 | 6.28E-12 | 4.89E-10 | Uggt2 |
| 10408629 | 1.88825 | 6.169895 | -12.8527 | 6.95E-12 | 5.38E-10 | Pxdc1 |
| 10512949 | 1.29567 | 7.283409 | -12.8504 | 6.97E-12 | 5.39E-10 | Abca1 |
| 10607752 | -1.71803 | 4.862898 | 12.84796 | 7.00E-12 | 5.39E-10 | Bmx |
| 10485198 | 1.797468 | 5.965453 | -12.8435 | 7.05E-12 | 5.42E-10 | Tspan18 |
| 10558345 | 1.244092 | 6.50155 | -12.8371 | 7.12E-12 | 5.45E-10 | Dock1 |
| 10439218 | 1.707593 | 6.284052 | -12.8364 | 7.13E-12 | 5.45E-10 | Pdia5 |
| 10596303 | -1.5623 | 5.244724 | 12.81214 | 7.40E-12 | 5.64E-10 | Acpp |
| 10513208 | 1.093275 | 6.566131 | -12.7963 | 7.59E-12 | 5.77E-10 | Svep1 |
| 10519913 | 1.4424 | 6.296708 | -12.7831 | 7.75E-12 | 5.87E-10 | Magi2 |
| 10514902 | 1.214488 | 5.99926 | -12.7791 | 7.79E-12 | 5.89E-10 | Lrrc42 |
| 10522217 | 1.811066 | 6.721071 | -12.7719 | 7.88E-12 | 5.95E-10 | Limch1 |
| 10545974 | 2.102798 | 8.354511 | -12.759 | 8.04E-12 | 6.05E-10 | Antxr1 |
| 10433887 | 1.216927 | 5.137474 | -12.7452 | 8.22E-12 | 6.17E-10 | Pkp2 |
| 10349051 | 1.423773 | 6.050982 | -12.7317 | 8.40E-12 | 6.27E-10 | Tnfrsf11a |
| 10451061 | 1.164521 | 8.437247 | -12.7268 | 8.46E-12 | 6.30E-10 | Runx2 |
| 10372177 | 2.477026 | 5.905604 | -12.7233 | 8.51E-12 | 6.32E-10 | Tmtc2 |
| 10402705 | 1.961591 | 6.206156 | -12.7209 | 8.54E-12 | 6.33E-10 | Gm266 |
| 10482929 | -1.65499 | 6.242896 | 12.70883 | 8.70E-12 | 6.43E-10 | Ly75 |
| 10387699 | -1.98281 | 6.502606 | 12.70444 | 8.76E-12 | 6.44E-10 | Acap1 |
| 10509014 | -1.00863 | 9.242674 | 12.70435 | 8.76E-12 | 6.44E-10 | Rsrp1 |
| 10490777 | 1.92963 | 7.559653 | -12.6984 | 8.85E-12 | 6.49E-10 | Zfhx4 |
| 10453747 | 2.080182 | 7.89271 | -12.6938 | 8.91E-12 | 6.52E-10 | Colec12 |
| 10603583 | 1.938377 | 6.498824 | -12.686 | 9.02E-12 | 6.56E-10 | Srpx |
| 10458663 | 1.940231 | 7.325765 | -12.685 | 9.03E-12 | 6.56E-10 | Dpysl3 |
| 10447190 | 1.13255 | 6.155564 | -12.6838 | 9.05E-12 | 6.56E-10 | Plekhh2 |
| 10568361 | 1.154003 | 7.480093 | -12.6835 | 9.06E-12 | 6.56E-10 | Yipf5 |
| 10407511 | -1.30971 | 10.34004 | 12.65884 | 9.41E-12 | 6.80E-10 | LOC102640615 |
| 10562709 | -2.11666 | 8.401739 | 12.64874 | 9.57E-12 | 6.89E-10 | Cd33 |
| 10555118 | 1.113757 | 7.726567 | -12.6302 | 9.85E-12 | 7.08E-10 | Pak1 |
| 10511429 | 1.696691 | 5.644259 | -12.6225 | 9.97E-12 | 7.15E-10 | Car8 |
| 10394258 | 1.454674 | 6.527677 | -12.6045 | 1.03E-11 | 7.33E-10 | Adcy3 |
| 10393887 | 1.77368 | 6.340788 | -12.6027 | 1.03E-11 | 7.33E-10 | Pycr1 |
| 10401852 | -1.54576 | 7.304632 | 12.60118 | 1.03E-11 | 7.33E-10 | Cep128 |
| 10535902 | -1.32831 | 4.122148 | 12.59224 | 1.05E-11 | 7.39E-10 | n-R5s180 |
| 10478415 | 1.416737 | 7.129106 | -12.5794 | 1.07E-11 | 7.50E-10 | Wisp2 |
| 10435271 | 1.349362 | 8.207273 | -12.5697 | 1.08E-11 | 7.58E-10 | Heg1 |
| 10597575 | 1.574984 | 6.143468 | -12.5696 | 1.08E-11 | 7.58E-10 | Plcd1 |
| 10493692 | 1.233308 | 6.645718 | -12.5648 | 1.09E-11 | 7.62E-10 | Rab13 |
| 10509246 | 1.667542 | 7.250042 | -12.5621 | 1.10E-11 | 7.64E-10 | Luzp1 |
| 10534202 | -1.92717 | 8.269133 | 12.55915 | 1.10E-11 | 7.65E-10 | Ncf1 |
| 10553131 | 1.23088 | 8.927593 | -12.5577 | 1.10E-11 | 7.65E-10 | Kdelr1 |
| 10367282 | 1.165164 | 6.992127 | -12.5526 | 1.11E-11 | 7.70E-10 | Cnpy2 |
| 10422321 | 1.203204 | 5.841492 | -12.5457 | 1.13E-11 | 7.75E-10 | Dzip1 |
| 10500666 | 1.623983 | 6.289752 | -12.5452 | 1.13E-11 | 7.75E-10 | Ptgfrn |
| 10409502 | -1.18864 | 6.970294 | 12.54375 | 1.13E-11 | 7.75E-10 | Dok3 |
| 10423548 | 1.960351 | 8.052954 | -12.5423 | 1.13E-11 | 7.75E-10 | Sdc2 |
| 10500808 | 1.922255 | 8.663049 | -12.5301 | 1.15E-11 | 7.87E-10 | Olfml3 |
| 10406334 | -2.12604 | 6.551212 | 12.51665 | 1.18E-11 | 8.01E-10 | Mctp1 |
| 10598403 | 2.075273 | 8.169368 | -12.4881 | 1.23E-11 | 8.34E-10 | Praf2 |
| 10547869 | 1.569334 | 6.780177 | -12.4869 | 1.24E-11 | 8.34E-10 | Leprel2 |
| 10562576 | 1.675557 | 6.998454 | -12.4855 | 1.24E-11 | 8.34E-10 | Plekhf1 |
| 10501164 | 1.449405 | 7.456365 | -12.4847 | 1.24E-11 | 8.34E-10 | Csf1 |
| 10485070 | 1.516233 | 6.820978 | -12.4845 | 1.24E-11 | 8.34E-10 | Mdk |
| 10530287 | 1.604178 | 7.031361 | -12.478 | 1.25E-11 | 8.37E-10 | Apbb2 |
| 10397416 | 1.311695 | 7.216783 | -12.4471 | 1.32E-11 | 8.75E-10 | Ift43 |
| 10405179 | 1.785615 | 7.259308 | -12.4385 | 1.34E-11 | 8.85E-10 | S1pr3 |
| 10407985 | -2.06268 | 6.200565 | 12.43092 | 1.35E-11 | 8.93E-10 | Gpr141 |
| 10565255 | 2.008362 | 6.07358 | -12.4289 | 1.36E-11 | 8.94E-10 | Cemip |
| 10412207 | 2.467574 | 8.859082 | -12.4171 | 1.38E-11 | 9.09E-10 | Gpx8 |
| 10384223 | 1.799705 | 7.295373 | -12.3953 | 1.43E-11 | 9.39E-10 | Igfbp3 |
| 10428579 | 1.701346 | 7.845446 | -12.3806 | 1.47E-11 | 9.58E-10 | Ext1 |
| 10490903 | 1.254508 | 6.264981 | -12.3803 | 1.47E-11 | 9.58E-10 | Car13 |
| 10591120 | 2.059592 | 6.321917 | -12.3647 | 1.50E-11 | 9.78E-10 | Fat3 |
| 10345715 | 1.386823 | 8.089184 | -12.361 | 1.51E-11 | 9.81E-10 | Map4k4 |
| 10579860 | 1.276138 | 7.035489 | -12.3599 | 1.51E-11 | 9.81E-10 | Smad1 |
| 10365601 | 1.19882 | 7.667644 | -12.3559 | 1.52E-11 | 9.85E-10 | Gnptab |
| 10472860 | 1.194972 | 6.163796 | -12.3521 | 1.53E-11 | 9.85E-10 | Rapgef4 |
| 10440258 | 2.339612 | 6.125306 | -12.3512 | 1.54E-11 | 9.85E-10 | Epha3 |
| 10496656 | 1.900285 | 6.509476 | -12.3512 | 1.54E-11 | 9.85E-10 | Col24a1 |
| 10364375 | 1.583456 | 9.296079 | -12.3501 | 1.54E-11 | 9.85E-10 | Cstb |
| 10590306 | 1.744953 | 6.665709 | -12.3475 | 1.55E-11 | 9.86E-10 | Entpd3 |
| 10403604 | -1.40594 | 8.212136 | 12.34691 | 1.55E-11 | 9.86E-10 | Lyst |
| 10579659 | -1.07047 | 10.71335 | 12.33596 | 1.57E-11 | 1.00E-09 | Hmgn2 |
| 10361869 | 1.211912 | 6.509274 | -12.3331 | 1.58E-11 | 1.00E-09 | Nhsl1 |
| 10452613 | 1.585072 | 5.16483 | -12.3331 | 1.58E-11 | 1.00E-09 | Arhgap28 |
| 10525989 | 1.708917 | 6.504407 | -12.3221 | 1.61E-11 | 1.01E-09 | Gpr133 |
| 10532741 | 2.143043 | 7.896053 | -12.321 | 1.61E-11 | 1.01E-09 | Tmem119 |
| 10561008 | -2.3218 | 7.673874 | 12.32076 | 1.61E-11 | 1.01E-09 | Ceacam1 |
| 10556553 | 1.718362 | 6.03439 | -12.3126 | 1.63E-11 | 1.03E-09 | Insc |
| 10578477 | 1.29937 | 5.93068 | -12.3085 | 1.65E-11 | 1.03E-09 | Fam149a |
| 10423520 | 1.766792 | 7.13798 | -12.2957 | 1.68E-11 | 1.05E-09 | Sema5a |
| 10492689 | 1.956833 | 6.795855 | -12.2868 | 1.70E-11 | 1.06E-09 | Pdgfc |
| 10548899 | 1.967401 | 5.886144 | -12.2759 | 1.73E-11 | 1.08E-09 | Rerg |
| 10591773 | -1.05882 | 10.69714 | 12.2549 | 1.79E-11 | 1.11E-09 | Hmgn2 |
| 10566877 | 1.27158 | 7.434908 | -12.2487 | 1.81E-11 | 1.12E-09 | Sbf2 |
| 10418506 | 1.214253 | 6.20741 | -12.2465 | 1.82E-11 | 1.12E-09 | Stab1 |
| 10431711 | 1.818927 | 6.388424 | -12.2412 | 1.83E-11 | 1.13E-09 | Slc2a13 |
| 10542953 | 1.329637 | 6.262703 | -12.2352 | 1.85E-11 | 1.14E-09 | Tfpi2 |
| 10389222 | -1.80689 | 8.515205 | 12.22023 | 1.90E-11 | 1.16E-09 | Ccl6 |
| 10557177 | -1.20433 | 7.6809 | 12.18911 | 2.00E-11 | 1.22E-09 | Prkcb |
| 10484927 | 1.510714 | 7.484017 | -12.1887 | 2.00E-11 | 1.22E-09 | Slc39a13 |
| 10409464 | 1.268712 | 6.45905 | -12.1665 | 2.07E-11 | 1.26E-09 | Dbn1 |
| 10400844 | -2.11678 | 8.313976 | 12.15656 | 2.10E-11 | 1.28E-09 | Pygl |
| 10454369 | 1.444313 | 6.034982 | -12.1429 | 2.15E-11 | 1.30E-09 | Fhod3 |
| 10505008 | 1.209448 | 8.363711 | -12.1424 | 2.15E-11 | 1.30E-09 | Slc44a1 |
| 10379636 | -3.0731 | 7.86836 | 12.13971 | 2.16E-11 | 1.30E-09 | Slfn4 |
| 10554005 | 1.435399 | 8.678731 | -12.1392 | 2.16E-11 | 1.30E-09 | Vimp |
| 10545958 | 1.401292 | 7.873807 | -12.1347 | 2.18E-11 | 1.31E-09 | Anxa4 |
| 10455123 | 1.244993 | 4.017219 | -12.1337 | 2.18E-11 | 1.31E-09 | Pcdhb19 |
| 10472240 | 1.184537 | 6.140909 | -12.1261 | 2.21E-11 | 1.32E-09 | Tanc1 |
| 10585338 | 1.507769 | 5.658179 | -12.1224 | 2.22E-11 | 1.33E-09 | Kdelc2 |
| 10523701 | 2.219434 | 10.93678 | -12.1191 | 2.24E-11 | 1.33E-09 | Ibsp |
| 10496519 | 1.779716 | 5.366144 | -12.1154 | 2.25E-11 | 1.34E-09 | Unc5c |
| 10537146 | 1.839407 | 5.65937 | -12.1106 | 2.27E-11 | 1.34E-09 | Akr1b8 |
| 10368317 | 1.568457 | 5.555151 | -12.1047 | 2.29E-11 | 1.35E-09 | Enpp3 |
| 10550994 | -2.4006 | 6.273095 | 12.10448 | 2.29E-11 | 1.35E-09 | Ceacam10 |
| 10568436 | 1.456295 | 7.53921 | -12.0959 | 2.32E-11 | 1.37E-09 | Fgfr2 |
| 10354309 | 2.987658 | 7.981812 | -12.0942 | 2.33E-11 | 1.37E-09 | Col5a2 |
| 10424119 | 1.617185 | 6.259523 | -12.0935 | 2.33E-11 | 1.37E-09 | Nov |
| 10539119 | 1.407083 | 6.488808 | -12.0869 | 2.36E-11 | 1.38E-09 | Ggcx |
| 10595560 | 1.210634 | 5.346872 | -12.0838 | 2.37E-11 | 1.38E-09 | Tbx18 |
| 10431802 | 1.113493 | 8.568755 | -12.07 | 2.42E-11 | 1.41E-09 | Twf1 |
| 10571774 | 1.016192 | 7.469348 | -12.068 | 2.43E-11 | 1.41E-09 | Aga |
| 10570068 | 1.499009 | 6.525608 | -12.0667 | 2.44E-11 | 1.41E-09 | Col4a2 |
| 10456046 | 1.630241 | 7.208987 | -12.0658 | 2.44E-11 | 1.41E-09 | Pdgfrb |
| 10441178 | -2.37908 | 7.256483 | 12.06224 | 2.45E-11 | 1.42E-09 | Itgb2l |
| 10548701 | 1.000407 | 7.565034 | -12.0612 | 2.46E-11 | 1.42E-09 | Lrp6 |
| 10455108 | 1.667717 | 5.128009 | -12.0553 | 2.48E-11 | 1.43E-09 | Pcdhb16 |
| 10380501 | 1.497674 | 7.116898 | -12.0449 | 2.52E-11 | 1.45E-09 | Dlx3 |
| 10593219 | 1.58504 | 6.888331 | -12.0217 | 2.62E-11 | 1.50E-09 | Nnmt |
| 10481186 | 1.050659 | 5.858869 | -12.0217 | 2.62E-11 | 1.50E-09 | Sardh |
| 10465833 | 1.765058 | 6.457434 | -12.0205 | 2.63E-11 | 1.50E-09 | Rom1 |
| 10474619 | 1.291372 | 6.490838 | -12.016 | 2.65E-11 | 1.51E-09 | Fmn1 |
| 10430145 | 1.630557 | 6.804669 | -12.0101 | 2.67E-11 | 1.52E-09 | Rbfox2 |
| 10494565 | -1.36382 | 6.808562 | 12.00898 | 2.68E-11 | 1.52E-09 | Fmo5 |
| 10370210 | 2.2543 | 7.638303 | -12.0046 | 2.70E-11 | 1.53E-09 | Col6a1 |
| 10423836 | 3.484306 | 7.985852 | -12.0038 | 2.70E-11 | 1.53E-09 | Cthrc1 |
| 10403466 | 1.144074 | 6.016239 | -11.9958 | 2.73E-11 | 1.54E-09 | Dip2c |
| 10591127 | 2.36288 | 6.580688 | -11.993 | 2.75E-11 | 1.54E-09 | Fat3 |
| 10523579 | 1.359029 | 6.676791 | -11.9884 | 2.77E-11 | 1.55E-09 | Arhgap24 |
| 10601778 | 1.390108 | 6.936128 | -11.9737 | 2.84E-11 | 1.59E-09 | Armcx3 |
| 10565794 | 2.211797 | 8.647257 | -11.968 | 2.86E-11 | 1.60E-09 | Serpinh1 |
| 10570516 | 1.09928 | 6.217894 | -11.967 | 2.87E-11 | 1.60E-09 | Kbtbd11 |
| 10495449 | 3.076546 | 8.105668 | -11.9668 | 2.87E-11 | 1.60E-09 | Col11a1 |
| 10368647 | 1.229834 | 6.53496 | -11.962 | 2.89E-11 | 1.61E-09 | Dse |
| 10467115 | -2.17807 | 6.241473 | 11.95623 | 2.92E-11 | 1.62E-09 | Ankrd22 |
| 10445627 | 1.219684 | 8.501294 | -11.9434 | 2.98E-11 | 1.65E-09 | 2310039H08Rik |
| 10567580 | -1.82876 | 8.200358 | 11.94073 | 2.99E-11 | 1.65E-09 | Igsf6 |
| 10452047 | 1.49794 | 7.029745 | -11.9385 | 3.01E-11 | 1.65E-09 | Ptprs |
| 10509777 | 1.138796 | 5.874641 | -11.9339 | 3.03E-11 | 1.66E-09 | Iffo2 |
| 10433264 | 1.400514 | 7.692557 | -11.919 | 3.10E-11 | 1.70E-09 | Glis2 |
| 10530841 | 1.606831 | 9.839447 | -11.9122 | 3.14E-11 | 1.71E-09 | Igfbp7 |
| 10443463 | 1.765156 | 7.97298 | -11.9095 | 3.15E-11 | 1.72E-09 | Cdkn1a |
| 10480714 | 1.034934 | 6.678306 | -11.9083 | 3.16E-11 | 1.72E-09 | Uap1l1 |
| 10422728 | 1.699258 | 8.646182 | -11.9069 | 3.17E-11 | 1.72E-09 | Dab2 |
| 10372891 | 1.31283 | 6.148031 | -11.9016 | 3.19E-11 | 1.73E-09 | Srgap1 |
| 10418171 | 1.280455 | 7.061615 | -11.9001 | 3.20E-11 | 1.73E-09 | Zcchc24 |
| 10363118 | 1.013101 | 4.304592 | -11.8879 | 3.27E-11 | 1.76E-09 | Dcbld1 |
| 10359929 | 1.786659 | 7.434549 | -11.8873 | 3.27E-11 | 1.76E-09 | Ddr2 |
| 10449581 | 1.020712 | 8.728477 | -11.8705 | 3.36E-11 | 1.80E-09 | Mtch1 |
| 10535807 | 1.28395 | 6.561039 | -11.8641 | 3.40E-11 | 1.82E-09 | Flt1 |
| 10371230 | 1.281895 | 7.697588 | -11.8619 | 3.41E-11 | 1.82E-09 | Gna11 |
| 10560886 | -2.55516 | 8.160036 | 11.85979 | 3.42E-11 | 1.83E-09 | Cd177 |
| 10374083 | 1.839464 | 7.454414 | -11.8483 | 3.49E-11 | 1.85E-09 | Aebp1 |
| 10382376 | 1.605478 | 6.929651 | -11.8463 | 3.50E-11 | 1.86E-09 | Ttyh2 |
| 10474860 | 1.345295 | 7.14872 | -11.8361 | 3.56E-11 | 1.88E-09 | Chst14 |
| 10552380 | -1.34734 | 5.786988 | 11.83578 | 3.56E-11 | 1.88E-09 | Siglecg |
| 10396421 | 1.403776 | 8.379363 | -11.8283 | 3.61E-11 | 1.90E-09 | Hif1a |
| 10444152 | 1.972935 | 7.447159 | -11.8251 | 3.63E-11 | 1.90E-09 | Col11a2 |
| 10405343 | 1.18265 | 6.487168 | -11.8123 | 3.70E-11 | 1.94E-09 | Tspan17 |
| 10578904 | 2.776819 | 9.24697 | -11.8066 | 3.74E-11 | 1.95E-09 | Cpe |
| 10396383 | 1.161407 | 6.862692 | -11.806 | 3.74E-11 | 1.95E-09 | Slc38a6 |
| 10584674 | 1.760917 | 7.443219 | -11.7882 | 3.85E-11 | 2.00E-09 | Mcam |
| 10346321 | 2.708759 | 5.984205 | -11.7811 | 3.90E-11 | 2.02E-09 | Gm10561 |
| 10434806 | 1.870177 | 8.269734 | -11.7786 | 3.92E-11 | 2.02E-09 | Lpp |
| 10501860 | 1.608843 | 7.793218 | -11.7771 | 3.93E-11 | 2.02E-09 | Fnbp1l |
| 10520965 | 1.820955 | 6.269253 | -11.7635 | 4.02E-11 | 2.07E-09 | Yes1 |
| 10392739 | 1.720664 | 5.846458 | -11.7584 | 4.05E-11 | 2.08E-09 | Sdk2 |
| 10362115 | 1.087152 | 5.686768 | -11.7493 | 4.11E-11 | 2.10E-09 | Slc18b1 |
| 10420988 | 1.0794 | 7.533381 | -11.7488 | 4.12E-11 | 2.10E-09 | Dpysl2 |
| 10523595 | 1.660098 | 6.740961 | -11.7346 | 4.21E-11 | 2.15E-09 | Ptpn13 |
| 10424695 | 1.611212 | 8.408587 | -11.7333 | 4.22E-11 | 2.15E-09 | Gpihbp1 |
| 10604347 | 2.247189 | 6.117162 | -11.7283 | 4.26E-11 | 2.16E-09 | Smarca1 |
| 10377804 | -1.781 | 8.270531 | 11.71185 | 4.38E-11 | 2.21E-09 | Arrb2 |
| 10376889 | 1.076175 | 6.385268 | -11.7117 | 4.38E-11 | 2.21E-09 | Mmgt2 |
| 10521972 | 1.590088 | 7.056379 | -11.7017 | 4.45E-11 | 2.25E-09 | Pcdh7 |
| 10436519 | 1.866676 | 6.398459 | -11.6865 | 4.57E-11 | 2.30E-09 | Robo1 |
| 10542791 | 1.778052 | 7.703831 | -11.6861 | 4.57E-11 | 2.30E-09 | Ppfibp1 |
| 10573893 | 1.182635 | 6.828079 | -11.6854 | 4.58E-11 | 2.30E-09 | Fto |
| 10529375 | 1.275818 | 8.531686 | -11.6741 | 4.66E-11 | 2.33E-09 | Mxd4 |
| 10369844 | 1.63916 | 8.050348 | -11.674 | 4.66E-11 | 2.33E-09 | Bicc1 |
| 10574456 | -1.15885 | 6.47819 | 11.66433 | 4.74E-11 | 2.37E-09 | Cklf |
| 10363082 | -1.61185 | 8.537226 | 11.66248 | 4.75E-11 | 2.37E-09 | Lilrb4 |
| 10354168 | -1.51503 | 6.923412 | 11.65873 | 4.78E-11 | 2.38E-09 | Tbc1d8 |
| 10359034 | 1.386655 | 7.183168 | -11.6477 | 4.87E-11 | 2.42E-09 | Qsox1 |
| 10469312 | 1.579927 | 6.261874 | -11.6466 | 4.88E-11 | 2.42E-09 | Pter |
| 10571567 | 1.41032 | 7.191849 | -11.6444 | 4.90E-11 | 2.43E-09 | Sorbs2 |
| 10351140 | 1.311028 | 6.346197 | -11.6344 | 4.98E-11 | 2.46E-09 | Kifap3 |
| 10547153 | -2.24516 | 7.144485 | 11.63152 | 5.01E-11 | 2.47E-09 | Alox5 |
| 10502224 | 1.856951 | 8.621835 | -11.6142 | 5.15E-11 | 2.53E-09 | Sgms2 |
| 10363350 | 1.77495 | 6.919605 | -11.6125 | 5.17E-11 | 2.53E-09 | P4ha1 |
| 10359235 | 1.482036 | 6.306179 | -11.612 | 5.17E-11 | 2.53E-09 | Rasal2 |
| 10534940 | -1.81971 | 6.743953 | 11.61141 | 5.18E-11 | 2.53E-09 | Pilrb2 |
| 10528227 | 2.476324 | 7.363956 | -11.6053 | 5.23E-11 | 2.54E-09 | Gnai1 |
| 10408531 | 1.167227 | 7.000006 | -11.6033 | 5.25E-11 | 2.54E-09 | Gmds |
| 10385719 | 1.007705 | 7.89415 | -11.6031 | 5.25E-11 | 2.54E-09 | Sec24a |
| 10436945 | 1.534073 | 6.932072 | -11.6001 | 5.28E-11 | 2.55E-09 | Slc5a3 |
| 10581013 | 2.032938 | 9.976528 | -11.5886 | 5.38E-11 | 2.60E-09 | Cdh11 |
| 10509280 | 1.967651 | 7.79241 | -11.5844 | 5.42E-11 | 2.61E-09 | Hspg2 |
| 10546137 | -1.42364 | 6.603069 | 11.58421 | 5.42E-11 | 2.61E-09 | Abtb1 |
| 10374106 | 1.298277 | 6.994347 | -11.5609 | 5.64E-11 | 2.71E-09 | Ykt6 |
| 10527940 | 1.380994 | 6.300441 | -11.5604 | 5.64E-11 | 2.71E-09 | Cdk14 |
| 10346651 | 1.335533 | 8.206049 | -11.5584 | 5.66E-11 | 2.71E-09 | Bmpr2 |
| 10584208 | 1.021926 | 6.10092 | -11.5564 | 5.68E-11 | 2.72E-09 | Cdon |
| 10601854 | 1.402494 | 8.991007 | -11.5542 | 5.70E-11 | 2.72E-09 | Wbp5 |
| 10579772 | -1.1806 | 5.075498 | 11.54943 | 5.75E-11 | 2.74E-09 | Gm22509 |
| 10359624 | 1.655229 | 8.634591 | -11.5488 | 5.76E-11 | 2.74E-09 | Prrx1 |
| 10382502 | 1.558582 | 6.178667 | -11.5381 | 5.86E-11 | 2.78E-09 | Cdr2l |
| 10508651 | 1.75557 | 7.877716 | -11.5255 | 5.99E-11 | 2.83E-09 | Sdc3 |
| 10601768 | 1.350045 | 5.641921 | -11.5249 | 5.99E-11 | 2.83E-09 | Armcx4 |
| 10392845 | -1.86096 | 6.692457 | 11.52252 | 6.02E-11 | 2.84E-09 | Cd300lf |
| 10362245 | 1.412308 | 8.372374 | -11.5219 | 6.02E-11 | 2.84E-09 | Epb4.1l2 |
| 10421737 | 1.156637 | 6.765804 | -11.5126 | 6.12E-11 | 2.88E-09 | Tnfsf11 |
| 10361882 | 1.910373 | 6.652758 | -11.5038 | 6.21E-11 | 2.91E-09 | Nhsl1 |
| 10600169 | 1.595607 | 10.98727 | -11.4956 | 6.30E-11 | 2.95E-09 | Bgn |
| 10406905 | -1.60482 | 6.511593 | 11.49273 | 6.33E-11 | 2.96E-09 | Ccdc125 |
| 10472050 | 1.568348 | 5.24908 | -11.4871 | 6.39E-11 | 2.98E-09 | Tnfaip6 |
| 10435961 | 1.616515 | 5.502382 | -11.4866 | 6.39E-11 | 2.98E-09 | Gm10808 |
| 10584803 | 1.036885 | 7.56471 | -11.4844 | 6.42E-11 | 2.98E-09 | Ift46 |
| 10569335 | 2.254148 | 8.168723 | -11.4813 | 6.45E-11 | 2.99E-09 | H19 |
| 10478219 | 1.065925 | 6.966647 | -11.4759 | 6.51E-11 | 3.02E-09 | Plcg1 |
| 10352661 | 1.33662 | 7.1072 | -11.4671 | 6.61E-11 | 3.05E-09 | Ptpn14 |
| 10570018 | -1.37617 | 5.978541 | 11.46153 | 6.67E-11 | 3.07E-09 | Tnfsf13b |
| 10452571 | 1.564806 | 7.074727 | -11.4581 | 6.71E-11 | 3.08E-09 | Ptprm |
| 10470948 | 1.232749 | 6.13737 | -11.4446 | 6.87E-11 | 3.14E-09 | Slc39a1 |
| 10538802 | 1.901253 | 5.51618 | -11.4433 | 6.88E-11 | 3.14E-09 | Ndnf |
| 10502748 | 1.185186 | 6.078118 | -11.436 | 6.97E-11 | 3.18E-09 | Lphn2 |
| 10445268 | 1.417305 | 7.641824 | -11.4325 | 7.01E-11 | 3.19E-09 | Gpr116 |
| 10377560 | 1.382948 | 5.474133 | -11.4164 | 7.20E-11 | 3.27E-09 | Sat2 |
| 10436978 | 1.341274 | 6.295869 | -11.4088 | 7.30E-11 | 3.31E-09 | Cbr3 |
| 10483865 | 2.650524 | 8.573903 | -11.3975 | 7.44E-11 | 3.37E-09 | Fkbp7 |
| 10591110 | 1.917065 | 6.401168 | -11.3947 | 7.47E-11 | 3.38E-09 | Fat3 |
| 10573008 | 1.199567 | 6.516935 | -11.3788 | 7.68E-11 | 3.47E-09 | Zfp827 |
| 10417095 | 1.210051 | 5.859217 | -11.3769 | 7.70E-11 | 3.47E-09 | Farp1 |
| 10523281 | 1.061201 | 8.735215 | -11.372 | 7.77E-11 | 3.50E-09 | 44450 |
| 10401607 | 1.207749 | 5.147999 | -11.3708 | 7.79E-11 | 3.50E-09 | Pgf |
| 10435948 | 2.491059 | 8.389554 | -11.3704 | 7.79E-11 | 3.50E-09 | Ccdc80 |
| 10389022 | 1.767183 | 8.724166 | -11.3689 | 7.81E-11 | 3.50E-09 | Myo1d |
| 10472724 | 1.116647 | 9.000231 | -11.3465 | 8.11E-11 | 3.63E-09 | Gorasp2 |
| 10357472 | -1.28764 | 9.801082 | 11.34389 | 8.15E-11 | 3.64E-09 | Cxcr4 |
| 10360972 | 1.119177 | 5.461192 | -11.3437 | 8.15E-11 | 3.64E-09 | Kcnk2 |
| 10358816 | 1.503902 | 7.750368 | -11.3427 | 8.17E-11 | 3.64E-09 | Lamc1 |
| 10361771 | 1.429908 | 6.935856 | -11.3329 | 8.31E-11 | 3.68E-09 | Plagl1 |
| 10594044 | 1.790287 | 8.279718 | -11.324 | 8.43E-11 | 3.73E-09 | Islr |
| 10470027 | 1.255236 | 6.649279 | -11.323 | 8.45E-11 | 3.73E-09 | Npdc1 |
| 10565996 | 1.157543 | 7.010334 | -11.3224 | 8.46E-11 | 3.73E-09 | Inppl1 |
| 10475544 | 1.252637 | 7.776114 | -11.3206 | 8.48E-11 | 3.74E-09 | Sema6d |
| 10581395 | 1.009514 | 6.490788 | -11.3122 | 8.61E-11 | 3.78E-09 | Slc12a4 |
| 10372069 | 1.345099 | 6.351122 | -11.303 | 8.74E-11 | 3.82E-09 | Socs2 |
| 10591123 | 3.252329 | 6.943117 | -11.301 | 8.77E-11 | 3.83E-09 | Fat3 |
| 10413304 | 1.284004 | 9.258658 | -11.2881 | 8.97E-11 | 3.91E-09 | Arf4 |
| 10375175 | 1.703903 | 7.657819 | -11.2861 | 9.00E-11 | 3.92E-09 | Slit3 |
| 10416700 | 1.280769 | 6.085312 | -11.2855 | 9.01E-11 | 3.92E-09 | Pcdh17 |
| 10440344 | 1.676569 | 5.993595 | -11.2839 | 9.03E-11 | 3.92E-09 | Robo2 |
| 10570982 | 1.60808 | 7.747606 | -11.2807 | 9.08E-11 | 3.94E-09 | Fgfr1 |
| 10591118 | 2.865154 | 7.554397 | -11.2774 | 9.13E-11 | 3.95E-09 | Fat3 |
| 10502191 | 1.405642 | 9.876721 | -11.2764 | 9.15E-11 | 3.95E-09 | Ostc |
| 10472538 | -1.69737 | 5.98095 | 11.27322 | 9.20E-11 | 3.96E-09 | Dhrs9 |
| 10606735 | 1.271228 | 5.519224 | -11.2681 | 9.28E-11 | 3.99E-09 | Armcx2 |
| 10472181 | 1.929836 | 5.42018 | -11.265 | 9.33E-11 | 4.00E-09 | Galnt5 |
| 10488382 | 1.093834 | 8.708981 | -11.2537 | 9.51E-11 | 4.07E-09 | Cd93 |
| 10482920 | 1.051335 | 7.664366 | -11.2514 | 9.55E-11 | 4.08E-09 | Cd302 |
| 10600852 | -2.56119 | 9.00811 | 11.24922 | 9.59E-11 | 4.09E-09 | F630028O10Rik |
| 10556381 | 1.208663 | 6.554738 | -11.2347 | 9.83E-11 | 4.18E-09 | Mical2 |
| 10529485 | 1.372829 | 6.361254 | -11.2335 | 9.85E-11 | 4.18E-09 | Htra3 |
| 10606369 | 2.265107 | 8.593891 | -11.2332 | 9.86E-11 | 4.18E-09 | Itm2a |
| 10534927 | -1.86044 | 8.147686 | 11.23255 | 9.87E-11 | 4.18E-09 | Pilra |
| 10604637 | 1.159203 | 6.862397 | -11.2309 | 9.90E-11 | 4.19E-09 | Cxx1b |
| 10372028 | -1.55554 | 7.015691 | 11.22739 | 9.96E-11 | 4.20E-09 | Plxnc1 |
| 10357115 | 1.726062 | 5.71186 | -11.2273 | 9.96E-11 | 4.20E-09 | Dsel |
| 10544875 | 1.723654 | 5.741988 | -11.2199 | 1.01E-10 | 4.25E-09 | Scrn1 |
| 10539517 | 1.242874 | 6.25409 | -11.217 | 1.01E-10 | 4.26E-09 | Dysf |
| 10455852 | 1.091381 | 7.474403 | -11.2144 | 1.02E-10 | 4.28E-09 | Prrc1 |
| 10439766 | 1.474167 | 6.392615 | -11.2077 | 1.03E-10 | 4.32E-09 | Pvrl3 |
| 10484318 | 1.069945 | 8.698281 | -11.2073 | 1.03E-10 | 4.32E-09 | Nckap1 |
| 10559796 | 2.464086 | 6.912734 | -11.1966 | 1.05E-10 | 4.38E-09 | Peg3 |
| 10506643 | 1.357881 | 9.624768 | -11.1928 | 1.06E-10 | 4.41E-09 | Tmem59 |
| 10443408 | -1.27741 | 6.460476 | 11.18782 | 1.07E-10 | 4.44E-09 | Mapk13 |
| 10360764 | 1.969352 | 7.770739 | -11.1853 | 1.07E-10 | 4.45E-09 | Enah |
| 10363173 | 2.206539 | 9.981664 | -11.1769 | 1.09E-10 | 4.50E-09 | Gja1 |
| 10571865 | -2.46967 | 6.130445 | 11.17225 | 1.09E-10 | 4.53E-09 | Scrg1 |
| 10400072 | 1.611759 | 4.878179 | -11.1715 | 1.10E-10 | 4.53E-09 | Scin |
| 10451646 | -1.8416 | 6.486678 | 11.17032 | 1.10E-10 | 4.53E-09 | A530064D06Rik |
| 10381096 | 1.269256 | 9.563953 | -11.1682 | 1.10E-10 | 4.54E-09 | Igfbp4 |
| 10606948 | 1.293811 | 5.45755 | -11.1622 | 1.11E-10 | 4.57E-09 | Morc4 |
| 10568363 | 1.432327 | 7.241484 | -11.1607 | 1.12E-10 | 4.58E-09 | Armcx3 |
| 10599200 | 1.105075 | 7.2211 | -11.1569 | 1.12E-10 | 4.60E-09 | Pgrmc1 |
| 10496872 | 1.275158 | 7.803915 | -11.1553 | 1.13E-10 | 4.61E-09 | Eltd1 |
| 10451547 | -1.40108 | 7.597526 | 11.15028 | 1.14E-10 | 4.64E-09 | Gm16494 |
| 10498284 | 1.466053 | 7.565733 | -11.1468 | 1.14E-10 | 4.66E-09 | Wwtr1 |
| 10496727 | 1.767915 | 5.551846 | -11.1465 | 1.14E-10 | 4.66E-09 | Ddah1 |
| 10384652 | -1.24312 | 5.50149 | 11.14482 | 1.15E-10 | 4.67E-09 | Gm12057 |
| 10579744 | 1.231671 | 5.975708 | -11.1366 | 1.16E-10 | 4.71E-09 | Large |
| 10590489 | 1.214553 | 4.8732 | -11.1328 | 1.17E-10 | 4.74E-09 | Zfp105 |
| 10543967 | 1.040269 | 4.968141 | -11.1316 | 1.17E-10 | 4.74E-09 | Dgki |
| 10379262 | 1.114626 | 7.847125 | -11.1281 | 1.18E-10 | 4.76E-09 | Nf1 |
| 10557862 | -1.56736 | 8.58294 | 11.12703 | 1.18E-10 | 4.76E-09 | Itgam |
| 10588049 | 1.013069 | 8.983749 | -11.1221 | 1.19E-10 | 4.79E-09 | Copb2 |
| 10587023 | -1.8685 | 7.140141 | 11.11176 | 1.22E-10 | 4.87E-09 | Rab27a |
| 10406663 | 1.967438 | 9.134308 | -11.1012 | 1.24E-10 | 4.96E-09 | Arsb |
| 10353549 | 1.750331 | 6.20765 | -11.0911 | 1.26E-10 | 5.04E-09 | Fam135a |
| 10481262 | -2.18011 | 7.683691 | 11.07777 | 1.29E-10 | 5.15E-09 | Fcnb |
| 10591116 | 2.532077 | 7.383229 | -11.0758 | 1.29E-10 | 5.15E-09 | Fat3 |
| 10459288 | -1.83755 | 7.704022 | 11.07478 | 1.30E-10 | 5.15E-09 | Adrb2 |
| 10422013 | 1.433964 | 5.932179 | -11.0708 | 1.31E-10 | 5.17E-09 | Klf12 |
| 10469786 | -2.26492 | 6.979675 | 11.06223 | 1.32E-10 | 5.24E-09 | Il1f9 |
| 10440491 | 1.202205 | 9.278165 | -11.0614 | 1.33E-10 | 5.25E-09 | App |
| 10466886 | 1.103219 | 6.211846 | -11.0419 | 1.37E-10 | 5.42E-09 | Glis3 |
| 10579525 | 1.547962 | 7.39503 | -11.0393 | 1.38E-10 | 5.43E-09 | Plvap |
| 10424140 | 1.693276 | 6.141889 | -11.0392 | 1.38E-10 | 5.43E-09 | Col14a1 |
| 10451851 | 1.340581 | 6.715265 | -11.0342 | 1.39E-10 | 5.47E-09 | Armcx3 |
| 10364251 | 1.365575 | 6.997094 | -11.0327 | 1.39E-10 | 5.48E-09 | Pofut2 |
| 10350337 | 1.463445 | 5.529877 | -11.0132 | 1.44E-10 | 5.65E-09 | A130050O07Rik |
| 10492815 | -1.76468 | 6.638638 | 11.01232 | 1.45E-10 | 5.65E-09 | Tmem154 |
| 10413657 | 1.27446 | 6.794852 | -11.0116 | 1.45E-10 | 5.65E-09 | Glt8d1 |
| 10607403 | 1.349808 | 5.81006 | -11.0061 | 1.46E-10 | 5.70E-09 | Kctd12b |
| 10604633 | 1.209569 | 6.823942 | -11.0052 | 1.46E-10 | 5.70E-09 | Cxx1b |
| 10418096 | 1.247588 | 6.434391 | -11.003 | 1.47E-10 | 5.71E-09 | Dlg5 |
| 10440019 | 2.218387 | 6.399905 | -10.9964 | 1.49E-10 | 5.77E-09 | Tmem45a |
| 10554863 | 2.037809 | 6.276358 | -10.9962 | 1.49E-10 | 5.77E-09 | Sytl2 |
| 10429234 | 1.376924 | 6.849293 | -10.9951 | 1.49E-10 | 5.77E-09 | Col22a1 |
| 10523693 | 2.800036 | 9.002904 | -10.9932 | 1.49E-10 | 5.78E-09 | Dmp1 |
| 10492540 | -1.09671 | 5.33575 | 10.99067 | 1.50E-10 | 5.80E-09 | Il12a |
| 10430358 | 2.097375 | 6.688571 | -10.9866 | 1.51E-10 | 5.83E-09 | C1qtnf6 |
| 10500685 | 1.223761 | 9.483812 | -10.983 | 1.52E-10 | 5.86E-09 | Atp1a1 |
| 10426315 | -1.12412 | 7.20843 | 10.97932 | 1.53E-10 | 5.89E-09 | Lrrk2 |
| 10489246 | 1.193056 | 7.19453 | -10.9765 | 1.54E-10 | 5.90E-09 | Mafb |
| 10492341 | 1.015183 | 4.824824 | -10.9732 | 1.55E-10 | 5.92E-09 | Arhgef26 |
| 10382106 | -1.80685 | 6.273005 | 10.97233 | 1.55E-10 | 5.92E-09 | Milr1 |
| 10403108 | -1.19247 | 10.72227 | 10.9711 | 1.55E-10 | 5.93E-09 | Hmgn2 |
| 10548385 | -2.13513 | 5.675527 | 10.96531 | 1.57E-10 | 5.98E-09 | Olr1 |
| 10589640 | 1.010196 | 5.494438 | -10.9642 | 1.57E-10 | 5.98E-09 | Prss46 |
| 10607499 | 2.609936 | 8.29074 | -10.9639 | 1.57E-10 | 5.98E-09 | Phex |
| 10433274 | 1.330122 | 8.039048 | -10.9495 | 1.61E-10 | 6.12E-09 | Vasn |
| 10410695 | 1.369103 | 7.280886 | -10.9473 | 1.62E-10 | 6.12E-09 | Rhobtb3 |
| 10594110 | 2.059935 | 8.291355 | -10.9468 | 1.62E-10 | 6.12E-09 | Neo1 |
| 10574276 | -1.52484 | 6.80559 | 10.93828 | 1.65E-10 | 6.19E-09 | Gpr97 |
| 10392464 | 1.532905 | 6.198708 | -10.9378 | 1.65E-10 | 6.19E-09 | Fam20a |
| 10382449 | -1.00092 | 4.987589 | 10.9329 | 1.66E-10 | 6.24E-09 | Rab37 |
| 10379215 | 1.220154 | 8.596272 | -10.9285 | 1.67E-10 | 6.28E-09 | Ift20 |
| 10521759 | 1.971331 | 8.112153 | -10.9255 | 1.68E-10 | 6.31E-09 | Slit2 |
| 10437080 | 1.220596 | 7.851891 | -10.9214 | 1.69E-10 | 6.34E-09 | Ttc3 |
| 10403727 | 1.54676 | 6.02966 | -10.9198 | 1.70E-10 | 6.35E-09 | Gli3 |
| 10542993 | 1.141148 | 7.678933 | -10.9158 | 1.71E-10 | 6.39E-09 | Pon3 |
| 10498576 | 1.593859 | 6.883624 | -10.9127 | 1.72E-10 | 6.42E-09 | Lxn |
| 10429341 | 1.324486 | 6.587426 | -10.9092 | 1.73E-10 | 6.45E-09 | Ptk2 |
| 10385635 | 1.74051 | 7.123643 | -10.9036 | 1.75E-10 | 6.50E-09 | Zfp354c |
| 10495651 | 1.400699 | 6.879357 | -10.9027 | 1.75E-10 | 6.50E-09 | Alg14 |
| 10585545 | 1.267539 | 6.358797 | -10.902 | 1.75E-10 | 6.50E-09 | Rcn2 |
| 10367734 | 1.674508 | 6.401669 | -10.9018 | 1.75E-10 | 6.50E-09 | Ust |
| 10456522 | 1.072966 | 8.222647 | -10.9012 | 1.76E-10 | 6.50E-09 | Tcf4 |
| 10513362 | -1.15548 | 6.582609 | 10.89541 | 1.77E-10 | 6.55E-09 | Susd1 |
| 10473097 | 1.114177 | 7.986286 | -10.8846 | 1.81E-10 | 6.66E-09 | Plekha3 |
| 10545458 | 1.089399 | 6.224631 | -10.877 | 1.83E-10 | 6.74E-09 | Tcf7l1 |
| 10459335 | 1.453628 | 5.131142 | -10.8366 | 1.97E-10 | 7.21E-09 | Piezo2 |
| 10436666 | 1.919953 | 7.507807 | -10.8145 | 2.05E-10 | 7.48E-09 | Jam2 |
| 10451679 | 1.313467 | 6.493622 | -10.8082 | 2.07E-10 | 7.55E-09 | Daam2 |
| 10563715 | -2.21134 | 7.501322 | 10.80532 | 2.08E-10 | 7.57E-09 | Mrgpra2a |
| 10402142 | -1.27558 | 7.03237 | 10.79169 | 2.13E-10 | 7.72E-09 | Ccdc88c |
| 10371959 | 1.204686 | 7.874921 | -10.7912 | 2.13E-10 | 7.72E-09 | Elk3 |
| 10577641 | 2.017273 | 6.329777 | -10.791 | 2.13E-10 | 7.72E-09 | 1810011O10Rik |
| 10472350 | -2.63777 | 6.761171 | 10.78756 | 2.15E-10 | 7.76E-09 | Gca |
| 10517373 | 1.12749 | 6.508685 | -10.7726 | 2.20E-10 | 7.95E-09 | Rcan3 |
| 10415045 | 1.169854 | 9.0141 | -10.7668 | 2.23E-10 | 8.02E-09 | Mrpl52 |
| 10476538 | 1.547668 | 6.719986 | -10.7641 | 2.24E-10 | 8.05E-09 | Btbd3 |
| 10575497 | 1.629018 | 7.357658 | -10.7518 | 2.29E-10 | 8.20E-09 | Mtss1l |
| 10474361 | 1.301253 | 5.685406 | -10.7253 | 2.40E-10 | 8.56E-09 | Mpped2 |
| 10489878 | 1.673707 | 7.802646 | -10.7241 | 2.40E-10 | 8.57E-09 | Ptgis |
| 10595211 | 2.806878 | 9.097704 | -10.7168 | 2.43E-10 | 8.67E-09 | Col12a1 |
| 10457250 | 1.035439 | 7.090281 | -10.7116 | 2.46E-10 | 8.74E-09 | Arhgap12 |
| 10411611 | -1.59238 | 5.517839 | 10.70956 | 2.46E-10 | 8.75E-09 | Naip5 |
| 10471929 | -1.82721 | 8.333129 | 10.70917 | 2.47E-10 | 8.75E-09 | Arhgap15 |
| 10596637 | -1.17048 | 7.363331 | 10.70596 | 2.48E-10 | 8.79E-09 | Mapkapk3 |
| 10425053 | -2.03866 | 7.086308 | 10.69463 | 2.53E-10 | 8.96E-09 | Ncf4 |
| 10516637 | 1.053771 | 6.419475 | -10.6935 | 2.54E-10 | 8.97E-09 | Fam167b |
| 10578322 | 2.011488 | 7.641734 | -10.6914 | 2.55E-10 | 8.99E-09 | Gm9868 |
| 10458303 | 1.298189 | 7.50484 | -10.6869 | 2.57E-10 | 9.05E-09 | Ecscr |
| 10591092 | 2.041493 | 5.718169 | -10.6853 | 2.57E-10 | 9.07E-09 | Fat3 |
| 10423599 | 1.704908 | 6.478416 | -10.6823 | 2.59E-10 | 9.10E-09 | Matn2 |
| 10536505 | 1.032897 | 5.950794 | -10.6704 | 2.64E-10 | 9.29E-09 | Met |
| 10478447 | -1.22078 | 8.085597 | 10.66098 | 2.69E-10 | 9.42E-09 | Stk4 |
| 10394627 | 1.138476 | 6.840781 | -10.6609 | 2.69E-10 | 9.42E-09 | Nbas |
| 10376332 | -1.33093 | 5.333822 | 10.66013 | 2.69E-10 | 9.42E-09 | 4930438A08Rik |
| 10363887 | 1.091489 | 4.589761 | -10.6574 | 2.71E-10 | 9.44E-09 | LOC101056131 |
| 10429754 | 1.212842 | 5.865125 | -10.657 | 2.71E-10 | 9.44E-09 | Nrbp2 |
| 10505073 | 1.093522 | 6.654921 | -10.6511 | 2.74E-10 | 9.51E-09 | Zfp462 |
| 10365749 | -1.40093 | 7.94453 | 10.64421 | 2.77E-10 | 9.60E-09 | Lta4h |
| 10497689 | 1.045986 | 6.170184 | -10.6384 | 2.80E-10 | 9.69E-09 | Gnb4 |
| 10534102 | 1.107784 | 7.106625 | -10.6374 | 2.80E-10 | 9.70E-09 | Gusb |
| 10381371 | 1.47269 | 5.67038 | -10.6349 | 2.82E-10 | 9.73E-09 | Aoc3 |
| 10410124 | 1.192776 | 8.72236 | -10.6265 | 2.86E-10 | 9.85E-09 | Ctsl |
| 10534168 | 1.199287 | 5.745506 | -10.6263 | 2.86E-10 | 9.85E-09 | Auts2 |
| 10471457 | 1.348284 | 6.567706 | -10.6226 | 2.88E-10 | 9.89E-09 | St6galnac4 |
| 10526566 | 1.150656 | 5.846926 | -10.6169 | 2.91E-10 | 9.97E-09 | Ephb4 |
| 10549647 | -1.28811 | 5.100565 | 10.60783 | 2.96E-10 | 1.01E-08 | Ncr1 |
| 10451604 | 1.118861 | 6.249722 | -10.599 | 3.00E-10 | 1.02E-08 | Mdfi |
| 10418868 | -1.26416 | 5.786409 | 10.59884 | 3.00E-10 | 1.02E-08 | Wdfy4 |
| 10531931 | 1.939104 | 9.073399 | -10.5988 | 3.00E-10 | 1.02E-08 | Sparcl1 |
| 10539263 | 1.206312 | 6.49036 | -10.5975 | 3.01E-10 | 1.02E-08 | Loxl3 |
| 10585048 | 1.601071 | 7.983102 | -10.5883 | 3.06E-10 | 1.04E-08 | Cadm1 |
| 10379044 | 1.370743 | 6.264515 | -10.5858 | 3.07E-10 | 1.04E-08 | Rab34 |
| 10444665 | 1.12347 | 7.478608 | -10.5844 | 3.08E-10 | 1.04E-08 | Ddah2 |
| 10524684 | 1.55899 | 5.331833 | -10.5799 | 3.11E-10 | 1.05E-08 | Msi1 |
| 10569569 | 1.149666 | 7.294015 | -10.5784 | 3.12E-10 | 1.05E-08 | Cttn |
| 10433776 | 2.08523 | 8.439388 | -10.5763 | 3.13E-10 | 1.05E-08 | Snai2 |
| 10436372 | 1.191637 | 6.01141 | -10.5741 | 3.14E-10 | 1.06E-08 | Dcbld2 |
| 10520187 | 1.091801 | 7.009743 | -10.5683 | 3.17E-10 | 1.07E-08 | Slc4a2 |
| 10564818 | 1.82327 | 7.404519 | -10.5661 | 3.19E-10 | 1.07E-08 | Anpep |
| 10477012 | 1.0629 | 8.63918 | -10.5582 | 3.23E-10 | 1.08E-08 | Fkbp1a |
| 10499168 | 1.483468 | 6.66145 | -10.5499 | 3.28E-10 | 1.10E-08 | Kirrel |
| 10571312 | 1.225175 | 5.800838 | -10.5476 | 3.29E-10 | 1.10E-08 | Dusp4 |
| 10460666 | 1.00342 | 7.039856 | -10.5428 | 3.32E-10 | 1.11E-08 | Ltbp3 |
| 10601412 | 1.469907 | 5.77313 | -10.537 | 3.36E-10 | 1.12E-08 | Lpar4 |
| 10438639 | -1.6359 | 6.107743 | 10.52337 | 3.44E-10 | 1.14E-08 | Dgkg |
| 10603796 | 1.104685 | 4.682745 | -10.5125 | 3.51E-10 | 1.16E-08 | 4930578C19Rik |
| 10472501 | -1.46723 | 7.577905 | 10.51 | 3.52E-10 | 1.16E-08 | Cers6 |
| 10497817 | 1.107396 | 10.0112 | -10.5098 | 3.53E-10 | 1.16E-08 | Anxa5 |
| 10600597 | 1.521332 | 6.922319 | -10.5022 | 3.57E-10 | 1.17E-08 | Tmem47 |
| 10498998 | -1.57454 | 8.033554 | 10.49602 | 3.61E-10 | 1.19E-08 | D930015E06Rik |
| 10489701 | 2.53392 | 6.726626 | -10.495 | 3.62E-10 | 1.19E-08 | Ocstamp |
| 10593878 | 1.009576 | 5.620685 | -10.4891 | 3.66E-10 | 1.20E-08 | Snx33 |
| 10551815 | 1.664043 | 7.941056 | -10.4869 | 3.67E-10 | 1.20E-08 | Zfp260 |
| 10563712 | -2.31804 | 7.724774 | 10.47274 | 3.77E-10 | 1.23E-08 | Mrgpra2a |
| 10594092 | 1.404798 | 7.00294 | -10.4637 | 3.83E-10 | 1.25E-08 | Cd276 |
| 10572282 | 1.082741 | 5.437 | -10.4601 | 3.86E-10 | 1.25E-08 | Hapln4 |
| 10421697 | 1.441012 | 6.602042 | -10.4569 | 3.88E-10 | 1.26E-08 | Lacc1 |
| 10604175 | 1.378965 | 5.236121 | -10.4515 | 3.92E-10 | 1.27E-08 | Tmem255a |
| 10585874 | 1.397285 | 9.491599 | -10.4509 | 3.92E-10 | 1.27E-08 | Hexa |
| 10585588 | 1.285484 | 6.743135 | -10.4496 | 3.93E-10 | 1.27E-08 | Cspg4 |
| 10566350 | -1.88084 | 6.132001 | 10.44938 | 3.93E-10 | 1.27E-08 | Trim30b |
| 10605437 | 2.395544 | 7.567845 | -10.4479 | 3.94E-10 | 1.27E-08 | Pls3 |
| 10395466 | 1.121481 | 6.02784 | -10.4468 | 3.95E-10 | 1.27E-08 | Dock4 |
| 10436598 | 1.411674 | 4.352741 | -10.4409 | 3.99E-10 | 1.28E-08 | 2810055G20Rik |
| 10498018 | 1.939422 | 7.355766 | -10.435 | 4.04E-10 | 1.29E-08 | Pcdh18 |
| 10435497 | -2.39541 | 8.778434 | 10.43079 | 4.07E-10 | 1.29E-08 | Stfa2l1 |
| 10512489 | -1.08353 | 6.126102 | 10.43046 | 4.07E-10 | 1.29E-08 | Arhgef39 |
| 10599174 | 1.033364 | 6.631221 | -10.4297 | 4.07E-10 | 1.29E-08 | Il13ra1 |
| 10553559 | -1.61493 | 5.91266 | 10.42727 | 4.09E-10 | 1.30E-08 | Siglech |
| 10456071 | 1.318644 | 8.360349 | -10.4222 | 4.13E-10 | 1.31E-08 | Csf1r |
| 10359762 | -1.19456 | 7.578734 | 10.42056 | 4.14E-10 | 1.31E-08 | Rcsd1 |
| 10416533 | 1.357148 | 6.183269 | -10.4183 | 4.16E-10 | 1.31E-08 | Ccdc122 |
| 10460468 | 1.061264 | 6.876626 | -10.415 | 4.18E-10 | 1.32E-08 | Ctsf |
| 10488673 | 1.200999 | 6.133947 | -10.4143 | 4.19E-10 | 1.32E-08 | Foxs1 |
| 10488060 | 1.152542 | 5.50168 | -10.4071 | 4.24E-10 | 1.33E-08 | Jag1 |
| 10374366 | 1.305737 | 6.795416 | -10.4049 | 4.26E-10 | 1.34E-08 | Egfr |
| 10399924 | -1.32231 | 7.369074 | 10.40424 | 4.27E-10 | 1.34E-08 | Pik3cg |
| 10552760 | -1.21374 | 6.620527 | 10.39998 | 4.30E-10 | 1.34E-08 | Pnkp |
| 10461423 | 1.193407 | 7.055695 | -10.3959 | 4.33E-10 | 1.35E-08 | Fads3 |
| 10446253 | -1.36815 | 6.579515 | 10.39468 | 4.34E-10 | 1.35E-08 | Vav1 |
| 10354677 | -1.08067 | 7.018507 | 10.39267 | 4.36E-10 | 1.36E-08 | Ankrd44 |
| 10509901 | 1.886465 | 7.404834 | -10.3917 | 4.36E-10 | 1.36E-08 | Mfap2 |
| 10549760 | 1.40646 | 7.159643 | -10.3778 | 4.48E-10 | 1.39E-08 | Zfp580 |
| 10362596 | 1.197535 | 7.455501 | -10.3769 | 4.48E-10 | 1.39E-08 | Fyn |
| 10419156 | -2.43948 | 8.41834 | 10.37318 | 4.51E-10 | 1.39E-08 | Ear2 |
| 10441497 | 1.498781 | 7.42449 | -10.3684 | 4.55E-10 | 1.40E-08 | Tulp4 |
| 10350473 | 1.085812 | 7.35397 | -10.367 | 4.57E-10 | 1.40E-08 | B3galt2 |
| 10607225 | 1.183942 | 4.507017 | -10.3667 | 4.57E-10 | 1.40E-08 | Lrch2 |
| 10522060 | 1.692877 | 7.987857 | -10.3596 | 4.63E-10 | 1.42E-08 | Fam114a1 |
| 10456184 | 1.172773 | 6.222565 | -10.355 | 4.67E-10 | 1.43E-08 | Apcdd1 |
| 10587299 | 1.097408 | 6.595541 | -10.3528 | 4.68E-10 | 1.43E-08 | Ick |
| 10430425 | 1.096272 | 4.763891 | -10.3461 | 4.74E-10 | 1.45E-08 | Lgals2 |
| 10435043 | 2.000856 | 6.948568 | -10.3454 | 4.75E-10 | 1.45E-08 | Tm4sf19 |
| 10418053 | 1.722411 | 7.146514 | -10.3417 | 4.78E-10 | 1.46E-08 | Kcnma1 |
| 10480956 | -1.00621 | 6.246332 | 10.33459 | 4.84E-10 | 1.47E-08 | Card9 |
| 10548817 | -1.95076 | 9.102389 | 10.33453 | 4.84E-10 | 1.47E-08 | Plbd1 |
| 10434229 | 1.041211 | 6.689442 | -10.3299 | 4.88E-10 | 1.48E-08 | Cldn5 |
| 10501007 | -1.55327 | 4.706774 | 10.32494 | 4.93E-10 | 1.50E-08 | Chil5 |
| 10504054 | -1.17156 | 5.131285 | 10.31291 | 5.04E-10 | 1.53E-08 | n-R5s183 |
| 10595793 | 1.015684 | 9.05738 | -10.3108 | 5.06E-10 | 1.53E-08 | Atp1b3 |
| 10598175 | -2.35058 | 8.122477 | 10.29881 | 5.17E-10 | 1.56E-08 | Ear10 |
| 10419892 | 1.266799 | 6.040174 | -10.2983 | 5.17E-10 | 1.56E-08 | Efs |
| 10412298 | 1.481113 | 7.27628 | -10.2982 | 5.18E-10 | 1.56E-08 | Itga1 |
| 10542522 | 1.066154 | 6.258873 | -10.2948 | 5.21E-10 | 1.56E-08 | Plekha5 |
| 10455128 | 1.076706 | 4.149501 | -10.2766 | 5.38E-10 | 1.61E-08 | Pcdhb20 |
| 10464084 | 1.012251 | 7.930093 | -10.2756 | 5.39E-10 | 1.61E-08 | Tcf7l2 |
| 10544273 | -2.31126 | 6.56615 | 10.27409 | 5.41E-10 | 1.61E-08 | Clec5a |
| 10437817 | 1.009522 | 7.732718 | -10.2703 | 5.45E-10 | 1.62E-08 | Pdxdc1 |
| 10363070 | -1.70446 | 8.659573 | 10.27022 | 5.45E-10 | 1.62E-08 | Gp49a |
| 10587829 | 2.688345 | 7.511789 | -10.2608 | 5.54E-10 | 1.64E-08 | Plod2 |
| 10555323 | 1.01062 | 5.23575 | -10.2592 | 5.56E-10 | 1.65E-08 | P4ha3 |
| 10598081 | -2.67765 | 8.004636 | 10.25364 | 5.62E-10 | 1.66E-08 | mt-Ty |
| 10347291 | -1.95255 | 9.091726 | 10.25255 | 5.63E-10 | 1.66E-08 | Cxcr2 |
| 10443598 | -1.18929 | 4.986585 | 10.25054 | 5.65E-10 | 1.66E-08 | Dnah8 |
| 10345824 | -2.00458 | 6.593583 | 10.24916 | 5.66E-10 | 1.66E-08 | Il18rap |
| 10436596 | 1.687136 | 5.845773 | -10.2491 | 5.66E-10 | 1.66E-08 | 2810055G20Rik |
| 10380419 | 2.541725 | 11.10957 | -10.2449 | 5.71E-10 | 1.68E-08 | Col1a1 |
| 10539080 | -1.50191 | 8.768699 | 10.24331 | 5.72E-10 | 1.68E-08 | St3gal5 |
| 10347980 | 1.272139 | 8.156114 | -10.2325 | 5.84E-10 | 1.71E-08 | Itm2c |
| 10588263 | 1.724587 | 6.747665 | -10.2267 | 5.90E-10 | 1.72E-08 | Slco2a1 |
| 10458583 | 1.352745 | 8.36493 | -10.2262 | 5.91E-10 | 1.72E-08 | Yipf5 |
| 10429856 | -2.20174 | 7.446041 | 10.22613 | 5.91E-10 | 1.72E-08 | Gm10872 |
| 10419850 | -1.72288 | 7.166051 | 10.22492 | 5.92E-10 | 1.73E-08 | Cebpe |
| 10347781 | 1.034112 | 4.771538 | -10.2223 | 5.95E-10 | 1.73E-08 | Nyap2 |
| 10498273 | 1.336327 | 6.778633 | -10.2174 | 6.00E-10 | 1.75E-08 | Tm4sf1 |
| 10559454 | -1.54522 | 9.015006 | 10.20882 | 6.10E-10 | 1.77E-08 | Gm10693 |
| 10490212 | 1.238102 | 9.012691 | -10.2038 | 6.15E-10 | 1.78E-08 | Ctsz |
| 10511368 | 1.109317 | 7.154028 | -10.2021 | 6.17E-10 | 1.78E-08 | Impad1 |
| 10539739 | -1.29069 | 6.533688 | 10.19517 | 6.25E-10 | 1.81E-08 | Asprv1 |
| 10587446 | 1.352201 | 6.457947 | -10.1895 | 6.32E-10 | 1.82E-08 | Myo6 |
| 10502776 | 1.499922 | 7.01111 | -10.1832 | 6.39E-10 | 1.84E-08 | Lphn2 |
| 10353899 | 1.023199 | 5.807459 | -10.1743 | 6.50E-10 | 1.87E-08 | Sema4c |
| 10569504 | 1.476451 | 6.091915 | -10.1679 | 6.57E-10 | 1.89E-08 | Tnfrsf23 |
| 10384370 | -1.00805 | 6.771264 | 10.16434 | 6.62E-10 | 1.90E-08 | Gm12000 |
| 10451641 | -1.79742 | 5.33986 | 10.16096 | 6.66E-10 | 1.91E-08 | 9830107B12Rik |
| 10548892 | -1.6652 | 10.18392 | 10.15966 | 6.67E-10 | 1.91E-08 | Arhgdib |
| 10505172 | 1.028029 | 6.272596 | -10.1516 | 6.77E-10 | 1.94E-08 | Dnajc25 |
| 10571252 | -1.28203 | 3.780455 | 10.15108 | 6.78E-10 | 1.94E-08 | Tex15 |
| 10402268 | 1.655876 | 8.962466 | -10.1504 | 6.79E-10 | 1.94E-08 | Lgmn |
| 10483081 | 2.166712 | 8.972122 | -10.1502 | 6.79E-10 | 1.94E-08 | Fap |
| 10412562 | 1.092146 | 6.837707 | -10.1499 | 6.80E-10 | 1.94E-08 | Flnb |
| 10418410 | -1.11971 | 8.448857 | 10.14579 | 6.85E-10 | 1.95E-08 | Prkcd |
| 10471882 | 1.711855 | 6.105832 | -10.1445 | 6.86E-10 | 1.95E-08 | Olfml2a |
| 10485711 | 1.874041 | 5.672938 | -10.1431 | 6.88E-10 | 1.96E-08 | Fibin |
| 10591135 | 2.599327 | 8.057721 | -10.1376 | 6.95E-10 | 1.97E-08 | Fat3 |
| 10346000 | 1.411738 | 5.955913 | -10.1178 | 7.21E-10 | 2.04E-08 | Gulp1 |
| 10591125 | 2.118895 | 6.765464 | -10.1159 | 7.24E-10 | 2.05E-08 | Fat3 |
| 10559478 | -1.75444 | 6.746058 | 10.10846 | 7.34E-10 | 2.07E-08 | Lilra6 |
| 10534935 | -1.73601 | 7.76665 | 10.10732 | 7.35E-10 | 2.07E-08 | Pilrb1 |
| 10505489 | 1.703522 | 7.111124 | -10.1061 | 7.37E-10 | 2.08E-08 | Pappa |
| 10569719 | -1.21714 | 6.357317 | 10.10504 | 7.38E-10 | 2.08E-08 | A430078G23Rik |
| 10541599 | -1.51748 | 5.40447 | 10.09778 | 7.48E-10 | 2.10E-08 | Clec4b2 |
| 10536917 | 1.155592 | 6.646267 | -10.0657 | 7.94E-10 | 2.22E-08 | Smo |
| 10603551 | -1.99893 | 9.421879 | 10.06052 | 8.02E-10 | 2.24E-08 | Cybb |
| 10567049 | 1.116389 | 8.989088 | -10.0496 | 8.18E-10 | 2.28E-08 | Copb1 |
| 10370587 | 1.204295 | 5.196904 | -10.0298 | 8.49E-10 | 2.36E-08 | Shc2 |
| 10574985 | 1.179317 | 6.895165 | -10.0252 | 8.56E-10 | 2.37E-08 | Slc7a6 |
| 10564527 | 1.039842 | 6.493572 | -10.0252 | 8.56E-10 | 2.37E-08 | Nr2f2 |
| 10402783 | 1.147238 | 6.385579 | -10.0138 | 8.75E-10 | 2.42E-08 | Ahnak2 |
| 10542140 | -1.88583 | 5.54131 | 10.01072 | 8.80E-10 | 2.43E-08 | Klrb1f |
| 10381298 | 1.25235 | 6.311474 | -10.0106 | 8.80E-10 | 2.43E-08 | Ramp2 |
| 10514590 | 1.126222 | 7.244836 | -10.0075 | 8.85E-10 | 2.44E-08 | Dock7 |
| 10351867 | -1.25979 | 6.771026 | 10.00248 | 8.93E-10 | 2.46E-08 | Aim2 |
| 10513256 | 1.33215 | 7.512187 | -10.0007 | 8.96E-10 | 2.46E-08 | Lpar1 |
| 10573939 | -1.0336 | 7.282731 | 9.999694 | 8.98E-10 | 2.46E-08 | Lpcat2 |
| 10504375 | 1.277088 | 6.414862 | -9.99864 | 9.00E-10 | 2.47E-08 | Npr2 |
| 10464754 | 1.143906 | 5.911098 | -9.99629 | 9.04E-10 | 2.47E-08 | Rhod |
| 10455112 | 1.412441 | 5.232173 | -9.9901 | 9.14E-10 | 2.50E-08 | Pcdhb17 |
| 10491732 | 1.406796 | 6.474829 | -9.9887 | 9.16E-10 | 2.50E-08 | Fat4 |
| 10382341 | 1.494893 | 4.943049 | -9.9818 | 9.28E-10 | 2.53E-08 | Sstr2 |
| 10569877 | -2.39698 | 8.702335 | 9.976019 | 9.38E-10 | 2.55E-08 | Mcemp1 |
| 10566454 | 1.270756 | 7.691713 | -9.97592 | 9.39E-10 | 2.55E-08 | Prkcdbp |
| 10368675 | 1.229262 | 10.48538 | -9.97469 | 9.41E-10 | 2.55E-08 | Marcks |
| 10482824 | 1.25837 | 6.347587 | -9.96884 | 9.51E-10 | 2.58E-08 | Acvr1 |
| 10405216 | -1.44291 | 7.520279 | 9.968351 | 9.52E-10 | 2.58E-08 | Syk |
| 10468311 | 1.131987 | 5.805756 | -9.96547 | 9.57E-10 | 2.59E-08 | Sh3pxd2a |
| 10436456 | 1.134626 | 7.878293 | -9.96296 | 9.61E-10 | 2.60E-08 | Pros1 |
| 10439296 | -2.14902 | 6.308809 | 9.962185 | 9.63E-10 | 2.60E-08 | Stfa2 |
| 10591094 | 1.777834 | 6.20973 | -9.95776 | 9.71E-10 | 2.62E-08 | Fat3 |
| 10605143 | -1.6662 | 6.987878 | 9.95299 | 9.80E-10 | 2.64E-08 | Arhgap4 |
| 10543306 | 1.328655 | 6.84923 | -9.9526 | 9.80E-10 | 2.64E-08 | Tspan12 |
| 10555892 | 1.028114 | 7.624825 | -9.94778 | 9.89E-10 | 2.66E-08 | Twf1 |
| 10429580 | -2.74674 | 8.685972 | 9.942038 | 1.00E-09 | 2.69E-08 | I830127L07Rik |
| 10421950 | -1.54176 | 6.005043 | 9.940159 | 1.00E-09 | 2.69E-08 | Dach1 |
| 10460108 | 1.13719 | 6.960788 | -9.9391 | 1.01E-09 | 2.69E-08 | Gnpnat1 |
| 10490989 | 1.296462 | 8.448674 | -9.93503 | 1.01E-09 | 2.71E-08 | Cp |
| 10523727 | 1.495029 | 7.58363 | -9.93083 | 1.02E-09 | 2.73E-08 | Pkd2 |
| 10391762 | 1.174881 | 5.614799 | -9.93035 | 1.02E-09 | 2.73E-08 | Gjc1 |
| 10538150 | 1.378001 | 8.158937 | -9.92969 | 1.02E-09 | 2.73E-08 | Tmem176a |
| 10504775 | 1.147737 | 6.362712 | -9.92429 | 1.03E-09 | 2.75E-08 | Col15a1 |
| 10594418 | 1.058179 | 6.103765 | -9.91996 | 1.04E-09 | 2.77E-08 | Smad6 |
| 10568873 | -1.33367 | 6.471544 | 9.912606 | 1.06E-09 | 2.80E-08 | Adam8 |
| 10455752 | 1.01456 | 6.269265 | -9.91227 | 1.06E-09 | 2.80E-08 | Snx24 |
| 10446656 | -1.07179 | 7.835046 | 9.91071 | 1.06E-09 | 2.81E-08 | Lpin2 |
| 10401296 | 1.517295 | 6.446182 | -9.89029 | 1.10E-09 | 2.91E-08 | Slc8a3 |
| 10488655 | -1.40957 | 8.204465 | 9.886047 | 1.11E-09 | 2.93E-08 | Bcl2l1 |
| 10429114 | -1.81477 | 6.085244 | 9.884322 | 1.11E-09 | 2.93E-08 | Tmem71 |
| 10542181 | -1.97003 | 5.645092 | 9.883177 | 1.12E-09 | 2.93E-08 | Clec9a |
| 10406504 | 2.270517 | 6.235492 | -9.8831 | 1.12E-09 | 2.93E-08 | Edil3 |
| 10498357 | -1.24218 | 5.291963 | 9.882539 | 1.12E-09 | 2.93E-08 | F630111L10Rik |
| 10357875 | -1.42152 | 8.238624 | 9.850078 | 1.19E-09 | 3.10E-08 | Btg2 |
| 10403871 | -1.42431 | 6.665648 | 9.846026 | 1.20E-09 | 3.12E-08 | Aoah |
| 10536220 | 2.386625 | 11.10501 | -9.84178 | 1.21E-09 | 3.14E-08 | Col1a2 |
| 10448278 | -2.21921 | 6.121496 | 9.837871 | 1.22E-09 | 3.16E-08 | Mmp25 |
| 10361215 | -1.69494 | 5.918194 | 9.834722 | 1.22E-09 | 3.17E-08 | Traf3ip3 |
| 10601846 | 1.063272 | 5.794595 | -9.82377 | 1.25E-09 | 3.24E-08 | Arxes2 |
| 10393559 | 1.302629 | 9.345062 | -9.82074 | 1.26E-09 | 3.25E-08 | Timp2 |
| 10542965 | 1.052799 | 6.364247 | -9.81254 | 1.28E-09 | 3.30E-08 | Sgce |
| 10354506 | -1.02585 | 6.136472 | 9.81014 | 1.28E-09 | 3.31E-08 | Mfsd6 |
| 10532839 | 1.512297 | 5.915946 | -9.80737 | 1.29E-09 | 3.32E-08 | Trpv4 |
| 10356084 | 1.532662 | 7.682104 | -9.782 | 1.35E-09 | 3.46E-08 | Irs1 |
| 10362495 | 2.359481 | 7.38997 | -9.77933 | 1.36E-09 | 3.47E-08 | Col10a1 |
| 10503376 | 1.377604 | 5.593273 | -9.77932 | 1.36E-09 | 3.47E-08 | Triqk |
| 10587554 | 1.49006 | 6.45489 | -9.77769 | 1.36E-09 | 3.48E-08 | Tpbg |
| 10500982 | -2.07031 | 7.349622 | 9.776279 | 1.37E-09 | 3.48E-08 | I830077J02Rik |
| 10578493 | 1.064415 | 5.517861 | -9.76985 | 1.38E-09 | 3.52E-08 | Tlr3 |
| 10587241 | 1.572779 | 5.865585 | -9.76226 | 1.40E-09 | 3.57E-08 | Hmgcll1 |
| 10509122 | -1.34405 | 6.173093 | 9.756156 | 1.42E-09 | 3.60E-08 | Cnr2 |
| 10452269 | -1.53721 | 6.499024 | 9.751435 | 1.43E-09 | 3.63E-08 | Dennd1c |
| 10565727 | 1.162667 | 6.020831 | -9.7495 | 1.44E-09 | 3.64E-08 | Tsku |
| 10383615 | 1.142564 | 6.342024 | -9.74841 | 1.44E-09 | 3.65E-08 | Metrnl |
| 10362102 | -1.32052 | 7.22952 | 9.739076 | 1.47E-09 | 3.71E-08 | Gm10825 |
| 10483163 | 1.669045 | 6.797592 | -9.72507 | 1.50E-09 | 3.80E-08 | Grb14 |
| 10425116 | 1.488159 | 5.507083 | -9.72051 | 1.52E-09 | 3.83E-08 | Cdc42ep1 |
| 10572605 | -1.10415 | 5.796694 | 9.708346 | 1.55E-09 | 3.92E-08 | Ankle1 |
| 10521757 | -1.59423 | 6.600996 | 9.703019 | 1.57E-09 | 3.95E-08 | Gm7931 |
| 10454353 | -1.20015 | 5.760574 | 9.700842 | 1.58E-09 | 3.96E-08 | Mocos |
| 10500804 | 1.246594 | 5.263153 | -9.6936 | 1.60E-09 | 4.01E-08 | LOC269472 |
| 10439612 | 1.282677 | 6.192756 | -9.68069 | 1.64E-09 | 4.10E-08 | Boc |
| 10429128 | -1.85851 | 7.846361 | 9.676234 | 1.65E-09 | 4.13E-08 | Sla |
| 10587880 | 1.632996 | 6.804264 | -9.6675 | 1.68E-09 | 4.18E-08 | Pcolce2 |
| 10526853 | 1.144079 | 6.555866 | -9.66156 | 1.70E-09 | 4.23E-08 | Fam20c |
| 10412260 | 1.014669 | 6.628345 | -9.65796 | 1.71E-09 | 4.25E-08 | Fst |
| 10556082 | 1.037731 | 7.333099 | -9.65499 | 1.72E-09 | 4.27E-08 | Ppfibp2 |
| 10462343 | -1.25713 | 6.221375 | 9.648763 | 1.74E-09 | 4.32E-08 | Gm9895 |
| 10383532 | -1.14719 | 7.167304 | 9.644094 | 1.75E-09 | 4.35E-08 | Narf |
| 10484307 | 1.985885 | 5.16224 | -9.64269 | 1.76E-09 | 4.36E-08 | Frzb |
| 10467006 | 1.689916 | 6.845454 | -9.64257 | 1.76E-09 | 4.36E-08 | Dkk1 |
| 10511180 | 1.717327 | 9.346479 | -9.6375 | 1.78E-09 | 4.39E-08 | Mxra8 |
| 10367746 | 1.320928 | 7.813274 | -9.63361 | 1.79E-09 | 4.42E-08 | Sash1 |
| 10595466 | 1.367622 | 6.347079 | -9.62848 | 1.81E-09 | 4.46E-08 | Pgm3 |
| 10545921 | -1.31944 | 7.993005 | 9.626549 | 1.81E-09 | 4.47E-08 | Mxd1 |
| 10469581 | 1.263297 | 6.568556 | -9.62012 | 1.84E-09 | 4.52E-08 | Etl4 |
| 10507840 | 1.447935 | 6.04217 | -9.61579 | 1.85E-09 | 4.54E-08 | Heyl |
| 10597470 | 1.341345 | 5.96461 | -9.61568 | 1.85E-09 | 4.54E-08 | Cmtm8 |
| 10530319 | -1.80461 | 8.143376 | 9.614854 | 1.86E-09 | 4.55E-08 | Atp8a1 |
| 10587616 | 2.754024 | 6.456914 | -9.61397 | 1.86E-09 | 4.55E-08 | Prss35 |
| 10348817 | 1.058554 | 8.937067 | -9.61306 | 1.86E-09 | 4.55E-08 | 44441 |
| 10414262 | -2.48242 | 7.543066 | 9.610616 | 1.87E-09 | 4.56E-08 | Ear2 |
| 10378549 | 1.36107 | 7.033708 | -9.60916 | 1.88E-09 | 4.57E-08 | Rtn4rl1 |
| 10399148 | 1.289579 | 4.877365 | -9.60738 | 1.88E-09 | 4.58E-08 | Rapgef5 |
| 10434105 | 1.205206 | 6.442703 | -9.59668 | 1.92E-09 | 4.64E-08 | Scarf2 |
| 10410460 | 1.328221 | 5.645303 | -9.58922 | 1.95E-09 | 4.70E-08 | Ube2ql1 |
| 10503709 | -1.37388 | 5.000639 | 9.571103 | 2.02E-09 | 4.87E-08 | D130062J21Rik |
| 10432439 | 1.189264 | 6.855274 | -9.56826 | 2.03E-09 | 4.88E-08 | Fmnl3 |
| 10434782 | 1.237511 | 7.003592 | -9.56783 | 2.03E-09 | 4.88E-08 | Lpp |
| 10545780 | 1.021643 | 6.87767 | -9.56502 | 2.04E-09 | 4.91E-08 | Exoc6b |
| 10492078 | 1.18144 | 7.721275 | -9.56307 | 2.05E-09 | 4.92E-08 | Alg5 |
| 10595209 | 1.20213 | 4.168224 | -9.56261 | 2.05E-09 | 4.92E-08 | Gm10635 |
| 10389581 | 1.053944 | 6.828284 | -9.55759 | 2.07E-09 | 4.95E-08 | Ypel2 |
| 10409660 | 1.040008 | 7.527995 | -9.55754 | 2.07E-09 | 4.95E-08 | Gkap1 |
| 10547641 | -1.96083 | 7.551939 | 9.557022 | 2.07E-09 | 4.95E-08 | Slc2a3 |
| 10542650 | 1.078294 | 8.892284 | -9.54198 | 2.13E-09 | 5.09E-08 | Golt1b |
| 10445767 | -2.20327 | 6.994618 | 9.541427 | 2.14E-09 | 5.09E-08 | Treml2 |
| 10511617 | 1.540717 | 6.914729 | -9.53986 | 2.14E-09 | 5.10E-08 | Fam92a |
| 10460544 | 1.07922 | 7.511784 | -9.53354 | 2.17E-09 | 5.16E-08 | Yif1a |
| 10460253 | -1.25228 | 4.988793 | 9.529702 | 2.18E-09 | 5.19E-08 | Aldh3b2 |
| 10487208 | -2.28601 | 7.586919 | 9.529317 | 2.19E-09 | 5.19E-08 | Atp8b4 |
| 10419216 | 1.03848 | 7.133381 | -9.5281 | 2.19E-09 | 5.20E-08 | Gnpnat1 |
| 10520388 | -1.31514 | 7.494653 | 9.525701 | 2.20E-09 | 5.22E-08 | Rbm33 |
| 10381934 | 1.025628 | 6.77951 | -9.51853 | 2.23E-09 | 5.28E-08 | Tanc2 |
| 10538811 | 1.543822 | 6.192825 | -9.51471 | 2.25E-09 | 5.32E-08 | Prdm5 |
| 10375677 | -1.237 | 3.554963 | 9.508526 | 2.28E-09 | 5.37E-08 | Mir340 |
| 10550509 | -2.96538 | 9.320425 | 9.5083 | 2.28E-09 | 5.37E-08 | Pglyrp1 |
| 10442098 | -1.22311 | 5.793186 | 9.504183 | 2.29E-09 | 5.41E-08 | Fpr2 |
| 10372917 | 1.226099 | 6.562356 | -9.50379 | 2.30E-09 | 5.41E-08 | Tmem5 |
| 10530156 | -1.20416 | 5.066296 | 9.500435 | 2.31E-09 | 5.44E-08 | Tmem156 |
| 10540207 | 1.49292 | 5.127193 | -9.49694 | 2.33E-09 | 5.47E-08 | A730049H05Rik |
| 10493449 | 1.078939 | 5.660269 | -9.49464 | 2.34E-09 | 5.49E-08 | Thbs3 |
| 10434934 | 1.078139 | 5.854181 | -9.48721 | 2.37E-09 | 5.55E-08 | Bdh1 |
| 10392221 | 1.056046 | 8.165377 | -9.48681 | 2.37E-09 | 5.55E-08 | Pecam1 |
| 10373223 | 1.211289 | 8.224749 | -9.48644 | 2.37E-09 | 5.55E-08 | Lrp1 |
| 10598093 | -1.41402 | 7.142264 | 9.476052 | 2.42E-09 | 5.65E-08 | Tarm1 |
| 10411728 | -1.44707 | 6.591701 | 9.472164 | 2.44E-09 | 5.68E-08 | Cenph |
| 10429926 | -1.04422 | 7.557484 | 9.471997 | 2.44E-09 | 5.68E-08 | Dgat1 |
| 10594855 | 1.308804 | 6.611435 | -9.47107 | 2.45E-09 | 5.68E-08 | Cgnl1 |
| 10581890 | 1.033247 | 6.345305 | -9.46339 | 2.48E-09 | 5.76E-08 | Bcar1 |
| 10558961 | 1.201267 | 7.116251 | -9.4626 | 2.49E-09 | 5.76E-08 | Tspan4 |
| 10348653 | 1.4838 | 7.759592 | -9.45959 | 2.50E-09 | 5.79E-08 | Gpc1 |
| 10490894 | 1.047857 | 5.320065 | -9.44881 | 2.55E-09 | 5.90E-08 | E2f5 |
| 10495659 | 1.204394 | 7.058761 | -9.44837 | 2.55E-09 | 5.90E-08 | Cnn3 |
| 10487645 | 1.473017 | 6.356988 | -9.44817 | 2.56E-09 | 5.90E-08 | Cpxm1 |
| 10536818 | 1.472447 | 9.367907 | -9.4458 | 2.57E-09 | 5.92E-08 | Calu |
| 10475378 | 1.298226 | 6.589058 | -9.44381 | 2.58E-09 | 5.93E-08 | Casc4 |
| 10583326 | 1.097163 | 5.849229 | -9.43961 | 2.60E-09 | 5.97E-08 | Slc36a4 |
| 10407946 | 1.020037 | 7.275188 | -9.43779 | 2.61E-09 | 5.98E-08 | Stard3nl |
| 10490731 | 1.615321 | 7.114262 | -9.43334 | 2.63E-09 | 6.03E-08 | Sox18 |
| 10547100 | 1.48255 | 7.604715 | -9.42288 | 2.68E-09 | 6.14E-08 | Plxnd1 |
| 10367673 | 1.001737 | 6.369083 | -9.41739 | 2.71E-09 | 6.20E-08 | Plekhg1 |
| 10481147 | 1.268493 | 9.328703 | -9.4036 | 2.79E-09 | 6.36E-08 | Surf4 |
| 10497149 | 1.130704 | 7.520498 | -9.40025 | 2.80E-09 | 6.39E-08 | Wls |
| 10557571 | -1.31247 | 6.290401 | 9.394914 | 2.83E-09 | 6.45E-08 | AI467606 |
| 10458046 | 2.28626 | 9.176174 | -9.37291 | 2.96E-09 | 6.71E-08 | Nrep |
| 10370544 | 1.268115 | 7.056412 | -9.37072 | 2.97E-09 | 6.73E-08 | 2610008E11Rik |
| 10559467 | -1.67957 | 8.202396 | 9.366081 | 3.00E-09 | 6.79E-08 | Gm15448 |
| 10591131 | 2.187066 | 5.891434 | -9.36125 | 3.02E-09 | 6.83E-08 | Fat3 |
| 10580577 | 1.03741 | 6.548538 | -9.36103 | 3.03E-09 | 6.83E-08 | Irx3 |
| 10371321 | 1.168351 | 5.905964 | -9.34569 | 3.12E-09 | 7.00E-08 | Slc41a2 |
| 10554926 | 1.369425 | 7.561602 | -9.34562 | 3.12E-09 | 7.00E-08 | Ccdc90b |
| 10604380 | 1.035079 | 7.80133 | -9.34174 | 3.14E-09 | 7.05E-08 | Zdhhc9 |
| 10523802 | -1.22988 | 5.655323 | 9.339122 | 3.16E-09 | 7.08E-08 | Cdc7 |
| 10389617 | 1.158072 | 5.982589 | -9.3323 | 3.20E-09 | 7.17E-08 | Ppm1e |
| 10357191 | 1.237763 | 7.347534 | -9.32659 | 3.24E-09 | 7.24E-08 | Ptpn4 |
| 10364650 | -1.74869 | 7.632242 | 9.321297 | 3.27E-09 | 7.30E-08 | Hmha1 |
| 10583669 | -1.50792 | 8.549965 | 9.321285 | 3.27E-09 | 7.30E-08 | AB124611 |
| 10546452 | 1.415827 | 6.75167 | -9.31781 | 3.29E-09 | 7.35E-08 | Adamts9 |
| 10513957 | 1.611661 | 8.60237 | -9.31305 | 3.32E-09 | 7.40E-08 | Ptprd |
| 10542470 | -1.23558 | 9.25358 | 9.307494 | 3.36E-09 | 7.48E-08 | Mgst1 |
| 10384154 | -1.48633 | 7.126227 | 9.295639 | 3.44E-09 | 7.63E-08 | Myo1g |
| 10459353 | 1.181247 | 5.258675 | -9.28191 | 3.53E-09 | 7.83E-08 | Piezo2 |
| 10440393 | -1.33511 | 7.235456 | 9.278759 | 3.55E-09 | 7.87E-08 | Samsn1 |
| 10553773 | 1.050622 | 4.841362 | -9.27596 | 3.57E-09 | 7.90E-08 | Gabrb3 |
| 10569341 | 1.520007 | 6.949078 | -9.2746 | 3.58E-09 | 7.91E-08 | H19 |
| 10607952 | 1.196202 | 7.845914 | -9.27384 | 3.59E-09 | 7.91E-08 | Vamp7 |
| 10583021 | 1.775974 | 7.032207 | -9.27244 | 3.60E-09 | 7.92E-08 | Pdgfd |
| 10408557 | -2.12281 | 8.678163 | 9.271949 | 3.60E-09 | 7.92E-08 | Serpinb1a |
| 10378216 | -2.04291 | 7.169169 | 9.268435 | 3.62E-09 | 7.97E-08 | Atp2a3 |
| 10445746 | -1.65236 | 5.607296 | 9.264587 | 3.65E-09 | 8.02E-08 | Trem1 |
| 10347910 | 1.044251 | 6.884848 | -9.26029 | 3.68E-09 | 8.07E-08 | Fbxo36 |
| 10566438 | -1.11003 | 6.666933 | 9.257679 | 3.70E-09 | 8.11E-08 | Fam160a2 |
| 10473312 | 1.726878 | 5.42144 | -9.25407 | 3.73E-09 | 8.15E-08 | Fam171b |
| 10514510 | 1.143127 | 5.268712 | -9.24429 | 3.80E-09 | 8.27E-08 | Cyp2j6 |
| 10466659 | -1.54231 | 9.18392 | 9.240894 | 3.82E-09 | 8.31E-08 | Gda |
| 10493114 | 1.432017 | 4.928012 | -9.23399 | 3.88E-09 | 8.42E-08 | Nes |
| 10370339 | -1.51234 | 5.137844 | 9.233424 | 3.88E-09 | 8.42E-08 | Trpm2 |
| 10430679 | -1.69192 | 4.746665 | 9.232296 | 3.89E-09 | 8.43E-08 | Gm24204 |
| 10359635 | 1.095124 | 5.928461 | -9.23191 | 3.89E-09 | 8.43E-08 | Gorab |
| 10452815 | -1.06621 | 7.589912 | 9.230249 | 3.90E-09 | 8.45E-08 | Xdh |
| 10600765 | -1.15808 | 5.497184 | 9.212068 | 4.05E-09 | 8.72E-08 | Pcyt1b |
| 10406982 | 1.037598 | 5.075897 | -9.21148 | 4.05E-09 | 8.72E-08 | Adamts6 |
| 10368577 | 1.360127 | 6.803432 | -9.20757 | 4.08E-09 | 8.77E-08 | Rnf217 |
| 10440376 | 1.647513 | 4.242967 | -9.20378 | 4.11E-09 | 8.83E-08 | Lipi |
| 10600857 | 1.062203 | 5.124811 | -9.1938 | 4.19E-09 | 8.97E-08 | Heph |
| 10594812 | 1.030537 | 5.955962 | -9.1916 | 4.21E-09 | 8.99E-08 | Lipc |
| 10351603 | -1.33306 | 7.53392 | 9.191503 | 4.21E-09 | 8.99E-08 | Arhgap30 |
| 10554574 | -1.04832 | 8.632663 | 9.187519 | 4.25E-09 | 9.04E-08 | Tm6sf1 |
| 10470775 | 1.304367 | 7.277853 | -9.18616 | 4.26E-09 | 9.06E-08 | Cercam |
| 10356293 | -1.13735 | 6.65254 | 9.176526 | 4.34E-09 | 9.21E-08 | A630001G21Rik |
| 10508074 | -1.8322 | 7.922936 | 9.176384 | 4.34E-09 | 9.21E-08 | Csf3r |
| 10422164 | 1.213767 | 5.6296 | -9.17623 | 4.34E-09 | 9.21E-08 | Ednrb |
| 10566543 | 1.172912 | 5.747629 | -9.16617 | 4.43E-09 | 9.36E-08 | Dchs1 |
| 10464647 | -1.20735 | 6.069262 | 9.164326 | 4.44E-09 | 9.39E-08 | Tbc1d10c |
| 10379736 | -2.13474 | 9.97551 | 9.16191 | 4.47E-09 | 9.43E-08 | Wfdc21 |
| 10555297 | 1.362178 | 5.73406 | -9.15961 | 4.49E-09 | 9.46E-08 | Kcne3 |
| 10475517 | -2.13447 | 7.320069 | 9.159002 | 4.49E-09 | 9.47E-08 | AA467197 |
| 10492102 | 1.084135 | 6.321944 | -9.14807 | 4.59E-09 | 9.65E-08 | Spg20 |
| 10400896 | 1.06746 | 5.220434 | -9.14518 | 4.62E-09 | 9.70E-08 | L3hypdh |
| 10581061 | 1.100539 | 6.373117 | -9.13692 | 4.69E-09 | 9.83E-08 | Cmtm4 |
| 10346015 | 2.199131 | 8.906954 | -9.13601 | 4.70E-09 | 9.84E-08 | Col3a1 |
| 10536499 | 1.562434 | 7.821041 | -9.12903 | 4.76E-09 | 9.95E-08 | Cav1 |
| 10495685 | 1.654331 | 7.063511 | -9.12161 | 4.83E-09 | 1.01E-07 | Arhgap29 |
| 10519140 | 1.78852 | 7.109213 | -9.11959 | 4.85E-09 | 1.01E-07 | Mmp23 |
| 10490159 | 1.657455 | 8.223992 | -9.11233 | 4.92E-09 | 1.02E-07 | Pmepa1 |
| 10430929 | -1.05524 | 6.984577 | 9.109855 | 4.95E-09 | 1.03E-07 | Tbrg3 |
| 10526559 | -2.07727 | 6.517239 | 9.107036 | 4.98E-09 | 1.03E-07 | Ache |
| 10455139 | 1.104581 | 5.075859 | -9.09241 | 5.12E-09 | 1.06E-07 | Pcdhb22 |
| 10568024 | -1.81607 | 8.728913 | 9.086119 | 5.19E-09 | 1.07E-07 | Coro1a |
| 10349383 | 1.032966 | 7.103316 | -9.08491 | 5.20E-09 | 1.07E-07 | Slc35f5 |
| 10593842 | 1.324818 | 8.111822 | -9.08487 | 5.20E-09 | 1.07E-07 | Tspan3 |
| 10469941 | -1.45444 | 6.062681 | 9.084546 | 5.20E-09 | 1.07E-07 | Gm22572 |
| 10465895 | 1.151927 | 8.324112 | -9.08447 | 5.20E-09 | 1.07E-07 | Fads2 |
| 10554249 | 2.379124 | 6.183841 | -9.08346 | 5.21E-09 | 1.07E-07 | Acan |
| 10393379 | 1.101732 | 6.391851 | -9.07415 | 5.31E-09 | 1.09E-07 | Mxra7 |
| 10475487 | -1.15699 | 6.099469 | 9.071975 | 5.33E-09 | 1.09E-07 | Slc28a2 |
| 10575976 | -1.03268 | 6.309289 | 9.067365 | 5.38E-09 | 1.10E-07 | Crispld2 |
| 10376929 | 1.050237 | 6.803665 | -9.06294 | 5.43E-09 | 1.11E-07 | Tvp23b |
| 10569848 | -1.33505 | 7.916203 | 9.041706 | 5.66E-09 | 1.15E-07 | Stxbp2 |
| 10491952 | -1.98464 | 8.034322 | 9.03264 | 5.76E-09 | 1.17E-07 | Mgst2 |
| 10439483 | 1.160462 | 7.723815 | -9.02523 | 5.85E-09 | 1.18E-07 | Arhgap31 |
| 10498160 | 1.172756 | 7.591468 | -9.02362 | 5.87E-09 | 1.19E-07 | Ufm1 |
| 10469066 | 1.591986 | 7.519415 | -9.02253 | 5.88E-09 | 1.19E-07 | Ccdc3 |
| 10448124 | -2.61009 | 7.407924 | 9.017209 | 5.94E-09 | 1.20E-07 | Fpr1 |
| 10571274 | -1.22219 | 9.472148 | 9.010057 | 6.03E-09 | 1.21E-07 | Gsr |
| 10559446 | -1.56695 | 8.453034 | 9.009756 | 6.03E-09 | 1.21E-07 | Pirb |
| 10415413 | 1.531465 | 6.07784 | -9.00782 | 6.06E-09 | 1.22E-07 | Nynrin |
| 10571142 | 1.164975 | 5.901395 | -9.0047 | 6.09E-09 | 1.22E-07 | Gpr124 |
| 10419154 | -2.59288 | 8.397669 | 8.998924 | 6.16E-09 | 1.23E-07 | Ear1 |
| 10462140 | -1.40095 | 8.395965 | 8.992747 | 6.24E-09 | 1.25E-07 | Dock8 |
| 10449266 | 1.097333 | 6.411724 | -8.98342 | 6.36E-09 | 1.27E-07 | Itfg3 |
| 10532620 | 1.453241 | 10.50457 | -8.98087 | 6.39E-09 | 1.27E-07 | Myo18b |
| 10404152 | -1.87736 | 7.522944 | 8.980561 | 6.39E-09 | 1.27E-07 | Fam65b |
| 10347792 | 2.052152 | 6.841619 | -8.97611 | 6.45E-09 | 1.28E-07 | Gm9747 |
| 10524310 | 1.206129 | 7.983643 | -8.96465 | 6.60E-09 | 1.31E-07 | Ttc28 |
| 10440993 | 1.382194 | 7.753307 | -8.96125 | 6.64E-09 | 1.32E-07 | Rcan1 |
| 10597960 | -1.3611 | 6.481606 | 8.953329 | 6.75E-09 | 1.33E-07 | Slc6a20a |
| 10453057 | 1.282035 | 8.267022 | -8.9509 | 6.78E-09 | 1.34E-07 | Cyp1b1 |
| 10564849 | 1.040661 | 6.05083 | -8.94974 | 6.80E-09 | 1.34E-07 | 2610034B18Rik |
| 10550906 | -1.50353 | 7.267182 | 8.938456 | 6.95E-09 | 1.37E-07 | Plaur |
| 10502780 | 1.140821 | 6.76613 | -8.93842 | 6.95E-09 | 1.37E-07 | Lphn2 |
| 10366951 | 1.765416 | 6.253925 | -8.93655 | 6.98E-09 | 1.37E-07 | Ndufa4l2 |
| 10350297 | -1.31156 | 7.258784 | 8.93427 | 7.01E-09 | 1.38E-07 | Kif14 |
| 10572130 | 1.347342 | 9.20765 | -8.93203 | 7.04E-09 | 1.38E-07 | Lpl |
| 10574471 | 1.260196 | 8.49203 | -8.93096 | 7.06E-09 | 1.38E-07 | Cmtm3 |
| 10547410 | 1.081 | 6.844468 | -8.92996 | 7.07E-09 | 1.38E-07 | Erc1 |
| 10364109 | -2.49204 | 6.740648 | 8.929193 | 7.08E-09 | 1.38E-07 | Vpreb3 |
| 10500656 | -1.15049 | 4.941111 | 8.916902 | 7.26E-09 | 1.42E-07 | Cd101 |
| 10358717 | 1.115264 | 8.617927 | -8.91014 | 7.36E-09 | 1.43E-07 | 1700025G04Rik |
| 10495186 | -2.45082 | 6.631939 | 8.907091 | 7.40E-09 | 1.44E-07 | AI504432 |
| 10482772 | 1.002534 | 6.50667 | -8.90408 | 7.45E-09 | 1.44E-07 | Nr4a2 |
| 10423556 | 1.271012 | 7.78792 | -8.89288 | 7.62E-09 | 1.47E-07 | Cpq |
| 10542981 | -1.40988 | 9.797357 | 8.891312 | 7.64E-09 | 1.48E-07 | Gmfg |
| 10350377 | 1.028961 | 6.674312 | -8.89024 | 7.66E-09 | 1.48E-07 | Zbtb41 |
| 10526952 | 1.002016 | 5.974953 | -8.86519 | 8.05E-09 | 1.55E-07 | Gper1 |
| 10571788 | 1.222368 | 6.88948 | -8.86234 | 8.10E-09 | 1.55E-07 | Vegfc |
| 10544002 | 1.115108 | 6.399279 | -8.85509 | 8.22E-09 | 1.57E-07 | Creb3l2 |
| 10499285 | 1.362078 | 4.905778 | -8.85199 | 8.27E-09 | 1.58E-07 | Bcan |
| 10478594 | 1.089028 | 9.703024 | -8.85035 | 8.29E-09 | 1.58E-07 | Ctsa |
| 10547056 | -1.88628 | 6.547806 | 8.847354 | 8.34E-09 | 1.59E-07 | Tmem40 |
| 10397633 | 1.402208 | 7.254725 | -8.8449 | 8.39E-09 | 1.60E-07 | Flrt2 |
| 10362499 | 1.167145 | 5.671062 | -8.83733 | 8.51E-09 | 1.62E-07 | Frk |
| 10593668 | -1.0148 | 6.473151 | 8.835966 | 8.54E-09 | 1.62E-07 | Dmxl2 |
| 10466712 | 1.804248 | 5.849496 | -8.83444 | 8.56E-09 | 1.62E-07 | Mamdc2 |
| 10360120 | 1.020642 | 8.581015 | -8.8308 | 8.63E-09 | 1.64E-07 | Ufc1 |
| 10607395 | 1.239596 | 5.918997 | -8.82912 | 8.66E-09 | 1.64E-07 | Mageh1 |
| 10471535 | 1.138412 | 6.20911 | -8.82113 | 8.80E-09 | 1.67E-07 | Fam129b |
| 10588007 | -1.30973 | 8.821734 | 8.816071 | 8.89E-09 | 1.68E-07 | Tfdp2 |
| 10522788 | -1.88468 | 6.387492 | 8.805024 | 9.09E-09 | 1.71E-07 | Stap1 |
| 10467110 | 1.193153 | 6.02729 | -8.80226 | 9.14E-09 | 1.72E-07 | Lipo1 |
| 10582658 | 1.265559 | 7.407582 | -8.79893 | 9.20E-09 | 1.73E-07 | Agt |
| 10603492 | 1.260221 | 6.557785 | -8.79741 | 9.23E-09 | 1.73E-07 | Porcn |
| 10591114 | 1.740195 | 6.084073 | -8.78775 | 9.41E-09 | 1.76E-07 | Fat3 |
| 10536667 | 2.238406 | 6.549382 | -8.78229 | 9.51E-09 | 1.78E-07 | Ptprz1 |
| 10416777 | -1.10781 | 6.771432 | 8.776305 | 9.63E-09 | 1.80E-07 | Klf5 |
| 10385826 | -1.84505 | 5.523041 | 8.772539 | 9.70E-09 | 1.81E-07 | Sowaha |
| 10433480 | 1.538712 | 6.014194 | -8.74886 | 1.02E-08 | 1.89E-07 | Rpl39l |
| 10418092 | 1.274962 | 5.158039 | -8.7304 | 1.06E-08 | 1.96E-07 | A830039N20Rik |
| 10519693 | 1.853403 | 5.812605 | -8.72639 | 1.06E-08 | 1.97E-07 | Sema3d |
| 10362097 | -1.36339 | 6.678188 | 8.725292 | 1.07E-08 | 1.98E-07 | H60b |
| 10497001 | 1.015823 | 6.140505 | -8.72416 | 1.07E-08 | 1.98E-07 | Cryz |
| 10576911 | 1.07654 | 6.082103 | -8.71771 | 1.08E-08 | 2.00E-07 | Efnb2 |
| 10466521 | -1.05258 | 7.730346 | 8.712435 | 1.10E-08 | 2.02E-07 | Gcnt1 |
| 10409833 | 1.104474 | 6.942735 | -8.71022 | 1.10E-08 | 2.03E-07 | Gas1 |
| 10551852 | 1.289844 | 6.623006 | -8.70091 | 1.12E-08 | 2.07E-07 | Clip3 |
| 10554752 | 1.526429 | 5.689426 | -8.69722 | 1.13E-08 | 2.08E-07 | Nox4 |
| 10432640 | -1.84508 | 7.534918 | 8.693766 | 1.14E-08 | 2.09E-07 | Bin2 |
| 10543058 | 1.51909 | 7.845389 | -8.69202 | 1.14E-08 | 2.10E-07 | Dlx5 |
| 10398240 | 1.220816 | 6.903883 | -8.68813 | 1.15E-08 | 2.11E-07 | Eml1 |
| 10537834 | 1.014288 | 6.005961 | -8.68782 | 1.15E-08 | 2.11E-07 | Arhgef5 |
| 10563820 | -1.0214 | 8.067333 | 8.685398 | 1.16E-08 | 2.12E-07 | Svip |
| 10554808 | 1.130337 | 6.095163 | -8.68481 | 1.16E-08 | 2.12E-07 | Fzd4 |
| 10605542 | -1.55141 | 4.738334 | 8.657539 | 1.22E-08 | 2.23E-07 | Mageb16 |
| 10464704 | -1.12804 | 8.20572 | 8.649677 | 1.24E-08 | 2.26E-07 | Adrbk1 |
| 10445753 | -1.95661 | 6.994372 | 8.649597 | 1.24E-08 | 2.26E-07 | Trem3 |
| 10425410 | -1.82788 | 6.696062 | 8.645419 | 1.26E-08 | 2.27E-07 | Grap2 |
| 10487238 | -1.83627 | 7.589398 | 8.645005 | 1.26E-08 | 2.27E-07 | Hdc |
| 10586244 | -1.40222 | 8.203759 | 8.643523 | 1.26E-08 | 2.28E-07 | Dennd4a |
| 10412123 | -1.24315 | 7.630925 | 8.642645 | 1.26E-08 | 2.28E-07 | Ncf2 |
| 10447951 | 2.020668 | 7.913783 | -8.63739 | 1.28E-08 | 2.30E-07 | Thbs2 |
| 10523923 | -1.12109 | 5.080973 | 8.618689 | 1.33E-08 | 2.38E-07 | Ccdc18 |
| 10362201 | 1.310922 | 8.48546 | -8.61565 | 1.33E-08 | 2.39E-07 | Ctgf |
| 10443980 | -1.80111 | 8.285038 | 8.613075 | 1.34E-08 | 2.40E-07 | Myo1f |
| 10504450 | -1.11314 | 6.827722 | 8.611653 | 1.34E-08 | 2.40E-07 | Glipr2 |
| 10361995 | -1.1853 | 5.748519 | 8.598833 | 1.38E-08 | 2.46E-07 | Mtfr2 |
| 10523511 | 1.06742 | 4.246368 | -8.59857 | 1.38E-08 | 2.46E-07 | Prkg2 |
| 10468980 | -1.26734 | 7.30695 | 8.59493 | 1.39E-08 | 2.48E-07 | Fam107b |
| 10590620 | -1.07286 | 4.760322 | 8.575256 | 1.45E-08 | 2.57E-07 | Ccr9 |
| 10458983 | -1.15521 | 6.811576 | 8.574248 | 1.45E-08 | 2.58E-07 | 44258 |
| 10483800 | 1.000687 | 5.052757 | -8.56965 | 1.46E-08 | 2.60E-07 | Hoxd3os1 |
| 10405432 | -1.05269 | 6.30977 | 8.569589 | 1.47E-08 | 2.60E-07 | Rgs14 |
| 10495147 | -1.11202 | 5.027706 | 8.560257 | 1.49E-08 | 2.64E-07 | Dennd2d |
| 10365559 | 1.002142 | 8.265862 | -8.55589 | 1.51E-08 | 2.66E-07 | Igf1 |
| 10438378 | -1.05274 | 7.474809 | 8.550787 | 1.52E-08 | 2.68E-07 | Cdc45 |
| 10567173 | 1.003016 | 7.48954 | -8.54669 | 1.54E-08 | 2.70E-07 | Pik3c2a |
| 10462822 | -1.3184 | 8.157614 | 8.542533 | 1.55E-08 | 2.72E-07 | Exoc6 |
| 10436100 | -2.03801 | 11.12161 | 8.539645 | 1.56E-08 | 2.73E-07 | Retnlg |
| 10438769 | -1.29813 | 5.557442 | 8.539213 | 1.56E-08 | 2.73E-07 | Cldn1 |
| 10485372 | -1.68828 | 5.131903 | 8.536239 | 1.57E-08 | 2.74E-07 | Rag1 |
| 10405811 | 1.018946 | 6.314917 | -8.53545 | 1.57E-08 | 2.75E-07 | Habp4 |
| 10466216 | -1.00336 | 4.361135 | 8.530323 | 1.59E-08 | 2.77E-07 | Ms4a2 |
| 10416800 | 1.329035 | 7.262217 | -8.53022 | 1.59E-08 | 2.77E-07 | Lmo7 |
| 10503508 | 1.140826 | 6.93232 | -8.52846 | 1.59E-08 | 2.77E-07 | Ggh |
| 10503523 | 1.140826 | 6.93232 | -8.52846 | 1.59E-08 | 2.77E-07 | Ggh |
| 10569020 | -2.42811 | 9.913275 | 8.523558 | 1.61E-08 | 2.80E-07 | Ifitm6 |
| 10564507 | 1.137573 | 7.385135 | -8.52051 | 1.62E-08 | 2.81E-07 | Arrdc4 |
| 10431424 | 1.19079 | 7.150311 | -8.51696 | 1.63E-08 | 2.83E-07 | Plxnb2 |
| 10489463 | -1.7177 | 8.810048 | 8.516778 | 1.63E-08 | 2.83E-07 | Slpi |
| 10544186 | -1.51874 | 8.904086 | 8.513513 | 1.64E-08 | 2.84E-07 | Mkrn1 |
| 10483249 | 1.337914 | 6.580948 | -8.51262 | 1.65E-08 | 2.85E-07 | Galnt3 |
| 10451993 | 1.072612 | 7.332709 | -8.5074 | 1.66E-08 | 2.87E-07 | D17Wsu104e |
| 10517967 | 1.816626 | 6.440119 | -8.50216 | 1.68E-08 | 2.90E-07 | Fblim1 |
| 10495945 | -1.11534 | 5.573472 | 8.502064 | 1.68E-08 | 2.90E-07 | Zgrf1 |
| 10419151 | -3.0616 | 7.929203 | 8.489713 | 1.73E-08 | 2.96E-07 | Ear1 |
| 10348299 | -1.48254 | 4.955837 | 8.487288 | 1.73E-08 | 2.97E-07 | 5830472F04Rik |
| 10551696 | -1.85061 | 7.054728 | 8.483342 | 1.75E-08 | 2.99E-07 | Rasgrp4 |
| 10541034 | -1.12839 | 6.101084 | 8.482821 | 1.75E-08 | 2.99E-07 | Zfand4 |
| 10349166 | -2.21805 | 5.840366 | 8.473152 | 1.79E-08 | 3.05E-07 | Serpinb10 |
| 10537909 | 1.398789 | 11.17841 | -8.47155 | 1.79E-08 | 3.06E-07 | Rny3 |
| 10351197 | -1.98086 | 8.346012 | 8.467613 | 1.81E-08 | 3.08E-07 | Sell |
| 10356461 | -1.04758 | 7.730012 | 8.465718 | 1.81E-08 | 3.09E-07 | Hjurp |
| 10383025 | 1.319127 | 6.418567 | -8.46517 | 1.81E-08 | 3.09E-07 | C1qtnf1 |
| 10344897 | 1.444359 | 6.680233 | -8.46471 | 1.82E-08 | 3.09E-07 | Sulf1 |
| 10607658 | 1.011384 | 5.709749 | -8.46414 | 1.82E-08 | 3.09E-07 | Reps2 |
| 10595979 | 1.250238 | 6.913753 | -8.45464 | 1.85E-08 | 3.15E-07 | Mras |
| 10595126 | -1.0321 | 7.710659 | 8.443994 | 1.90E-08 | 3.21E-07 | Fbxo9 |
| 10424683 | -2.85732 | 9.093925 | 8.443549 | 1.90E-08 | 3.21E-07 | Ly6g |
| 10463211 | 1.010862 | 6.235077 | -8.43669 | 1.92E-08 | 3.25E-07 | Pi4k2a |
| 10588942 | 1.056505 | 7.015618 | -8.43532 | 1.93E-08 | 3.26E-07 | Lamb2 |
| 10576610 | 1.08367 | 5.882271 | -8.43285 | 1.94E-08 | 3.28E-07 | Pard3 |
| 10428534 | 1.464146 | 7.78715 | -8.42711 | 1.96E-08 | 3.31E-07 | Trps1 |
| 10598198 | 1.093736 | 7.443614 | -8.42098 | 1.99E-08 | 3.35E-07 | Mia3 |
| 10430245 | -1.45639 | 6.884153 | 8.414923 | 2.01E-08 | 3.38E-07 | Gm22107 |
| 10498952 | 1.139336 | 6.933767 | -8.41407 | 2.02E-08 | 3.38E-07 | Gucy1a3 |
| 10460968 | -1.60541 | 7.791966 | 8.410017 | 2.03E-08 | 3.41E-07 | Rasgrp2 |
| 10588691 | 1.184132 | 5.59017 | -8.40852 | 2.04E-08 | 3.42E-07 | Hyal1 |
| 10417027 | 1.318704 | 5.345739 | -8.40422 | 2.06E-08 | 3.45E-07 | Cldn10 |
| 10464370 | -1.0056 | 5.026428 | 8.396955 | 2.09E-08 | 3.49E-07 | Slc18a2 |
| 10498568 | 1.304541 | 5.776615 | -8.39501 | 2.10E-08 | 3.50E-07 | Shox2 |
| 10519060 | -1.52514 | 8.284666 | 8.37763 | 2.17E-08 | 3.61E-07 | Tnfrsf14 |
| 10465580 | 1.008834 | 6.740138 | -8.37614 | 2.18E-08 | 3.62E-07 | Nudt22 |
| 10588091 | -1.09318 | 6.087881 | 8.370284 | 2.21E-08 | 3.66E-07 | Cep70 |
| 10537184 | 1.013376 | 8.187978 | -8.36644 | 2.23E-08 | 3.68E-07 | Cald1 |
| 10527638 | -1.52466 | 9.63497 | 8.362012 | 2.25E-08 | 3.71E-07 | Alox5ap |
| 10469457 | 1.383176 | 8.240467 | -8.35692 | 2.27E-08 | 3.74E-07 | Plxdc2 |
| 10374333 | -1.69098 | 7.733182 | 8.349045 | 2.31E-08 | 3.79E-07 | Ikzf1 |
| 10450025 | -1.3212 | 8.551844 | 8.335384 | 2.37E-08 | 3.89E-07 | 44257 |
| 10560242 | -1.39394 | 6.995457 | 8.327187 | 2.41E-08 | 3.95E-07 | C5ar1 |
| 10441361 | 1.040268 | 6.164754 | -8.32135 | 2.44E-08 | 3.99E-07 | Tiam2 |
| 10351224 | -1.84505 | 6.75865 | 8.305604 | 2.52E-08 | 4.10E-07 | F5 |
| 10423080 | 1.755239 | 5.756412 | -8.30441 | 2.53E-08 | 4.11E-07 | C1qtnf3 |
| 10525195 | -1.44651 | 8.728565 | 8.303583 | 2.54E-08 | 4.12E-07 | Gm15800 |
| 10574018 | 1.081448 | 7.395238 | -8.29834 | 2.56E-08 | 4.15E-07 | Mt3 |
| 10563178 | -1.52011 | 7.647158 | 8.294375 | 2.58E-08 | 4.18E-07 | Cd37 |
| 10386058 | 1.135615 | 12.07202 | -8.29183 | 2.60E-08 | 4.20E-07 | Sparc |
| 10376396 | -1.34668 | 5.860836 | 8.29174 | 2.60E-08 | 4.20E-07 | Trim58 |
| 10395692 | 1.271739 | 6.46729 | -8.28822 | 2.62E-08 | 4.22E-07 | Arhgap5 |
| 10434291 | -1.29461 | 5.731702 | 8.283349 | 2.64E-08 | 4.26E-07 | B3gnt5 |
| 10485225 | 1.12608 | 7.536135 | -8.2814 | 2.66E-08 | 4.28E-07 | Ext2 |
| 10434698 | 1.338707 | 4.98997 | -8.28018 | 2.66E-08 | 4.29E-07 | Fetub |
| 10570291 | -1.45818 | 6.62165 | 8.277987 | 2.67E-08 | 4.30E-07 | F10 |
| 10591739 | 1.79469 | 10.65976 | -8.2645 | 2.75E-08 | 4.42E-07 | Acp5 |
| 10514054 | 1.175993 | 8.600058 | -8.26293 | 2.76E-08 | 4.42E-07 | Nfib |
| 10422052 | 1.031332 | 7.260844 | -8.25947 | 2.78E-08 | 4.44E-07 | Commd6 |
| 10460891 | -1.65995 | 7.038326 | 8.244172 | 2.87E-08 | 4.57E-07 | Map4k2 |
| 10588731 | 1.206628 | 5.723106 | -8.24198 | 2.88E-08 | 4.59E-07 | Mst1r |
| 10411235 | -1.26967 | 7.370552 | 8.23661 | 2.92E-08 | 4.63E-07 | Iqgap2 |
| 10479950 | -1.30914 | 7.858786 | 8.227466 | 2.97E-08 | 4.72E-07 | Celf2 |
| 10537179 | -1.55578 | 9.322796 | 8.216832 | 3.04E-08 | 4.82E-07 | Bpgm |
| 10457587 | 1.378234 | 6.711386 | -8.21464 | 3.05E-08 | 4.84E-07 | Zfp521 |
| 10458843 | 1.178213 | 6.247548 | -8.20929 | 3.09E-08 | 4.88E-07 | Sema6a |
| 10384458 | -1.69175 | 8.972651 | 8.205481 | 3.11E-08 | 4.90E-07 | Plek |
| 10432045 | 1.453375 | 6.861857 | -8.19973 | 3.15E-08 | 4.96E-07 | Col2a1 |
| 10361234 | -1.47002 | 6.492764 | 8.188255 | 3.23E-08 | 5.06E-07 | Hsd11b1 |
| 10541910 | -1.58473 | 7.101666 | 8.183566 | 3.26E-08 | 5.10E-07 | Vwf |
| 10404036 | -1.44739 | 6.258579 | 8.180245 | 3.28E-08 | 5.13E-07 | Hist1h2bg |
| 10589703 | -2.62066 | 10.20488 | 8.179475 | 3.29E-08 | 5.13E-07 | Ltf |
| 10445688 | -1.21879 | 8.267115 | 8.177372 | 3.30E-08 | 5.15E-07 | Ccnd3 |
| 10437639 | 1.248917 | 7.437801 | -8.17583 | 3.31E-08 | 5.17E-07 | Emp2 |
| 10372965 | -1.0129 | 8.878936 | 8.173391 | 3.33E-08 | 5.19E-07 | Usp15 |
| 10445325 | 1.075147 | 6.723385 | -8.16714 | 3.37E-08 | 5.25E-07 | Rcan2 |
| 10508772 | -1.49279 | 7.138374 | 8.163665 | 3.40E-08 | 5.28E-07 | Fgr |
| 10566333 | -1.00968 | 7.85562 | 8.14684 | 3.52E-08 | 5.44E-07 | Trim5 |
| 10497237 | -1.06706 | 5.605462 | 8.146106 | 3.52E-08 | 5.44E-07 | Pag1 |
| 10368638 | 1.320268 | 5.857063 | -8.13813 | 3.58E-08 | 5.52E-07 | Fam26e |
| 10373542 | -1.10758 | 7.114443 | 8.130621 | 3.64E-08 | 5.59E-07 | Dgka |
| 10544523 | 2.320133 | 7.797572 | -8.12692 | 3.67E-08 | 5.62E-07 | Rny1 |
| 10395702 | 1.428134 | 5.778671 | -8.12308 | 3.70E-08 | 5.65E-07 | Akap6 |
| 10541496 | 1.558206 | 8.220536 | -8.12158 | 3.71E-08 | 5.67E-07 | Mfap5 |
| 10390299 | -1.0833 | 6.863922 | 8.120237 | 3.72E-08 | 5.68E-07 | Pnpo |
| 10435288 | -1.37657 | 4.843269 | 8.111403 | 3.79E-08 | 5.78E-07 | Muc13 |
| 10591129 | 1.949693 | 7.191303 | -8.1102 | 3.80E-08 | 5.79E-07 | Fat3 |
| 10504670 | -1.06607 | 5.935124 | 8.106699 | 3.83E-08 | 5.82E-07 | E230008N13Rik |
| 10586242 | -1.32922 | 9.098509 | 8.100878 | 3.88E-08 | 5.89E-07 | Dennd4a |
| 10447341 | 1.041336 | 7.133242 | -8.10085 | 3.88E-08 | 5.89E-07 | Rhoq |
| 10595371 | 1.089396 | 6.970561 | -8.09728 | 3.91E-08 | 5.92E-07 | Hmgn3 |
| 10360745 | -1.49436 | 8.510298 | 8.09718 | 3.91E-08 | 5.92E-07 | Lbr |
| 10553324 | 1.206334 | 7.242504 | -8.09502 | 3.92E-08 | 5.94E-07 | Tmem86a |
| 10346722 | 1.145426 | 7.357191 | -8.09167 | 3.95E-08 | 5.96E-07 | Nbeal1 |
| 10432675 | -1.79509 | 5.984536 | 8.08331 | 4.02E-08 | 6.05E-07 | I730030J21Rik |
| 10530592 | -1.0233 | 8.126914 | 8.079633 | 4.05E-08 | 6.09E-07 | Fryl |
| 10566926 | -1.00323 | 6.949861 | 8.061984 | 4.21E-08 | 6.29E-07 | Rnf141 |
| 10422312 | 1.191236 | 5.013088 | -8.06149 | 4.21E-08 | 6.30E-07 | Cldn10 |
| 10445977 | -1.60793 | 6.66505 | 8.054319 | 4.28E-08 | 6.39E-07 | Ebi3 |
| 10586079 | 1.385396 | 6.511055 | -8.04742 | 4.34E-08 | 6.46E-07 | Itga11 |
| 10487894 | -1.21524 | 6.726831 | 8.045945 | 4.35E-08 | 6.48E-07 | Rassf2 |
| 10575799 | -1.04141 | 7.11688 | 8.045875 | 4.35E-08 | 6.48E-07 | Plcg2 |
| 10606876 | 1.004199 | 8.834402 | -8.04376 | 4.37E-08 | 6.50E-07 | Morf4l2 |
| 10442762 | -2.20192 | 7.232552 | 8.042816 | 4.38E-08 | 6.51E-07 | Prss34 |
| 10439299 | -1.88878 | 6.274479 | 8.042571 | 4.38E-08 | 6.51E-07 | Stfa3 |
| 10360040 | -1.17397 | 8.323569 | 8.039292 | 4.41E-08 | 6.54E-07 | Fcgr3 |
| 10358339 | 1.402465 | 10.188 | -8.03133 | 4.49E-08 | 6.63E-07 | Cfh |
| 10505438 | -1.37612 | 6.388375 | 8.023311 | 4.57E-08 | 6.73E-07 | Orm1 |
| 10492824 | -1.09103 | 4.503991 | 8.020516 | 4.59E-08 | 6.76E-07 | Tmem154 |
| 10430319 | -1.14787 | 7.070909 | 8.019517 | 4.60E-08 | 6.77E-07 | Tst |
| 10482500 | 1.151592 | 8.039781 | -8.01649 | 4.63E-08 | 6.81E-07 | Rnd3 |
| 10385776 | 1.093901 | 5.907063 | -8.00833 | 4.71E-08 | 6.91E-07 | Tcf7 |
| 10380719 | 1.222664 | 5.133256 | -8.00775 | 4.72E-08 | 6.92E-07 | Sp6 |
| 10406270 | -1.06328 | 8.262519 | 7.999818 | 4.80E-08 | 7.02E-07 | Glrx |
| 10486119 | -1.44868 | 7.006912 | 7.994286 | 4.85E-08 | 7.09E-07 | Plcb2 |
| 10542172 | -2.41738 | 7.091171 | 7.98552 | 4.95E-08 | 7.21E-07 | Clec1b |
| 10438891 | -1.67578 | 5.166645 | 7.980021 | 5.00E-08 | 7.28E-07 | Gm1968 |
| 10551587 | -1.02036 | 6.018412 | 7.978801 | 5.02E-08 | 7.30E-07 | Rinl |
| 10572212 | -1.09887 | 6.953248 | 7.97758 | 5.03E-08 | 7.31E-07 | Gmip |
| 10586227 | -1.01438 | 9.164188 | 7.967474 | 5.14E-08 | 7.45E-07 | Dennd4a |
| 10350247 | -1.26724 | 6.280488 | 7.961344 | 5.21E-08 | 7.53E-07 | Kif21b |
| 10492798 | 1.950466 | 8.068862 | -7.95827 | 5.24E-08 | 7.58E-07 | Sfrp2 |
| 10554900 | -1.07471 | 5.34324 | 7.953291 | 5.30E-08 | 7.64E-07 | Dlg2 |
| 10502778 | 1.179498 | 4.790882 | -7.94792 | 5.36E-08 | 7.70E-07 | Lphn2 |
| 10364361 | -1.16147 | 5.824775 | 7.945479 | 5.39E-08 | 7.74E-07 | Icosl |
| 10401673 | 1.545042 | 7.008307 | -7.9417 | 5.43E-08 | 7.79E-07 | Tgfb3 |
| 10537849 | 1.237224 | 6.03334 | -7.93118 | 5.55E-08 | 7.92E-07 | Arhgef5 |
| 10558134 | 1.008954 | 7.165158 | -7.93092 | 5.55E-08 | 7.92E-07 | Plekha1 |
| 10368199 | -1.96231 | 7.806846 | 7.91437 | 5.75E-08 | 8.17E-07 | Myb |
| 10495935 | -1.12177 | 6.173002 | 7.905006 | 5.87E-08 | 8.32E-07 | Zgrf1 |
| 10352767 | -1.04187 | 6.40642 | 7.898745 | 5.95E-08 | 8.42E-07 | Nek2 |
| 10586246 | -1.24327 | 9.092686 | 7.895136 | 6.00E-08 | 8.48E-07 | Dennd4a |
| 10498383 | 1.196054 | 7.070634 | -7.87679 | 6.23E-08 | 8.78E-07 | Igsf10 |
| 10542120 | -1.48206 | 6.147827 | 7.873116 | 6.28E-08 | 8.84E-07 | Clec2i |
| 10395293 | -1.55283 | 7.613351 | 7.866962 | 6.37E-08 | 8.95E-07 | Atxn7l1 |
| 10378240 | -1.53176 | 5.757033 | 7.862086 | 6.43E-08 | 9.03E-07 | P2rx1 |
| 10526564 | -1.64054 | 7.179327 | 7.856387 | 6.51E-08 | 9.14E-07 | Ufsp1 |
| 10494388 | -1.04297 | 7.091721 | 7.851689 | 6.58E-08 | 9.22E-07 | Hist2h2be |
| 10496204 | -1.17939 | 7.519541 | 7.849523 | 6.61E-08 | 9.25E-07 | Cenpe |
| 10380514 | -1.35135 | 8.09028 | 7.847994 | 6.63E-08 | 9.27E-07 | Fam117a |
| 10562761 | 1.399221 | 7.072681 | -7.84758 | 6.64E-08 | 9.27E-07 | Clec11a |
| 10389816 | -1.18181 | 6.959895 | 7.84485 | 6.68E-08 | 9.32E-07 | Tom1l1 |
| 10490775 | 1.242338 | 5.170321 | -7.83759 | 6.78E-08 | 9.44E-07 | Gm10748 |
| 10522655 | -1.37891 | 4.417266 | 7.834695 | 6.82E-08 | 9.48E-07 | C530008M17Rik |
| 10579609 | -1.47135 | 5.389645 | 7.811957 | 7.16E-08 | 9.90E-07 | Fcho1 |
| 10581961 | 1.06714 | 5.040372 | -7.80563 | 7.26E-08 | 1.00E-06 | Adamts18 |
| 10366667 | 1.246797 | 8.467185 | -7.80506 | 7.27E-08 | 1.00E-06 | Gns |
| 10363445 | -1.4374 | 7.852794 | 7.79443 | 7.44E-08 | 1.02E-06 | 4632428N05Rik |
| 10575209 | -1.03772 | 5.821955 | 7.787487 | 7.55E-08 | 1.03E-06 | A430107J10Rik |
| 10512747 | -1.05112 | 6.73122 | 7.769258 | 7.85E-08 | 1.07E-06 | 5830415F09Rik |
| 10500610 | -1.41313 | 9.520235 | 7.764004 | 7.94E-08 | 1.08E-06 | Fam46c |
| 10364601 | -1.35156 | 6.648924 | 7.763703 | 7.95E-08 | 1.08E-06 | Abca7 |
| 10502359 | -1.35333 | 6.586061 | 7.761866 | 7.98E-08 | 1.09E-06 | Dapp1 |
| 10592891 | 1.012963 | 6.295061 | -7.75844 | 8.04E-08 | 1.09E-06 | Phldb1 |
| 10499536 | 1.180701 | 6.093945 | -7.7407 | 8.35E-08 | 1.13E-06 | Efna1 |
| 10447602 | -1.15783 | 7.791661 | 7.734903 | 8.45E-08 | 1.14E-06 | Ezr |
| 10513666 | -1.74295 | 7.256383 | 7.722295 | 8.69E-08 | 1.17E-06 | Akna |
| 10397645 | -1.29873 | 7.250816 | 7.720332 | 8.72E-08 | 1.18E-06 | Gpr65 |
| 10495574 | -1.22571 | 7.0212 | 7.717586 | 8.78E-08 | 1.18E-06 | Sass6 |
| 10375062 | -1.51127 | 5.380797 | 7.707278 | 8.97E-08 | 1.21E-06 | Hbq1a |
| 10418848 | -1.18834 | 7.177268 | 7.706343 | 8.99E-08 | 1.21E-06 | Wdfy4 |
| 10425333 | -1.48225 | 6.692652 | 7.698371 | 9.15E-08 | 1.23E-06 | Apobec3 |
| 10591517 | -1.21369 | 7.451472 | 7.694788 | 9.22E-08 | 1.23E-06 | Cdkn2d |
| 10590918 | 1.139465 | 7.254632 | -7.69054 | 9.30E-08 | 1.24E-06 | Amotl1 |
| 10600500 | -1.1703 | 8.002114 | 7.688975 | 9.33E-08 | 1.25E-06 | Fam220a |
| 10473399 | -2.23927 | 9.21392 | 7.688229 | 9.35E-08 | 1.25E-06 | Prg2 |
| 10559248 | -1.55782 | 6.599509 | 7.688148 | 9.35E-08 | 1.25E-06 | Tspan32 |
| 10415392 | -1.31888 | 7.18415 | 7.682448 | 9.47E-08 | 1.26E-06 | Ltb4r1 |
| 10414548 | -1.48363 | 7.084863 | 7.679069 | 9.54E-08 | 1.27E-06 | Rnase6 |
| 10360018 | -1.05796 | 5.150603 | 7.677746 | 9.56E-08 | 1.27E-06 | Fcrla |
| 10450412 | -2.12768 | 6.688794 | 7.677125 | 9.58E-08 | 1.27E-06 | G6b |
| 10547664 | -1.4577 | 6.004176 | 7.671654 | 9.69E-08 | 1.28E-06 | Clec4e |
| 10360090 | -1.57939 | 7.569108 | 7.670304 | 9.72E-08 | 1.28E-06 | Ppox |
| 10450675 | -1.40023 | 6.878973 | 7.655841 | 1.00E-07 | 1.32E-06 | H2-T24 |
| 10366746 | 1.087152 | 5.32226 | -7.6513 | 1.01E-07 | 1.33E-06 | Lrig3 |
| 10554445 | -1.26297 | 7.982744 | 7.646367 | 1.02E-07 | 1.35E-06 | Prc1 |
| 10360985 | -1.09716 | 7.634287 | 7.645553 | 1.03E-07 | 1.35E-06 | Cenpf |
| 10541260 | -1.4157 | 5.753238 | 7.645457 | 1.03E-07 | 1.35E-06 | Cecr2 |
| 10545812 | -1.16007 | 6.233862 | 7.643597 | 1.03E-07 | 1.35E-06 | Sfxn5 |
| 10597182 | -1.46928 | 6.898567 | 7.643489 | 1.03E-07 | 1.35E-06 | Nbeal2 |
| 10387797 | 1.084403 | 5.465082 | -7.64338 | 1.03E-07 | 1.35E-06 | Bcl6b |
| 10352178 | 1.346454 | 7.163622 | -7.63819 | 1.04E-07 | 1.37E-06 | Sccpdh |
| 10566943 | -1.40652 | 6.326777 | 7.633479 | 1.05E-07 | 1.38E-06 | Mrvi1 |
| 10547769 | -1.00942 | 8.041795 | 7.633247 | 1.05E-07 | 1.38E-06 | Ptpn6 |
| 10475990 | -1.1378 | 8.099064 | 7.632822 | 1.05E-07 | 1.38E-06 | Slc20a1 |
| 10358583 | 1.479899 | 5.252892 | -7.62863 | 1.06E-07 | 1.39E-06 | Hmcn1 |
| 10487577 | -1.08903 | 6.253047 | 7.623887 | 1.07E-07 | 1.40E-06 | Ckap2l |
| 10535006 | -1.17007 | 7.033625 | 7.622211 | 1.08E-07 | 1.41E-06 | BC037034 |
| 10407072 | -1.38327 | 5.317069 | 7.614171 | 1.10E-07 | 1.43E-06 | Elovl7 |
| 10545096 | -2.00874 | 5.480131 | 7.60976 | 1.11E-07 | 1.44E-06 | Mageb16 |
| 10361055 | 1.165226 | 5.135773 | -7.6092 | 1.11E-07 | 1.44E-06 | Vash2 |
| 10457168 | -2.12297 | 6.482068 | 7.607389 | 1.11E-07 | 1.45E-06 | Cd226 |
| 10452316 | -1.64815 | 8.549192 | 7.606215 | 1.12E-07 | 1.45E-06 | C3 |
| 10497490 | 1.025232 | 5.257603 | -7.59034 | 1.16E-07 | 1.49E-06 | Naaladl2 |
| 10572378 | 1.566903 | 7.105671 | -7.58776 | 1.16E-07 | 1.50E-06 | Comp |
| 10358224 | -1.06874 | 9.200303 | 7.586514 | 1.17E-07 | 1.50E-06 | Ptprc |
| 10379633 | -1.75923 | 8.44525 | 7.581078 | 1.18E-07 | 1.52E-06 | Slfn1 |
| 10419082 | -1.89415 | 7.996862 | 7.580393 | 1.18E-07 | 1.52E-06 | Fam213a |
| 10412667 | 1.086771 | 6.187826 | -7.57949 | 1.18E-07 | 1.52E-06 | Ptprg |
| 10598018 | -1.40919 | 8.894231 | 7.570208 | 1.21E-07 | 1.54E-06 | mt-Tf |
| 10582626 | -1.51102 | 6.673399 | 7.567233 | 1.22E-07 | 1.55E-06 | Abcb10 |
| 10358529 | 1.179841 | 5.046578 | -7.56413 | 1.22E-07 | 1.56E-06 | Hmcn1 |
| 10596166 | -1.26922 | 6.202626 | 7.563447 | 1.23E-07 | 1.56E-06 | 1300017J02Rik |
| 10597279 | -1.35384 | 5.870841 | 7.553557 | 1.25E-07 | 1.60E-06 | Ccrl2 |
| 10586017 | 1.420652 | 8.264586 | -7.54189 | 1.28E-07 | 1.63E-06 | Uaca |
| 10369481 | 1.085867 | 6.808531 | -7.54163 | 1.29E-07 | 1.63E-06 | H2afy2 |
| 10435581 | -1.14527 | 6.205758 | 7.537676 | 1.30E-07 | 1.64E-06 | Polq |
| 10354897 | -1.18163 | 8.8692 | 7.535802 | 1.30E-07 | 1.65E-06 | Trak2 |
| 10552697 | -1.52132 | 6.580945 | 7.52683 | 1.33E-07 | 1.68E-06 | Napsa |
| 10594798 | -1.86491 | 6.055708 | 7.51851 | 1.35E-07 | 1.70E-06 | Gm23730 |
| 10421970 | -1.45836 | 4.495541 | 7.517415 | 1.36E-07 | 1.70E-06 | Gm25831 |
| 10453260 | -1.01434 | 5.585509 | 7.516134 | 1.36E-07 | 1.71E-06 | Haao |
| 10397364 | 1.008807 | 5.604749 | -7.51522 | 1.36E-07 | 1.71E-06 | Mfsd7c |
| 10351347 | -1.21244 | 9.56388 | 7.506556 | 1.39E-07 | 1.74E-06 | Creg1 |
| 10358559 | 1.412985 | 6.080154 | -7.50655 | 1.39E-07 | 1.74E-06 | Hmcn1 |
| 10542857 | -1.17273 | 5.595146 | 7.498895 | 1.41E-07 | 1.76E-06 | Far2 |
| 10578690 | -1.43522 | 7.259354 | 7.496898 | 1.42E-07 | 1.77E-06 | Neil3 |
| 10605338 | -1.09693 | 8.533656 | 7.483131 | 1.46E-07 | 1.82E-06 | G6pdx |
| 10384233 | 1.072613 | 8.338149 | -7.47912 | 1.47E-07 | 1.83E-06 | Tns3 |
| 10567108 | -1.33332 | 7.423054 | 7.474915 | 1.49E-07 | 1.84E-06 | Sox6 |
| 10544596 | 1.077172 | 8.709371 | -7.47105 | 1.50E-07 | 1.86E-06 | Tmem176b |
| 10591643 | -1.06208 | 6.18739 | 7.469627 | 1.50E-07 | 1.86E-06 | Rab3d |
| 10425866 | -1.71099 | 6.997423 | 7.462766 | 1.53E-07 | 1.89E-06 | Parvg |
| 10515613 | 1.010383 | 6.357778 | -7.46023 | 1.54E-07 | 1.89E-06 | Ptprf |
| 10499062 | -1.57831 | 6.872539 | 7.458308 | 1.54E-07 | 1.90E-06 | Fhdc1 |
| 10462281 | 1.59369 | 7.920255 | -7.45702 | 1.55E-07 | 1.90E-06 | Vldlr |
| 10429573 | -1.27184 | 9.900152 | 7.456091 | 1.55E-07 | 1.91E-06 | Ly6c2 |
| 10505931 | 1.055759 | 5.435852 | -7.42977 | 1.64E-07 | 2.01E-06 | Ift74 |
| 10525236 | -1.31484 | 7.852786 | 7.426825 | 1.65E-07 | 2.02E-06 | Gm15800 |
| 10369842 | 1.747734 | 7.94638 | -7.42145 | 1.67E-07 | 2.04E-06 | Bicc1 |
| 10607475 | 1.045838 | 8.866765 | -7.42078 | 1.67E-07 | 2.04E-06 | Prdx4 |
| 10421456 | -1.46966 | 9.725152 | 7.414217 | 1.70E-07 | 2.07E-06 | Xpo7 |
| 10547469 | -1.16923 | 8.300475 | 7.413584 | 1.70E-07 | 2.07E-06 | Wnk1 |
| 10575019 | 1.860547 | 5.495755 | -7.4111 | 1.71E-07 | 2.08E-06 | Gm10629 |
| 10419416 | 1.418619 | 5.296255 | -7.41059 | 1.71E-07 | 2.08E-06 | 3632451O06Rik |
| 10492231 | -1.21217 | 5.524365 | 7.407779 | 1.72E-07 | 2.09E-06 | Med12l |
| 10573419 | -1.35866 | 7.389975 | 7.402723 | 1.74E-07 | 2.11E-06 | Lyl1 |
| 10501020 | -2.47331 | 10.23971 | 7.402104 | 1.75E-07 | 2.11E-06 | Chil3 |
| 10463123 | -1.52762 | 4.960877 | 7.399649 | 1.75E-07 | 2.12E-06 | Dntt |
| 10523891 | -1.07926 | 5.189553 | 7.39411 | 1.78E-07 | 2.15E-06 | Ube2d2b |
| 10564713 | 1.103431 | 7.06123 | -7.38474 | 1.81E-07 | 2.19E-06 | Mfge8 |
| 10578986 | 1.148339 | 5.966723 | -7.38294 | 1.82E-07 | 2.19E-06 | Psd3 |
| 10459391 | -1.33239 | 7.979922 | 7.3786 | 1.84E-07 | 2.21E-06 | Fech |
| 10409567 | -1.4241 | 5.893302 | 7.377577 | 1.84E-07 | 2.22E-06 | Tifab |
| 10446553 | 1.058344 | 6.064638 | -7.37462 | 1.85E-07 | 2.23E-06 | Epb4.1l3 |
| 10596815 | -1.34569 | 8.029821 | 7.371823 | 1.87E-07 | 2.24E-06 | Rnf123 |
| 10367532 | -1.02307 | 4.186699 | 7.352327 | 1.95E-07 | 2.32E-06 | Tespa1 |
| 10498386 | 1.237602 | 7.065762 | -7.35147 | 1.95E-07 | 2.33E-06 | Igsf10 |
| 10438907 | -2.2475 | 7.847881 | 7.343449 | 1.99E-07 | 2.36E-06 | Gp5 |
| 10348451 | 1.13462 | 6.551044 | -7.33884 | 2.01E-07 | 2.38E-06 | Ackr3 |
| 10605674 | -1.12428 | 7.290558 | 7.336416 | 2.02E-07 | 2.39E-06 | Pola1 |
| 10456140 | -1.01345 | 5.24367 | 7.321545 | 2.08E-07 | 2.46E-06 | Sh3tc2 |
| 10345752 | -1.26861 | 5.866458 | 7.321499 | 2.08E-07 | 2.46E-06 | Il1r2 |
| 10347968 | -1.09992 | 4.942797 | 7.318392 | 2.10E-07 | 2.48E-06 | n-R5s215 |
| 10455015 | 1.447433 | 7.201562 | -7.31154 | 2.13E-07 | 2.51E-06 | Vaultrc5 |
| 10442691 | 1.060827 | 6.257714 | -7.30714 | 2.15E-07 | 2.53E-06 | Clcn7 |
| 10523709 | 1.191918 | 6.833502 | -7.30345 | 2.17E-07 | 2.54E-06 | Mepe |
| 10481592 | 1.040719 | 6.831759 | -7.30268 | 2.17E-07 | 2.54E-06 | Dnm1 |
| 10480891 | -1.24973 | 8.149763 | 7.292821 | 2.22E-07 | 2.59E-06 | Ubac1 |
| 10594251 | -1.13792 | 7.873781 | 7.289259 | 2.24E-07 | 2.61E-06 | Kif23 |
| 10358421 | -2.01793 | 7.928503 | 7.288699 | 2.24E-07 | 2.61E-06 | Rgs18 |
| 10498345 | -1.21574 | 6.362406 | 7.288672 | 2.24E-07 | 2.61E-06 | Gpr171 |
| 10359582 | -1.29026 | 5.939935 | 7.285264 | 2.26E-07 | 2.63E-06 | Fmo2 |
| 10566583 | -1.60952 | 8.340745 | 7.274784 | 2.31E-07 | 2.68E-06 | Gm8995 |
| 10431659 | 1.105804 | 5.501829 | -7.26902 | 2.34E-07 | 2.71E-06 | Kif21a |
| 10587231 | 1.337448 | 6.787712 | -7.26852 | 2.34E-07 | 2.71E-06 | Bmp5 |
| 10364535 | -2.37702 | 8.755242 | 7.261792 | 2.38E-07 | 2.75E-06 | Elane |
| 10358555 | 1.413125 | 6.23633 | -7.25084 | 2.44E-07 | 2.80E-06 | Hmcn1 |
| 10562720 | -1.58254 | 7.34845 | 7.245373 | 2.47E-07 | 2.83E-06 | Siglece |
| 10415411 | 1.005523 | 5.974023 | -7.24491 | 2.47E-07 | 2.83E-06 | Nynrin |
| 10525210 | -1.32049 | 7.892397 | 7.243179 | 2.48E-07 | 2.84E-06 | Gm15800 |
| 10402991 | -1.53647 | 6.86991 | 7.242191 | 2.49E-07 | 2.85E-06 | Ighv2-4 |
| 10365983 | 1.973555 | 10.5492 | -7.23565 | 2.52E-07 | 2.88E-06 | Lum |
| 10548552 | -1.28491 | 6.795245 | 7.227577 | 2.57E-07 | 2.93E-06 | Klra2 |
| 10366052 | 1.13174 | 6.926779 | -7.22721 | 2.57E-07 | 2.93E-06 | Kitl |
| 10356271 | -1.34917 | 7.715485 | 7.225231 | 2.58E-07 | 2.94E-06 | A530032D15Rik |
| 10598077 | -1.73707 | 8.743715 | 7.223531 | 2.59E-07 | 2.95E-06 | mt-Tn |
| 10359307 | 1.659353 | 6.390693 | -7.21838 | 2.62E-07 | 2.98E-06 | Tnn |
| 10562812 | -2.02707 | 6.528863 | 7.203945 | 2.71E-07 | 3.07E-06 | Spib |
| 10434191 | -1.0685 | 6.2998 | 7.202183 | 2.72E-07 | 3.08E-06 | Txnrd2 |
| 10411622 | -1.14245 | 5.157902 | 7.201695 | 2.72E-07 | 3.08E-06 | Naip6 |
| 10471486 | 1.308262 | 7.275347 | -7.19517 | 2.76E-07 | 3.12E-06 | Eng |
| 10356248 | -1.4326 | 6.559752 | 7.195064 | 2.76E-07 | 3.12E-06 | C130026I21Rik |
| 10590494 | -1.22734 | 7.561826 | 7.193963 | 2.77E-07 | 3.12E-06 | Kif15 |
| 10448559 | -1.04797 | 4.375123 | 7.191095 | 2.78E-07 | 3.14E-06 | D330041H03Rik |
| 10387536 | 1.558966 | 9.434303 | -7.18477 | 2.82E-07 | 3.18E-06 | Cd68 |
| 10473349 | -2.29562 | 7.78346 | 7.173383 | 2.90E-07 | 3.25E-06 | Ypel4 |
| 10375259 | 1.294005 | 3.914143 | -7.17264 | 2.90E-07 | 3.25E-06 | Gabrb2 |
| 10573261 | -1.55066 | 7.087272 | 7.172359 | 2.90E-07 | 3.25E-06 | Asf1b |
| 10381708 | -1.23902 | 6.795536 | 7.171832 | 2.91E-07 | 3.25E-06 | Fmnl1 |
| 10598057 | -1.73813 | 6.81964 | 7.170296 | 2.92E-07 | 3.26E-06 | mt-Tr |
| 10407081 | -1.36068 | 6.244242 | 7.169185 | 2.92E-07 | 3.27E-06 | Depdc1b |
| 10461723 | -1.0003 | 8.847437 | 7.161515 | 2.97E-07 | 3.32E-06 | Fam111a |
| 10548875 | -1.43764 | 6.596212 | 7.14911 | 3.06E-07 | 3.40E-06 | Art4 |
| 10408689 | 1.226065 | 4.935689 | -7.14855 | 3.06E-07 | 3.40E-06 | Nrn1 |
| 10473356 | -1.75544 | 9.091103 | 7.14435 | 3.09E-07 | 3.43E-06 | Ube2l6 |
| 10604832 | -1.20149 | 6.229312 | 7.14114 | 3.11E-07 | 3.45E-06 | Mir505 |
| 10505568 | 1.264978 | 4.435717 | -7.13445 | 3.16E-07 | 3.50E-06 | Frmd3 |
| 10391697 | -1.88872 | 6.881142 | 7.134145 | 3.16E-07 | 3.50E-06 | Itga2b |
| 10541301 | -1.54427 | 7.783331 | 7.129273 | 3.20E-07 | 3.53E-06 | Tuba8 |
| 10439292 | -2.0413 | 7.597903 | 7.129245 | 3.20E-07 | 3.53E-06 | Stfa1 |
| 10575548 | -1.07809 | 7.421635 | 7.127445 | 3.21E-07 | 3.55E-06 | Gm26132 |
| 10541246 | -1.15844 | 7.917648 | 7.119586 | 3.27E-07 | 3.60E-06 | Il17ra |
| 10583314 | -1.04027 | 10.03593 | 7.118544 | 3.27E-07 | 3.61E-06 | Gm24357 |
| 10498379 | 1.234744 | 7.07577 | -7.10927 | 3.34E-07 | 3.67E-06 | Igsf10 |
| 10413710 | 1.404954 | 6.884861 | -7.08852 | 3.50E-07 | 3.82E-06 | Nt5dc2 |
| 10438445 | -1.24501 | 7.500907 | 7.087563 | 3.51E-07 | 3.83E-06 | Klhl6 |
| 10541614 | -1.30566 | 7.18614 | 7.080566 | 3.56E-07 | 3.88E-06 | Clec4d |
| 10450501 | -1.12312 | 5.942078 | 7.078123 | 3.58E-07 | 3.90E-06 | Tnf |
| 10539669 | -1.26548 | 5.935981 | 7.059186 | 3.74E-07 | 4.05E-06 | Add2 |
| 10383564 | -1.49527 | 6.322419 | 7.056748 | 3.76E-07 | 4.06E-06 | Fn3k |
| 10379630 | -1.10156 | 8.455073 | 7.052384 | 3.80E-07 | 4.09E-06 | Slfn2 |
| 10521667 | -1.21272 | 5.701854 | 7.048407 | 3.83E-07 | 4.12E-06 | Bst1 |
| 10389339 | -1.13136 | 8.993873 | 7.048316 | 3.83E-07 | 4.12E-06 | Usp32 |
| 10402211 | 1.240527 | 7.735904 | -7.04258 | 3.88E-07 | 4.17E-06 | Fbln5 |
| 10419223 | 1.242716 | 7.57815 | -7.04162 | 3.89E-07 | 4.18E-06 | Fermt2 |
| 10407122 | -1.01476 | 6.763762 | 7.039201 | 3.91E-07 | 4.20E-06 | Pde4d |
| 10403028 | -1.9561 | 5.914822 | 7.03702 | 3.93E-07 | 4.22E-06 | Ighv1-52 |
| 10363962 | -1.60881 | 5.855891 | 7.031591 | 3.98E-07 | 4.26E-06 | Gnaz |
| 10518147 | 1.295386 | 6.866718 | -7.02793 | 4.01E-07 | 4.29E-06 | Pdpn |
| 10430818 | -1.1466 | 6.886446 | 7.025823 | 4.03E-07 | 4.31E-06 | Tnfrsf13c |
| 10462632 | -1.00654 | 6.899537 | 7.021907 | 4.07E-07 | 4.34E-06 | Kif20b |
| 10499378 | -1.94124 | 7.179482 | 7.015919 | 4.12E-07 | 4.40E-06 | Sema4a |
| 10395409 | 1.275946 | 6.244243 | -7.01096 | 4.17E-07 | 4.44E-06 | Meox2 |
| 10525591 | -1.18807 | 6.515402 | 7.009182 | 4.18E-07 | 4.45E-06 | Kntc1 |
| 10349559 | -1.03191 | 6.979292 | 7.00388 | 4.23E-07 | 4.50E-06 | Yod1 |
| 10497372 | -1.3569 | 7.462181 | 6.991684 | 4.35E-07 | 4.61E-06 | Gm5150 |
| 10481627 | -2.6469 | 10.92164 | 6.990979 | 4.36E-07 | 4.61E-06 | Lcn2 |
| 10392796 | -1.15983 | 5.851157 | 6.990702 | 4.36E-07 | 4.61E-06 | Cd300lb |
| 10437399 | -1.27619 | 7.158907 | 6.988202 | 4.39E-07 | 4.63E-06 | Coro7 |
| 10580349 | -1.16694 | 5.91365 | 6.982674 | 4.44E-07 | 4.69E-06 | Mylk3 |
| 10462111 | 1.065356 | 7.542127 | -6.97748 | 4.49E-07 | 4.73E-06 | Gm9938 |
| 10476252 | -1.23028 | 7.363391 | 6.975733 | 4.51E-07 | 4.75E-06 | Cdc25b |
| 10530633 | 1.038204 | 7.124988 | -6.9744 | 4.53E-07 | 4.76E-06 | Sgcb |
| 10576034 | -1.02257 | 7.31493 | 6.970464 | 4.57E-07 | 4.79E-06 | Irf8 |
| 10499160 | 1.115044 | 5.775947 | -6.96718 | 4.60E-07 | 4.82E-06 | Cd1d1 |
| 10550956 | -1.11176 | 7.274535 | 6.965168 | 4.62E-07 | 4.84E-06 | Ethe1 |
| 10566326 | -1.04332 | 7.063174 | 6.964418 | 4.63E-07 | 4.84E-06 | Trim12a |
| 10408321 | -1.05196 | 5.844886 | 6.960619 | 4.67E-07 | 4.88E-06 | Gmnn |
| 10577164 | 1.081452 | 8.357516 | -6.95808 | 4.70E-07 | 4.90E-06 | Gas6 |
| 10363575 | -1.03437 | 6.644949 | 6.956432 | 4.71E-07 | 4.92E-06 | Dna2 |
| 10421186 | -1.91874 | 6.145775 | 6.954911 | 4.73E-07 | 4.93E-06 | Gm10002 |
| 10466224 | -1.99458 | 8.30385 | 6.951346 | 4.77E-07 | 4.96E-06 | Ms4a3 |
| 10347919 | -1.2421 | 5.494783 | 6.946918 | 4.82E-07 | 5.00E-06 | LOC102634459 |
| 10356291 | -1.2421 | 5.494783 | 6.946918 | 4.82E-07 | 5.00E-06 | LOC102634459 |
| 10421172 | -2.04462 | 9.516874 | 6.943093 | 4.86E-07 | 5.04E-06 | Slc25a37 |
| 10364262 | -1.07518 | 9.008253 | 6.94193 | 4.87E-07 | 5.05E-06 | Itgb2 |
| 10358547 | 1.385082 | 4.847353 | -6.93848 | 4.91E-07 | 5.09E-06 | Hmcn1 |
| 10355806 | -1.91555 | 9.976242 | 6.936356 | 4.93E-07 | 5.11E-06 | Tuba4a |
| 10403060 | -2.01976 | 8.461943 | 6.935198 | 4.94E-07 | 5.12E-06 | Igh-VJ558 |
| 10516246 | -1.37377 | 7.655223 | 6.918009 | 5.14E-07 | 5.30E-06 | Cdca8 |
| 10539135 | 1.004408 | 8.131217 | -6.91311 | 5.20E-07 | 5.35E-06 | Capg |
| 10479041 | -2.08056 | 8.68752 | 6.908578 | 5.25E-07 | 5.40E-06 | Rbm38 |
| 10474875 | -1.36873 | 8.702214 | 6.904043 | 5.31E-07 | 5.45E-06 | Casc5 |
| 10347921 | -1.47435 | 7.045954 | 6.90383 | 5.31E-07 | 5.45E-06 | A530040E14Rik |
| 10445789 | -1.68159 | 7.763645 | 6.901632 | 5.34E-07 | 5.47E-06 | Treml1 |
| 10400941 | -1.00213 | 7.238067 | 6.899443 | 5.36E-07 | 5.50E-06 | Dhrs7 |
| 10379127 | -1.16223 | 7.172478 | 6.898883 | 5.37E-07 | 5.50E-06 | Spag5 |
| 10483401 | -1.00172 | 6.984505 | 6.886675 | 5.52E-07 | 5.63E-06 | Spc25 |
| 10404132 | -1.59332 | 6.087319 | 6.885111 | 5.54E-07 | 5.65E-06 | Cmah |
| 10591660 | -1.49655 | 6.638978 | 6.876011 | 5.65E-07 | 5.75E-06 | Epor |
| 10399908 | -1.27723 | 8.393342 | 6.873609 | 5.68E-07 | 5.78E-06 | Prkar2b |
| 10479154 | -1.99856 | 6.829477 | 6.851406 | 5.98E-07 | 6.03E-06 | Tubb1 |
| 10434668 | 1.099818 | 6.907495 | -6.84257 | 6.10E-07 | 6.15E-06 | Tmem97 |
| 10563114 | 1.940112 | 10.04796 | -6.83526 | 6.20E-07 | 6.24E-06 | Snord32a |
| 10599581 | -1.18408 | 7.178785 | 6.833827 | 6.22E-07 | 6.25E-06 | 2610018G03Rik |
| 10598079 | -2.23914 | 5.547621 | 6.831107 | 6.26E-07 | 6.29E-06 | mt-Tc |
| 10359689 | -1.257 | 7.15242 | 6.817322 | 6.46E-07 | 6.46E-06 | Atp1b1 |
| 10517116 | -1.45095 | 7.633209 | 6.805549 | 6.64E-07 | 6.62E-06 | Rps6ka1 |
| 10414065 | 1.392994 | 5.866898 | -6.80386 | 6.66E-07 | 6.64E-06 | Anxa8 |
| 10517165 | -1.69982 | 10.53256 | 6.797562 | 6.76E-07 | 6.73E-06 | Cd52 |
| 10578810 | -1.34098 | 7.17402 | 6.790345 | 6.87E-07 | 6.83E-06 | Clcn3 |
| 10403834 | 1.144721 | 9.494146 | -6.78862 | 6.90E-07 | 6.85E-06 | Sfrp4 |
| 10495035 | -1.05182 | 7.624542 | 6.782793 | 6.99E-07 | 6.93E-06 | Slc16a1 |
| 10594825 | -1.39162 | 6.591823 | 6.782638 | 6.99E-07 | 6.93E-06 | Aqp9 |
| 10366196 | 1.204366 | 5.785123 | -6.7809 | 7.02E-07 | 6.95E-06 | Ppfia2 |
| 10582868 | -1.01702 | 8.791367 | 6.771177 | 7.18E-07 | 7.08E-06 | LOC546061 |
| 10362899 | -1.17579 | 7.228345 | 6.76572 | 7.27E-07 | 7.16E-06 | F830002L21Rik |
| 10347948 | -1.15415 | 8.504737 | 6.761979 | 7.33E-07 | 7.21E-06 | Sp100 |
| 10374400 | -1.02281 | 4.56232 | 6.754585 | 7.45E-07 | 7.32E-06 | Fbxo48 |
| 10464560 | -1.04827 | 6.440988 | 6.751335 | 7.51E-07 | 7.36E-06 | Aldh3b1 |
| 10515257 | -1.07904 | 6.797104 | 6.736796 | 7.76E-07 | 7.58E-06 | Rad54l |
| 10433101 | -1.01687 | 5.364292 | 6.733023 | 7.83E-07 | 7.64E-06 | Gpr84 |
| 10483150 | 1.002897 | 4.931751 | -6.73264 | 7.84E-07 | 7.64E-06 | Fign |
| 10476939 | 1.042771 | 7.652142 | -6.73089 | 7.87E-07 | 7.66E-06 | Zfp937 |
| 10579958 | -1.02818 | 6.150636 | 6.72955 | 7.89E-07 | 7.68E-06 | Il15 |
| 10376201 | 1.110392 | 10.39828 | -6.71856 | 8.09E-07 | 7.85E-06 | Gpx3 |
| 10567863 | -1.56853 | 6.133034 | 6.707267 | 8.31E-07 | 8.03E-06 | Cd19 |
| 10582860 | -1.13773 | 5.458213 | 6.703214 | 8.38E-07 | 8.09E-06 | LOC102634459 |
| 10582877 | -1.13773 | 5.458213 | 6.703214 | 8.38E-07 | 8.09E-06 | LOC102634459 |
| 10549108 | 1.028879 | 8.136822 | -6.70262 | 8.40E-07 | 8.09E-06 | Abcc9 |
| 10378816 | -1.59407 | 5.773188 | 6.696412 | 8.52E-07 | 8.20E-06 | Slc6a4 |
| 10527229 | -1.27366 | 8.413163 | 6.694432 | 8.55E-07 | 8.22E-06 | Fam220a |
| 10389300 | -1.90744 | 7.881754 | 6.693821 | 8.57E-07 | 8.23E-06 | Dhrs11 |
| 10487588 | -1.74608 | 4.727021 | 6.68586 | 8.72E-07 | 8.37E-06 | Il1a |
| 10526654 | -1.0761 | 6.700771 | 6.685259 | 8.74E-07 | 8.37E-06 | Gm7285 |
| 10526656 | -1.26681 | 7.776709 | 6.678658 | 8.87E-07 | 8.49E-06 | Lrch4 |
| 10422760 | -1.20604 | 8.849544 | 6.675994 | 8.92E-07 | 8.54E-06 | Fyb |
| 10433096 | -1.74564 | 8.34151 | 6.674791 | 8.95E-07 | 8.56E-06 | Nfe2 |
| 10375055 | -1.87514 | 7.686202 | 6.674096 | 8.96E-07 | 8.57E-06 | Hbq1b |
| 10514049 | 1.357204 | 8.656423 | -6.66965 | 9.05E-07 | 8.65E-06 | Nfib |
| 10448506 | -1.1434 | 6.850279 | 6.661188 | 9.23E-07 | 8.79E-06 | Ccnf |
| 10403048 | -2.10481 | 9.055897 | 6.66024 | 9.25E-07 | 8.81E-06 | Ighv1-62-3 |
| 10461558 | -1.12475 | 7.138221 | 6.659148 | 9.28E-07 | 8.82E-06 | Slc15a3 |
| 10540738 | -1.01144 | 5.683492 | 6.636789 | 9.76E-07 | 9.22E-06 | Fancd2 |
| 10368243 | 1.034861 | 6.556061 | -6.63446 | 9.82E-07 | 9.26E-06 | Eya4 |
| 10494407 | -1.39353 | 8.01465 | 6.632559 | 9.86E-07 | 9.30E-06 | Hist2h2bb |
| 10358668 | 1.318949 | 5.623756 | -6.62751 | 9.97E-07 | 9.39E-06 | Hmcn1 |
| 10576883 | -1.04108 | 6.201793 | 6.624205 | 1.01E-06 | 9.44E-06 | Shcbp1 |
| 10419288 | -1.99317 | 6.434938 | 6.622463 | 1.01E-06 | 9.47E-06 | Gch1 |
| 10545202 | -1.25036 | 6.191187 | 6.620457 | 1.01E-06 | 9.51E-06 | LOC102642862 |
| 10377924 | -2.13464 | 7.259084 | 6.609771 | 1.04E-06 | 9.69E-06 | Gp1ba |
| 10482802 | -1.43208 | 7.181261 | 6.602986 | 1.06E-06 | 9.83E-06 | Cytip |
| 10351691 | -1.13362 | 5.077467 | 6.599924 | 1.06E-06 | 9.89E-06 | Slamf6 |
| 10461093 | -1.03321 | 7.961982 | 6.590634 | 1.09E-06 | 1.01E-05 | Pla2g16 |
| 10400748 | -1.03109 | 4.764864 | 6.580193 | 1.11E-06 | 1.03E-05 | Cdkl1 |
| 10382985 | -1.17488 | 6.383453 | 6.579136 | 1.12E-06 | 1.03E-05 | Afmid |
| 10595768 | -1.28499 | 5.611701 | 6.578225 | 1.12E-06 | 1.03E-05 | Pls1 |
| 10498620 | -1.12488 | 8.588758 | 6.575794 | 1.12E-06 | 1.04E-05 | Trim59 |
| 10350516 | 1.298201 | 6.217056 | -6.57286 | 1.13E-06 | 1.04E-05 | Ptgs2 |
| 10403063 | -1.49977 | 6.323114 | 6.571263 | 1.14E-06 | 1.05E-05 | Ighv8-12 |
| 10568150 | -1.10904 | 7.018455 | 6.569124 | 1.14E-06 | 1.05E-05 | Kif22 |
| 10472408 | 1.100931 | 5.000509 | -6.56666 | 1.15E-06 | 1.06E-05 | Csrnp3 |
| 10515755 | -1.73223 | 6.40327 | 6.556807 | 1.17E-06 | 1.08E-05 | Mpl |
| 10544383 | -2.25851 | 8.115566 | 6.554544 | 1.18E-06 | 1.08E-05 | Kel |
| 10403038 | -2.00026 | 5.946116 | 6.55351 | 1.18E-06 | 1.08E-05 | Ighv1-61 |
| 10387743 | -1.41863 | 7.189429 | 6.539511 | 1.22E-06 | 1.12E-05 | Slc2a4 |
| 10516966 | -1.13655 | 7.436752 | 6.536606 | 1.23E-06 | 1.12E-05 | Themis2 |
| 10416655 | -1.01556 | 5.118626 | 6.533769 | 1.24E-06 | 1.13E-05 | Zbtbd6 |
| 10462796 | -1.38076 | 8.504377 | 6.533133 | 1.24E-06 | 1.13E-05 | Kif11 |
| 10515028 | 1.224056 | 6.193318 | -6.53079 | 1.25E-06 | 1.14E-05 | Zfyve9 |
| 10485370 | -1.20827 | 4.05339 | 6.521933 | 1.27E-06 | 1.15E-05 | B230118H07Rik |
| 10347928 | -1.65971 | 6.442258 | 6.519419 | 1.28E-06 | 1.16E-05 | Sp110 |
| 10582874 | -1.65971 | 6.442258 | 6.519419 | 1.28E-06 | 1.16E-05 | Sp110 |
| 10358567 | 1.033443 | 4.61327 | -6.51511 | 1.29E-06 | 1.17E-05 | Hmcn1 |
| 10356262 | -1.52719 | 6.892276 | 6.50944 | 1.31E-06 | 1.18E-05 | Gm7609 |
| 10357698 | -1.35771 | 6.809488 | 6.503086 | 1.33E-06 | 1.20E-05 | Tmcc2 |
| 10545130 | -1.0147 | 9.283528 | 6.499865 | 1.34E-06 | 1.21E-05 | Gadd45a |
| 10352000 | -1.17445 | 4.837284 | 6.49337 | 1.36E-06 | 1.22E-05 | Kmo |
| 10586240 | -1.21107 | 9.41206 | 6.492024 | 1.36E-06 | 1.22E-05 | Dennd4a |
| 10423498 | 1.126538 | 9.432198 | -6.49037 | 1.37E-06 | 1.23E-05 | Dap |
| 10512669 | -1.28828 | 5.707813 | 6.489007 | 1.37E-06 | 1.23E-05 | Pax5 |
| 10598075 | -1.86245 | 8.658908 | 6.483894 | 1.39E-06 | 1.24E-05 | mt-Ta |
| 10346168 | -1.51803 | 5.800898 | 6.479707 | 1.40E-06 | 1.25E-05 | Stat4 |
| 10406736 | -2.26538 | 7.463631 | 6.477792 | 1.41E-06 | 1.26E-05 | F2rl2 |
| 10419296 | -1.28083 | 7.32022 | 6.471711 | 1.43E-06 | 1.27E-05 | Wdhd1 |
| 10536908 | -1.99221 | 7.60026 | 6.470445 | 1.43E-06 | 1.28E-05 | Tspan33 |
| 10406519 | 2.024218 | 5.02764 | -6.45683 | 1.48E-06 | 1.31E-05 | Hapln1 |
| 10355327 | -1.14242 | 6.81133 | 6.449532 | 1.51E-06 | 1.33E-05 | Bard1 |
| 10569008 | -1.88008 | 10.92993 | 6.441761 | 1.53E-06 | 1.35E-05 | Cox8b |
| 10379176 | -1.23257 | 6.340243 | 6.437881 | 1.55E-06 | 1.36E-05 | Unc119 |
| 10358670 | 1.277601 | 5.6459 | -6.43653 | 1.55E-06 | 1.37E-05 | Hmcn1 |
| 10376778 | 1.270425 | 6.563513 | -6.43217 | 1.57E-06 | 1.38E-05 | Mfap4 |
| 10474201 | -1.03911 | 8.957539 | 6.427818 | 1.58E-06 | 1.39E-05 | Lmo2 |
| 10498367 | -1.07581 | 6.817132 | 6.425706 | 1.59E-06 | 1.40E-05 | P2ry13 |
| 10598032 | -1.27613 | 8.699453 | 6.424648 | 1.59E-06 | 1.40E-05 | mt-Tm |
| 10368720 | -1.37418 | 7.61206 | 6.420863 | 1.61E-06 | 1.41E-05 | Slc16a10 |
| 10438060 | -1.44424 | 6.288094 | 6.412356 | 1.64E-06 | 1.44E-05 | Igll1 |
| 10516823 | -1.38993 | 9.47314 | 6.412194 | 1.64E-06 | 1.44E-05 | Epb4.1 |
| 10358650 | 1.188131 | 5.737823 | -6.40811 | 1.66E-06 | 1.45E-05 | Hmcn1 |
| 10461152 | -1.06449 | 6.615711 | 6.389954 | 1.73E-06 | 1.50E-05 | Gm24452 |
| 10523128 | -2.17005 | 11.24182 | 6.387372 | 1.74E-06 | 1.51E-05 | Ppbp |
| 10383556 | -1.05032 | 7.055557 | 6.386911 | 1.74E-06 | 1.51E-05 | Fn3krp |
| 10350102 | -1.11469 | 6.664223 | 6.377303 | 1.78E-06 | 1.54E-05 | Ptpn7 |
| 10428081 | -1.13417 | 6.159204 | 6.377285 | 1.78E-06 | 1.54E-05 | Hrsp12 |
| 10395414 | 1.134851 | 6.08111 | -6.37553 | 1.79E-06 | 1.54E-05 | Agmo |
| 10376885 | 1.258055 | 10.54883 | -6.37305 | 1.80E-06 | 1.55E-05 | Snord49b |
| 10581340 | -1.37977 | 8.073609 | 6.372212 | 1.80E-06 | 1.55E-05 | Ranbp10 |
| 10576090 | -1.17176 | 7.29221 | 6.370579 | 1.81E-06 | 1.56E-05 | Zfpm1 |
| 10427468 | -1.1873 | 7.998723 | 6.369202 | 1.81E-06 | 1.56E-05 | LOC102633612 |
| 10392440 | -1.37493 | 6.742222 | 6.368649 | 1.82E-06 | 1.56E-05 | Slc16a6 |
| 10509168 | -1.846 | 7.215773 | 6.365622 | 1.83E-06 | 1.57E-05 | E2f2 |
| 10430195 | -1.04743 | 6.325058 | 6.360053 | 1.85E-06 | 1.59E-05 | Apol8 |
| 10393320 | -1.4524 | 8.306755 | 6.358784 | 1.86E-06 | 1.60E-05 | Ube2o |
| 10602372 | -1.57759 | 9.780179 | 6.355731 | 1.87E-06 | 1.60E-05 | Alas2 |
| 10387821 | -2.28769 | 7.727667 | 6.355427 | 1.87E-06 | 1.61E-05 | Alox12 |
| 10436590 | 1.170982 | 4.999684 | -6.35395 | 1.88E-06 | 1.61E-05 | 2810055G20Rik |
| 10382956 | -1.38482 | 6.183876 | 6.353264 | 1.88E-06 | 1.61E-05 | Tmc8 |
| 10534909 | -1.44743 | 7.96967 | 6.348509 | 1.90E-06 | 1.63E-05 | Gm15753 |
| 10371379 | 1.056023 | 6.228439 | -6.3447 | 1.92E-06 | 1.64E-05 | Nuak1 |
| 10385118 | -1.23526 | 7.782224 | 6.344177 | 1.92E-06 | 1.64E-05 | Dock2 |
| 10393431 | -1.19717 | 7.562203 | 6.344001 | 1.92E-06 | 1.64E-05 | Tk1 |
| 10451670 | -1.66872 | 6.64839 | 6.337744 | 1.95E-06 | 1.66E-05 | Tspo2 |
| 10458195 | -1.11354 | 5.924305 | 6.336552 | 1.96E-06 | 1.66E-05 | Cdc25c |
| 10427402 | 1.029952 | 7.739114 | -6.3354 | 1.96E-06 | 1.66E-05 | Ghr |
| 10531146 | -1.03404 | 5.25736 | 6.331921 | 1.98E-06 | 1.67E-05 | Mkrn1-ps1 |
| 10374727 | -1.54518 | 7.180925 | 6.330904 | 1.98E-06 | 1.67E-05 | Bcl11a |
| 10546294 | -1.56418 | 7.756608 | 6.329395 | 1.99E-06 | 1.68E-05 | Nup210 |
| 10521731 | -1.21891 | 7.72886 | 6.329133 | 1.99E-06 | 1.68E-05 | Ncapg |
| 10467637 | -1.30192 | 7.46388 | 6.323073 | 2.02E-06 | 1.70E-05 | Arhgap19 |
| 10364529 | -2.23116 | 8.554456 | 6.320946 | 2.03E-06 | 1.71E-05 | Prtn3 |
| 10349157 | -1.47547 | 5.563798 | 6.32073 | 2.03E-06 | 1.71E-05 | Serpinb2 |
| 10424781 | -1.77661 | 9.014297 | 6.305364 | 2.11E-06 | 1.76E-05 | Grina |
| 10524790 | -1.02046 | 6.758789 | 6.290133 | 2.18E-06 | 1.82E-05 | Cit |
| 10556302 | -1.09424 | 7.384907 | 6.282593 | 2.22E-06 | 1.85E-05 | Ampd3 |
| 10503107 | -1.02586 | 7.650722 | 6.278863 | 2.24E-06 | 1.86E-05 | 6330407A03Rik |
| 10351873 | -1.55445 | 6.393898 | 6.276619 | 2.25E-06 | 1.87E-05 | Pyhin1 |
| 10604897 | -1.13272 | 4.820661 | 6.275268 | 2.26E-06 | 1.87E-05 | Gm22435 |
| 10404376 | -1.02471 | 7.114456 | 6.267207 | 2.30E-06 | 1.91E-05 | Agtr1a |
| 10486255 | -1.27447 | 5.474104 | 6.264144 | 2.32E-06 | 1.92E-05 | Oip5 |
| 10377982 | 1.282456 | 9.183998 | -6.25986 | 2.34E-06 | 1.93E-05 | Kif1c |
| 10504692 | -1.61768 | 8.073824 | 6.253132 | 2.38E-06 | 1.96E-05 | Tmod1 |
| 10500345 | -1.57379 | 6.347411 | 6.253006 | 2.38E-06 | 1.96E-05 | Terc |
| 10507112 | -1.25133 | 6.25343 | 6.241291 | 2.45E-06 | 2.01E-05 | Stil |
| 10601421 | 1.074874 | 5.715227 | -6.23361 | 2.49E-06 | 2.04E-05 | A630033H20Rik |
| 10350335 | -1.10998 | 8.222901 | 6.232824 | 2.50E-06 | 2.05E-05 | Hmbs |
| 10568174 | -1.09952 | 7.42705 | 6.226483 | 2.53E-06 | 2.08E-05 | Spn |
| 10604528 | -1.33687 | 7.427581 | 6.225687 | 2.54E-06 | 2.08E-05 | Mbnl3 |
| 10399087 | -1.19563 | 7.115181 | 6.219578 | 2.58E-06 | 2.10E-05 | Ncapg2 |
| 10421877 | -1.19501 | 7.273611 | 6.219268 | 2.58E-06 | 2.10E-05 | Diap3 |
| 10447649 | 1.13803 | 6.944133 | -6.21501 | 2.60E-06 | 2.12E-05 | Fndc1 |
| 10449893 | -1.12485 | 6.154309 | 6.212414 | 2.62E-06 | 2.13E-05 | Rasal3 |
| 10351667 | -1.4282 | 4.973498 | 6.211615 | 2.62E-06 | 2.13E-05 | Slamf1 |
| 10474984 | -1.1829 | 8.576075 | 6.208581 | 2.64E-06 | 2.15E-05 | Nusap1 |
| 10501063 | -1.16848 | 9.889961 | 6.20571 | 2.66E-06 | 2.16E-05 | Cd53 |
| 10356278 | -1.2719 | 8.966902 | 6.190785 | 2.76E-06 | 2.23E-05 | Sp110 |
| 10420261 | -2.11702 | 8.600152 | 6.18173 | 2.81E-06 | 2.27E-05 | Ctsg |
| 10599487 | -1.13835 | 6.336912 | 6.181467 | 2.82E-06 | 2.27E-05 | Sash3 |
| 10418927 | 1.037828 | 7.069111 | -6.18033 | 2.82E-06 | 2.28E-05 | Bmpr1a |
| 10421418 | -1.32731 | 7.853616 | 6.180176 | 2.83E-06 | 2.28E-05 | Dmtn |
| 10531724 | -1.46684 | 9.268332 | 6.180161 | 2.83E-06 | 2.28E-05 | Plac8 |
| 10429568 | -1.09172 | 10.19759 | 6.17242 | 2.88E-06 | 2.32E-05 | Ly6c1 |
| 10538706 | -1.71801 | 6.27107 | 6.168522 | 2.90E-06 | 2.34E-05 | Mmrn1 |
| 10601044 | 1.094154 | 8.073282 | -6.16493 | 2.93E-06 | 2.35E-05 | Gdpd2 |
| 10471880 | 1.376294 | 5.955375 | -6.16146 | 2.95E-06 | 2.37E-05 | Mir181b-2 |
| 10606694 | -1.06462 | 7.073574 | 6.160467 | 2.96E-06 | 2.37E-05 | Btk |
| 10601011 | -1.0652 | 7.140864 | 6.155812 | 2.99E-06 | 2.40E-05 | Kif4 |
| 10545175 | -2.006 | 7.186785 | 6.148605 | 3.04E-06 | 2.43E-05 | Igkv10-94 |
| 10370644 | -1.11484 | 6.05924 | 6.142862 | 3.08E-06 | 2.46E-05 | Prss57 |
| 10414590 | -2.33681 | 6.350501 | 6.138894 | 3.11E-06 | 2.48E-05 | Ear6 |
| 10541644 | -1.01563 | 5.090027 | 6.138096 | 3.12E-06 | 2.49E-05 | Cd163 |
| 10597098 | -1.83319 | 11.68131 | 6.134226 | 3.15E-06 | 2.51E-05 | Camp |
| 10445046 | -1.99984 | 7.942083 | 6.132137 | 3.16E-06 | 2.52E-05 | Trim10 |
| 10580210 | -1.09129 | 8.452605 | 6.11867 | 3.27E-06 | 2.59E-05 | Rad23a |
| 10372807 | 1.027449 | 7.73324 | -6.11824 | 3.27E-06 | 2.59E-05 | Msrb3 |
| 10474998 | -1.02315 | 5.756603 | 6.11805 | 3.27E-06 | 2.59E-05 | n-R5s204 |
| 10450496 | -1.22971 | 8.966033 | 6.117443 | 3.28E-06 | 2.60E-05 | Lst1 |
| 10507137 | -1.59772 | 7.715135 | 6.102945 | 3.39E-06 | 2.67E-05 | Pdzk1ip1 |
| 10404063 | -1.70888 | 7.994911 | 6.101936 | 3.40E-06 | 2.68E-05 | Hist1h2ab |
| 10502335 | -1.6251 | 6.715206 | 6.096094 | 3.44E-06 | 2.71E-05 | Bank1 |
| 10408197 | -1.11044 | 9.140532 | 6.095823 | 3.45E-06 | 2.71E-05 | Hist1h2bh |
| 10404389 | -1.01364 | 4.89102 | 6.093907 | 3.46E-06 | 2.72E-05 | Irf4 |
| 10351905 | -1.94824 | 8.067278 | 6.092677 | 3.47E-06 | 2.73E-05 | Spta1 |
| 10381211 | 1.175922 | 6.315689 | -6.08947 | 3.50E-06 | 2.74E-05 | Naglu |
| 10465861 | -1.01268 | 7.94604 | 6.088815 | 3.50E-06 | 2.75E-05 | Incenp |
| 10401068 | -1.54893 | 7.772394 | 6.084317 | 3.54E-06 | 2.77E-05 | Sptb |
| 10385248 | -1.23383 | 7.150728 | 6.081888 | 3.56E-06 | 2.78E-05 | Hmmr |
| 10568202 | -1.23583 | 7.315675 | 6.080813 | 3.57E-06 | 2.79E-05 | 44440 |
| 10508454 | -1.10828 | 9.153976 | 6.076064 | 3.61E-06 | 2.81E-05 | Bsdc1 |
| 10428672 | -1.05044 | 5.779059 | 6.0748 | 3.62E-06 | 2.82E-05 | Dscc1 |
| 10598064 | -1.61124 | 7.262875 | 6.073328 | 3.64E-06 | 2.83E-05 | mt-Ts2 |
| 10358662 | 1.091831 | 5.760663 | -6.06793 | 3.68E-06 | 2.86E-05 | Hmcn1 |
| 10515848 | -1.72048 | 8.642741 | 6.067408 | 3.69E-06 | 2.86E-05 | Ermap |
| 10403043 | -2.00779 | 7.789604 | 6.063798 | 3.72E-06 | 2.88E-05 | Ighv1-62-3 |
| 10472916 | -1.12087 | 7.081935 | 6.062874 | 3.73E-06 | 2.89E-05 | Cdca7 |
| 10568536 | 1.012573 | 5.931527 | -6.06119 | 3.74E-06 | 2.90E-05 | Cpxm2 |
| 10582303 | -1.21102 | 8.857293 | 6.057551 | 3.77E-06 | 2.92E-05 | Cyba |
| 10501222 | 1.09097 | 7.556189 | -6.05359 | 3.81E-06 | 2.94E-05 | Gstm2 |
| 10492971 | -1.20114 | 5.071808 | 6.043392 | 3.90E-06 | 3.01E-05 | Fcrl1 |
| 10566585 | -1.54088 | 7.590239 | 6.041086 | 3.92E-06 | 3.02E-05 | Gm1966 |
| 10358654 | 1.142897 | 6.386357 | -6.02562 | 4.07E-06 | 3.11E-05 | Hmcn1 |
| 10592515 | -1.46719 | 6.803965 | 6.022481 | 4.10E-06 | 3.13E-05 | Ubash3b |
| 10380174 | -1.97565 | 10.18182 | 6.019598 | 4.13E-06 | 3.15E-05 | Mpo |
| 10581643 | -1.00177 | 4.258849 | 6.018425 | 4.14E-06 | 3.16E-05 | Gm25321 |
| 10588786 | -1.07104 | 6.314107 | 6.015282 | 4.17E-06 | 3.18E-05 | Uba7 |
| 10490053 | -1.03487 | 6.467099 | 6.014369 | 4.18E-06 | 3.18E-05 | Zfp217 |
| 10564978 | -1.16332 | 6.461079 | 6.000784 | 4.32E-06 | 3.27E-05 | Blm |
| 10350392 | -1.2156 | 7.008538 | 5.99987 | 4.33E-06 | 3.28E-05 | Aspm |
| 10430931 | -1.13589 | 7.012314 | 5.99915 | 4.33E-06 | 3.28E-05 | Nfam1 |
| 10392142 | -1.58984 | 8.176813 | 5.989289 | 4.44E-06 | 3.35E-05 | Cd79b |
| 10478633 | 1.315821 | 10.50575 | -5.98757 | 4.45E-06 | 3.36E-05 | Mmp9 |
| 10598073 | -1.08329 | 10.75843 | 5.978086 | 4.56E-06 | 3.43E-05 | mt-Tq |
| 10523134 | -1.81663 | 10.45051 | 5.976465 | 4.57E-06 | 3.44E-05 | Pf4 |
| 10431915 | 1.293649 | 6.6664 | -5.96229 | 4.73E-06 | 3.54E-05 | Slc38a4 |
| 10427436 | -1.22481 | 5.491229 | 5.957456 | 4.78E-06 | 3.58E-05 | C7 |
| 10479221 | 1.025492 | 6.726691 | -5.95356 | 4.83E-06 | 3.61E-05 | Gm14403 |
| 10504668 | -1.06559 | 6.086557 | 5.948652 | 4.89E-06 | 3.65E-05 | E230008N13Rik |
| 10373768 | 1.290728 | 8.73939 | -5.93372 | 5.06E-06 | 3.76E-05 | Selm |
| 10588707 | -1.08977 | 7.842606 | 5.929726 | 5.11E-06 | 3.79E-05 | Ifrd2 |
| 10393064 | -1.06393 | 5.537317 | 5.92707 | 5.14E-06 | 3.81E-05 | Unc13d |
| 10391461 | -1.07539 | 6.589696 | 5.91019 | 5.35E-06 | 3.94E-05 | Brca1 |
| 10403959 | -1.02369 | 11.09007 | 5.910016 | 5.36E-06 | 3.94E-05 | Hist1h2bq |
| 10408087 | -1.02369 | 11.09007 | 5.910016 | 5.36E-06 | 3.94E-05 | Hist1h2bq |
| 10473125 | -1.05974 | 8.597372 | 5.889226 | 5.63E-06 | 4.11E-05 | Itga4 |
| 10419323 | -1.12115 | 7.65361 | 5.886733 | 5.66E-06 | 4.13E-05 | Dlgap5 |
| 10358658 | 1.033729 | 5.454135 | -5.88293 | 5.71E-06 | 4.16E-05 | Hmcn1 |
| 10531126 | -2.34281 | 7.937299 | 5.878628 | 5.77E-06 | 4.20E-05 | Igj |
| 10358652 | 1.855715 | 5.511987 | -5.8778 | 5.78E-06 | 4.21E-05 | Hmcn1 |
| 10420877 | -1.26424 | 7.039302 | 5.873988 | 5.84E-06 | 4.24E-05 | Esco2 |
| 10360504 | -1.18659 | 3.817131 | 5.868629 | 5.91E-06 | 4.28E-05 | Mir350 |
| 10413542 | -1.2537 | 8.673356 | 5.865819 | 5.95E-06 | 4.31E-05 | Tkt |
| 10586454 | -1.12149 | 5.589804 | 5.865045 | 5.96E-06 | 4.32E-05 | D030028M11Rik |
| 10589535 | -1.37608 | 11.38216 | 5.848097 | 6.21E-06 | 4.47E-05 | Ngp |
| 10423971 | -1.86384 | 6.705825 | 5.841121 | 6.31E-06 | 4.53E-05 | Pkhd1l1 |
| 10438415 | -1.52024 | 5.981188 | 5.841096 | 6.31E-06 | 4.53E-05 | Iglv2 |
| 10403021 | -1.80543 | 5.349707 | 5.837514 | 6.37E-06 | 4.56E-05 | Ighv1-42 |
| 10416736 | -1.01128 | 6.80248 | 5.832566 | 6.44E-06 | 4.61E-05 | Bora |
| 10366446 | -1.82018 | 8.417489 | 5.828842 | 6.50E-06 | 4.64E-05 | Tspan8 |
| 10404061 | -2.0357 | 7.914931 | 5.824304 | 6.57E-06 | 4.68E-05 | Hist1h2bb |
| 10563780 | -1.67531 | 7.496123 | 5.81755 | 6.68E-06 | 4.75E-05 | E2f8 |
| 10465587 | -1.3952 | 7.68942 | 5.808835 | 6.82E-06 | 4.83E-05 | Fermt3 |
| 10389654 | -1.13234 | 5.345822 | 5.802958 | 6.92E-06 | 4.88E-05 | Epx |
| 10358894 | -1.05425 | 8.557575 | 5.798139 | 7.00E-06 | 4.93E-05 | Sord |
| 10459866 | -2.1139 | 8.40805 | 5.79807 | 7.00E-06 | 4.93E-05 | Slc14a1 |
| 10520521 | -1.08457 | 8.266284 | 5.79764 | 7.01E-06 | 4.94E-05 | Cenpa |
| 10537410 | -1.10755 | 6.612536 | 5.793914 | 7.07E-06 | 4.98E-05 | Tbxas1 |
| 10500204 | 1.305177 | 9.18021 | -5.79306 | 7.08E-06 | 4.99E-05 | Ecm1 |
| 10420254 | -1.71254 | 7.304379 | 5.785847 | 7.21E-06 | 5.06E-05 | Mcpt8 |
| 10441787 | 1.101612 | 7.672796 | -5.78519 | 7.22E-06 | 5.07E-05 | Airn |
| 10478572 | -1.1878 | 9.226595 | 5.781082 | 7.29E-06 | 5.11E-05 | Ube2c |
| 10545086 | -1.37958 | 9.289698 | 5.779974 | 7.31E-06 | 5.12E-05 | Snca |
| 10570894 | -1.70311 | 8.367877 | 5.779877 | 7.31E-06 | 5.12E-05 | Ank1 |
| 10551666 | -1.05921 | 6.336629 | 5.778684 | 7.33E-06 | 5.13E-05 | Map4k1 |
| 10351455 | 1.147808 | 9.072288 | -5.76701 | 7.54E-06 | 5.25E-05 | Rgs5 |
| 10585276 | -1.35623 | 6.741969 | 5.766732 | 7.55E-06 | 5.25E-05 | Pou2af1 |
| 10569646 | 1.042938 | 7.782982 | -5.76402 | 7.59E-06 | 5.28E-05 | Ccnd1 |
| 10389606 | -1.18837 | 7.942527 | 5.759125 | 7.68E-06 | 5.34E-05 | Prr11 |
| 10591614 | 1.136801 | 6.792759 | -5.75884 | 7.69E-06 | 5.34E-05 | Dock6 |
| 10461979 | -1.5047 | 8.322701 | 5.754522 | 7.77E-06 | 5.38E-05 | Aldh1a1 |
| 10593198 | -1.68285 | 8.482523 | 5.752028 | 7.82E-06 | 5.41E-05 | Nxpe2 |
| 10429520 | -1.55876 | 7.323879 | 5.744432 | 7.96E-06 | 5.49E-05 | Ly6d |
| 10445758 | -1.49173 | 5.938228 | 5.73349 | 8.17E-06 | 5.62E-05 | Treml4 |
| 10358666 | 1.346921 | 6.167645 | -5.73005 | 8.24E-06 | 5.66E-05 | Hmcn1 |
| 10403034 | -1.62233 | 7.139641 | 5.726213 | 8.32E-06 | 5.70E-05 | Ighv8-8 |
| 10446425 | -1.21805 | 6.828321 | 5.725435 | 8.33E-06 | 5.71E-05 | Gm23264 |
| 10414315 | -1.02041 | 7.838518 | 5.723174 | 8.38E-06 | 5.74E-05 | Cdkn3 |
| 10466779 | -1.12506 | 7.364881 | 5.723114 | 8.38E-06 | 5.74E-05 | Pip5k1b |
| 10483046 | -1.15525 | 6.006108 | 5.713917 | 8.57E-06 | 5.85E-05 | Dpp4 |
| 10527801 | -1.05896 | 5.974087 | 5.697219 | 8.92E-06 | 6.06E-05 | Brca2 |
| 10403015 | -1.97527 | 6.93496 | 5.693754 | 8.99E-06 | 6.10E-05 | Ighv1-18 |
| 10592816 | -1.52061 | 9.241977 | 5.681649 | 9.26E-06 | 6.27E-05 | Hmbs |
| 10377405 | -1.15408 | 7.839728 | 5.680964 | 9.27E-06 | 6.28E-05 | Aurkb |
| 10358664 | 1.288276 | 5.181921 | -5.67727 | 9.35E-06 | 6.33E-05 | Hmcn1 |
| 10563883 | -1.2934 | 7.033137 | 5.67378 | 9.43E-06 | 6.37E-05 | Depdc1a |
| 10399314 | -1.74132 | 7.70531 | 5.673779 | 9.43E-06 | 6.37E-05 | Mfsd2b |
| 10544982 | -1.44395 | 8.059199 | 5.664915 | 9.64E-06 | 6.48E-05 | Nt5c3 |
| 10402864 | -1.16881 | 6.310389 | 5.660059 | 9.75E-06 | 6.55E-05 | Igh-VX24 |
| 10376444 | -1.22141 | 6.46955 | 5.65308 | 9.92E-06 | 6.64E-05 | Hist3h2ba |
| 10556244 | 1.068628 | 9.303907 | -5.64202 | 1.02E-05 | 6.80E-05 | Snora23 |
| 10497122 | -1.28323 | 7.06331 | 5.636546 | 1.03E-05 | 6.88E-05 | Depdc1a |
| 10390640 | -1.48357 | 5.426474 | 5.63508 | 1.04E-05 | 6.90E-05 | Ikzf3 |
| 10492964 | -1.22255 | 4.613327 | 5.634869 | 1.04E-05 | 6.91E-05 | Cd5l |
| 10476945 | -1.35574 | 7.343396 | 5.634066 | 1.04E-05 | 6.92E-05 | Cst7 |
| 10568369 | -1.80867 | 10.44004 | 5.627472 | 1.05E-05 | 7.01E-05 | Cox6a2 |
| 10497520 | -1.0915 | 7.005113 | 5.62564 | 1.06E-05 | 7.04E-05 | Ect2 |
| 10399710 | -1.60406 | 8.338097 | 5.62528 | 1.06E-05 | 7.05E-05 | Rsad2 |
| 10603417 | -1.52249 | 6.776492 | 5.625098 | 1.06E-05 | 7.05E-05 | Gata1 |
| 10400483 | -1.29489 | 5.66913 | 5.62412 | 1.06E-05 | 7.06E-05 | Slc25a21 |
| 10391811 | -1.57241 | 7.069444 | 5.617843 | 1.08E-05 | 7.16E-05 | Kif18b |
| 10403054 | -1.51595 | 7.534034 | 5.617018 | 1.08E-05 | 7.17E-05 | Igh-VJ558 |
| 10358577 | 1.240322 | 5.061005 | -5.60908 | 1.10E-05 | 7.29E-05 | Hmcn1 |
| 10590628 | -1.1756 | 5.89796 | 5.604096 | 1.12E-05 | 7.37E-05 | Ccr3 |
| 10554074 | 1.010572 | 4.88891 | -5.60388 | 1.12E-05 | 7.37E-05 | Adamts17 |
| 10381072 | -1.13716 | 6.425202 | 5.601336 | 1.12E-05 | 7.41E-05 | Cdc6 |
| 10351463 | 1.105238 | 9.873589 | -5.59153 | 1.15E-05 | 7.57E-05 | Rgs5 |
| 10562132 | -1.22353 | 6.066346 | 5.57476 | 1.20E-05 | 7.85E-05 | Cd22 |
| 10595094 | 1.275495 | 7.845368 | -5.56578 | 1.22E-05 | 8.00E-05 | Mlip |
| 10504957 | -1.00428 | 8.252552 | 5.564837 | 1.23E-05 | 8.02E-05 | Smc2 |
| 10539818 | -2.22532 | 7.753017 | 5.562701 | 1.23E-05 | 8.05E-05 | Gp9 |
| 10507500 | -1.20783 | 7.247536 | 5.55591 | 1.25E-05 | 8.17E-05 | Slc6a9 |
| 10349593 | -1.12644 | 6.15348 | 5.553446 | 1.26E-05 | 8.21E-05 | Faim3 |
| 10575702 | 1.881881 | 6.034436 | -5.54961 | 1.27E-05 | 8.28E-05 | Clec3a |
| 10358561 | 1.274235 | 4.841653 | -5.54453 | 1.29E-05 | 8.37E-05 | Hmcn1 |
| 10408210 | -1.05031 | 11.1842 | 5.536523 | 1.31E-05 | 8.51E-05 | Hist1h2bf |
| 10561920 | -1.46769 | 8.315731 | 5.532786 | 1.33E-05 | 8.58E-05 | Hcst |
| 10403943 | -1.69363 | 9.982086 | 5.509462 | 1.40E-05 | 9.02E-05 | Hist1h2bm |
| 10473367 | -1.56675 | 8.71165 | 5.508947 | 1.40E-05 | 9.03E-05 | Slc43a1 |
| 10566580 | -1.39775 | 5.213466 | 5.505894 | 1.41E-05 | 9.08E-05 | Gm4759 |
| 10598041 | -1.51832 | 7.899754 | 5.502754 | 1.43E-05 | 9.14E-05 | mt-Tk |
| 10487480 | -1.00699 | 7.467085 | 5.493898 | 1.46E-05 | 9.30E-05 | Bub1 |
| 10492021 | 1.421532 | 10.03023 | -5.49108 | 1.47E-05 | 9.36E-05 | Postn |
| 10495763 | -1.26635 | 8.462291 | 5.478752 | 1.51E-05 | 9.60E-05 | Gclm |
| 10598152 | -1.08437 | 5.84664 | 5.472501 | 1.53E-05 | 9.73E-05 | BC147527 |
| 10408081 | -1.30193 | 10.10032 | 5.470714 | 1.54E-05 | 9.77E-05 | Hist1h1b |
| 10438064 | -1.66978 | 7.949584 | 5.46362 | 1.57E-05 | 9.91E-05 | Vpreb1 |
| 10358631 | 1.439535 | 5.515454 | -5.45835 | 1.59E-05 | 0.0001 | Hmcn1 |
| 10398907 | -1.33047 | 6.768169 | 5.453644 | 1.61E-05 | 0.000101 | Pld4 |
| 10558919 | -1.41201 | 7.63655 | 5.453507 | 1.61E-05 | 0.000101 | Snora52 |
| 10553833 | 1.069458 | 5.074612 | -5.45122 | 1.62E-05 | 0.000102 | Ndn |
| 10471721 | -1.26684 | 7.425317 | 5.442661 | 1.65E-05 | 0.000103 | Ptgs1 |
| 10592330 | -1.1224 | 7.007449 | 5.432171 | 1.69E-05 | 0.000106 | Nrgn |
| 10473406 | -1.10342 | 5.61256 | 5.431699 | 1.69E-05 | 0.000106 | Prg3 |
| 10427904 | 1.0799 | 5.195698 | -5.42961 | 1.70E-05 | 0.000106 | Fbxl7 |
| 10474381 | -1.26856 | 7.38861 | 5.427723 | 1.71E-05 | 0.000106 | Kif18a |
| 10371662 | -1.63353 | 5.85973 | 5.423987 | 1.73E-05 | 0.000107 | Spic |
| 10486664 | -1.81732 | 8.270558 | 5.422126 | 1.73E-05 | 0.000108 | Epb4.2 |
| 10357590 | -1.13505 | 7.019822 | 5.421283 | 1.74E-05 | 0.000108 | Dyrk3 |
| 10598586 | -1.12491 | 6.516694 | 5.421054 | 1.74E-05 | 0.000108 | Xk |
| 10403031 | -1.89029 | 6.951688 | 5.394343 | 1.85E-05 | 0.000114 | Ighv1-55 |
| 10506822 | -1.14261 | 5.765866 | 5.388611 | 1.88E-05 | 0.000116 | Orc1 |
| 10583529 | -1.23977 | 6.421631 | 5.377694 | 1.93E-05 | 0.000119 | Icam4 |
| 10361375 | -1.04474 | 8.661696 | 5.37389 | 1.95E-05 | 0.00012 | Fbxo5 |
| 10461614 | -1.03643 | 8.436665 | 5.369816 | 1.97E-05 | 0.000121 | Ms4a6c |
| 10545184 | -2.22831 | 8.14059 | 5.369148 | 1.97E-05 | 0.000121 | Igkv4-74 |
| 10554240 | -1.55726 | 8.633933 | 5.34661 | 2.08E-05 | 0.000127 | Isg20 |
| 10551025 | -1.79276 | 5.986565 | 5.345131 | 2.09E-05 | 0.000127 | Cd79a |
| 10463355 | 1.271598 | 6.936988 | -5.32976 | 2.17E-05 | 0.000132 | Scd2 |
| 10376434 | -1.57455 | 7.920473 | 5.322658 | 2.21E-05 | 0.000134 | Btnl10 |
| 10403948 | -1.12236 | 11.30159 | 5.319183 | 2.23E-05 | 0.000135 | Hist1h2bn |
| 10568714 | -1.0639 | 10.08379 | 5.318597 | 2.23E-05 | 0.000135 | Mki67 |
| 10408077 | -1.03005 | 8.50927 | 5.316748 | 2.24E-05 | 0.000135 | Hist1h2ak |
| 10443749 | -1.01248 | 5.150636 | 5.315742 | 2.25E-05 | 0.000135 | Ubash3a |
| 10438738 | -1.06218 | 7.418864 | 5.304071 | 2.31E-05 | 0.000139 | Bcl6 |
| 10513608 | -1.26885 | 8.471623 | 5.302586 | 2.32E-05 | 0.000139 | Alad |
| 10531177 | 1.104573 | 5.466703 | -5.3023 | 2.32E-05 | 0.000139 | Adamts3 |
| 10538882 | -1.83175 | 5.505989 | 5.301359 | 2.33E-05 | 0.00014 | Igkv9-124 |
| 10541729 | -1.03097 | 9.370509 | 5.284812 | 2.42E-05 | 0.000145 | Cdca3 |
| 10592772 | -1.16562 | 6.971553 | 5.280331 | 2.45E-05 | 0.000146 | Abcg4 |
| 10481304 | -1.54662 | 8.102548 | 5.275725 | 2.48E-05 | 0.000147 | Gfi1b |
| 10417887 | -1.45215 | 7.623069 | 5.261321 | 2.56E-05 | 0.000152 | Mss51 |
| 10442932 | -1.19116 | 6.624602 | 5.261298 | 2.57E-05 | 0.000152 | Tmem8 |
| 10559606 | -1.25091 | 5.999416 | 5.251568 | 2.63E-05 | 0.000155 | Tmem86b |
| 10449163 | -1.06521 | 8.97419 | 5.246378 | 2.66E-05 | 0.000157 | Pigq |
| 10358656 | 1.042996 | 5.603151 | -5.24007 | 2.70E-05 | 0.000159 | Hmcn1 |
| 10399391 | -1.10032 | 6.701796 | 5.238729 | 2.71E-05 | 0.000159 | Gen1 |
| 10403980 | -1.14534 | 11.49541 | 5.23786 | 2.72E-05 | 0.00016 | Hist1h2bq |
| 10487011 | -1.06492 | 6.989929 | 5.234981 | 2.74E-05 | 0.000161 | Gatm |
| 10406968 | -1.16735 | 7.416841 | 5.226987 | 2.79E-05 | 0.000163 | Cenpk |
| 10435504 | -1.07021 | 4.280166 | 5.225423 | 2.80E-05 | 0.000164 | Gm5416 |
| 10425321 | -1.07087 | 8.518102 | 5.209028 | 2.91E-05 | 0.000169 | Apobec3 |
| 10403978 | -1.1609 | 11.32657 | 5.199667 | 2.98E-05 | 0.000173 | Hist1h2bq |
| 10560190 | 1.008467 | 6.565426 | -5.18741 | 3.07E-05 | 0.000178 | Ehd2 |
| 10584710 | -1.12184 | 9.335975 | 5.17716 | 3.15E-05 | 0.000181 | H2afx |
| 10478884 | 1.144723 | 6.774704 | -5.17259 | 3.19E-05 | 0.000183 | Snai1 |
| 10400589 | -1.12716 | 7.079457 | 5.166011 | 3.24E-05 | 0.000186 | Mis18bp1 |
| 10391649 | -1.73518 | 10.12309 | 5.165697 | 3.24E-05 | 0.000186 | Slc4a1 |
| 10594513 | 1.484448 | 5.809794 | -5.16386 | 3.26E-05 | 0.000187 | Gm25313 |
| 10408070 | -1.1429 | 11.38162 | 5.156945 | 3.31E-05 | 0.000189 | Hist1h2bq |
| 10562563 | -1.058 | 7.5182 | 5.153922 | 3.34E-05 | 0.000191 | Ccne1 |
| 10537770 | -1.13576 | 8.590536 | 5.147916 | 3.38E-05 | 0.000193 | Zyx |
| 10545190 | -2.58485 | 5.789866 | 5.143292 | 3.42E-05 | 0.000195 | Igkv4-69 |
| 10563338 | -1.38135 | 7.94981 | 5.121434 | 3.61E-05 | 0.000204 | Ppp1r15a |
| 10576757 | -1.50208 | 5.129865 | 5.107232 | 3.74E-05 | 0.00021 | Fcer2a |
| 10582821 | -1.75102 | 10.85185 | 5.094801 | 3.86E-05 | 0.000215 | Gm26397 |
| 10403018 | -1.96975 | 7.283 | 5.090473 | 3.90E-05 | 0.000217 | Igh-VJ558 |
| 10384956 | -1.04567 | 7.501624 | 5.083933 | 3.96E-05 | 0.00022 | Chac2 |
| 10379363 | -1.00442 | 6.641738 | 5.077078 | 4.03E-05 | 0.000224 | Atad5 |
| 10409014 | -1.67924 | 8.827816 | 5.067536 | 4.12E-05 | 0.000228 | Gm24620 |
| 10404731 | -1.09039 | 9.026397 | 5.067195 | 4.13E-05 | 0.000228 | Tmem14c |
| 10515836 | -1.05357 | 7.685835 | 5.063956 | 4.16E-05 | 0.00023 | Ccnb1 |
| 10509002 | -1.87282 | 8.754903 | 5.055588 | 4.25E-05 | 0.000234 | Rhd |
| 10573054 | -1.79624 | 10.02656 | 5.051742 | 4.29E-05 | 0.000236 | Gypa |
| 10434743 | -1.72019 | 10.88166 | 5.028606 | 4.54E-05 | 0.000248 | Gm26447 |
| 10504757 | -1.04803 | 4.908819 | 5.017594 | 4.66E-05 | 0.000254 | BC005685 |
| 10507872 | -1.50496 | 8.530234 | 5.002761 | 4.83E-05 | 0.000262 | Gm25788 |
| 10363856 | -1.30922 | 8.345178 | 5.002757 | 4.83E-05 | 0.000262 | 2310015B20Rik |
| 10444284 | -1.1764 | 5.911821 | 5.001183 | 4.85E-05 | 0.000263 | H2-Ob |
| 10460237 | -1.03079 | 7.862428 | 4.977277 | 5.15E-05 | 0.000277 | Unc93b1 |
| 10563112 | 1.487696 | 7.874196 | -4.9677 | 5.27E-05 | 0.000283 | Snord33 |
| 10412559 | -1.02884 | 7.486224 | 4.963994 | 5.32E-05 | 0.000285 | Slbp |
| 10511363 | 1.137706 | 7.461129 | -4.95586 | 5.42E-05 | 0.00029 | Penk |
| 10573457 | -1.29705 | 7.180857 | 4.934781 | 5.71E-05 | 0.000301 | Klf1 |
| 10497345 | -1.15915 | 6.063095 | 4.93285 | 5.74E-05 | 0.000302 | Gm9733 |
| 10512757 | -1.58711 | 8.744762 | 4.931234 | 5.76E-05 | 0.000303 | Hemgn |
| 10547985 | -1.14048 | 5.426113 | 4.928774 | 5.80E-05 | 0.000305 | Cd27 |
| 10507870 | -1.54941 | 8.801845 | 4.928111 | 5.81E-05 | 0.000305 | Gm24620 |
| 10586907 | -1.06719 | 6.98929 | 4.9158 | 5.99E-05 | 0.000313 | Mns1 |
| 10587871 | -1.15389 | 6.333896 | 4.903675 | 6.17E-05 | 0.000322 | Paqr9 |
| 10545187 | -2.32443 | 7.291056 | 4.902238 | 6.19E-05 | 0.000323 | Igkv4-70 |
| 10444223 | -1.18542 | 6.042277 | 4.884601 | 6.47E-05 | 0.000336 | H2-Oa |
| 10545173 | -1.98169 | 6.882691 | 4.873676 | 6.64E-05 | 0.000344 | Igkv10-96 |
| 10466172 | -1.58512 | 6.952172 | 4.861252 | 6.85E-05 | 0.000353 | Ms4a1 |
| 10394978 | -1.45327 | 8.792327 | 4.854936 | 6.96E-05 | 0.000358 | Rrm2 |
| 10351509 | -1.15166 | 6.13801 | 4.846694 | 7.10E-05 | 0.000364 | Fcgr4 |
| 10531737 | -1.07561 | 6.459232 | 4.828783 | 7.42E-05 | 0.000379 | Hpse |
| 10410984 | -1.65266 | 7.945097 | 4.823133 | 7.52E-05 | 0.000384 | Ckmt2 |
| 10359861 | -1.18179 | 9.680897 | 4.807418 | 7.82E-05 | 0.000397 | Mgst3 |
| 10582464 | -1.46577 | 6.016634 | 4.802261 | 7.92E-05 | 0.000401 | Gm24445 |
| 10582916 | -1.04744 | 4.029381 | 4.770125 | 8.58E-05 | 0.00043 | Gm17535 |
| 10550877 | -1.20952 | 7.33723 | 4.766853 | 8.64E-05 | 0.000433 | Kcnn4 |
| 10351293 | 1.267601 | 7.905181 | -4.75622 | 8.87E-05 | 0.000442 | Dpt |
| 10362426 | -1.66795 | 6.643025 | 4.747249 | 9.07E-05 | 0.00045 | Trdn |
| 10403073 | -1.06988 | 3.979726 | 4.742375 | 9.18E-05 | 0.000455 | Ighv1-84 |
| 10436024 | -1.17979 | 5.853459 | 4.740413 | 9.23E-05 | 0.000457 | Gcsam |
| 10469255 | -1.14075 | 6.901174 | 4.732904 | 9.40E-05 | 0.000464 | Prkcq |
| 10425037 | -1.76039 | 8.059811 | 4.714027 | 9.85E-05 | 0.000483 | Apol10a |
| 10501555 | -1.16836 | 6.794478 | 4.709306 | 9.97E-05 | 0.000488 | Amy1 |
| 10408083 | -1.04447 | 11.1731 | 4.700704 | 0.000102 | 0.000496 | Hist1h3a |
| 10404028 | -1.01681 | 11.17933 | 4.692133 | 0.000104 | 0.000506 | Hist1h3f |
| 10538871 | -1.59007 | 6.751279 | 4.690005 | 0.000105 | 0.000508 | Igkv1-135 |
| 10354267 | -1.23302 | 6.425192 | 4.678097 | 0.000108 | 0.000522 | Mettl21c |
| 10458278 | -1.16615 | 5.749934 | 4.670361 | 0.00011 | 0.000531 | Mzb1 |
| 10408246 | -1.00111 | 11.17941 | 4.650789 | 0.000115 | 0.000554 | Hist1h3f |
| 10485982 | 1.39504 | 6.967421 | -4.64761 | 0.000116 | 0.000557 | Actc1 |
| 10427628 | -1.42944 | 6.120833 | 4.643084 | 0.000117 | 0.000563 | Il7r |
| 10545220 | -1.58539 | 4.871625 | 4.619357 | 0.000124 | 0.000593 | Igkv12-41 |
| 10499363 | 1.032526 | 9.71933 | -4.61869 | 0.000125 | 0.000593 | Bglap |
| 10501802 | -1.21329 | 6.395718 | 4.60484 | 0.000129 | 0.000613 | Tmem56 |
| 10545215 | -2.31198 | 6.005364 | 4.603881 | 0.000129 | 0.000614 | Igkv12-46 |
| 10481845 | -1.15818 | 6.884855 | 4.602913 | 0.00013 | 0.000615 | Mvb12b |
| 10544588 | -1.54412 | 5.255325 | 4.591868 | 0.000133 | 0.00063 | Gimap3 |
| 10538903 | -1.25795 | 6.01501 | 4.588453 | 0.000134 | 0.000635 | Igk |
| 10375123 | -1.19519 | 7.818149 | 4.584263 | 0.000136 | 0.00064 | C530030P08Rik |
| 10504755 | -1.73441 | 7.23212 | 4.561104 | 0.000144 | 0.000673 | Sympk |
| 10557591 | -1.21191 | 6.839316 | 4.555194 | 0.000146 | 0.000682 | Itgal |
| 10598507 | -1.34862 | 6.664519 | 4.550092 | 0.000148 | 0.000689 | Slc38a5 |
| 10587778 | 1.239793 | 7.44487 | -4.5385 | 0.000152 | 0.000707 | Gm22866 |
| 10404049 | -1.03232 | 11.28512 | 4.523256 | 0.000158 | 0.000731 | Hist1h3a |
| 10545208 | -1.28158 | 6.497557 | 4.515628 | 0.000161 | 0.000744 | Igkv4-57 |
| 10377429 | 1.046695 | 8.603635 | -4.50585 | 0.000165 | 0.00076 | Snord118 |
| 10399428 | 1.046695 | 8.603635 | -4.50585 | 0.000165 | 0.00076 | Snord118 |
| 10451953 | -1.62281 | 9.113785 | 4.498547 | 0.000168 | 0.000772 | Lrg1 |
| 10601648 | 1.066987 | 7.231584 | -4.49779 | 0.000168 | 0.000773 | Tnmd |
| 10502552 | -1.30301 | 5.516624 | 4.494959 | 0.000169 | 0.000778 | Clca1 |
| 10408239 | -1.03234 | 11.23503 | 4.482321 | 0.000175 | 0.0008 | Hist1h3a |
| 10574572 | -1.39411 | 7.690507 | 4.474818 | 0.000178 | 0.000813 | Ces2g |
| 10534389 | -1.61734 | 8.639704 | 4.462025 | 0.000184 | 0.000836 | Cldn13 |
| 10404065 | -1.00933 | 11.28046 | 4.45646 | 0.000186 | 0.000846 | Hist1h3f |
| 10545196 | -1.74369 | 8.282094 | 4.450554 | 0.000189 | 0.000856 | Igkv4-61 |
| 10434860 | -1.3284 | 6.779651 | 4.446529 | 0.000191 | 0.000864 | Ostn |
| 10413229 | -1.02849 | 7.71989 | 4.425662 | 0.000201 | 0.000903 | Anxa11 |
| 10530783 | -1.00496 | 6.178452 | 4.413892 | 0.000207 | 0.000926 | A730089K16Rik |
| 10375121 | -1.21458 | 7.884138 | 4.398759 | 0.000215 | 0.000957 | C530030P08Rik |
| 10545177 | -1.62322 | 6.343181 | 4.391574 | 0.000219 | 0.000972 | Igkv19-93 |
| 10438405 | -1.30496 | 8.152898 | 4.382262 | 0.000224 | 0.000991 | Iglv1 |
| 10545210 | -1.66179 | 5.842654 | 4.378463 | 0.000226 | 0.001 | Igkv4-55 |
| 10349648 | -1.33462 | 10.39619 | 4.370268 | 0.000231 | 0.001017 | Ctse |
| 10405047 | 1.02412 | 10.5687 | -4.36847 | 0.000232 | 0.001021 | Aspn |
| 10545194 | -1.51027 | 5.50574 | 4.354663 | 0.00024 | 0.001051 | Igkv4-62 |
| 10566132 | -1.18906 | 8.668992 | 4.349987 | 0.000243 | 0.001061 | Rhog |
| 10436392 | -1.00362 | 8.908032 | 4.336552 | 0.000251 | 0.001092 | Cpox |
| 10533213 | -1.22946 | 6.039896 | 4.327129 | 0.000257 | 0.001115 | Oas3 |
| 10494411 | 1.209933 | 7.607077 | -4.32279 | 0.000259 | 0.001125 | Rnu1b1 |
| 10494417 | 1.209933 | 7.607077 | -4.32279 | 0.000259 | 0.001125 | Rnu1b1 |
| 10500356 | 1.209933 | 7.607077 | -4.32279 | 0.000259 | 0.001125 | Rnu1b1 |
| 10445192 | -1.68398 | 8.697727 | 4.320113 | 0.000261 | 0.001132 | Rhag |
| 10513945 | -1.21898 | 7.023462 | 4.295162 | 0.000278 | 0.001196 | 2310002L09Rik |
| 10384015 | -1.17978 | 10.3078 | 4.292769 | 0.000279 | 0.001201 | Pgam2 |
| 10482030 | -1.03932 | 8.861736 | 4.288202 | 0.000283 | 0.001213 | Stom |
| 10522653 | -1.2847 | 6.293632 | 4.278959 | 0.000289 | 0.001238 | A730089K16Rik |
| 10384373 | -1.02536 | 7.800808 | 4.250116 | 0.000311 | 0.001317 | Fignl1 |
| 10559649 | -1.19852 | 7.227951 | 4.246616 | 0.000313 | 0.001326 | Cox6b2 |
| 10494405 | -1.02606 | 11.13974 | 4.219721 | 0.000335 | 0.001403 | Hist2h3c2 |
| 10371082 | -1.29575 | 7.264812 | 4.197261 | 0.000354 | 0.001475 | Nmrk2 |
| 10430372 | -1.24666 | 9.817704 | 4.182245 | 0.000367 | 0.001525 | Rac2 |
| 10545231 | -1.64925 | 6.63515 | 4.143021 | 0.000405 | 0.001659 | Igkv6-32 |
| 10358565 | 1.026496 | 4.189185 | -4.14116 | 0.000407 | 0.001666 | Hmcn1 |
| 10403079 | -1.32502 | 6.707273 | 4.130731 | 0.000417 | 0.001703 | LOC435333 |
| 10551347 | -1.15698 | 9.224083 | 4.087612 | 0.000464 | 0.001871 | Blvrb |
| 10355325 | -1.00852 | 6.449541 | 3.930778 | 0.000684 | 0.002632 | Bard1 |
| 10494413 | 1.162421 | 7.789798 | -3.9242 | 0.000695 | 0.00267 | Rnu1b1 |
| 10494421 | 1.162421 | 7.789798 | -3.9242 | 0.000695 | 0.00267 | Rnu1b1 |
| 10500343 | 1.162421 | 7.789798 | -3.9242 | 0.000695 | 0.00267 | Rnu1b1 |
| 10500358 | 1.162421 | 7.789798 | -3.9242 | 0.000695 | 0.00267 | Rnu1b1 |
| 10512937 | 1.162421 | 7.789798 | -3.9242 | 0.000695 | 0.00267 | Rnu1b1 |
| 10490923 | -1.04224 | 10.74519 | 3.91471 | 0.000711 | 0.002723 | Car2 |
| 10532744 | -1.02302 | 8.941066 | 3.882549 | 0.00077 | 0.002922 | Selplg |
| 10425852 | -1.13766 | 8.788035 | 3.872013 | 0.00079 | 0.002991 | Parvb |
| 10405058 | 1.105478 | 7.789553 | -3.86579 | 0.000802 | 0.003032 | Omd |
| 10366705 | -1.00607 | 7.950536 | 3.851926 | 0.00083 | 0.003125 | Gm9081 |
| 10545212 | -1.26035 | 4.950758 | 3.792488 | 0.00096 | 0.003544 | Igkv5-48 |
| 10497337 | -1.11105 | 10.95184 | 3.780583 | 0.000989 | 0.003632 | Car1 |
| 10545198 | -1.775 | 8.344824 | 3.75065 | 0.001064 | 0.003868 | Igkv4-59 |
| 10538921 | -1.30742 | 8.834545 | 3.728506 | 0.001124 | 0.004058 | Igkv1-117 |
| 10408543 | -1.40701 | 7.566945 | 3.710053 | 0.001175 | 0.004218 | Mylk4 |
| 10403069 | -1.29604 | 5.037927 | 3.708019 | 0.001181 | 0.004235 | Igh-VJ558 |
| 10582879 | -1.06706 | 6.346114 | 3.707697 | 0.001182 | 0.004237 | Csprs |
| 10582162 | -1.06127 | 8.442016 | 3.707527 | 0.001183 | 0.004238 | Cotl1 |
| 10552406 | -1.33737 | 8.30051 | 3.64709 | 0.001371 | 0.004818 | Nkg7 |
| 10497752 | -1.0668 | 7.559175 | 3.583079 | 0.001603 | 0.005531 | Gm9791 |
| 10538880 | -1.53029 | 6.690256 | 3.553196 | 0.001723 | 0.0059 | Igkv1-117 |
| 10538924 | -1.0068 | 6.799591 | 3.547074 | 0.001749 | 0.005975 | Igkv2-109 |
| 10545247 | -1.8617 | 6.6136 | 3.492399 | 0.001997 | 0.006687 | Igkv6-14 |
| 10494160 | -1.07102 | 8.116007 | 3.46126 | 0.002154 | 0.007151 | Tmod4 |
| 10362442 | -1.17883 | 8.56524 | 3.407312 | 0.002453 | 0.007994 | Trdn |
| 10494873 | -1.05284 | 9.276021 | 3.288806 | 0.003262 | 0.010286 | Ampd1 |
| 10455461 | -1.16375 | 9.569347 | 3.285157 | 0.00329 | 0.010367 | Myot |
| 10485357 | -1.28035 | 8.433369 | 3.232056 | 0.003735 | 0.01155 | Gm10800 |
| 10543233 | -1.08732 | 7.161213 | 3.168352 | 0.004346 | 0.013168 | Ppp1r3a |
| 10551966 | -1.11648 | 9.414615 | 3.081577 | 0.005335 | 0.015754 | Hspb6 |
| 10347222 | -1.00982 | 10.24417 | 3.066095 | 0.005533 | 0.016249 | Gm23444 |
| 10582582 | 1.043806 | 7.19862 | -2.93061 | 0.007594 | 0.021379 | Gm24089 |
| 10582584 | 1.043806 | 7.19862 | -2.93061 | 0.007594 | 0.021379 | Gm24089 |
| 10362436 | -1.08814 | 7.245008 | 2.91153 | 0.007937 | 0.022211 | Trdn |
| 10466127 | -1.0333 | 8.574006 | 2.896377 | 0.008221 | 0.022891 | AW112010 |
| 10379535 | 1.315735 | 6.067711 | -2.88169 | 0.008504 | 0.02359 | Ccl8 |
| 10362462 | -1.0031 | 5.587495 | 2.841759 | 0.009324 | 0.025549 | Trdn |
| 10362456 | -1.07829 | 6.397344 | 2.573853 | 0.017092 | 0.042376 | Trdn |
| 10445112 | -1.01846 | 5.983699 | 2.32085 | 0.029667 | 0.067369 | Ubd |
